# Novel MicroRNA Candidates and miRNA-mRNA Pairs in Embryonic Stem (ES) Cells

**DOI:** 10.1371/journal.pone.0002548

**Published:** 2008-07-02

**Authors:** Peili Gu, Jeffrey G. Reid, Xiaolian Gao, Chad A. Shaw, Chad Creighton, Peter L. Tran, Xiaochuan Zhou, Rafal B. Drabek, David L. Steffen, David M. Hoang, Michelle K. Weiss, Arash O. Naghavi, Jad El-daye, Mahjabeen F. Khan, Glen B. Legge, David A. Wheeler, Richard A. Gibbs, Jonathan N. Miller, Austin J. Cooney, Preethi H. Gunaratne

**Affiliations:** 1 Department of Biology & Biochemistry, University of Houston, Houston, Texas, United States of America; 2 Department of Chemistry, University of Houston, Houston, Texas, United States of America; 3 Department of Biochemistry & Molecular Biology, Baylor College of Medicine, Houston, Texas, United States of America; 4 Department of Pathology, Baylor College of Medicine, Houston, Texas, United States of America; 5 Department of Molecular & Cellular Biology, Baylor College of Medicine, Houston, Texas, United States of America; 6 Department of Human Genome Sequencing Center, Baylor College of Medicine, Houston, Texas, United States of America; 7 Department of Molecular & Human Genetics, Baylor College of Medicine, Houston, Texas, United States of America; 8 Bioinformatics Research Center, Baylor College of Medicine, Houston, Texas, United States of America; 9 Duncan Cancer Center, Baylor College of Medicine, Houston, Texas, United States of America; 10 W. M. Keck Center for Interdisciplinary Bioscience Training, Houston, Texas, United States of America; 11 LC Sciences, Houston, Texas, United States of America; 12 Department of Cancer Genetics, M.D. Anderson Cancer Center, University of Texas , Houston, Texas, United States of America; Columbia University, United States of America

## Abstract

**Background:**

MicroRNAs (miRNAs: a class of short non-coding RNAs) are emerging as important agents of post transcriptional gene regulation and integral components of gene networks. MiRNAs have been strongly linked to stem cells, which have a remarkable dual role in development. They can either continuously replenish themselves (self-renewal), or differentiate into cells that execute a limited number of specific actions (pluripotence).

**Methodology/Principal Findings:**

In order to identify novel miRNAs from narrow windows of development we carried out an *in silico* search for micro-conserved elements (MCE) in adult tissue progenitor transcript sequences. A plethora of previously unknown miRNA candidates were revealed including 545 small RNAs that are enriched in embryonic stem (ES) cells over adult cells. Approximately 20% of these novel candidates are down-regulated in ES (*Dicer* ^−/−^) ES cells that are impaired in miRNA maturation. The ES-enriched miRNA candidates exhibit distinct and opposite expression trends from mmu-mirs (an abundant class in adult tissues) during retinoic acid (RA)-induced ES cell differentiation. Significant perturbation of trends is found in both miRNAs and novel candidates in ES (G*CNF* ^−/−^) cells, which display loss of repression of pluripotence genes upon differentiation.

**Conclusion/Significance:**

Combining expression profile information with miRNA target prediction, we identified miRNA-mRNA pairs that correlate with ES cell pluripotence and differentiation. Perturbation of these pairs in the ES (G*CNF* ^−/−^) mutant suggests a role for miRNAs in the core regulatory networks underlying ES cell self-renewal, pluripotence and differentiation.

## Introduction

Maintaining the balance between self-renewal and differentiation and the ability to switch from one state to the other is fundamental to both embryonic stem cell plasticity and developmental re-programming of mature cells during tissue homeostasis and injury [1]–[2]. ES cells derived from the inner cell mass (ICM) of the mammalian blastocyst provide an ideal model for studying this transition. ES cell pluripotence is regulated both by extrinsic signaling pathways and by intrinsic gene regulatory mechanisms [1]–[12] involving a network of transcription factors including *Oct4*, *Sox2*, *Nanog*, *Tbx3*, *Essrb*, *Dppa4*, *Tcl1*, *Klf4* and *cMyc* [3]–[14]. The active repression of genes that induce and maintain differentiation is also essential for this state and is primarily mediated by Polycomb group (PcG) repressor proteins. PcG repressor complexes cooperate with *Oct4* and *Nanog* to silence several hundred developmental regulators [10]–[11]. This silencing is achieved through histone H3 Lysine tri-methylation (H3Kme3) and the establishment of bi-valent domains of active H3K4me3 and inactive H3K27me3 [15]. The orphan nuclear receptor *GCNF* (germ cell nuclear factor) or *NR6A1* (nuclear receptor 6A1) is the best characterized transcriptional repressor of *Oct4* and *Nanog* [16]. *GCNF* is widely expressed in early mouse embryos [17]–[18] and transiently expressed in P19 EC cells and ES cells during RA-induced differentiation (Day 1–3 of RA-induction) [16]. ES (G*CNF^−/−^*) embryos die around E10.5 and fail to properly repress *Oct4* or to restrict its expression to primordial germ cells after gastrulation [16]. All of the key genes involved in ES cell pluripotence, self-renewal and differentiation contain predicted miRNA target sites in their 3′-UTR regions.

MicroRNAs are widespread agents of post-transcriptional silencing and have been strongly linked with stem cells [19]–[25]. In mammals *Dicer* ^−/−^ embryos die on embryonic day 7.5 and ES (*Dicer* ^−/−^) cells are characterized by defects in proliferation, miRNA maturation, and failure to differentiate [19]–[22]. Recently, pyrosequencing of small RNAs isolated from ES and ES (*Dicer* ^−/−^) revealed 46 novel miRNAs among over 110,000 miRNA transcripts per ES cell [26], of which more than 75% were accounted for by 6 distinct loci in the mouse genome [26].

MiRNAs exhibit a high degree of stage- and tissue-specificity, and therefore it is likely that those that operate during narrow windows of development may be under-represented in the current databases. We therefore executed an exhaustive search for novel miRNA candidates expressed from tissue progenitor transcripts based on ‘micro-conservation’ – perfect conservation of 20–50 nucleotide (nt) sequences among diverse multiple genomes [27]–[28]. We limited our search to mammalian genomes because segregation of the pluripotent germ cell lineage in lower organisms and in species like *Xenopus* and Zebrafish is distinctly different from what has evolved in mammals. In lower organisms the germ cell lineage is designated after the first cleavage; however, in mammals the germ cell lineage is segregated relatively late in development, peri-gastrulation [29]. This adaptation to the uterine environment and implantation means that pluripotence has to be maintained up to that stage throughout the early embryo.

Using this strategy we uncovered ∼4600 novel candidates (MCE-MIR-micro-conserved element miRNA prediction) of which 545 were found to be expressed in the small RNA fraction of ES cells. The majority exhibited dynamic expression patterns during RA-induced differentiation on Day 1, Day 3 and Day 6. Approximately, 100 MCE-MIRs expressed in ES cells were decreased in ES (*Dicer* ^−/−^) mutants and are thus likely to be genuine miRNAs. Given that three of the self-renewal regulators are directly (*Oct4* and *Nanog*) or indirectly (*Sox2*) under *GCNF* regulation, we further characterized the miRNA and novel candidates in the ES (G*CNF* ^−/−^) cells. We have also generated mRNA expression profiles in ES and on Day 3 and Day 6 of RA-induced differentiation (RA-D3 and D6). From this work we uncovered three classes of miRNAs that correlate with pluripotence/self-renewal, differentiation, and the transition from one state to the other. Using miRNA target prediction programs in combination with target enrichment analyses we have identified miRNA-mRNA pairs that may be central to ES self-renewal and differentiation.

## Results

### Transcriptome-wide search in tissue progenitor sequences reveals novel miRNA candidates

To identify novel miRNAs involved in the maturation of adult stem cells and tissue progenitors, we undertook an intensive search for candidates in the Stem Cell Genome Anatomy Project (SCGAP) sequence data. Our SCGAP source sequences were derived from mouse and included transcripts from hematopoietic stem cells (SCDb, Hematopietic Stem Cell-Side Population (HSC-SP) HSC-SP-Quiescent, HSC-SP-Activated), the stromal cell microenvironment (StroCDb), whole bone marrow (WBM), bi-potential murine embryonic liver stem cells (BMEL), small intestine epithelial (SiEP) and gastric epithelial progenitors (GEP) (Figure S1).

**Figure 1:**
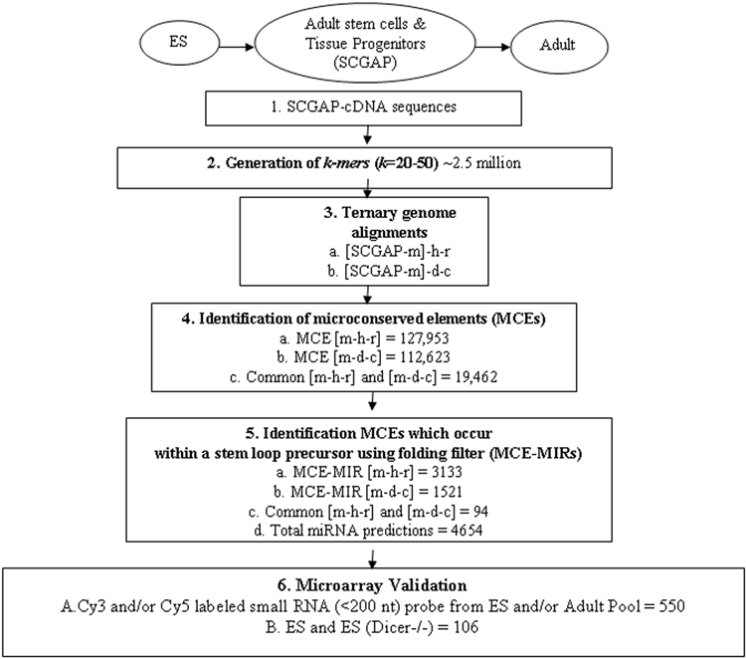
Novel microRNA dicovery pipeline and yields. This flow chart summarizes the yields from SCGAP consortium sequences using a k-mer based miRNA prediction algorithm described Tran et al. (25). The SCGAP sequence composition is described in Figure S1 and the strategy used is described in Figure S2 and the Methods section.

Our miRNA discovery paradigm is distinct from others in that: 1) it is based on transcripts from biological samples; and 2) it is aimed at short (<50 bp) sequences that are perfectly conserved (micro-conserved) among multiple organisms [27]–[28]. Through our exhaustive transcriptome-wide search we obtained ∼120,000 microconserved elements (MCEs). Approximately, 4,600 MCEs (i) formed a sufficiently low-energy stem-loop secondary structure; and (ii) spanned just one strand of the stem [29] and were selected as novel miRNA candidates (MCE-MIRs). Our miRNA discovery strategy, pipeline and yields are shown in Figure 1, Figures S2 and Supplemental Tables S1–S2. A total of ∼4600 distinct MCE-MIRs, of which only ∼100 are common to both intersections, were identified from the SCGAP sequences. Approximately 10% of the MCE-MIRs hairpins had a subsequence similar to previously cloned or predicted miRNAs or their reverse complements (Supplemental Table S3). Four hundred and twenty four (424) MCE-MIR hairpins overlapped with functional RNA structures (fRNAs) obtained by a new algorithm (EvoFold) designed to identify conserved RNA secondary structures in the human genome [30]. Some MCE-MIRs were similar to mmu-mirs, piRNAs and other predictions [26], [31], [32].

To discover miRNAs that are common to embryonic and adult stem cells we applied a high-throughput screening procedure that employed a custom designed miRNA microarray [Mouse Array Version 1 - Figure S3] printed with a comprehensive panel of miRNAs in miRBase version 7.1 (238 mmu-miRs) together with 2617 MCE-MIR sequences, 321 ‘Cand’ sequences predicted by phylogenetic-shadowing [32], and 129 ‘MIR’ sequences discovered by searching for miRNA targets in 3′ UTR sequences [33]. This custom array was probed with the small-RNA (<200 nt) fractions of ES cells (ES) and with RNA pooled from 18 different adult tissues (Adult Pool) in order to identify MCE-MIRs that fulfill a third criterion (iii) - their precursor and/or mature form is found in the small RNA fraction [34]. The results are summarized in Figure S4.

**Figure 2:**
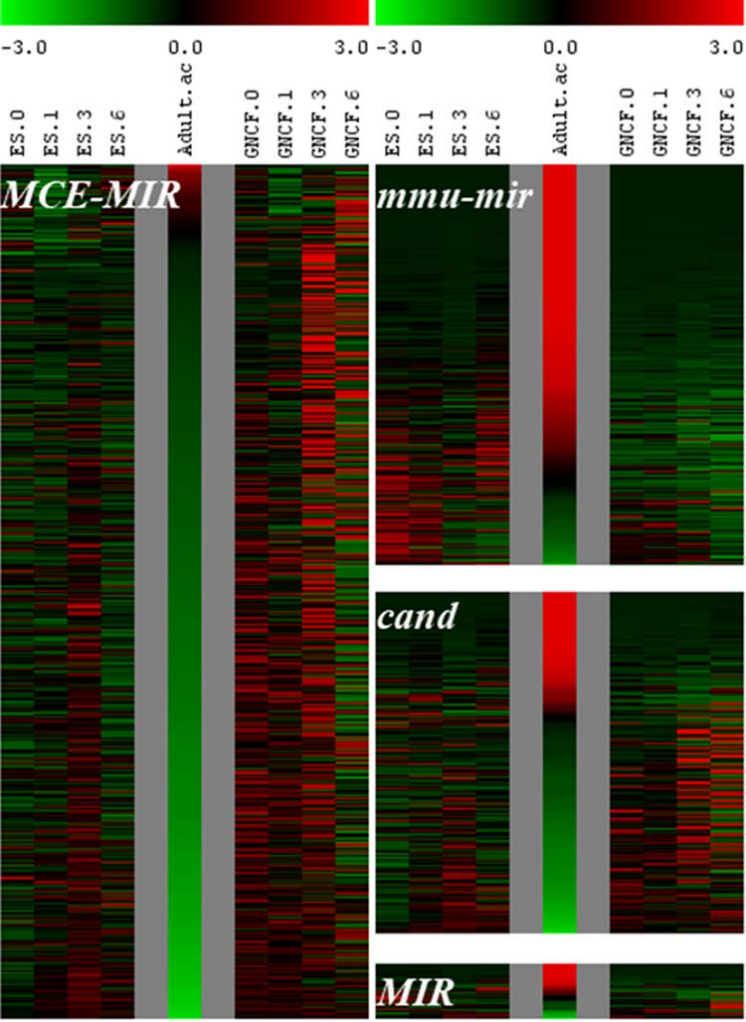
Comparison of expression trends of MCE-MIR, Cand and MIR predictions and mmu-miRs in ES cells, ES (*GCNF* ^−/−^) mutant and Adult Pool. The heat maps presented here summarize the expression trends of the four different classes of small RNA probes specific to mmu miRNAs in miRBase (mmu-mir) and miRNA predictions (Cand, MIR and MCE-MIR). Here we examine the expression trends of all of these small RNAs with respect to the trajectory of the ES cell during retinoic-acid (RA) induced differentiation up to day 6 using the fully differentiated adult panel as an end point in the differentiation program. Expression trends in the ES (*GCNF* ^−/−^) mutant during RA induction which is juxtaposed, clearly reveals the similarities and differences between. Particularly striking is the distinctly inverse patterns exhibited by MCE-MIRs as compared with mmu-miRs.

We found that 545 novel MCE-MIR candidates exhibit extraordinary enrichment in ES cells over adult tissue (Figure S5). The MCE-MIR expression patterns differed significantly from the majority of adult miRNAs (mmu-miRs) (Figure 2). The overlap between MCE-MIRs found in transcripts from adult stem cell and tissue progenitors and small RNAs expressed in ES cells, suggests that adult stem cells share common RNA signaling networks with ES cells. By contrast, the bulk of confirmed miRNAs from miRBase hybridize preferentially to the adult pool (P<10^−5^), as might be anticipated since they were cloned primarily from tissues represented in the adult pool. This outcome suggests that our ‘data-driven’ strategy may be particularly effective at the identification of miRNAs from cell types that are difficult to isolate in sufficient quantity for cloning small RNAs. Of the 545 novel MCE-MIR candidates a primary pool of 106 MCE-MIRs exhibited down-regulation in the ES (*Dicer* ^−/−^) mutant suggesting that they are putative novel miRNAs (Figure S6, Table S1). A second pool of 410 MCE-MIRs did not change in the ES (*Dicer* ^−/−^) mutant. It is possible that these MCE-MIRs are not processed at the stages we profiled them, or they are not miRNAs. Fifty confirmed mmu-miRs were also not down-regulated in the ES (*Dicer* ^−/−^) mutant, indicating that the requirement for down-regulation in Dicer knockouts may be too restrictive when we are looking at specific windows in developmental time in specific cell types. Thirty-four (34) MCE-MIRs and 9 mmu-miRs exhibited an increase in the ES (*Dicer* ^−/−^) mutant and may represent a novel class of small RNAs or miRNAs that are themselves repressed by other miRNAs.

### Three classes of miRNAs are revealed in pluripotent ES cells and during retinoic acid induced (RA-induced) differentiation

Within our expanded set of stem cell-related miRNA candidates, we were interested in identifying a minimal set of miRNAs that could underlie the distinctive properties of stem cells, including self-renewal, pluripotence and differentiation. We therefore used Mouse Array Version 2 (Figure S7A–7B) and containing the 545 MCE-MIRs found to be enriched in ES cells through, Cand and MIR predictions that yielded signal on Mouse Array Version 1 and mmu-miRs and mmu-miR* (S-mmu-mir) from miRBase. Using this second custom array we examined global miRNA expression profiles during RA-induced differentiation of wild-type (wt) and ES (G*CNF* ^−/−^) cells that fail to repress pluripotency genes *Oct4* and *Nanog* during differentiation. Undifferentiated ES cells (RA-D0), and ES cells on RA-D1, 3 and 6 following RA-induction were profiled with a miRNA microarray platform. The results are shown in Supplemental Tables S4A–S4C and Figure 2. MCE-MIRs identified from tissue progenitors clearly exhibit distinct and inverse behavior to adult mmu-miRs during RA-induced ES cell differentiation. The majority of MCE-MIRs exhibit high levels of expression in ES cells (see ES panel on left) in contrast to the majority of mmu-mirs which exhibit high levels of expression in adult tissue (see adult panel in the center). Furthermore, MCE-MIRs are characteristically induced on RA-D3, in contrast to the adult-enriched mmu-mirs, which show no such striking change on Day 3. Most importantly, in the differentiation-impaired ES (G*CNF* ^−/−^) mutant, MCE-MIR expression is significantly perturbed. Consistently high expression is evident together with failure to down-regulate during RA-induced. We also examined novel candidates from Cand and MIR prediction groups (see Figure 2 panels below, mmu-miR), which appear to contain a mixture of MCE-MIR-like and mmu-mir-like candidates.

**Figure 3:**
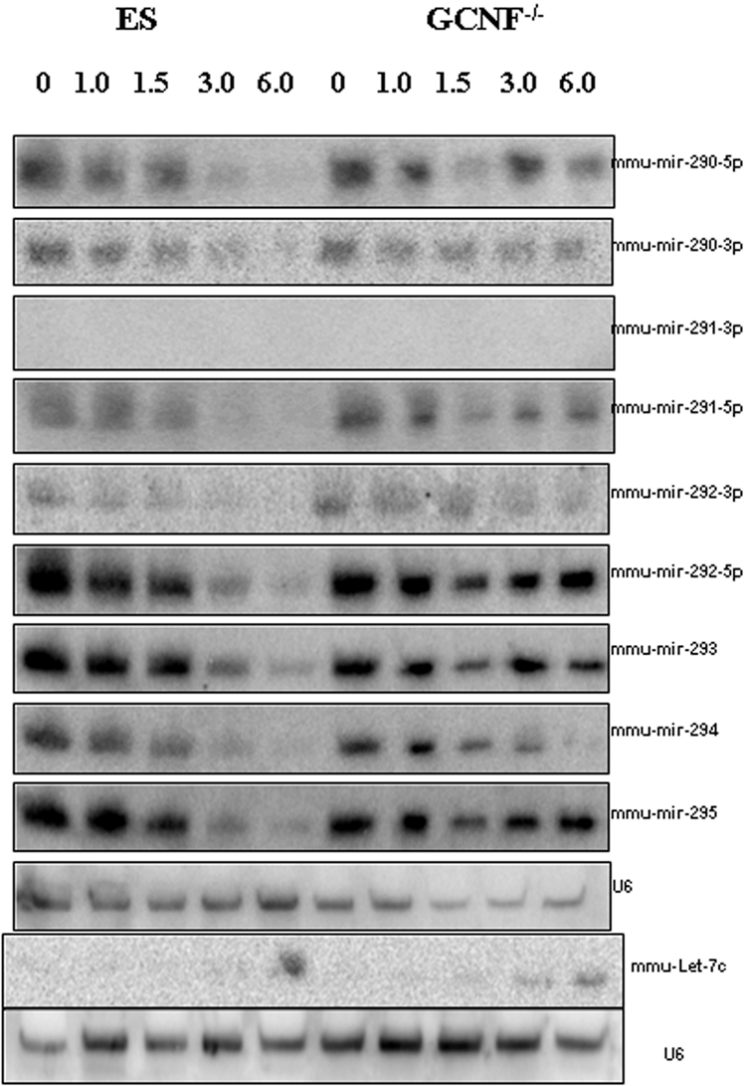
Northern Analyses of ES-specific mmu-miR 290–295 and adult-specific let-7c. The right panel shows the expression profile of the mmu-miR-290-295 cluster in ES cells during RA-induction. The left panel shows the expression profile of the mmu-miR-290-295 cluster in ES (*GCNF* ^−/−^) mutant during RA-induction. Here we see that the mmu-miR-290-295 cluster is enriched in ES cells and go down during RA-induced differentiation. This is by contrast, to let-7c which is not present in ES cells and goes up during RA-induction only in the ES (*GCNF* ^−/−^) mutant. The expression pattern of the mmu-miR-290-295 cluster is perturbed in the ES (*GCNF* ^−/−^) mutant and characterized by a failure to down-regulate in the mutant as compared to wild type ES.

**Figure 4:**
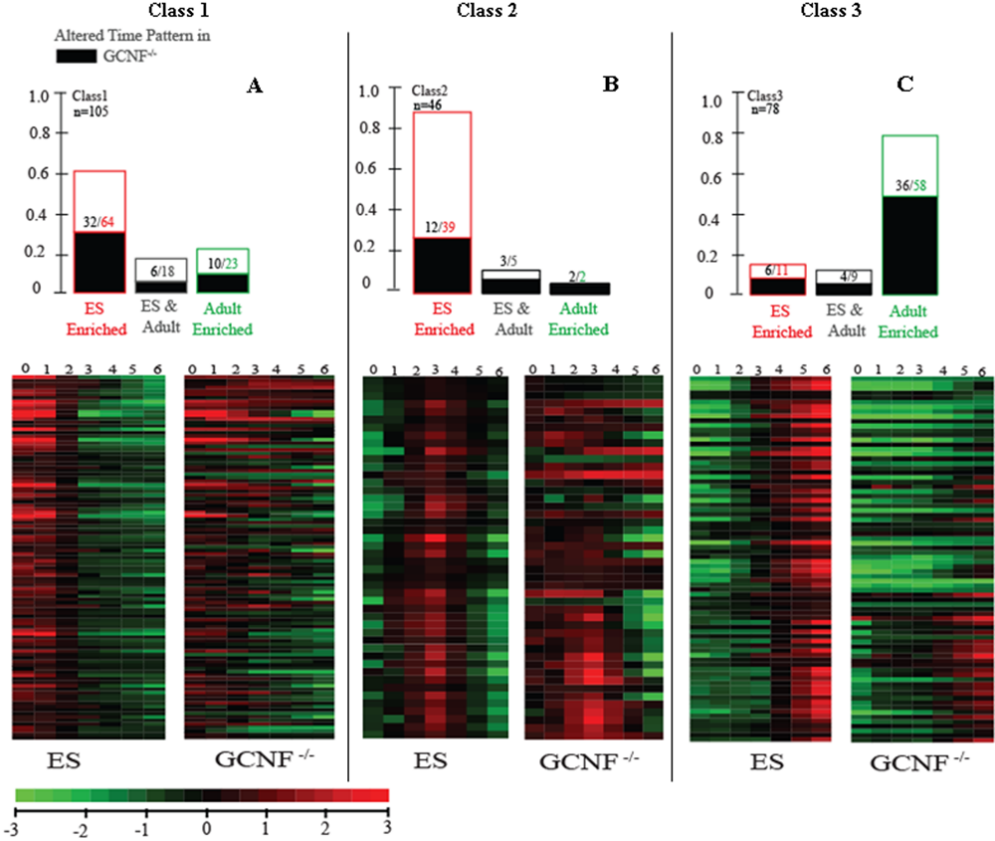
ES-GCNF retinoic acid induction time series. Dramatic time ordered patterns of differential expression are revealed by miRNA microarray data after retinoic-acid (RA) treatment. Three distinct classes were identified including Class 1 miRNAs, which, exhibit enrichment in ES (RA-D0) and down-regulation upon RA-induction (Panel A). Class 2 miRNAs that are transient in nature (Panel B) and Class 3 miRNAs that are low in ES (RA-D0) and up-regulated upon RA-induction (Panel C). In each bar plot we show the number of probes in each class, as well as the number whose time pattern is significantly disrupted in the ES (*GCNF* ^−/−^) mutant (colored black). Each row represents a distinct miRNA probe and each column represents a time point after RA treatment. Both the data for the ES cells and ES (*GCNF* ^−/−^) are shown, and the rows are normalized to their ES time-course mean. The actual measurements of miRNA levels were carried out on RA-Day 1, 3 and 6. The other days RA-Day 2, 4 and 5 have been interpolated as described in the Methods. The color legend for the heat map is shown below.

The mmu-mir-290-295 cluster and mmu-mir-302-cluster were the first sets of ES cell enriched miRNAs to be discovered [35]. Northern analysis of a subset including mmu-miR-290-295 is shown in Figure 3. This group of miRNAs is highly-expressed in ES cells and down-regulated during RA-induced differentiation – but their down-regulation fails in the ES (G*CNF* ^−/−^) mutant. In order to identify distinct classes of ES-related miRNAs that may be involved in pluripotence vs. differentiation, we carried out statistical analysis of the expression trends of these groups in the differentiation-impaired ES (G*CNF* ^−/−^) cells as compared to wild-type ES cells. RNA was isolated from ES and ES (G*CNF* ^−/−^) cells before (RA-D0) and after retinoic acid treatment on Day-1, 3 and 6. We linearly interpolated between the mean expression values at each of the 4 time points to obtain a 7-value time profile including Day-0, 1, 2, 3, 4, 5 and 6. As shown in Supplemental Table S5A and S5B, our analysis revealed three significant classes of temporal variation: **Class 1** (Figure 4A) representing miRNAs that are enriched in ES cells and down-regulated (105 miRNAs and candidates); **Class 2** (Figure 4B): Transiently induced miRNAs (46 novel miRNA candidates); and **Class 3** (Figure 4C): miRNAs that are absent or present only in low abundance in ES cells and up-regulated upon RA-induced differentiation (78 miRNAs and candidates). All miRNAs and candidates that exhibited minimal changes in expression during the RA treatment were classified by default in **Class 4** (881 miRNAs and candidates). The majority of **Class 1** (61%) and **Class 2** (85%) miRNAs and candidates were enriched in undifferentiated ES cells (RA-D0) over the Adult Pool. Conversely, the majority of **Class 3** miRNAs and candidates (74%) were enriched in the Adult Pool over undifferentiated ES cells (RA-D0).

The third significant finding is revealed by the juxtaposition of the expression patterns of ES and ES (G*CNF* ^−/−^) mutant cells. Dramatic perturbation is evident in the expression trends of members in all three classes in the ES (G*CNF* ^−/−^) mutant. To more precisely identify and characterize the ES (G*CNF* ^−/−^) responsive subclasses within these groups, we performed an analysis (F-test of interaction effects) that evaluates the effect of genotype on the pattern of temporal expression. We found that the expression patterns of 45% (48/105) of **Class 1**, 37% (17/46) of **Class 2**, and 59% (46/78) of **Class 3** miRNAs were significantly altered in the ES (G*CNF* ^−/−^) cells. These data are summarized in Tables S5A and S5B and Figure 4. At the peak of *GCNF* expression on RA-D3, *Oct4* and *Nanog* are repressed, leading to the down-regulation of self-renewal regulators, differentiation inhibitors and Class 1 miRNAs. Class 2 miRNAs shown in Fig. 4B are exclusively represented by novel miRNA candidates derived from our search for MCEs. Their transient induction during RA-D1-3 parallels *GCNF*, so that they are likely to function in the transition of pluripotent ES cells to RA-induced differentiation. This finding highlights the need for exhaustive searches for miRNAs that are transiently expressed in limited numbers of cells or during narrow windows of developmental time.

### Key miRNA-mRNA pairs correlating with ES self-renewal, pluripotence and differentiation

**Figure 5:**
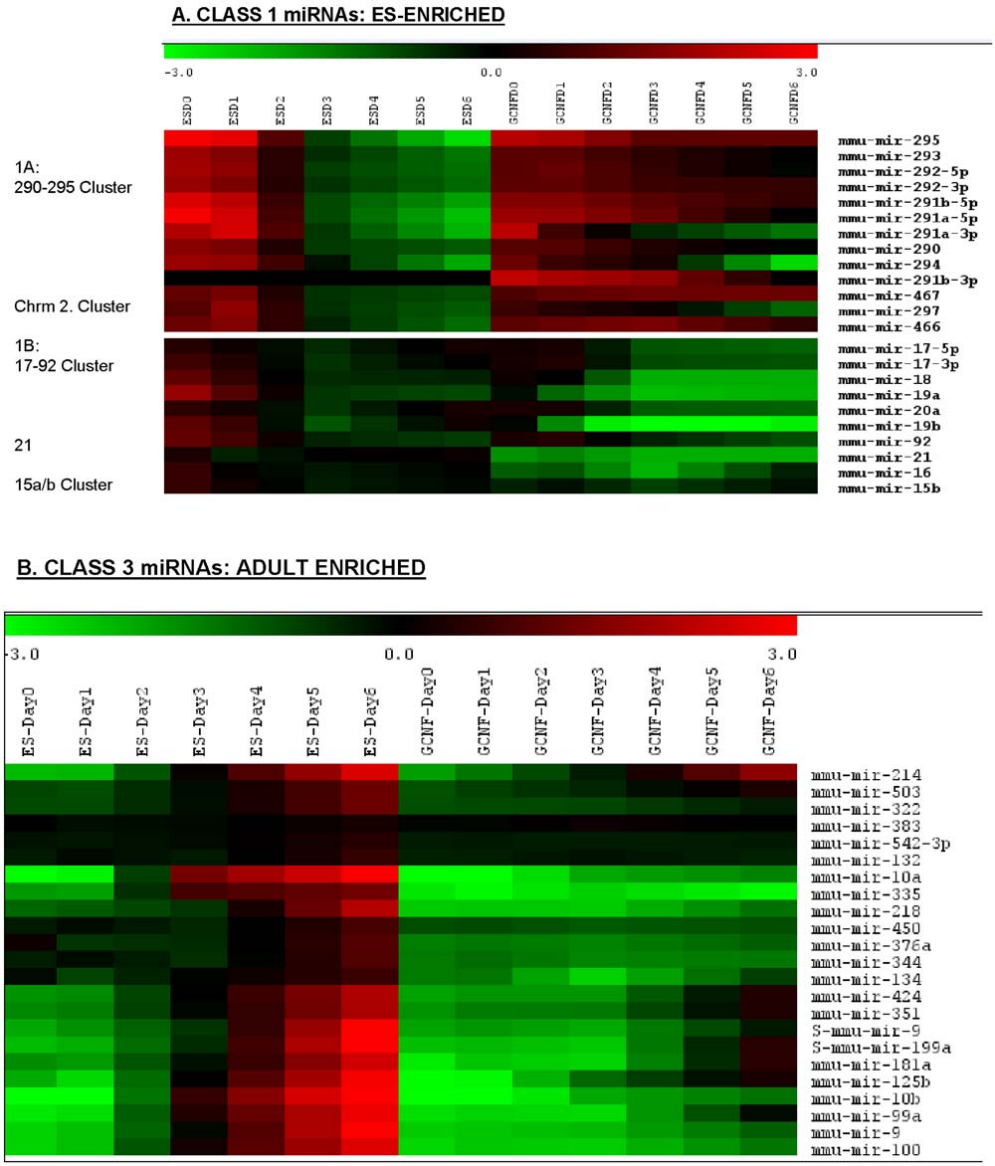
Class 1A, 1B and 3 miRNA expression profiles. This figure shows the expression profiles of key miRNA clusters found in ES cells [26] determined both in ES and ES (*GCNF* ^−/−^) mutant. ES (Day0) corresponds to miRNA profiles in pluripotent ES cells during self-renewal. GCNF (Day0) corresponds to miRNA profiles in ES (*GCNF* ^−/−^) mutant. Day 1–6 correspond to ES or GCNF following retinoic acid (RA) treatment. Experimental data was obtained for Day0, 1, 3 and 6. All other days have been linearly interpolated based on experimental values. Expression profile of miRNAs belonging to Class 1A and 1B are shown in Panel A. Class 3 miRNAs are shown in Panel B.

Recently, more than 75% of miRNAs identified in ES cells by pyrosequencing were found to be transcribed from six genomic locations. These include the 290–295 cluster (29%), Chr. 2 cluster (27%), 17–92 cluster (11%), Chr. 12 cluster (4%), mmu-mir-21 (2%) and 15a/b cluster (3%) [26]. The miR-290-295 cluster, Chr. 2 cluster, 17–92 cluster, 15a/b and 21 clusters all have properties consistent with miRNAs that support self-renewal and pluripotence. They are elevated in ES cells, go down during RA-induced differentiation and are perturbed in the ES (G*CNF* ^−/−^) mutant. The ES (G*CNF* ^−/−^) mutant allows us to further stratify these Class 1 miRNAs based on the nature of perturbation during RA-induction. The miR-290-295 cluster and Chr. 2 clusters remain elevated and fail to go down during RA-induction in the ES (G*CNF* ^−/−^) mutant. In contrast, 17–92 cluster, 15a/b and 21 clusters fail to induce at all in the ES (G*CNF* ^−/−^) mutant. We therefore categorize them into two subclasses Class 1A and 1B respectively. The expression profiles of these clusters are shown in Figure 5A. Class 3 miRNAs that are induced during differentiation and exhibit opposite expression characteristics to Class 1 are shown in Figure 5B.

**Figure 6:**
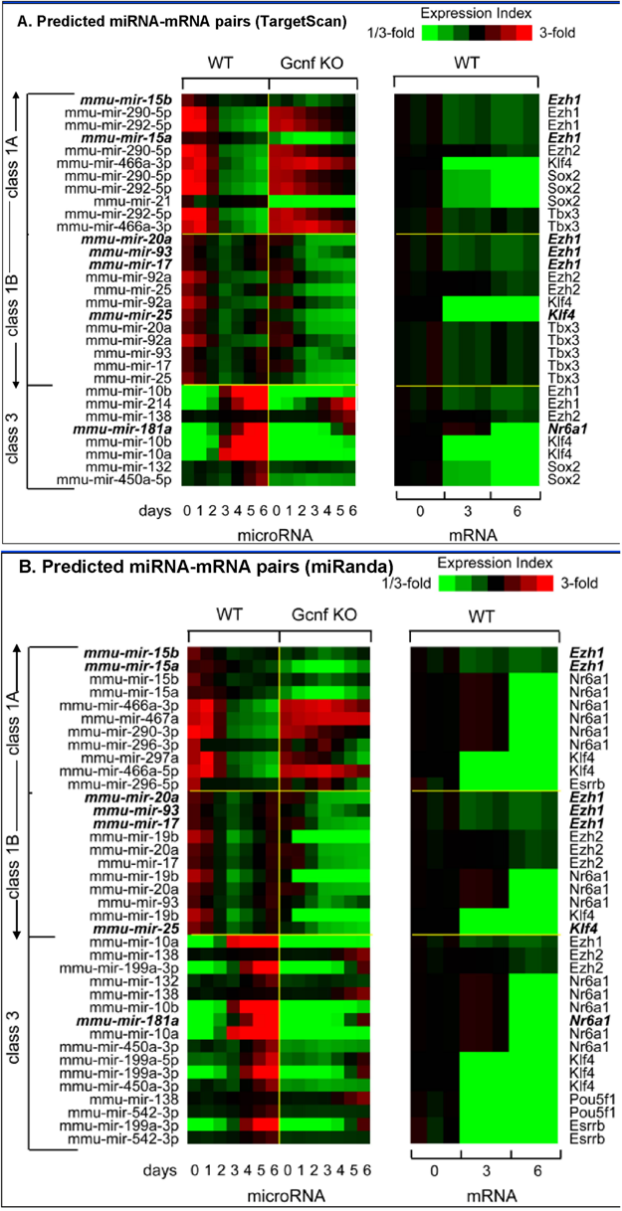
Relative expression profiles of predicted miRNA-mRNA pairs. This figure shows results from microRNA microarray combined with mRNA data obtained through Affymetrix expression array profiling. Panel A shows miRNA-mRNA relationships based on TargetScan and Panel B shows miRNA-mRNA relationships based on miRanda. Here we see that in general Class 1 miRNAs are positively correlated in their expression trends as compared with their predicted targets. By contrast, Class 3 miRNAs are negatively correlated. Significant miRNA-mRNA relationships are determined through an enrichment analysis, which, is described in the methods section and results are shown in Table 1.

Figure 6A shows the relative expression profile of miRNA and target mRNAs relating to key genes involved in ES cell self-renewal and pluripotence as predicted by TargetScan [36]. Figure 6B shows the relative expression profile of miRNA and target mRNAs relating to key genes involved in ES cell self-renewal and pluripotence as predicted by miRanda [37]. Table 1 shows the enrichment analysis on miRNA-mRNA pairs obtained from TargetScan predictions. In this analysis, for each of the key genes involved in regulating ES cell self-renewal and pluripotence, we compute a specificity score for each miRNA class. The specificity score allows us to determine the relative enrichment of a specific miRNA-mRNA targeting event in relation to all of the other miRNAs that target that gene that was also expressed in these cells. Class 1A miRNAs miR-290, miR-292-5p, miR-467 and Class 1B miR-21 target *Sox2* with a significant enrichment score of 4.3. Class 1B miRNAs miR-17-92 cluster targets *Tbx3* and *Ezh2* with significant enrichment scores of >2. In both cases the miRNA-mRNA pairs are positively correlated in their expression as shown in Figure 6A–B. In contrast, Class 3 miRNAs miR-138, miR-542-3p target *Oct4* with enrichment scores of 3.9. Class 3 miRNAs miR-214, miR-199, miR-542-3p and miR-10 all target *Ezh1* with an enrichment score of >2. In both cases the miRNA-mRNA pairs are negatively correlated in their expression as shown in Fig. 6A–B.

**Table 1:** Specificity of predicted miRNA-mRNA targeting associations.

Genes	Genes in Tscan	Targeting Events/class	Targeting Events/all miRs expressed	miRs/class	Enrichment Score
**Class1A**					
**Class 1B**					
Sox2	Sox2	4	19	mmu-miR-467,mmu-miR-290,mmu-miR-292-5p	4.33
Tbx3	Tbx3	2	34	mmu-miR-17-5p/20/93.mr/106/519.d,miR-25/32/92/363/367	2.33
Ezh2	Ezh2	1	11	mmu-miR-25/32/92/363/367	2.26
Ezh2	Ezh2	1	11	mmu-miR-290	1.08
Tbx3	Tbx3	2	34	mmu-miR-292-5p,mmu-miR-466	0.84
Klf4	Klf4	1	25	mmu-miR-466	0.2
Oct4	Pou5f1	0	7		−0.49
Gcnf	Nr6a1	0	13		−0.67
Ezh1	Ezh1	2	48	mmu-miR-19,miR-17-5p/20/93.mr/106/519.d	1.73
Klf4	Klf4	1	25	miR-25/32/92/363/367	1.17
Oct4	Pou5f1	0	7		−0.31
Nanog	LOC100038891	0	10		−0.37
Gcnf	Nr6a1	0	13		−0.42
Sox2	Sox2	0	19		−0.5
**Class3**					
Oct4	Pou5f1	2	7	mmu-miR-138,mmu-miR-542-3p	3.99
Ezh1	Ezh1	4	48	mmu-miR-214,mmu-miR-199,mmu-miR-542-3p,mmu-miR-10	2.19
Sox2	Sox2	2	19	mmu-miR-450,mmu-miR-132/212	1.94
Ezh2	Ezh2	1	11	mmu-miR-138	1.2
Gcnf	Nr6a1	1	13	mmu-miR-181	1
Klf4	Klf4	1	25	mmu-miR-10	0.3
Nanog	LOC100038891	0	10		−0.55
Tbx3	Tbx3	0	34		−1.02

For the set of mRNAs differentially expressed miRNAs in ES cells that satisfied the criteria of Class 1A, 1B and 3 we asked the question, is there a non-random number of predicted targets? All conserved and non-conserved target sites for each of the genes indicated in the table were downloaded from TargetScan. Scaling by the corresponding variance estimate, we derived Z-scores for each gene for targeting enrichment. Enrichment scores of >2 are significant. Of the large number of predicted interaction between miRNAs in Class 1A, 1B and 3 we find that only a fraction exhibits enrichment scores of >2 and therefore are highly specific. We conclude that these miRNA-mRNA pairs are the most important in the regulation of ES cell self-renewal and pluripotence.

**Figure 7:**
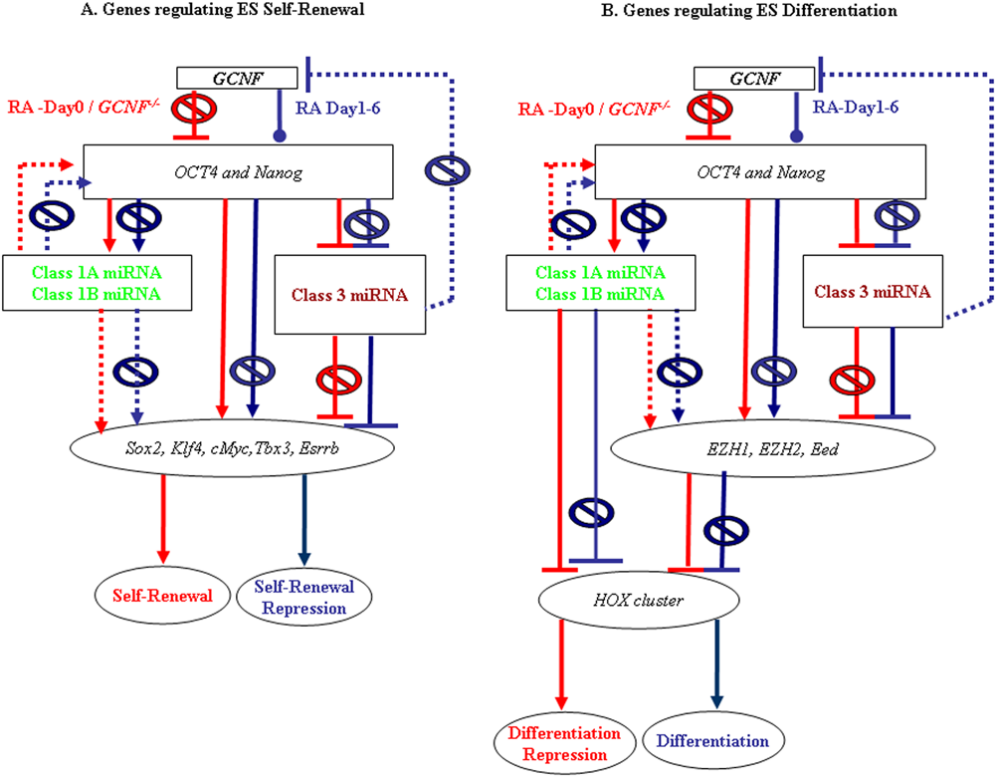
Predicted miRNA-mRNA regulatory loops underlying ES self-renewal maintenance and differentiation repression. Here we compared the expression of key miRNAs (mmu-miRs) with target genes that have been established to play an important role in ES cell self-renewal maintenance, differentiation inhibition and differentiation potentiation. Class 1 miRNAs are shown in green and Class 3 miRNAs are shown in purple. Arrows represent relationships that reflect activation. Bars with a hash at end represent relationships that reflect inhibition. The dotted lines represent miRNAs-target relationships that exhibit co-expression and positive correlation. Solid lines represent miRNAs-mRNA relationships that exhibit anti-correlation. Red represents events that occur during self-renewal. Blue represents events following RA-induction. Panel A represents the predicted regulatory miRNA-mRNA relationships that may be in operation during ES cell self-renewal. Panel B represents the predicted regulatory miRNA-mRNA relationships that may be in operation during ES cell differentiation inhibition or potentiation.

We expect miRNAs that are involved in the maintenance of the differentiated state to be enriched in adult tissue and rapidly induced in a GCNF-dependent late response to RA treatment. These miRNAs may support differentiation by stably repressing self-renewal regulators and/or differentiation inhibitors. MiRNAs in this group are rapidly up-regulated following RA-D3 and are expressed at very low levels in self-renewing ES cells on RA-D0. Class 3 miRNAs that are induced following the peak of *GCNF* expression are mostly negatively correlated in their expression with self-renewal regulators, PcG repressors and *GCNF* itself (Fig. 6A–B). Especially striking is a potential feed-back loop in which mmu-miR-181a, expressed from the opposite strand of an intron of *GCNF*, also has target sites in the 3′-UTR of *GCNF*. Since one of the primary functions of *GCNF* is to repress *Oct4/Nanog*, which is essential to maintenance of pluripotence, it is possible that miR-181a is repressed by *Oct4/Nanog* in ES cells. *GCNF*-mediated repression of *Oct4/Nanog* during ES (RA-D1-3) may mitigate this repression and explain the up-regulation of mmu-miR-181a on ES (RA-D2-6). Mmu-miR-181a mediated repression of *GCNF* may be in part responsible for the transient nature of *GCNF* expression of ES (RA-D1-3). Based on these correlations we have formulated potential regulatory loops that may be fundamental to the inhibition of differentiation in ES cells. Figure 7 shows the predicted behavior and relationships of the miRNA-mRNA pairs that may be fundamental to the facilitation of differentiation through the repression of pluripotence and self-renewal of ES cells.

## Discussion

Recently, we have witnessed a rapid expansion in the numbers of potential and confirmed miRNAs in mammalian genomes. Many of these novel miRNAs and candidates have been inferred by comparative genomics, [31]–[34] and others by high-throughput sequencing [26], [38]. Although they overlap with one another, with previously cloned miRNAs, and with other sets of predictions, what is most striking is that these varied approaches to miRNA discovery have also yielded sharply distinct sets of new miRNAs. In this paper, our search for novel miRNAs is based upon the perfect conservation of sequence among two sets each comprised of three species in relation to rare tissue progenitor sequences. Many confirmed miRNAs are not strongly conserved among our choice of organisms; indeed, fewer than 40% of the miRNAs that had already been confirmed when this project was begun actually satisfy this condition. While it has been remarked that most miRNAs recently discovered by massively parallel sequencing are by conventional measures not well-conserved [26], this observation is by itself no more meaningful than the observation that most protein coding sequences are far less conserved than, for example, actin- or homeo- coding domains [28].

Sequence conservation, on the other hand, is generally believed when properly interpreted to reflect selection for function and in this sense can be highly specific. Strongly-conserved elements in the genome have been argued to constitute cores of conserved networks. In summary, conservation-based gene discovery permits high specificity for function, but often at a substantial cost in sensitivity. It can enable discovery of genomic sequences that play pivotal roles in the cell but are never transcribed or are expressed at such small copy number and/or so intermittently that they elude the resolution of current sequencing technology. That is, massively-parallel sequencing complements comparative genomics rather than replacing it. The relative sensitivity and specificity for miRNA of comparative genomics and massively-parallel sequencing technologies will eventually be determined by empirical studies such as this one. For the purposes of the work described in this paper, our primary goal is to identify the central contributors to regulating the essential and ancient networks involved in stem-cell differentiation, and strong conservation, as realized in MCEs, represents a uniquely suitable means toward this end.

The discovery of miRNA candidates that are common to embryonic and adult stem cells first reported here is suggestive of common RNA signaling networks underlying multi-potent and self-renewal properties unique to these two cell types. Our work sets the stage for studying the role of miRNAs in the key developmental transition from pluripotence to differentiation. The phenotype of ES (*Dicer^−/−^*) is characterized by failure to down-regulate master self-renewal regulators *Oct4* and *Nanog*, failure to differentiate, and lethality at embryonic day E7.5 [21], suggesting an important role for miRNAs early in stem cell development. On the other hand, knockdown and knockout of individual miRNAs generally yields less drastic phenotypes [39]–[44].

Recently, computational analysis of gene expression data revealed two types of recurrent circuit motifs [45]. Type I circuits in which miRNAs and their target mRNAs are positively correlated in a relationship are consistent with miRNAs forming a threshold which must be exceeded in order for the target mRNAs to be translated [46]–[47]. In contrast, Type II circuits in which miRNA-mRNA pairs are oppositely correlated (miRNA-up/mRNA-down or miRNA-down/mRNA-up) are more consistent with a straightforward silencing function for miRNAs. Class 1A and 1B miRNAs exhibit two distinct patterns of expression in relation to their predicted targets. Class 1A/1B miRNAs form two key Type I circuits in which they are positively correlated and co-expressed with self-renewal regulators *Sox2* and *Tbx3* and the differentiation inhibitor *Ezh2*. Class 3 miRNAs on the other hand form Type II circuits in which they are oppositely correlated with the master regulator of self-renewal and pluripotence *Oct4* and the differentiation inhibitor *Ezh1*.

From these data, we infer that the Class 1A/1B miRNAs, which account for over 50% of the miRNAs in ES cells, have dual functions. One is to establish thresholds for gene networks regulating the maintenance of pluripotent self-renewal state through translational inhibition. In this mode they cooperate with transcription factors and act to ensure translation exclusively of the target genes that are transcribed above the threshold set by Class 1A/1B miRNAs. The other function is to co-operate with PcG repressors to inhibit differentiation by post-transcriptionally silencing *Hox* targets.

Class 3 miRNAs appear to be mainly driving Type II circuits, in which they cooperate with GCNF-mediated repression of self-renewal regulators and differentiation inhibitors. The Class 3 miR-181a is especially interesting since it is expressed from an intron of *GCNF* - although from the non-coding strand - and also is predicted to target *GCNF* through 3′-UTR binding sites. Loss of miR-181a expression in the ES (*GCNF* ^−/−^) mutant and the up-regulation of the mutant *GCNF* transcript (only the DNA-binding domain of *GCNF* was disrupted in ES (*GCNF* ^−/−^) suggests that mmu-miR-181a may be involved in a feed-back loop that ensures the transient expression of *GCNF* (RA-D1-3) required for the transition from pluripotence to differentiation. From this work we propose that Class I miRNAs, are critical for regulating precision control and robustness stem cell gene networks. We also speculate that some of the phenotypes of the ES (*Dicer^−/−^*) may be consequences of loss of stability of gene networks regulating pluripotence, self-renewal and differentiation in ES cells.

## Materials and Methods

### Discovery Strategy

Our discovery strategy was motivated by the need to apply pure conservation-based discovery methods of [27], [28] to the purpose of finding novel miRNAs in stem cells. The fundamental observation of [27], [28] was that longer stretches of conserved sequence are no more indicative of evolutionary constraint than are short sequences. As a consequence, MCEs were dismissed by computational biologists as “poorly-conserved” because they are so short; however, in reality they are strongly-enriched for functional elements, and in particular for miRNAs.

On the other hand, among evolutionarily-constrained sequences in the mammalian genome we are aware of no demonstrated enrichment for stem-cell roles. For our study, enrichment was obtained by a “data-based strategy” of searching for conserved sequence only within a subset of genomic sequence that had already been collated in stem-cell transcript libraries [SCGAP]. This transcribed sequence amounts only to a tiny fraction of the genome (∼1%), eliminating the overwhelming majority of strongly-conserved sequence genome-wide and cutting the characteristic lengths of these sequences to scales below those most strongly enriched in mammals for confirmed miRNAs [27], [28].

The prediction of [27], [28] is that those sequences falling on the scaling curve from genomic alignments are under strong evolutionary constraint; however, their functions – if not miRNAs - are most likely novel, presenting an intangible experimental challenge. We therefore selected only a subset of the MCEs that satisfied certain characteristics of previously confirmed miRNAs. Although we anticipated that as more miRNAs were experimentally confirmed these conditions would turn out to be overly restrictive, our judgment was that the potential costs of validating potential miRNAs that violated the conventional wisdom were too great, and we therefore adopted the practice – standard in bioinformatics – of “over-fitting” to the set of confirmed miRNAs and sacrificing our ability to generalize. These characteristics included criteria on sequence complexity, on the number of occurrences of the sequence in the genome, and on secondary structure. For example, mammalian miRNAs confirmed at the time rarely occurred more than a few times within each genome, and we eliminated any sequence that appeared more than three times within in any genome contributing to the intersection. These limitations, which render a truly realistic assessment of the rate of false negatives impractical and apply quite apart from considerations of experimental validation, suggest that there are many additional miRNAs within the set of SCGAP transcripts that remain to be validated.

### *k*-mer based MiRNA prediction algorithm

#### False Positives

Following the work of Lai et al. [48] and Lim et al. [49], we estimate our false positive rate from the experimental tests carried out on the predicted sequences. For the purpose of this calculation, we consider all sequences that yielded positive signals on the array to be microRNAs.

#### False Negatives

We have taken two distinct approaches to calculating false negatives.

First, we computed the fraction of confirmed miRNAs that satisfy our folding criteria. We obtained flanking sequence for all human mature miRNA sequences from miRbase and applied our folding filter. Approximately 7% of the known human miRNA precursors failed on both strands; 13% on one strand; and 80% passed the folding filter on both strands. It is worth noting that our miRNA filter depends on the folding of MCE cognates from multiple genomes and; therefore, is more stringent than the direct folding filter that we applied to confirmed miRNA, which relies on flanking sequence from one organism only.

Second, we identified all MCEs in the intersection of human-mouse-rat whole genomes that share a sequence of length k≥20 with one of the 321 human miRNA precursor predictions in miRBase. There are 269 such MCEs with length in the 20–50 nucleotide range, representing 183 distinct hairpins. We found that 200 of the 269 MCEs (74%) satisfied our miRNA structure filter, accounting for 149 out of the 183 unique hairpins (81%). Finally, we took the intersection of all 321 human miRNA precursors in the miRBase with the mouse and rat genomes, and the dog and cow genomes, yielding the outcomes summarized below.

a. miRNA-mouse-rat: There are 92 miRNA precursors (28%) containing a sequence with k≥20 that is conserved among the two genomes and the human miRNAs. There are 280 MCEs in this intersection with 50≥k≥20 and 222 of them pass the folding filter (79%). Of the 86 MCEs in the length 22–29 range, 76 pass the folding filter (88%).

b. miRNA-dog-cow: There are 127 miRNA precursors (39%) containing a sequence with k≥20 that is conserved among the two genomes and the human miRNAs. Of the 285 MCEs in this intersection with 50≥k≥20, 212 of them pass the folding filter (74%). Of the 84 MCEs in the length 22–29 range, 75 of them pass the folding filter (89%).

Our folding filter is novel in that it takes advantage of our discovery [50] that requiring both strands of the genomic sequence of a putative miRNA precursor to satisfy Ambros-like secondary structure criteria yields higher specificity for confirmed miRNAs than does the customary requirement on only one strand. The cost in sensitivity for confirmed miRNAs is relatively small. We stress that these are empirical observations on the set of confirmed miRNAs in insects and vertebrates that we apply simply because they are effective, without speculating on their origin.

Using this procedure, approximately 2.5 million SCGAP-k-mers were compared to each of the respective of k-mer sets from repeat-masked human, mouse, rat, cow and dog genomes. Through our exhaustive transcriptome-wide search we identified all distinct k-mers (sequences of length k bases, with 50≥k≥20 nt) perfectly conserved between SCGAP-mouse transcripts and two different pairs of whole genomes: (i) SCGAP-mouse-human-rat genomes ([SCGAP-m]-h-r); and (ii) SCGAP-mouse-dog-cow genomes ([SCGAP-m]-d-c). from each of the [SCGAP-mouse]-human-rat ([SCGAP-m]-h-r) and [SCGAP-mouse]-dog-cow ([SCGAP-m]-d-c) ternary intersections. Only ∼20,000 MCEs were shared between the two intersections.

We reject “uncharacteristically simple” sequences to avoid polluting the MCE population with simple repeats or other sequences which are likely strongly conserved for reasons other than being functionally important short RNAs. *We define an “uncharacteristically simple sequence” as any sequence which is within 4 mismatches of a 1-,2-,3-, or 4-mer repeat, or any sequences which have significantly uneven base distribution*. We use the entropy as our base distribution measure, defined as the sum over bases of fi*ln[fi] where fi is the fraction of the MCE which consists of the ith base. MCEs with entropy greater than 1.275 were rejected.

We selected MCEs that (i) formed a sufficiently low-energy stem-loop secondary structure; and (ii) spanned just one strand of the stem [29]. Eliminating MCEs whose sequences were uncharacteristically “simple” for microRNAs previously confirmed by that time, and applying our folding criteria, we identified. Approximately, 3000 distinct microRNA candidates (MCE-MIR) from [SCGAP-m]-h-r (∼75 kbps) and ∼1600 distinct MCE-MIRs from [SCGAP-m]-d-c (∼40 kbps) were uncovered.

### SCGAP sequences

The largest fraction of MCE-MIR predictions (44%) were derived from SCDb, which contains sequences from cDNA subtraction between extensively purified “Sca+” (stem cell-enriched) and “AA4-” (stem cell-depleted) populations from fresh day-14 mouse fetal liver (http://stemcell.princeton.edu/). Thirteen percent (13%) were derived from the stem cell supportive cell line AFT024, derived from the murine fetal liver microenvironment (http://stromalcell.princeton.edu). MCEs from murine Hematopoietic Stem Cell (HSC) side population (SP) cells in the quiescent and 5′-fluorouracil-activated states, as well as whole bone marrow (representing the differentiated state), were also included on the custom array (http://condor.bcm.tmc.edu/scgap/hscseq.htm). The SiEP and GEP sequences were derived through laser capture microdissected (LCM) epithelial progenitors from the small intestine and gastric epithelium, respectively (http://genome.wustl.edu/GSCGAP). MCEs identified from bipotential mouse embryonic liver (BMEL) cells were cultured in the stem cell-like state and normalized to enrich for rare transcripts and subtracted against BMEL cells (http://condor.bcm.tmc.edu/scgap/liverseq.html) cultured in a hepatocyte-like state to enrich for stem cell specific transcripts, these were used as representatives of the liver stem cell.

### µParaflo™ MicroRNA microarray Assay

Microarray assay was performed on a custom array by a service provider (LC Sciences). A 5 µg total-RNA sample was size-fractionated with a mirVana Isolation kit (Ambion); the small RNAs (<200 nt) isolated were 3′-extended with a poly-A tail by poly-A polymerase. An oligonucleotide tag was ligated to the poly-A tail for subsequent fluorescent dye staining; two different tags were used for the two RNA samples in dual-sample experiments. Hybridization was performed overnight on a µParaFlo microfluidic chip using a micro-circulation pump (Atactic Technologies) [51]. On the microfluidic chip, each detection probe consisted of a chemically modified nucleotide “coding” segment complementary to target microRNA (from miRBase, http://microrna.sanger.ac.uk/sequences/) or other RNA (control or customer defined sequences) and a spacer segment of polyethylene glycol to extend the “coding” segment away from the substrate. The detection probes were synthesized *in situ* with PGR (photogenerated reagent) chemistry. The hybridization melting temperatures were balanced by chemical modifications of the detection probes. Hybridization was carried out in 100 µL 6xSSPE buffer (0.90 M NaCl, 60 mM Na_2_HPO_4_, 6 mM EDTA, pH 6.8) containing 25% formamide at 34°C. After hybridization, detection was performed by fluorescence labeling with tag-specific Cy3 and Cy5 dyes (Invitrogen). Hybridization images were collected with a laser scanner (GenePix 4000B, Molecular Device) and quantified. Data were analyzed by first subtracting the background and then normalizing with a cyclic LOWESS filter (Locally-weighted Regression) [51]. For two color experiments, the ratio of the two sets of detected signals (log2 transformed, balanced) and p-values of the t-test were calculated; a p-value of less than 0.01 was our criterion for a differentially-detected signal. Data classification was accomplished by hierarchical clustering based on average linkage and Euclidean distance metric, and visualized with TIGR's MeV (Multiple Experimental Viewer) (the Institute for Genomic Research).

All microarray data are based on six probe replicates for each miRNA prediction (MCE-MIR, Cand and MIR) and eight probe replicates for mmu-mirs. ES and GCNF samples represent RNA isolated from pooled material from two independent 10 cm dishes with ∼75 million cells each from each time point of the ES and GCNF−/− time series experiment. For each time point, two dishes were pooled to harvest 0.5–1.0 mg of RNA for the arrays. Each time point is represented by duplicate arrays with dye swap of the label.

RNA was isolated from ES and ES (GCNF−/−) as well as on Day 1, 3 and 6 following retinoic acid (RA) treatment. Two color data were normalized by performing quantile normalization on the channel values [52] within each hybridization. A dendrogram was constructed on the single channel values both before and after normalization to examine the effect of normalization on the treatment differences. Although treatment information was not utilized in the normalization, we observed that the effect of the quantile normalization was to make the single channel values within treatment groups more similar and to enhance the distinction between different treatments. We infer that the effect of quantile normalization is to make single channel values within individual arrays and between arrays more comparable and to improve the multi-array data analysis. The data was then extracted and the single channel normalized values were used in subsequent data analysis.

### Maintenance and RA-differentiation of ES cells and Sample Preparation

ES cells were maintained in ES cell media [DMEM medium supplemented with 15% CFS tested for ES cell culture, 100 mM non essential amino acid, 2 mM glutamine, 100 U of penicillin-streptomycin/ml (Invitrogen) and 0.55 µM β-mercaptoethanol (Sigma)] supplemented with 1000 U/ml of LIF (Chemicon, Temecula, PA). The media was changed daily. For differentiation, ES cells were cultured in ES media without LIF but containing 10^−6^ M all-trans-RA (Sigma) and harvested at different time points. For RA-D0 differentiation, cells were cultured in ES cell media with LIF instead of RA, and media was changed daily for 2 days.

RNA isolated at each time point of the ES and GCNF−/− time series experiment from material pooled from two independent 10 cm dishes each with ∼75 million cells were used to probe Mouse Array Version 2. For each time point, two dishes were pooled to harvest 0.5–1.0 mg of RNA for the arrays. Each time point is represented by duplicate arrays with dye swap of the label.

### Pattern Analysis and Statistical Modeling

Our experiment involved 9 treatments: (1) Adult tissue pool (Adult Pool); time ordered samples taken at (2) RA-D0, (3) D1, (4) D3 and (5) D6; subsequent to application of Retinoic Acid (RA) from (7) cultured ES cells; and (8) from cultured GCNF^−/−^ cells. We combined the within-array replicates on individual arrays and searched for patterns. To perform pattern analysis, we first obtained mean values by averaging across the replicate arrays within each treatment. For the ES and GCNF^−/−^ time course samples, we linearly interpolated between the mean expression values at each of the 4 time points, obtaining a 7-value time profile for each oligonucleotide probe. We then centered the values about their mean, and performed principal components analysis and k-means clustering. This analysis suggested that three simple patterns dominate the time-course data. To make this assessment more precise and to incorporate variance information available from the sample replicates, we computed an ANOVA model and performed statistical testing. We fit a linear model treating time as a factor variable and including interaction effects for the GCNF^−/−^ treatment. This model yields an estimate of the within-treatment error variance for each probe. We then use this error variance together with the mean values at each treatment to construct linear contrasts for pattern analysis. Our first comparison of the variance information was between the RNA from a pool of differentiated adult tissue (Adult Pool) and the ES cells prior to treatment with RA (RA-D0). We computed a linear contrast score for each probe and used the *limma* (2004) R package [53] to derive an empirical Bayes adjustment to our single-probe variance estimates and the Benjamini-Hochberg linear step-up procedure to hold the False Discovery Rate (1995) [54] at 0.05. We used the PCA analysis to define two mean-0 linear contrasts according to the first two PCA modes; these two modes account for more than 95% of the variance in the time course data. As in the analysis of the Adult Pool vs. ES RA-D0, we used the *limma* package to improve our single-probe variance estimates and the linear step-up FDR correction [53] account for multiple comparisons.

The time-course pattern analysis yields three distinct classes of genes: Go-Up, Up-Middle, and Go-Down: Class 1, exhibiting a downward trend; Class 2, transient in nature; and Class 3, exhibiting an upward trend. To evaluate the effect of GCNF ^−/−^ on these time patterns, we examined the F-statistic for the interaction of GCNF^−/−^ on the time effects. We identified 200 probes where the GCNF treatment had a significant effect on the time pattern of expression.

### RNA extraction and Northern Blot

Total RNA was extracted with Trizol reagents (Invitrogen) and precipitated with 5 volumes of ethanol. 30 µg of total RNA was resolved in 15% of denaturing polyacrylamide gel containing 7 M urea in 0.5XTBE buffer system and transferred onto Zeta-Probe membrane (BioRad) in 0.5XTBE. DNA oligos were radioactively labelled with [γ-^32^P] ATP (MP Biomedialcs) and T4 kinase (Invitrogen). UV-cross linked membrane was hybridized with radioactively-labelled DNA oligo probe at 45°C in Quickhyb solution (Stratagene) and washed with 2xSSC, 0.1% SDS at 45°C. The radioactive signals were detected with a phosphoimaging system (Molecular Dynamics).

### Affymetrix analysis for mRNA expression

We carried out mRNA expression profiles on ES D0, D3 and D6 using Affymetrix mouse 430 2 array. Three biological replicated were performed per time point and 9 arrays were generated in total. Criteria for differential expression: P<0.01 (t-test on log-transformed data), fold change>2 (unlogged data), compared to zero time point.

### miRNA-mRNA enrichment analysis

By gene specificity scores were determined for the set of expressed miRNAs that follow a specific pattern (as defined by group analysis) and therefore fall into a specific class. The gene scoring algorithm examines the extent to which a particular subset of miRNAs are enriched among the family of miRNAs which target each gene. To compute the score, full target site prediction data was obtained by downloading the TargetScan database [36]. We then compute the number of miRNAs which target each gene as well as the number of miRNA targeting the gene, which are dynamically expressed in the embryonic development according to our miRNA expression data. To make the specificity scores more accurate, we excluded those miRNAs we found to be non-expressed in miRNA tissue. The expected number of targeting events for each gene is the number of miRNA targeting events total (for that gene, according to TScan) times the proportion of TargetScan predicted miRNAs which are dynamic in our expression experiment. Scaling by the corresponding variance estimate, we derived Z-scores for each gene for targeting enrichment.

## Supporting Information

Figure S1 Relative contribution of miRNA candidates from SCGAP tissues. SCDb sequences represent Hematopoietic stem cells (HSC) from Princeton SCGAP. StroCDb sequences represent the stromal cell microenvironment of HSCs obtained from Princeton SCGAP. SiEP and GEP or Gastric_EP sequences represent Small intestine Epithelial Progenitors and Gastric Epithelial Progenitors, respectively. These sequences were obtained from Washington University SCGAP. The Baylor SCGAP contributed the hematopoietic stem cell side population (HSC-SP) in the quiescent (HSC-SP Quiescent), 5′ fluorouracil-activated state (HSC-SP- Activated), and sequences from whole bone marrow (WBM) representing the differentiated state. The bi-potential murine embryonic stem cell line (BMEL) was used as a model for liver stem cells (Baylor SCGAP). Overall 12–22% of each category was validated on the microRNA microarray Mouse Array Version 1.
(0.09 MB TIF)

Figure S2 Strategy for targeted discovery of novel miRNAs using k-mer-based microconservation analysis on expressed sequences. All possible k-mers of nucleotides 18–28 were generated from SCGAP transcript sequences. Each sequence was tested in two different ternary alignments of Mouse (SCGAP)-human-rat [m-h-r] and Mouse (SCGAP)-Dog-Cow [m-d-c]. Perfectly conserved k-mers are categorized as micro conserved elements (MCE). Each MCE is mapped on all five genomes and 100 nucleotides of flanking sequences extracted. The ∼200 nt. sequence containing the MCE subsequence is then tested through a microRNA folding filter. All sequences forming a single stem loop structure that satisfies a minimu free energy described in the methods is selected as a novel miRNA candidate (MCE-MIR). Approximately 4600 MCE-MIRs were identified through this work. Approximately, 2600 of the MCE-MIRs were tested on a custom miRNA microarray for expression in the small RNA fraction of ES cells (Mouse Array Version 1). Approximately 545 MCE-MIRs were found to be enriched in ES cells. These were selected to construct Mouse Array Version 2and used for ES and ES (GCNF−/−) time series analyses.
(0.07 MB TIF)

Figure S3 Mouse Array Version 1 image. The first array contained 2617 MCE-MIR predictions, 321 ‘Cand’ predictions, 129 ‘MIR’ predictions and 238 mmu-mirs from miRBase version 7.1. The probes included the <200 nt RNA fraction of adult pool (Cy3) and ES cells (Cy5). RNAs expressed at high levels in fully-differentiated adult tissue appear green (Adult Pool) (Cy3) and RNAs enriched in stem cells appear red (Cy5). The majority of miRNAs in miRBase (upper left-hand panel) appear green. The embryonic stem cell cluster (mmu-mir 290–295) appears red. The lower left-hand panel contains the “star (*) sequences” of the miRBase miRNAs (S-mmu-mir) and can be considered to be negative controls. However, some S-mmu-mir have been shown to have miRNA activity. The probes on the array are MCE-MIR, Cand and MIR they occupy the rest of the array. The majority of MCEs yield An orange color with higher signals from the ES RNA probe(Cy5) masking the lower signals from the Adult Pool (Cy3).
(0.46 MB TIF)

Figure S4 Mouse Array Version 1 summary. This Figure summarizes results relating to the four groups of probes used on Mouse Array Version 1 (mmu-mir, MCE-MIR and Cand/MIR group) using Venn diagrams. This custom array was probed with ES or Adult Pool RNA (<200 nt) fraction (shown in Supplementary Fig. 3). The Venn diagrams summarize the number of miRNAs (mmu-mir) and novel candidates (MCE-MIR, Cand and MIR) that are unique to ES or the Adult Pool and commonly expressed in both.
(0.11 MB TIF)

Figure S5 Expression of miRNAs and predictions in embryonic stem cells (ES) vs. fully Differentiated adult tissue (Adult Pool). MCE-MIR sequences which were derived from adult stem cells through our algorithmic procedure are compared with three other groups. These include the MMU-MIR group which corresponds to miRNAs from miRBase (mmu-mir), the Cand group which corresponds to predictions generated through an algorithm based on phylogenetic shadowing (5) and the MIR group which corresponds to predictions generated through a miRNA target search in 3′ UTR sequences (6). Statistical testing reveals a large number of probe sequences with evidence for differential expression between ES Day 0 cells and the Adult Pool. Panel A depicts the ratio of the frequencies of upregulated probes to those downregulated when ES Day 0 is compared to the Adult Pool. Each class of probe sequence is depicted by a separate bar. Panel B shows the distribution of scores for the differentially expressed probes of each class. The score in panel B depicts the distribution of scores for the difference in mean normalized expression in Adult Pool vs. ES Day 0 (Adult Pool -ES Day 0). Both panels indicate that MCE-MIR probes are dramatically upregulated in ES cells compared to other classes of probes. In contrast, the MMU-MIRs show higher expression in adult cells.
(0.15 MB TIF)

Figure S6 A high-throughput assay to confirm novel miRNA candidates using an ES (Dicer−/−) mutant. This graph summarizes results from a microarray experiment that allowed us to compare expression of novel miRNA candidates and known miRNAs (mmu miRs) in ES cells with and without the enzyme Dicer-1. We expect miRNAs and novel candidates (MCE-MIRs) that are novel miRNAs to exhibit down-regulation in the Dicer−/− mutant. One hundred & six (106) novel MCE-MIRs satisfied these criteria and are likely to be genuine miRNAs. 410 MCE-MIRs did not exhibit a difference in expression between ES and ES (Dicer−/−) mutant as did 50 known mmu-miRs. This could mean either these MCE-MIRs are not miRNAs or that they are novel miRNAs that are not processed in ES cells much like the known mmu-miRs that also exhibited no difference in expression between ES and ES (Dicer−/−). S-mmu-miR detec the passenger strand or the star (*) sequence of mature miRNAs and mmu miR_L probes were designed to hybridize with the loop regions of mature miRNAs.
(0.09 MB TIF)

Figure S7 S7A:Mouse Array Version 2 image. The two samples used Adult Pool (Cy3) vs. GCNF −/− (Cy5). RNAs expressed at high levels in fully-differentiated adult tissue appear green (Cy3) and RNAs enriched in ES cells in which the orphan nuclear receptor GCNF has been knocked out (GCNF −/−) appear red (Cy5). The majority of miRNAs in miRBase (upper left-hand panel) appear green. The embryonic stem cell cluster (mmu-mir-290-295) appears red. The lower left-hand panel contains the “star (*) sequences” of the miRBase miRNAs (S-mmu-mir) and can be thought of as a sort of negative control, although some S-mmu-mir have been shown to have miRNA activity. The probes on the array are MCE-MIR (545), Cand and MIR occupy the rest of the array. The majority of MCE-MIR yield a strong red color. S7B - Hierarchical Clustering of 10 arrays. All microarray data are based on six probe replicates for each miRNA prediction (MCE-MIR, Cand and MIR) and eight probe replicates for mmu-mirs. ES and GCNF samples represent RNA isolated from pooled material from two independent 10 cm dishes with ∼75 million cells each from each time point of the ES and GCNF−/− time series experiment. For each time point, two dishes were pooled to harvest 0.5–1.0 mg of RNA for the arrays. Each time point is represented by duplicate arrays with dye swap of the label. The Adult Pool clearly separates as an independent group from ES and GCNF−/− ES Day 6 following retinoic acid treatment (ESD6) and the GCNF−/− Day 6 following retinoic acid treatment (GCNFD6) appear to be more related to each other as a separate group. During Day 0, Day 1 and Day 3 the ES cells appear to be different from GCNF−/− mutant during that period.
(15.97 MB RTF)

Table S1 SCGAP Source Table. This table lists the SCGAP sources which MCE-MIRs were derived from. The sources are fully described in Material & Methods and Figure S1.
(0.07 MB PDF)

Table S2 Chromosome location of Mouse Array Version 2 MCE-MIR hairpins with unique hits on the genome. This data was derived using the mm7 (Aug 2005) assembly of the Mouse Genome. ST2A shows results from MCE-MIRs with unique hits to the genome and ST2B shows results from MCE-MIRs with multiple hits to the genome.
(0.14 MB PDF)

Table S3 Sequence Cross Reference Table. This table represents MCE-MIR hairpins exhibiting similarity (17 nt≤match) to miRNAs in miRBase or identified through cloning and other predictions.
(0.04 MB PDF)

Table S4 Mouse Array Version 2 data. This table contains the simple detectable values, which lists average signal values of all transcripts on the array, of the ES-GCNF −/− time series. Each array includes 545 MCE-MIR, 266 mmu-mir, 170 Cand, 46 MIR, and 177 S-mmu-mir. A total of 10 arrays were used in this study. This table represents results obtained with the <200 nt RNA probe from the Adult Pool which consist of 18 different fully differentiated tissues from adult mouse. ST4A shows data using small RNA probe from Adult Mouse Panel (Adult Pool). ST4B shows data using small RNA probe from ES. ST4C shows data using small RNA probe from GCNF−/−.
(0.49 MB PDF)

Table S5 ES-GCNF Time Series. This table shows normalized microarray data for ES (ST5A) and GCNF−/− (ST5B). In each case the values have been normalized and interpolated. The actual experiment was carried out with RNA isolated from the ES cells from Day 0, Day 1, Day 3 and Day 6. Values for other days (Days 2, 4 and 5) have been linearly interpolated from these.
(0.39 MB PDF)
