# Supplementary figures and images for: Novel MicroRNA Candidates and miRNA-mRNA Pairs in Embryonic Stem (ES) Cells
Source: PLoS One. 2008 Jul 2;3(7):e2548. doi: 10.1371/journal.pone.0002548 (PMC2481296; doi:10.1371/journal.pone.0002548)

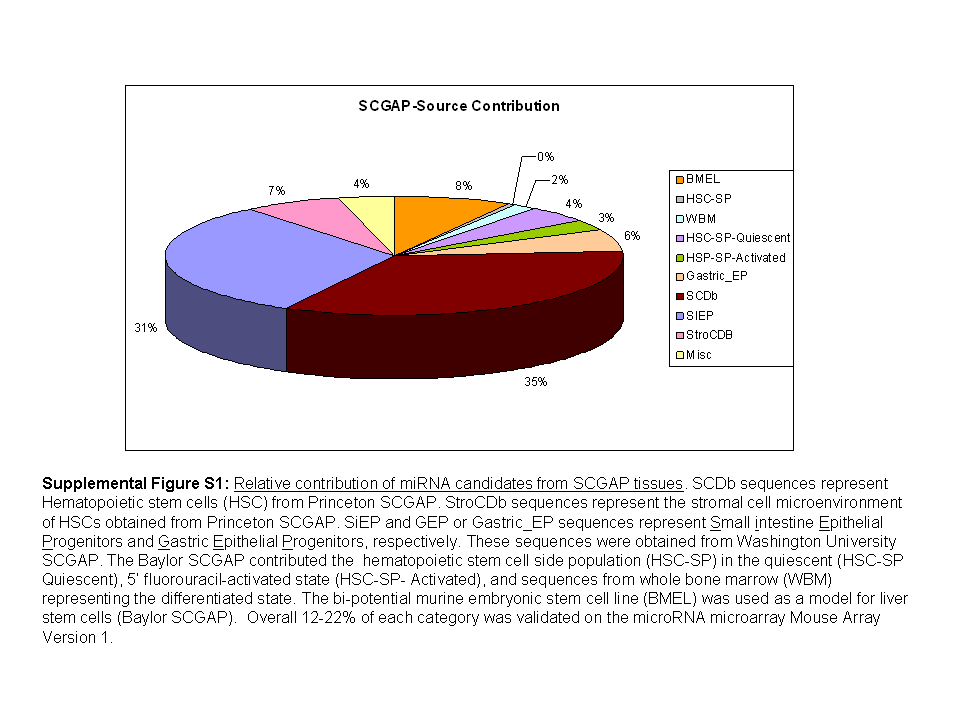

Supplement: Figure S1 — Relative contribution of miRNA candidates from SCGAP tissues. SCDb sequences represent Hematopoietic stem cells (HSC) from Princeton SCGAP. StroCDb sequences represent the stromal cell microenvironment of HSCs obtained from Princeton SCGAP. SiEP and GEP or Gastric_EP sequences represent Small intestine Epithelial Progenitors and Gastric Epithelial Progenitors, respectively. These sequences were obtained from Washington University SCGAP. The Baylor SCGAP contributed the hematopoietic stem cell side population (HSC-SP) in the quiescent (HSC-SP Quiescent), 5′ fluorouracil-activated state (HSC-SP- Activated), and sequences from whole bone marrow (WBM) representing the differentiated state. The bi-potential murine embryonic stem cell line (BMEL) was used as a model for liver stem cells (Baylor SCGAP). Overall 12–22% of each category was validated on the microRNA microarray Mouse Array Version 1. (0.09 MB TIF) [file pone.0002548.s001.tif]

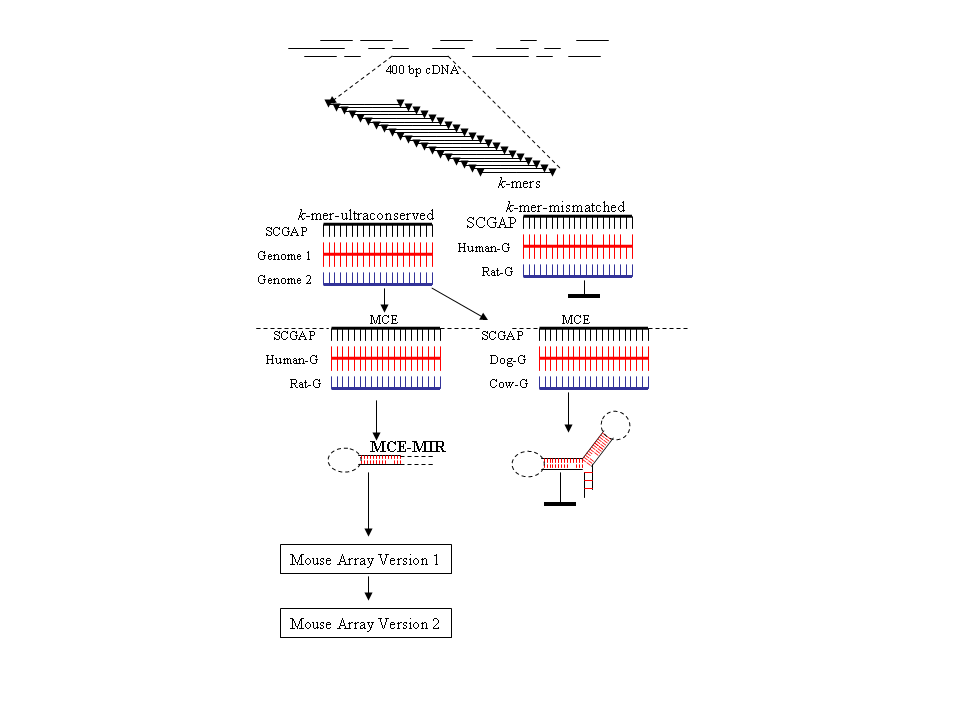

Supplement: Figure S2 — Strategy for targeted discovery of novel miRNAs using k-mer-based microconservation analysis on expressed sequences. All possible k-mers of nucleotides 18–28 were generated from SCGAP transcript sequences. Each sequence was tested in two different ternary alignments of Mouse (SCGAP)-human-rat [m-h-r] and Mouse (SCGAP)-Dog-Cow [m-d-c]. Perfectly conserved k-mers are categorized as micro conserved elements (MCE). Each MCE is mapped on all five genomes and 100 nucleotides of flanking sequences extracted. The ∼200 nt. sequence containing the MCE subsequence is then tested through a microRNA folding filter. All sequences forming a single stem loop structure that satisfies a minimu free energy described in the methods is selected as a novel miRNA candidate (MCE-MIR). Approximately 4600 MCE-MIRs were identified through this work. Approximately, 2600 of the MCE-MIRs were tested on a custom miRNA microarray for expression in the small RNA fraction of ES cells (Mouse Array Version 1). Approximately 545 MCE-MIRs were found to be enriched in ES cells. These were selected to construct Mouse Array Version 2and used for ES and ES (GCNF−/−) time series analyses. (0.07 MB TIF) [file pone.0002548.s002.tif]

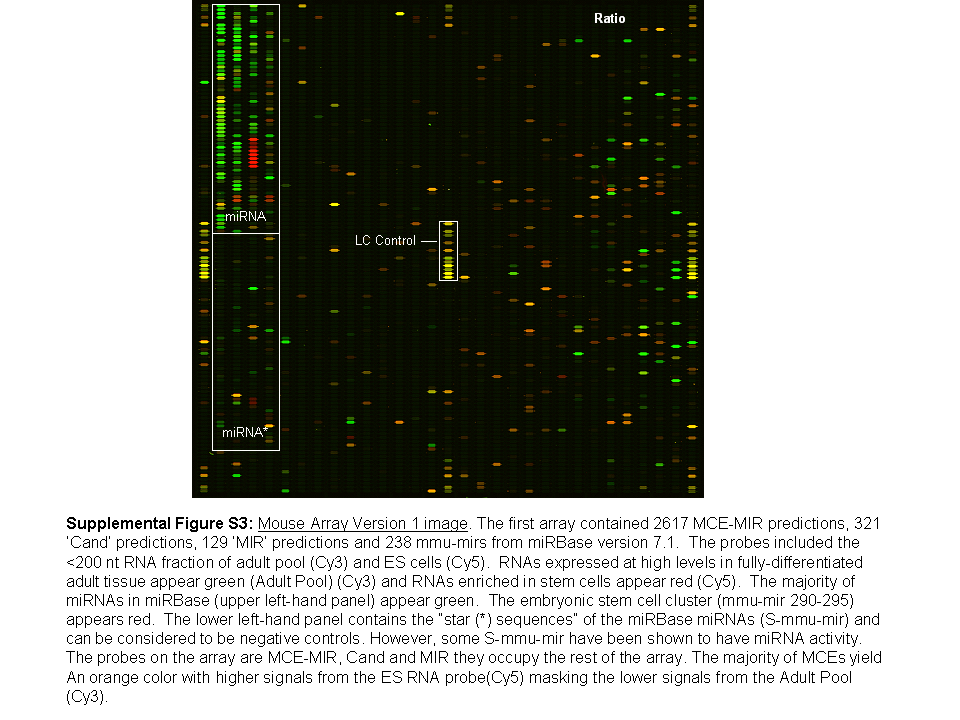

Supplement: Figure S3 — Mouse Array Version 1 image. The first array contained 2617 MCE-MIR predictions, 321 ‘Cand’ predictions, 129 ‘MIR’ predictions and 238 mmu-mirs from miRBase version 7.1. The probes included the <200 nt RNA fraction of adult pool (Cy3) and ES cells (Cy5). RNAs expressed at high levels in fully-differentiated adult tissue appear green (Adult Pool) (Cy3) and RNAs enriched in stem cells appear red (Cy5). The majority of miRNAs in miRBase (upper left-hand panel) appear green. The embryonic stem cell cluster (mmu-mir 290–295) appears red. The lower left-hand panel contains the “star (*) sequences” of the miRBase miRNAs (S-mmu-mir) and can be considered to be negative controls. However, some S-mmu-mir have been shown to have miRNA activity. The probes on the array are MCE-MIR, Cand and MIR they occupy the rest of the array. The majority of MCEs yield An orange color with higher signals from the ES RNA probe(Cy5) masking the lower signals from the Adult Pool (Cy3). (0.46 MB TIF) [file pone.0002548.s003.tif]

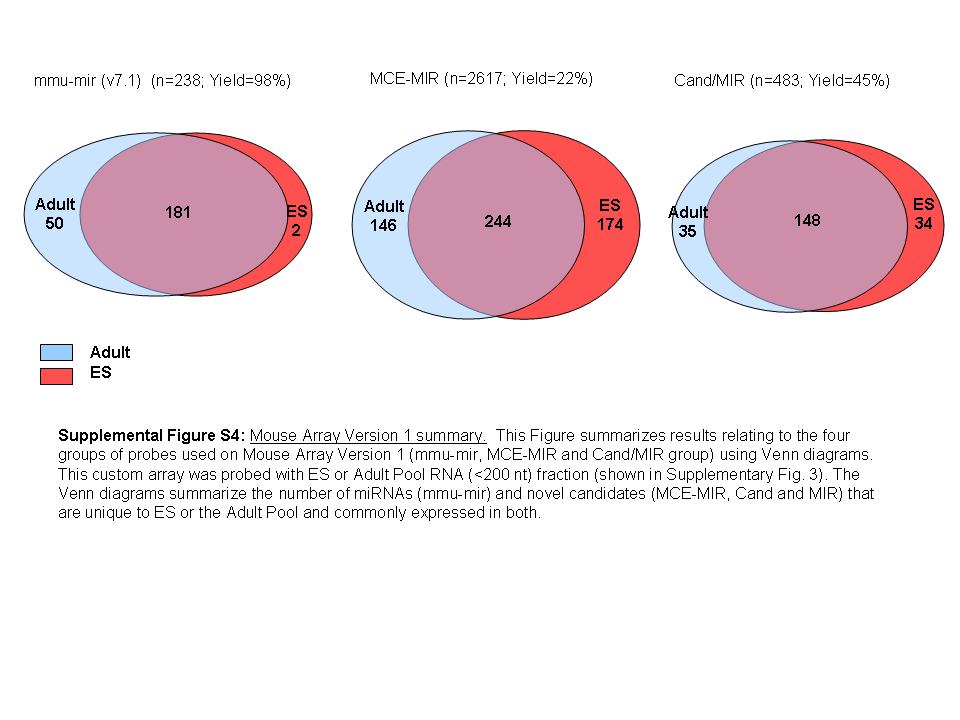

Supplement: Figure S4 — Mouse Array Version 1 summary. This Figure summarizes results relating to the four groups of probes used on Mouse Array Version 1 (mmu-mir, MCE-MIR and Cand/MIR group) using Venn diagrams. This custom array was probed with ES or Adult Pool RNA (<200 nt) fraction (shown in Supplementary Fig. 3). The Venn diagrams summarize the number of miRNAs (mmu-mir) and novel candidates (MCE-MIR, Cand and MIR) that are unique to ES or the Adult Pool and commonly expressed in both. (0.11 MB TIF) [file pone.0002548.s004.tif]

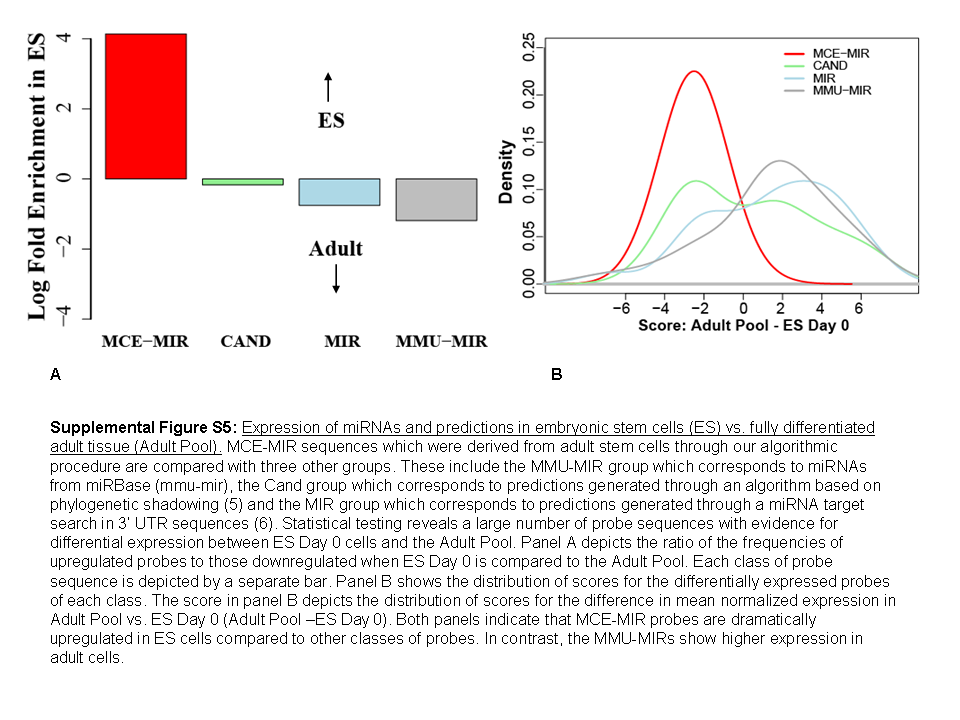

Supplement: Figure S5 — Expression of miRNAs and predictions in embryonic stem cells (ES) vs. fully Differentiated adult tissue (Adult Pool). MCE-MIR sequences which were derived from adult stem cells through our algorithmic procedure are compared with three other groups. These include the MMU-MIR group which corresponds to miRNAs from miRBase (mmu-mir), the Cand group which corresponds to predictions generated through an algorithm based on phylogenetic shadowing (5) and the MIR group which corresponds to predictions generated through a miRNA target search in 3′ UTR sequences (6). Statistical testing reveals a large number of probe sequences with evidence for differential expression between ES Day 0 cells and the Adult Pool. Panel A depicts the ratio of the frequencies of upregulated probes to those downregulated when ES Day 0 is compared to the Adult Pool. Each class of probe sequence is depicted by a separate bar. Panel B shows the distribution of scores for the differentially expressed probes of each class. The score in panel B depicts the distribution of scores for the difference in mean normalized expression in Adult Pool vs. ES Day 0 (Adult Pool -ES Day 0). Both panels indicate that MCE-MIR probes are dramatically upregulated in ES cells compared to other classes of probes. In contrast, the MMU-MIRs show higher expression in adult cells. (0.15 MB TIF) [file pone.0002548.s005.tif]

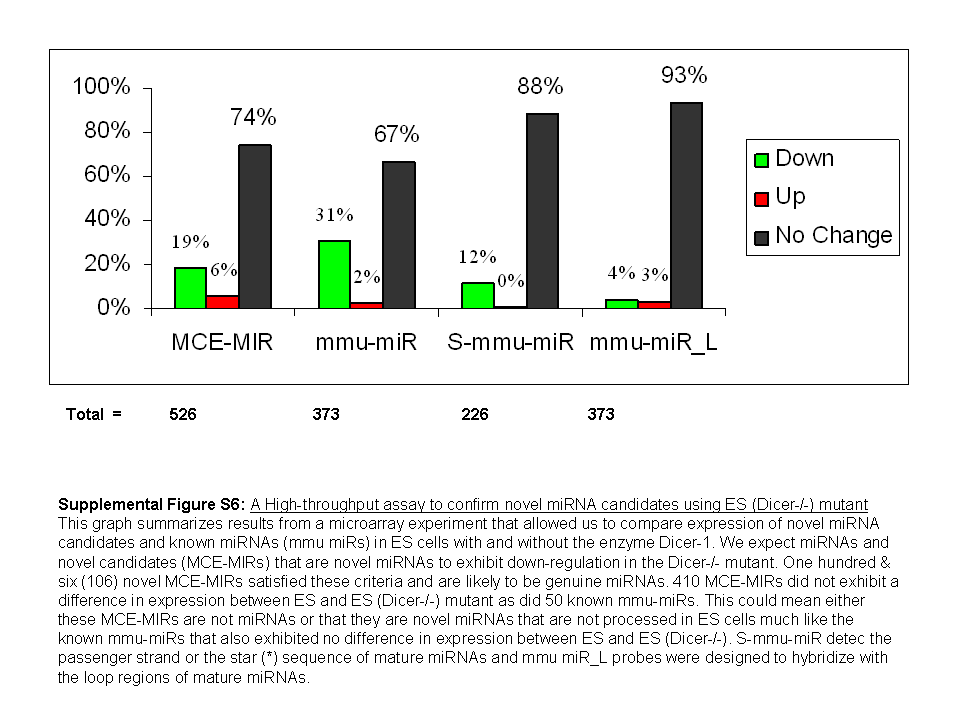

Supplement: Figure S6 — A high-throughput assay to confirm novel miRNA candidates using an ES (Dicer−/−) mutant. This graph summarizes results from a microarray experiment that allowed us to compare expression of novel miRNA candidates and known miRNAs (mmu miRs) in ES cells with and without the enzyme Dicer-1. We expect miRNAs and novel candidates (MCE-MIRs) that are novel miRNAs to exhibit down-regulation in the Dicer−/− mutant. One hundred & six (106) novel MCE-MIRs satisfied these criteria and are likely to be genuine miRNAs. 410 MCE-MIRs did not exhibit a difference in expression between ES and ES (Dicer−/−) mutant as did 50 known mmu-miRs. This could mean either these MCE-MIRs are not miRNAs or that they are novel miRNAs that are not processed in ES cells much like the known mmu-miRs that also exhibited no difference in expression between ES and ES (Dicer−/−). S-mmu-miR detec the passenger strand or the star (*) sequence of mature miRNAs and mmu miR_L probes were designed to hybridize with the loop regions of mature miRNAs. (0.09 MB TIF) [file pone.0002548.s006.tif]
