# Supplementary material for: Novel MicroRNA Candidates and miRNA-mRNA Pairs in Embryonic Stem (ES) Cells
Source: PLoS One. 2008 Jul 2;3(7):e2548. doi: 10.1371/journal.pone.0002548 (PMC2481296; doi:10.1371/journal.pone.0002548)
Supplement: Table S1 — SCGAP Source Table. This table lists the SCGAP sources which MCE-MIRs were derived from. The sources are fully described in Material & Methods and Figure S1. (0.07 MB PDF) [file pone.0002548.s008.pdf]

MCE-MIR	MCE-MIR Sequence	Source	Fold-change in Dicer-/-
MCE-MIR_4854:rev	UGGAAUGGGUGGUGGAGAGCAUC	SCDb	-6.57
MCE-MIR_2310:rev	GUCCCGGCAGGAGCAGAAGAGCAU	SCDb	-6.25
MCE-MIR_5061:rev	GGCAUCAGGUGGUGCCGCUGGGGC	SCDb, StroCDb	-4.99
MCE-MIR_2337:fwd	AUGGCAGCUGGGGCUGCAGA	SCDb	-4.49
MCE-MIR_4673:fwd	GAGAUUCCCACUGUCCCUACCUAC	HSC-SP-Activated	-4.49
MCE-MIR_1371:fwd	AGAGGGGCCUCCAGGCUGGG	SCDb	-4.41
MCE-MIR_4822:rev	GAUUGGAUGGUUUAGUGAGGCCCUCGGAUC	SCDb, StroCDb	-4.36
MCE-MIR_4945:fwd	GCAGGCAGUACAUGGUGUGGGGCAC	SCDb	-4.19
MCE-MIR_822:fwd	ACAGAGGGCUGAGGGGCUCC	SCDb, StroCDb	-4.15
MCE-MIR_4087:fwd	CUCUUCCGUCUGGCUGCUGGGGCCUC	SCDb, StroCDb	-4.12
MCE-MIR_4743:fwd	GAGGCCUUGGGGCGGCCACA	SCDb	-4.10
MCE-MIR_3762:rev	UCAUUCACCUUGAUGAGGGGAUCG	SCDb	-4.08
MCE-MIR_3686:fwd	CCUGGCCUCUGGUGGGGCAG	SCDb	-4.06
MCE-MIR_3609:rev	UUAUACCUGUGGGGCUGAGG	HSC-SP-Activated	-4.02
MCE-MIR_2169:rev	GAGCCGGCGAACCAGGGGUCGAGGAU	SCDb, StroCDb	-3.96
MCE-MIR_3503:rev	GCUCAGUGCUUGGUGACCAGGGG	SiEP	-3.93
MCE-MIR_3416:rev	CUGUGCUGGGCCCAGGGUGGG	SCDb	-3.92
MCE-MIR_5030:rev	UUGUGGGGUGCAGGGCCUGGC	SCDb	-3.86
MCE-MIR_6026:rev	UUACACAAUGACGUGUUGCUGGGGCCUAAUGUUCUCA	SiEP	-3.85
MCE-MIR_5606:fwd	GUCCCUGAGAGGCUGGGUCA	SCDb	-3.82
MCE-MIR_2388:rev	UGAGAUGUGGCUCCAGGGGCUGGCCGGGUACAU	SCDb	-3.79
MCE-MIR_1458:rev	GAUGCAGAUGCGCUUCGACGGGCUGCU	SCDb	-3.76
MCE-MIR_2139:fwd	AUCAUCCGCCUGGAGGGCGUGGUC	SCDb	-3.73
MCE-MIR_5062:rev	GUUGCGGUGAUGGUGACUGGGGC	SCDb	-3.71
MCE-MIR_783:rev	ACCAGAGGGCUGGGCUGUGU	SCDb	-3.62
MCE-MIR_135:fwd	AAAGAGCUGGGCUGGUGCGG	SCDb	-3.60
MCE-MIR_5192:rev	GGGAUCUCCAGGCUGGGGUGGAUGGCAGC	SCDb	-3.56
MCE-MIR_3782:rev	GGGGGCUCAGGAGCUCUUGGUGGGGCG	SiEP	-3.56
MCE-MIR_1504:rev	CAGCCCAGGGUGGAGGUGGCU	SCDb, StroCDb	-3.54
MCE-MIR_968:fwd	ACCAUGCGGACCAGUGGGAC	SiEP	-3.53

MCE-MIR_995:rev	GCCAGAGGGAGAGGCUGGGGU	SCDb	-3.51
MCE-MIR_1792:rev	UGCUUGGGCUGGGUCCGGAUUGCCCU	Misc, SCDb	-3.50
MCE-MIR_136:fwd	AAAGAGCUGGGCUGGUGCGGGCAC	SCDb	-3.49
MCE-MIR_5005:rev	GUCAACUGGGGCAAGGUGGC	SCDb, StroCDb	-3.49
MCE-MIR_3820:rev	GGGGCUCAGGAGCUCUUGGUGGGGCGGGUCCG	GEP, SiEP	-3.44
MCE-MIR_1190:fwd	ACUGUCCCAAUGGGGCAGGGAGGAAAGGUGGCCGCUGGGA	GEP	-3.38
MCE-MIR_4625:fwd	GACUGUCCCAAUGGGGCAGGGAGGAAAGGUGGCCGCUGGGA	GEP	-3.35
MCE-MIR_1325:rev	GCCCAGCUUGGGGAGGGUCU	SCDb, StroCDb	-3.34
MCE-MIR_4661:fwd	GAGAGGCGGGGCUGGGCCGCCCCCAUC	SCDb	-3.32
MCE-MIR_5384:rev	UCUGGGCGGUACUGGCUGGGUCUCCCAGGUCGCC	SCDb, StroCDb	-3.32
MCE-MIR_1409:fwd	AGAUGGGCAUCUGGAGGGCUGG	SCDb	-3.30
MCE-MIR_5440:fwd	GGGCCUGGGGCUGGGCCCAGA	GEP, SiEP	-3.29
MCE-MIR_5403:fwd	GGCUGGGCAGUGGGGCUGCCA	SCDb	-3.28
MCE-MIR_5607:rev	UCCUCAGAGCUCACCCGGAC	SCDb	-3.26
MCE-MIR_4820:rev	UUCACCUUGAUGAGGGGAUC	HSC-SP-Quiescent	-3.23
MCE-MIR_4861:fwd	GAUGGGGCCGGCGUGGGCAA	SCDb, StroCDb	-3.18
MCE-MIR_1506:rev	UGUGUCUCAGGAAGGUGGCU	SCDb	-3.18
MCE-MIR_1394:rev	UUAGAGGUCUUGGGGCCGAAACGAUCU	BMEL	-3.16
MCE-MIR_543:fwd	AAGGUGACCUCGAUCUGGGGGACCCC	SCDb	-3.15
MCE-MIR_5216:rev	UACCUGGACUGGGAGGUGAGAACAGC	SCDb	-3.15
MCE-MIR_3571:rev	GCUCAGGAGCUCUUGGUGGGGCGG	SiEP	-3.11
MCE-MIR_3295:rev	GAGGUUGGCCAUGGGGCUGG	SCDb	-3.11
MCE-MIR_645:fwd	AAUGAAGGGGACCAGGGGCC	SiEP	-3.05
MCE-MIR_4821:rev	UUCAGAGCACUAAGGGGAUC	BMEL	-3.04
MCE-MIR_254:fwd	AACAGGGGCAUGGGGCUGGC	SCDb	-3.03
MCE-MIR_4297:fwd	CUGUCCAUGGGGGCCAGGGA	SCDb, StroCDb	-3.03
MCE-MIR_1365:fwd	AGAGGCCAGCACUGUGGGGC	GEP, SiEP	-3.03
MCE-MIR_5152:rev	GCUGCUGUGGGGCUGGAGUAGC	SiEP	-3.02
MCE-MIR_4922:fwd	GCAGACCCUGCCGGAGCCGCUGCCGC	Misc, SiEP	-3.02
MCE-MIR_4453:fwd	GAACAAUGUAGGUAAGGGAA	HSC-SP-Activated	-2.97
MCE-MIR_3111:fwd	CAUGAAUGGAUGAACGAGAUUCCCACUGUCCCUACCUAC	HSC-SP-Activated	-2.91
MCE-MIR_3490:rev	GUGAACUGGAAGGACUCUCGGGG	SCDb	-2.91
MCE-	GGCGGCUUUGGUGACUCUAGAUA	BMEL,	-2.83

MIR_5389:fwd		GEP	
MCE-MIR_2222:fwd	AUCUUGGCCCCACGGCGGGAAAU	SCDb	-2.82
MCE-MIR_984:rev	GCCACUCUCCGGGGCUGGGU	SCDb	-2.80
MCE-MIR_3468:rev	CUGCAGCCUUGGGGGUGGGG	SiEP	-2.78
MCE-MIR_5418:fwd	GGGAAGGUGGCUGAGGCCCCUGCAUCUCCUGUCUCGAAGUCCAA	BMEL	-2.75
MCE-MIR_3491:rev	GUGAAGGUGCGGCCUCGGGG	SCDb, StroCDb	-2.73
MCE-MIR_5291:fwd	GGAGGCUGGGGGAGGAGUGGGUUGUCAGCA	SCDb, StroCDb	-2.71
MCE-MIR_4273:fwd	CUGGGGCCACCCUGGGGAGC	SCDb	-2.69
MCE-MIR_1773:fwd	AGGCUGUCGGGGAGCAGGGGC	SCDb	-2.65
MCE-MIR_530:fwd	AAGGGACAGCCAGGGCCCUCC	SiEP	-2.64
MCE-MIR_3470:rev	CCGGCUCUUCUGGGAGUGGGG	SCDb	-2.63
MCE-MIR_2327:fwd	AUGGACAUGGACCGGCGGAG	SCDb	-2.60
MCE-MIR_3334:fwd	CCAGGUGGGAGGGCUCUGGG	SCDb	-2.57
MCE-MIR_2243:fwd	AUGAGAACCAGGGCGCUGGGG	SCDb	-2.57
MCE-MIR_5109:rev	GUGCUCAUGUCUGGGGCAGGC	SCDb, StroCDb	-2.54
MCE-MIR_321:rev	UGGACAGGGAGCCUGGGGGAGUU	SiEP	-2.53
MCE-MIR_3485:rev	CUGAUGGGGCGUCCAGGGGG	SCDb	-2.48
MCE-MIR_3847:fwd	CGGCGGCUUUGGUGACUCUAGAUA	SiEP	-2.48
MCE-MIR_3642:rev	CUGGGACUGGGGGACAGAGG	SCDb, SiEP	-2.45
MCE-MIR_1535:rev	GUGGUCAGCUGCAGGGGGCU	SCDb	-2.43
MCE-MIR_3488:rev	GGAAGAGGCAGAGGUCGUCGGGG	SCDb	-2.42
MCE-MIR_335:fwd	AACUGGUUGCAGGGGAAGGC	WBM, SiEP	-2.42
MCE-MIR_3147:fwd	CAUGUCAGCAGGGAUGGGGG	SCDb	-2.29
MCE-MIR_3595:fwd	CCUAGGAGAUGGGGGCAGAG	SiEP	-2.28
MCE-MIR_1539:rev	AUCAAGGGCUGGGACCAGGGGCU	SiEP	-2.22
MCE-MIR_3155:fwd	CAUGUUGGAACAAUGUAGGUAA	HSC-SP-Activated	-2.15
MCE-MIR_1457:rev	AUCAAGGGCUGGGACCAGGGGCUGCU	SiEP	-2.13
MCE-MIR_5544:rev	GUGGGGCAGGGCAGGAUUUAC	SCDb	-2.06
MCE-MIR_5079:rev	UUGGCACUGACGAGGAGCAGGGC	SCDb	-2.04
MCE-MIR_2999:fwd	CAGGGUCAGGCCUGGGAAGG	SiEP	-2.00
MCE-MIR_5699:fwd	GUGUACAUGGAUGGGCGGGGAGGUAACUAAAAGA	SCDb	-1.97
MCE-MIR_3143:fwd	CAUGGUGGCAGGGAGCAGGG	HSC-SP-Quiescent, SCDb, StroCDb	-1.91
MCE-	GCCGGGGAUAGUGGAGCUGCC	GEP	-1.90

MIR_5089:fwd			
MCE-MIR_3837:rev	UGGCCGGGGAGGAGCUGCCG	SCDb	-1.83
MCE-MIR_4740:fwd	GAGGCCCUGUAAUUGGAAUGAGUCC	GEP	-1.80
MCE-MIR_2563:rev	GCCUGUCUGAGCGUCGCUUG	SCDb	-1.73
MCE-MIR_3484:rev	UUCCACAGCUUUGGAGGGGG	SCDb	-1.72
MCE-MIR_2563:fwd	CAAGCGACGCUCAGACAGGC	SCDb	-1.62
MCE-MIR_5195:rev	UCUGGGAGGAAAGGGGGCAGC	SCDb	-1.52
MCE-MIR_5014:fwd	GCCAGCAACACCAUCGGCCAGGA	BMEL	-0.89
MCE-MIR_1538:fwd	AGCCCCUGGGGCUGGCAGGGAGGAG	SiEP	-0.86
MCE-MIR_3190:rev	UCUCAGGGGUCAGGGGCAUUUGG	SCDb	-0.84
MCE-MIR_3191:rev	GCUUCUCAGGGGUCAGGGGCAUUUGG	SiEP	-0.81
MCE-MIR_6054:rev	UUCAUCUGGUAGUUCUCGGGCAGGCA	SCDb	-0.68
MCE-MIR_2164:rev	CUGGAGCAGAGGCUGGGGGAU	SCDb	0.17
MCE-MIR_5643:fwd	GUGAGGGCAGCAGGUGGGAA	SCDb, StroCDb	0.50
MCE-MIR_4274:fwd	CUGGGGCUGGAGCAGGAGGC	SCDb	0.81
MCE-MIR_810:rev	CGGCCUUCUGGCUAAGAUCAAGUGU	SiEP	0.88
MCE-MIR_4791:fwd	GAGUUCAGGACAGCCAGGGC	SiEP	0.91
MCE-MIR_1066:rev	GAGGAGGAAGCAGCCCAGGU	SCDb	1.29
MCE-MIR_3667:fwd	CCUGCAGGAGGCGCAGCACCAG	SCDb	1.39
MCE-MIR_3626:rev	UGAUCCGCAGAGAGGGCAGGAGG	SCDb	1.47
MCE-MIR_2817:fwd	CAGAGCUUGCUCUAUGAGGAGGCAGAA	SCDb	1.57
MCE-MIR_3502:rev	CUCAGAGGGUGGAGGCAGGGG	SiEP	1.71
MCE-MIR_4226:fwd	CUGGAGGCAGGAGGAUGAGA	SCDb	1.93
MCE-MIR_2953:fwd	CAGGAGAGCUGGCAGGAGGA	SCDb	1.94
MCE-MIR_5197:rev	GGUGCCAAGAUGGAGACGGCAGC	GEP, SiEP	2.12
MCE-MIR_5100:rev	GCGGGCAAUAUGUCGGAGGC	SCDb	2.49
MCE-MIR_2745:fwd	CACUGGCGCUUGGGCAGGCGGCAGAAGUCACC	SCDb, SiEP	2.94
MCE-MIR_3543:rev	GCUGUGAGGAGGCAGCAGGG	SCDb	2.95
MCE-MIR_1495:fwd	AGCCAACAGUAGCCACACAAC	SiEP	3.17
MCE-MIR_3134:rev	GAGGCAGGAGGAUUGCCAUG	HSC-SP	3.20
MCE-MIR_3529:rev	GGCAGAGGCUGUCAGGAGGG	SCDb	3.23
MCE-MIR_3651:fwd	CCUGAACACCAUGUUGGCAGC	SCDb	3.33
MCE-MIR_4022:fwd	CUCCCAGCCAAGGUGGCAGC	SCDb, StroCDb	3.33
MCE-	GAGAGGAACACAGCAGCCAU	Misc,	3.69

MIR_2349:rev		SCDb, SiEP	
MCE-MIR_2196:fwd	AUCUGACCAUAGGCAGCAGG	SCDb	3.72
MCE-MIR_4748:rev	UUUCUGGCCUCAGCAGCCUC	SCDb	3.86
MCE-MIR_5193:rev	GGCCACAGGCAGUGGGCAGC	SiEP	3.93
MCE-MIR_1734:fwd	AGGCCAGAGGAGGCAGGUGC	SCDb	3.97
MCE-MIR_1778:fwd	AGGCUUUGGGGGCACAGGAGG	SCDb	4.33
MCE-MIR_3628:rev	UGUGAGCAAAGCAGAAGGAGG	WBM	4.56
MCE-MIR_4202:rev	GCUAUGAAGCCCAGGCAGCAG	HSC-SP-Activated,	4.63
MCE-MIR_4179:rev	GCUUGUUCCUACUGAAGGCAG	SCDb	4.70
MCE-MIR_4726:fwd	GAGGAGGCAGCCUUGGGGCA	SCDb	5.02
MCE-MIR_3059:rev	GCAUAGGAGCUCAUCUGCCACUG	SiEP	5.20
MCE-MIR_5745:fwd	GUUUUCAGGGUUUCAGGAGGCAGCUGAGAA	SCDb	5.43
MCE-MIR_1015:rev	AGGGUUCCAGGACAUAGUCUGAGGCAAGAUGGAGGGU	SiEP	
MCE-MIR_1038:fwd	ACCUCAGUCACAGGGGAGGC	SCDb	
MCE-MIR_1046:rev	CUGGAGGAGCUUGAGGAGGU	SCDb	
MCE-MIR_1052:rev	UCUACCUGCUUUGGAAAGGAGGU	GEP	
MCE-MIR_1059:fwd	ACCUGAGUGAUGACAGAGGC	SCDb	
MCE-MIR_1074:rev	GGGAGGCAGAUGGGACAGGU	SCDb	
MCE-MIR_1192:rev	AGUUUGACUUCCAGGCACAGU	SCDb, StroCDb	
MCE-MIR_1226:fwd	ACUUGUGUGCAUGUGUGUAUA	SCDb	
MCE-MIR_1259:rev	CUGCUAAUAGAAGCCUUGUUCU	SCDb	
MCE-MIR_1264:rev	UCAGGUAGAAGGAGAAGAUGUUCU	HSC-SP-Quiescent, Misc, SCDb	
MCE-MIR_1269:rev	GGACACUUGAUCUGGCGUUCU	SCDb	
MCE-MIR_1283:fwd	AGAAGGUGUUCGCCAGCCUCCC	WBM, Misc	
MCE-MIR_1283:rev	GGGAGGCUGGCGAACACCUUCU	WBM, Misc	
MCE-MIR_1311:rev	CUGCAGAGCCAGGACCUGUCU	SCDb, StroCDb	
MCE-MIR_1342:fwd	AGAGACUGUUUAUUUGUUUGGUGGCUGGGAUGGAACACAAGAUC	HSC-SP	
MCE-MIR_1352:fwd	AGAGCAUGUUUGACACUCUGGGGAUC	BMEL	
MCE-MIR_1356:fwd	AGAGCUUCACGGUGCCAGGG	SCDb	
MCE-MIR_1356:rev	CCCUGGCACCGUGAAGCUCU	SCDb	
MCE-MIR_1364:fwd	AGAGGCAGUGGGGUGGUCUC	SCDb, StroCDb	
MCE-MIR_1365:rev	GCCCCACAGUGCUGGCCUCU	GEP, SiEP	

MCE-MIR_1401:fwd	AGAUGACAGAAGUGAGAAUCCACAGAAGC	BMEL, Misc, SCDb, SiEP	
MCE-MIR_1408:fwd	AGAUGGCUGGAGAGGAGUGA	SCDb	
MCE-MIR_1412:fwd	AGAUGUAACACCAGCUCUUC	SCDb	
MCE-MIR_1433:fwd	AGCAACGGCUGGAUCCUGCC	SCDb	
MCE-MIR_1433:rev	GGCAGGAUCCAGCCGUUGCU	SCDb	
MCE-MIR_1442:fwd	AGCACAUACUCCUCCUUAUGCAGCUG	GEP	
MCE-MIR_1478:fwd	AGCAGUGUUGGUGGAAGGAG	SCDb, StroCDb	
MCE-MIR_1482:rev	UUAAACUUCAGCCCAGUGAUGCU	SCDb, StroCDb	
MCE-MIR_1508:fwd	AGCCACUCGGUGGAGGCCAGCUGGCG	BMEL	
MCE-MIR_151:fwd	AAAGCUCUUUCCCACCUCCU	SCDb, StroCDb	
MCE-MIR_1514:fwd	AGCCAGGAGAAGGAGCGGCU	SCDb	
MCE-MIR_1536:rev	UUCCCUUGGACCUGCCGGGGCU	SCDb	
MCE-MIR_1544:rev	GUACUGUGUGUCAAGGCAGGGCU	SCDb, StroCDb	
MCE-MIR_1546:rev	GCUUCUGUGCAGACAAGGGCU	SCDb, StroCDb	
MCE-MIR_1569:rev	CUUCUGGCCGAGGAAGAGGCU	HSC-SP-Activated, SCDb, SiEP	
MCE-MIR_1576:rev	GCUGGGAUGGAACCCAGGCU	SCDb	
MCE-MIR_1597:fwd	AGCUCCGGGAAGAACGACAUGGCUGCGGCGGGG	SCDb	
MCE-MIR_1611:fwd	AGCUGCAGCUGGAGGCCGAGG	SCDb	
MCE-MIR_162:fwd	AAAGGCUGGAUUGACUGGGC	SCDb	
MCE-MIR_1642:rev	GGCAGCUUUUCUGGGAGAAGAAGCU	SCDb, StroCDb	
MCE-MIR_1645:rev	GCUCCUGUGCCCAAGAAGCU	BMEL, GEP, SCDb, SiEP	
MCE-MIR_1670:fwd	AGGAAGAGGCUCUUGGUGGAGAG	HSC-SP-Activated,	
		Misc, SCDb, SiEP	
MCE-MIR_1679:fwd	AGGACAUCUCUUGGCACCAG	SCDb	
MCE-MIR_1689:rev	CCUGUGCCCAAGAAGCUCCU	BMEL, GEP, SCDb, SiEP	
MCE-MIR_1697:fwd	AGGAGUGUGCAGACUUGUGGCCCA	BMEL, HSC-SP-Quiescent, GEP, SCDb, SiEP	
MCE-MIR_1710:rev	UGGUACGUGGACACCAUCCU	SCDb	

MCE-MIR_1742:rev	AUGGUGAUGAGCUGCUGGGCCU	SCDb	
MCE-MIR_1746:rev	CGGAUAGACAUGGUGAAGGGCCU	SCDb, StroCDb	
MCE-MIR_1756:rev	CAGCACAACGCCUACCGCCU	SCDb	
MCE-MIR_1773:rev	GCCCCUGCUCCCCGACAGCCU	SCDb	
MCE-MIR_1784:fwd	AGGGAGCCUGAGAAUCUGGC	SCDb	
MCE-MIR_1786:fwd	AGGGAGGUCUGCUUGGCAAA	SCDb	
MCE-MIR_1788:fwd	AGGGAGUGUCUCCCCAUACU	SCDb	
MCE-MIR_1788:rev	AGUAUGGGGAGACACUCCCU	SCDb	
MCE-MIR_1793:fwd	AGGGCACCUACCGGCCCAGCAG	SiEP	
MCE-MIR_18:fwd	AAAAAGCCAACAGGGCAGGGGUUUUGU	Misc, SCDb	
MCE-MIR_1811:rev	UCUGACUCAGCAUCGUCCACCCCU	SiEP	
MCE-MIR_1829:rev	CGCAAGCACCUGGCCUACCU	SCDb, StroCDb	
MCE-MIR_1857:fwd	AGGUGCACAAGAGCUGGGGUA	Misc, SiEP	
MCE-MIR_188:fwd	AAAUACAUGUUUCAUAAGACACUGCUA	SiEP	
MCE-MIR_1905:rev	GUGAAGGAACAGUUUGCUUGGAGACAUUUCUACU	BMEL, SiEP	
MCE-MIR_1929:fwd	AGUCCUUCAUUGACAAACCA	BMEL	
MCE-MIR_1931:fwd	AGUCGCUCAGGACAUGGCAGGCA	GEP, SCDb, SiEP	
MCE-MIR_1973:fwd	AGUGGGGGAGGCAUCACCUC	SCDb	
MCE-MIR_1974:fwd	AGUGGGGGAGGUUCUGGAAU	SCDb	
MCE-MIR_1986:fwd	AGUGUUUGCCAGGGCCAGGGCUGC	SiEP	
MCE-MIR_1998:fwd	AGUUGUGCUCCCUCUGGGAC	SCDb	
MCE-MIR_2078:fwd	AUAUGAACAGGAUGAGAGAGA	SCDb	
MCE-MIR_2087:rev	AUUCUGUACAACUUUCUGUACAAUAU	SiEP	
MCE-MIR_2092:fwd	AUCAAACUCCGGGCCACAGGAGGAAA	HSC-SP-Quiescent, GEP, SCDb, SiEP	
MCE-MIR_2099:fwd	AUCAACAAGAUGUUGUCCUGUGCUGGGGCUGACAGGCUCCAGACAGG	BMEL, HSC-SP-Quiescent, HSC-SP-Activated, GEP, SCDb	
MCE-MIR_2111:fwd	AUCAAGGGACGGCUGAACAGACUUCC	HSC-SP-Activated, SCDb	
MCE-MIR_2134:fwd	AUCAGGGCAGAGUAGGUGGC	SCDb, StroCDb	
MCE-MIR_2166:fwd	AUCCUCAGCUCCAGCUGCUC	SCDb, StroCDb	

MCE-MIR_2166:rev	GAGCAGCUGGAGCUGAGGAU	SCDb, StroCDb	
MCE-MIR_2171:rev	UCAACCAGGGUCAAGUCUGGAUUGGAGGCAGGAU	WBM, Misc	
MCE-MIR_2173:rev	CUGGGUAAAGAGUGGAAGGAU	SCDb	
MCE-MIR_2192:rev	GAUCAUGAAGGUGGAAGAGAU	BMEL	
MCE-MIR_2197:fwd	AUCUGAUGCUUAUUGGCCUUGAC	BMEL, HSC-SP-Activated, SCDb	
MCE-MIR_2198:fwd	AUCUGCACUGCCAAGACUGA	BMEL, WBM, HSC-SP-Activated	
MCE-MIR_2205:fwd	AUCUGGAAGUUCUUGACUGA	SiEP	
MCE-MIR_2285:fwd	AUGCAGAGGCAGAAGCCAGGC	BMEL, Misc	
MCE-MIR_2288:fwd	AUGCAGUACAUGGUCUUGUUCUCCCA	GEP, SiEP	
MCE-MIR_2304:rev	GUGCAGGCCAUGAACCGCAU	SiEP	
MCE-MIR_2339:fwd	AUGGCCCGAACCAAGCAGAC	BMEL, Misc, SCDb	
MCE-MIR_2339:rev	GUCUGCUUGGUUCGGGCCAU	BMEL, Misc, SCDb	
MCE-MIR_2345:fwd	AUGGCUCGGAGGGAGAUCAUCUCUGC	SCDb	
MCE-MIR_2361:fwd	AUGGGGGCACUGGGCACAGGUGUGCUG	SCDb, StroCDb	
MCE-MIR_2364:rev	GAGGUAUCCUGACCCUGAAGUACCCCAU	BMEL, WBM	
MCE-MIR_2371:fwd	AUGGUCAGCCUAGACAUCUG	SCDb	
MCE-MIR_2371:rev	CAGAUGUCUAGGCUGACCAU	SCDb	
MCE-MIR_2417:fwd	AUGUUUAGAGUCCUCAUUGG	SCDb	
MCE-MIR_2419:fwd	AUGUUUCCUAUCUAGUUUGUAAAUAAUCAUGGUGCA	SCDb, StroCDb	
MCE-MIR_2464:fwd	AUUGAGGAGCAGAUGGUGGCAG	SCDb	
MCE-MIR_2470:fwd	AUUGGAUGGGCAGAGUAUAGGUGGCGGUGGUUGGUC	SiEP	
MCE-MIR_2474:rev	UAAAACUGAGAAUCACCCCAAU	HSC-SP-Quiescent, SCDb	
MCE-MIR_2501:fwd	AUUUGGAGCAUCUGGAGGAGUG	HSC-SP-Activated, SCDb	
MCE-MIR_2522:rev	GCACAUGCACUUUUAAAAAU	SiEP	
MCE-MIR_2524:fwd	AUUUUUGGCACAGUAAUAUUGCCAAACACACCACC	SCDb	
MCE-MIR_2566:fwd	CAAGCUGCAGGACCUGGGAGCA	SCDb	
MCE-MIR_2566:rev	UGCUCCCAGGUCCUGCAGCUUG	SCDb	
MCE-MIR_2617:fwd	CACAAGCCGACCCAGGAGCC	SCDb, StroCDb	
MCE-	GUGCAUGUGAGCACGUGUGUG	SiEP	

MIR_2624:rev			
MCE-MIR_2661:rev	UUCUCGCCUGUGUGGGUGCGGAUGUG	SCDb	
MCE-MIR_2679:fwd	CACCAGGCGAUGUCUAGCACAGGGGC	SCDb	
MCE-MIR_2680:fwd	CACCAGGGGGCCUCAGCGAG	SCDb, StroCDb	
MCE-MIR_2680:rev	CUCGCUGAGGCCCCCUGGUG	SCDb, StroCDb	
MCE-MIR_2691:rev	UAGAGAGAAUCAAUCUCAAUACUGGCCUGGGUG	BMEL, HSC-SP-Quiescent, Misc, SCDb	
MCE-MIR_2698:rev	GACCUGAGAAACUGCAGGGGGUG	SCDb	
MCE-MIR_2711:rev	CCACUCACCACAGCCAGGUG	SCDb, SiEP	
MCE-MIR_2714:rev	GAGGAGAUGCUGCGGGUGAAGGUG	SCDb, StroCDb	
MCE-MIR_2722:rev	CCUCACUUCAUCCGGCGACUAGCACCGUG	GEP	
MCE-MIR_273:fwd	AACCAAGAUGAUGAUGGCUUCUGGGAAGGGGA	SCDb, StroCDb	
MCE-MIR_2798:fwd	CAGACUCCACAUUGGCUUAAA	GEP	
MCE-MIR_281:rev	GGAUAAAGUGACUCCUGGUU	WBM	
MCE-MIR_2815:fwd	CAGAGCCUGGUGGAGCUGGAG	SCDb	
MCE-MIR_2866:rev	UUUGGGAGUCGCCAUUGUGUCCAUUGGGAGCCUGCUG	SCDb	
MCE-MIR_2889:fwd	CAGCCAUGGCGGUGGAAGGAGGAAUGAA	SCDb, SiEP	
MCE-MIR_2894:fwd	CAGCCCUGAGUCUGGGAGAG	GEP, SiEP	
MCE-MIR_2902:fwd	CAGCCUGGCUCCCAUCCUGGG	SCDb	
MCE-MIR_2902:rev	CCCAGGAUGGGAGCCAGGCUG	SCDb	
MCE-MIR_291:rev	AAGAUGCUGGCAAUAGGGUU	SiEP	
MCE-MIR_293:fwd	AACCGGCAGCUGGAGCAGGA	SCDb	
MCE-MIR_293:rev	UCCUGCUCCAGCUGCCGGUU	SCDb	
MCE-MIR_2968:fwd	CAGGCAGAAAAUGAGGGUGAGGA	WBM	
MCE-MIR_2977:rev	GGCCUCUUGGGCAGGGCCUG	SCDb	
MCE-MIR_298:rev	UUAACUGUGCUAUGGAGUAGAAGCAGGAGGUU	SCDb, StroCDb	
MCE-MIR_2983:rev	GAGGCAUAGAGAGACAGCACCGCCUG	SCDb	
MCE-MIR_2986:fwd	CAGGCUGAGGUGCUGGGCCA	SCDb	
MCE-MIR_3007:rev	CUCCAUGUAUCUUUGGGACCUG	SCDb	
MCE-MIR_3032:fwd	CAGUCACCCAUAAAGUAGAA	SCDb	
MCE-MIR_3048:rev	GAUCAGCACAAUGCCUUCCCAGACUG	HSC-SP-Quiescent	
MCE-MIR_3057:fwd	CAGUGCUACCGAGACAUGGG	BMEL, SCDb, SiEP	

MCE-MIR_3057:rev	CCCAUGUCUCGGUAGCACUG	BMEL, SCDb, SiEP	
MCE-MIR_3084:fwd	CAUACUGGAGAGAAACCCUAUGAAUGUAAUCA	SiEP	
MCE-MIR_3101:rev	UGCCCUGGUCAGGGAGGAUG	SCDb, StroCDb	
MCE-MIR_3113:fwd	CAUGACUUGGCCCACAGUGCC	SCDb	
MCE-MIR_3226:fwd	CCACAGCAGUACCAGGCGAGCA	GEP, SiEP	
MCE-MIR_3260:rev	UUCUGUGGAGUCUGCGUGGGCAUCGUGGGCAGUGG	SiEP	
MCE-MIR_3261:fwd	CCACUGUGCCCUGGGGCUUCCC	BMEL, HSC-SP-Quiescent, GEP, SCDb	
MCE-MIR_329:fwd	AACUGCACCAUGGCUUGGAAGGGGAAAUC	SCDb, StroCDb	
MCE-MIR_3330:fwd	CCAGGGUCCCUUGGACUGGC	SiEP	
MCE-MIR_3333:rev	GGGGCGUCCAGUAGCACCUGG	SCDb	
MCE-MIR_334:rev	GCAGAGGUACCCAUUCCAUUCCCAGUU	SCDb	
MCE-MIR_3379:fwd	CCAUGUUCAUUGGCUCCCAUUUCUGC	SCDb, StroCDb	
MCE-MIR_3407:rev	GUGGAUAAAGGCGAGUGUGUGGG	SCDb, StroCDb	
MCE-MIR_3408:fwd	CCCACACAGGAGUAGGUGGUGCC	SCDb	
MCE-MIR_3429:fwd	CCCAGCACCCGAAUCACUCGAAG	SiEP	
MCE-MIR_3429:rev	CUUCGAGUGAUUCGGGUGCUGGG	SiEP	
MCE-MIR_3439:rev	CUUCAAGAGGAGAGCCUGGG	SCDb, StroCDb	
MCE-MIR_3441:fwd	CCCAGGCUGGUGGCAGGCUGC	SCDb	
MCE-MIR_3444:rev	GGCUGAUGGUCAGCCCUGGG	BMEL, SCDb, SiEP	
MCE-MIR_3471:rev	GUGGACAUCACCUUGCUGGGG	SCDb	
MCE-MIR_3474:rev	CCUCUGGGCCAGCUCCUGGGG	SCDb	
MCE-MIR_3477:rev	UACUUGAUGGAGAUGAGGUAGCUGCCAGGUGCCAUGGGG	SCDb	
MCE-MIR_3478:rev	CUCCCUUUGGACAACACCUUUGUUGGGGG	SCDb	
MCE-MIR_3492:fwd	CCCCGGAUCAGGGCAUGGGA	SCDb, StroCDb	
MCE-MIR_3495:rev	GACCAGGCAUCAGCACAACAGACCGGGG	HSC-SP-Activated	
MCE-MIR_3513:fwd	CCCGGACAUCUAAGGGCAUCACAGA	SCDb, SiEP, StroCDb	
MCE-MIR_3513:rev	UCUGUGAUGCCCUUAGAUGUCCGGG	SCDb, SiEP, StroCDb	
MCE-MIR_3518:rev	UCUGGCUUGCCGUUGGGGUCGUAGGG	SCDb, SiEP	

MCE-MIR_3522:rev	UUUGCCAUCAUCACACAGUGGUGUGAGGG	SCDb, StroCDb	
MCE-MIR_3523:rev	UUCCAGGACAUAGUCUGAGGCAAGAUGGAGGGUGUGAGGG	SiEP	
MCE-MIR_3531:rev	UGGGCCAUGUAGCGCUCGAAGGAGGG	GEP, SCDb, SiEP	
MCE-MIR_3541:rev	GGCUCGCUGGGCACGCAGGG	SCDb, StroCDb	
MCE-MIR_3550:fwd	CCCUUCAGACAGGCCCAGUGCAAGGC	SCDb	
MCE-MIR_3557:rev	UGUCGGAGUUCCCGGCCAGGGAAGAGAAGGG	SCDb, StroCDb	
MCE-MIR_3572:rev	GCCGACGGCAUGUGCACCCAGGGCGG	SCDb	
MCE-MIR_3573:rev	GAGGCCUUCGCCAUCCUGGACGGCGG	SiEP	
MCE-MIR_3613:rev	UGUGUGAUGAGCUGGUGGAGG	SCDb	
MCE-MIR_3619:rev	UGGUAAGUUGGAAAUGGAGG	BMEL	
MCE-MIR_3624:fwd	CCUCCUCGCCAAAGACAGCC	HSC-SP-Activated, SCDb, SiEP	
MCE-MIR_3624:rev	GGCUGUCUUUGGCGAGGAGG	HSC-SP-Activated, SCDb, SiEP	
MCE-MIR_3637:rev	GAUAUGAAUCCCUUCCUCAGAGG	SiEP	
MCE-MIR_364:fwd	AAGAACUGGAAGGCCAUGGAGGC	SCDb	
MCE-MIR_3646:rev	CUCUUGAGAGCUGGAAGAGG	SCDb	
MCE-MIR_3653:rev	UCCAGCUGGGGGAUGUCAGG	GEP	
MCE-MIR_3663:fwd	CCUGCAGAAGGUGUGCAGACACUCACGCAGCACCACGGCCUC	SCDb	
MCE-MIR_3667:rev	CUGGUGCUGCGCCUCCUGCAGG	SCDb	
MCE-MIR_3684:rev	UCUGAAGACUGUGGGCCAGG	SCDb	
MCE-MIR_3685:fwd	CCUGGCCGCCGGUGAUAAACUAGCUC	SCDb	
MCE-MIR_3685:rev	GAGCUAGUUUAUCACCGGCGGCCAGG	SCDb	
MCE-MIR_3695:fwd	CCUGUGCCCAAGAAGCUCCUGAUGAUGGC	BMEL, GEP, SCDb, SiEP	
MCE-MIR_3715:rev	UUUGUUCUGGCUCCAGAAGG	SCDb	
MCE-MIR_3751:fwd	CGAGCCCAGGCAGGAACCCCUGGUGCAGGCAGGCUUA	SCDb	
MCE-MIR_3754:rev	CGGUACAUCUGGGCAGCUCG	SCDb	
MCE-MIR_3780:rev	UGGAGAUGCUGGAGGCGCUGGUGAAUGAGGGCAGCCUGGCG	SCDb	
MCE-MIR_3791:rev	CGUUGUGAGAUCCCAGAGGCG	BMEL, SCDb	
MCE-MIR_3793:rev	UGCUUCAGGGACAUGAGCACCGAGCGCAGGCG	SCDb	
MCE-MIR_3797:fwd	CGCUCAGGACAUGGCAGGCA	GEP, SCDb,	

		SiEP	
MCE-MIR_3820:fwd	CGGACCCGCCCCACCAAGAGCUCCUGAGCCCC	GEP, SiEP	
MCE-MIR_3832:rev	GAGCACUUCUCCAUGAUCCG	SCDb	
MCE-MIR_3841:fwd	CGGCCAGCAGAGCUGUCAGAUGAGGAAG	SCDb	
MCE-MIR_3847:rev	UAUCUAGAGUCACCAAAGCCGCCG	SiEP	
MCE-MIR_3859:fwd	CGGCUUUGGUGACUCUAGAUA	BMEL, GEP, SiEP	
MCE-MIR_3859:rev	UAUCUAGAGUCACCAAAGCCG	BMEL, GEP, SiEP	
MCE-MIR_3867:rev	GACUUCUAUACCUCCAUUACCCG	BMEL, HSC-SP-Quiescent, SCDb, SiEP	
MCE-MIR_3886:fwd	CGGUGAUGGUAGCCUUUCUGCCCAGC	GEP, SCDb, SiEP	
MCE-MIR_3886:rev	GCUGGGCAGAAAGGCUACCAUCACCG	GEP, SCDb, SiEP	
MCE-MIR_3888:rev	GGGGUGAGGCCCUCACUUCAUCCGGCGACUAGCACCG	GEP	
MCE-MIR_3958:fwd	CUCAAGCGCCGCCUGGCGGGC	SCDb, StroCDb	
MCE-MIR_399:fwd	AAGAGCCUGGUGAGCAAGGG	SCDb	
MCE-MIR_4010:rev	GAGGUGCUGGAGCAGGUGGAG	Misc, SCDb	
MCE-MIR_4015:fwd	CUCCAUCUCCUAAACAAGCAC	SCDb, StroCDb	
MCE-MIR_4027:rev	GGCUUUCACUUGGGCAGAGGGAG	GEP, SiEP	
MCE-MIR_4030:rev	UGGGGAAACUGAGGCCCGGAG	SiEP	
MCE-MIR_4031:fwd	CUCCGGUCAAUCAGCACCCA	SCDb	
MCE-MIR_4034:rev	UCCGGGUGAGCUCUGAGGAG	SCDb	
MCE-MIR_405:rev	GUUGAUGCCACCUGACUCUU	SiEP	
MCE-MIR_406:rev	GGAGGACCCGGCGGGCCACACUCUU	BMEL	
MCE-MIR_4060:rev	GACUGCCACAAUACAAGUCAGGAGAG	GEP, SiEP	
MCE-MIR_4061:rev	CUGACCAUGUGCAUCAAGGAGAG	SCDb	
MCE-MIR_4063:fwd	CUCUCUGACCGUUACGUGAGUCACUUUGAGAC	SCDb, StroCDb	
MCE-MIR_4063:rev	GUCUCAAAGUGACUCACGUAACGGUCAGAGAG	SCDb, StroCDb	
MCE-MIR_4069:rev	GAGAAGUUCUCCAGGCAGAG	SCDb	
MCE-MIR_407:fwd	AAGAGUUCAGCAAGACCACU	SiEP	
MCE-MIR_4087:rev	GAGGCCCCAGCAGCCAGACGGAAGAG	SCDb, StroCDb	
MCE-MIR_4124:fwd	CUGAUACUUGGCUGCUAUUCC	BMEL, SCDb	
MCE-	CUGCACCAGGCUGAGGACAG	SCDb	

MIR_4153:fwd			
MCE-MIR_4179:fwd	CUGCCUUCAGUAGGAACAAGC	SCDb	
MCE-MIR_4182:rev	UUCAGCAUCGCAGACAAGAAGCGCAG	BMEL, SiEP	
MCE-MIR_4198:fwd	CUGCUGCAGGGAGGCCUGGC	GEP, SiEP	
		Misc, SCDb	
MCE-MIR_4207:fwd	CUGCUUCUCCUUCUUGGCCAC	SCDb	
MCE-MIR_4209:fwd	CUGCUUGGAGUGUUCUGGCA	SCDb	
MCE-MIR_4232:fwd	CUGGAUGAUGUCACGGCAGUCGUUGAA	GEP, SCDb, SiEP	
MCE-MIR_4236:rev	UGGGAAGUUACUGGUGCCAG	SCDb	
MCE-MIR_4239:rev	GGCUGCCUGGGGUCUGCCAG	SCDb, StroCDb	
MCE-MIR_4280:fwd	CUGGUAGAAGCAGGGCUUCA	SCDb, StroCDb	
MCE-MIR_4280:rev	UGAAGCCCUGCUUCUACCAG	SCDb, StroCDb	
MCE-MIR_4303:fwd	CUGUGAACUGCUCUGAGAUGC	BMEL	
MCE-MIR_4320:rev	GGUGGCCAAGGGGAGACAACAG	SiEP	
MCE-MIR_4342:fwd	CUUCAUCUUGUGUUGAGACUUC	Misc, SCDb	
MCE-MIR_4345:rev	UCAGCUGGUGGGGUCAGUGGAAG	SiEP	
MCE-MIR_4353:rev	UGACCAGCUACCAGAGGAAG	SCDb, StroCDb	
MCE-MIR_4383:rev	GAGCAGGUGAAGCAGGCAAG	SCDb	
MCE-MIR_4413:fwd	CUUUGUAAAGACUAAAACACA	SiEP	
MCE-MIR_4442:fwd	GAAAGCCAGGCAGCUGAUGGC	SCDb	
MCE-MIR_4449:rev	UUACUAACAUCACGAAUUUC	SCDb	
MCE-MIR_4462:fwd	GAACUAUAAUACUAACAGGUUCUUGGGGAGGGAC	SCDb	
MCE-MIR_4472:fwd	GAAGAAGUCUUACACCACUCCCAAGAAGAAUAAGCA	Misc, SCDb	
MCE-MIR_4474:fwd	GAAGACUGUCACUCUCCAAC	GEP, SCDb	
MCE-MIR_4491:fwd	GAAGCUACUGUGUGUGUGAAUGAACACUC	GEP, Misc, SiEP	
MCE-MIR_4491:rev	GAGUGUUCAUUCACACACACAGUAGCUUC	GEP, Misc, SiEP	
MCE-MIR_4493:fwd	GAAGCUGACCAGAGAUGAGAC	BMEL, SCDb	
MCE-MIR_4497:rev	GUAGAGCGGUGGGUCUCCUUC	SCDb	
MCE-MIR_4503:rev	UGUUAACUCACAAGGAAACACCUUC	SCDb, SiEP	
MCE-MIR_451:rev	GUGCUGUCCCCCUACUCCUGGCUGGGCUU	SCDb	
MCE-MIR_4513:fwd	GAAUCAAUCUCAAUACUGGCCUGGGUGCUGGA	BMEL, HSC-SP-Quiescent, SCDb	

MCE-MIR_4521:fwd	GAAUGCUGGCUUCAGUGUGUC	SCDb, StroCDb	
MCE-MIR_4521:rev	GACACACUGAAGCCAGCAUUC	SCDb, StroCDb	
MCE-MIR_4554:rev	UGCUACUGCUGUCCCCUGUC	SCDb	
MCE-MIR_4592:fwd	GACCUGGGCUUAGACAGUUUGGACCAAGUGGA	GEP, SiEP	
MCE-MIR_4607:fwd	GACUCCAUGACAGCUGCAAA	SCDb	
MCE-MIR_4610:fwd	GACUCUGGUGUCUCUAAGGGAGC	SCDb	
MCE-MIR_4614:rev	GGAAUCCCUCCUGAGCAGUC	SCDb	
MCE-MIR_4627:fwd	GACUGUGGGGCAGCUCCUGGGGCC	SCDb	
MCE-MIR_463:fwd	AAGCCUGUGACUGUCCAUUCCC	BMEL, SCDb, SiEP	
MCE-MIR_466:fwd	AAGCGGAUCUUCACCUGGUC	SCDb	
MCE-MIR_4661:rev	GAUGGGGGCGGCCCAGCCCCGCCUCUC	SCDb	
MCE-MIR_4667:fwd	GAGAUGCUGGUGGACCUCCAGUA	SCDb	
MCE-MIR_4674:fwd	GAGCACAGACAGGAUCGCAGGGAGAGGCC	GEP, SCDb, SiEP	
MCE-MIR_4675:fwd	GAGCACAGAGGAGGCUGCCA	SCDb	
MCE-MIR_4684:fwd	GAGCAGGUGCAUGGGGCAGC	SCDb	
MCE-MIR_469:fwd	AAGCUCAAAGCCUCCAGCCGCAC	SCDb	
MCE-MIR_4711:fwd	GAGCUGCAGCACAGCCUCCA	SCDb	
MCE-MIR_4712:fwd	GAGCUGCAGUAUCGGCCCUC	SCDb, StroCDb	
MCE-MIR_4712:rev	GAGGGCCGAUACUGCAGCUC	SCDb, StroCDb	
MCE-MIR_4714:fwd	GAGCUGCGUGUGGCCCCUGAGGAGCACCC	BMEL, WBM	
MCE-MIR_4714:rev	GGGUGCUCCUCAGGGGCCACACGCAGCUC	BMEL, WBM	
MCE-MIR_4716:fwd	GAGCUGGCAGGAGAAGGCAUUGGGGA	SCDb	
MCE-MIR_4726:rev	UGCCCCAAGGCUGCCUCCUC	SCDb	
MCE-MIR_4738:fwd	GAGGCCAGAGUGUGAGGAGC	SCDb	
MCE-MIR_4745:fwd	GAGGCUCAGUCCCAGCUCAUC	SiEP	
MCE-MIR_4752:rev	GGCCUGGCACCUCUGCCCUC	SCDb	
MCE-MIR_4755:fwd	GAGGGCAGGCCCUGGGCUCC	SCDb	
MCE-MIR_4756:rev	UUAAAGAUGGCCAGCAAGCCCUC	BMEL	
MCE-MIR_4762:fwd	GAGGUAUCCUGACCCUGAAGUACC	BMEL	
MCE-MIR_4763:fwd	GAGGUAUCCUGACCCUGAAGUACCCC	SiEP	
MCE-MIR_4789:rev	UUCAAGUGUCUGUUGAACUC	SCDb	
MCE-MIR_4791:rev	GCCCUGGCUGUCCUGAACUC	SiEP	

MCE-MIR_4799:rev	GGAUGUUCUGCACAGCAAGUGUAGACAGGCAGACACAUGACAACUC	Misc	
MCE-MIR_4809:fwd	GAUAUAAUGGCCAAGAGGAAUCAGAAACC	HSC-SP-Activated, SCDb	
MCE-MIR_482:fwd	AAGCUGGAGUUCUCCAUUUACC	BMEL,	
		HSC-SP-Quiescent	
MCE-MIR_482:rev	GGUAAAUGGAGAACUCCAGCUU	BMEL,	
		HSC-SP-Quiescent	
MCE-MIR_4830:fwd	GAUCUGGCACCACACCUUCUACAAUGAGCUGCGUGUGGC	BMEL, WBM	
MCE-MIR_4832:rev	UGCAUCAACAUCAAUUUAGCAGAGGAAGAUC	WBM	
MCE-MIR_4853:fwd	GAUGCUCAUCUUCUCCUGGGCA	SCDb	
MCE-MIR_4893:fwd	GCAAAGUUGCUGGUGAGGACCAC	SCDb	
MCE-MIR_4893:rev	GUGGUCCUCACCAGCAACUUUGC	SCDb	
MCE-MIR_4913:rev	GUGGGCGGGCAGCUGACCGAGUGC	SCDb, StroCDb	
MCE-MIR_4922:rev	GCGGCAGCGGCUCCGGCAGGGUCUGC	Misc, SiEP	
MCE-MIR_4932:rev	GUGAAGAACUUUGGCAUCUGGCUGC	BMEL, SCDb, SiEP	
MCE-MIR_4972:fwd	GCAUUACAGAGAGGAGAAACACGUCUUCCCUCGA	SCDb	
MCE-MIR_4978:rev	GUAGCAGAGAUCACCAAUGC	BMEL	
MCE-MIR_4999:rev	UUCCAACUGGGCAUCAUAAAUUUGAGGUGGUGGC	StroCDb	
MCE-MIR_5004:rev	UACCACUGCGAGGCCGAGCAGGUGGC	SCDb	
MCE-MIR_5008:rev	UGCUUGAGGUCCGACGUGGC	SCDb	
MCE-MIR_5033:rev	UGUGCCCAAGAAGCUCCUGAUGAUGGC	HSC-SP-Activated	
MCE-MIR_504:fwd	AAGGAUCUGGUCAUCUUGCU	BMEL, SCDb	
MCE-MIR_5046:rev	GGUUGCAAACCAUACAUGGC	SCDb	
MCE-MIR_5055:fwd	GCCCAGCACCUCAGGGUGAC	GEP, SCDb	
MCE-MIR_5055:rev	GUCACCCUGAGGUGCUGGGC	GEP, SCDb	
MCE-MIR_5056:rev	UUCAGGCUCAUGGAGCAGUGCUGGGC	SCDb	
MCE-MIR_5057:fwd	GCCCAGCGGCGGGUGAGGAAGCUGCC	SCDb, StroCDb	
MCE-MIR_5060:rev	GGGCUGAGCACCAAGGCAAGCGGGUGGGCUGGGGC	GEP, SiEP	
MCE-MIR_5068:rev	UCAUGGUAGGCUUUCUCAGCAGAGAUGACAGGGGC	BMEL, SCDb, SiEP	
MCE-MIR_5083:rev	GUGAGGACAGGAGCCAGGGC	SCDb	
MCE-MIR_5088:rev	UCCUUCACCCAGUGGUCAGCGGC	SCDb	
MCE-MIR_5105:rev	GUGGGUUUGGCACUAAGAGGC	SCDb	

MCE-MIR_5122:rev	UUGGUGCCCCUUUUUGAGAAGGC	HSC-SP-Quiescent, Misc	
MCE-MIR_5135:fwd	GCGCGGAGGAUGGUGUGGAAA	SCDb	
MCE-MIR_5141:rev	UGCUCUGGCGCGGGGCUUCACGGCCGC	SCDb, StroCDb	
MCE-MIR_5143:rev	UCAGCAUCGUGGACUACCGC	SCDb	
MCE-MIR_5152:fwd	GCUACUCCAGCCCCACAGCAGC	SiEP	
MCE-MIR_5167:fwd	GCUCCUGGCCAUCUUCAACA	SCDb, SiEP	
MCE-MIR_5167:rev	UGUUGAAGAUGGCCAGGAGC	SCDb, SiEP	
MCE-MIR_5172:fwd	GCUCUGGUCUGCGAUGGGGCCACGGAGGAGAC	SCDb	
MCE-MIR_5180:fwd	GCUGAGCAGGCCCAGGCCUUCAAGAA	SCDb, StroCDb	
MCE-MIR_5180:rev	UUCUUGAAGGCCUGGGCCUGCUCAGC	SCDb, StroCDb	
MCE-MIR_5210:fwd	GCUGGUGAGAUUGGAGAGAUGAAGGAUGGAGUCCC	SCDb	
MCE-MIR_5236:rev	UGCUCCUGUGCCCAAGAAGC	BMEL, GEP, SCDb, SiEP	
MCE-MIR_5260:fwd	GGAAUUGACUUCUAUACCUCCAUUACC	BMEL, HSC-SP-Quiescent, SCDb, SiEP	
MCE-MIR_5276:fwd	GGACCCAAGAGGCAGGUGGA	SCDb	
MCE-MIR_5279:fwd	GGACCUUGGCUACCCUGAGA	SCDb, StroCDb	
MCE-MIR_5287:rev	UGCCUGCUCUCAUUGUUGCUCAAUUUGCUCC	SiEP	
MCE-MIR_5295:rev	GGCAUGAGAAGGCCCCCUCC	SCDb	
MCE-MIR_5300:fwd	GGAGUCAUGUUGCAGAGCCC	SCDb, StroCDb	
MCE-MIR_5300:rev	GGGCUCUGCAACAUGACUCC	SCDb, StroCDb	
MCE-MIR_5303:fwd	GGAGUUGGUGGUCAGAUGCAGGA	BMEL, Misc, SCDb	
MCE-MIR_5322:fwd	GGCAAGGCCCCAGGAACCCC	BMEL, SCDb, SiEP	
MCE-MIR_5322:rev	GGGGUUCCUGGGGCCUUGCC	BMEL, SCDb, SiEP	
MCE-MIR_5328:rev	GGGCUGGGCUCUCCCUGGCUAGUGCC	SCDb	
MCE-MIR_5339:rev	GUGCUGCAGCGAGCCCUGGAGUGCCUGCC	SCDb	
MCE-MIR_534:fwd	AAGGGGGAGGGUCCUGGAGGUGA	BMEL	
MCE-MIR_5340:fwd	GGCAGGCCAGGCUCUGGAGGA	SCDb	
MCE-MIR_5354:rev	GUUAAGUGCUCCAAGGAGGUGGCC	SCDb	
MCE-MIR_5363:fwd	GGCCCCAGGGGUGGCUCCUCA	SCDb, StroCDb	
MCE-	UGAGGAGCCACCCCUGGGGCC	SCDb,	

MIR_5363:rev		StroCDb	
MCE-MIR_5366:rev	GGGCACUCCUGGCAAUCCAUCUUCACCUUGGAGGGGCC	HSC-SP-Activated, Misc	
MCE-MIR_5367:rev	UCUAGAAGCAUUUGCGGUGGACGAUGGAGGGGCC	SiEP	
MCE-MIR_5369:rev	UACGCCAUGAACUGGAGCGUGCGGCC	SCDb	
MCE-MIR_5374:fwd	GGCCUCAUGCACGGGAAGAC	SCDb	
MCE-MIR_5374:rev	GUCUUCCCGUGCAUGAGGCC	SCDb	
MCE-MIR_5389:rev	UAUCUAGAGUCACCAAAGCCGCC	BMEL, GEP	
MCE-MIR_5396:rev	GUGUAACUUGAAGCUAAUUUGUACUACUGGAUAUCUGACUGGAGCC	BMEL, Misc, SCDb, StroCDb	
MCE-MIR_5399:fwd	GGCUGAAAAUGGUGGAAAAGGACCAAGAUGGGGGCCGCAA	BMEL, SiEP	
MCE-MIR_5406:rev	UGGAGUUCUCCAUUUACCCAGCC	BMEL, HSC-SP-Quiescent	
MCE-MIR_5411:rev	GGGUGGGGGUCACAGUGCACGAAGCC	SCDb	
MCE-MIR_5443:fwd	GGGCGGACUCUCCGGGAGAC	SCDb	
MCE-MIR_5454:rev	UGGCCCAGGAACAGAGGUCCCC	SiEP	
MCE-MIR_5470:fwd	GGGGUCAAGGGACACGCCUUCUGA	SCDb	
MCE-MIR_5473:fwd	GGGGUGCUGGAUGAGGGCAA	SCDb	
MCE-MIR_5473:rev	UUGCCCUCAUCCAGCACCCC	SCDb	
MCE-MIR_5488:rev	UUCCUGAACCGAAUCCACAAGAAGCACCC	SCDb	
MCE-MIR_5503:rev	GUGAAGGCACCAGGCUGACC	HSC-SP-Quiescent, SCDb, SiEP	
MCE-MIR_5504:rev	UGCAGAACUGGAGUCUUUACUGACC	SCDb	
MCE-MIR_5511:rev	UUGGGCAGGCGGCAGAAGUCACC	SCDb, SiEP	
MCE-MIR_557:fwd	AAGUACGCACACUCAUCCCC	SiEP	
MCE-MIR_5581:fwd	GUCACCACCUUGGUGGAGAAC	SiEP	
MCE-MIR_5581:rev	GUUCUCCACCAAGGUGGUGAC	SiEP	
MCE-MIR_5596:rev	UUUGUUGAGGAAGAGGAUGAC	SCDb	
MCE-MIR_5597:fwd	GUCAUGCCCUGCUGCACCAC	SCDb, StroCDb	
MCE-MIR_5598:fwd	GUCAUUGAACACAUCCAGGUGAACAA	BMEL, HSC-SP-Activated, GEP, SCDb	
MCE-MIR_5620:fwd	GUCUGACACAAUUUGAGCUUGCUAUAGCAA	SCDb	
MCE-MIR_5623:rev	UAUGAGCUGUUCUUCAAGAC	SCDb	
MCE-MIR_5641:rev	UGAAGCAUCCCAGGCGGCUCAC	SCDb, StroCDb	

MCE-MIR_5704:rev	UCAUCAUCCCACAUAGACAC	SiEP	
MCE-MIR_5712:rev	UGGAACACAUGUGCACACAC	SCDb, StroCDb	
MCE-MIR_5736:rev	UGAGAACAUUAGGCCCCAGCAAC	SiEP	
MCE-MIR_5790:fwd	UACAUGGCCAGCACGAAGUGGAAGAAGUUGGA	SCDb, StroCDb	
MCE-MIR_5864:fwd	UCAAUUCCAAGCUCUUCUAGUCCCAA	BMEL	
MCE-MIR_5872:fwd	UCACCAUCGCCAGGGAGGGCCUUGAGGACAA	BMEL, SCDb	
MCE-MIR_5914:rev	UCGUACAUGGCCAGCACGAAGUGGAAGAAGUUGGA	SCDb, StroCDb	
MCE-MIR_5970:rev	UUGGCUCAGGGUUUGACAGAGA	GEP, SiEP	
MCE-MIR_6001:rev	UGCUGGUUGGAGGACGAAGA	SCDb	
MCE-MIR_6026:fwd	UGAGAACAUUAGGCCCCAGCAACACGUCAUUGUGUAA	SiEP	
MCE-MIR_6033:fwd	UGAUCCCCUCAUCAAGGUGAA	BMEL	
MCE-MIR_6034:rev	UUCUUGGCCUUUUGGCUAAGAUCA	SiEP	
MCE-MIR_6050:rev	UGCUUGGCCUGGCGCAGGGCA	BMEL, SCDb	
MCE-MIR_6055:rev	UUGUAGAGCAGGGAGCGCAGGCA	SCDb	
MCE-MIR_6084:rev	UGUUCCUCAAAAGCUGCCCA	SCDb, StroCDb	
MCE-MIR_6107:fwd	UGUGGGGACCUAGAGGAGGAGCUGAA	SCDb	
MCE-MIR_6120:fwd	UUAUGGAAUUGAUUUGCAUUGAACACAAACUGUAAAUAAAA	GEP, Misc, SiEP	
MCE-MIR_638:fwd	AAUCGGUCUUGUCUGGUGGUGGCAUU	SCDb, SiEP, StroCDb	
MCE-MIR_670:fwd	AAUGGAGGUGGGCAGGUGUC	SCDb, StroCDb	
MCE-MIR_689:rev	CUUUGUGGUCACUGUAACAUU	BMEL, HSC-SP-Quiescent, GEP, SiEP	
MCE-MIR_725:fwd	AAUUUUAUGCCACCACUCCC	SiEP	
MCE-MIR_734:fwd	ACAAACUUCUGCAGCACCUGCUC	Misc, SiEP	
MCE-MIR_755:fwd	ACAAGCCGGGCAUAAUAUGCAGGGGC	SCDb, StroCDb	
MCE-MIR_774:rev	CUUGCUCCUGGCCUGUGUGU	SiEP	
MCE-MIR_777:rev	CAAAGGAUGGGGAUGGUGUGU	SCDb	
MCE-MIR_780:rev	GGAGGCUGGUGGCAGUGUGU	WBM	
MCE-MIR_782:rev	UGACAGAGCUGUGUGCUGUGU	SiEP	
MCE-MIR_809:fwd	ACACUUGACUGCUGGAGGAAGAUUGC	BMEL	
MCE-MIR_81:fwd	AAACAGAACUUCUCAUGGGCA	SCDb	
MCE-MIR_855:fwd	ACAGUAGAGGGAUGCAACUG	SCDb	

MCE-MIR_855:rev	CAGUUGCAUCCCUCUACUGU	SCDb	
MCE-MIR_871:fwd	ACAUAGGAGUCCUUCUGACCCAU	BMEL, WBM, Misc, SiEP	
MCE-MIR_871:rev	AUGGGUCAGAAGGACUCCUAUGU	BMEL, WBM, Misc, SiEP	
MCE-MIR_89:fwd	AAACAUGAGGUUGCUCAGGCAGAG	SCDb	
MCE-MIR_936:rev	CAGUGGAGCUUGGAGCUGGUGAUGGUGGU	SCDb	
MCE-MIR_942:rev	GAAGCCCACCAGGCAGCACAGGGACGUGGU	SCDb, StroCDb	
MCE-MIR_946:rev	AUCUGCACUGCCAAGACUGAGUGGU	SCDb	
MCE-MIR_959:rev	UUCAAUGUGAUCCGCCUGGU	GEP, SiEP	
MCE-MIR_988:fwd	ACCCAGGUCCUACUUCUCCC	SCDb, StroCDb	
MCE-MIR_993:rev	GUUUUGCUAAUUCUUUUCCGUGGGGU	SCDb	
