# Supplementary material for: Novel MicroRNA Candidates and miRNA-mRNA Pairs in Embryonic Stem (ES) Cells
Source: PLoS One. 2008 Jul 2;3(7):e2548. doi: 10.1371/journal.pone.0002548 (PMC2481296; doi:10.1371/journal.pone.0002548)
Supplement: Table S2 — Chromosome location of Mouse Array Version 2 MCE-MIR hairpins with unique hits on the genome. This data was derived using the mm7 (Aug 2005) assembly of the Mouse Genome. ST2A shows results from MCE-MIRs with unique hits to the genome and ST2B shows results from MCE-MIRs with multiple hits to the genome. (0.14 MB PDF) [file pone.0002548.s009.pdf]

Supplemental Table ST2A: Chromosome location and genome context of MCE-MIRs on Mouse Array Version 2. This data was derived using the mm7 (Aug 2005) assembly of the Mouse Genome.

MCE-MIR	Genomic Context	RefSeq Gene	Position (mm7)
MCE-MIR_1038:fwd	Exon anti	Zfp295	chr16: 98,334,763-98,334,955
MCE-MIR_1046:rev	Exon anti	Rbm18	chr2: 36,134,480-36,134,556
MCE-MIR_1052:rev	Intron/Exon Intersection parallel	AW547186	chrX: 137,470,295-137,470,398
MCE-MIR_1059:fwd	Exon anti	Hdlbp	chr1: 93,346,047-93,346,105
MCE-MIR_1066:rev	Intergenic		chr17: 78,152,776-78,152,995
MCE-MIR_1074:rev	Intergenic		chr19: 6,667,093-6,667,158
MCE-MIR_1190:fwd	UTR anti	Mark2	chr19: 7,096,454-7,096,693
MCE-MIR_1192:rev	Intron/Exon Intersection parallel	Sorbs3	chr14: 64,835,307-64,835,727
MCE-MIR_1226:fwd	Intergenic		chr11: 42,818,806-42,818,868
MCE-MIR_1259:rev	Exon parallel	Ifrg15	chr1: 156,013,866-156,013,991
MCE-MIR_1264:rev	Intron/Exon Intersection anti	Ctsd	chr7: 138,807,964-138,808,090
MCE-MIR_1269:rev	Intron/Exon Intersection anti	Prdm4	chr10: 85,451,616-85,451,691
MCE-MIR_1283:fwd	Intron/Exon Intersection parallel	Wdr1	chr5: 37,884,005-37,884,426
MCE-MIR_1283:rev	Intron/Exon Intersection anti	Wdr1	chr5: 37,884,143-37,884,228
MCE-MIR_1311:rev	Intron/Exon Intersection anti	Lmbr1l	chr15: 98,893,753-98,894,173
MCE-MIR_1325:rev	Intergenic		chr8: 103,954,328-103,954,547
MCE-MIR_1342:fwd	Intron/Exon Intersection parallel	Mkln1	chr6: 31,389,152-31,389,395
MCE-MIR_135:fwd	UTR-Intersection anti	Ppgb	chr2: 164,573,793-164,573,894
MCE-MIR_1352:fwd	Exon anti	Mapk6	chr9: 75,434,987-75,435,212
MCE-MIR_1356:fwd	UTR-Intersection anti	Xpo6	chr7: 122,198,788-122,198,884
MCE-MIR_1356:rev	UTR-Intersection parallel	Xpo6	chr7: 122,198,792-122,198,880
MCE-MIR_136:fwd	UTR-Intersection anti	Ppgb	chr2: 164,573,655-164,574,078
MCE-MIR_1364:fwd	Intergenic		chr4: 61,738,294-61,738,513
MCE-MIR_1365:fwd	UTR-Intersection anti	Ogfr	chr2: 181,096,413-181,096,632
MCE-MIR_1365:rev	UTR-Intersection parallel	Ogfr	chr2: 181,096,313-181,096,732
MCE-MIR_1371:fwd	Intron/Exon Intersection parallel	Cuedc2	chr19: 46,227,838-46,228,057
MCE-MIR_1401:fwd	Exon anti	Prdx1	chr4: 116,068,276-116,068,368
MCE-MIR_1412:fwd	Intergenic		chr9: 53,077,781-53,077,904
MCE-MIR_1433:fwd	Intron/Exon Intersection parallel	Akr7a5	chr4: 138,692,945-138,693,013
MCE-MIR_1433:rev	Intron/Exon Intersection anti	Akr7a5	chr4: 138,692,945-138,693,013
MCE-MIR_1442:fwd	Intron/Exon Intersection anti	Nr1i2	chr16: 37,957,907-37,958,000
MCE-MIR_1458:rev	Intron/Exon Intersection parallel	Nudt16l1	chr16: 4,612,813-4,612,942
MCE-MIR_1482:rev	Exon anti	Ddb2	chr2: 91,069,535-91,069,595
MCE-MIR_1495:fwd	Intron/Exon Intersection anti	Arnt	chr3: 95,358,811-95,359,031
MCE-MIR_1504:rev	Intergenic		chr7: 122,967,139-122,967,359

MCE-MIR	Genomic Context	RefSeq Gene	Position (mm7)
MCE-MIR_1506:rev	Intergenic		chr11: 4,433,375-4,433,430
MCE-MIR_1508:fwd	Intron parallel	Il6ra	chr3: 89,373,164-89,373,389
MCE-MIR_151:fwd	Intergenic		chr3: 88,474,104-88,474,164
MCE-MIR_1514:fwd	Intron/Exon Intersection parallel	Osbpl2	chr2: 180,659,850-180,660,069
MCE-MIR_1535:rev	Exon anti	Bc3324	chr5: 123,723,799-123,723,905
MCE-MIR_1536:rev	Intron/Exon Intersection parallel	Zfp513	chr5: 30,232,475-30,232,729
MCE-MIR_1538:fwd	UTR parallel	95358B2Rik	chr17: 24,046,866-24,047,090
MCE-MIR_1539:rev	Intron/Exon Intersection parallel	Fkbp2	chr19: 6,798,328-6,798,550
MCE-MIR_1544:rev	Intergenic		chr1: 105,930,148-105,930,370
MCE-MIR_1546:rev	Intron/Exon Intersection anti	573593F17Rik	chr11: 95,484,513-95,484,933
MCE-MIR_1569:rev	Intron/Exon Intersection anti	H2afy	chr13: 55,185,424-55,185,644
MCE-MIR_1576:rev	Intron parallel	St6galnac2	chr11: 116,558,576-116,558,795
MCE-MIR_1597:fwd	UTR-Intersection anti	Atp6vd1	chr8: 104,164,432-104,164,664
MCE-MIR_1611:fwd	Intron/Exon Intersection parallel	Lrrc47	chr4: 153,007,861-153,008,081
MCE-MIR_162:fwd	Intron/Exon Intersection parallel	Ndrg4	chr8: 94,297,419-94,297,838
MCE-MIR_1642:rev	Intron/Exon Intersection anti	Cd82	chr2: 93,272,530-93,272,614
MCE-MIR_1670:fwd	Intron/Exon Intersection anti	H2afy	chr13: 55,185,434-55,185,656
MCE-MIR_1679:fwd	Intergenic		chr3: 150,482,410-150,482,489
MCE-MIR_1689:rev	Intron anti	1719N19Rik	chr19: 58,712,476-58,712,584
MCE-MIR_1710:rev	Exon parallel	Ap2a1	chr7: 41,080,263-41,080,343
MCE-MIR_1734:fwd	Intergenic		chr9: 110,342,130-110,342,349
MCE-MIR_1742:rev	Intron/Exon Intersection anti	Copa	chr1: 172,089,529-172,089,750
MCE-MIR_1746:rev	Intron/Exon Intersection parallel	Ankrd15	chr19: 25,130,807-25,131,029
MCE-MIR_1756:rev	Exon parallel	Trim8	chr19: 46,399,416-46,399,635
MCE-MIR_1773:fwd	UTR-Intersection anti	Fbxw5	chr2: 25,509,187-25,509,407
MCE-MIR_1773:rev	UTR-Intersection parallel	Fbxw5	chr2: 25,509,187-25,509,407
MCE-MIR_1778:fwd	Exon anti	Centb1	chr11: 69,965,930-69,965,988
MCE-MIR_1784:fwd	Intergenic		chr16: 18,634,741-18,634,960
MCE-MIR_1786:fwd	Intron/Exon Intersection anti	Map3k7ip1	chr15: 80,242,074-80,242,139
MCE-MIR_1788:fwd	UTR anti	Hif1an	chr19: 44,467,961-44,468,180
MCE-MIR_1788:rev	UTR parallel	Hif1an	chr19: 44,467,961-44,468,180
MCE-MIR_1793:fwd	UTR-Intersection parallel	Crb3	chr17: 55,276,155-55,276,576
MCE-MIR_1811:rev	Intron parallel	Il6ra	chr3: 89,595,074-89,595,297
MCE-MIR_1829:rev	Intron/Exon Intersection parallel	Rutbc3	chr15: 81,093,215-81,093,434
MCE-MIR_1857:fwd	UTR parallel	Sec24c	chr14: 18,350,605-18,351,025
MCE-MIR_1929:fwd	Intron/Exon Intersection anti	Eprs	chr1: 184,910,578-184,910,706
MCE-MIR_1973:fwd	Intron/Exon Intersection anti	633569M22Rik	chr3: 107,439,578-107,439,797
MCE-MIR_1974:fwd	Intron/Exon Intersection parallel	AI31418	chr4: 58,913,963-58,914,182
MCE-MIR_1986:fwd	Intron/Exon Intersection parallel	Pex6	chr17: 44,853,004-44,853,227

MCE-MIR	Genomic Context	RefSeq Gene	Position (mm7)
MCE-MIR_2078:fwd	Intergenic		chr5: 86,754,165-86,754,185
MCE-MIR_2087:rev	Intergenic		chr1: 10,280,227-10,280,364
MCE-MIR_2134:fwd	Intron/Exon Intersection anti	Stno	chr10: 79,849,036-79,849,252
MCE-MIR_2139:fwd	Intron/Exon Intersection parallel	Epha2	chr4: 140,700,279-140,700,383
MCE-MIR_2164:rev	Intron/Exon Intersection parallel	Fgd5	chr6: 92,131,378-92,131,598
MCE-MIR_2166:fwd	Intron/Exon Intersection anti	Nfatc4	chr14: 50,695,802-50,696,221
MCE-MIR_2166:rev	Intron/Exon Intersection parallel	Nfatc4	chr14: 50,695,802-50,696,221
MCE-MIR_2169:rev	Intron/Exon Intersection anti	Cry1	chr10: 84,725,605-84,725,830
MCE-MIR_2171:rev	Intron/Exon Intersection parallel	Prg2	chr2: 84,826,293-84,826,526
MCE-MIR_2173:rev	NO MATCH		no match
MCE-MIR_2192:rev	Intron anti	Itsn2	chr12: 4,621,887-4,621,962
MCE-MIR_2196:fwd	Exon anti	Brunol4	chr18: 25,880,884-25,880,952
MCE-MIR_2205:fwd	Intron/Exon Intersection anti	Tulp2	chr7: 41,697,701-41,697,762
MCE-MIR_2222:fwd	Exon anti	Tgif	chr17: 69,124,699-69,124,786
MCE-MIR_2243:fwd	Intron/Exon Intersection anti	Gramd1a	chr7: 28,076,090-28,076,310
MCE-MIR_2285:fwd	Intron/Exon Intersection parallel	Calu	chr6: 29,304,667-29,304,887
MCE-MIR_2304:rev	Intron/Exon Intersection parallel	Guk1	chr11: 59,266,739-59,266,958
MCE-MIR_2310:rev	Exon anti	Gfra1	chr19: 58,216,430-58,216,513
MCE-MIR_2327:fwd	Intron/Exon Intersection parallel	Smarca2	chr19: 26,420,719-26,420,782
MCE-MIR_2337:fwd	Intron/Exon Intersection parallel	Gyk	chrX: 81,098,975-81,099,194
MCE-MIR_2345:fwd	Intergenic		chr7: 78,790,436-78,790,661
MCE-MIR_2349:rev	Intron/Exon Intersection anti	A9331D7Rik	chr1: 131,936,032-131,936,251
MCE-MIR_2361:fwd	Intron/Exon Intersection anti	Pknox2	chr9: 36,883,254-36,883,480
MCE-MIR_2371:fwd	Intron/Exon Intersection parallel	Ddx41	chr13: 54,620,795-54,621,214
MCE-MIR_2371:rev	Exon anti	Ddx41	chr13: 54,620,925-54,621,060
MCE-MIR_2388:rev	Intron/Exon Intersection anti	Prkd2	chr7: 14,629,518-14,629,750
MCE-MIR_2417:fwd	Intron/Exon Intersection parallel	Ezh2	chr6: 47,581,110-47,581,260
MCE-MIR_2419:fwd	Intron parallel	Spint1	chr2: 119,707,073-119,707,174
MCE-MIR_2464:fwd	Intron/Exon Intersection parallel	Nat1	chr2: 103,576,401-103,576,622
MCE-MIR_2470:fwd	Intron parallel	Rbm5	chr9: 107,488,672-107,488,907
MCE-MIR_2474:rev	Intron/Exon Intersection anti	Nckap1l	chr15: 103,475,443-103,475,864
MCE-MIR_2522:rev	UTR anti	Unc84a	chr5: 138,221,693-138,222,112
MCE-MIR_2524:fwd	Intergenic		chr19: 44,876,679-44,876,913
MCE-MIR_254:fwd	Exon anti	Dus31	chr17: 54,979,964-54,980,023
MCE-MIR_2563:fwd	NO MATCH		no match
MCE-MIR_2563:rev	Exon anti	A931N9Rik	chr17: 24,944,784-24,944,861
MCE-MIR_2566:fwd	Intron parallel	Igsf4a	chr9: 47,492,541-47,492,562
MCE-MIR_2566:rev	Intron anti	Igsf4a	chr9: 47,492,541-47,492,562
MCE-MIR_2617:fwd	Exon parallel	Zbtb38	chr9: 96,329,470-96,329,689

MCE-MIR	Genomic Context	RefSeq Gene	Position (mm7)
MCE-MIR_2624:rev	Intergenic		chr5: 72,697,247-72,697,267
MCE-MIR_2661:rev	Exon anti	Egr1	chr18: 35,239,402-35,239,479
MCE-MIR_2679:fwd	Exon anti	Coro1a	chr7: 122,798,585-122,798,641
MCE-MIR_2680:fwd	UTR-Intersection anti	C85492	chr9: 121,691,409-121,691,628
MCE-MIR_2680:rev	UTR-Intersection parallel	C85492	chr9: 121,691,409-121,691,628
MCE-MIR_2698:rev	Intergenic		chr9: 110,011,343-110,011,565
MCE-MIR_2714:rev	Intron/Exon Intersection parallel	Rfx1	chr8: 82,675,926-82,676,149
MCE-MIR_273:fwd	Intron/Exon Intersection parallel	Fchsd2	chr7: 97,323,651-97,323,882
MCE-MIR_2798:fwd	UTR anti	Strn3	chr12: 49,622,497-49,622,882
MCE-MIR_281:rev	Intron anti	Lilrb4	chr10: 51,310,982-51,311,068
MCE-MIR_2815:fwd	Intergenic		chr3: 94,299,755-94,299,974
MCE-MIR_2817:fwd	Intron/Exon Intersection anti	Cyfip2	chr11: 46,177,989-46,178,215
MCE-MIR_2866:rev	Exon anti	Hoxa2	chr6: 52,126,444-52,126,597
MCE-MIR_2894:fwd	UTR parallel	Itm2c	chr1: 85,809,251-85,809,319
MCE-MIR_2902:fwd	Intron/Exon Intersection parallel	Map4k4	chr1: 40,245,127-40,245,538
MCE-MIR_2902:rev	Intron/Exon Intersection anti	Map4k4	chr1: 40,245,218-40,245,438
MCE-MIR_291:rev	Intron/Exon Intersection anti	Idh2	chr7: 75,963,393-75,963,525
MCE-MIR_293:fwd	Exon parallel	Lzts2	chr19: 44,920,130-44,920,349
MCE-MIR_293:rev	Exon anti	Lzts2	chr19: 44,920,193-44,920,260
MCE-MIR_2968:fwd	Intron/Exon Intersection parallel	Eif3s8	chr7: 122,652,931-122,653,153
MCE-MIR_2977:rev	UTR parallel	Dab2ip	chr2: 35,737,118-35,737,137
MCE-MIR_298:rev	Intergenic		chr10: 97,042,003-97,042,234
MCE-MIR_2983:rev	UTR anti	Fbxo32	chr15: 58,246,194-58,246,276
MCE-MIR_2986:fwd	Intron/Exon Intersection anti	Brd4	chr17: 30,414,198-30,414,271
MCE-MIR_2999:fwd	UTR anti	Itpk1	chr12: 100,063,851-100,064,070
MCE-MIR_3007:rev	Intergenic		chr3: 123,346,630-123,346,706
MCE-MIR_3032:fwd	Intergenic		chr11: 87,826,761-87,826,845
MCE-MIR_3057:rev	Intergenic		chr1: 95,348,851-95,348,905
MCE-MIR_3059:rev	Exon anti	Cldn7	chr11: 70,047,559-70,047,611
MCE-MIR_3084:fwd	Intergenic		chrUn_random: 10,190,390-10,190,421
MCE-MIR_3113:fwd	Intron/Exon Intersection anti	Nup188	chr2: 30,329,176-30,329,321
MCE-MIR_3134:rev	Intron parallel	Slc44a2	chr9: 21,228,843-21,228,995
MCE-MIR_3147:fwd	Exon anti	Lmo2	chr2: 103,826,810-103,826,873
MCE-MIR_3155:fwd	Intergenic		chr11: 109,120,615-109,120,808
MCE-MIR_3190:rev	UTR-Intersection anti	Bc17c	chr7: 123,801,406-123,801,828
MCE-MIR_3191:rev	UTR-Intersection anti	Bc17c	chr7: 123,801,503-123,801,728
MCE-MIR_321:rev	UTR-Intersection anti	Fkbp8	chr8: 68,990,033-68,990,255
MCE-MIR_3226:fwd	Exon parallel	Tfg	chr16: 56,547,730-56,547,951
MCE-MIR_3260:rev	Exon parallel	Atp6vb	chr4: 117,261,627-117,261,687

MCE-MIR	Genomic Context	RefSeq Gene	Position (mm7)
MCE-MIR_329:fwd	Intron/Exon Intersection anti	Gmppb	chr9: 107,781,753-107,781,981
MCE-MIR_3295:rev	NO MATCH		no match
MCE-MIR_3330:fwd	Intergenic		chrX: 43,498,538-43,498,957
MCE-MIR_3333:rev	Intron/Exon Intersection anti	Abcf3	chr16: 20,099,254-20,099,303
MCE-MIR_3334:fwd	UTR parallel	Nfic	chr10: 81,191,786-81,192,005
MCE-MIR_334:rev	Intron parallel	Rpl32	chr6: 115,833,655-115,833,714
MCE-MIR_335:fwd	Exon anti	Gpx4	chr10: 45,687,261-45,687,480
MCE-MIR_3379:fwd	Exon anti	Scrn1	chr6: 54,497,823-54,497,902
MCE-MIR_3407:rev	Intron/Exon Intersection parallel	Tubgcp2	chr7: 136,420,698-136,420,920
MCE-MIR_3416:rev	Intergenic		chr19: 5,962,674-5,962,721
MCE-MIR_3429:fwd	UTR-Intersection parallel	Obfc2b	chr10: 127,903,112-127,903,534
MCE-MIR_3429:rev	UTR-Intersection anti	Obfc2b	chr10: 127,903,212-127,903,434
MCE-MIR_3441:fwd	UTR anti	Ralbp1	chr17: 64,066,842-64,066,928
MCE-MIR_3470:rev	Intron/Exon Intersection parallel	1318I5Rik	chr17: 27,894,532-27,894,588
MCE-MIR_3471:rev	Intron/Exon Intersection parallel	Ankrd13a	chr5: 114,012,065-114,012,265
MCE-MIR_3474:rev	Exon anti	AI428795	chr5: 110,451,758-110,451,978
MCE-MIR_3477:rev	Intergenic		chrX: 69,611,639-69,611,877
MCE-MIR_3478:rev	Intergenic		chr14: 88,233,477-88,233,505
MCE-MIR_3484:rev	Intron/Exon Intersection parallel	Tuba4	chr1: 75,439,274-75,439,493
MCE-MIR_3485:rev	Intron/Exon Intersection anti	Map2k2	chr10: 80,911,579-80,911,798
MCE-MIR_3488:rev	Intron/Exon Intersection parallel	Fxr1h	chr3: 33,866,852-33,867,074
MCE-MIR_3490:rev	Intergenic		chr18: 55,276,939-55,277,007
MCE-MIR_3491:rev	Intron/Exon Intersection anti	Gipc1	chr8: 82,240,691-82,240,910
MCE-MIR_3492:fwd	UTR-Intersection anti	Ddah2	chr17: 33,157,188-33,157,407
MCE-MIR_3502:rev	Intron anti	Eppk1	chr15: 76,253,550-76,253,770
MCE-MIR_3503:rev	UTR-Intersection anti	Gamt	chr10: 80,040,506-80,040,728
MCE-MIR_3513:rev	Intergenic		chr17: 38,066,118-38,066,342
MCE-MIR_3522:rev	Exon parallel	Araf	chrX: 19,290,802-19,290,893
MCE-MIR_3529:rev	UTR anti	Gpc1	chr1: 92,766,642-92,766,688
MCE-MIR_3531:rev	Intron/Exon Intersection anti	Psmd8	chr7: 26,118,081-26,118,306
MCE-MIR_3541:rev	Exon parallel	Wnt5a	chr14: 25,952,235-25,952,454
MCE-MIR_3543:rev	Intron anti	Nnp1	chr10: 78,057,115-78,057,190
MCE-MIR_3550:fwd	Intron/Exon Intersection anti	Ankrd23	chr1: 36,773,703-36,773,784
MCE-MIR_3557:rev	Intron/Exon Intersection anti	Ubqln4	chr3: 88,676,821-88,677,051
MCE-MIR_3572:rev	UTR-Intersection anti	2318M1Rik	chr3: 130,545,674-130,545,899
MCE-MIR_3573:rev	Intron/Exon Intersection parallel	Clta	chr4: 44,026,653-44,026,878
MCE-MIR_3613:rev	Intron/Exon Intersection parallel	Plod3	chr5: 135,964,115-135,964,210
MCE-MIR_3619:rev	Exon anti	Anp32a	chr9: 62,398,458-62,398,580
MCE-MIR_3626:rev	Intron/Exon Intersection parallel	Eif2b2	chr12: 83,219,575-83,219,797

MCE-MIR	Genomic Context	RefSeq Gene	Position (mm7)
MCE-MIR_3637:rev	Intergenic		chr3: 93,537,960-93,538,010
MCE-MIR_364:fwd	Intergenic		chr2: 143,697,629-143,697,781
MCE-MIR_3642:rev	UTR-Intersection parallel	Abhd14a	chr9: 106,169,474-106,169,693
MCE-MIR_3646:rev	Intergenic		chr1: 118,369,374-118,369,593
MCE-MIR_3651:fwd	NO MATCH		no match
MCE-MIR_3653:rev	UTR-Intersection anti	Ehhadh	chr16: 21,411,517-21,411,736
MCE-MIR_3663:fwd	Intron/Exon Intersection anti	AL33326	chr2: 152,063,928-152,064,169
MCE-MIR_3667:fwd	Intron/Exon Intersection anti	AI4516	chr15: 89,435,149-89,435,370
MCE-MIR_3667:rev	Intron/Exon Intersection parallel	AI4516	chr15: 89,435,233-89,435,314
MCE-MIR_3684:rev	Intron anti	BC12278	chrUn_random: 1,269,207-1,269,321
MCE-MIR_3685:fwd	Intron/Exon Intersection anti	Tomm4l	chr1: 171,190,335-171,190,560
MCE-MIR_3685:rev	Intron/Exon Intersection parallel	Tomm41	chr1: 171,190,335-171,190,560
MCE-MIR_3686:fwd	Exon parallel	Slc25a4	chr8: 44,987,060-44,987,160
MCE-MIR_3715:rev	NO MATCH		no match
MCE-MIR_3751:fwd	UTR-Intersection anti	Mea1	chr17: 44,811,345-44,811,574
MCE-MIR_3762:rev	Intergenic		chr7: 39,237,162-39,237,184
MCE-MIR_3780:rev	Exon parallel	BC3324	chr5: 123,723,724-123,723,764
MCE-MIR_3782:rev	Intron parallel	Pla2g4e	chr2: 119,979,673-119,979,736
MCE-MIR_3791:rev	Intron/Exon Intersection anti	Gnb2l1	chr11: 48,758,468-48,758,688
MCE-MIR_3793:rev	UTR-Intersection anti	Cltb	chr13: 53,678,056-53,678,287
MCE-MIR_3797:fwd	UTR-Intersection parallel	Ccnl1	chr3: 66,047,602-66,047,821
MCE-MIR_3832:rev	Intergenic		chr7: 78,790,538-78,790,589
MCE-MIR_3837:rev	UTR-Intersection anti	Cnih	chr14: 42,108,245-42,108,464
MCE-MIR_3841:fwd	Exon parallel	Gripap1	chrX: 6,251,208-6,251,266
MCE-MIR_3859:fwd	Intergenic		chr17: 38,064,906-38,065,126
MCE-MIR_3958:fwd	Exon parallel	Rpp25	chr9: 57,532,052-57,532,272
MCE-MIR_4010:rev	Exon parallel	Fyn	chr10: 39,343,575-39,343,640
MCE-MIR_4015:fwd	UTR-Intersection parallel	Nfrkb	chr9: 31,322,364-31,322,584
MCE-MIR_4022:fwd	Intron/Exon Intersection anti	Smtn	chr11: 3,484,393-3,484,612
MCE-MIR_4027:rev	Intergenic		chrX: 69,610,130-69,610,352
MCE-MIR_4030:rev	UTR-Intersection anti	C3311M18Rik	chr8: 82,648,560-82,648,780
MCE-MIR_4034:rev	Intron/Exon Intersection parallel	Aplp2	chr9: 31,057,219-31,057,438
MCE-MIR_405:rev	Intergenic		chr18: 35,193,427-35,193,846
MCE-MIR_4060:rev	UTR anti	Kpnb1	chr11: 97,270,707-97,270,826
MCE-MIR_4063:fwd	Intron/Exon Intersection parallel	C4a	chr17: 32,816,535-32,816,654
MCE-MIR_4069:rev	Intron/Exon Intersection anti	Arpc4	chr6: 113,541,608-113,541,827
MCE-MIR_407:fwd	Intergenic		chr2: 118,489,241-118,489,660
MCE-MIR_4087:fwd	Intron/Exon Intersection parallel	Sh3bp1	chr15: 78,988,626-78,988,851
MCE-MIR_4087:rev	Intron/Exon Intersection anti	Sh3bp1	chr15: 78,988,626-78,988,851

MCE-MIR	Genomic Context	RefSeq Gene	Position (mm7)
MCE-MIR_4153:fwd	Intron/Exon Intersection anti	Usp2	chr2: 31,018,670-31,018,889
MCE-MIR_4179:fwd	UTR parallel	Srf	chr17: 44,676,200-44,676,357
MCE-MIR_4179:rev	UTR anti	Srf	chr17: 44,676,146-44,676,366
MCE-MIR_4198:fwd	UTR parallel	241166I5Rik	chr4: 133,904,205-133,904,424
MCE-MIR_4207:fwd	Exon anti	Trim8	chr19: 46,411,285-46,411,349
MCE-MIR_4209:fwd	Intron/Exon Intersection parallel	Inpp5b	chr4: 124,242,972-124,243,042
MCE-MIR_4226:fwd	Intron anti	Csnk1e	chr15: 79,506,910-79,506,977
MCE-MIR_4232:fwd	Exon anti	Gnas	chr2: 174,090,686-174,090,757
MCE-MIR_4236:rev	Intron/Exon Intersection parallel	Egfl7	chr2: 26,598,188-26,598,286
MCE-MIR_4239:rev	Intergenic		chr17: 26,502,036-26,502,222
MCE-MIR_4273:fwd	Intergenic		chr6: 52,169,325-52,169,400
MCE-MIR_4274:fwd	Intron parallel	Il6ra	chr3: 89,305,432-89,305,558
MCE-MIR_4280:fwd	Intron/Exon Intersection anti	Scamp4	chr10: 80,397,970-80,398,389
MCE-MIR_4280:rev	Exon parallel	Scamp4	chr10: 80,398,148-80,398,252
MCE-MIR_4303:fwd	Exon anti	Tubb2c	chr2: 25,229,739-25,229,817
MCE-MIR_4320:rev	UTR anti	28127O19Rik	chr2: 109,868,756-109,868,977
MCE-MIR_4342:fwd	Intergenic		chr2: 92,411,975-92,412,358
MCE-MIR_4345:rev	UTR-Intersection parallel	D1Ertd641e	chr10: 59,514,638-59,514,860
MCE-MIR_4353:rev	Intron/Exon Intersection parallel	Rorc	chr3: 94,263,326-94,263,745
MCE-MIR_4383:rev	UTR-Intersection anti	543432M24Rik	chr2: 151,711,010-151,711,429
MCE-MIR_4413:fwd	Intron anti	Lnk	chr5: 121,063,661-121,063,756
MCE-MIR_4442:fwd	Intron/Exon Intersection anti	4932417H2Rik	chr11: 120,005,560-120,005,667
MCE-MIR_4449:rev	Exon anti	Itm2a	chrX: 102,725,482-102,725,552
MCE-MIR_4462:fwd	UTR anti	Crsp2	chrX: 11,113,529-11,113,562
MCE-MIR_4474:fwd	Exon parallel	Eif4b	chr15: 102,079,151-102,079,231
MCE-MIR_4491:fwd	UTR parallel	Cd24a	chr10: 43,397,293-43,397,409
MCE-MIR_4491:rev	UTR anti	Cd24a	chr10: 43,397,291-43,397,519
MCE-MIR_4493:fwd	Intron/Exon Intersection parallel	Gnb2l1	chr11: 48,758,335-48,758,555
MCE-MIR_4497:rev	Intron/Exon Intersection anti	Edem1	chr6: 108,942,742-108,942,868
MCE-MIR_451:rev	Exon parallel	Gstk1	chr6: 42,189,043-42,189,071
MCE-MIR_4521:fwd	UTR anti	Srf	chr17: 44,680,420-44,680,526
MCE-MIR_4521:rev	Intron/Exon Intersection parallel	Srf	chr17: 44,680,304-44,680,671
MCE-MIR_4554:rev	NO MATCH		no match
MCE-MIR_4592:fwd	Intron/Exon Intersection parallel	Ndufab1	chr7: 118,017,891-118,018,006
MCE-MIR_4607:fwd	Exon anti	Usp28	chr9: 49,013,542-49,013,616
MCE-MIR_4610:fwd	Intron/Exon Intersection parallel	Gpbp1l1	chr4: 115,950,717-115,950,939
MCE-MIR_4614:rev	Exon parallel	Actr3b	chr5: 24,870,929-24,870,983
MCE-MIR_4627:fwd	Exon parallel	BC17647	chr11: 78,236,545-78,236,768
MCE-MIR_466:fwd	Exon anti	Sin3b	chr8: 71,246,642-71,246,701

MCE-MIR	Genomic Context	RefSeq Gene	Position (mm7)
MCE-MIR_4661:fwd	Intron/Exon Intersection parallel	Cecr5	chr6: 120,535,852-120,536,078
MCE-MIR_4661:rev	Intron/Exon Intersection anti	Cecr5	chr6: 120,535,852-120,536,078
MCE-MIR_4667:fwd	Intergenic		chr14: 115,984,936-115,985,007
MCE-MIR_4674:fwd	UTR-Intersection parallel	Fus	chr7: 124,078,253-124,078,481
MCE-MIR_4675:fwd	Intergenic		chr5: 28,943,707-28,943,726
MCE-MIR_4684:fwd	Intron/Exon Intersection anti	Xpo6	chr7: 122,201,405-122,201,624
MCE-MIR_469:fwd	Intron/Exon Intersection parallel	BC61259	chr7: 123,866,624-123,866,846
MCE-MIR_4712:fwd	Intron/Exon Intersection parallel	Tbc1d1b	chr7: 123,295,570-123,295,789
MCE-MIR_4712:rev	Exon anti	Tbc1d1b	chr7: 123,295,631-123,295,677
MCE-MIR_4716:fwd	Intron/Exon Intersection anti	Aff1	chr5: 103,038,578-103,038,803
MCE-MIR_4726:fwd	Intergenic		chr8: 28,038,023-28,038,125
MCE-MIR_4726:rev	NO MATCH		no match
MCE-MIR_4738:fwd	Intergenic		chr11: 6,550,193-6,550,412
MCE-MIR_4743:fwd	Intergenic		chr15: 27,844,065-27,844,147
MCE-MIR_4745:fwd	Intron/Exon Intersection anti	Chmp2a	chr7: 11,618,686-11,619,106
MCE-MIR_4748:rev	Exon anti	Akap8	chr17: 30,500,215-30,500,312
MCE-MIR_4752:rev	UTR-Intersection parallel	26134G8Rik	chr2: 157,815,822-157,816,041
MCE-MIR_4755:fwd	UTR parallel	Ihpk1	chr9: 107,778,181-107,778,253
MCE-MIR_4756:rev	Intron/Exon Intersection anti	Ctnnb1	chr9: 120,649,681-120,649,903
MCE-MIR_4762:fwd	Exon parallel	Acta1	chr8: 122,480,510-122,480,611
MCE-MIR_4789:rev	Intron/Exon Intersection anti	Zfp46	chr15: 68,193,550-68,193,646
MCE-MIR_4791:fwd	Intergenic		chr7: 137,870,563-137,870,782
MCE-MIR_4791:rev	Intergenic		chr7: 137,870,563-137,870,782
MCE-MIR_4799:rev	Intron parallel	Dnm3	chr1: 162,184,411-162,184,856
MCE-MIR_4821:rev	Intron/Exon Intersection anti	Mkln1	chr6: 31,426,890-31,427,109
MCE-MIR_4822:rev	Intergenic		chr8: 3,716,808-3,717,001
MCE-MIR_4832:rev	UTR anti	Fmr1	chrX: 64,111,844-64,112,274
MCE-MIR_4853:fwd	Exon anti	Cnot8	chr11: 58,199,001-58,199,119
MCE-MIR_4854:rev	Exon parallel	Ccdc14	chr11: 29,199,038-29,199,116
MCE-MIR_4861:fwd	Intron/Exon Intersection parallel	Stno	chr10: 79,849,545-79,849,764
MCE-MIR_4893:fwd	Intron/Exon Intersection anti	Adam11	chr11: 102,881,474-102,881,896
MCE-MIR_4893:rev	Intron/Exon Intersection parallel	Adam11	chr11: 102,881,474-102,881,896
MCE-MIR_4913:rev	UTR-Intersection parallel	Dedd2	chr7: 22,007,843-22,008,066
MCE-MIR_4922:fwd	UTR-Intersection parallel	Ywhae	chr11: 75,814,475-75,814,700
MCE-MIR_4922:rev	UTR-Intersection anti	Ywhae	chr11: 75,814,475-75,814,700
MCE-MIR_4932:rev	Intergenic		chr10: 116,937,662-116,937,761
MCE-MIR_4945:fwd	Intron/Exon Intersection anti	Hps1	chr19: 42,656,204-42,656,428
MCE-MIR_4972:fwd	UTR parallel	Fos	chr12: 83,477,990-83,478,078
MCE-MIR_4999:rev	Intron/Exon Intersection anti	Mapk9	chr11: 49,837,875-49,838,108

MCE-MIR	Genomic Context	RefSeq Gene	Position (mm7)
MCE-MIR_5004:rev	Intron/Exon Intersection parallel	Trim8	chr19: 46,399,437-46,399,662
MCE-MIR_5005:rev	Intron/Exon Intersection parallel	Katnb1	chr8: 93,684,399-93,684,618
MCE-MIR_5008:rev	Exon anti	Irx2	chr13: 69,562,567-69,562,652
MCE-MIR_5014:fwd	Intron/Exon Intersection parallel	Ncam1	chr9: 49,521,328-49,521,550
MCE-MIR_5033:rev	Intron anti	Usp4	chr9: 108,092,003-108,092,125
MCE-MIR_5046:rev	Intron/Exon Intersection parallel	Map2k6	chr11: 110,605,287-110,605,706
MCE-MIR_5056:rev	UTR-Intersection parallel	Fes	chr7: 76,247,093-76,247,318
MCE-MIR_5057:fwd	UTR-Intersection parallel	Rps6ka1	chr4: 133,226,401-133,226,626
MCE-MIR_5060:rev	Intergenic		chr9: 65,722,136-65,722,351
MCE-MIR_5062:rev	Intergenic		chr7: 116,520,028-116,520,353
MCE-MIR_5079:rev	Intron/Exon Intersection anti	Gtpbp2	chr17: 44,294,983-44,295,068
MCE-MIR_5083:rev	UTR anti	Impad1	chr4: 4,691,742-4,691,961
MCE-MIR_5088:rev	Intron/Exon Intersection anti	Wdfy3	chr5: 101,032,376-101,032,598
MCE-MIR_5089:fwd	UTR-Intersection parallel	Ndufa8	chr2: 36,056,431-36,056,651
MCE-MIR_5100:rev	UTR-Intersection anti	Tmem39b	chr4: 129,141,306-129,141,525
MCE-MIR_5105:rev	UTR anti	Ebf3	chr7: 133,599,109-133,599,232
MCE-MIR_5109:rev	Intron/Exon Intersection anti	Glud1	chr14: 31,763,734-31,763,954
MCE-MIR_5122:rev	Exon parallel	Hnrpr	chr4: 135,705,709-135,705,807
MCE-MIR_5135:fwd	UTR-Intersection parallel	Dhrs3	chr4: 144,305,835-144,306,055
MCE-MIR_5143:rev	Exon parallel	2315P5Rik	chr9: 25,365,859-25,365,929
MCE-MIR_5152:fwd	Intron/Exon Intersection anti	Psmb1	chr8: 104,536,116-104,536,337
MCE-MIR_5152:rev	Intron/Exon Intersection parallel	Psmb1	chr8: 104,536,116-104,536,337
MCE-MIR_5167:fwd	Exon parallel	Lyar	chr5: 37,315,431-37,315,485
MCE-MIR_5167:rev	Exon anti	Lyar	chr5: 37,315,424-37,315,492
MCE-MIR_5172:fwd	Exon anti	Fos	chr12: 83,476,418-83,476,649
MCE-MIR_5180:fwd	Intron/Exon Intersection parallel	Araf	chrX: 19,287,022-19,287,447
MCE-MIR_5180:rev	Intron/Exon Intersection anti	Araf	chrX: 19,287,022-19,287,447
MCE-MIR_5192:rev	Intergenic		chr2: 77,135,649-77,135,877
MCE-MIR_5193:rev	UTR parallel	Maml1	chr11: 50,212,766-50,212,985
MCE-MIR_5195:rev	Intron/Exon Intersection parallel	Rad18	chr6: 112,847,657-112,847,753
MCE-MIR_5197:rev	UTR-Intersection parallel	Skiv2l	chr17: 32,935,478-32,935,700
MCE-MIR_5210:fwd	Exon parallel	Csda	chr6: 131,443,672-131,443,706
MCE-MIR_5216:rev	Intron/Exon Intersection parallel	E2322H4Rik	chr18: 32,166,769-32,166,939
MCE-MIR_5276:fwd	UTR anti	Arfgap1	chr2: 181,482,355-181,482,574
MCE-MIR_5279:fwd	Intron/Exon Intersection anti	Col1a2	chr6: 4,490,312-4,490,390
MCE-MIR_5287:rev	Intron parallel	Prnpip1	chr4: 116,987,987-116,988,076
MCE-MIR_5291:fwd	Intergenic		chr8: 103,954,361-103,954,590
MCE-MIR_5295:rev	Exon parallel	Tiparp	chr3: 65,654,368-65,654,587
MCE-MIR_530:fwd	UTR parallel	Hdac11	chr6: 91,256,422-91,256,642

MCE-MIR	Genomic Context	RefSeq Gene	Position (mm7)
MCE-MIR_5300:rev	Intergenic		chr7_random: 1,156,234-1,156,314
MCE-MIR_5303:fwd	Intron/Exon Intersection parallel	BC68171	chrX: 70,487,617-70,487,832
MCE-MIR_5328:rev	Exon anti	Fyco1	chr9: 123,541,126-123,541,195
MCE-MIR_5339:rev	Intron/Exon Intersection parallel	Pdcd11	chr19: 47,030,228-47,030,365
MCE-MIR_534:fwd	UTR anti	C4314K11Rik	chr15: 98,817,120-98,817,342
MCE-MIR_5354:rev	Intergenic		chrX: 99,253,930-99,253,953
MCE-MIR_5363:fwd	Intron/Exon Intersection parallel	Hdgfrp2	chr17: 54,259,158-54,259,378
MCE-MIR_5363:rev	Intron/Exon Intersection anti	Hdgfrp2	chr17: 54,259,158-54,259,378
MCE-MIR_5369:rev	Exon parallel	Wdr68	chr11: 106,146,220-106,146,294
MCE-MIR_5374:fwd	Exon parallel	Rdh14	chr12: 10,426,699-10,426,918
MCE-MIR_5374:rev	UTR-Intersection anti	Rdh14	chr12: 10,426,599-10,427,018
MCE-MIR_5384:rev	Intron/Exon Intersection anti	A6325C2Rik	chr19: 41,452,049-41,452,282
MCE-MIR_5396:rev	UTR parallel	Ywhae	chr11: 75,846,917-75,847,162
MCE-MIR_5403:fwd	Intron/Exon Intersection anti	Pld4	chr12: 110,288,853-110,289,073
MCE-MIR_5411:rev	Intron/Exon Intersection anti	Adck1	chr12_random: 183,789-184,014
MCE-MIR_5418:fwd	UTR-Intersection anti	Eif5a	chr11: 70,000,734-70,000,977
MCE-MIR_543:fwd	Exon anti	Hspa2	chr12: 74,364,111-74,364,150
MCE-MIR_5440:fwd	UTR parallel	Cenpb	chr2: 130,918,130-130,918,350
MCE-MIR_5443:fwd	UTR-Intersection parallel	Sepn1	chr4: 133,919,248-133,919,467
MCE-MIR_5470:fwd	UTR-Intersection parallel	Junb	chr8: 83,563,410-83,563,633
MCE-MIR_5473:fwd	Intergenic		chr2: 54,357,231-54,357,450
MCE-MIR_5473:rev	Intergenic		chr2: 54,357,231-54,357,450
MCE-MIR_5488:rev	Intron/Exon Intersection parallel	Phf12	chr11: 78,099,630-78,099,858
MCE-MIR_5504:rev	UTR-Intersection parallel	Sacm1l	chr9: 123,307,116-123,307,272
MCE-MIR_5511:rev	Intron/Exon Intersection anti	Cxxc1	chr18: 74,615,132-74,615,343
MCE-MIR_5544:rev	UTR anti	Slc17a7	chr7: 41,351,716-41,351,936
MCE-MIR_557:fwd	Intron/Exon Intersection parallel	Sepx1	chr17: 22,921,779-22,921,998
MCE-MIR_5596:rev	Exon parallel	Pex7	chr10: 19,611,802-19,611,900
MCE-MIR_5597:fwd	Intergenic		chr10: 79,817,201-79,817,420
MCE-MIR_5606:fwd	UTR-Intersection anti	Nfe2	chr15: 103,244,211-103,244,430
MCE-MIR_5607:rev	Intron/Exon Intersection anti	Aplp2	chr9: 31,057,220-31,057,439
MCE-MIR_5620:fwd	Intron parallel	Eif5	chr12: 109,057,046-109,057,275
MCE-MIR_5623:rev	Intron/Exon Intersection parallel	Vav1	chr17: 55,514,003-55,514,088
MCE-MIR_5641:rev	Intron/Exon Intersection anti	Dennd2a	chr6: 39,415,498-39,415,719
MCE-MIR_5699:fwd	Intron parallel	Sat1	chrX: 150,016,165-150,016,398
MCE-MIR_5704:rev	Exon parallel	Saps3	chr19: 3,421,639-3,421,718
MCE-MIR_5712:rev	Intron anti	Arid4b	chr13: 13,436,064-13,436,253
MCE-MIR_5745:fwd	Intron/Exon Intersection parallel	Jub	chr14: 49,437,427-49,437,513
MCE-MIR_5790:fwd	Intron/Exon Intersection anti	Ebf4	chr2: 130,047,219-130,047,450

MCE-MIR	Genomic Context	RefSeq Gene	Position (mm7)
MCE-MIR_5864:fwd	Intron/Exon Intersection anti	Dld	chr12: 29,175,016-29,175,441
MCE-MIR_5872:fwd	Intron/Exon Intersection parallel	Nop17	chr7: 41,334,243-41,334,473
MCE-MIR_6001:rev	Exon anti	Ube2e1	chr14: 16,151,078-16,151,151
MCE-MIR_6034:rev	Intergenic		chrX: 105,950,353-105,950,376
MCE-MIR_6050:rev	Intron/Exon Intersection anti	Vim	chr2: 13,518,528-13,518,737
MCE-MIR_6054:rev	Intron anti	Txn14	chr18: 80,497,696-80,498,121
MCE-MIR_6055:rev	Exon anti	Zdhhc16	chr19: 41,831,537-41,831,616
MCE-MIR_6084:rev	UTR-Intersection anti	Prpf39	chr12: 62,994,730-62,994,891
MCE-MIR_6107:fwd	Intron/Exon Intersection parallel	Tpm2	chr4: 43,532,404-43,532,629
MCE-MIR_6120:fwd	UTR parallel	Cd24a	chr10: 43,397,308-43,397,593
MCE-MIR_638:fwd	Intron/Exon Intersection anti	Hadhsc	chr3: 131,081,600-131,081,825
MCE-MIR_645:fwd	Intron/Exon Intersection parallel	Mfsd3	chr15: 76,785,008-76,785,227
MCE-MIR_670:fwd	Intron/Exon Intersection anti	Cog1	chr11: 113,762,900-113,763,119
MCE-MIR_725:fwd	UTR parallel	Pogz	chr3: 94,754,283-94,754,502
MCE-MIR_734:fwd	Intron/Exon Intersection anti	Nsmce1	chr7: 121,567,593-121,567,815
MCE-MIR_755:fwd	Intron/Exon Intersection anti	Eif2c4	chr4: 125,941,357-125,941,566
MCE-MIR_774:rev	UTR parallel	Llgl1	chr11: 60,795,425-60,795,552
MCE-MIR_780:rev	Intron/Exon Intersection anti	Soat1	chr1: 156,403,755-156,403,974
MCE-MIR_783:rev	Intergenic		chr14: 18,378,732-18,378,788
MCE-MIR_809:fwd	Intron/Exon Intersection parallel	Slc25a4	chr8: 44,985,371-44,985,519
MCE-MIR_81:fwd	Intron/Exon Intersection anti	Zc3hc1	chr6: 30,313,906-30,313,986
MCE-MIR_822:fwd	UTR anti	Ubn1	chr16: 4,758,341-4,758,560
MCE-MIR_855:fwd	Intron/Exon Intersection anti	Dhx15	chr5: 51,525,012-51,525,371
MCE-MIR_855:rev	Intron/Exon Intersection parallel	Dhx15	chr5: 51,525,012-51,525,371
MCE-MIR_871:fwd	Intron/Exon Intersection anti	Actb	chr5: 141,938,244-141,938,466
MCE-MIR_871:rev	Exon parallel	Actb	chr5: 141,938,288-141,938,380
MCE-MIR_89:fwd	Intergenic		chr4: 43,699,836-43,699,917
MCE-MIR_936:rev	Exon parallel	Ilf3	chr9: 21,265,915-21,265,966
MCE-MIR_942:rev	Intron/Exon Intersection anti	D4Ertd429e	chr4: 148,716,725-148,716,954
MCE-MIR_946:rev	Intergenic		chr7: 116,937,558-116,937,577
MCE-MIR_959:rev	Intron/Exon Intersection parallel	Hadh2	chrX: 146,762,246-146,762,465
MCE-MIR_968:fwd	Intron/Exon Intersection parallel	Pgm1	chr5: 63,470,161-63,470,380
MCE-MIR_984:rev	Intron/Exon Intersection anti	Dhx16	chr17: 33,987,922-33,988,141
MCE-MIR_988:fwd	Intergenic		chr2: 182,083,828-182,084,047
MCE-MIR_993:rev	Exon anti	Gmps	chr3: 64,138,968-64,139,056
MCE-MIR_995:rev	Intron/Exon Intersection anti	Mark2	chr19: 7,100,827-7,101,047

Supplemental Table ST2B: Chromosome location of Mouse Array Version 2 MCE-MIR hairpins with multiple hits on the genome. This data was derived using the mm7 (Aug 2005) assembly of the Mouse Genome.

MCE-MIR	Genomic Context	RefSeq Gene	Position (mm7)
MCE-MIR-1015:rev	UTR-Intersection parallel	23116E2Rik	chr5: 29,939,148-29,939,384
MCE-MIR-1015:rev	Intergenic		chr18: 39,090,636-39,090,872
MCE-MIR-1394:rev	Intron anti	Jarid2	chr13: 44,319,255-44,319,481
MCE-MIR-1394:rev	Intron parallel	261528E23Rik	chr16: 57,252,906-57,253,132
MCE-MIR-1394:rev	Intron parallel	Kcnip1	chr11: 33,706,775-33,707,001
MCE-MIR-1394:rev	Exon	Myg1	chr17: 33,949,390-33,949,616
MCE-MIR-1394:rev	Intergenic		chr18: 69,084,373-69,084,599
MCE-MIR-1394:rev	Intergenic		chr15: 86,277,397-86,277,623
MCE-MIR-1394:rev	Intergenic		chr13: 104,251,075-104,251,301
MCE-MIR-1394:rev	Intergenic		chr14: 99,145,485-99,145,711
MCE-MIR-1394:rev	Intergenic		chr8: 15,493,984-15,494,210
MCE-MIR-1394:rev	Intergenic		chr6: 146,023,152-146,023,378
MCE-MIR-1394:rev	Intergenic		chr6: 109,660,943-109,661,169
MCE-MIR-1394:rev	Intergenic		chr6: 44,834,601-44,834,827
MCE-MIR-1394:rev	Intergenic		chr2: 20,965,116-20,965,342
MCE-MIR-1408:fwd	UTR-Intersection anti	Lysmd1	chr3: 95,012,483-95,012,702
MCE-MIR-1408:fwd	Intergenic		chr12: 91,410,978-91,411,197
MCE-MIR-1409:fwd	Exon parallel	Gvin1	chr7: 102,055,471-102,055,692
MCE-MIR-1409:fwd	Intron parallel	Gvin1	chr7: 102,080,229-102,080,450
MCE-MIR-1409:fwd	Intergenic		chr7: 102,494,345-102,494,566
MCE-MIR-1409:fwd	Intergenic		chr7: 102,401,981-102,402,202
MCE-MIR-1409:fwd	Intergenic		chr7: 102,220,998-102,221,219
MCE-MIR-1409:fwd	Intergenic		chr7: 101,974,244-101,974,465
MCE-MIR-1457:rev	Intron/Exon Intersection parallel	Fkbp2	chr19: 6,798,328-6,798,550
MCE-MIR-1457:rev	Intron/Exon Intersection parallel	Fkbp2	chr19: 6,798,325-6,798,550
MCE-MIR-1478:fwd	Intron/Exon Intersection anti	Vps11	chr9: 44,283,270-44,283,489
MCE-MIR-1478:fwd	Intron/Exon Intersection parallel	Dazap2	chr15: 100,603,243-100,603,462
MCE-MIR-1478:fwd	Intergenic		chr13: 12,221,785-12,222,004
MCE-MIR-1645:rev	Intron parallel	Terf2	chr8: 105,684,856-105,684,964
MCE-MIR-1645:rev	Intergenic		chr17: 24,728,001-24,728,083
MCE-MIR-1645:rev	Intergenic		chr1: 152,910,682-152,910,773
MCE-MIR-1645:rev	Intergenic		chr3: 95,284,504-95,284,612
MCE-MIR-1645:rev	Intergenic		chr8: 60,956,379-60,956,487
MCE-MIR-1645:rev	Intergenic		chr4: 126,110,452-126,110,560

MCE-MIR	Genomic Context	RefSeq Gene	Position (mm7)
MCE-MIR-1645:rev	Intergenic		chr12: 42,078,481-42,078,613
MCE-MIR-1645:rev	Intergenic		chr9: 115,102,731-115,102,839
MCE-MIR-1697:fwd	Intergenic		chr11: 77,721,537-77,721,625
MCE-MIR-1697:fwd	Intergenic		chr13_random: 11,345,637-11,345,705
MCE-MIR-1792:rev	Intron anti	Nup93	chr8: 92,812,348-92,812,412
MCE-MIR-1792:rev	Intergenic		chr15: 52,147,002-52,147,066
MCE-MIR-1792:rev	Intergenic		chr12: 47,785,448-47,785,512
MCE-MIR-1792:rev	Intergenic		chr9: 105,277,879-105,277,943
MCE-MIR-18:fwd	Intron/Exon Intersection parallel	Cecr5	chr6: 120,535,883-120,536,005
MCE-MIR-18:fwd	UTR parallel	11134C4Rik	chr12: 98,535,678-98,535,753
MCE-MIR-18:fwd	Intergenic		chr17: 3,085,522-3,085,590
MCE-MIR-188:fwd	Intron parallel	C3314B19Rik	chr13: 64,266,185-64,266,314
MCE-MIR-188:fwd	Intergenic		chr9: 100,793,860-100,793,973
MCE-MIR-1905:rev	Intron/Exon Intersection parallel	Rps1	chr17: 25,819,632-25,820,053
MCE-MIR-1905:rev	Intergenic		chr16: 20,371,226-20,371,659
MCE-MIR-1905:rev	Intergenic		chr3: 95,496,198-95,496,631
MCE-MIR-1931:fwd	UTR-Intersection parallel	Ccnl1	chr3: 66,047,602-66,047,824
MCE-MIR-1931:fwd	UTR-Intersection parallel	Ccnl1	chr3: 66,047,602-66,047,821
MCE-MIR-1998:fwd	Intron/Exon Intersection parallel	Serpinb1a	chr13: 32,212,885-32,213,023
MCE-MIR-1998:fwd	Intergenic		chr13: 32,227,564-32,227,645
MCE-MIR-2092:fwd	Intron/Exon Intersection parallel	Rps14	chr18: 61,166,402-61,166,621
MCE-MIR-2092:fwd	Intergenic		chr15: 82,056,967-82,057,192
MCE-MIR-2092:fwd	Intergenic		chr9: 49,051,070-49,051,295
MCE-MIR-2092:fwd	Intergenic		chr3: 53,821,677-53,821,902
MCE-MIR-2092:fwd	Intergenic		chr3: 88,082,168-88,082,393
MCE-MIR-2099:fwd	Intron parallel	Ide	chr19: 36,996,060-36,996,285
MCE-MIR-2099:fwd	Intron parallel	Nup93	chr8: 92,812,231-92,812,456
MCE-MIR-2099:fwd	Intron parallel	Ide	chr19: 36,996,040-36,996,259
MCE-MIR-2099:fwd	Intron parallel	Nup93	chr8: 92,812,257-92,812,476
MCE-MIR-2099:fwd	Intron parallel	Ilirl2	chr1: 40,593,230-40,593,449
MCE-MIR-2099:fwd	Intron parallel	Ide	chr19: 36,996,039-36,996,285
MCE-MIR-2099:fwd	Intron parallel	Nup93	chr8: 92,812,231-92,812,477
MCE-MIR-2099:fwd	Intron/Exon Intersection parallel	Rpli	chrX: 69,658,832-69,658,977
MCE-MIR-2099:fwd	Intergenic		chr15: 52,146,885-52,147,110
MCE-MIR-2099:fwd	Intergenic		chr12: 107,414,694-107,414,919
MCE-MIR-2099:fwd	Intergenic		chr12: 47,785,404-47,785,629
MCE-MIR-2099:fwd	Intergenic		chr9: 105,277,835-105,278,060
MCE-MIR-2099:fwd	Intergenic		chr9: 50,323,386-50,323,611
MCE-MIR-2099:fwd	Intergenic		chr2: 105,405,213-105,405,438

MCE-MIR	Genomic Context	RefSeq Gene	Position (mm7)
MCE-MIR-2099:fwd	Intergenic		chr12: 107,414,674-107,414,893
MCE-MIR-2099:fwd	Intergenic		chr12: 47,785,384-47,785,603
MCE-MIR-2099:fwd	Intergenic		chr9: 50,323,366-50,323,585
MCE-MIR-2099:fwd	Intergenic		chr5: 21,969,482-21,969,701
MCE-MIR-2099:fwd	Intergenic		chr2: 105,405,193-105,405,412
MCE-MIR-2099:fwd	Intergenic		chr12: 107,414,673-107,414,919
MCE-MIR-2099:fwd	Intergenic		chr12: 47,785,383-47,785,629
MCE-MIR-2099:fwd	Intergenic		chr9: 50,323,365-50,323,611
MCE-MIR-2099:fwd	Intergenic		chr2: 105,405,192-105,405,438
MCE-MIR-2111:fwd	Intron/Exon Intersection parallel	Rpl23	chr11: 97,890,355-97,890,566
MCE-MIR-2111:fwd	Intergenic		chr6: 66,857,271-66,857,496
MCE-MIR-2197:fwd	Intergenic		chr1_random: 4,216,884-4,216,975
MCE-MIR-2197:fwd	Intergenic		chr14: 31,603,577-31,603,666
MCE-MIR-2198:fwd	Intergenic		chr3: 39,034,740-39,034,807
MCE-MIR-2198:fwd	Intergenic		chr17: 61,779,344-61,779,443
MCE-MIR-2198:fwd	Intergenic		chr17: 4,826,880-4,826,979
MCE-MIR-2198:fwd	Intergenic		chr4: 105,170,988-105,171,105
MCE-MIR-2198:fwd	Intergenic		chr6: 65,983,463-65,983,562
MCE-MIR-2198:fwd	Intergenic		chr10: 57,604,176-57,604,236
MCE-MIR-2198:fwd	Intergenic		chr6: 71,659,621-71,659,720
MCE-MIR-2198:fwd	Intergenic		chr13: 62,457,608-62,457,707
MCE-MIR-2288:fwd	UTR-Intersection anti	Dynlt1	chr6: 135,363,704-135,363,809
MCE-MIR-2288:fwd	UTR-Intersection anti	Dynlt1	chr13: 45,704,309-45,704,369
MCE-MIR-2339:fwd	UTR-Intersection parallel	H3f3b	chr11: 116,132,935-116,133,154
MCE-MIR-2339:fwd	Intergenic		chr3: 104,546,025-104,546,244
MCE-MIR-2339:fwd	Intergenic		chr1: 178,278,604-178,278,823
MCE-MIR-2339:rev	UTR-Intersection anti	H3f3b	chr11: 116,133,004-116,133,112
MCE-MIR-2339:rev	Intergenic		chr1: 178,278,627-178,278,726
MCE-MIR-2339:rev	Intergenic		chr3: 104,546,122-104,546,220
MCE-MIR-2364:rev	Intron/Exon Intersection parallel	Acta1	chr8: 122,480,427-122,480,650
MCE-MIR-2364:rev	Intron/Exon Intersection parallel	Actb	chr5: 141,938,197-141,938,420
MCE-MIR-2364:rev	Intron/Exon Intersection parallel	Acta1	chr8: 122,480,423-122,480,650
MCE-MIR-2364:rev	Intron/Exon Intersection parallel	Actb	chr5: 141,938,193-141,938,420
MCE-MIR-2364:rev	Intron/Exon Intersection parallel	Acta1	chr8: 122,480,425-122,480,650
MCE-MIR-2364:rev	Intron/Exon Intersection parallel	Actb	chr5: 141,938,195-141,938,420
MCE-MIR-2501:fwd	UTR-Intersection anti	Nutf2	chr8: 104,477,728-104,477,949
MCE-MIR-2501:fwd	Intergenic		chr17_random: 4,025,596-4,025,817
MCE-MIR-2501:fwd	Intergenic		chr19: 53,487,770-53,487,991
MCE-MIR-2501:fwd	Intergenic		chr18: 10,689,457-10,689,678

MCE-MIR	Genomic Context	RefSeq Gene	Position (mm7)
MCE-MIR-2501:fwd	Intergenic		chr17: 53,302,072-53,302,293
MCE-MIR-2501:fwd	Intergenic		chr17: 79,235,758-79,235,979
MCE-MIR-2501:fwd	Intergenic		chr10: 111,305,078-111,305,299
MCE-MIR-2691:rev	Exon anti	Hspa8	chr9: 40,733,439-40,733,789
MCE-MIR-2691:rev	Exon anti	Hspa8	chr9: 40,733,439-40,733,871
MCE-MIR-2691:rev	Intergenic		chr5: 5,788,499-5,788,931
MCE-MIR-2691:rev	Intergenic		chr6: 75,310,685-75,311,117
MCE-MIR-2691:rev	Intergenic		chr14: 86,951,024-86,951,456
MCE-MIR-2691:rev	Intergenic		chr10: 14,448,533-14,448,965
MCE-MIR-2691:rev	Intergenic		chr1: 47,888,701-47,889,133
MCE-MIR-2711:rev	Intron/Exon Intersection anti	Myo1c	chr11: 75,753,614-75,754,033
MCE-MIR-2711:rev	Intron parallel	Eppk1	chr15: 76,254,228-76,254,622
MCE-MIR-2722:rev	UTR-Intersection parallel	Rplp1	chr9: 61,940,812-61,941,240
MCE-MIR-2722:rev	Intergenic		chr8: 105,171,811-105,172,118
MCE-MIR-2722:rev	Intergenic		chr4: 33,243,168-33,243,477
MCE-MIR-2745:fwd	Intron/Exon Intersection anti	Cxxc1	chr18: 74,615,132-74,615,343
MCE-MIR-2745:fwd	Intron/Exon Intersection anti	Cxxc1	chr18: 74,615,132-74,615,352
MCE-MIR-2889:fwd	Intron parallel	Hsd17b4	chr18: 50,512,457-50,512,653
MCE-MIR-2889:fwd	UTR-Intersection parallel	Cd63	chr10: 128,412,069-128,412,261
MCE-MIR-2953:fwd	Intergenic		chr10: 128,179,077-128,179,296
MCE-MIR-2953:fwd	Intergenic		chr2: 116,266,465-116,266,684
MCE-MIR-3048:rev	Intron parallel	Ift8	chr3: 69,003,261-69,003,686
MCE-MIR-3048:rev	Intron parallel	Ift8	chr3: 69,003,261-69,003,686
MCE-MIR-3048:rev	Intron/Exon Intersection parallel	Ospb19	chr4: 108,463,646-108,464,071
MCE-MIR-3057:fwd	Intron anti	Tiam2	chr17: 3,461,533-3,461,952
MCE-MIR-3057:fwd	Intron anti	Itsn2	chr12: 4,621,796-4,622,215
MCE-MIR-3057:fwd	Intergenic		chr12: 53,321,724-53,322,143
MCE-MIR-3057:fwd	Intergenic		chr1: 95,348,663-95,349,082
MCE-MIR-3101:rev	Intron/Exon Intersection anti	Serpina3n	chr12: 101,905,533-101,905,752
MCE-MIR-3101:rev	Intron/Exon Intersection anti	Serpina3m	chr12: 101,885,862-101,886,081
MCE-MIR-3101:rev	Intron/Exon Intersection anti	Serpina3h	chr12: 101,745,955-101,746,097
MCE-MIR-3101:rev	Intron/Exon Intersection anti	Serpina3g	chr12: 101,733,532-101,733,660
MCE-MIR-3101:rev	Intron/Exon Intersection anti	Serpina3g	chr12: 101,627,271-101,627,490
MCE-MIR-3101:rev	Intergenic		chr12: 101,811,688-101,811,907
MCE-MIR-3111:fwd	Intron parallel	Trpm3	chr19: 22,550,098-22,550,258
MCE-MIR-3111:fwd	Intron parallel	Il31ra	chr13: 109,734,081-109,734,240
MCE-MIR-3111:fwd	Intron parallel	Camk1d	chr2: 5,296,315-5,296,536
MCE-MIR-3111:fwd	Intron parallel	Trpm3	chr19: 22,550,098-22,550,258
MCE-MIR-3111:fwd	Intron parallel	Camk1d	chr2: 5,296,315-5,296,551

MCE-MIR	Genomic Context	RefSeq Gene	Position (mm7)
MCE-MIR-3111:fwd	Intergenic		chr18: 55,080,710-55,080,933
MCE-MIR-3111:fwd	Intergenic		chr12: 78,404,144-78,404,367
MCE-MIR-3111:fwd	Intergenic		chr3: 5,866,131-5,866,354
MCE-MIR-3111:fwd	Intergenic		chrX: 111,462,588-111,462,795
MCE-MIR-3111:fwd	Intergenic		chr18: 55,080,710-55,080,948
MCE-MIR-3111:fwd	Intergenic		chr3: 5,866,116-5,866,354
MCE-MIR-3111:fwd	Intergenic		chrX: 111,462,588-111,462,810
MCE-MIR-3143:fwd	UTR-Intersection anti	Trpc2	chr7_random: 6,119,109-6,119,328
MCE-MIR-3143:fwd	UTR-Intersection anti	Trpc2	chr7: 98,121,868-98,122,087
MCE-MIR-3261:fwd	Intron anti	Ide	chr19: 36,996,001-36,996,222
MCE-MIR-3261:fwd	Intron anti	Nup93	chr8: 92,812,294-92,812,515
MCE-MIR-3261:fwd	Intron anti	Prps2	chrX: 162,138,343-162,138,564
MCE-MIR-3261:fwd	Intron/Exon Intersection anti	Rpli	chrX: 69,659,249-69,659,470
MCE-MIR-3261:fwd	Intergenic		chr15: 52,146,948-52,147,169
MCE-MIR-3261:fwd	Intergenic		chr12: 107,414,635-107,414,856
MCE-MIR-3261:fwd	Intergenic		chr9: 105,277,776-105,277,997
MCE-MIR-3261:fwd	Intergenic		chr9: 50,323,327-50,323,548
MCE-MIR-3261:fwd	Intergenic		chr8: 105,528,072-105,528,293
MCE-MIR-3261:fwd	Intergenic		chr2: 162,546,435-162,546,646
MCE-MIR-3261:fwd	Intergenic		chr2: 136,140,175-136,140,396
MCE-MIR-3261:fwd	Intergenic		chr2: 105,405,154-105,405,375
MCE-MIR-3408:fwd	Intergenic		chr10: 14,449,488-14,449,580
MCE-MIR-3408:fwd	Intergenic		chr1: 47,888,101-47,888,172
MCE-MIR-3408:fwd	Intergenic		chr1: 46,651,184-46,651,255
MCE-MIR-3439:rev	Intron/Exon Intersection parallel	34C1Rik	chr11: 103,021,944-103,022,153
MCE-MIR-3439:rev	Intergenic		chrX: 105,701,279-105,701,498
MCE-MIR-3444:rev	Exon anti	Rpsa	chr9: 119,826,039-119,826,258
MCE-MIR-3444:rev	Intergenic		chr1_random: 2,603,554-2,603,773
MCE-MIR-3444:rev	Intergenic		chr17: 80,709,387-80,709,606
MCE-MIR-3444:rev	Intergenic		chr14: 14,768,043-14,768,262
MCE-MIR-3444:rev	Intergenic		chr9: 107,022,273-107,022,492
MCE-MIR-3444:rev	Intergenic		chr8: 5,494,876-5,495,095
MCE-MIR-3444:rev	Intergenic		chr10: 128,278,522-128,278,741
MCE-MIR-3444:rev	Intergenic		chr3: 149,973,234-149,973,453
MCE-MIR-3444:rev	Intergenic		chr2: 28,484,754-28,484,973
MCE-MIR-3468:rev	Intron parallel	Pde4d	chr13: 106,399,151-106,399,370
MCE-MIR-3468:rev	Intergenic		chr19: 9,925,771-9,925,990
MCE-MIR-3495:rev	Intron/Exon Intersection parallel	Pabpn1	chr14: 49,763,977-49,764,201
MCE-MIR-3495:rev	Intron/Exon Intersection parallel	Pabpn1	chr14: 49,763,977-49,764,204

MCE-MIR	Genomic Context	RefSeq Gene	Position (mm7)
MCE-MIR-3495:rev	Intergenic		chr1: 190,961,487-190,961,711
MCE-MIR-3495:rev	Intergenic		chr1: 190,961,487-190,961,714
MCE-MIR-3513:fwd	Intergenic		chr17: 38,066,018-38,066,442
MCE-MIR-3513:fwd	Intergenic		chr17: 38,066,018-38,066,299
MCE-MIR-3513:fwd	Intergenic		chr17: 38,066,042-38,066,442
MCE-MIR-3513:fwd	Intergenic		chr17: 38,066,018-38,066,442
MCE-MIR-3513:fwd	Intergenic		chr17: 38,066,018-38,066,442
MCE-MIR-3513:fwd	Intergenic		chr17: 38,066,018-38,066,345
MCE-MIR-3513:fwd	Intergenic		chr17: 38,066,101-38,066,442
MCE-MIR-3513:fwd	Intergenic		chr17: 38,066,070-38,066,442
MCE-MIR-3518:rev	Intron anti	LOC432823	chrUn_random: 11,749,940-11,750,102
MCE-MIR-3518:rev	Intron/Exon Intersection anti	Polr2c	chr8: 93,451,800-93,452,025
MCE-MIR-3518:rev	Intergenic		chr18: 51,381,204-51,381,367
MCE-MIR-3523:rev	UTR-Intersection parallel	23116E2Rik	chr5: 29,939,141-29,939,380
MCE-MIR-3523:rev	Intergenic		chr18: 39,090,640-39,090,879
MCE-MIR-3571:rev	UTR-Intersection anti	Rp128	chr7: 4,368,744-4,368,967
MCE-MIR-3571:rev	Intron parallel	1817P19Rik	chr4: 125,514,474-125,514,635
MCE-MIR-3571:rev	Intergenic		chr6: 117,239,960-117,240,130
MCE-MIR-3571:rev	Intergenic		chr5: 132,718,385-132,718,557
MCE-MIR-3571:rev	Intergenic		chr1: 127,918,713-127,918,936
MCE-MIR-3595:fwd	Intron anti	Elk1	chrX: 19,382,536-19,382,718
MCE-MIR-3595:fwd	Intron/Exon Intersection anti	Rab37	chr11: 115,265,853-115,266,072
MCE-MIR-3609:rev	Intron parallel	Galc	chr12: 95,713,319-95,713,538
MCE-MIR-3609:rev	UTR parallel	Ptbp1	chr10: 79,644,103-79,644,322
MCE-MIR-3609:rev	Intergenic		chr15: 93,051,504-93,051,723
MCE-MIR-3624:fwd	Intron/Exon Intersection parallel	Ybx1	chr4: 118,655,515-118,655,734
MCE-MIR-3624:fwd	Intergenic		chr17: 20,103,401-20,103,620
MCE-MIR-3624:fwd	Intergenic		chr11: 100,041,816-100,042,035
MCE-MIR-3624:fwd	Intergenic		chr7: 62,941,411-62,941,630
MCE-MIR-3624:rev	Intron/Exon Intersection anti	Ybx1	chr4: 118,655,515-118,655,734
MCE-MIR-3624:rev	Intergenic		chr17: 20,103,401-20,103,620
MCE-MIR-3624:rev	Intergenic		chr11: 100,041,816-100,042,035
MCE-MIR-3624:rev	Intergenic		chr7: 62,941,411-62,941,630
MCE-MIR-3628:rev	Intron anti	Eya2	chr2: 165,406,334-165,406,554
MCE-MIR-3628:rev	UTR parallel	Gdi1	chrX: 69,698,235-69,698,455
MCE-MIR-3695:fwd	Intron anti	Usp4	chr9: 108,091,831-108,092,259
MCE-MIR-3695:fwd	Intron anti	Pde6a	chr18: 61,648,551-61,648,979
MCE-MIR-3695:fwd	Intron anti	Sh3rf1	chr8: 59,789,762-59,790,190
MCE-MIR-3695:fwd	Intron anti	Dcamkl2	chr3: 86,977,719-86,978,147

MCE-MIR	Genomic Context	RefSeq Gene	Position (mm7)
MCE-MIR-3695:fwd	Intron anti	1719N19Rik	chr19: 58,712,336-58,712,764
MCE-MIR-3695:fwd	Intron parallel	Terf2	chr8: 105,684,676-105,685,104
MCE-MIR-3695:fwd	Intron parallel	Btbd7	chr12: 100,361,956-100,362,384
MCE-MIR-3695:fwd	Intron/Exon Intersection parallel	Rps2	chr17: 22,903,382-22,903,746
MCE-MIR-3695:fwd	Intergenic		chr4: 149,217,564-149,217,953
MCE-MIR-3695:fwd	Intergenic		chr1: 152,910,491-152,910,919
MCE-MIR-3695:fwd	Intergenic		chr17: 29,834,853-29,835,281
MCE-MIR-3695:fwd	Intergenic		chr16: 91,589,559-91,589,987
MCE-MIR-3695:fwd	Intergenic		chr16: 18,231,099-18,231,527
MCE-MIR-3695:fwd	Intergenic		chr15: 27,128,863-27,129,291
MCE-MIR-3695:fwd	Intergenic		chr13: 72,966,160-72,966,588
MCE-MIR-3695:fwd	Intergenic		chr12: 57,016,334-57,016,762
MCE-MIR-3695:fwd	Intergenic		chr14: 64,021,619-64,022,047
MCE-MIR-3695:fwd	Intergenic		chr14: 70,917,070-70,917,498
MCE-MIR-3695:fwd	Intergenic		chr9: 115,102,551-115,102,979
MCE-MIR-3695:fwd	Intergenic		chr13: 79,422,119-79,422,547
MCE-MIR-3695:fwd	Intergenic		chr4: 126,110,312-126,110,740
MCE-MIR-3695:fwd	Intergenic		chr2: 143,883,377-143,883,805
MCE-MIR-3695:fwd	Intergenic		chr8: 104,183,990-104,184,418
MCE-MIR-3695:fwd	Intergenic		chr8: 60,956,205-60,956,627
MCE-MIR-3695:fwd	Intergenic		chr6: 146,861,133-146,861,561
MCE-MIR-3695:fwd	Intergenic		chr6: 122,733,243-122,733,671
MCE-MIR-3695:fwd	Intergenic		chr5: 140,238,562-140,238,990
MCE-MIR-3695:fwd	Intergenic		chr5: 128,860,853-128,861,281
MCE-MIR-3695:fwd	Intergenic		chr11: 29,553,813-29,554,241
MCE-MIR-3695:fwd	Intergenic		chr4: 67,364,977-67,365,405
MCE-MIR-3695:fwd	Intergenic		chr4: 123,153,897-123,154,325
MCE-MIR-3695:fwd	Intergenic		chr3: 95,284,324-95,284,752
MCE-MIR-3695:fwd	Intergenic		chr3: 65,607,381-65,607,809
MCE-MIR-3695:fwd	Intergenic		chr4: 140,463,673-140,464,101
MCE-MIR-3695:fwd	Intergenic		chrX: 47,991,261-47,991,689
MCE-MIR-3754:rev	Intron/Exon Intersection anti	Zfp289	chr2: 91,117,881-91,117,977
MCE-MIR-3754:rev	Intergenic		chr6: 112,724,886-112,724,956
MCE-MIR-3820:fwd	Intron anti	1817P19Rik	chr4: 125,514,474-125,514,635
MCE-MIR-3820:fwd	Intron anti	1817P19Rik	chr4: 125,514,474-125,514,640
MCE-MIR-3820:fwd	UTR-Intersection parallel	Rpl28	chr7: 4,368,744-4,368,967
MCE-MIR-3820:fwd	UTR-Intersection parallel	Rpl28	chr7: 4,368,739-4,368,970
MCE-MIR-3820:fwd	Intergenic		chr6: 117,239,960-117,240,130
MCE-MIR-3820:fwd	Intergenic		chr5: 132,718,385-132,718,557

MCE-MIR	Genomic Context	RefSeq Gene	Position (mm7)
MCE-MIR-3820:fwd	Intergenic		chr1: 127,918,713-127,918,936
MCE-MIR-3820:fwd	Intergenic		chr5: 132,718,380-132,718,557
MCE-MIR-3820:fwd	Intergenic		chr1: 127,918,708-127,918,939
MCE-MIR-3820:rev	UTR-Intersection anti	Rp128	chr7: 4,368,739-4,368,970
MCE-MIR-3820:rev	UTR-Intersection anti	Rp128	chr7: 4,368,744-4,368,967
MCE-MIR-3820:rev	Intron parallel	1817P19Rik	chr4: 125,514,474-125,514,640
MCE-MIR-3820:rev	Intron parallel	1817P19Rik	chr4: 125,514,474-125,514,635
MCE-MIR-3820:rev	Intergenic		chr5: 132,718,380-132,718,557
MCE-MIR-3820:rev	Intergenic		chr1: 127,918,708-127,918,939
MCE-MIR-3820:rev	Intergenic		chr6: 117,239,960-117,240,130
MCE-MIR-3820:rev	Intergenic		chr5: 132,718,385-132,718,557
MCE-MIR-3820:rev	Intergenic		chr1: 127,918,713-127,918,936
MCE-MIR-3847:fwd	Intergenic		chr17: 38,064,906-38,065,126
MCE-MIR-3847:fwd	Intergenic		chr17: 38,064,904-38,065,126
MCE-MIR-3847:fwd	Intergenic		chr17: 38,064,903-38,065,126
MCE-MIR-3847:rev	Intergenic		chr17: 38,064,906-38,065,126
MCE-MIR-3847:rev	Intergenic		chr17: 38,064,904-38,065,126
MCE-MIR-3847:rev	Intergenic		chr17: 38,064,903-38,065,126
MCE-MIR-3859:rev	Intergenic		chr17: 38,064,944-38,065,027
MCE-MIR-3859:rev	Intergenic		chr17: 38,064,944-38,065,027
MCE-MIR-3859:rev	Intergenic		chr17: 38,064,928-38,065,050
MCE-MIR-3859:rev	Intergenic		chr17: 38,064,927-38,065,050
MCE-MIR-3867:rev	Exon parallel	Hspa8	chr9: 40,733,482-40,733,904
MCE-MIR-3867:rev	Exon parallel	Hspa8	chr9: 40,733,482-40,733,789
MCE-MIR-3867:rev	Intergenic		chr1: 47,888,744-47,889,166
MCE-MIR-3867:rev	Intergenic		chr6: 75,310,652-75,311,074
MCE-MIR-3867:rev	Intergenic		chr5: 5,788,466-5,788,888
MCE-MIR-3867:rev	Intergenic		chr10: 14,448,500-14,448,922
MCE-MIR-3867:rev	Intergenic		chr14: 86,950,991-86,951,413
MCE-MIR-3886:fwd	Intron/Exon Intersection anti	Rpl3	chr15: 80,164,027-80,164,252
MCE-MIR-3886:fwd	Intergenic		chr13: 112,701,054-112,701,279
MCE-MIR-3886:fwd	Intergenic		chr8: 73,816,674-73,816,899
MCE-MIR-3886:fwd	Intergenic		chr3: 92,368,586-92,368,811
MCE-MIR-3886:fwd	Intergenic		chr3: 92,261,218-92,261,443
MCE-MIR-3886:fwd	Intergenic		chrX: 69,558,008-69,558,233
MCE-MIR-3886:fwd	Intergenic		chrX: 11,636,426-11,636,651
MCE-MIR-3886:rev	Exon parallel	Rpl3	chr15: 80,164,122-80,164,201
MCE-MIR-3886:rev	Intergenic		chr13: 112,701,156-112,701,226
MCE-MIR-3886:rev	Intergenic		chr3: 92,261,324-92,261,386

MCE-MIR	Genomic Context	RefSeq Gene	Position (mm7)
MCE-MIR-3888:rev	UTR-Intersection parallel	Rplp1	chr9: 61,940,814-61,941,250
MCE-MIR-3888:rev	Intergenic		chr8: 105,171,813-105,172,118
MCE-MIR-3888:rev	Intergenic		chr4: 33,243,168-33,243,475
MCE-MIR-399:fwd	Intron anti	Hist1h3h	chr13: 22,987,928-22,988,147
MCE-MIR-399:fwd	Intron parallel	Hist1h3h	chr13: 23,104,803-23,105,022
MCE-MIR-399:fwd	Intron parallel	Hist1h3h	chr13: 22,921,238-22,921,457
MCE-MIR-4031:fwd	Intron/Exon Intersection anti	Kctd13	chr7: 123,027,178-123,027,255
MCE-MIR-4031:fwd	Intron/Exon Intersection anti	Kctd13	chr7: 123,027,089-123,027,312
MCE-MIR-406:rev	Intron/Exon Intersection anti	Rps19	chr7: 21,677,786-21,678,010
MCE-MIR-406:rev	Intergenic		chr18: 41,102,685-41,102,909
MCE-MIR-406:rev	Intergenic		chr15: 37,978,926-37,979,150
MCE-MIR-406:rev	Intergenic		chr12: 108,416,562-108,416,786
MCE-MIR-406:rev	Intergenic		chr14: 48,159,183-48,159,407
MCE-MIR-4061:rev	Exon parallel	Cyp4f14	chr17: 31,108,242-31,108,317
MCE-MIR-4061:rev	Exon parallel	Cyp4f13	chr17: 31,128,067-31,128,142
MCE-MIR-4061:rev	Intron/Exon Intersection parallel	Cyp4f16	chr17: 30,748,655-30,748,812
MCE-MIR-4061:rev	Intron/Exon Intersection parallel	Cyp4f16	chr17: 30,748,667-30,748,807
MCE-MIR-4061:rev	Intergenic		chr17: 30,836,449-30,836,606
MCE-MIR-4061:rev	Intergenic		chr17: 30,878,024-30,878,187
MCE-MIR-4061:rev	Intergenic		chr17: 30,726,592-30,726,651
MCE-MIR-4061:rev	Intergenic		chr17: 30,836,461-30,836,601
MCE-MIR-4063:rev	Intron anti	C4a	chr17: 32,905,455-32,905,686
MCE-MIR-4063:rev	Intron/Exon Intersection anti	C4a	chr17: 32,816,499-32,816,730
MCE-MIR-4124:fwd	Intergenic		chr1_random: 5,754,580-5,754,642
MCE-MIR-4124:fwd	Intergenic		chr7: 112,726,321-112,726,384
MCE-MIR-4182:rev	Intron/Exon Intersection parallel	Rpl11	chr4: 135,428,776-135,428,909
MCE-MIR-4182:rev	Intergenic		chr7: 118,934,732-118,934,803
MCE-MIR-4202:rev	Intron/Exon Intersection anti	Ifitm3	chr7: 137,427,579-137,427,799
MCE-MIR-4202:rev	Intergenic		chr8: 13,870,574-13,870,794
MCE-MIR-4297:fwd	Exon anti	Zbed4	chr15: 88,863,361-88,863,580
MCE-MIR-4297:fwd	Intergenic		chr16: 35,875,078-35,875,297
MCE-MIR-4297:fwd	Intergenic		chr12: 42,589,139-42,589,358
MCE-MIR-4297:fwd	Intergenic		chr3: 91,210,677-91,210,896
MCE-MIR-4453:fwd	Intergenic		chr17: 21,142,256-21,142,408
MCE-MIR-4453:fwd	Intergenic		chr11: 109,120,687-109,120,898
MCE-MIR-4453:fwd	Intergenic		chr11: 109,120,687-109,120,897
MCE-MIR-4472:fwd	Intron anti	Vps13a	chr19: 16,416,859-16,417,088
MCE-MIR-4472:fwd	Intron anti	493431B11Rik	chr9: 83,508,048-83,508,277
MCE-MIR-4472:fwd	Intron anti	Slc24a3	chr2: 145,251,142-145,251,371

MCE-MIR	Genomic Context	RefSeq Gene	Position (mm7)
MCE-MIR-4472:fwd	Intron anti	Prim2	chr1: 33,705,252-33,705,481
MCE-MIR-4472:fwd	Intron parallel	BC2463	chrUn_random: 9,646,651-9,646,880
MCE-MIR-4472:fwd	Intron/Exon Intersection parallel	Rps27a	chr11: 29,498,246-29,498,475
MCE-MIR-4472:fwd	Intergenic		chr14: 41,176,018-41,176,247
MCE-MIR-4472:fwd	Intergenic		chr7: 47,690,904-47,691,133
MCE-MIR-4472:fwd	Intergenic		chr6: 122,494,084-122,494,313
MCE-MIR-4472:fwd	Intergenic		chr4: 131,362,932-131,363,161
MCE-MIR-4503:rev	Intergenic		chr7: 90,068,835-90,068,989
MCE-MIR-4503:rev	Intergenic		chr13: 93,152,891-93,152,975
MCE-MIR-4513:fwd	Exon anti	Hspa	chr9: 40,733,534-40,733,759
MCE-MIR-4513:fwd	Exon anti	Hspa8	chr9: 40,733,534-40,733,765
MCE-MIR-4513:fwd	Intergenic		chr14: 86,951,136-86,951,361
MCE-MIR-4513:fwd	Intergenic		chr10: 14,448,645-14,448,870
MCE-MIR-4513:fwd	Intergenic		chr6: 75,310,797-75,311,022
MCE-MIR-4513:fwd	Intergenic		chr5: 5,788,611-5,788,836
MCE-MIR-4513:fwd	Intergenic		chr1: 47,888,796-47,889,021
MCE-MIR-4513:fwd	Intergenic		chr14: 86,951,130-86,951,361
MCE-MIR-4513:fwd	Intergenic		chr10: 14,448,639-14,448,870
MCE-MIR-4513:fwd	Intergenic		chr6: 75,310,791-75,311,022
MCE-MIR-4513:fwd	Intergenic		chr5: 5,788,605-5,788,836
MCE-MIR-4513:fwd	Intergenic		chr1: 47,888,796-47,889,027
MCE-MIR-4625:fwd	UTR anti	Mark2	chr19: 7,096,453-7,096,693
MCE-MIR-4625:fwd	UTR anti	Mark2	chr19: 7,096,454-7,096,693
MCE-MIR-463:fwd	Intron anti	Camkid	chr2: 5,520,900-5,521,121
MCE-MIR-463:fwd	Intron parallel	Myrip	chr9: 120,014,742-120,014,963
MCE-MIR-463:fwd	Intron/Exon Intersection parallel	Rpl19	chr11: 98,137,329-98,137,550
MCE-MIR-463:fwd	Intergenic		chr18: 24,329,674-24,329,895
MCE-MIR-463:fwd	Intergenic		chr15: 7,801,944-7,802,165
MCE-MIR-463:fwd	Intergenic		chr14: 115,117,425-115,117,646
MCE-MIR-463:fwd	Intergenic		chr8: 19,469,745-19,469,966
MCE-MIR-463:fwd	Intergenic		chr7: 111,088,093-111,088,314
MCE-MIR-463:fwd	Intergenic		chr6: 71,701,285-71,701,506
MCE-MIR-463:fwd	Intergenic		chr3: 127,529,736-127,529,957
MCE-MIR-463:fwd	Intergenic		chrX: 4,545,842-4,546,063
MCE-MIR-4673:fwd	Intron parallel	Trpm3	chr19: 22,550,098-22,550,258
MCE-MIR-4673:fwd	Intron parallel	Il31ra	chr13: 109,734,081-109,734,240
MCE-MIR-4673:fwd	Intron parallel	Camk1d	chr2: 5,296,315-5,296,536
MCE-MIR-4673:fwd	Intergenic		chr18: 55,080,710-55,080,933
MCE-MIR-4673:fwd	Intergenic		chr12: 78,404,144-78,404,367

MCE-MIR	Genomic Context	RefSeq Gene	Position (mm7)
MCE-MIR-4673:fwd	Intergenic		chr3: 5,866,131-5,866,354
MCE-MIR-4673:fwd	Intergenic		chrX: 111,462,588-111,462,795
MCE-MIR-4711:fwd	Exon anti	Vps53	chr11: 76,260,989-76,261,038
MCE-MIR-4711:fwd	Intergenic		chr3: 133,972,806-133,972,855
MCE-MIR-4714:fwd	Exon parallel	Acta1	chr8: 122,480,330-122,480,558
MCE-MIR-4714:fwd	Intron/Exon Intersection parallel	Actb	chr5: 141,938,100-141,938,328
MCE-MIR-4714:fwd	Intergenic		chr17: 61,555,100-61,555,328
MCE-MIR-4714:fwd	Intergenic		chr9: 39,003,577-39,003,805
MCE-MIR-4714:fwd	Intergenic		chr7: 4,708,201-4,708,429
MCE-MIR-4714:rev	Exon anti	Acta1	chr8: 122,480,330-122,480,558
MCE-MIR-4714:rev	Intron/Exon Intersection anti	Actb	chr5: 141,938,100-141,938,328
MCE-MIR-4714:rev	Intergenic		chr17: 61,555,100-61,555,328
MCE-MIR-4714:rev	Intergenic		chr9: 39,003,577-39,003,805
MCE-MIR-4714:rev	Intergenic		chr7: 4,708,201-4,708,429
MCE-MIR-4740:fwd	Exon	Pigt	chr17: 38,065,168-38,065,392
MCE-MIR-4740:fwd	Intergenic		chr6: 3,153,443-3,153,667
MCE-MIR-4740:fwd	Intergenic		chr5: 12,200,283-12,200,475
MCE-MIR-4740:fwd	Intergenic		chrX: 22,441,474-22,441,694
MCE-MIR-4763:fwd	Intron/Exon Intersection parallel	Acta1	chr8: 122,480,427-122,480,650
MCE-MIR-4763:fwd	Intron/Exon Intersection parallel	Actb	chr5: 141,938,197-141,938,420
MCE-MIR-4763:fwd	Intron/Exon Intersection parallel	Acta1	chr8: 122,480,425-122,480,650
MCE-MIR-4763:fwd	Intron/Exon Intersection parallel	Actb	chr5: 141,938,195-141,938,420
MCE-MIR-4809:fwd	Intron/Exon Intersection parallel	Rp124	chr16: 55,824,492-55,824,874
MCE-MIR-4809:fwd	Intergenic		chr11: 58,590,317-58,590,745
MCE-MIR-4809:fwd	Intergenic		chr9: 115,712,898-115,713,326
MCE-MIR-4809:fwd	Intergenic		chr7: 136,188,563-136,188,991
MCE-MIR-4809:fwd	Intergenic		chr1: 148,863,511-148,863,939
MCE-MIR-482:fwd	Intron anti	Sh3rf1	chr8: 59,789,942-59,790,050
MCE-MIR-482:fwd	Intergenic		chr12: 4,718,009-4,718,063
MCE-MIR-482:fwd	Intergenic		chr9: 31,837,726-31,837,780
MCE-MIR-482:fwd	Intergenic		chr2: 151,200,264-151,200,332
MCE-MIR-482:rev	Exon anti	Tuba6	chr15: 99,022,480-99,022,610
MCE-MIR-482:rev	Exon anti	Tuba6	chr15: 99,022,454-99,022,629
MCE-MIR-482:rev	Intron parallel	Sh3rf1	chr8: 59,789,962-59,790,033
MCE-MIR-482:rev	Intergenic		chr9: 90,726,297-90,726,427
MCE-MIR-482:rev	Intergenic		chr9: 90,726,271-90,726,446
MCE-MIR-482:rev	Intergenic		chr12: 4,717,964-4,718,094
MCE-MIR-4820:rev	Intron anti	Dock7	chr4: 98,495,856-98,496,075
MCE-MIR-4820:rev	Intron/Exon Intersection anti	Rps4x	chrX: 97,513,713-97,513,932

MCE-MIR	Genomic Context	RefSeq Gene	Position (mm7)
MCE-MIR-4820:rev	Intergenic		chr13: 3,183,605-3,183,824
MCE-MIR-4820:rev	Intergenic		chr13: 34,281,228-34,281,447
MCE-MIR-4820:rev	Intergenic		chr11: 60,530,986-60,531,205
MCE-MIR-4820:rev	Intergenic		chr7: 39,237,065-39,237,284
MCE-MIR-4820:rev	Intergenic		chrX: 134,433,125-134,433,344
MCE-MIR-4820:rev	Intergenic		chr2: 83,197,096-83,197,315
MCE-MIR-4830:fwd	Intron/Exon Intersection parallel	Acta1	chr8: 122,480,278-122,480,683
MCE-MIR-4830:fwd	Intron/Exon Intersection parallel	Actb	chr5: 141,938,015-141,938,453
MCE-MIR-4830:fwd	Intron/Exon Intersection parallel	Actg1	chr11: 120,456,554-120,456,992
MCE-MIR-4830:fwd	Intergenic		chr17: 61,555,015-61,555,453
MCE-MIR-4830:fwd	Intergenic		chr8: 45,006,913-45,007,351
MCE-MIR-4830:fwd	Intergenic		chr9: 39,003,452-39,003,890
MCE-MIR-4830:fwd	Intergenic		chr13: 78,093,574-78,094,011
MCE-MIR-4830:fwd	Intergenic		chr7: 4,708,187-4,708,554
MCE-MIR-4830:fwd	Intergenic		chr16: 64,865,300-64,865,737
MCE-MIR-4830:fwd	Intergenic		chr4: 102,939,892-102,940,330
MCE-MIR-4830:fwd	Intergenic		chr16: 64,865,300-64,865,737
MCE-MIR-4830:fwd	Intergenic		chr6: 133,775,552-133,775,834
MCE-MIR-4978:rev	Intergenic		chr12: 4,718,355-4,718,445
MCE-MIR-4978:rev	Intergenic		chr14: 69,400,153-69,400,242
MCE-MIR-4978:rev	Intergenic		chr9: 90,726,688-90,726,778
MCE-MIR-4978:rev	Intergenic		chr9: 31,837,345-31,837,435
MCE-MIR-5030:rev	UTR parallel	Arf1	chr11: 59,293,339-59,293,445
MCE-MIR-5030:rev	Intergenic		chr1: 44,129,627-44,129,718
MCE-MIR-504:fwd	Intron parallel	Ssh2	chr11: 77,377,052-77,377,471
MCE-MIR-504:fwd	Intron/Exon Intersection parallel	Hsp9aa1	chr12: 108,210,319-108,210,685
MCE-MIR-504:fwd	Intergenic		chr3: 17,736,377-17,736,796
MCE-MIR-5055:fwd	Exon anti	LOC547349	chr17: 33,361,081-33,361,300
MCE-MIR-5055:fwd	Intron anti	LOC195531	chrUn_random: 1,231,668-1,231,880
MCE-MIR-5055:fwd	Intron/Exon Intersection anti	H2-Q7	chr17: 33,494,160-33,494,372
MCE-MIR-5055:fwd	Intron/Exon Intersection anti	H2-Q2	chr17: 33,440,080-33,440,299
MCE-MIR-5055:fwd	Intron/Exon Intersection anti	LOC547349	chr17: 33,418,386-33,418,605
MCE-MIR-5055:fwd	Intron/Exon Intersection anti	H2-T23	chr17: 34,134,973-34,135,192
MCE-MIR-5055:fwd	Intron/Exon Intersection anti	H2-T24	chr17: 34,118,951-34,119,170
MCE-MIR-5055:fwd	Intron parallel	LOC547347	chr17: 34,255,127-34,255,346
MCE-MIR-5055:fwd	Intron parallel	LOC547347	chr17: 34,240,434-34,240,653
MCE-MIR-5055:fwd	Intergenic		chrUn_random: 444,990-445,209
MCE-MIR-5055:fwd	Intergenic		chr17: 33,479,694-33,479,891
MCE-MIR-5055:fwd	Intergenic		chr17: 32,075,729-32,075,948

MCE-MIR	Genomic Context	RefSeq Gene	Position (mm7)
MCE-MIR-5055:rev	Intron anti	LOC547347	chr17: 34,255,127-34,255,346
MCE-MIR-5055:rev	Intron anti	LOC547347	chr17: 34,240,434-34,240,653
MCE-MIR-5055:rev	Exon parallel	LOC547349	chr17: 33,361,081-33,361,300
MCE-MIR-5055:rev	Intron parallel	LOC195531	chrUn_random: 1,231,668-1,231,880
MCE-MIR-5055:rev	Intron parallel	LOC547349	chr17: 33,418,386-33,418,605
MCE-MIR-5055:rev	Intron/Exon Intersection parallel	H2-T23	chr17: 34,134,973-34,135,192
MCE-MIR-5055:rev	Intron/Exon Intersection parallel	H2-T24	chr17: 34,118,951-34,119,170
MCE-MIR-5055:rev	Intron/Exon Intersection parallel	H2-Q7	chr17: 33,494,160-33,494,372
MCE-MIR-5055:rev	Intron/Exon Intersection parallel	H2-Q2	chr17: 33,440,080-33,440,299
MCE-MIR-5055:rev	Intergenic		chrUn_random: 444,990-445,209
MCE-MIR-5055:rev	Intergenic		chr17: 32,075,729-32,075,948
MCE-MIR-5055:rev	Intergenic		chr17: 33,479,694-33,479,891
MCE-MIR-5061:rev	Exon parallel	Slc25a5	chrX: 31,556,745-31,556,968
MCE-MIR-5061:rev	Intergenic		chr1: 71,956,299-71,956,522
MCE-MIR-5068:rev	Exon anti	Tuba6	chr15: 99,022,783-99,023,017
MCE-MIR-5068:rev	Exon anti	Tuba2	chr15: 98,917,245-98,917,479
MCE-MIR-5068:rev	UTR parallel	Slc7a11	chr3: 50,494,935-50,495,169
MCE-MIR-5068:rev	Intergenic		chr17: 5,057,102-5,057,336
MCE-MIR-5068:rev	Intergenic		chr13: 109,750,040-109,750,274
MCE-MIR-5068:rev	Intergenic		chr12: 49,147,482-49,147,716
MCE-MIR-5068:rev	Intergenic		chr9: 90,726,600-90,726,834
MCE-MIR-5068:rev	Intergenic		chr9: 31,837,289-31,837,523
MCE-MIR-5068:rev	Intergenic		chr6: 79,065,703-79,065,937
MCE-MIR-5068:rev	Intergenic		chr2: 151,200,527-151,200,761
MCE-MIR-5141:rev	UTR-Intersection parallel	Pycr2	chr1: 180,884,056-180,884,277
MCE-MIR-5141:rev	UTR-Intersection parallel	Pycr2	chr1: 180,884,051-180,884,277
MCE-MIR-5236:rev	Intron anti	Trip4	chr9: 65,867,877-65,868,296
MCE-MIR-5236:rev	Intron anti	Gpr111	chr17: 40,774,297-40,774,716
MCE-MIR-5236:rev	Intron anti	Adamts19	chr18: 59,319,600-59,320,019
MCE-MIR-5236:rev	Intron anti	Pde6a	chr18: 61,648,564-61,648,983
MCE-MIR-5236:rev	Intron anti	Usp4	chr9: 108,091,844-108,092,263
MCE-MIR-5236:rev	Intron anti	Dcamkl2	chr3: 86,977,715-86,978,134
MCE-MIR-5236:rev	Intron anti	Sh3rf1	chr8: 59,789,775-59,790,194
MCE-MIR-5236:rev	Intron anti	Foxj3	chr4: 118,930,081-118,930,500
MCE-MIR-5236:rev	Intron parallel	Atp8a2	chr14: 54,908,477-54,908,896
MCE-MIR-5236:rev	Intron parallel	Btbd7	chr12: 100,361,969-100,362,388
MCE-MIR-5236:rev	Intron parallel	Terf2	chr8: 105,684,689-105,685,108
MCE-MIR-5236:rev	Intron parallel	Cpa3	chr3: 19,955,763-19,956,182
MCE-MIR-5236:rev	Intron/Exon Intersection parallel	Rps2	chr17: 22,903,378-22,903,746

MCE-MIR	Genomic Context	RefSeq Gene	Position (mm7)
MCE-MIR-5236:rev	Intergenic		chr14: 73,141,715-73,142,134
MCE-MIR-5236:rev	Intergenic		chr16: 91,589,572-91,589,991
MCE-MIR-5236:rev	Intergenic		chr17: 24,727,801-24,728,220
MCE-MIR-5236:rev	Intergenic		chr17: 29,834,866-29,835,285
MCE-MIR-5236:rev	Intergenic		chr12: 57,016,330-57,016,749
MCE-MIR-5236:rev	Intergenic		chr9: 92,326,503-92,326,922
MCE-MIR-5236:rev	Intergenic		chr12: 42,078,396-42,078,749
MCE-MIR-5236:rev	Intergenic		chr13: 72,966,156-72,966,575
MCE-MIR-5236:rev	Intergenic		chr14: 64,021,615-64,022,034
MCE-MIR-5236:rev	Intergenic		chr14: 51,660,025-51,660,444
MCE-MIR-5236:rev	Intergenic		chr14: 70,917,083-70,917,502
MCE-MIR-5236:rev	Intergenic		chr11: 29,553,826-29,554,245
MCE-MIR-5236:rev	Intergenic		chr11: 77,725,763-77,726,182
MCE-MIR-5236:rev	Intergenic		chr15: 27,128,859-27,129,278
MCE-MIR-5236:rev	Intergenic		chr8: 104,184,003-104,184,422
MCE-MIR-5236:rev	Intergenic		chr8: 82,126,230-82,126,649
MCE-MIR-5236:rev	Intergenic		chr8: 82,178,709-82,179,128
MCE-MIR-5236:rev	Intergenic		chr9: 14,222,222-14,222,639
MCE-MIR-5236:rev	Intergenic		chr13: 79,422,132-79,422,551
MCE-MIR-5236:rev	Intergenic		chr9: 115,102,564-115,102,983
MCE-MIR-5236:rev	Intergenic		chr16: 18,231,112-18,231,531
MCE-MIR-5236:rev	Intergenic		chr3: 95,284,337-95,284,756
MCE-MIR-5236:rev	Intergenic		chr6: 146,861,129-146,861,548
MCE-MIR-5236:rev	Intergenic		chr6: 122,733,239-122,733,658
MCE-MIR-5236:rev	Intergenic		chr5: 140,238,575-140,238,994
MCE-MIR-5236:rev	Intergenic		chr5: 128,860,866-128,861,285
MCE-MIR-5236:rev	Intergenic		chr4: 140,463,669-140,464,088
MCE-MIR-5236:rev	Intergenic		chr4: 126,110,308-126,110,727
MCE-MIR-5236:rev	Intergenic		chr4: 67,364,973-67,365,392
MCE-MIR-5236:rev	Intergenic		chr7: 31,382,937-31,383,356
MCE-MIR-5236:rev	Intergenic		chr4: 21,674,636-21,674,945
MCE-MIR-5236:rev	Intergenic		chr3: 65,607,394-65,607,813
MCE-MIR-5236:rev	Intergenic		chrX: 47,991,257-47,991,676
MCE-MIR-5236:rev	Intergenic		chr2: 170,746,770-170,747,189
MCE-MIR-5236:rev	Intergenic		chr2: 143,883,390-143,883,809
MCE-MIR-5236:rev	Intergenic		chr1: 152,910,504-152,910,923
MCE-MIR-5236:rev	Intergenic		chr1: 7,773,664-7,774,083
MCE-MIR-5236:rev	Intergenic		chr4: 123,153,910-123,154,329
MCE-MIR-5236:rev	Intergenic		chr8: 60,956,212-60,956,631

MCE-MIR	Genomic Context	RefSeq Gene	Position (mm7)
MCE-MIR-5236:rev	Intergenic		chr10: 107,944,590-107,945,009
MCE-MIR-5236:rev	Intergenic		chr7: 106,217,643-106,218,062
MCE-MIR-5260:fwd	Exon parallel	Hspa8	chr9: 40,733,476-40,733,902
MCE-MIR-5260:fwd	Exon parallel	Hspa8	chr9: 40,733,476-40,733,789
MCE-MIR-5260:fwd	Intergenic		chr1: 47,888,738-47,889,164
MCE-MIR-5260:fwd	Intergenic		chr5: 5,788,468-5,788,894
MCE-MIR-5260:fwd	Intergenic		chr14: 86,950,993-86,951,419
MCE-MIR-5260:fwd	Intergenic		chr6: 75,310,654-75,311,080
MCE-MIR-5260:fwd	Intergenic		chr10: 14,448,502-14,448,928
MCE-MIR-5300:fwd	Exon parallel	Socs6	chr18: 89,192,777-89,192,996
MCE-MIR-5300:fwd	Intergenic		chr7_random: 1,156,142-1,156,361
MCE-MIR-5322:fwd	Intron anti	Abr	chr11: 76,595,932-76,596,151
MCE-MIR-5322:fwd	Intron/Exon Intersection parallel	Rpli8	chr7: 41,896,501-41,896,720
MCE-MIR-5322:fwd	UTR-Intersection parallel	Rpli8	chr6: 128,336,952-128,337,171
MCE-MIR-5322:fwd	UTR-Intersection parallel	Rpli8	chr2: 36,815,128-36,815,347
MCE-MIR-5322:fwd	Intergenic		chr11: 85,142,693-85,142,912
MCE-MIR-5322:fwd	Intergenic		chr6: 83,867,399-83,867,618
MCE-MIR-5322:fwd	Intergenic		chr5: 6,786,529-6,786,748
MCE-MIR-5322:fwd	Intergenic		chr2: 167,332,362-167,332,581
MCE-MIR-5322:rev	Exon anti	Rpl18	chr6: 128,337,057-128,337,109
MCE-MIR-5322:rev	Intron parallel	Abr	chr11: 76,596,000-76,596,068
MCE-MIR-5322:rev	Intergenic		chr5: 6,786,535-6,786,673
MCE-MIR-5322:rev	Intergenic		chr6: 83,867,499-83,867,594
MCE-MIR-5322:rev	Intergenic		chr2: 167,332,476-167,332,539
MCE-MIR-5340:fwd	Intron/Exon Intersection anti	Siglecf	chr7: 39,530,859-39,531,079
MCE-MIR-5340:fwd	UTR anti	Siglecg	chr7: 39,587,422-39,587,642
MCE-MIR-5340:fwd	Intergenic		chr7: 40,964,691-40,964,878
MCE-MIR-5366:rev	Intergenic		chrX: 69,611,705-69,611,925
MCE-MIR-5366:rev	Intergenic		chrX: 69,611,711-69,611,933
MCE-MIR-5366:rev	Intergenic		chrX: 69,611,702-69,611,939
MCE-MIR-5367:rev	UTR-Intersection anti	Actg1	chr11: 120,455,334-120,455,567
MCE-MIR-5367:rev	Intergenic		chr13: 78,094,509-78,094,742
MCE-MIR-5367:rev	Intergenic		chr8: 45,007,857-45,008,090
MCE-MIR-5367:rev	Intergenic		chr4: 102,940,836-102,941,069
MCE-MIR-5389:fwd	Intergenic		chr17: 38,064,906-38,065,126
MCE-MIR-5389:fwd	Intergenic		chr17: 38,064,904-38,065,126
MCE-MIR-5389:rev	Intergenic		chr17: 38,064,906-38,065,126
MCE-MIR-5389:rev	Intergenic		chr17: 38,064,904-38,065,126
MCE-MIR-5399:fwd	Intron parallel	Tmem63c	chr12: 85,070,070-85,070,222

MCE-MIR	Genomic Context	RefSeq Gene	Position (mm7)
MCE-MIR-5399:fwd	Intergenic		chr15: 37,978,963-37,979,202
MCE-MIR-5399:fwd	Intergenic		chr12: 108,416,599-108,416,838
MCE-MIR-5399:fwd	Intergenic		chr8: 39,473,997-39,474,236
MCE-MIR-5399:fwd	Intergenic		chr4: 146,811,292-146,811,531
MCE-MIR-5399:fwd	Intergenic		chr2: 139,813,858-139,814,097
MCE-MIR-5406:rev	Exon parallel	Tuba1	chr15: 98,936,028-98,936,250
MCE-MIR-5406:rev	Exon parallel	Tuba2	chr15: 98,917,574-98,917,796
MCE-MIR-5406:rev	Exon parallel	Tuba6	chr15: 99,022,466-99,022,688
MCE-MIR-5406:rev	Intergenic		chr18: 82,688,224-82,688,446
MCE-MIR-5406:rev	Intergenic		chr17: 5,057,442-5,057,664
MCE-MIR-5406:rev	Intergenic		chr13: 109,750,369-109,750,591
MCE-MIR-5406:rev	Intergenic		chr12: 4,717,950-4,718,172
MCE-MIR-5406:rev	Intergenic		chr9: 90,726,283-90,726,505
MCE-MIR-5454:rev	UTR anti	Pcyt2	chr11: 120,719,200-120,719,421
MCE-MIR-5454:rev	Intergenic		chr1: 61,964,670-61,964,891
MCE-MIR-5503:rev	Intron anti	Gabrb1	chr5: 71,417,941-71,418,160
MCE-MIR-5503:rev	Intron/Exon Intersection anti	Rp15	chr5: 107,080,988-107,081,207
MCE-MIR-5503:rev	Intergenic		chr18: 71,041,666-71,041,885
MCE-MIR-5503:rev	Intergenic		chr15: 30,117,809-30,118,028
MCE-MIR-5503:rev	Intergenic		chr15: 23,032,075-23,032,271
MCE-MIR-5503:rev	Intergenic		chr13: 22,042,367-22,042,586
MCE-MIR-5503:rev	Intergenic		chr12: 117,662,922-117,663,141
MCE-MIR-5503:rev	Intergenic		chr7: 110,621,701-110,621,920
MCE-MIR-5503:rev	Intergenic		chr6: 83,626,564-83,626,783
MCE-MIR-5503:rev	Intergenic		chr1: 15,047,493-15,047,712
MCE-MIR-5581:fwd	Intron anti	Dlgh1	chr16: 31,486,686-31,486,751
MCE-MIR-5581:fwd	Intron anti	Rnf13	chr3: 57,895,615-57,895,689
MCE-MIR-5581:fwd	Intergenic		chr1: 71,397,397-71,397,459
MCE-MIR-5581:fwd	Intergenic		chr2: 152,208,668-152,208,730
MCE-MIR-5581:rev	Intron parallel	Ostf1	chr19: 18,282,799-18,282,860
MCE-MIR-5581:rev	Intron parallel	Nrxn3	chr12: 87,551,045-87,551,193
MCE-MIR-5581:rev	Intergenic		chr17: 22,091,766-22,091,870
MCE-MIR-5581:rev	Intergenic		chr10: 102,916,999-102,917,104
MCE-MIR-5581:rev	Intergenic		chr7: 12,030,397-12,030,459
MCE-MIR-5581:rev	Intergenic		chr2: 152,208,668-152,208,730
MCE-MIR-5581:rev	Intergenic		chr1: 152,090,071-152,090,129
MCE-MIR-5598:fwd	Intron anti	Rab38	chr7: 84,385,732-84,386,157
MCE-MIR-5598:fwd	Intron anti	Reln	chr5: 20,927,608-20,928,033
MCE-MIR-5598:fwd	Intron anti	Rfx4	chr10: 84,335,507-84,335,932

MCE-MIR	Genomic Context	RefSeq Gene	Position (mm7)
MCE-MIR-5598:fwd	Intron anti	843426H19Rik	chrUn_random: 9,276,197-9,276,622
MCE-MIR-5598:fwd	Intron anti	Myo5a	chr9: 75,135,734-75,136,159
MCE-MIR-5598:fwd	Intron anti	Acin1	chr14: 49,548,210-49,548,635
MCE-MIR-5598:fwd	Intron anti	Nkd2	chr13: 70,762,910-70,763,335
MCE-MIR-5598:fwd	Intron parallel	Heph	chrX: 91,812,386-91,812,811
MCE-MIR-5598:fwd	Intron parallel	Cpb2	chr14: 69,945,197-69,945,611
MCE-MIR-5598:fwd	Intron/Exon Intersection parallel	Rpl17	chr18: 75,397,782-75,398,207
MCE-MIR-5598:fwd	Intergenic		chr7: 24,507,679-24,508,104
MCE-MIR-5598:fwd	Intergenic		chr6: 34,970,399-34,970,824
MCE-MIR-5598:fwd	Intergenic		chr1: 170,607,560-170,607,985
MCE-MIR-5598:fwd	Intergenic		chr14: 34,451,553-34,451,926
MCE-MIR-5598:fwd	Intergenic		chr1: 93,214,494-93,214,919
MCE-MIR-5598:fwd	Intergenic		chr1: 77,276,612-77,277,037
MCE-MIR-5598:fwd	Intergenic		chr3: 49,655,593-49,656,018
MCE-MIR-5598:fwd	Intergenic		chr2: 166,827,831-166,828,256
MCE-MIR-5598:fwd	Intergenic		chr5: 21,650,510-21,650,935
MCE-MIR-5598:fwd	Intergenic		chr4: 63,329,234-63,329,659
MCE-MIR-5598:fwd	Intergenic		chrX: 35,229,253-35,229,678
MCE-MIR-5598:fwd	Intergenic		chr1: 43,511,116-43,511,529
MCE-MIR-5598:fwd	Intergenic		chr12: 65,451,873-65,452,298
MCE-MIR-5598:fwd	Intergenic		chr10: 14,289,375-14,289,800
MCE-MIR-5598:fwd	Intergenic		chr7: 33,009,567-33,009,992
MCE-MIR-5598:fwd	Intergenic		chr11: 96,501,287-96,501,712
MCE-MIR-5598:fwd	Intergenic		chr14: 47,403,285-47,403,710
MCE-MIR-5598:fwd	Intergenic		chr13: 77,194,226-77,194,651
MCE-MIR-5598:fwd	Intergenic		chr12: 69,994,851-69,995,276
MCE-MIR-5598:fwd	Intergenic		chr7: 68,498,389-68,498,814
MCE-MIR-5598:fwd	Intergenic		chr12: 80,536,410-80,536,835
MCE-MIR-5598:fwd	Intergenic		chr13: 96,104,099-96,104,524
MCE-MIR-5598:fwd	Intergenic		chr13: 63,179,400-63,179,825
MCE-MIR-5598:fwd	Intergenic		chr15: 73,508,900-73,509,325
MCE-MIR-5598:fwd	Intergenic		chr15: 59,305,047-59,305,472
MCE-MIR-5598:fwd	Intergenic		chr16: 20,346,207-20,346,632
MCE-MIR-5598:fwd	Intergenic		chr18: 38,621,165-38,621,590
MCE-MIR-5598:fwd	Intergenic		chr18_random: 702,105-702,530
MCE-MIR-5598:fwd	Intergenic		chr14: 47,403,286-47,403,710
MCE-MIR-5598:fwd	Intergenic		chr14: 97,441,159-97,441,584
MCE-MIR-5643:fwd	Intron/Exon Intersection anti	Raver1	chr9: 20,951,906-20,952,125
MCE-MIR-5643:fwd	Intergenic		chr9_random: 4,771,908-4,772,127

MCE-MIR	Genomic Context	RefSeq Gene	Position (mm7)
MCE-MIR-5736:rev	UTR parallel	Actg1	chr11: 120,454,675-120,454,897
MCE-MIR-5736:rev	Intergenic		chr17: 61,556,505-61,556,727
MCE-MIR-5736:rev	Intergenic		chr16: 64,863,961-64,864,122
MCE-MIR-5736:rev	Intergenic		chr13: 78,095,179-78,095,396
MCE-MIR-5736:rev	Intergenic		chr10: 24,807,095-24,807,258
MCE-MIR-5736:rev	Intergenic		chr7: 4,705,858-4,706,080
MCE-MIR-5736:rev	Intergenic		chr4: 102,941,506-102,941,728
MCE-MIR-5736:rev	Intergenic		chrX: 46,310,139-46,310,361
MCE-MIR-5914:rev	Intron/Exon Intersection anti	Ebf4	chr2: 130,047,219-130,047,453
MCE-MIR-5914:rev	Intron/Exon Intersection anti	Ebf4	chr2: 130,047,219-130,047,450
MCE-MIR-5970:rev	Intergenic		chr12: 78,591,953-78,592,053
MCE-MIR-5970:rev	Intergenic		chr7: 20,734,341-20,734,432
MCE-MIR-6026:fwd	UTR parallel	Actg1	chr11: 120,454,675-120,454,897
MCE-MIR-6026:fwd	UTR parallel	Actg1	chr11: 120,454,661-120,454,897
MCE-MIR-6026:fwd	Intergenic		chr17: 61,556,505-61,556,727
MCE-MIR-6026:fwd	Intergenic		chr16: 64,863,961-64,864,122
MCE-MIR-6026:fwd	Intergenic		chr13: 78,095,179-78,095,396
MCE-MIR-6026:fwd	Intergenic		chr10: 24,807,095-24,807,258
MCE-MIR-6026:fwd	Intergenic		chr7: 4,705,858-4,706,080
MCE-MIR-6026:fwd	Intergenic		chr4: 102,941,506-102,941,728
MCE-MIR-6026:fwd	Intergenic		chrX: 46,310,139-46,310,361
MCE-MIR-6026:fwd	Intergenic		chr16: 64,863,886-64,864,122
MCE-MIR-6026:fwd	Intergenic		chr13: 78,095,179-78,095,396
MCE-MIR-6026:fwd	Intergenic		chr7: 4,705,844-4,706,080
MCE-MIR-6026:fwd	Intergenic		chr4: 102,941,506-102,941,742
MCE-MIR-6026:rev	UTR anti	Actg1	chr11: 120,454,675-120,454,897
MCE-MIR-6026:rev	UTR anti	Actg1	chr11: 120,454,661-120,454,897
MCE-MIR-6026:rev	Intergenic		chr17: 61,556,505-61,556,727
MCE-MIR-6026:rev	Intergenic		chr16: 64,863,961-64,864,122
MCE-MIR-6026:rev	Intergenic		chr13: 78,095,179-78,095,396
MCE-MIR-6026:rev	Intergenic		chr10: 24,807,095-24,807,258
MCE-MIR-6026:rev	Intergenic		chr7: 4,705,858-4,706,080
MCE-MIR-6026:rev	Intergenic		chr4: 102,941,506-102,941,728
MCE-MIR-6026:rev	Intergenic		chrX: 46,310,139-46,310,361
MCE-MIR-6026:rev	Intergenic		chr16: 64,863,886-64,864,122
MCE-MIR-6026:rev	Intergenic		chr13: 78,095,179-78,095,396
MCE-MIR-6026:rev	Intergenic		chr7: 4,705,844-4,706,080
MCE-MIR-6026:rev	Intergenic		chr4: 102,941,506-102,941,742
MCE-MIR-6033:fwd	Intron parallel	Dock7	chr4: 98,495,756-98,496,176

MCE-MIR	Genomic Context	RefSeq Gene	Position (mm7)
MCE-MIR-6033:fwd	Intron/Exon Intersection parallel	Rps4x	chrX: 97,513,613-97,514,033
MCE-MIR-6033:fwd	Intergenic		chr13: 3,183,504-3,183,924
MCE-MIR-6033:fwd	Intergenic		chr7: 39,236,965-39,237,385
MCE-MIR-6033:fwd	Intergenic		chr13: 34,281,128-34,281,548
MCE-MIR-6033:fwd	Intergenic		chrX: 134,433,025-134,433,445
MCE-MIR-6033:fwd	Intergenic		chr2: 83,196,996-83,197,416
MCE-MIR-6033:fwd	Intergenic		chr11: 60,530,886-60,531,306
MCE-MIR-689:rev	UTR parallel	Hnrpk	chr13: 57,486,551-57,486,971
MCE-MIR-689:rev	Intergenic		chr13: 88,334,155-88,334,574
MCE-MIR-689:rev	Intergenic		chr7: 79,679,026-79,679,389
MCE-MIR-777:rev	Intergenic		chr4: 102,940,999-102,941,062
MCE-MIR-777:rev	Intergenic		chr9: 39,005,064-39,005,135
MCE-MIR-777:rev	Intergenic		chr6: 133,774,818-133,774,902
MCE-MIR-777:rev	Intergenic		chr4: 102,940,985-102,941,069
MCE-MIR-777:rev	Intergenic		chr8: 63,558,564-63,558,651
MCE-MIR-782:rev	UTR anti	Aes	chr10: 81,360,195-81,360,288
MCE-MIR-782:rev	Intergenic		chr7: 16,868,694-16,868,779
MCE-MIR-810:rev	Exon parallel	Tuba1	chr15: 98,935,676-98,935,736
MCE-MIR-810:rev	Exon parallel	Tuba2	chr15: 98,917,222-98,917,282
