# Supplementary material for: Novel MicroRNA Candidates and miRNA-mRNA Pairs in Embryonic Stem (ES) Cells
Source: PLoS One. 2008 Jul 2;3(7):e2548. doi: 10.1371/journal.pone.0002548 (PMC2481296; doi:10.1371/journal.pone.0002548)
Supplement: Table S3 — Sequence Cross Reference Table. This table represents MCE-MIR hairpins exhibiting similarity (17 nt≤match) to miRNAs in miRBase or identified through cloning and other predictions. (0.04 MB PDF) [file pone.0002548.s010.pdf]

MCE ID	CELL[5]	miRBase/piRNA	NAR[19]	NATURE[6]	PNAS[17]	Evofold[33]
MCE-MIR-1190:rev	cand145					
MCE-MIR-1290:fwd	cand895					
MCE-MIR-1307:fwd		mmu-mir-706	miRNA338			
MCE-MIR-1323:rev	cand181					
MCE-MIR-1409:fwd	cand452					
MCE-MIR-1457:fwd	cand368					
MCE-MIR-1496:rev	cand363					
MCE-MIR-1509:fwd	cand720					
MCE-MIR-1522:fwd	cand931					
MCE-MIR-1539:fwd	cand368					
MCE-MIR-174:rev	cand426					
MCE-MIR-1776:rev	cand406					
MCE-MIR-1783:rev	cand879					
MCE-MIR-1817:rev	cand437					
MCE-MIR-1915:fwd	cand17<br>cand661<br>cand913					
MCE-MIR-1915:rev	cand829					
MCE-MIR-1916:fwd	cand17<br>cand661<br>cand913					
MCE-MIR-1916:rev	cand829					
MCE-MIR-1963:fwd	cand92					
MCE-MIR-2006:fwd	cand407					
MCE-MIR-2006:rev	cand719					
MCE-MIR-2035:fwd	cand759					
MCE-MIR-2035:rev	cand581<br>cand704<br>cand793					
MCE-MIR-2040:fwd	cand581<br>cand793					
MCE-MIR-2067:fwd	cand581<br>cand704					
MCE-MIR-2067:rev	cand341					
	cand581<br>cand793					
MCE-MIR-2069:rev	cand581<br>cand704<br>cand793					

MCE ID	CELL[5]	miRBase/piRNA	NAR[19]	NATURE[6]	PNAS[17]	Evofold[33]
MCE-MIR-2070:fwd	cand793					
MCE-MIR-2070:rev	cand581					
MCE-MIR-2092:rev	cand513					Evofold
MCE-MIR-2098:rev		mmu-mir-706	miRNA338			
MCE-MIR-217:rev	cand677					
MCE-MIR-218:rev	cand677					
MCE-MIR-2246:rev	cand525					
MCE-MIR-2247:rev	cand525					
MCE-MIR-2271:rev	cand818					
MCE-MIR-2272:rev	cand818					
MCE-MIR-2287:fwd	cand829					
MCE-MIR-2287:rev	cand17<br>cand913					
MCE-MIR-2323:rev	cand379<br>cand685					
MCE-MIR-2351:fwd	cand793					
MCE-MIR-2402:rev	cand793					
MCE-MIR-240:rev	cand893					
MCE-MIR-2413:fwd	cand17<br>cand913					
MCE-MIR-2413:rev	cand829					
MCE-MIR-2415:fwd	cand519					
MCE-MIR-243:rev	cand677<br>cand704					
MCE-MIR-2451:rev	cand775					
MCE-MIR-2561:fwd	cand488					
MCE-MIR-2628:rev	cand341<br>cand581					
MCE-MIR-2666:rev	cand341<br>cand581					
MCE-MIR-2709:rev	cand710					
MCE-MIR-2728:rev	cand914					
MCE-MIR-2733:fwd	cand503					
MCE-MIR-273:fwd	cand703					
MCE-MIR-2847:fwd	cand950					
MCE-MIR-293:rev	cand689					
MCE-MIR-3017:fwd	cand779					
MCE-MIR-3032:fwd	cand30	mmu-mir-142-3p<br>mmu-mir-142-5p			HSA-MIR-142-3P<br>HSA-MIR-142-5P	Evofold

MCE ID	CELL[5]	miRBase/piRNA	NAR[19]	NATURE[6]	PNAS[17]	Evofold[33]
MCE-MIR-3032:rev				hsa-miR-142-3p		
MCE-MIR-3052:rev	cand262					
MCE-MIR-3092:rev	cand698					
MCE-MIR-3134:rev		mmu-mir-706<br>mmu-mir-709	miRNA338<br>miRNA357			
MCE-MIR-3239:rev	cand572					
MCE-MIR-329:fwd	cand174					
MCE-MIR-3382:rev	cand779					
MCE-MIR-3421:fwd	cand240					
MCE-MIR-3421:rev	cand240					
MCE-MIR-3477:rev	cand503					Evofold
MCE-MIR-3489:fwd	cand275					
MCE-MIR-3490:fwd	cand275					
MCE-MIR-3530:rev	cand202					
MCE-MIR-3532:rev	cand482					
MCE-MIR-3607:fwd	cand686					
MCE-MIR-3607:rev	cand744					
MCE-MIR-3608:fwd	cand686					
MCE-MIR-3608:rev	cand744					
MCE-MIR-3774:fwd	cand830					
MCE-MIR-3775:fwd	cand830					
MCE-MIR-3824:rev	cand950					
MCE-MIR-3830:rev	cand514					
MCE-MIR-4065:fwd	cand379<br>cand685					
MCE-MIR-406:fwd	cand43	mmu-mir-671	miRNA307			
MCE-MIR-4097:rev	cand465					
MCE-MIR-4190:fwd	cand30					
MCE-MIR-419:fwd	cand17					
MCE-MIR-4309:fwd	cand237					
MCE-MIR-4342:fwd	cand572					Evofold
MCE-MIR-4354:fwd	cand745					
MCE-MIR-4357:fwd	cand379<br>cand685					
MCE-MIR-4440:fwd	cand632					
MCE-MIR-4471:fwd	cand572					
MCE-MIR-4472:fwd	cand572					Evofold
MCE-MIR-4533:fwd	cand703					
MCE-MIR-4625:rev	cand145					

MCE ID	CELL[5]	miRBase/piRNA	NAR[19]	NATURE[6]	PNAS[17]	Evofold[33]
MCE-MIR-4743:fwd	cand914					
MCE-MIR-4746:rev	cand558					
MCE-MIR-4772:fwd	cand686					
MCE-MIR-4772:rev	cand744					
MCE-MIR-4790:fwd	cand689					
MCE-MIR-4791:fwd	cand592	mmu-mir-706<br>mmu-mir-709	miRNA338<br>miRNA357			Evofold
MCE-MIR-4822:rev	cand768					
MCE-MIR-4856:fwd	cand760					
MCE-MIR-4875:fwd	cand837					
MCE-MIR-4881:fwd	cand191<br>cand282<br>cand36			hsa-miR-133a<br>hsa-miR-133b		
MCE-MIR-4881:rev		mmu-mir-133a<br>S-mmu-mir-133a<br>mmu-mir-133b<br>S-mmu-mir-133a	miRNA222		HSA-MIR-133A	
MCE-MIR-4964:rev	cand433<br>cand435<br>cand436<br>cand575<br>cand577<br>cand766					
MCE-MIR-5042:rev	cand428					
MCE-MIR-5062:rev	cand525					
MCE-MIR-5127:rev	cand292					
MCE-MIR-512:fwd	cand793					
MCE-MIR-5184:fwd	cand534					
MCE-MIR-5366:rev	cand503					Evofold
MCE-MIR-5376:fwd	cand576					
MCE-MIR-5399:fwd	cand43	mmu-mir-671	miRNA307			
MCE-MIR-5400:rev	cand418					
MCE-MIR-5440:fwd	cand880					
MCE-MIR-5503:fwd	cand576					
MCE-MIR-5504:fwd	cand407					
MCE-MIR-5504:rev	cand719					
MCE-MIR-5524:fwd	cand341					
MCE-MIR-5533:rev	cand437					

MCE ID	CELL[5]	miRBase/piRNA	NAR[19]	NATURE[6]	PNAS[17]	Evofold[33]
MCE-MIR-5586:fwd	cand410<br>cand413<br>cand598					
MCE-MIR-5616:rev	cand950					
MCE-MIR-5699:fwd	cand110					Evofold
MCE-MIR-5707:fwd	cand572					
MCE-MIR-5871:fwd	cand922					
MCE-MIR-5916:rev	cand503					
MCE-MIR-598:fwd	cand576					
MCE-MIR-6000:fwd	cand407					
MCE-MIR-6000:rev	cand719					
MCE-MIR-6001:rev	cand713					
MCE-MIR-6015:rev	cand141					
MCE-MIR-6034:rev	cand967	mmu-mir-709	miRNA357			
MCE-MIR-6039:fwd	cand691<br>cand793	mmu-mir-669b	miRNA238			
MCE-MIR-6039:rev		mmu-mir-669b	miRNA238			
MCE-MIR-6129:rev	cand780					
MCE-MIR-6173:fwd	cand677					
MCE-MIR-6192:fwd	cand592	mmu-mir-706<br>mmu-mir-709	miRNA338<br>miRNA357			
MCE-MIR-624:rev	cand43					
MCE-MIR-69:fwd	cand614					
MCE-MIR-704:rev	cand780					
MCE-MIR-72:fwd	cand629					
MCE-MIR-745:rev	cand428					
MCE-MIR-787:rev	cand704	mmu-mir-297				
MCE-MIR-789:fwd	cand341<br>cand581					
MCE-MIR-789:rev	cand581<br>cand793					
MCE-MIR-965:rev	cand677					
MCE-MIR-974:rev	cand503					
MCE-MIR-990:rev	cand950					
MCE-MIR-996:rev	cand848					
MCE-MIR-2222:fwd						Evofold
MCE-MIR-3147:fwd						Evofold
MCE-MIR-1352:fwd						Evofold
MCE-MIR-5384:rev						Evofold

MCE ID	CELL[5]	miRBase/piRNA	NAR[19]	NATURE[6]	PNAS[17]	Evofold[33]
MCE-MIR-755:fwd						Evofold
MCE-MIR-2679:fwd						Evofold
MCE-MIR-5088:rev						Evofold
MCE-MIR-5367:rev						Evofold
MCE-MIR-645:fwd						Evofold
MCE-MIR-5192:rev						Evofold
MCE-MIR-3484:rev						Evofold
MCE-MIR-6054:rev						Evofold
MCE-MIR-5443:fwd						Evofold
MCE-MIR-4714:rev						Evofold
MCE-MIR-5068:rev						Evofold
MCE-MIR-5216:rev						Evofold
MCE-MIR-3541:rev						Evofold
MCE-MIR-5470:fwd						Evofold
MCE-MIR-3572:rev						Evofold
MCE-MIR-2345:fwd						Evofold
MCE-MIR-4010:rev						Evofold
MCE-MIR-482:rev						Evofold
MCE-MIR-5704:rev						Evofold
MCE-MIR-4978:rev						Evofold
MCE-MIR-1264:rev						Evofold
MCE-MIR-2371:fwd						Evofold
MCE-MIR-1905:rev						Evofold
MCE-MIR-2371:rev						Evofold
MCE-MIR-3832:rev						Evofold
MCE-MIR-855:rev						Evofold
MCE-MIR-871:rev						Evofold
MCE-MIR-482:fwd						Evofold
MCE-MIR-3619:rev						Evofold
MCE-MIR-3057:rev						Evofold
MCE-MIR-188:fwd						Evofold
MCE-MIR-4303:fwd						Evofold
MCE-MIR-3637:rev						Evofold
MCE-MIR-5598:fwd						Evofold
MCE-MIR-2419:fwd						Evofold
MCE-MIR-2087:rev						Evofold
MCE-MIR-5418:fwd						Evofold
MCE-MIR-5004:rev						Evofold
MCE-MIR-1066:rev						Evofold

MCE ID	CELL[5]	miRBase/piRNA	NAR[19]	NATURE[6]	PNAS[17]	Evofold[33]
MCE-MIR-3886:fwd						Evofold
MCE-MIR-5454:rev						Evofold
MCE-MIR-4763:fwd						Evofold
MCE-MIR-5295:rev						Evofold
MCE-MIR-1756:rev						Evofold
MCE-MIR-3261:fwd						Evofold
MCE-MIR-4714:fwd						Evofold
MCE-MIR-2111:fwd						Evofold
MCE-MIR-3444:rev						Evofold
MCE-MIR-5597:fwd						Evofold
MCE-MIR-4799:rev						Evofold
MCE-MIR-2661:rev						Evofold
MCE-MIR-5406:rev						Evofold
MCE-MIR-4497:rev						Evofold
MCE-MIR-2364:rev						Evofold
MCE-MIR-3646:rev						Evofold
MCE-MIR-3888:rev						Evofold
MCE-MIR-3886:rev						Evofold
MCE-MIR-3113:fwd						Evofold
MCE-MIR-4932:rev						Evofold
MCE-MIR-2722:rev						Evofold
MCE-MIR-871:fwd						Evofold
MCE-MIR-4069:rev						Evofold
MCE-MIR-4762:fwd						Evofold
MCE-MIR-4830:fwd						Evofold
MCE-MIR-5396:rev						Evofold
MCE-MIR-4280:rev						Evofold
MCE-MIR-4614:rev						Evofold
MCE-MIR-3522:rev						Evofold
MCE-MIR-4832:rev						Evofold
MCE-MIR-4280:fwd						Evofold
MCE-MIR-4667:fwd						Evofold
MCE-MIR-2192:rev						Evofold
MCE-MIR-3057:fwd						Evofold
MCE-MIR-855:fwd						Evofold
MCE-MIR-5620:fwd	hsa-pir-018165					
MCE-MIR-5503:fwd	hsa-pir-010095					
MCE-MIR-5389:fwd	rno-pir-008639					

MCE ID	CELL[5]	miRBase/piRNA	NAR[19]	NATURE[6]	PNAS[17]	Evofold[33]
MCE-MIR-4342:fwd	mmu-pir-009238					
MCE-MIR-3859:fwd	rno-pir-008639					
MCE-MIR-399:fwd	hsa-pir-014740					
MCE-MIR-2563:fwd	hsa-pir-009294					
MCE-MIR-3513:fwd	hsa-pir-019675					
MCE-MIR-4472:fwd	mmu-pir-009238					
MCE-MIR-2339:fwd	hsa-pir-013215					
