# Supplementary material for: Novel MicroRNA Candidates and miRNA-mRNA Pairs in Embryonic Stem (ES) Cells
Source: PLoS One. 2008 Jul 2;3(7):e2548. doi: 10.1371/journal.pone.0002548 (PMC2481296; doi:10.1371/journal.pone.0002548)
Supplement: Table S4 — Mouse Array Version 2 data. This table contains the simple detectable values, which lists average signal values of all transcripts on the array, of the ES-GCNF −/− time series. Each array includes 545 MCE-MIR, 266 mmu-mir, 170 Cand, 46 MIR, and 177 S-mmu-mir. A total of 10 arrays were used in this study. This table represents results obtained with the <200 nt RNA probe from the Adult Pool which consist of 18 different fully differentiated tissues from adult mouse. ST4A shows data using small RNA probe from Adult Mouse Panel (Adult Pool). ST4B shows data using small RNA probe from ES. ST4C shows data using small RNA probe from GCNF−/−. (0.49 MB PDF) [file pone.0002548.s011.pdf]

Supplemental Table ST4A: Mouse Array Version 2 data-Adult Mouse Panel (Adult Pool). This table contains the simple detectable values, which lists average signal values of all transcripts on the array, of the ES-GCNF -/- time series. Each array includes 545 MCE-MIR, 266 mmu-mir, 170 Cand, 46 MIR, and 177 S-mmu-mir. A total of 10 arrays were used in this study. This table represents results obtained with the <200 nt RNA probe from the Adult Pool which consist of 18 different fully differentiated tissues from adult mouse.

Array Chip ID:	204	216	246
mmu-mir-451	53,536	65,444	34,574
mmu-mir-16	43,963	37,723	24,651
mmu-mir-26a	43,284	41,197	30,504
mmu-mir-30c	30,195	36,641	17,524
mmu-mir-126-3p	29,582	39,036	22,019
mmu-let-7a	28,945	36,734	19,719
cand284:a	28,165	28,120	15,341
mmu-mir-23b	26,716	27,250	15,221
mmu-mir-30b	26,490	32,828	16,753
mmu-mir-29a	26,385	33,926	18,924
mmu-mir-21	26,329	25,738	18,222
cand709	25,290	28,322	15,642
mmu-let-7c	25,254	30,546	21,025
mmu-mir-1	25,147	37,031	20,413
mmu-mir-122a	24,986	34,378	20,649
mmu-let-7f	24,929	33,280	19,096
mmu-mir-23a	24,798	25,730	14,076
cand137:a	24,537	25,268	13,828
cand118	24,034	26,618	17,234
cand374:a	23,744	29,516	17,558
mmu-let-7d	23,424	31,439	16,245
cand490	23,317	28,343	13,686
cand161	22,915	25,754	14,829
mmu-mir-26b	22,731	21,976	18,076
MIR85	22,039	25,124	13,242
mmu-let-7g	22,022	31,421	16,476
mmu-mir-181a	21,723	25,242	14,409
mmu-mir-125b	20,961	30,150	14,493
cand106:a	20,872	26,003	13,255
cand90:a	20,739	22,938	12,684
cand119	20,696	24,848	14,602

Array Chip ID:	204	216	246
mmu-mir-30a-5p	20,344	25,845	12,362
cand314	20,184	22,536	10,444
mmu-mir-24	19,872	19,769	14,087
cand348	19,649	26,135	11,533
mmu-let-7b	19,634	25,954	16,204
mmu-mir-145	18,889	25,813	8,644
cand297:b	18,073	23,557	10,706
mmu-let-7i	17,922	24,945	15,138
MCE-MIR_4453:fwd	17,610	13,048	9,300
cand497:a	17,152	25,460	7,903
mmu-mir-29b	17,127	25,043	9,157
mmu-mir-143	17,027	22,380	10,383
cand614	16,862	25,149	13,121
mmu-mir-22	16,368	19,771	10,580
mmu-mir-30d	15,934	22,657	8,539
S-mmu-mir-199a	15,817	22,077	13,572
mmu-mir-27b	14,855	17,624	9,069
cand252	14,722	15,477	7,781
cand103:b	14,615	20,159	5,377
mmu-mir-15a	14,429	17,420	9,022
mmu-mir-27a	13,955	17,428	7,515
mmu-mir-124a	13,772	19,760	8,607
mmu-mir-17-5p	13,279	13,142	8,345
mmu-mir-191	13,271	16,148	9,354
mmu-let-7e	13,208	18,864	7,126
cand157	13,196	20,505	7,894
mmu-mir-133b	13,191	21,771	3,742
mmu-mir-30e	12,784	18,256	8,091
mmu-mir-125a	12,523	17,081	8,760
mmu-mir-195	12,467	18,195	10,784
cand375	12,423	17,836	8,475
mmu-mir-103	12,414	14,579	6,367
mmu-mir-99a	12,387	16,984	8,334
mmu-mir-128a	12,308	18,579	8,068
mmu-mir-19b	11,860	12,866	5,964
MIR35	11,845	16,853	8,042
mmu-mir-15b	11,626	13,901	7,164
mmu-mir-20a	11,437	11,968	8,764
mmu-mir-130a	11,363	11,735	7,412

Array Chip ID:	204	216	246
mmu-mir-133a	11,092	19,624	3,757
mmu-mir-25	10,967	11,445	7,003
cand104:a	10,840	15,713	5,263
mmu-mir-148a	10,734	13,171	7,371
mmu-mir-107	10,438	13,485	5,166
mmu-mir-29c	10,398	17,888	5,000
cand1:b	10,347	17,192	5,026
mmu-mir-181b	10,177	14,169	8,670
cand706	10,057	10,015	6,341
mmu-mir-128b	9,945	17,096	6,148
cand304	9,632	14,292	4,877
cand341	9,423	10,780	2,819
mmu-mir-146	9,300	12,862	10,077
mmu-mir-20b	9,293	11,311	5,998
MCE-MIR_4274:fwd	9,043	8,385	4,496
MIR216	8,641	10,575	4,480
mmu-mir-221	8,583	11,583	7,688
MCE-MIR_3626:rev	8,504	7,348	4,336
mmu-mir-424	8,440	14,995	7,490
mmu-mir-200a	8,303	12,069	6,132
cand79	7,957	10,951	3,465
cand497:b	7,842	16,597	3,072
mmu-mir-100	7,805	14,910	4,670
mmu-mir-98	7,744	16,636	4,130
S-mmu-mir-140	7,621	8,709	4,913
mmu-mir-106b	7,549	9,548	4,740
mmu-mir-9	7,508	19,486	6,039
mmu-mir-106a	7,158	10,282	3,539
MCE-MIR_5060:rev	7,106	6,463	6,238
mmu-mir-194	7,066	12,743	5,542
mmu-mir-127	7,023	9,330	5,424
S-mmu-mir-9	7,015	10,717	5,363
mmu-mir-199a	6,948	12,914	3,772
MIR74	6,933	10,203	6,160
mmu-mir-101a	6,904	11,697	3,297
MCE-MIR_1597:fwd	6,854	6,329	4,809
mmu-mir-93	6,813	8,274	4,288
mmu-mir-200b	6,581	9,359	5,064
mmu-mir-99b	6,392	8,986	4,316

Array Chip ID:	204	216	246
MCE-MIR_3820:rev	6,333	6,595	4,749
MIR121	6,157	10,303	4,795
mmu-mir-422b	6,150	10,418	4,126
cand151	6,123	6,525	4,579
cand516	6,102	6,271	4,465
cand337	6,020	8,805	3,358
MCE-MIR_2563:rev	5,664	8,713	3,452
mmu-mir-34c	5,437	9,147	3,376
mmu-mir-199b	5,326	11,458	1,939
mmu-mir-150	5,283	10,873	2,598
cand278:a	5,278	8,056	4,447
cand135:b	5,278	6,529	2,234
mmu-mir-101b	5,099	8,663	2,919
mmu-mir-222	5,048	7,880	3,969
MCE-MIR_1394:rev	4,815	4,935	2,361
mmu-mir-218	4,732	8,436	4,643
MCE-MIR_3468:rev	4,680	5,259	4,344
mmu-mir-152	4,670	7,011	4,414
MCE-MIR_2953:fwd	4,646	6,185	1,682
MIR136	4,574	7,700	2,512
cand315:b	4,507	5,703	2,879
cand268:b	4,470	6,057	1,960
mmu-mir-181c	4,413	11,052	1,226
MIR12	4,292	7,382	3,912
cand336	4,272	6,930	2,164
MCE-MIR_1773:fwd	4,261	4,402	2,817
MCE-MIR_4226:fwd	4,251	6,021	847
cand564:b	4,184	6,158	1,880
cand385	4,101	3,741	3,690
MCE-MIR_3571:rev	4,036	3,789	3,783
mmu-mir-92	4,013	6,720	2,628
mmu-mir-10a	3,869	8,614	3,694
MIR75	3,808	5,648	2,038
mmu-mir-34a	3,781	5,501	1,849
mmu-mir-192	3,753	9,864	2,797
mmu-mir-200c	3,717	5,821	2,994
mmu-mir-10b	3,710	9,549	3,219
cand262	3,657	6,203	2,438
mmu-mir-429	3,624	5,787	3,895

Array Chip ID:	204	216	246
cand144:a	3,605	5,794	1,222
mmu-mir-206	3,583	5,224	3,761
mmu-mir-185	3,580	6,010	2,493
mmu-mir-214	3,531	5,375	2,353
cand350	3,526	5,077	2,668
MIR77	3,455	8,851	2,292
MCE-MIR_3191:rev	3,414	4,731	3,068
mmu-mir-34b	3,268	6,781	1,709
mmu-mir-361	3,258	4,728	2,610
MCE-MIR_4087:fwd	3,211	4,390	1,614
MCE-MIR_1539:rev	3,191	4,299	1,972
cand306	3,147	3,623	3,373
mmu-mir-19a	3,132	6,022	1,387
cand90:b	3,103	7,040	1,536
MIR4	3,089	5,588	1,552
cand342:a	3,071	6,142	1,577
MCE-MIR_2171:rev	3,011	2,094	3,650
MCE-MIR_3502:rev	3,002	3,474	1,987
mmu-mir-203	2,969	5,658	3,504
MCE-MIR_3190:rev	2,962	4,347	1,972
cand172:b	2,960	6,002	2,006
mmu-mir-342	2,893	5,292	2,385
mmu-mir-376b	2,844	4,221	2,227
cand68	2,756	2,268	3,547
mmu-mir-129-3p	2,752	6,943	1,201
mmu-mir-130b	2,654	3,825	1,973
MCE-MIR_3495:rev	2,647	2,195	3,692
mmu-mir-335	2,646	5,405	2,584
cand324:a	2,623	4,126	1,290
cand349:a	2,599	9,534	1,086
mmu-mir-341	2,510	2,032	2,127
mmu-mir-320	2,509	3,220	1,656
MIR43	2,347	6,402	1,273
mmu-mir-155	2,334	3,308	3,531
cand317	2,329	3,160	1,543
cand276:a	2,215	3,628	934
mmu-mir-223	2,156	7,445	1,543
mmu-mir-351	2,155	4,152	1,294
mmu-mir-486	2,118	5,001	1,582

Array Chip ID:	204	216	246
cand97	2,116	4,674	919
cand500:a	2,104	3,967	936
MCE-MIR_1457:rev	2,098	3,383	851
MCE-MIR_5195:rev	2,084	2,443	1,408
MCE-MIR_5699:fwd	1,915	2,657	178
MCE-MIR_3782:rev	1,886	1,975	2,586
mmu-mir-434-3p	1,844	3,911	1,947
mmu-mir-350	1,828	4,178	1,009
mmu-mir-138	1,799	4,481	800
MCE-MIR_4297:fwd	1,785	2,757	684
MCE-MIR_3111:fwd	1,776	2,112	988
mmu-mir-31	1,748	2,982	1,411
MCE-MIR_1535:rev	1,705	1,737	2,150
mmu-mir-142-5p	1,670	5,106	475
mmu-mir-379	1,618	2,837	1,971
mmu-mir-7	1,587	3,140	1,353
MCE-MIR_5367:rev	1,540	1,761	1,918
mmu-mir-449	1,529	3,540	1,128
MIR41	1,525	3,278	1,217
MCE-MIR_1538:fwd	1,472	2,254	827
mmu-mir-142-3p	1,468	10,982	509
cand594	1,451	1,290	1,580
cand595:b	1,431	2,085	619
MCE-MIR_254:fwd	1,425	1,974	709
cand135:a	1,372	2,719	1,029
mmu-mir-541	1,328	2,255	966
mmu-mir-382	1,321	2,672	1,662
cand667:a	1,317	2,379	647
mmu-mir-205	1,310	3,023	672
mmu-mir-204	1,310	4,255	850
MIR124	1,308	2,572	1,420
cand153:a	1,286	3,994	612
MCE-MIR_5109:rev	1,270	1,465	895
mmu-mir-301	1,268	2,537	1,076
cand186	1,264	1,257	909
mmu-mir-28	1,263	2,851	580
MCE-MIR_3595:fwd	1,262	1,806	1,031
cand271	1,258	1,645	847
MCE-MIR_2222:fwd	1,254	1,453	431

Array Chip ID:	204	216	246
cand78:b	1,250	1,274	1,601
mmu-mir-149	1,217	3,117	670
mmu-mir-324-5p	1,216	2,106	740
MCE-MIR_3543:rev	1,199	3,800	217
mmu-mir-18	1,176	2,082	681
mmu-mir-322	1,161	3,019	1,191
cand1:a	1,155	2,712	717
cand294	1,148	1,965	1,086
mmu-mir-186	1,146	2,579	955
MCE-MIR_2164:rev	1,145	1,746	1,037
MCE-MIR_984:rev	1,113	1,273	994
mmu-mir-30a-3p	1,112	2,966	749
MIR30	1,092	3,556	384
MIR257	1,081	3,180	703
cand692:b	1,028	776	1,201
MCE-MIR_3147:fwd	1,025	1,281	1,369
mmu-mir-137	1,014	3,824	1,488
cand371:a	1,004	1,537	1,651
MCE-MIR_4822:rev	998	1,197	629
mmu-mir-487b	992	2,071	788
MCE-MIR_4673:fwd	986	1,632	483
MCE-MIR_3847:fwd	980	1,567	298
cand231	955	1,199	1,393
MCE-MIR_2388:rev	945	1,768	450
mmu-mir-329	930	1,992	766
MCE-MIR_5062:rev	925	1,016	1,677
MCE-MIR_5005:rev	923	1,382	706
MCE-MIR_4861:fwd	917	2,060	220
mmu-mir-148b	896	2,302	778
MCE-MIR_5389:fwd	873	1,392	236
cand489	822	1,024	1,603
mmu-mir-450	821	2,996	639
cand572:b	811	1,689	701
MCE-MIR_4661:fwd	800	2,108	95
cand152:b	795	2,257	397
MCE-MIR_6054:rev	792	729	582
MCE-MIR_136:fwd	789	1,387	284
MCE-MIR_1506:rev	787	1,168	658
MCE-MIR_1038:fwd	765	1,183	1,423

Array Chip ID:	204	216	246
mmu-mir-132	762	3,328	749
MIR112	744	901	972
MCE-MIR_3155:fwd	735	1,095	404
MCE-MIR_968:fwd	728	1,380	435
cand718:b	724	1,034	464
mmu-mir-503	722	1,865	397
cand255	720	855	385
MIR207	716	1,235	542
mmu-mir-187	709	1,896	634
cand647	702	1,083	645
MCE-MIR_5544:rev	692	1,935	
MCE-MIR_2563:fwd	679	1,245	319
MCE-MIR_810:rev	678	1,763	300
MIR88	667	1,348	868
mmu-mir-139	663	2,440	518
cand371:b	661	1,237	469
mmu-mir-337	652	1,338	613
mmu-mir-141	646	1,757	705
MCE-MIR_5374:fwd	639	2,105	310
MCE-MIR_4462:fwd	636	720	1,074
mmu-mir-470	635	1,282	534
mmu-mir-433-3p	632	1,452	518
mmu-mir-338	627	2,630	792
MCE-MIR_1504:rev	615	970	561
MCE-MIR_3470:rev	611	878	1,045
MCE-MIR_4625:fwd	611	1,208	373
MCE-MIR_995:rev	600	861	463
MIR253	592	1,348	230
mmu-mir-154	584	1,499	418
MCE-MIR_1190:fwd	581	1,296	343
mmu-mir-140	580	1,628	592
MCE-MIR_645:fwd	575	1,406	220
mmu-mir-345	572	1,384	282
mmu-mir-210	566	1,014	401
MCE-MIR_3859:fwd	561	1,148	164
MCE-MIR_3491:rev	556	831	656
MCE-MIR_3334:fwd	547	1,092	225
MCE-MIR_5014:fwd	542	540	460
mmu-mir-17-3p	534	1,025	311

Array Chip ID:	204	216	246
cand146	518	2,118	266
MCE-MIR_321:rev	516	1,245	170
MIR165	510	679	1,337
mmu-mir-410	499	1,354	662
mmu-mir-324-3p	475	986	349
MIR141	475	1,545	280
mmu-mir-144	474	3,130	460
mmu-mir-494	474	2,139	293
cand572:a	473	1,042	374
mmu-mir-381	469	1,310	411
MCE-MIR_1778:fwd	468	846	344
mmu-mir-202	462	995	730
MIR202	462	1,139	369
mmu-mir-182	457	997	470
cand523	445	1,639	45
cand137:b	443	1,759	43
MCE-MIR_335:fwd	439	553	491
mmu-mir-383	438	995	715
mmu-mir-344	432	1,053	465
MCE-MIR_5172:fwd	432	1,084	4,442
cand613	429	776	884
MCE-MIR_3686:fwd	425	555	794
MIR102	425	1,027	215
MCE-MIR_2139:fwd	423	679	814
mmu-mir-300	419	1,175	272
cand362	412	827	131
mmu-mir-134	412	684	532
MCE-MIR_2243:fwd	409	746	865
MCE-MIR_3573:rev	400	60	627
cand11:b	398	509	553
MCE-MIR_3134:rev	396	1,731	63
cand26	394	717	555
MCE-MIR_3485:rev	392	754	401
mmu-mir-484	386	1,228	242
cand212	384	1,162	263
MCE-MIR_5291:fwd	379	1,034	45
MIR61	377	1,186	124
mmu-mir-543	374	1,077	427
MCE-MIR_3513:rev	374	641	190

Array Chip ID:	204	216	246
mmu-mir-434-5p	373	1,279	397
mmu-mir-431	368	975	285
MCE-MIR_3685:rev	363	480	444
mmu-mir-290	362	502	106
mmu-mir-212	360	969	359
cand425	359	636	243
MCE-MIR_5399:fwd	342	580	346
MCE-MIR_4913:rev	329	1,587	43
MCE-MIR_4740:fwd	319	569	213
MCE-MIR_1409:fwd	316	371	441
MCE-MIR_2745:fwd	315	349	32
mmu-mir-183	313	678	308
cand284:b	309	1,161	94
mmu-mir-213	307	876	303
MCE-MIR_783:rev	305	618	86
MCE-MIR_3484:rev	302	355	831
MCE-MIR_2698:rev	299	307	809
MCE-MIR_2169:rev	296	778	219
MCE-MIR_5079:rev	292	528	460
MCE-MIR_4030:rev	291	681	165
mmu-mir-376a	289	877	371
cand617	287	542	108
mmu-mir-298	286	736	122
MCE-MIR_822:fwd	283	718	67
MCE-MIR_2310:rev	282	663	43
MCE-MIR_5089:fwd	279	736	154
MCE-MIR_3642:rev	276	560	136
mmu-mir-409	272	831	182
MCE-MIR_3751:fwd	264	714	131
MCE-MIR_3837:rev	263	711	48
MCE-MIR_5192:rev	260	545	364
mmu-mir-153	260	1,032	634
MCE-MIR_3143:fwd	259	464	140
MCE-MIR_755:fwd	251	247	776
MCE-MIR_3416:rev	248	468	739
cand22	246	1,110	302
cand708:b	244	517	47
MCE-MIR_3529:rev	242	1,388	268
MCE-MIR_4854:rev	242	727	44

Array Chip ID:	204	216	246
MCE-MIR_1325:rev	240	416	354
MCE-MIR_5030:rev	238	818	49
mmu-mir-378	233	744	196
mmu-mir-365	232	1,601	247
MCE-MIR_5606:fwd	231	569	156
cand210:a	230	745	90
MCE-MIR_4273:fwd	229	400	469
MCE-MIR_135:fwd	227	341	86
cand315:a	225	507	179
cand116	223	507	219
cand178:a	223	424	133
MCE-MIR_2470:fwd	223	241	105
MCE-MIR_1365:fwd	221	194	974
MCE-MIR_4743:fwd	215	585	30
MCE-MIR_2337:fwd	213	430	41
mmu-mir-362	208	496	161
mmu-mir-126-5p	207	8,764	535
MCE-MIR_5057:fwd	207	505	40
MCE-MIR_3503:rev	198	298	345
cand268:a	198	381	1,220
MCE-MIR_2817:fwd	198	290	447
MCE-MIR_3295:rev	197	233	344
MCE-MIR_1734:fwd	196	538	70
mmu-mir-328	195	685	99
mmu-mir-363	193	600	180
mmu-mir-375	193	816	267
MCE-MIR_5403:fwd	191	518	46
MCE-MIR_3488:rev	191	310	458
cand13	190	474	190
MCE-MIR_4999:rev	189	211	529
mmu-mir-151	188	415	209
mmu-mir-330	178	391	150
cand564:a	178	352	157
MIR167	176	606	103
MCE-MIR_4820:rev	171	238	288
cand345	170	310	86
mmu-mir-331	168	554	79
MCE-MIR_4821:rev	165	234	214
MCE-MIR_4945:fwd	160	241	481

Array Chip ID:	204	216	246
MCE-MIR_2327:fwd	159	204	125
cand50	158	528	211
mmu-mir-346	155	324	131
MCE-MIR_3780:rev	151	578	157
cand648	150	808	27
MCE-MIR_2999:fwd	150	390	83
MIR47	149	2,891	46
mmu-mir-129-5p	149	428	180
MCE-MIR_5061:rev	148	225	421
S-mmu-mir-30e	148	965	58
cand686	144	181	498
mmu-mir-292-5p	143	260	36
cand699	140	186	260
mmu-mir-184	140	301	338
cand279:b	139	179	240
mmu-mir-299	138	585	102
cand557	136	169	214
MCE-MIR_451:rev	135	342	48
cand616:a	134	246	382
cand213	133	269	110
MCE-MIR_4791:fwd	132	408	78
MCE-MIR_4027:rev	132	167	218
MCE-MIR_1746:rev	132	561	65
MCE-MIR_5068:rev	131	75	637
MCE-MIR_4799:rev	131	339	
mmu-mir-380-3p	130	494	313
cand515	130	61	126
MCE-MIR_2680:rev	129	875	121
cand126	126	552	25
MCE-MIR_1792:rev	125	606	39
cand374:b	123	294	89
MCE-MIR_5088:rev	121	60	37
MCE-MIR_1342:fwd	120	125	
cand501	119	206	154
MCE-MIR_725:fwd	119	198	254
mmu-mir-370	119	284	122
cand203	118	590	125
cand302:b	117	834	134
mmu-mir-377	114	440	47

Array Chip ID:	204	216	246
MCE-MIR_3490:rev	114	142	265
MCE-MIR_298:rev	110	156	112
cand324:b	105	507	126
MCE-MIR_5197:rev	104	95	
mmu-mir-485-3p	104	372	69
MCE-MIR_670:fwd	104	257	25
MCE-MIR_273:fwd	103	145	269
cand650	103	302	76
cand106:b	100	223	77
cand78:a	100	244	305
mmu-mir-196a	98	524	396
MIR206	95	147	258
cand297:a	92	315	36
mmu-mir-296	91	271	66
MCE-MIR_4684:fwd	91	123	102
MCE-MIR_3523:rev	91	154	349
MCE-MIR_1829:rev	90	1,593	54
cand707	90	370	52
mmu-mir-188	90	260	179
MCE-MIR_5440:fwd	89	408	25
MCE-MIR_1283:fwd	89	189	164
cand129	88	284	28
mmu-mir-468	88	263	75
mmu-mir-201	87	370	144
mmu-mir-224	86	209	102
MCE-MIR_534:fwd	86	265	
MCE-MIR_4472:fwd	85	177	43
MCE-MIR_988:fwd	85	180	189
MCE-MIR_3609:rev	84	126	80
cand104:b	83	177	99
MCE-MIR_1514:fwd	83	135	31
cand156	81	430	26
MCE-MIR_2524:fwd	81	99	61
cand179:a	81	250	13
MCE-MIR_5511:rev	81	183	
MCE-MIR_530:fwd	80	312	75
mmu-mir-425	79	311	112
MCE-MIR_3685:fwd	78	725	34
S-'mmu-mir-376b	78	400	273

Array Chip ID:	204	216	246
cand24	77	115	138
MCE-MIR_4922:fwd	76	508	64
mmu-mir-292-3p	76	72	81
MCE-MIR_5473:rev	76	210	173
mmu-mir-219	76	591	30
MCE-MIR_543:fwd	76	139	163
MCE-MIR_557:fwd	74	199	182
MCE-MIR_5300:fwd	74	139	130
MCE-MIR_5152:rev	73	214	24
MCE-MIR_5366:rev	73	31	125
cand467	73	185	34
cand346	73	198	93
mmu-mir-323	72	314	90
cand624	71	104	129
mmu-mir-339	71	272	44
MCE-MIR_5363:rev	71	105	127
mmu-mir-467	70	131	69
cand42:b	69	49	77
cand91:a	69	43	161
MCE-MIR_5295:rev	67	150	140
MCE-MIR_4661:rev	66	600	27
cand276:b	65	188	43
MCE-MIR_1352:fwd	65	73	152
MCE-MIR_5322:fwd	64	181	183
S-'mmu-let-7d	64	193	88
MCE-MIR_3624:rev	63	74	144
MCE-MIR_6107:fwd	63	148	
MCE-MIR_3958:fwd	62	64	350
MCE-MIR_638:fwd	62	111	54
cand563	61	299	54
mmu-mir-485-5p	61	185	74
MCE-MIR_4922:rev	61	106	26
MCE-MIR_5141:rev	60	443	24
mmu-mir-539	60	218	310
cand210:b	59	162	35
MIR166	59	101	318
MCE-MIR_5454:rev	59	163	186
MCE-MIR_4763:fwd	58	194	137
cand279:a	58	88	97

Array Chip ID:	204	216	246
MCE-MIR_1986:fwd	58	168	59
MCE-MIR_1066:rev	58	110	35
mmu-mir-369-5p	57	259	106
cand500:b	57	166	57
cand347	57	116	202
MCE-MIR_2983:rev	57	58	30
mmu-mir-489	57	181	111
MCE-MIR_2099:fwd	56	132	52
MCE-MIR_3557:rev	56	72	68
MCE-MIR_3531:rev	56	61	217
MCE-MIR_1973:fwd	56	302	54
MCE-MIR_5643:fwd	56	207	51
mmu-mir-293	55	56	32
MCE-MIR_3572:rev	53	46	122
mmu-mir-291b-5p	52	76	123
MCE-MIR_3762:rev	51	68	102
mmu-mir-294	51	49	
MCE-MIR_5083:rev	50	134	54
MCE-MIR_3628:rev	50	66	124
MCE-MIR_2349:rev	50	88	85
MCE-MIR_2679:fwd	50	44	398
MCE-MIR_5488:rev	49	139	96
mmu-mir-291a-3p	49	54	32
MCE-MIR_5354:rev	49	105	74
MCE-MIR_5193:rev	49	66	56
MCE-MIR_3820:fwd	48	95	120
MCE-MIR_936:rev	48	89	105
mmu-mir-208	48	210	67
MCE-MIR_4752:rev	47	79	128
cand153:b	46	133	42
MCE-MIR_1536:rev	46	74	
cand42:a	45	248	67
MCE-MIR_3886:fwd	44	38	39
MCE-MIR_2889:fwd	44	113	32
MCE-MIR_5443:fwd	44	187	109
cand692:a	43	81	83
mmu-mir-326	43	188	25
mmu-mir-540	43	131	42
MCE-MIR_3541:rev	43	81	33

Array Chip ID:	204	216	246
MCE-MIR_1371:fwd	43	77	57
MCE-MIR_5055:rev	42	58	48
cand588	42	52	127
MCE-MIR_5411:rev	42	401	30
MCE-MIR_4714:fwd	42	76	100
cand545	42	128	26
cand618	42	35	79
cand459	41	91	81
MCE-MIR_1364:fwd	40	75	30
MIR201	39	343	22
MCE-MIR_463:fwd	39	121	91
MCE-MIR_5210:fwd	39	64	112
MCE-MIR_1773:rev	39	41	42
cand457:a	39	128	28
mmu-mir-465	39	159	299
mmu-mir-466	39	105	40
MCE-MIR_3408:fwd	39	70	79
MCE-MIR_4712:fwd	38	87	123
MCE-MIR_3260:rev	37	110	114
MCE-MIR_5056:rev	37	60	49
MCE-MIR_5641:rev	37	145	36
MCE-MIR_4627:fwd	37	162	31
MCE-MIR_4748:rev	37	42	84
MCE-MIR_2714:rev	36	56	22
cand5	36	64	103
MCE-MIR_1784:fwd	36	127	
MCE-MIR_4202:rev	36	55	50
MIR103	35	46	88
MCE-MIR_4714:rev	35	95	
MCE-MIR_3663:fwd	34	36	54
mmu-mir-325	34	93	57
mmu-mir-542-5p	34	102	48
mmu-mir-295	33	36	15
MCE-MIR_5384:rev	33	84	17
MCE-MIR_4791:rev	32	129	44
MCE-MIR_3624:fwd	32		36
mmu-mir-189	32	167	33
cand178:b	32	158	
MCE-MIR_4031:fwd	32	34	76

Array Chip ID:	204	216	246
MCE-MIR_4010:rev	32	47	25
cand6	32	49	77
MCE-MIR_4756:rev	32	60	112
MCE-MIR_4674:fwd	32	123	223
MCE-MIR_4320:rev	32	134	16
cand226	31	104	26
MCE-MIR_1495:fwd	31	43	48
MCE-MIR_3441:fwd	30	30	31
MCE-MIR_5369:rev	30	26	27
MCE-MIR_4022:fwd	30	26	45
cand352:b	30	61	25
MCE-MIR_5216:rev	30	63	29
cand73	30	69	
MCE-MIR_3847:rev	30	32	18
MCE-MIR_2977:rev	30	109	24
MCE-MIR_4726:fwd	30	157	30
mmu-mir-215	29	76	120
MCE-MIR_2196:fwd	28	35	53
MCE-MIR_2361:fwd	28	202	16
MCE-MIR_3059:rev	27	20	31
MCE-MIR_2364:rev	27	40	17
cand532:b	27	256	24
MCE-MIR_1611:fwd	26	58	21
MCE-MIR_5389:rev	26	35	36
cand462	26	68	31
cand100:b			
cand103:a		55	26
cand109:b		39	45
cand11:a			
cand115:b		47	
cand120		19	
cand130		48	
cand139			
cand144:b		38	
cand149:a			
cand149:b		27	
cand152:a		22	15
cand172:a		40	21
cand179:b		228	35

Array Chip ID:	204	216	246
cand184		26	
cand192		16	
cand200		45	
cand202			
cand224			
cand234			
cand244		43	
cand25			
cand27		82	
cand275			
cand278:b			
cand286			34
cand302:a			27
cand309			
cand334:a		23	13
cand334:b			23
cand340			17
cand342:b		43	
cand349:b			
cand35			
cand351		91	20
cand352:a			
cand361			
cand386:a			23
cand386:b		31	
cand40			23
cand412		64	24
cand415			31
cand418		104	31
cand427			
cand445		78	
cand457:b		24	19
cand465:a		28	
cand465:b			
cand492			
cand524			
cand529			
cand532:a		36	80
cand541		38	25

Array Chip ID:	204	216	246
cand549			
cand57		97	
cand570		32	
cand585			
cand590			
cand595:a			
cand616:b			
cand619		173	18
cand64			
cand65		20	32
cand664			
cand667:b			
cand669		19	
cand678			
cand690		69	
cand7			
cand70			
cand705		20	22
cand708:a		419	59
cand718:a			20
cand82			
cand91:b			
MCE-MIR_1015:rev		30	49
MCE-MIR_1046:rev		54	45
MCE-MIR_1052:rev			18
MCE-MIR_1059:fwd		17	
MCE-MIR_1074:rev		78	16
MCE-MIR_1192:rev		19	
MCE-MIR_1226:fwd			
MCE-MIR_1259:rev			
MCE-MIR_1264:rev			
MCE-MIR_1269:rev			
MCE-MIR_1283:rev		36	
MCE-MIR_1311:rev			
MCE-MIR_1356:fwd			
MCE-MIR_1356:rev			
MCE-MIR_1365:rev			
MCE-MIR_1401:fwd			
MCE-MIR_1408:fwd		92	

Array Chip ID:	204	216	246
MCE-MIR_1412:fwd			
MCE-MIR_1433:fwd			23
MCE-MIR_1433:rev			
MCE-MIR_1442:fwd			
MCE-MIR_1458:rev		24	41
MCE-MIR_1478:fwd			30
MCE-MIR_1482:rev			
MCE-MIR_1508:fwd		32	
MCE-MIR_151:fwd		16	20
MCE-MIR_1544:rev		27	25
MCE-MIR_1546:rev			
MCE-MIR_1569:rev		40	26
MCE-MIR_1576:rev		18	21
MCE-MIR_162:fwd			22
MCE-MIR_1642:rev			
MCE-MIR_1645:rev			
MCE-MIR_1670:fwd			
MCE-MIR_1679:fwd			
MCE-MIR_1689:rev			
MCE-MIR_1697:fwd			
MCE-MIR_1710:rev			
MCE-MIR_1742:rev		31	22
MCE-MIR_1756:rev		17	24
MCE-MIR_1786:fwd			
MCE-MIR_1788:fwd		27	
MCE-MIR_1788:rev			24
MCE-MIR_1793:fwd			
MCE-MIR_18:fwd			
MCE-MIR_1811:rev		23	23
MCE-MIR_1857:fwd			
MCE-MIR_188:fwd			
MCE-MIR_1905:rev			
MCE-MIR_1929:fwd			
MCE-MIR_1931:fwd		23	29
MCE-MIR_1974:fwd		47	
MCE-MIR_1998:fwd			
MCE-MIR_2078:fwd		30	
MCE-MIR_2087:rev			
MCE-MIR_2092:fwd		22	

Array Chip ID:	204	216	246
MCE-MIR_2111:fwd		23	23
MCE-MIR_2134:fwd		32	51
MCE-MIR_2166:fwd			20
MCE-MIR_2166:rev			
MCE-MIR_2173:rev			
MCE-MIR_2192:rev			
MCE-MIR_2197:fwd			
MCE-MIR_2198:fwd			
MCE-MIR_2205:fwd			
MCE-MIR_2285:fwd		20	24
MCE-MIR_2288:fwd			
MCE-MIR_2304:rev			
MCE-MIR_2339:fwd			14
MCE-MIR_2339:rev			
MCE-MIR_2345:fwd		50	29
MCE-MIR_2371:fwd			
MCE-MIR_2371:rev			
MCE-MIR_2417:fwd			
MCE-MIR_2419:fwd			
MCE-MIR_2464:fwd		30	22
MCE-MIR_2474:rev		45	
MCE-MIR_2501:fwd		19	27
MCE-MIR_2522:rev			
MCE-MIR_2566:fwd		42	18
MCE-MIR_2566:rev			
MCE-MIR_2617:fwd		26	
MCE-MIR_2624:rev			
MCE-MIR_2661:rev			
MCE-MIR_2680:fwd		145	
MCE-MIR_2691:rev			
MCE-MIR_2711:rev			
MCE-MIR_2722:rev			
MCE-MIR_2798:fwd			
MCE-MIR_281:rev			
MCE-MIR_2815:fwd		59	29
MCE-MIR_2866:rev		45	50
MCE-MIR_2894:fwd			
MCE-MIR_2902:fwd			
MCE-MIR_2902:rev		70	

Array Chip ID:	204	216	246
MCE-MIR_291:rev			
MCE-MIR_293:fwd			
MCE-MIR_293:rev			
MCE-MIR_2968:fwd		15	18
MCE-MIR_2986:fwd		71	
MCE-MIR_3007:rev			
MCE-MIR_3032:fwd			
MCE-MIR_3048:rev			
MCE-MIR_3057:fwd			
MCE-MIR_3057:rev			
MCE-MIR_3084:fwd			
MCE-MIR_3101:rev		21	32
MCE-MIR_3113:fwd			
MCE-MIR_3226:fwd			
MCE-MIR_3261:fwd		34	66
MCE-MIR_329:fwd		27	
MCE-MIR_3330:fwd		19	29
MCE-MIR_3333:rev			
MCE-MIR_334:rev		26	
MCE-MIR_3379:fwd			
MCE-MIR_3407:rev			
MCE-MIR_3429:fwd			
MCE-MIR_3429:rev			81
MCE-MIR_3439:rev			20
MCE-MIR_3444:rev			
MCE-MIR_3471:rev			19
MCE-MIR_3474:rev			
MCE-MIR_3477:rev		20	31
MCE-MIR_3478:rev			54
MCE-MIR_3492:fwd			
MCE-MIR_3513:fwd			
MCE-MIR_3518:rev			38
MCE-MIR_3522:rev			26
MCE-MIR_3550:fwd			19
MCE-MIR_3613:rev		21	64
MCE-MIR_3619:rev			17
MCE-MIR_3637:rev			
MCE-MIR_364:fwd			
MCE-MIR_3646:rev			

Array Chip ID:	204	216	246
MCE-MIR_3651:fwd			57
MCE-MIR_3653:rev		49	35
MCE-MIR_3667:fwd			22
MCE-MIR_3667:rev			
MCE-MIR_3684:rev		39	37
MCE-MIR_3695:fwd			
MCE-MIR_3715:rev			
MCE-MIR_3754:rev		31	51
MCE-MIR_3791:rev		20	
MCE-MIR_3793:rev			
MCE-MIR_3797:fwd		28	22
MCE-MIR_3832:rev			
MCE-MIR_3841:fwd			
MCE-MIR_3859:rev			
MCE-MIR_3867:rev			
MCE-MIR_3886:rev			
MCE-MIR_3888:rev			
MCE-MIR_399:fwd			
MCE-MIR_4015:fwd			16
MCE-MIR_4034:rev			24
MCE-MIR_405:rev			
MCE-MIR_406:rev		58	
MCE-MIR_4060:rev			
MCE-MIR_4061:rev			
MCE-MIR_4063:fwd			
MCE-MIR_4063:rev			
MCE-MIR_4069:rev			
MCE-MIR_407:fwd			
MCE-MIR_4087:rev			
MCE-MIR_4124:fwd			
MCE-MIR_4153:fwd			
MCE-MIR_4179:fwd			
MCE-MIR_4179:rev			
MCE-MIR_4182:rev			
MCE-MIR_4198:fwd		49	33
MCE-MIR_4207:fwd			
MCE-MIR_4209:fwd			
MCE-MIR_4232:fwd			
MCE-MIR_4236:rev			

Array Chip ID:	204	216	246
MCE-MIR_4239:rev		49	31
MCE-MIR_4280:fwd			
MCE-MIR_4280:rev			
MCE-MIR_4303:fwd			
MCE-MIR_4342:fwd		95	62
MCE-MIR_4345:rev		17	
MCE-MIR_4353:rev			
MCE-MIR_4383:rev			
MCE-MIR_4413:fwd			
MCE-MIR_4442:fwd			
MCE-MIR_4449:rev			
MCE-MIR_4474:fwd			
MCE-MIR_4491:fwd			
MCE-MIR_4491:rev			
MCE-MIR_4493:fwd			
MCE-MIR_4497:rev			
MCE-MIR_4503:rev			
MCE-MIR_4513:fwd			
MCE-MIR_4521:fwd			
MCE-MIR_4521:rev			
MCE-MIR_4554:rev		33	25
MCE-MIR_4592:fwd			
MCE-MIR_4607:fwd			
MCE-MIR_4610:fwd			
MCE-MIR_4614:rev		18	13
MCE-MIR_466:fwd		16	
MCE-MIR_4667:fwd			
MCE-MIR_4675:fwd		33	
MCE-MIR_469:fwd		25	
MCE-MIR_4711:fwd		17	
MCE-MIR_4712:rev			
MCE-MIR_4716:fwd		21	
MCE-MIR_4726:rev		52	52
MCE-MIR_4738:fwd			26
MCE-MIR_4745:fwd			
MCE-MIR_4755:fwd		53	25
MCE-MIR_4762:fwd		23	
MCE-MIR_4789:rev			
MCE-MIR_4809:fwd			

Array Chip ID:	204	216	246
MCE-MIR_482:fwd			
MCE-MIR_482:rev			
MCE-MIR_4830:fwd			
MCE-MIR_4832:rev			
MCE-MIR_4853:fwd			
MCE-MIR_4893:fwd		23	20
MCE-MIR_4893:rev		19	
MCE-MIR_4932:rev		33	17
MCE-MIR_4972:fwd			
MCE-MIR_4978:rev		21	
MCE-MIR_5004:rev		52	
MCE-MIR_5008:rev			
MCE-MIR_5033:rev			
MCE-MIR_504:fwd			
MCE-MIR_5046:rev			
MCE-MIR_5055:fwd			14
MCE-MIR_5100:rev			29
MCE-MIR_5105:rev		17	
MCE-MIR_5122:rev		19	
MCE-MIR_5135:fwd		56	
MCE-MIR_5143:rev			
MCE-MIR_5152:fwd			29
MCE-MIR_5167:fwd			
MCE-MIR_5167:rev			
MCE-MIR_5180:fwd			
MCE-MIR_5180:rev			
MCE-MIR_5236:rev			
MCE-MIR_5260:fwd			
MCE-MIR_5276:fwd		59	13
MCE-MIR_5279:fwd			
MCE-MIR_5287:rev			
MCE-MIR_5300:rev			23
MCE-MIR_5303:fwd			
MCE-MIR_5322:rev			
MCE-MIR_5328:rev			
MCE-MIR_5339:rev		60	35
MCE-MIR_5340:fwd			
MCE-MIR_5363:fwd		60	
MCE-MIR_5374:rev			

Array Chip ID:	204	216	246
MCE-MIR_5396:rev			19
MCE-MIR_5406:rev			27
MCE-MIR_5418:fwd		107	
MCE-MIR_5470:fwd		60	
MCE-MIR_5473:fwd		39	31
MCE-MIR_5503:rev		29	
MCE-MIR_5504:rev			
MCE-MIR_5581:fwd			
MCE-MIR_5581:rev			23
MCE-MIR_5596:rev		33	
MCE-MIR_5597:fwd		34	25
MCE-MIR_5598:fwd			
MCE-MIR_5607:rev		95	15
MCE-MIR_5620:fwd		61	35
MCE-MIR_5623:rev			21
MCE-MIR_5704:rev			
MCE-MIR_5712:rev			
MCE-MIR_5736:rev		30	21
MCE-MIR_5745:fwd		68	
MCE-MIR_5790:fwd			
MCE-MIR_5864:fwd			
MCE-MIR_5872:fwd		86	
MCE-MIR_5914:rev		31	
MCE-MIR_5970:rev			
MCE-MIR_6001:rev			
MCE-MIR_6026:fwd		22	
MCE-MIR_6026:rev		89	
MCE-MIR_6033:fwd			
MCE-MIR_6034:rev			
MCE-MIR_6050:rev		76	35
MCE-MIR_6055:rev		44	
MCE-MIR_6084:rev		27	
MCE-MIR_6120:fwd			
MCE-MIR_689:rev			
MCE-MIR_734:fwd			26
MCE-MIR_774:rev			
MCE-MIR_777:rev			
MCE-MIR_780:rev		31	
MCE-MIR_782:rev			

Array Chip ID:	204	216	246
MCE-MIR_809:fwd		27	
MCE-MIR_81:fwd			
MCE-MIR_855:fwd			
MCE-MIR_855:rev			
MCE-MIR_871:fwd		15	18
MCE-MIR_871:rev			
MCE-MIR_89:fwd			
MCE-MIR_942:rev		33	
MCE-MIR_946:rev			
MCE-MIR_959:rev			
MCE-MIR_993:rev			
MIR100			
MIR122		836	
MIR140			
MIR161		16	
MIR169			
MIR177			
MIR180			
MIR184			
MIR188			
MIR220			
MIR237			
MIR255			
MIR52			
MIR70			
MIR71			
MIR79			
mmu-mir-135a		144	70
mmu-mir-135b			
mmu-mir-136			
mmu-mir-190		82	
mmu-mir-193		157	23
mmu-mir-196b		56	
mmu-mir-207		46	51
mmu-mir-211			19
mmu-mir-216		53	25
mmu-mir-217		24	25
mmu-mir-291a-5p		41	
mmu-mir-291b-3p			

Array Chip ID:	204	216	246
mmu-mir-297		29	39
mmu-mir-302			
mmu-mir-302b			
S-mmu-mir-302b			
mmu-mir-302c			
S-mmu-mir-302c		20	
mmu-mir-302d		27	
mmu-mir-32		21	
mmu-mir-33			
mmu-mir-340		78	80
mmu-mir-367			
mmu-mir-369-3p		21	
S-mmu-mir-376a		36	
mmu-mir-376c		16	
mmu-mir-380-5p		85	72
mmu-mir-384		17	
mmu-mir-411			
mmu-mir-412		30	30
mmu-mir-433-5p		105	36
mmu-mir-448		30	
mmu-mir-452		40	
mmu-mir-463		23	
mmu-mir-464		13	
mmu-mir-469		67	20
mmu-mir-471		65	33
mmu-mir-483		30	19
mmu-mir-542-3p		142	116
mmu-mir-546		17	
mmu-mir-547		66	39
mmu-mir-7b		32	
mmu-mir-96		149	32
S-mmu-let-7a-1		32	
S-mmu-let-7a-2		17	
S-mmu-let-7b		68	55
S-mmu-let-7c-1			
S-mmu-let-7c-2			
S-mmu-let-7e		83	17
S-mmu-let-7f-1			
S-mmu-let-7f-2		13	

Array Chip ID:	204	216	246
S-mmu-let-7g		74	40
S-mmu-let-7i		417	89
S-mmu-mir-100		15	
S-mmu-mir-101a		25	
S-mmu-mir-101b			
S-mmu-mir-103-1		140	80
S-mmu-mir-103-2		45	24
S-mmu-mir-106a		19	
S-mmu-mir-106b		1,556	445
S-mmu-mir-107		47	26
S-mmu-mir-10a		148	95
S-mmu-mir-10b		70	78
S-mmu-mir-1-1		28	26
S-mmu-mir-1-2		24	12
S-mmu-mir-122a		1,321	106
S-mmu-mir-124a-1		192	
S-mmu-mir-124a-2		192	47
S-mmu-mir-124a-3		218	60
S-mmu-mir-125a		157	73
S-mmu-mir-125b-1		237	78
S-mmu-mir-125b-2		530	145
S-mmu-mir-127		514	168
S-mmu-mir-128a		26	
S-mmu-mir-128b		697	9
S-mmu-mir-129-1		4,702	320
S-mmu-mir-130a			
S-mmu-mir-132		519	63
S-mmu-mir-133a-1		1,055	274
S-mmu-mir-133a-2		1,130	259
S-mmu-mir-133b		753	
S-mmu-mir-134		70	16
S-mmu-mir-135a-1		28	
S-mmu-mir-135a-2		101	42
S-mmu-mir-137		33	14
S-mmu-mir-138-1		18	
S-mmu-mir-138-2		28	
S-mmu-mir-139		113	17
S-mmu-mir-141		119	29
S-mmu-mir-143		123	22

Array Chip ID:	204	216	246
S-mmu-mir-144		227	27
S-mmu-mir-145		3,335	664
S-mmu-mir-146			
S-mmu-mir-148a		31	12
S-mmu-mir-148b		16	
S-mmu-mir-149		7,173	4,566
S-mmu-mir-150		463	435
S-mmu-mir-151		6,123	2,437
S-mmu-mir-154		1,023	252
S-mmu-mir-15a		94	79
S-mmu-mir-15b		198	32
S-mmu-mir-16-1		267	96
S-mmu-mir-16-2		56	51
S-mmu-mir-18		116	36
S-mmu-mir-181a		348	63
S-mmu-mir-181c		192	52
S-mmu-mir-182		19	24
S-mmu-mir-183			24
S-mmu-mir-184		20	
S-mmu-mir-185			
S-mmu-mir-186			
S-mmu-mir-187		70	
S-mmu-mir-188		32	
S-mmu-mir-190			
S-mmu-mir-191		62	
S-mmu-mir-192		48	
S-mmu-mir-193		255	113
S-mmu-mir-194-1		46	
S-mmu-mir-194-2		175	40
S-mmu-mir-195		68	59
S-mmu-mir-196a-1		19	22
S-mmu-mir-196a-2			22
S-mmu-mir-196b		102	79
S-mmu-mir-199b		22,864	14,886
S-mmu-mir-20		47	15
S-mmu-mir-200a		115	46
S-mmu-mir-200b		197	78
S-mmu-mir-204		329	118
S-mmu-mir-207		5,435	2,876

Array Chip ID:	204	216	246
S-mmu-mir-208		590	152
S-mmu-mir-21		118	65
S-mmu-mir-210		101	40
S-mmu-mir-211		799	1,284
S-mmu-mir-212		487	140
S-mmu-mir-214		454	88
S-mmu-mir-218-1		13	26
S-mmu-mir-218-2		23	15
S-mmu-mir-219-2		1,469	516
S-mmu-mir-22		3,400	1,014
S-mmu-mir-221		182	41
S-mmu-mir-223		147	55
S-mmu-mir-23a		222	66
S-mmu-mir-23b		76	
S-mmu-mir-24-2		2,573	781
S-mmu-mir-25		147	36
S-mmu-mir-26a-1			
S-mmu-mir-27a		91	
S-mmu-mir-27b		187	42
S-mmu-mir-28		911	285
S-mmu-mir-290			
S-mmu-mir-293		24	
S-mmu-mir-294			
S-mmu-mir-295		45	11
S-mmu-mir-296		1,081	226
S-mmu-mir-298		24	
S-mmu-mir-29a		19	
S-mmu-mir-29b-1		293	71
S-mmu-mir-29b-2		426	112
S-mmu-mir-300		99	24
S-mmu-mir-30b		202	48
S-mmu-mir-30c-1		320	25
S-mmu-mir-30c-2		574	125
S-mmu-mir-31		229	85
S-mmu-mir-32			
S-mmu-mir-323		22	
S-mmu-mir-326		2,403	73
S-mmu-mir-328		7,940	1,341
S-mmu-mir-329		494	88

Array Chip ID:	204	216	246
S-mmu-mir-33		30	
S-mmu-mir-330		483	
S-mmu-mir-331		49	37
S-mmu-mir-335			
S-mmu-mir-337		817	456
S-mmu-mir-338		233	
S-mmu-mir-339		179	117
S-mmu-mir-342		435	238
S-mmu-mir-345		624	218
S-mmu-mir-346		1,332	112
S-mmu-mir-34a		249	113
S-mmu-mir-34b		4,320	1,645
S-mmu-mir-34c		1,961	1,075
S-mmu-mir-350		84	24
S-mmu-mir-351		175	102
S-mmu-mir-361		408	112
S-mmu-mir-363		428	35
S-mmu-mir-365-1		1,257	418
S-mmu-mir-365-2		474	149
S-mmu-mir-370		123	30
S-mmu-mir-377		38	
S-mmu-mir-378		11,802	6,831
S-mmu-mir-379		266	64
S-mmu-mir-381		180	16
S-mmu-mir-382		375	169
S-mmu-mir-384		360	111
S-mmu-mir-409		328	
S-mmu-mir-412		191	106
S-mmu-mir-425		6,460	1,452
S-mmu-mir-431		192	59
S-mmu-mir-448		49	
S-mmu-mir-449		28	
S-mmu-mir-451		42	27
S-mmu-mir-465		2,206	720
S-mmu-mir-466		22	
S-mmu-mir-467		972	240
S-mmu-mir-468		90	27
S-mmu-mir-469		7,309	5,824
S-mmu-mir-471		1,083	256

Array Chip ID:	204	216	246
S-mmu-mir-483		1,023	239
S-mmu-mir-484		2,103	1,862
S-mmu-mir-486		432	71
S-mmu-mir-7-1		626	318
S-mmu-mir-7-2		169	70
S-mmu-mir-7b		89	39
S-mmu-mir-92-1		52	
S-mmu-mir-92-2		69	
S-mmu-mir-93		226	80
S-mmu-mir-96		14	
S-mmu-mir-98			
S-mmu-mir-99a			
S-mmu-mir-99b		238	98

Supplemental Table ST4B: Mouse Array Version 2 data-ES. This table contains the simple detectable values, which lists average signal values of all transcripts on the array, of the ES-GCNF -/- time series. Each array includes 545 MCE-MIR, 266 mmu-mir, 170 Cand, 46 MIR, and 177 S-mmu-mir. A total of 10 arrays were used in this study. This table represents results obtained with the <200 nt RNA probe from the embryonic stem cell line (ES).

ES Time:	Day 0		Day 1		Day 3			Day 6	
Array Chip ID:	210	357	124	216	217	222	356	222	245
MCE-MIR_2563:rev	28,952	36,727	29,972	49,192	33,250	28,246	25,091	69,869	54,257
mmu-mir-294	28,798	64,320	43,281	42,890	10,732	15,640	19,635	9,433	847
mmu-mir-292-3p	28,567	43,818	34,043	30,082	10,533	12,013	11,240	7,874	4,207
MCE-MIR_4453:fwd	28,324	31,160	44,292	51,511	57,954	39,555	43,066	57,660	57,702
MCE-MIR_5060:rev	25,093	32,208	38,348	45,306	51,291	43,425	39,407	42,235	64,981
mmu-mir-293	23,576	40,135	28,525	27,838	9,173	10,297	10,247	6,380	2,484
S-mmu-mir-149	20,726	23,745		38,367	39,003	45,642	32,511	30,720	50,082
MCE-MIR_3820:rev	20,358	20,436	29,296	41,186	42,807	42,009	32,476	37,937	54,778
cand706	19,771	28,157	29,660	36,962	40,419	46,497	34,324	36,933	59,225
S-mmu-mir-469	19,453	22,496		35,226	34,265	42,608	28,656	38,421	51,449
cand151	19,098	21,546	24,201	40,635	43,019	43,653	34,770	37,759	59,842
mmu-mir-290	19,066	32,248	22,218	26,737	8,246	11,782	7,252	8,011	2,674
cand516	17,618	19,274	24,701	38,890	42,184	41,325	32,395	36,533	56,808
MCE-MIR_1597:fwd	17,239	16,722	20,555	30,183	37,525	40,445	25,635	30,544	53,097
mmu-mir-295	17,023	23,062	13,438	22,988	3,856	4,122	1,720	832	141

ES Time:	Day 0		Day 1		Day 3			Day 6	
**Array Chip ID:**	**210**	**357**	**124**	**216**	**217**	**222**	**356**	**222**	**245**
mmu-mir-292-5p	16,982	28,005	17,987	21,228	5,874	8,195	5,154	5,799	1,153
MCE-MIR_3571:rev	16,946	13,866	17,439	24,261	27,004	32,765	20,915	25,343	34,971
S-mmu-mir-207	16,624	20,625		31,630	27,495	35,028	27,695	24,489	25,572
MCE-MIR_1773:fwd	15,908	14,420	17,503	23,713	29,390	32,905	26,984	27,507	27,972
MCE-MIR_4822:rev	15,608	20,299	29,598	39,095	36,798	32,968	33,842	17,274	14,810
MCE-MIR_4274:fwd	15,564	19,198	19,537	24,415	30,492	26,323	25,505	32,698	24,878
MCE-MIR_1394:rev	15,135	31,289	30,705	34,363	36,770	38,553	28,609	25,579	25,709
cand385	14,841	12,992	16,612	20,842	30,105	30,941	22,597	26,522	38,633
MCE-MIR_3626:rev	14,811	8,762	10,969	13,306	23,349	17,624	17,655	30,258	21,720
MCE-MIR_5062:rev	14,183	10,110	15,781	19,398	28,143	26,362	21,271	20,999	20,028
MCE-MIR_1539:rev	14,065	19,791	20,222	27,199	32,324	33,934	27,249	28,391	26,712
cand271	12,706	19,711	21,704	20,792	24,953	26,899	20,385	21,543	23,757
cand594	12,696	13,726	15,458	19,605	22,405	20,599	17,756	21,364	15,682
MCE-MIR_3191:rev	12,571	16,380	14,322	21,449	26,261	24,958	23,140	31,419	24,128
MCE-MIR_4087:fwd	12,478	6,591	6,814	10,898	11,841	12,142	11,647	9,529	5,738
cand294	12,352	2,399	4,808	10,456	15,561	15,055	8,683	38,050	14,924
MCE-MIR_3190:rev	11,748	15,806	12,766	20,403	24,699	23,280	22,482	28,790	17,183
S-mmu-mir-328	11,472	19,258		23,312	19,884	28,339	22,008	22,219	12,327
MCE-MIR_3782:rev	11,471	6,376	10,358	17,337	23,885	25,143	16,767	20,503	25,698
MCE-MIR_1457:rev	11,370	15,762	15,240	24,110	26,276	28,525	25,587	25,841	16,178
mmu-mir-17-5p	11,254	16,818	13,471	14,562	14,218	9,820	13,845	21,240	8,732
MCE-MIR_984:rev	10,959	8,066	11,145	14,467	19,901	21,454	17,238	19,510	17,075
MCE-MIR_3111:fwd	10,862	14,272	16,861	22,772	19,404	14,179	23,128	11,735	5,464
MCE-MIR_3468:rev	10,858	9,910	10,261	16,789	21,853	23,913	18,236	24,365	22,871
MCE-MIR_3495:rev	10,577	2,549	3,538	8,723	14,242	14,785	7,141	13,950	15,571
MCE-MIR_3502:rev	10,401	7,601	9,112	13,539	18,010	19,407	17,551	23,371	14,312
MCE-MIR_3503:rev	10,335	7,723	13,858	17,026	25,265	24,519	18,962	21,099	20,262
MCE-MIR_2953:fwd	10,278	9,844	7,667	10,992	19,761	15,094	19,153	26,184	7,959
MCE-MIR_2222:fwd	10,118	7,347	7,729	17,212	22,763	19,013	14,656	14,795	9,315
MCE-MIR_5367:rev	9,658	6,429	8,312	14,904	20,132	20,028	13,700	17,282	17,564
MCE-MIR_254:fwd	9,561	10,946	9,998	15,651	17,475	21,199	17,484	17,883	9,774
MCE-MIR_995:rev	9,519	6,833	10,387	13,539	16,979	20,750	16,125	16,262	11,035
cand595:b	9,466	12,875	14,394	19,481	18,960	27,656	17,790	21,246	14,434
mmu-mir-20a	9,268	16,654	11,786	13,136	12,555	9,253	12,037	19,404	7,182
MCE-MIR_5699:fwd	9,212	9,536	9,447	17,501	11,495	17,484	14,682	14,430	1,653
mmu-mir-291a-3p	8,958	15,451	15,238	20,691	5,145	4,393	2,186	1,470	254
cand78:b	8,864	6,447	6,705	13,230	19,052	18,808	14,898	18,423	14,040
MCE-MIR_4861:fwd	8,752	11,332	9,165	19,552	18,340	22,489	18,628	16,731	7,880

ES Time:	Day 0		Day 1		Day 3			Day 6	
**Array Chip ID:**	**210**	**357**	**124**	**216**	**217**	**222**	**356**	**222**	**245**
mmu-mir-19b	8,720	6,321	2,749	12,949	8,941	7,105	764	9,213	2,535
MCE-MIR_2164:rev	8,715	7,160	7,876	14,401	16,052	19,328	17,637	18,432	11,921
MCE-MIR_2171:rev	8,704	2,662	6,058	6,701	17,168	12,775	12,698	23,740	14,721
MCE-MIR_4226:fwd	8,640	12,036	9,954	12,744	24,360	18,442	21,375	19,446	6,746
cand489	8,525	4,961	9,056	14,354	23,547	19,974	15,699	18,988	18,813
MCE-MIR_1538:fwd	8,494	6,517	12,597	17,318	17,623	24,926	13,626	20,618	8,531
cand11:b	8,395	7,785	8,872	12,906	16,007	15,063	14,045	13,726	9,654
cand186	8,154	10,547	9,436	15,484	21,098	22,669	16,000	17,622	17,867
cand692:b	8,032	5,535	6,533	11,982	17,989	17,996	14,090	14,392	13,873
MCE-MIR_5374:fwd	7,939	991	2,837	6,138	15,414	17,544	7,172	17,028	5,621
mmu-mir-21	7,922	16,753	10,859	7,440	17,111	10,183	18,695	19,232	6,139
cand255	7,907	6,044	9,673	15,094	29,910	20,470	17,170	13,394	7,973
S-mmu-mir-484	7,885	4,950		13,059	16,738	21,478	11,103	17,929	18,576
cand68	7,726	1,137	3,974	7,416	20,907	12,190	12,299	23,240	15,290
MCE-MIR_968:fwd	7,656	13,678	9,700	15,734	16,378	19,273	17,467	16,754	10,171
mmu-mir-25	7,651	14,889	10,213	9,736	8,230	6,282	8,828	12,867	4,673
cand306	7,538	7,782	7,793	14,222	18,216	20,822	19,823	22,936	15,505
MCE-MIR_4673:fwd	7,302	11,583	12,156	16,567	10,856	9,462	20,149	7,325	2,243
MCE-MIR_2388:rev	7,277	5,507	7,399	14,683	24,066	20,015	14,845	20,419	11,346
MCE-MIR_2999:fwd	7,255	15,314	6,230	14,286	15,799	15,030	21,329	10,034	1,852
MIR112	7,220	4,720	5,812	13,570	17,277	17,199	13,238	13,178	10,960
MIR216	6,914	11,073	7,831	10,485	6,568	5,723	8,160	9,833	1,840
MCE-MIR_5195:rev	6,842	5,413	7,151	12,619	16,274	19,385	18,040	15,098	10,952
MCE-MIR_5005:rev	6,687	7,033	7,787	13,095	19,765	16,103	17,144	15,869	6,841
MCE-MIR_1535:rev	6,641	3,252	6,177	10,075	16,606	18,834	11,185	15,424	13,929
cand647	6,634	8,158	6,133	9,142	4,845	2,977	5,008	4,450	1,073
mmu-mir-20b	6,570	12,469	8,705	11,529	8,165	6,134	7,999	10,662	1,962
mmu-mir-302d	6,468	13,090	7,596	7,927	237	131	383	868	189
MCE-MIR_2337:fwd	6,455	7,441	7,531	11,712	11,148	11,515	12,286	10,428	2,117
MCE-MIR_3491:rev	6,403	4,686	7,001	13,820	17,079	17,533	15,210	14,636	10,230
MCE-MIR_3155:fwd	6,336	9,335	10,968	13,492	19,156	13,443	19,552	13,913	5,505
MCE-MIR_3686:fwd	6,307	1,883	4,576	11,018	12,759	15,088	6,456	11,777	8,119
cand341	6,276	4,311	2,181	11,292	4,211	5,181	479	5,614	854
MIR70	6,264	11,984	7,419	7,619	190	82	284	670	146
MCE-MIR_5606:fwd	6,245	4,863	5,842	10,942	13,781	13,709	11,972	11,602	4,753
mmu-mir-106a	6,224	11,598	7,489	10,346	7,732	6,182	8,142	12,559	1,809
MCE-MIR_136:fwd	6,176	6,579	6,648	12,123	15,196	15,306	14,098	12,506	4,812
MCE-MIR_4661:fwd	6,086	9,386	7,158	17,577	9,225	15,561	14,902	12,372	1,580

ES Time:	Day 0		Day 1		Day 3			Day 6	
**Array Chip ID:**	**210**	**357**	**124**	**216**	**217**	**222**	**356**	**222**	**245**
MCE-MIR_1038:fwd	6,061	2,385	4,618	9,157	14,822	17,156	7,983	16,519	13,012
MCE-MIR_5061:rev	6,039	4,392	4,620	9,155	13,549	12,807	11,455	8,722	8,124
MCE-MIR_783:rev	6,037	4,576	6,640	10,672	13,081	13,847	13,264	10,763	2,725
MCE-MIR_4854:rev	6,024	9,467	4,975	14,875	9,426	13,762	16,999	12,004	413
MCE-MIR_822:fwd	6,012	4,550	5,357	12,606	11,125	14,892	12,254	10,681	1,289
MCE-MIR_645:fwd	5,976	6,667	6,181	14,990	14,281	18,964	14,778	14,573	4,891
mmu-mir-92	5,877	12,182	7,528	9,114	5,434	4,141	7,981	5,888	1,341
MCE-MIR_3543:rev	5,869	6,107	4,211	9,844	11,054	11,933	15,290	15,258	1,258
MCE-MIR_1409:fwd	5,795	2,548	6,297	8,146	16,554	14,216	12,468	12,482	6,788
MIR88	5,766	3,719	5,371	13,393	15,327	18,362	10,033	15,840	13,621
MCE-MIR_2310:rev	5,735	6,587	4,281	11,623	13,665	13,324	13,209	6,566	1,285
MCE-MIR_1504:rev	5,652	5,042	5,845	11,664	15,231	15,906	12,524	14,282	5,508
MCE-MIR_3295:rev	5,607	2,418	5,620	7,951	15,024	13,353	11,048	10,920	6,411
S-mmu-mir-346	5,591	6,710		13,353	15,823	14,360	14,155	13,485	2,563
MCE-MIR_3751:fwd	5,525	2,537	2,480	12,324	12,253	10,199	5,908	19,543	4,655
MCE-MIR_321:rev	5,513	7,920	3,262	13,990	18,687	19,560	19,887	18,382	1,821
MIR136	5,445	10,660	6,845	10,520	4,577	4,245	8,043	6,355	1,234
MCE-MIR_135:fwd	5,367	3,760	5,839	9,830	15,375	13,742	11,705	10,520	2,796
mmu-mir-16	5,265	9,161	7,038	5,060	9,363	7,083	6,089	8,443	4,391
cand371:a	5,251	2,050	3,037	9,013	17,065	15,821	12,822	14,987	14,695
MCE-MIR_335:fwd	5,193	2,618	5,572	8,855	15,367	13,051	11,189	12,781	6,455
cand667:a	4,964	4,467	5,475	11,961	11,406	16,420	10,987	13,204	4,493
MCE-MIR_4625:fwd	4,849	5,397	5,798	11,278	11,077	14,901	12,509	12,386	4,617
MCE-MIR_2243:fwd	4,826	2,438	2,852	9,803	11,079	13,394	10,367	10,215	10,935
mmu-mir-127	4,815	6,465	2,886	4,638	9,167	5,620	7,185	15,685	5,988
cand342:a	4,654	10,838	6,965	9,226	3,247	4,191	6,710	5,221	1,054
MCE-MIR_1190:fwd	4,546	5,809	5,536	12,299	11,817	14,968	11,588	13,377	4,394
MCE-MIR_3147:fwd	4,544	2,744	4,000	8,098	12,233	13,856	8,784	12,380	8,351
mmu-mir-182	4,515	7,665	5,174	6,661	3,163	2,280	3,390	2,580	401
mmu-mir-341	4,512	3,363	1,595	3,506	3,827	2,518	4,603	5,953	1,236
mmu-mir-130a	4,496	4,732	2,420	7,028	4,632	4,075	1,876	7,561	3,120
MCE-MIR_5089:fwd	4,493	7,570	4,439	11,986	13,906	12,664	18,030	11,264	2,314
cand172:b	4,419	11,621	6,292	7,699	3,134	3,641	6,518	5,343	1,171
mmu-mir-183	4,359	7,680	4,566	6,222	3,898	2,650	4,185	4,012	704
MCE-MIR_5109:rev	4,333	3,174	5,000	7,517	11,130	15,235	7,580	11,347	9,590
MCE-MIR_3837:rev	4,316	5,217	2,972	13,220	11,765	12,775	16,971	11,656	849
S-mmu-mir-365-1	4,260	6,816		12,830	12,033	20,446	11,107	11,436	5,672
S-mmu-mir-211	4,250	2,442		8,892	11,584	11,798	11,348	9,881	7,565

ES Time:	Day 0		Day 1		Day 3			Day 6	
**Array Chip ID:**	**210**	**357**	**124**	**216**	**217**	**222**	**356**	**222**	**245**
mmu-mir-541	4,228	6,897	3,117	4,217	5,487	4,036	6,075	10,368	3,246
MCE-MIR_2169:rev	4,076	5,795	3,024	11,033	8,625	13,299	10,136	11,203	4,665
cand648	4,036	10,347	7,146	15,023	12,034	17,200	16,994	10,629	667
MCE-MIR_5172:fwd	4,004	333	920	4,724	18,377	15,607	3,975	14,093	16,445
MCE-MIR_3595:fwd	3,999	2,349	2,482	7,180	6,989	11,703	5,728	9,818	6,393
cand252	3,967	4,813	4,710	5,279	6,115	4,221	4,715	9,449	1,732
mmu-mir-26a	3,948	7,877	8,398	2,592	12,746	7,009	11,159	24,609	13,708
MIR61	3,936	3,950	3,974	11,513	17,337	9,263	17,532	8,738	320
mmu-mir-93	3,895	6,697	4,231	5,819	6,715	4,607	6,275	9,711	4,127
MCE-MIR_5544:rev	3,822	6,779	2,257	10,956	4,430	12,233	12,052	9,063	644
cand279:b	3,817	2,402	3,358	6,033	11,755	10,994	9,791	7,852	5,520
MCE-MIR_4820:rev	3,804	3,451	7,426	7,837	14,345	19,717	6,419	6,870	8,523
MCE-MIR_4821:rev	3,767	3,677	7,484	8,212	13,899	19,640	6,008	6,972	7,900
mmu-mir-320	3,762	5,054	4,438	7,326	9,003	10,379	7,550	10,769	5,455
cand616:a	3,762	2,403	2,095	8,605	19,379	14,382	9,023	11,524	9,651
MCE-MIR_810:rev	3,760	5,869	982	4,635	1,531	1,136	2,224	2,077	251
mmu-mir-106b	3,756	5,579	3,705	5,639	5,510	4,370	3,968	8,715	2,694
MCE-MIR_3416:rev	3,577	2,064	2,691	9,039	11,804	14,417	8,123	11,362	8,546
MCE-MIR_1506:rev	3,570	2,919	4,638	7,724	13,606	11,685	12,909	10,773	4,381
MCE-MIR_3485:rev	3,569	3,053	2,605	9,321	8,434	12,110	10,219	9,116	3,861
mmu-mir-291b-5p	3,527	7,567	3,190	7,496	1,211	984	1,176	307	120
mmu-mir-191	3,517	6,528	3,626	3,570	3,322	2,597	3,629	7,257	1,734
MCE-MIR_4945:fwd	3,424	503	1,651	6,182	11,279	11,796	5,029	8,246	6,282
MIR207	3,411	4,917	2,782	7,634	9,022	14,471	10,588	9,898	6,040
MCE-MIR_5403:fwd	3,386	4,868	3,172	8,872	7,225	12,538	10,900	7,795	485
cand718:b	3,375	896	1,846	5,357	12,367	9,660	8,081	12,633	4,713
mmu-mir-130b	3,269	3,579	1,922	4,340	2,895	2,599	1,386	8,038	3,279
cand231	3,185	1,292		5,269	13,609	10,905	8,679	11,998	9,412
MCE-MIR_3847:fwd	3,161	5,320	3,249	8,626	8,897	8,528	10,553	3,230	614
cand613	3,160	2,905	3,925	7,708	9,766	12,564	7,005	9,270	5,259
MCE-MIR_4297:fwd	2,986	3,952	2,365	8,709	9,771	13,804	9,414	11,890	6,217
cand79	2,972	2,087	2,872	5,179	14,308	11,752	7,995	7,483	2,890
MCE-MIR_3334:fwd	2,927	2,171	2,727	7,688	7,885	10,390	10,311	8,646	1,165
mmu-mir-103	2,904	4,529	3,307	4,077	5,190	4,741	3,647	10,206	3,845
mmu-mir-23a	2,893	6,214	3,862	3,672	3,594	2,579	5,215	5,434	1,051
MCE-MIR_4743:fwd	2,806	5,425	1,973	10,658	5,434	12,048	7,555	6,950	1,112
mmu-mir-15b	2,785	5,245	3,046	4,631	4,650	3,089	6,000	6,741	1,363
mmu-mir-23b	2,780	5,928	3,723	3,215	4,023	2,692	5,285	9,546	3,265

ES Time:	Day 0		Day 1		Day 3			Day 6	
**Array Chip ID:**	**210**	**357**	**124**	**216**	**217**	**222**	**356**	**222**	**245**
MCE-MIR_3490:rev	2,751	927	3,083	6,419	11,159	10,710	8,109	8,067	5,157
mmu-mir-302b	2,751	10,251	2,941	4,779	24		91	215	22
cand564:b	2,668	3,516	1,700	3,069	4,740	3,915	3,826	9,651	2,419
cand324:a	2,655	4,271	3,881	4,164	1,172	1,680	2,052	1,351	151
mmu-mir-379	2,652	4,278	2,792	3,457	5,874	3,820	4,849	11,601	3,523
MCE-MIR_3470:rev	2,647	1,644	1,609	5,214	7,993	9,759	6,941	8,649	5,902
mmu-mir-291a-5p	2,625	5,939	2,148	5,846	604	454	691	119	23
MCE-MIR_1792:rev	2,617	6,974	2,068	10,646	8,821	11,117	18,483	9,006	856
mmu-mir-24	2,609	4,305	2,963	2,595	3,856	2,776	2,523	6,392	2,811
cand178:a	2,593	4,778	2,489	12,626	8,126	9,066	16,415	6,566	430
MCE-MIR_1365:fwd	2,588	478	1,601	4,200	11,942	11,360	4,942	8,161	9,726
S-mmu-mir-293	2,569	804		7,031	268	118			
MCE-MIR_3609:rev	2,550	926	1,736	4,240	5,486	6,719	5,942	4,157	1,329
S-mmu-mir-21	2,537	669		4,146	12,922	10,026	13,205	5,087	1,534
S-mmu-mir-365-2	2,532	4,698		9,855	9,737	18,109	8,704	7,071	2,933
cand708:b	2,512	4,297	6,148	8,492	7,629	16,084	7,749	7,510	2,192
MCE-MIR_5192:rev	2,512	2,355	2,443	7,642	10,513	11,339	9,703	7,635	2,773
MCE-MIR_5389:fwd	2,429	4,009	2,500	6,395	5,981	6,940	7,757	2,194	410
MCE-MIR_5030:rev	2,407	4,013	726	10,012	7,350	10,868	10,197	6,876	401
mmu-mir-494	2,398	680	446	3,368	2,632	1,819	4,062	1,707	122
S-mmu-mir-150	2,379	2,606		5,938	7,476	14,664	6,237	7,559	7,525
mmu-mir-30c	2,347	5,922	4,326	2,266	4,850	3,745	6,041	10,267	2,720
S-mmu-mir-326	2,298	6,589		10,113	5,981	12,959	10,882	8,150	1,201
cand116	2,290	1,879	2,855	8,795	8,690	10,128	9,190	8,760	2,253
MIR165	2,281	1,391	2,347	5,538	7,945	9,236	5,784	7,485	4,797
S-mmu-mir-295	2,279	2,148		4,092	918	955	405	244	51
MCE-MIR_3642:rev	2,272	2,431	1,516	4,734	4,152	7,592	5,989	5,179	1,103
MCE-MIR_3529:rev	2,254	6,049	744	4,297	8,886	8,926	15,113	11,021	710
mmu-mir-134	2,249	2,456	1,598	2,432	6,732	5,750	5,477	8,318	4,475
cand523	2,223	3,494	3,426	9,234	4,512	12,825	8,681	11,541	907
MCE-MIR_6054:rev	2,175	1,161	452	2,087	2,450	1,886	2,821	2,921	893
MCE-MIR_4462:fwd	2,135	1,067	2,227	5,079	8,356	9,981	5,690	7,978	5,281
MCE-MIR_1778:fwd	2,098	926	1,235	4,927	9,094	9,648	6,527	10,232	1,631
cand91:a	2,094	288	494	2,788	14,153	9,091	6,772	6,395	3,521
S-mmu-mir-296	2,083	2,055		4,320	3,558	5,763	4,500	7,127	1,536
cand156	2,053	8,005	4,407	13,992	1,045	4,184	14,237	2,406	132
MCE-MIR_3573:rev	2,052	261	674	3,126	11,049	7,029	2,035	4,488	10,996
mmu-mir-107	2,012	3,436	2,170	3,239	3,697	3,594	2,885	7,473	2,321

ES Time:	Day 0		Day 1		Day 3			Day 6	
**Array Chip ID:**	**210**	**357**	**124**	**216**	**217**	**222**	**356**	**222**	**245**
MCE-MIR_3488:rev	2,010	941	1,270	4,614	7,199	8,443	7,801	5,020	2,905
MCE-MIR_5291:fwd	2,005	6,036	2,557	9,144	3,315	9,493	11,795	5,340	212
MCE-MIR_5440:fwd	2,003	3,479	1,633	6,874	5,104	8,230	9,836	5,103	434
mmu-mir-7	1,999	5,219	2,810	3,264	1,261	982	2,328	636	114
MCE-MIR_5079:rev	1,986	904	977	4,858	8,089	9,972	3,880	7,018	6,065
MCE-MIR_3134:rev	1,948	7,151	1,070	5,054	8,316	8,178	14,820	5,340	255
cand279:a	1,924	792	784	4,332	8,251	6,848	5,435	3,204	2,137
mmu-mir-200b	1,917	4,187	2,829	3,002	2,769	2,259	3,335	3,065	875
MCE-MIR_5152:rev	1,899	2,464	1,752	4,983	6,118	7,861	8,135	4,001	347
MCE-MIR_2139:fwd	1,844	752	1,195	4,161	8,403	11,427	2,089	7,173	8,937
MCE-MIR_3513:rev	1,834	2,927	3,450	6,702	7,099	5,753	8,132	2,029	593
cand465:a	1,822	6,049	2,508	4,091	53	50	172	219	27
MCE-MIR_1371:fwd	1,817	882	1,646	3,877	10,532	8,730	6,450	4,950	1,791
cand362	1,807	1,515	898	5,331	4,331	8,494	8,474	7,998	354
MCE-MIR_3762:rev	1,791	780	3,056	5,165	10,088	14,121	3,806	4,308	5,234
MCE-MIR_2327:fwd	1,765	443	834	6,571	11,360	9,895	2,676	5,783	3,014
cand686	1,727	821	1,080	1,167	3,808	3,102	2,065	4,314	2,935
MCE-MIR_5399:fwd	1,701	1,166	957	3,941	5,796	8,683	3,063	7,040	3,876
cand284:a	1,699	1,966	1,908	1,580	3,080	2,641	1,845	4,943	1,268
MCE-MIR_2817:fwd	1,662	517	1,804	2,037	10,440	8,222	4,388	8,621	3,541
MCE-MIR_4273:fwd	1,641	843	816	3,693	9,999	12,727	9,827	8,968	6,399
MCE-MIR_1734:fwd	1,624	1,646	2,050	3,383	7,243	7,933	8,171	4,708	508
MCE-MIR_1325:rev	1,622	905	1,803	4,266	7,161	8,037	5,306	6,037	1,350
MCE-MIR_451:rev	1,621	671	580	2,428	2,391	2,796	935	1,245	110
mmu-mir-376b	1,560	3,126	2,010	3,582	4,231	3,348	2,605	3,318	707
MCE-MIR_4684:fwd	1,552	678	801	4,132	5,747	7,751	4,508	3,663	1,449
MCE-MIR_3143:fwd	1,543	1,474	1,235	4,753	5,024	7,328	5,829	4,415	1,205
cand425	1,543	1,176	1,201	4,214	2,697	7,993	3,212	5,339	923
MCE-MIR_1784:fwd	1,542	5,187	1,734	7,672	6,251	7,295	9,735	2,754	152
MIR30	1,526	5,833	1,753	5,077	1,235	1,249	4,819	1,719	199
MCE-MIR_5057:fwd	1,500	2,061	554	9,008	4,426	8,039	6,547	4,776	190
MCE-MIR_3484:rev	1,460	856	1,499	4,503	6,498	7,385	5,551	5,531	3,958
MCE-MIR_2698:rev	1,455	407	966	2,690	6,423	8,849	2,628	7,233	5,700
S-mmu-mir-294	1,454	3,032		3,823	237	133	266	32	
MCE-MIR_755:fwd	1,446	305	395	3,791	7,063	8,725	2,911	5,630	6,427
mmu-mir-382	1,437	2,641	1,279	2,068	4,336	2,880	4,884	10,329	2,708
MCE-MIR_3685:rev	1,432	1,042	312	3,368	4,490	6,197	2,963	3,182	619
mmu-mir-210	1,421	1,837	982	3,476	547	549	247	2,419	846

ES Time:	Day 0		Day 1		Day 3			Day 6	
**Array Chip ID:**	**210**	**357**	**124**	**216**	**217**	**222**	**356**	**222**	**245**
mmu-mir-99b	1,406	1,792	1,991	2,405	6,221	4,143	5,548	11,269	3,846
MCE-MIR_1352:fwd	1,403	908	2,159	3,823	10,950	13,128	3,486	3,585	4,629
cand268:b	1,385	2,011	1,216	1,818	834	634	687	1,125	303
cand345	1,337	1,075	1,126	4,882	1,885	7,015	4,692	3,044	126
cand268:a	1,302	74	401	1,780	15,208	7,172	5,248	12,050	4,838
S-mmu-mir-467	1,291	4,073		4,716	875	679	2,056	502	110
MCE-MIR_5014:fwd	1,290	971	185	1,236	1,130	665	1,931	1,578	202
MCE-MIR_4740:fwd	1,283	2,140	1,559	4,510	3,124	2,750	4,349	692	202
mmu-mir-466	1,282	5,479	3,117	5,302	2,032	1,786	2,591	1,219	203
MCE-MIR_3859:fwd	1,277	2,548	1,286	4,039	3,931	3,549	5,355	842	118
mmu-mir-18	1,252	1,672	637	2,384	1,599	1,246	929	1,284	307
mmu-mir-200c	1,242	2,556	1,491	1,655	1,482	1,100	1,941	2,818	851
MCE-MIR_4922:fwd	1,234	2,381	442	6,948	4,689	4,220	4,879	1,029	88
MCE-MIR_4999:rev	1,217	552	237	2,065	4,292	3,750	1,697	2,169	2,190
mmu-mir-125a	1,208	2,413	2,257	2,331	4,394	3,626	5,277	13,248	5,302
cand617	1,193	2,036	1,240	4,127	2,177	6,121	4,755	3,092	301
cand135:b	1,181	843	942	3,399	4,159	3,454	1,896	4,731	1,233
mmu-mir-361	1,153	2,363	1,681	1,950	2,374	1,926	2,217	3,750	1,422
mmu-mir-19a	1,149	688	43	4,385	776	621	74	372	60
mmu-mir-409	1,147	3,595	1,010	2,228	2,880	2,997	6,698	5,086	724
mmu-mir-302	1,146	4,504	1,464	2,416	16		25	21	
MCE-MIR_1986:fwd	1,137	711	644	4,841	7,151	6,792	5,472	3,194	639
MCE-MIR_273:fwd	1,136	448	1,711	3,485	6,770	7,135	5,520	5,818	1,920
MIR74	1,130	1,460	1,015	2,398	2,518	1,691	1,859	6,218	1,712
MCE-MIR_3429:rev	1,121	334	950	1,723	7,850	7,370	3,945	3,881	2,208
MCE-MIR_2099:fwd	1,071	765	1,221	4,231	4,828	6,670	1,938	3,272	461
cand144:a	1,055	1,320	559	2,965	799	1,062	467	1,751	241
mmu-mir-467	1,028	3,621	2,561	3,718	1,235	1,546	1,551	1,339	200
mmu-mir-27b	1,021	2,683	1,211	1,778	1,532	994	1,408	4,252	991
MCE-MIR_5607:rev	997	580	402	6,811	6,276	4,168	3,514	1,201	43
MCE-MIR_1536:rev	997	568	1,023	4,315	7,856	6,827	3,903	2,508	2,240
cand374:b	980	621	807	3,906	8,304	7,135	4,471	5,195	2,354
cand314	947	1,564	1,165	1,260	1,769	1,738	1,729	3,560	909
cand179:a	945	2,233	3,434	5,587	3,640	12,570	4,307	3,453	444
cand137:a	939	1,086	1,150	1,466	2,049	1,823	867	3,323	693
MCE-MIR_2977:rev	933	694	67	4,046	4,599	4,085	5,355	2,291	463
mmu-mir-433-3p	918	1,469	749	1,385	2,262	2,046	3,130	4,966	1,109
S-mmu-mir-140	901	709	855	1,860	1,607	1,315	761	2,519	838

ES Time:	Day 0		Day 1		Day 3			Day 6	
**Array Chip ID:**	**210**	**357**	**124**	**216**	**217**	**222**	**356**	**222**	**245**
S-mmu-mir-151	901	1,453		1,272	1,348	1,005	1,308	1,885	629
MIR85	900	702	314	637	421	375	154	2,165	638
cand699	892	335	827	1,898	5,083	5,891	2,678	3,965	2,038
MCE-MIR_5141:rev	873	1,520	330	7,420	4,119	8,376	3,848	3,410	275
MIR124	872	1,568	570	2,831	2,624	2,016	1,628	3,540	682
cand315:b	864	1,197	1,130	1,322	1,391	1,256	1,577	2,579	800
MCE-MIR_3408:fwd	848	551	243	3,047	2,947	3,953	2,477	2,381	1,159
cand103:b	845	2,647	1,719	1,759	1,091	1,360	1,807	4,635	726
MIR206	843	165	506	1,235	9,073	5,447	3,730	4,169	1,778
MCE-MIR_3780:rev	835	1,211	460	2,863	3,287	2,847	5,266	5,698	1,105
MCE-MIR_4913:rev	835	4,260	377	6,455	1,611	7,088	6,392	3,781	66
S-mmu-mir-30c-1	830	4,518		4,889	894	5,751	8,358	2,530	67
mmu-mir-434-3p	811	1,818	792	1,751	2,267	1,915	3,269	4,481	1,057
MCE-MIR_3523:rev	789	385	447	1,578	3,715	4,688	1,842	2,831	2,024
cand588	766	260	198	2,478	8,027	4,600	1,929	3,090	1,888
MCE-MIR_2714:rev	761	404	1,135	2,736	6,140	6,211	6,080	4,287	356
cand418	759	1,165	593	4,278	2,035	4,288	2,805	1,792	524
mmu-mir-34a	742	876	455	1,601	1,287	1,100	657	1,122	206
MCE-MIR_298:rev	717	543	488	2,638	6,948	5,961	6,041	2,194	625
mmu-mir-195	703	2,263	794	1,566	737	507	1,232	307	70
MCE-MIR_530:fwd	696	4,936	2,016	7,260	4,494	11,839	9,416	2,589	736
S-mmu-mir-337	672	765		1,092	1,387	1,152	813	2,104	557
MCE-MIR_1514:fwd	668	759	334	2,848	2,392	3,377	2,102	1,208	174
MIR202	654	1,253	353	1,474	1,416	1,478	1,302	1,553	317
mmu-mir-298	651	1,564	471	2,357	1,347	2,091	2,042	2,761	355
cand515	647	256	158	1,427	3,759	4,278	963	2,028	1,113
cand467	626	895	280	3,721	1,383	2,257	2,092	1,194	43
cand104:a	626	1,822	1,039	1,481	765	756	849	2,497	415
S-mmu-mir-106b	617	800		1,256	756	718	740	1,298	288
MCE-MIR_5354:rev	615	1,411	94	3,191	2,368	2,298	2,682	1,227	456
MCE-MIR_4627:fwd	596	715	272	3,510	2,622	4,655	5,781	1,758	147
mmu-mir-185	576	1,156	629	1,073	960	999	1,064	1,396	260
MCE-MIR_5470:fwd	576	1,480	210	2,813	991	1,258	2,702	341	41
mmu-mir-543	571	1,240	331	1,234	1,419	1,371	1,140	1,258	328
MCE-MIR_3685:fwd	569	1,624	430	5,942	6,446	5,707	8,530	3,180	170
MCE-MIR_2680:rev	553	523	10	495	268	149	593	106	44
mmu-mir-101b	550	580	178	1,217	544	495	172	457	94
MIR253	549	1,078	224	1,443	677	551	646	629	145

ES Time:	Day 0		Day 1		Day 3			Day 6	
**Array Chip ID:**	**210**	**357**	**124**	**216**	**217**	**222**	**356**	**222**	**245**
mmu-mir-26b	545	1,829	1,156	1,145	2,942	2,167	3,624	8,356	2,478
MCE-MIR_5443:fwd	541	463	70	1,632	5,485	4,564	1,372	2,682	2,831
cand545	539	577	210	3,092	3,888	8,117	2,219	3,542	317
cand557	521	294	333	678	1,560	2,157	903	1,390	971
MCE-MIR_534:fwd	507	1,305		2,580	816	2,836	9,241	1,535	35
mmu-mir-329	505	1,370	361	1,254	1,475	1,428	1,171	4,098	814
MCE-MIR_3531:rev	503	275	562	2,135	3,846	4,030	3,667	2,941	1,237
cand350	500	836	949	1,318	945	698	844	2,248	292
mmu-mir-124a	496	461	124	456	233	223	99	1,229	342
MCE-MIR_5056:rev	490	241	85	912	2,269	1,572	712	404	250
MCE-MIR_5384:rev	484	780	421	2,500	2,140	3,968	3,973	1,835	64
S-mmu-mir-204	481	762		2,058	1,288	2,156	6,542	606	77
S-mmu-mir-378	474	411		594	357	349	181	1,012	436
MCE-MIR_670:fwd	470	723	128	2,233	894	2,683	2,552	1,531	65
cand276:a	467	441	358	959	623	607	357	658	112
cand126	464	913	341	3,850	537	3,975	3,257	2,418	82
cand457:a	451	436	296	2,086	402	1,971	1,020	887	82
mmu-mir-30b	449	2,336	1,121	1,351	2,247	1,656	3,257	4,957	720
mmu-mir-181b	443	982	425	1,372	4,042	2,942	3,616	11,012	3,975
S-mmu-mir-483	441	414		974	874	2,071	1,120	1,978	1,478
MCE-MIR_4714:rev	441	619	70	6,471	5,906	5,398	4,244	1,170	49
cand692:a	440	220	306	1,603	4,143	4,113	1,206	1,800	1,167
MIR166	436	193	139	880	2,061	2,258	1,271	1,443	624
MCE-MIR_6026:rev	431	593	149	2,109	789	1,810	3,041	477	
MCE-MIR_1857:fwd	420	173	248	1,462	2,611	2,730	4,338	701	22
MCE-MIR_2745:fwd	417	490	184	3,430	2,052	5,509	1,199	2,783	135
cand73	417	264	37	1,195	810	1,773	531	315	140
MCE-MIR_4027:rev	413	268	285	911	1,650	2,412	755	1,215	991
cand563	410	2,392	370	4,790	484	2,943	2,153	1,037	40
cand572:a	405	182	137	1,447	1,655	3,956	684	1,691	223
mmu-mir-342	395	1,002	483	1,037	1,315	1,236	2,164	3,178	712
cand317	382	370	265	1,322	464	634	340	441	93
MCE-MIR_3260:rev	381	339		2,710	3,966	2,662	1,507	1,459	1,373
cand24	381	279	254	804	1,107	3,409	596	841	563
MCE-MIR_1973:fwd	380	1,337	169	3,877	810	4,297	7,087	1,730	64
mmu-mir-429	379	1,030	824	1,317	1,575	1,236	1,214	1,158	285
MCE-MIR_4674:fwd	378	341	170	1,176	5,551	5,273	8,084	2,445	703
mmu-mir-148a	377	875	631	1,122	661	506	502	664	131

ES Time:	Day 0		Day 1		Day 3			Day 6	
**Array Chip ID:**	**210**	**357**	**124**	**216**	**217**	**222**	**356**	**222**	**245**
mmu-mir-431	375	586	216	814	1,683	1,207	992	2,395	525
mmu-mir-30d	375	1,098	337	713	927	724	1,231	1,518	236
S-mmu-mir-425	374	768		994	269	262	531	339	64
cand371:b	374	430	196	966	820	885	733	990	302
MCE-MIR_4791:fwd	372	993	252	3,517	2,701	3,446	2,322	850	168
MCE-MIR_4661:rev	364	1,703	301	6,436	417	6,610	3,121	1,914	65
MCE-MIR_5068:rev	348	121		642	4,068	4,003	666	2,372	4,615
mmu-mir-324-3p	346	399	217	759	724	622	425	978	254
mmu-mir-324-5p	344	376	282	779	1,144	1,029	600	2,609	703
MCE-MIR_6107:fwd	341	399	200	1,086	767	1,574	1,037	714	65
MCE-MIR_5914:rev	336	304	367	5,012	6,949	6,029	3,380	2,464	209
mmu-mir-151	325	542	298	515	492	399	532	712	196
cand78:a	320	252	196	857	54	34	24	14	
mmu-mir-205	316	795	132	739	264	107	311	285	38
MCE-MIR_5363:rev	310	108	70	951	2,523	3,974	274	2,159	1,612
MCE-MIR_3541:rev	304	162	56	1,802	1,689	3,038	864	881	379
MCE-MIR_5055:rev	303	136	166	796	3,101	3,052	850	805	226
MCE-MIR_3628:rev	296	127	131	324	2,441	1,538	2,745	1,218	297
mmu-mir-27a	294	1,525	482	1,440	834	606	635	1,167	152
mmu-mir-422b	293	483	142	499	292	364	190	1,060	339
cand42:a	287	196	237	2,233	2,804	5,813	1,183	2,866	296
mmu-mir-30a-5p	286	740	311	802	856	656	648	1,216	141
mmu-mir-17-3p	283	285	98	608	316	282	163	485	106
MCE-MIR_5369:rev	282	140	45	2,051	1,363	2,242	684	633	128
MCE-MIR_4320:rev	280	546	191	1,982	1,300	3,645	5,585	2,061	42
mmu-mir-363	280	1,108	438	1,274	334	166	494	33	
mmu-mir-370	280	504	166	538	1,587	1,328	1,439	1,748	529
mmu-mir-145	278	392	226	411	389	398	505	1,523	126
MCE-MIR_1364:fwd	274	289	113	1,184	656	1,379	1,457	982	29
MCE-MIR_5366:rev	271	188	74	525	1,217	2,391	560	820	861
cand690	271	965	631	2,583	1,238	3,328	18,746	1,729	42
mmu-mir-484	268	739	129	764	414	360	859	488	94
MCE-MIR_5643:fwd	263	881	61	1,382	488	2,001	2,592	854	69
mmu-mir-540	260	369	120	332	405	375	425	500	110
MCE-MIR_5276:fwd	260	274	396	917	2,568	4,206	1,501	614	78
MCE-MIR_2470:fwd	258	153	102	839	797	1,624	608	993	115
MCE-MIR_5088:rev	256	659	24	1,478	1,205	2,887	826	634	271
cand315:a	255	445	275	536	449	451	629	728	184

ES Time:	Day 0		Day 1		Day 3			Day 6	
**Array Chip ID:**	**210**	**357**	**124**	**216**	**217**	**222**	**356**	**222**	**245**
MCE-MIR_5641:rev	246	397	34	1,142	508	1,103	827	777	79
S-mmu-mir-128b	241	2,643		4,810	437	5,299	5,373	1,459	26
MCE-MIR_1544:rev	240	61	131	996	3,253	2,364	998	786	225
mmu-mir-152	238	516	446	758	910	754	777	2,739	1,085
cand210:a	237	730	204	790	546	744	661	749	87
cand412	235	1,265	135	1,799	752	1,235	3,993	575	104
MCE-MIR_5083:rev	233	322	36	1,228	1,127	1,099	1,398	445	128
MCE-MIR_4610:fwd	233	239	131	2,191	4,719	4,523	9,310	875	33
cand337	232	222	182	479	300	336	259	800	139
cand374:a	231	523	338	313	3,074	2,631	4,367	18,338	8,198
MCE-MIR_1074:rev	230	747	95	1,201	1,832	3,228	5,183	598	20
cand445	230	348	205	3,307	1,307	4,112	1,103	1,369	101
cand152:b	228	940	321	587	269	361	734	640	99
mmu-mir-31	226	263	133	518	304	266	122	218	55
cand304	226	244	81	425	179	193	111	659	174
S-mmu-mir-25	225	313		521	218	272	450	213	40
MCE-MIR_638:fwd	225	229	68	1,066	996	1,258	576	466	70
mmu-mir-22	223	602	78	680	459	335	316	210	74
mmu-mir-150	219	501	172	436	28	22	79	10	
MCE-MIR_2986:fwd	215	564	48	1,446	1,927	2,264	5,228	601	22
cand97	213	303	86	677	154	187	114	192	37
MCE-MIR_2679:fwd	209	102	17	724	3,808	3,389	387	1,787	3,275
mmu-mir-301	206	237	63	1,815	514	454	113	636	120
mmu-mir-181a	205	217	130	300	2,271	1,873	1,879	10,309	4,096
mmu-mir-154	205	333	71	413	572	383	583	803	175
mmu-mir-28	200	518	148	455	568	454	791	777	140
mmu-mir-337	200	285	157	801	855	662	493	730	150
cand618	191	114		928	3,998	3,666	1,479	1,847	1,184
MCE-MIR_4755:fwd	191	306	51	1,810	826	1,158	970	329	30
cand278:a	189	283	301	468	897	754	1,015	5,962	1,540
mmu-mir-346	187	127	90	310	569	738	274	480	80
MCE-MIR_5745:fwd	186	138	140	656	1,747	2,601	2,234	878	20
MCE-MIR_5216:rev	185	274	48	1,384	2,495	3,866	2,859	1,557	251
MCE-MIR_543:fwd	183	254	33	813	430	1,173	1,469	501	214
MCE-MIR_3557:rev	183	254	124	628	1,465	3,095	837	491	416
MCE-MIR_4010:rev	182	241	57	525	625	1,108	1,011	360	128
cand595:a	181	76	116	1,517	2,690	2,628	948	440	41
mmu-let-7c	180	537	365	176	503	649	611	12,790	3,478

ES Time:	Day 0		Day 1		Day 3			Day 6	
**Array Chip ID:**	**210**	**357**	**124**	**216**	**217**	**222**	**356**	**222**	**245**
mmu-mir-335	180	660	159	1,013	7,879	5,248	5,755	11,552	1,895
cand462	177	2,279	72	2,662	472	1,001	5,182	416	44
mmu-mir-143	177	270	78	213	212	205	137	588	131
cand336	175	198	120	445	403	344	233	624	139
S-mmu-mir-363	173	656		624	233	141	423	73	
MCE-MIR_3624:rev	173	111	92	311	890	1,032	473	766	486
MCE-MIR_1408:fwd	173	1,218	42	3,192	658	1,779	4,346	708	22
S-mmu-mir-23a	173	251		681	404	625	522	371	65
MCE-MIR_1066:rev	168	172	48	366	298	542	918	290	21
cand1:b	164	290	111	751	343	444	188	261	55
cand118	164	410	296	208	398	706	659	9,747	2,337
MCE-MIR_5411:rev	162	1,277	30	2,600	314	2,046	6,821	1,090	31
MCE-MIR_3572:rev	162	162	33	788	1,265	1,699	344	732	983
cand497:a	160	262	124	315	598	765	638	368	83
mmu-mir-15a	159	605	203	896	943	733	706	552	109
MCE-MIR_4198:fwd	159	208	45	994	700	699	1,119	184	48
S-mmu-mir-92-2	159	876		1,503	562	2,144	6,476	860	22
mmu-mir-485-3p	158	364	76	442	297	287	463	198	40
S-mmu-mir-183	157	336		417	115	87	235	60	23
MCE-MIR_2889:fwd	155	221	110	378	396	962	499	586	78
MCE-MIR_2866:rev	154	364	18	1,146	1,825	1,690	1,347	867	416
MCE-MIR_2563:fwd	153	958	68	207	666	573	1,540	3,005	458
S-mmu-mir-329	153	151		419	292	272	206	365	59
mmu-mir-222	152	265	107	366	337	283	270	601	149
cand532:a	151	50	24	134	1,904	1,376	298	879	648
MCE-MIR_3958:fwd	150	125	42	746	1,897	3,214	368	1,433	3,975
MCE-MIR_1611:fwd	149	111	52	594	825	903	1,318	422	69
mmu-mir-126-3p	145	309	146	171	941	715	863	221	68
cand297:b	143	281	122	278	341	301	264	174	43
MCE-MIR_5473:fwd	141	229	27	372	132	253	1,229	209	30
MCE-MIR_2983:rev	141	107	51	193	199	439	152	335	46
cand532:b	137	429	31	2,307	1,932	1,477	2,160	329	63
cand459	134	81	55	863	1,860	1,972	1,097	538	394
mmu-mir-376a	134	126	11	192	249	220	118	731	177
MCE-MIR_1746:rev	134	158		152	140	85	205	485	41
cand42:b	132	138	24	254	409	446	226	282	160
MCE-MIR_2680:fwd	132	282	12	1,999	1,285	2,404	1,613	752	48
cand161	132	281	233	146	271	529	435	7,742	1,708

ES Time:	Day 0		Day 1		Day 3			Day 6	
**Array Chip ID:**	**210**	**357**	**124**	**216**	**217**	**222**	**356**	**222**	**245**
mmu-mir-296	131	345		288	147	200	261	197	30
S-mmu-mir-210	130	262		406	132	246	285	164	54
mmu-mir-410	129	360	121	626	1,228	990	792	1,380	221
MCE-MIR_4922:rev	129	598	16	2,684	308	450	980	200	24
MCE-MIR_3653:rev	127	271		858	329	1,190	387	217	39
S-mmu-let-7d	126	228	33	106	121	236	170	207	20
MCE-MIR_3886:fwd	126	50	44	185	305	509	133	267	33
cand347	126	82	36	376	1,096	1,389	440	797	264
MCE-MIR_5363:fwd	124	132	100	1,397	578	2,744	728	892	22
cand342:b	123	212	170	1,445	1,109	2,555	2,146	1,059	60
mmu-mir-155	122	215	200	405	605	366	578	711	392
MCE-MIR_2902:rev	122	113	31	590	329	999	502	670	73
mmu-mir-221	121	283	82	327	273	182	323	554	165
mmu-mir-101a	120	156	19	608	119	65	31	54	
MCE-MIR_406:rev	120	234		1,380	161	764	730	214	
MCE-MIR_936:rev	118	123	37	450	395	502	505	410	93
S-mmu-mir-379	118	307		644	588	284	995	480	37
mmu-mir-487b	117	278	116	318	526	452	563	861	173
MCE-MIR_2968:fwd	117	55	34	241	216	402	228	220	42
S-mmu-mir-24-2	117	154		303	145	70	124	101	33
MCE-MIR_4726:fwd	117	686	10	798	340	607	4,969	231	55
MCE-MIR_2815:fwd	114	243	45	654	700	677	1,010	319	75
MCE-MIR_6050:rev	113	175		1,037	1,545	969	735	379	154
MCE-MIR_3439:rev	110	124		582	472	368	562	141	33
MCE-MIR_5511:rev	109	466	15	2,265	147	1,160	1,109	346	
S-mmu-mir-34c	106	99		172	186	220	196	307	42
cand501	105	97	23	238	226	237	98	183	109
mmu-mir-297	105	339	110	1,287	109	57	94	28	
MCE-MIR_3847:rev	104	68	41	203	300	422	108	227	42
mmu-mir-29a	104	311	214	234	246	137	161	90	24
MCE-MIR_3492:fwd	104	209	15	468	809	1,282	2,783	311	48
mmu-mir-125b	102	308	70	151	2,383	2,048	3,594	16,148	5,333
MIR257	102	745	151	582	371	170	1,073	755	76
S-mmu-mir-93	101	128		170	126	92	95	165	53
MCE-MIR_4030:rev	100	308	27	1,372	660	718	3,625	272	22
MCE-MIR_5473:rev	100	166	18	251	187	202	164	185	85
MCE-MIR_1283:fwd	100	136	14	265	288	284	141	196	88
cand109:b	100	102	46	420	266	520	316	253	62

ES Time:	Day 0		Day 1		Day 3			Day 6	
**Array Chip ID:**	**210**	**357**	**124**	**216**	**217**	**222**	**356**	**222**	**245**
MCE-MIR_3477:rev	100		167	388	3,243	2,623	1,896	1,735	778
cand624	99	76	18	458	504	343	75	198	69
MCE-MIR_988:fwd	99	116	11	179	201	146	53	137	100
MCE-MIR_725:fwd	98	101		211	265	233	96	197	120
mmu-mir-149	97	178	90	303	280	183	294	285	45
MCE-MIR_5004:rev	97	81	20	413	768	791	294	232	125
MCE-MIR_2078:fwd	95	43	64	177	170	165	91	152	38
mmu-mir-323	92	254	67	351	372	273	476	282	44
MIR121	92	246	84	211	140	87	277	115	40
MCE-MIR_5295:rev	91	95		209	156	165	132	113	71
cand149:b	91	452	69	1,646	1,085	2,588	2,516	522	29
MCE-MIR_1756:rev	90	99	24	125	126	226	161	189	24
mmu-mir-344	89	121	68	210	279	230	198	871	204
mmu-mir-129-5p	87	109	21	165	137	196	193	169	25
S-mmu-mir-431	87	94		200	719	937	400	312	175
MIR4	86	126	99	173	38	19	44	62	
MCE-MIR_1569:rev	85	84	43	327	388	420	1,100	166	30
MCE-MIR_3663:fwd	85	69	27	121	223	230	163	227	46
S-mmu-mir-302c	85	156	36	161	24	38	25	58	
mmu-mir-485-5p	85	179	55	120	156	174	263	236	63
mmu-mir-299	85	340	48	389	195	149	356	241	28
cand334:b	84			208	181	182	110	92	41
MCE-MIR_4031:fwd	84	84	35	111	110	223	127	275	32
mmu-mir-302c	83	1,171	47	908			20	16	
mmu-mir-434-5p	83	177	25	208	293	230	252	237	63
MCE-MIR_5503:rev	82	1,030		2,269	542	360	4,558	50	
cand678	82		36	310	483	378	126	162	48
MCE-MIR_5339:rev	82	51	26	298	280	257	117	121	24
mmu-mir-224	81	99	49	174	242	129	304	694	88
cand346	81	241	45	258	242	275	361	654	83
MCE-MIR_5488:rev	80	95		130	98	105	93	116	59
cand334:a	79	53	13	584	757	599	455	108	50
MCE-MIR_557:fwd	77	121		200	161	119	118	108	70
S-mmu-mir-7b	77	53		82	92	171	65	155	36
MCE-MIR_3613:rev	76	92	20	260	401	332	349	216	110
MCE-MIR_5454:rev	76	125		213	162	148	143	104	104
mmu-let-7a	76	400	209	126	356	556	566	12,869	2,248
S-mmu-mir-290	75	699		217	25	20	119		

ES Time:	Day 0		Day 1		Day 3			Day 6	
**Array Chip ID:**	**210**	**357**	**124**	**216**	**217**	**222**	**356**	**222**	**245**
S-mmu-mir-28	75	163		189	294	197	346	507	120
mmu-let-7e	75	202	129	126	377	577	648	6,989	897
MCE-MIR_2345:fwd	75	963		1,605	466	1,552	4,243	485	
cand5	75	48	18	153	837	287	146	177	413
cand386:a	74	83		85	124	165	117	152	28
MCE-MIR_5322:fwd	73	96		224	158	144	104	109	82
MCE-MIR_5596:rev	73	120	42	233	155	278	307	79	18
S-mmu-mir-342	73	144		178	228	220	243	711	266
S-mmu-mir-18	72	155		154	94	56	106	63	
mmu-mir-200a	72	154	65	525	101	59	65	27	
cand213	72	81	54	128	183	150	151	308	55
cand490	72	198	154	118	182	427	345	7,215	977
MCE-MIR_1046:rev	72	133	28	234	346	510	537	344	56
MCE-MIR_469:fwd	71	118	33	178	147	264	250	205	29
MCE-MIR_5872:fwd	71	70	36	546	331	951	378	296	47
mmu-mir-377	70	65		111	51	27	909	62	
mmu-mir-345	70	191	42	199	226	188	195	310	70
MCE-MIR_2349:rev	70	208	25	165	119	173	265	158	22
MCE-MIR_5055:fwd	70	130	19	225	1,026	970	352	182	109
mmu-mir-223	69	153		130	161	168	83	129	58
mmu-mir-381	69	187	48	401	262	174	215	294	31
S-mmu-mir-34a	67	86		152	92	81	67	67	40
MCE-MIR_2361:fwd	67	1,063		2,017	122	1,160	1,312	367	
MCE-MIR_4763:fwd	67	194	10	182	141	127	96	96	67
cand541	67	40	22	296	780	537	1,009	280	99
MIR43	67	100	25	271	47	26	21	23	
MCE-MIR_3791:rev	67	184	12	706	746	643	1,773	113	42
cand709	66	167	135	100	147	346	324	6,720	988
mmu-mir-351	65	95	40	142	895	808	867	4,024	1,195
MCE-MIR_6084:rev	65		23	95	121	238	33	113	19
MCE-MIR_5197:rev	63	38	18	180	349	223	116	196	41
MCE-MIR_4345:rev	63	176		511	166	295	432	145	
MCE-MIR_2524:fwd	63	45	37	75	199	366	84	235	55
MCE-MIR_1974:fwd	63	379		696	57	422	3,193	175	
cand26	63	45	28	154	193	182	163	168	81
cand203	62	123	27	326	185	189	206	155	43
mmu-mir-148b	62	168	71	283	140	86	159	165	28
cand153:a	61	99	12	277	105	143	141	79	44

ES Time:	Day 0		Day 1		Day 3			Day 6	
**Array Chip ID:**	**210**	**357**	**124**	**216**	**217**	**222**	**356**	**222**	**245**
MCE-MIR_3444:rev	61		15	64	86	125	88	81	19
MCE-MIR_1788:rev	60	229		184	67	127	326	203	19
MCE-MIR_1773:rev	60	90	20	67	78	156	124	97	29
MCE-MIR_1793:fwd	59	73	13	451	197	192	427	74	19
cand65	59	70		236	668	662	150	231	217
mmu-mir-96	59	170	14	657	36		31		
MCE-MIR_4493:fwd	59	58	23	61	35	27	40	17	
S-mmu-mir-127	58			158	162	158	86	236	92
S-mmu-mir-196b	58	67		35	59	82	39	105	20
MCE-MIR_4791:rev	58	144	38	345	140	79	147	48	
mmu-mir-300	58	159	48	351	228	177	173	264	27
MCE-MIR_3518:rev	58	75		480	773	1,248	417	314	755
cand244	57	68	31	809	138	1,731	351	400	23
MCE-MIR_3441:fwd	57	106		304	216	224	669	114	19
MCE-MIR_734:fwd	57	79	16	63	62	107	42	115	22
S-mmu-mir-1-1	57			64	87	148	28	95	21
MCE-MIR_5300:fwd	56	59		112	95	89	78	81	48
MCE-MIR_4714:fwd	56	71		107	110	100	79	85	59
MCE-MIR_5790:fwd	56	140	13	1,347	1,091	1,182	1,211	267	37
mmu-mir-330	56	81	34	99	104	78	121	197	42
mmu-mir-425	56	88	13	131	105	195	54	126	53
S-mmu-mir-412	56	65		107	381	313	360	413	148
mmu-mir-186	55	85	26	327	242	136	94	212	32
MCE-MIR_1342:fwd	54	169	108	225	60	426	112	84	35
MCE-MIR_5135:fwd	54	79		145	78	121	220	80	
MCE-MIR_2617:fwd	52	98	12	59	46	72	121	84	
S-mmu-mir-154	52	62		454	334	126	53	33	
cand57	51	107	31	521	369	3,866	446	555	112
S-mmu-mir-23b	51	81		69	51	30	47	46	
MCE-MIR_4712:fwd	51	116		110	116	114	56	72	71
MCE-MIR_1015:rev	51	52	36	95	207	183	140	143	60
MCE-MIR_5210:fwd	51			128	86	85	68	57	57
mmu-mir-539	50	104	18	83	161	93	254	246	89
MCE-MIR_4748:rev	50	65	13	65	78	97	65	94	38
MCE-MIR_6055:rev	49	95		235	119	147	192	106	
mmu-mir-328	49	198	31	223	210	296	475	421	47
MCE-MIR_4491:rev	49			87	38	77	24	105	
S-mmu-mir-194-2	49	148		466	95	167	411	49	

ES Time:	Day 0		Day 1		Day 3			Day 6	
**Array Chip ID:**	**210**	**357**	**124**	**216**	**217**	**222**	**356**	**222**	**245**
mmu-mir-214	48	152	24	237	2,006	1,496	1,998	10,152	2,336
MCE-MIR_2092:fwd	48	76	25	203	201	314	245	128	31
MCE-MIR_5389:rev	46	43	19	110	109	169	101	91	34
mmu-mir-218	46	182	43	233	459	310	797	5,163	801
S-mmu-mir-99b	46	82		176	366	187	274	276	141
MCE-MIR_4752:rev	46	119		111	153	112	52	67	68
MCE-MIR_2285:fwd	46	158		388	382	310	1,408	51	19
S-mmu-mir-7-2	46	66		84	101	144	80	79	26
cand103:a	45	46	24	63	51	34	52	50	
cand106:b	45	70	17	39	34	67	46	81	
MCE-MIR_5418:fwd	45	977		794	160	456	5,405	206	22
cand210:b	44	51	37	85	202	200	268	213	38
cand570	44	138	26	968	201	795	792	331	20
MCE-MIR_5193:rev	44			101	197	116	83	74	25
MCE-MIR_463:fwd	44	72		93	80	86	68	64	36
MCE-MIR_3820:fwd	44	69	15	119	122	149	71	87	50
MCE-MIR_5152:fwd	44	76	15	54	42	104	129	75	22
mmu-let-7d	43	182	59	89	103	220	264	8,185	674
cand212	43	104	55	189	223	200	303	398	68
MCE-MIR_780:rev	43	138		158	81	184	594	52	
cand564:a	43		12	78	161	118	64	219	81
MCE-MIR_4513:fwd	43	42	12	117	144	160	165	137	50
S-mmu-mir-345	42	47		163	175	191	66	347	75
cand465:b	42	63	22	83					
cand119	42	121	86	101	104	285	234	6,299	896
S-mmu-mir-377	42	37		60	49	30	51	42	
S-mmu-mir-125a	41	72		114	187	150	196	292	76
mmu-mir-184	41	95	50	91	116	110	118	158	41
MCE-MIR_5340:fwd	41	58		80	93	62	1,111	96	
S-mmu-mir-16-1	41	64		92	76	38	39	22	
MCE-MIR_3261:fwd	41	46		104	143	142	73	90	67
MCE-MIR_329:fwd	40		34	261	329	635	469	292	49
MCE-MIR_4716:fwd	39	74		159	241	329	369	199	25
cand707	39	46	28	96	53	62	112	87	
cand284:b	39	38	30	277	497	356	300	168	20
S-mmu-mir-187	38	58		92	89	123	58	71	18
MCE-MIR_5736:rev	38	75		77	64	92	99	64	
MCE-MIR_3684:rev	38	77		118	108	34	160	60	26

ES Time:	Day 0		Day 1		Day 3			Day 6	
**Array Chip ID:**	**210**	**357**	**124**	**216**	**217**	**222**	**356**	**222**	**245**
mmu-mir-470	38	88	36	95	134	89	190	324	71
MCE-MIR_3478:rev	38			75	248	381	103	216	176
mmu-mir-181c	37	72		119	130	76	229	548	42
cand457:b	37	85	15	87	54	104	258	110	
S-mmu-mir-184	37			39	52	100	29	69	18
S-mmu-mir-361	37	101		58	51	25	102	51	23
mmu-mir-129-3p	37	89	10	100	52	44	93	41	
cand1:a	37	50	33	145	103	61	66	65	
MCE-MIR_959:rev	37	54	11	66	64	126	38	59	
mmu-mir-380-3p	37	114	36	185	504	337	401	724	65
MCE-MIR_5406:rev	37		17	56	54	99	38	55	24
MCE-MIR_3101:rev	36	106	13	130	121	139	314	90	30
MCE-MIR_942:rev	36	72	14	144	129	203	360	82	39
cand115:b	36	890		950	151	375	3,035	78	29
MCE-MIR_4756:rev	36	100		90	73	79	52	43	59
S-mmu-mir-465	36	104		129	76	49	194	200	39
MCE-MIR_1508:fwd	36			372	474	554	169	202	50
S-mmu-mir-208	36	120		377	146	205	216	42	18
MCE-MIR_18:fwd	36	42		166	78	159	159	76	
mmu-let-7b	36	147	94	81	116	225	175	8,244	1,204
cand192	36	40		204	483	369	533	104	56
MCE-MIR_4153:fwd	36			155	2,080	1,455	376	349	26
cand106:a	36	126	56	137	66	206	422	4,941	403
MCE-MIR_4202:rev	36	34		80	107	56	107	50	
cand286	36			132	729	294	279	166	138
S-mmu-mir-193	36	56		96	99	92	148	177	35
MCE-MIR_5100:rev	36	48		89	251	123	194	80	32
MCE-MIR_2196:fwd	36	66		279	193	55	285	22	21
cand616:b	35		19	122	135	175	117	86	19
S-mmu-mir-200a	35	44		54	38		52	16	
MCE-MIR_2111:fwd	35	155	24	264	62	84	246	30	
MCE-MIR_5597:fwd	34	96	15	67	57	107	59	77	20
MIR102	34	51	19	187	154	137	204	171	28
cand172:a	34		13	45	65	62	50	44	
mmu-mir-188	34	48	11	92	205	247	93	208	123
mmu-mir-128a	34	121	26	92	78	42	150	93	23
mmu-mir-331	33	49	20	113	181	124	152	173	22
cand386:b	33	117		291	26	106	152	64	

ES Time:	Day 0		Day 1		Day 3			Day 6	
**Array Chip ID:**	**210**	**357**	**124**	**216**	**217**	**222**	**356**	**222**	**245**
S-mmu-mir-199b	33	56		77	773	515	799	9,882	2,319
MCE-MIR_5328:rev	33	620	13	537	266	440	4,873	136	
S-mmu-mir-384	32							15	
S-mmu-mir-34b	32	56		141	142	121	133	154	25
MCE-MIR_777:rev	32	78		158	65	334	733	37	
MCE-MIR_1829:rev	32	62		77	39		88		
S-mmu-mir-323	31	178		123	56	32	383	25	
MCE-MIR_399:fwd	31	37		71	71	45	74	25	
MCE-MIR_2134:fwd	31	54		94	98	78	220	62	23
S-mmu-mir-27a	31	56		92	51	23	55	17	
mmu-mir-187	30	74	24	115	125	99	116	116	25
MCE-MIR_2464:fwd	30	41		101	94	68	121	61	
MCE-MIR_4087:rev	29	46		112	125	77	211	38	
MCE-MIR_1576:rev	29	31		41	73	57	106	75	
MCE-MIR_2304:rev	29	40		23	48	61		36	
MCE-MIR_3667:fwd	29	51		52	52	55	126	65	
cand104:b	29	43	23	44	54	46	46	80	20
cand667:b	29		18	108	306	189	188	72	25
mmu-mir-207	29	95		96	78	129	146	83	24
MCE-MIR_1742:rev	28			117	263	56	2,130	33	
S-mmu-mir-218-2	28	52		36	35	48		86	20
cand500:a	28	42	17	117	264	214	115	114	35
MCE-MIR_3797:fwd	28	81		71	101	45	232	45	
MCE-MIR_1458:rev	27	50		109	488	408	122	151	35
MCE-MIR_1931:fwd	27	64		75	84	44	188	46	19
S-mmu-mir-30c-2	27	108		88	49	26	145	30	
MCE-MIR_1478:fwd	27			66	158	128	118	99	27
cand340	27	39		61	83	164	110	89	53
MCE-MIR_2566:fwd	27	41		146	125	75	93	59	19
MCE-MIR_4932:rev	27	68		30	29				
cand135:a	27		23	181	53	45		37	
MCE-MIR_5143:rev	27	54		26	45	84		41	
MCE-MIR_1495:fwd	27			47	46	79	28	88	
mmu-mir-412	27	53	17	56	122	148	213	129	24
MCE-MIR_3407:rev	26			98	87	112	127	85	
mmu-mir-450	26	53	12	139	79	54	133	765	53
MCE-MIR_3474:rev	26	36		190	587	482	447	234	31
S-mmu-mir-134	26	47		66	131	97	122	93	28

ES Time:	Day 0		Day 1		Day 3			Day 6	
**Array Chip ID:**	**210**	**357**	**124**	**216**	**217**	**222**	**356**	**222**	**245**
mmu-mir-350	26	113	42	313	146	84	143	118	18
cand275	26	72	26	211	134	257	332	95	20
MIR103	26	43		39	87	66	36	58	44
MCE-MIR_3624:fwd	26	48	14	26	35	42	27	63	22
S-mmu-mir-200b	26	45		92	83	64	87	27	
MCE-MIR_2364:rev	26	98	10	46	32	50	67	35	
MCE-MIR_4383:rev	26			81	61		88	21	
MCE-MIR_4726:rev	26	95		82	66	60	96	40	28
MCE-MIR_5033:rev	25			36	25		27	11	
cand718:a	25	32		28	45	62	37	41	
S-mmu-mir-124a-1	25			31	28	34	16	37	17
cand6	25	39		43	67	64	58	54	23
MCE-MIR_4745:fwd	25	45	9	33	31	53	41	25	
MCE-MIR_2661:rev	25	52		102	66	74	109	50	31
mmu-mir-133a	25	69		32	36	27	90	29	
S-mmu-mir-382	25	36		122	138	81	69	178	46
MCE-MIR_4675:fwd	25	46		118	57	43	176	36	
MCE-MIR_3695:fwd	25	41	10	38	23		32		
S-mmu-mir-181c	25	30		83	25	19	18	34	
S-mmu-mir-22	25	86		116	94	43	111	33	21
MCE-MIR_3471:rev	24			57	125	75	107	99	74
MCE-MIR_2474:rev	24	71		54	39	45	100	34	
MCE-MIR_4239:rev	24	34		65	86	46	137	42	
cand157	24	80	30	49	43	61	57	974	147
mmu-mir-146	24	74	14	42	177	100	274	59	29
mmu-mir-503	24	58		65	252	241	180	763	195
MCE-MIR_293:fwd	24	50		83	120	89	174	44	
MCE-MIR_4711:fwd	24			21	21	31		64	
mmu-mir-211	24			21	53	67	26	46	
mmu-mir-486	24	45		62	49	59	83	72	28
MCE-MIR_871:fwd	23			18		30		33	
mmu-mir-133b	23	55		37	41	43	96	38	
S-mmu-mir-370	23	40		61	147	111	155	120	27
S-mmu-mir-92-1	23	51		44	39	34	51	23	
MCE-MIR_4738:fwd	23	45		38	51	31	84		
cand705	23	67	14	29	47	67	201	48	20
mmu-mir-34c	22			92	65	52	23	70	
MIR75	22	86	16	189	1,850	1,283	1,569	10,056	1,847

ES Time:	Day 0		Day 1		Day 3			Day 6	
**Array Chip ID:**	**210**	**357**	**124**	**216**	**217**	**222**	**356**	**222**	**245**
S-mmu-mir-103-1	22	31		45	42	60	24	47	
mmu-mir-128b	22	112	18	78	62	33	120	72	19
MCE-MIR_4179:rev	22			27	246	51	48	56	
S-mmu-mir-351	22			49	273	244	165	474	148
MCE-MIR_3646:rev	22	32		40	106	56	65	31	
cand13	22	49		149	71	123	75	38	17
S-mmu-mir-125b-1	22	27		55	91	99	145	313	92
cand276:b	22	30	16	79	19			18	
cand664	22			53	45	49	146	32	
S-mmu-mir-15a	22	52		28	51	55	17	34	18
cand90:a	21	45	123	139	2,130	1,840	2,590	23,785	4,592
MCE-MIR_3886:rev	21			48	28	20	51	13	
MCE-MIR_5008:rev	21	55		29	25		43		
mmu-let-7g	21	73	34	60	25	27	57	1,443	164
cand146	20	36	15	46	160	92	72	61	73
cand572:b	20		18	37	35	28	42	17	
MCE-MIR_4762:fwd	20	51		14			35		
cand224	20			66	79	48	54	21	
cand619	20			81	131	90	126	62	19
S-mmu-mir-30b	20	82		36	46	20	112		
MCE-MIR_4497:rev	19	115		104	30	38	304	41	
S-mmu-mir-300	19	64		29	92	30	150	52	33
mmu-mir-30a-3p	19	99	18	89	81	51	125	165	29
MIR41	19	38	11	73	76	22	43	25	
S-mmu-mir-124a-3	19	34		33	31	44	23	42	
cand149:a	19	53		36	40	37	74	64	
S-mmu-mir-218-1	18	32		24	22	51	16	80	19
cand529	18			60	200	115	92	57	27
MCE-MIR_3793:rev	18			120	205	51	145	74	
MCE-MIR_4022:fwd	18			48	83	47	82	28	20
MCE-MIR_4554:rev	18	82		37	34	22	61	22	
S-mmu-mir-135a-1	18			24	18	30	25	46	
cand349:a	18	54		47	58	46	113	137	20
mmu-mir-362	18		18	71	90	62	46	106	28
MCE-MIR_3059:rev	18			26	45	60		36	
cand415	18			17	45	41	31	51	25
mmu-mir-339	18		10	70	37	25	33	30	
MCE-MIR_1283:rev	18	178		380	46	136	603	43	

**ES Time:**	**Day 0**		**Day 1**		**Day 3**			**Day 6**	
**Array Chip ID:**	**210**	**357**	**124**	**216**	**217**	**222**	**356**	**222**	**245**
cand302:b	18			36	124	61	45	34	22
cand137:b	17	50		79	21	33	35	55	
cand152:a	17	43		35	43	32	125	60	34
MCE-MIR_809:fwd	17			30	23	23	41		
MCE-MIR_2192:rev	17	50		30	15		51		
MCE-MIR_2894:fwd	17			34	40	18	63	24	
mmu-mir-30e	17	60	10	270	89	36	24	38	
S-mmu-mir-27b	17	50		33	32		49	33	
MCE-MIR_4236:rev	17			39	57		95		
MCE-MIR_4034:rev	17			42	54	40	109	40	20
MCE-MIR_2166:rev	17	49		39	26		58	24	
cand309	17		14	15	51	44	23	27	21
cand351	17	100		75	33	28	175	76	
MCE-MIR_1697:fwd	17			39	38	31		13	
MCE-MIR_4069:rev	17			19	41	26	46	29	
mmu-mir-468	17			21	27	17	20	38	
mmu-mir-132	17	35	12	55	34	44	82	332	37
cand262	17	29		84	12			49	
S-mmu-mir-486	17	58		253	22	89	131	23	
S-mmu-mir-188	17	37		29		24	37		
MCE-MIR_2501:fwd	17			35	64	59	61	79	23
MCE-MIR_3226:fwd	17	49		63	56	43	85	44	18
cand184	16	39		42	30	41		77	
S-mmu-mir-124a-2	16			25	32	41	18	42	17
mmu-mir-142-5p	16			34				19	
MCE-MIR_5180:rev	16			22	24	19	26		
MCE-MIR_4893:fwd	16	37		21	31	27	30	28	
MCE-MIR_5105:rev	16	39		20	47	18	57	22	
MCE-MIR_407:fwd	16				14	22		22	
S-mmu-mir-135a-2	16			24	180	145	148	400	84
MCE-MIR_1059:fwd	16			43	99	31	53	23	
mmu-let-7f	16	105	41	78	65	137	232	5,769	549
MCE-MIR_364:fwd	16	37		55	236	52	157	29	23
MCE-MIR_5236:rev	16			30	21	24	20	16	
MCE-MIR_3113:fwd	16			35	30	29	37	23	
mmu-mir-203	16	38	11	35	54	44	85	141	36
mmu-mir-193	16	37		31	20	26	27	41	
S-mmu-mir-219-2	16	34		56	1,195	895	1,090	1,398	317

ES Time:	Day 0		Day 1		Day 3			Day 6	
**Array Chip ID:**	**210**	**357**	**124**	**216**	**217**	**222**	**356**	**222**	**245**
mmu-mir-452	15			31	37	20	52	23	
S-mmu-mir-10b	15			25	178	219	74	1,079	319
mmu-mir-212	15	67	11	29	46	35	53	130	34
S-mmu-let-7i	15			17	26	30		26	
MCE-MIR_1689:rev	15			27	23	21	31	10	
mmu-mir-546	15	123		329	179	119	247	27	
cand200	15	4,459	19	1,585	695	1,492	11,473	211	82
S-mmu-mir-196a-2	15				20	23			
MCE-MIR_1192:rev	14				12		20	30	
mmu-mir-483	14	39		22	28	39	51	25	
MCE-MIR_1788:fwd	14	64		44	22	31	60	14	
S-mmu-let-7g	14			17	32	38	17	27	
mmu-mir-380-5p	14	38		33	75	33	78	67	
cand100:b	14			19					
cand35	14	41		95	32	53	76	33	
MCE-MIR_1356:fwd	14	41		49	95	44	86	26	19
S-mmu-mir-7-1	14			58	37	31	41	21	
MCE-MIR_1811:rev	14			26	24	30	27	20	
mmu-mir-194	13	52	16	68	27	17	42	22	
cand91:b	13			66	44	59	94	44	
MCE-MIR_993:rev	13			22	65	31	23	32	51
MCE-MIR_1412:fwd	13			13	18	17			
mmu-let-7i	13	51		35	28	28	33	1,400	277
S-mmu-mir-381	13			46			24		
MCE-MIR_1226:fwd	13			49			41		
MCE-MIR_4015:fwd	13		15		11	79		51	23
MCE-MIR_5122:rev	13	42		21	18	19	33		
MCE-MIR_162:fwd	13			29	73	37	68	17	18
MCE-MIR_3888:rev	13	44		54	58	47	90	45	
MCE-MIR_6026:fwd	13	48		17	15	18	42	27	
mmu-mir-199a	13	44	13	53	666	433	609	9,409	1,899
S-mmu-mir-409	13	55		104	94	55	154	92	25
cand64	13			27	143	56	55	39	28
MIR141	13	50		64	131	109	129	347	71
cand120	13	43		23	22	26	30	63	
MCE-MIR_6001:rev	13			28	44	30	76		
S-mmu-mir-106a	12			31	12			18	
cand348	12	53	28	62		61	63	2,439	111

ES Time:	Day 0		Day 1		Day 3			Day 6	
**Array Chip ID:**	**210**	**357**	**124**	**216**	**217**	**222**	**356**	**222**	**245**
S-mmu-mir-195	12				16				
MCE-MIR_89:fwd	12			31	37	26	55	17	
MCE-MIR_4063:rev	12			11	18				
mmu-mir-376c	12	43		88	40		50	39	
S-mmu-mir-107	11					21		22	
S-mmu-mir-138-2	11	46		14	17		38		
S-mmu-mir-20	11			26	20				
mmu-mir-489	11			15	15	17		28	
S-mmu-mir-139	11	32		28		22	45		
cand226	10			29	34	31	56	44	
MCE-MIR_1670:fwd	10			46	56	48	403	28	
MCE-MIR_3754:rev	10			25	21		41	13	
MCE-MIR_2722:rev	10			17	30	28	30	32	
mmu-mir-378	10	46		20	14			24	
cand90:b	10		28	66	200	216	703	2,813	293
MCE-MIR_2711:rev	9			25	27	45	54	37	
mmu-mir-122a	9			25	19	27	33	1,155	192
S-mmu-mir-181a	9	36			50	64	63	83	27
S-mmu-let-7a-2	8			11				18	
S-mmu-mir-137	8						14		
MCE-MIR_3429:fwd	8	75		14	17		111	26	
S-mmu-let-7a-1	7			14					
S-mmu-mir-125b-2	6			10	27	17	25	51	25
cand11:a									
cand129				9					
cand130									
cand139									
cand144:b				21	11				
cand153:b				18					
cand178:b				33	22	21	40	29	
cand179:b							30		
cand202									
cand22									
cand234									
cand25									
cand27				11	93	107	254	414	43
cand278:b		32		13			42	16	
cand297:a								10	

ES Time:	Day 0		Day 1		Day 3			Day 6	
**Array Chip ID:**	**210**	**357**	**124**	**216**	**217**	**222**	**356**	**222**	**245**
cand302:a				59	22	43	49		
cand324:b				63	18		23	17	
cand349:b									
cand352:a									
cand352:b					17			25	
cand361					19	19	24		
cand375			23	83	5,492	3,493	8,689	22,023	4,785
cand40								12	
cand427							45		
cand492		467		93		26	1,120		
cand497:b				17					
cand50									
cand500:b				15	18	20	33	24	
cand524									
cand549						17			
cand585					20				
cand590									
cand614				13					
cand650				18	22	20	26	24	
cand669				45	51	27	32	24	
cand7		584		166	52	29	1,129		
cand70				39	57	39	211	23	
cand708:a				16			47		
cand82									
MCE-MIR_1052:rev					26	19	23	22	
MCE-MIR_1259:rev									
MCE-MIR_1264:rev				7					
MCE-MIR_1269:rev									
MCE-MIR_1311:rev				13	35		67		
MCE-MIR_1356:rev				13	16		35		
MCE-MIR_1365:rev		36		19	29	34	53		
MCE-MIR_1401:fwd									
MCE-MIR_1433:fwd				15	36	21		18	
MCE-MIR_1433:rev					16			11	
MCE-MIR_1442:fwd									
MCE-MIR_1482:rev				18	19	42	30	19	
MCE-MIR_151:fwd				10	13	19	26		
MCE-MIR_1546:rev				19	35		32		

ES Time:	Day 0		Day 1		Day 3			Day 6	
**Array Chip ID:**	**210**	**357**	**124**	**216**	**217**	**222**	**356**	**222**	**245**
MCE-MIR_1642:rev				11	19				
MCE-MIR_1645:rev				20	15		19		
MCE-MIR_1679:fwd					15				
MCE-MIR_1710:rev									
MCE-MIR_1786:fwd				16					
MCE-MIR_188:fwd									
MCE-MIR_1905:rev									
MCE-MIR_1929:fwd									
MCE-MIR_1998:fwd				10		21			
MCE-MIR_2087:rev									
MCE-MIR_2166:fwd		40		11					
MCE-MIR_2173:rev		50		33	39		45		
MCE-MIR_2197:fwd									
MCE-MIR_2198:fwd									
MCE-MIR_2205:fwd									
MCE-MIR_2288:fwd									
MCE-MIR_2339:fwd		49			20		31		
MCE-MIR_2339:rev							23		
MCE-MIR_2371:fwd									
MCE-MIR_2371:rev									
MCE-MIR_2417:fwd									
MCE-MIR_2419:fwd									
MCE-MIR_2522:rev									
MCE-MIR_2566:rev		32				20	20	13	
MCE-MIR_2624:rev				11					
MCE-MIR_2691:rev									
MCE-MIR_2798:fwd									
MCE-MIR_281:rev									
MCE-MIR_2902:fwd		60				19	54		
MCE-MIR_291:rev									
MCE-MIR_293:rev				12		48		19	
MCE-MIR_3007:rev									
MCE-MIR_3032:fwd									
MCE-MIR_3048:rev									
MCE-MIR_3057:fwd				12	16		37		
MCE-MIR_3057:rev									
MCE-MIR_3084:fwd							37		
MCE-MIR_3330:fwd					18				

ES Time:	Day 0		Day 1		Day 3			Day 6	
**Array Chip ID:**	**210**	**357**	**124**	**216**	**217**	**222**	**356**	**222**	**245**
MCE-MIR_3333:rev				22	17	25	36	42	
MCE-MIR_334:rev		49		28	16	29	60	35	
MCE-MIR_3379:fwd								10	
MCE-MIR_3513:fwd				9	13		54		
MCE-MIR_3522:rev				12	21		30		
MCE-MIR_3550:fwd		31		11	14		31		
MCE-MIR_3619:rev							24		
MCE-MIR_3637:rev									
MCE-MIR_3651:fwd					16				
MCE-MIR_3667:rev				14	12		28		
MCE-MIR_3715:rev									
MCE-MIR_3832:rev									
MCE-MIR_3841:fwd				14	22		38		
MCE-MIR_3859:rev				12	43	33	50	29	
MCE-MIR_3867:rev									
MCE-MIR_405:rev									
MCE-MIR_4060:rev									
MCE-MIR_4061:rev					18				
MCE-MIR_4063:fwd									
MCE-MIR_4124:fwd									
MCE-MIR_4179:fwd									
MCE-MIR_4182:rev									
MCE-MIR_4207:fwd									
MCE-MIR_4209:fwd									
MCE-MIR_4232:fwd									
MCE-MIR_4280:fwd				21	24		39		
MCE-MIR_4280:rev		35		10			26	14	
MCE-MIR_4303:fwd									
MCE-MIR_4342:fwd									
MCE-MIR_4353:rev				10	19		23		
MCE-MIR_4413:fwd									
MCE-MIR_4442:fwd				27	19		35		
MCE-MIR_4449:rev									
MCE-MIR_4472:fwd				13	10				
MCE-MIR_4474:fwd									
MCE-MIR_4491:fwd									
MCE-MIR_4503:rev									
MCE-MIR_4521:fwd									

ES Time:	Day 0		Day 1		Day 3			Day 6	
**Array Chip ID:**	**210**	**357**	**124**	**216**	**217**	**222**	**356**	**222**	**245**
MCE-MIR_4521:rev				13	15			27	
MCE-MIR_4592:fwd									
MCE-MIR_4607:fwd									
MCE-MIR_4614:rev		35							
MCE-MIR_466:fwd		31							
MCE-MIR_4667:fwd		42		18			23		
MCE-MIR_4712:rev		33		10	11				
MCE-MIR_4789:rev									
MCE-MIR_4799:rev					41	45	36	1,633	
MCE-MIR_4809:fwd				21					
MCE-MIR_482:fwd									
MCE-MIR_482:rev									
MCE-MIR_4830:fwd				12	21		38	10	
MCE-MIR_4832:rev					11				
MCE-MIR_4853:fwd		33							
MCE-MIR_4893:rev		35							
MCE-MIR_4972:fwd									
MCE-MIR_4978:rev									
MCE-MIR_504:fwd									
MCE-MIR_5046:rev		28							
MCE-MIR_5167:fwd									
MCE-MIR_5167:rev				30	42		47		
MCE-MIR_5180:fwd									
MCE-MIR_5260:fwd									
MCE-MIR_5279:fwd									
MCE-MIR_5287:rev						18			
MCE-MIR_5300:rev									
MCE-MIR_5303:fwd				16			25		
MCE-MIR_5322:rev		38		31	15		59		
MCE-MIR_5374:rev				24					
MCE-MIR_5396:rev				12	20	18			
MCE-MIR_5504:rev									
MCE-MIR_5581:fwd				12	14				
MCE-MIR_5581:rev				17	18				
MCE-MIR_5598:fwd									
MCE-MIR_5620:fwd		55					33	17	
MCE-MIR_5623:rev									
MCE-MIR_5704:rev									

ES Time:	Day 0		Day 1		Day 3			Day 6	
**Array Chip ID:**	**210**	**357**	**124**	**216**	**217**	**222**	**356**	**222**	**245**
MCE-MIR_5712:rev		29			15				
MCE-MIR_5864:fwd									
MCE-MIR_5970:rev				20	15		29	10	
MCE-MIR_6033:fwd				10	14		39		
MCE-MIR_6034:rev		52		12			26		
MCE-MIR_6120:fwd									
MCE-MIR_689:rev									
MCE-MIR_774:rev									
MCE-MIR_782:rev									
MCE-MIR_81:fwd									
MCE-MIR_855:fwd				25			25		
MCE-MIR_855:rev									
MCE-MIR_871:rev									
MCE-MIR_946:rev									
MIR100							16		
MIR12				64				43	
MIR122							27		
MIR140									
MIR161									
MIR167		42		14			30	10	
MIR169									
MIR177							36		
MIR180									
MIR184		39				20			
MIR188									
MIR201		33		18			64		
MIR220				10					
MIR237									
MIR255									
MIR35				15	1,460	1,086	1,581	7,265	1,980
MIR47				14	16		36	26	
MIR52								11	
MIR71									
MIR77				10	195	168	577	12,526	1,312
MIR79									
mmu-mir-1				21					
mmu-mir-100				23	905	745	1,730	7,242	1,061
mmu-mir-10a			152	292	11,376	6,618	15,601	33,132	10,077

ES Time:	Day 0		Day 1		Day 3			Day 6	
**Array Chip ID:**	**210**	**357**	**124**	**216**	**217**	**222**	**356**	**222**	**245**
mmu-mir-10b				24	1,577	1,022	8,603	19,740	3,656
mmu-mir-126-5p				9					
mmu-mir-135a									
mmu-mir-135b		33		18					
mmu-mir-136									
mmu-mir-137									
mmu-mir-138					34		30		
mmu-mir-139				14					
mmu-mir-140				26					
mmu-mir-141				15					
mmu-mir-142-3p				14					
mmu-mir-144									
mmu-mir-153									
mmu-mir-189									
mmu-mir-190									
mmu-mir-192				10					
mmu-mir-196a				15			32		
mmu-mir-196b									
mmu-mir-199a		35		32	97	86	59	729	113
mmu-mir-199b				20	27	24	20	227	32
mmu-mir-201									
mmu-mir-202				12					
mmu-mir-204				13	27	29		29	
mmu-mir-206				18	20		18	85	
mmu-mir-208									
mmu-mir-213								17	
mmu-mir-215									
mmu-mir-216					60	26	43	26	
mmu-mir-217					60	26	44	29	
mmu-mir-219					41	24		51	
mmu-mir-291b-3p									
mmu-mir-29b				52					
mmu-mir-29c		35		51	19		38		
S-mmu-mir-302b									
S-mmu-mir-30e				8	9		23		
mmu-mir-32									
mmu-mir-322				21	159	95	155	673	134
mmu-mir-325				15	24	17	27	22	

ES Time:	Day 0		Day 1		Day 3			Day 6	
**Array Chip ID:**	**210**	**357**	**124**	**216**	**217**	**222**	**356**	**222**	**245**
mmu-mir-326				14	17	20	33	13	
mmu-mir-33									
mmu-mir-338				10					
mmu-mir-340									
mmu-mir-34b				27					
mmu-mir-365		44		37	26		102	26	
mmu-mir-367									
mmu-mir-369-3p				9					
mmu-mir-369-5p				43	64	25	101	60	
mmu-mir-375				51	39		59		
S-mmu-mir-376a									
S-mmu-mir-376b				16	49		56	43	
mmu-mir-383		26		17	23		25	63	19
mmu-mir-384									
mmu-mir-411		69		24	28	26	154	25	
mmu-mir-424		43		73	515	412	706	3,439	479
mmu-mir-433-5p		41		26	40	24	60	36	
mmu-mir-448									
mmu-mir-449				14					
mmu-mir-451				31		16			
mmu-mir-463									
mmu-mir-464									
mmu-mir-465							21	8	
mmu-mir-469									
mmu-mir-471									
mmu-mir-542-3p				9	19		27	107	25
mmu-mir-542-5p				29	19	20	28	57	
mmu-mir-547									
mmu-mir-7b									
mmu-mir-9				35	386	256	2,189	11,875	656
S-mmu-mir-9				34	303	223	336	4,942	1,189
mmu-mir-98				18			27	371	
mmu-mir-99a				16	1,251	1,055	1,525	6,789	1,788
S-mmu-let-7b				13	13	28		32	
S-mmu-let-7c-1									
S-mmu-let-7c-2									
S-mmu-let-7e									
S-mmu-let-7f-1								12	

ES Time:	Day 0		Day 1		Day 3			Day 6	
**Array Chip ID:**	**210**	**357**	**124**	**216**	**217**	**222**	**356**	**222**	**245**
S-mmu-let-7f-2									
S-mmu-mir-100									
S-mmu-mir-101a									
S-mmu-mir-101b									
S-mmu-mir-103-2									
S-mmu-mir-10a				24	638	552	553	1,910	412
S-mmu-mir-1-2									
S-mmu-mir-122a									
S-mmu-mir-128a		33		29			63		
S-mmu-mir-129-1		62		61	16	34	54	15	
S-mmu-mir-130a									
S-mmu-mir-132									
S-mmu-mir-133a-1									
S-mmu-mir-133a-2									
S-mmu-mir-133b		1,393				1,206			393
S-mmu-mir-138-1				10					
S-mmu-mir-141				8			14		
S-mmu-mir-143				7					
S-mmu-mir-144									
S-mmu-mir-145		45		22	12		51	26	
S-mmu-mir-146									
S-mmu-mir-148a									
S-mmu-mir-148b									
S-mmu-mir-15b				8			18		
S-mmu-mir-16-2									
S-mmu-mir-182				10					
S-mmu-mir-185		40		19	16		26		
S-mmu-mir-186									
S-mmu-mir-190									
S-mmu-mir-191				7					
S-mmu-mir-192									
S-mmu-mir-194-1				14	16		22		
S-mmu-mir-196a-1					11				
S-mmu-mir-212				9	16		266	42	
S-mmu-mir-214								26	
S-mmu-mir-221				12					
S-mmu-mir-223									
S-mmu-mir-26a-1									

ES Time:	Day 0		Day 1		Day 3			Day 6	
**Array Chip ID:**	**210**	**357**	**124**	**216**	**217**	**222**	**356**	**222**	**245**
S-mmu-mir-298		57		18	14	25	20	14	
S-mmu-mir-29a									
S-mmu-mir-29b-1		34		12			30		
S-mmu-mir-29b-2		31		11	14		28		
S-mmu-mir-31									
S-mmu-mir-32				12					
S-mmu-mir-33									
S-mmu-mir-330				42	19		25	27	
S-mmu-mir-331				30	19	17		17	
S-mmu-mir-335									
S-mmu-mir-338									
S-mmu-mir-339				41	33				
S-mmu-mir-350				11	13			16	
S-mmu-mir-448									
S-mmu-mir-449		27		15					
S-mmu-mir-451									
S-mmu-mir-466				14					
S-mmu-mir-468									
S-mmu-mir-471				18			28		
S-mmu-mir-96							14		
S-mmu-mir-98									
S-mmu-mir-99a							22		

Supplemental Table ST4C: Mouse Array Version 2 data-GCNF-/-. This table contains the simple detectable values, which lists average signal values of all transcripts on the array, of the ES-GCNF -/- time series. Each array includes 545 MCE-MIR, 266 mmu-mir, 170 Cand, 46 MIR, and 177 S-mmu-mir. A total of 10 arrays were used in this study. This table represents results obtained with the <200 nt RNA probe from GCNF-/- ES cell line.

GCNF-/- Time:	Day 0		Day 1		Day 3		Day 6	
**Array Chip ID:**	**204**	**210**	**124**	**217**	**356**	**357**	**245**	**246**
MCE-MIR_2563:rev	33,190	29,754	46,336	51,688	79,720	44,841	42,587	101,912
mmu-mir-294	27,977	21,138	7,146	46,012	25,123	9,874	1,190	386
MCE-MIR_5060:rev	27,442	29,011	24,596	41,783	38,577	23,439	28,398	88,804
MCE-MIR_4453:fwd	26,129	25,897	26,457	37,363	53,330	33,549	30,034	74,833
cand706	24,686	26,552	21,761	40,348	49,020	25,318	24,668	79,735

GCNF-/- Time:	Day 0		Day 1		Day 3		Day 6	
**Array Chip ID:**	**204**	**210**	**124**	**217**	**356**	**357**	**245**	**246**
MCE-MIR_3820:rev	24,390	25,743	23,626	39,464	33,795	20,734	23,817	67,341
cand151	23,804	25,500	20,485	38,573	47,617	23,299	28,331	91,269
cand516	23,341	24,063	21,294	37,881	43,507	21,392	29,286	85,350
mmu-mir-292-3p	22,689	14,493	17,266	36,063	30,157	12,338	22,013	17,756
MCE-MIR_1597:fwd	21,710	23,518	17,754	40,011	34,681	18,145	24,089	74,257
MCE-MIR_1539:rev	20,571	20,068	18,520	34,543	32,501	19,650	16,672	32,560
mmu-mir-293	17,630	12,067	13,487	30,530	22,292	9,695	8,031	7,233
MCE-MIR_3571:rev	17,076	18,765	16,850	32,867	28,329	12,746	27,186	61,510
MCE-MIR_4822:rev	16,950	14,575	12,689	19,539	35,179	23,691	10,848	14,609
MCE-MIR_1773:fwd	15,755	15,518	16,100	29,407	31,042	16,596	21,729	45,838
cand385	15,722	16,035	16,874	29,972	30,449	15,798	29,739	64,217
MCE-MIR_1457:rev	15,717	16,362	15,278	25,873	27,735	15,689	11,984	18,342
MCE-MIR_1394:rev	15,657	19,876	8,664	26,466	24,777	13,171	5,653	10,012
MCE-MIR_3503:rev	15,122	13,894	12,740	20,510	12,915	7,188	15,372	23,984
MCE-MIR_4274:fwd	14,984	15,521	28,549	35,668	47,343	23,205	45,424	84,335
mmu-mir-290	14,766	11,153	10,893	29,731	17,092	8,908	7,499	4,871
cand271	14,137	16,296	13,596	26,906	37,328	12,590	19,360	37,764
MCE-MIR_5062:rev	13,520	13,498	13,609	24,820	20,420	9,990	19,137	34,286
mmu-mir-291a-3p	13,146	11,019	1,844	13,431	3,376	2,001	555	244
MCE-MIR_3191:rev	12,868	11,288	12,863	19,110	29,216	19,106	8,304	14,775
MCE-MIR_2222:fwd	12,863	11,457	7,744	15,172	11,030	8,279	5,717	3,753
MCE-MIR_1538:fwd	12,819	12,814	17,204	25,555	21,325	12,664	10,999	11,070
MCE-MIR_3468:rev	12,456	11,866	13,187	20,420	23,630	13,049	19,168	31,566
MCE-MIR_3190:rev	12,286	10,754	11,528	18,152	28,441	18,813	4,624	7,871
cand78:b	12,269	9,221	8,242	18,344	15,385	9,763	18,949	24,034
cand294	12,231	13,744	8,752	32,834	29,377	9,246	9,965	14,938
MCE-MIR_968:fwd	11,791	12,149	9,786	21,673	18,094	10,274	4,028	4,590
mmu-mir-295	11,704	8,174	6,508	20,792	10,250	4,879	5,106	2,560
cand594	11,676	11,600	12,036	21,202	20,232	8,891	25,279	32,447
MCE-MIR_3782:rev	11,671	10,491	11,150	24,876	20,780	9,803	22,603	43,740
MCE-MIR_5367:rev	11,529	10,074	10,191	22,170	15,238	8,460	17,146	26,084
mmu-mir-292-5p	11,526	7,902	9,716	20,711	14,739	7,571	4,119	2,300
MCE-MIR_3111:fwd	11,292	8,530	15,377	13,869	23,820	16,896	23,241	18,756
cand595:b	11,200	11,910	10,183	16,252	22,751	11,108	6,239	13,106
cand186	11,069	12,064	6,332	16,447	15,745	8,020	11,452	20,501
MCE-MIR_4861:fwd	10,946	9,932	8,193	15,919	20,935	11,553	3,314	4,356
MCE-MIR_984:rev	10,814	10,596	13,655	18,142	18,123	12,012	17,459	28,361
MCE-MIR_3491:rev	10,159	8,491	11,188	17,601	17,178	9,623	10,601	16,063

GCNF-/- Time:	Day 0		Day 1		Day 3		Day 6	
**Array Chip ID:**	**204**	**210**	**124**	**217**	**356**	**357**	**245**	**246**
MCE-MIR_254:fwd	10,023	9,504	11,374	18,413	20,214	12,249	13,572	17,254
MCE-MIR_645:fwd	9,980	10,234	7,418	16,522	17,953	8,228	2,502	3,629
MCE-MIR_995:rev	9,970	10,020	11,675	16,986	19,512	9,173	13,541	18,384
cand692:b	9,864	7,441	6,787	15,836	14,468	8,203	13,159	18,193
MCE-MIR_2164:rev	9,856	9,228	10,642	15,833	23,212	10,940	10,317	15,796
MIR112	9,750	8,273	8,064	15,420	14,249	7,252	10,614	15,034
mmu-mir-17-5p	9,472	9,701	9,825	21,638	8,625	6,044	2,652	2,580
MCE-MIR_2388:rev	9,296	9,268	8,766	12,542	16,830	10,119	7,529	10,558
cand489	9,232	7,821	10,780	20,060	15,306	8,049	19,211	31,677
MCE-MIR_3502:rev	9,203	8,966	15,860	15,767	24,884	12,273	23,487	28,783
MCE-MIR_5195:rev	8,986	7,966	7,994	16,557	20,957	8,495	11,432	16,886
cand255	8,874	7,405	5,504	10,963	11,289	7,585	7,873	7,374
mmu-mir-20a	8,722	8,936	7,744	19,621	7,881	4,994	1,873	1,876
MCE-MIR_3416:rev	8,719	6,406	4,465	14,477	9,420	4,137	6,855	9,557
MCE-MIR_3751:fwd	8,577	8,012	2,575	13,758	9,006	6,484	3,692	3,013
MCE-MIR_5699:fwd	8,546	8,702	8,354	8,196	14,187	9,157	1,124	774
MCE-MIR_321:rev	8,543	7,548	3,643	11,017	20,792	10,599	1,430	1,711
cand306	8,527	7,825	7,690	12,516	17,518	9,112	7,176	13,582
MCE-MIR_3155:fwd	8,506	5,806	13,619	13,044	22,973	15,415	20,180	18,701
MCE-MIR_3626:rev	8,475	8,377	26,975	16,869	32,048	16,088	47,938	85,391
MCE-MIR_5005:rev	8,472	7,109	9,759	15,452	16,096	10,880	5,519	6,425
MCE-MIR_2169:rev	8,467	6,980	3,331	10,051	11,068	6,525	1,173	1,703
MCE-MIR_4226:fwd	8,367	6,820	18,655	11,821	33,790	19,810	19,646	16,985
MCE-MIR_3495:rev	8,341	10,013	3,072	17,346	9,400	2,548	8,666	16,075
MCE-MIR_5374:fwd	8,278	10,716	5,181	19,967	11,310	5,835	1,386	2,851
MCE-MIR_1504:rev	8,209	6,744	6,918	12,668	10,947	6,698	4,011	4,424
MCE-MIR_1535:rev	8,205	7,033	8,099	17,262	13,858	6,967	16,686	26,097
MCE-MIR_2999:fwd	8,181	5,838	3,635	11,702	19,022	9,282	1,593	1,253
MCE-MIR_5606:fwd	8,179	6,998	8,575	12,609	11,361	8,052	4,889	4,459
MCE-MIR_2310:rev	8,150	7,081	4,049	11,993	11,846	6,908	456	334
MCE-MIR_1190:fwd	8,057	6,131	8,248	11,771	13,744	9,905	4,996	4,512
cand667:a	8,054	6,417	6,013	9,837	14,039	6,860	5,534	4,855
MCE-MIR_3147:fwd	8,029	5,756	6,842	12,534	12,545	7,020	11,220	13,407
MCE-MIR_4854:rev	7,915	5,916	2,377	6,321	9,956	5,790	104	70
cand648	7,889	6,142		5,501	16,878	11,772	540	309
MIR88	7,868	6,956	6,012	13,583	11,172	5,785	11,236	17,349
MCE-MIR_3295:rev	7,826	5,338	8,588	14,277	12,743	7,332	13,006	15,561
MCE-MIR_4625:fwd	7,811	6,483	8,546	11,460	14,308	9,094	5,251	4,855

GCNF-/- Time:	Day 0		Day 1		Day 3		Day 6	
Array Chip ID:	204	210	124	217	356	357	245	246
cand11:b	7,740	6,836	7,393	13,836	11,038	5,980	11,490	13,851
MCE-MIR_2337:fwd	7,726	5,976	6,464	9,633	9,383	5,573	1,726	836
MCE-MIR_4661:fwd	7,658	6,838	6,676	8,843	15,328	9,558	2,094	1,431
MCE-MIR_135:fwd	7,626	5,954	8,562	13,663	12,592	8,369	5,795	5,608
MCE-MIR_335:fwd	7,570	5,431	9,773	14,684	17,003	10,264	10,047	12,488
MCE-MIR_1038:fwd	7,538	6,771	6,733	15,292	8,726	5,749	10,162	16,143
MCE-MIR_3686:fwd	7,523	5,927	4,587	13,288	7,826	3,559	8,570	10,379
MCE-MIR_136:fwd	7,429	6,798	8,752	13,863	16,614	9,829	8,507	8,930
MCE-MIR_1409:fwd	7,329	5,705	9,977	14,906	16,117	7,990	14,245	17,653
MCE-MIR_3837:rev	7,287	5,234	4,821	7,505	15,060	9,396	1,083	618
MCE-MIR_783:rev	7,194	5,891	8,781	12,340	14,007	8,585	5,355	4,137
MCE-MIR_822:fwd	7,165	6,388	6,825	12,386	13,912	8,134	1,972	1,441
MCE-MIR_2243:fwd	7,154	5,520	4,987	11,368	10,737	4,601	8,705	12,578
MCE-MIR_4297:fwd	7,132	5,750	4,302	11,184	15,560	5,480	6,244	6,431
MCE-MIR_5089:fwd	7,113	5,247	5,987	8,841	13,676	9,191	3,585	1,417
MCE-MIR_4673:fwd	6,984	6,372	10,059	10,647	19,480	11,651	12,043	7,769
MIR216	6,982	6,874	6,126	14,885	5,491	3,512	885	730
cand371:a	6,921	5,749	5,391	14,854	13,054	5,111	16,281	24,192
mmu-mir-302d	6,884	4,581	8,427	8,427	2,675	1,260	31	
mmu-mir-20b	6,833	6,998	6,304	17,876	5,911	3,855	1,084	891
cand708:b	6,805	7,398	6,223	11,783	15,628	7,753	2,470	3,544
MCE-MIR_3485:rev	6,595	5,202	5,084	10,397	13,717	5,765	5,396	5,381
MCE-MIR_3490:rev	6,582	3,972	7,135	12,299	9,599	5,218	5,596	7,016
MCE-MIR_4821:rev	6,543	8,047	4,100	16,869	10,556	5,009	2,932	6,239
MCE-MIR_4087:fwd	6,498	4,960	5,745	13,984	9,384	5,076	6,744	5,714
MCE-MIR_5061:rev	6,477	5,012	3,489	12,170	9,617	3,589	7,574	11,658
MCE-MIR_4820:rev	6,460	7,727	4,169	16,789	11,512	5,209	3,469	7,328
MIR70	6,446	4,205	7,759	7,944	2,126	1,176	32	
MCE-MIR_1506:rev	6,442	4,644	7,576	11,397	13,439	7,971	4,365	4,431
mmu-mir-106a	6,379	6,805	7,054	14,771	6,508	4,227	1,203	1,033
MIR207	6,378	6,412	2,154	8,740	11,671	3,713	1,882	2,373
MCE-MIR_2953:fwd	6,361	5,948	23,366	12,590	35,695	18,505	34,249	29,053
MCE-MIR_5109:rev	6,246	6,032	6,389	14,503	11,764	5,443	11,791	17,875
cand68	6,081	3,692	18,128	14,314	22,753	9,408	44,578	68,946
MIR61	6,076	2,923	9,149	9,973	11,790	12,035	2,892	368
MCE-MIR_4945:fwd	5,933	4,099	2,762	11,485	5,541	1,765	8,249	9,681
cand345	5,931	3,791	2,418	2,920	4,454	2,957	121	83
MCE-MIR_2327:fwd	5,925	3,936	1,480	10,436	2,314	863	1,161	1,236

GCNF-/- Time:	Day 0		Day 1		Day 3		Day 6	
**Array Chip ID:**	**204**	**210**	**124**	**217**	**356**	**357**	**245**	**246**
MCE-MIR_3334:fwd	5,882	3,890	4,679	8,690	12,864	5,354	2,039	1,529
cand279:b	5,860	4,133	4,151	10,980	8,243	3,966	7,173	7,311
MCE-MIR_3573:rev	5,814	2,399	610	18,674	956	171	6,649	11,673
MCE-MIR_5403:fwd	5,778	4,527	4,710	7,269	12,717	6,867	988	555
MCE-MIR_3543:rev	5,606	5,001	14,351	8,207	31,025	15,261	6,538	4,245
MCE-MIR_2171:rev	5,597	4,991	18,670	12,428	26,067	11,620	44,270	78,086
MCE-MIR_5192:rev	5,529	3,373	4,249	8,463	10,057	5,648	4,156	4,692
cand647	5,525	3,452	3,670	6,616	2,550	2,026	2,208	913
cand178:a	5,490	2,565	4,295	5,641	9,898	12,427	930	703
MCE-MIR_1792:rev	5,454	3,288	2,816	5,114	14,633	11,331	442	315
MCE-MIR_5172:fwd	5,307	4,444	1,708	22,998	6,555	874	7,679	20,178
MCE-MIR_4743:fwd	5,288	5,299	1,608	5,972	9,599	4,947	680	459
MCE-MIR_3595:fwd	5,222	4,365	3,743	7,308	8,243	4,245	8,793	8,258
MCE-MIR_3470:rev	5,172	3,253	3,544	8,042	8,907	4,154	8,204	8,354
cand116	5,129	3,142	5,799	8,490	6,565	6,873	3,509	2,489
MCE-MIR_3762:rev	5,108	4,872	1,966	11,808	5,753	2,227	2,056	3,503
mmu-mir-25	4,962	4,796	3,927	9,616	5,193	3,332	1,211	1,002
cand523	4,955	3,492	8,036	4,360	13,495	8,471	1,624	834
cand91:a	4,955	3,392	1,984	7,801	5,917	1,630	12,555	13,984
cand613	4,884	3,953	4,390	10,781	12,814	4,857	6,935	9,383
MCE-MIR_5030:rev	4,814	3,745	1,051	5,820	11,544	5,748	377	310
MCE-MIR_5544:rev	4,805	4,872	1,686	4,640	15,912	8,280	473	219
MCE-MIR_1365:fwd	4,741	3,199	2,891	12,084	6,583	1,672	13,055	18,861
MCE-MIR_5399:fwd	4,722	3,104	2,488	6,785	5,515	2,807	6,596	5,296
mmu-mir-92	4,698	3,914	5,637	7,932	5,210	3,266	1,011	806
MCE-MIR_5291:fwd	4,684	2,673	2,949	3,690	12,956	8,258	257	159
cand616:a	4,662	3,762	1,100	13,851	7,441	1,958	10,486	14,792
MIR165	4,639	2,923	4,254	8,616	6,281	4,126	4,291	5,490
MCE-MIR_1778:fwd	4,538	2,604	3,704	5,886	8,402	3,963	7,378	6,192
cand231	4,518	3,072	2,936	8,723	11,033	3,556	20,459	23,532
MIR136	4,469	3,794	5,061	7,006	5,349	2,931	870	752
MCE-MIR_4462:fwd	4,419	2,547	4,343	8,950	7,952	3,576	6,480	8,455
mmu-mir-183	4,387	3,028	3,091	4,995	2,803	2,028	905	821
mmu-mir-302b	4,304	2,148	4,725	3,735	1,685	876		
cand279:a	4,293	2,465	983	7,008	3,887	1,385	2,230	2,784
mmu-mir-320	4,287	3,835	3,896	7,399	7,800	4,269	4,008	4,434
cand179:a	4,193	5,356	3,283	8,128	11,025	5,015	402	538
MCE-MIR_5079:rev	4,180	3,186	2,108	10,016	6,273	2,373	10,950	14,487

GCNF-/- Time:	Day 0		Day 1		Day 3		Day 6	
**Array Chip ID:**	**204**	**210**	**124**	**217**	**356**	**357**	**245**	**246**
MCE-MIR_3488:rev	4,153	2,297	2,837	7,157	7,970	3,109	2,197	2,564
MCE-MIR_5440:fwd	4,146	2,801	3,090	5,198	10,099	6,957	648	333
MCE-MIR_1352:fwd	4,138	4,316	1,535	12,378	6,045	2,369	1,894	4,273
MCE-MIR_1371:fwd	4,135	2,358	3,320	9,241	6,728	3,110	4,161	3,870
MCE-MIR_2698:rev	4,133	2,361	2,299	6,127	3,064	1,636	6,759	7,520
cand718:b	4,128	2,362	8,503	9,283	11,879	4,210	13,157	11,599
MCE-MIR_3484:rev	4,127	2,103	3,470	7,761	6,034	3,220	5,021	5,521
mmu-mir-182	4,126	2,943	2,652	6,196	1,945	1,369	766	571
MCE-MIR_1325:rev	4,096	2,172	3,930	7,353	4,767	3,628	1,968	1,538
cand362	4,000	2,663	1,634	1,668	4,809	3,521	153	132
MCE-MIR_4273:fwd	3,966	3,070	840	7,059	10,803	1,985	3,517	4,929
MCE-MIR_3143:fwd	3,904	2,142	2,312	5,780	7,408	3,437	1,681	1,804
MCE-MIR_5152:rev	3,856	2,419	2,723	6,161	8,070	4,604	339	223
cand342:a	3,837	3,370	4,797	5,255	5,719	3,453	1,477	1,086
MCE-MIR_3609:rev	3,819	2,486	3,849	6,426	6,735	3,291	4,263	3,063
mmu-mir-93	3,796	3,296	2,602	7,386	3,427	2,142	1,570	1,459
MCE-MIR_2139:fwd	3,791	2,556	1,567	8,259	3,067	1,115	9,398	13,047
MCE-MIR_5057:fwd	3,736	2,869	1,621	3,679	6,449	4,799	277	163
cand79	3,732	3,399	3,002	6,684	5,871	3,998	3,733	3,276
MCE-MIR_3642:rev	3,725	2,630	3,899	6,110	9,782	5,473	2,039	1,811
MCE-MIR_273:fwd	3,569	1,628	4,195	6,555	5,290	2,900	2,605	2,347
cand156	3,562	1,632	2,565	1,342	13,554	10,255	487	307
MCE-MIR_530:fwd	3,538	3,993	1,197	8,051	18,267	5,839	566	605
mmu-mir-291b-5p	3,466	1,996	1,715	6,501	3,515	1,994	831	491
cand425	3,429	2,568	2,164	3,296	5,744	2,571	967	916
MCE-MIR_3429:rev	3,281	1,828	1,978	7,988	5,708	1,519	4,554	5,670
MCE-MIR_4684:fwd	3,264	2,508	1,368	7,116	5,324	1,906	1,485	2,453
cand172:b	3,236	2,922	3,710	4,909	4,660	3,304	883	682
MCE-MIR_755:fwd	3,227	2,489	1,200	8,147	4,508	1,524	8,840	11,701
cand588	3,212	1,637	851	12,197	3,224	1,161	4,583	3,352
MCE-MIR_2714:rev	3,175	1,373	3,365	5,964	4,541	2,621	274	161
MCE-MIR_5607:rev	3,157	2,676	741	3,478	1,236	1,609	115	59
MCE-MIR_3847:fwd	3,128	2,473	1,932	5,367	11,903	7,711	3,740	2,218
cand374:b	3,080	1,833	2,660	7,496	8,604	3,781	5,587	3,148
mmu-mir-466	2,938	1,990	2,174	5,895	6,363	3,824	1,286	562
mmu-mir-341	2,924	1,085	5,049	2,568	3,233	3,174	12,221	3,547
MCE-MIR_1986:fwd	2,887	1,575	1,558	6,558	5,414	2,488	1,161	1,461
MCE-MIR_1734:fwd	2,834	1,621	4,578	4,271	11,008	7,765	1,173	1,235

GCNF-/- Time:	Day 0		Day 1		Day 3		Day 6	
**Array Chip ID:**	**204**	**210**	**124**	**217**	**356**	**357**	**245**	**246**
MCE-MIR_1784:fwd	2,814	1,932	774	4,722	10,029	6,180	72	
MCE-MIR_2817:fwd	2,812	1,987	7,698	7,524	9,394	2,994	18,320	31,481
MCE-MIR_4922:fwd	2,759	1,667	912	3,470	9,351	5,840	419	311
MCE-MIR_298:rev	2,705	1,242	1,347	4,794	6,232	2,790	2,299	1,980
MCE-MIR_1536:rev	2,648	1,540	1,247	7,767	4,049	1,573	2,542	5,329
MCE-MIR_2977:rev	2,610	1,370	132	3,471	5,148	2,369	273	205
MCE-MIR_4913:rev	2,602	2,130	602	2,515	10,377	4,866	89	44
cand418	2,594	1,735	550	3,292	3,022	1,760	243	242
cand699	2,560	1,176	1,783	5,910	3,818	1,217	2,763	3,222
mmu-mir-106b	2,539	2,756	1,061	6,469	1,969	1,303	849	763
MCE-MIR_5389:fwd	2,494	1,867	1,504	4,261	9,869	6,863	3,377	1,664
MCE-MIR_3685:rev	2,422	1,622	1,730	4,200	3,287	2,119	3,616	1,369
cand617	2,403	1,689	1,546	1,704	7,420	3,564	844	868
MCE-MIR_3531:rev	2,389	832	1,861	4,650	3,348	1,523	1,519	1,413
mmu-mir-291a-5p	2,388	1,429	1,082	4,406	2,335	1,289	390	129
cand252	2,374	2,243	3,128	4,677	2,166	1,529	1,703	1,222
mmu-mir-302	2,360	1,008	1,451	1,755	312	194		
MCE-MIR_5141:rev	2,357	1,579	435	3,184	4,323	2,398	418	126
mmu-mir-130a	2,337	2,455	1,001	6,103	2,219	1,379	1,420	1,267
mmu-mir-130b	2,333	1,973	1,013	4,342	965	595	677	518
MCE-MIR_451:rev	2,297	1,297	357	2,090	819	510	135	90
mmu-mir-19b	2,267	4,172	181	4,380	325	187	191	248
cand515	2,252	1,103	387	2,737	715	808	1,709	2,189
MCE-MIR_2099:fwd	2,213	1,424	1,185	4,883	2,060	1,082	585	565
MCE-MIR_3134:rev	2,173	1,323	4,340	2,755	23,189	15,971	2,197	782
MCE-MIR_5354:rev	2,143	873	68	2,919	1,240	593	92	48
MCE-MIR_1514:fwd	2,135	1,294	790	3,298	3,952	1,919	415	293
mmu-mir-467	2,134	1,350	1,699	4,050	4,794	2,632	1,565	805
MCE-MIR_810:rev	2,118	1,657	4,819	10,435	11,709	6,241	3,410	1,020
MCE-MIR_2745:fwd	2,113	1,321	747	2,126	2,700	1,392	195	109
MCE-MIR_3513:rev	2,107	1,569	3,273	5,323	9,525	5,351	4,368	2,677
cand465:a	2,094	1,397	2,885	1,560	979	571		
cand324:a	2,045	1,636	1,752	3,493	1,541	1,068	216	250
cand686	2,031	1,212	7,362	8,024	12,112	4,207	9,582	11,312
cand467	2,013	821	324	1,621	990	641	42	53
mmu-mir-200b	2,002	1,972	2,329	6,216	2,985	1,995	1,755	1,028
mmu-mir-21	2,000	2,513	1,938	5,999	3,139	2,524	860	1,003
MCE-MIR_3408:fwd	1,987	1,144	164	2,276	1,186	403	381	439

GCNF-/- Time:	Day 0		Day 1		Day 3		Day 6	
**Array Chip ID:**	**204**	**210**	**124**	**217**	**356**	**357**	**245**	**246**
MCE-MIR_4627:fwd	1,975	1,188	605	3,502	6,497	2,527	250	305
MCE-MIR_4999:rev	1,955	1,136	254	3,449	1,231	373	1,888	2,469
MCE-MIR_3780:rev	1,947	801	6,007	2,267	9,065	5,624	6,356	4,650
MCE-MIR_6054:rev	1,910	754	1,998	2,209	2,045	1,505	5,720	2,682
MIR30	1,898	1,128	1,535	2,214	2,992	1,661	171	200
MCE-MIR_5443:fwd	1,879	1,403	164	6,953	2,484	1,228	600	967
MCE-MIR_5384:rev	1,876	888	1,248	2,269	3,691	2,689	97	66
cand545	1,870	1,467	416	5,925	4,462	1,056	714	742
MCE-MIR_3523:rev	1,870	947	971	4,634	3,689	754	3,583	3,565
MCE-MIR_3529:rev	1,863	1,583	4,583	4,612	29,177	15,692	5,086	2,276
mmu-mir-7	1,825	1,240	809	3,987	1,517	1,101	102	123
mmu-mir-15b	1,817	1,529	2,075	3,689	2,411	1,744	1,428	902
cand126	1,805	1,004	1,050	775	4,798	2,959	413	185
MCE-MIR_4714:rev	1,782	1,343	82	2,329	2,524	1,584	74	66
MCE-MIR_5068:rev	1,753	753	179	5,010	1,427	371	6,450	9,622
MCE-MIR_534:fwd	1,748	809	576	865	8,978	3,658	63	
MCE-MIR_1973:fwd	1,746	1,099	365	978	6,848	4,175	29	60
MCE-MIR_4791:fwd	1,734	1,226	701	4,102	6,028	2,993	1,674	919
mmu-mir-200c	1,692	1,459	2,205	4,022	2,667	1,669	2,261	1,215
MCE-MIR_3260:rev	1,662	550	110	2,626	841	418	1,626	1,445
cand341	1,640	2,969	269	2,516	255	176	97	131
MCE-MIR_4661:rev	1,629	1,818	272	1,231	6,727	2,792	59	59
cand268:b	1,610	1,205	1,194	2,694	797	482	1,899	932
cand268:a	1,592	606	3,768	7,708	6,640	880	25,246	31,323
MCE-MIR_5470:fwd	1,579	1,042	482	1,822	2,698	1,641	114	66
MCE-MIR_3685:fwd	1,540	789	1,874	3,353	10,269	6,019	1,040	380
MCE-MIR_3859:fwd	1,495	1,074	630	2,931	7,563	4,702	2,290	1,179
cand42:a	1,478	1,288	992	5,101	5,929	2,161	1,131	634
mmu-mir-23b	1,469	1,983	2,903	6,210	2,955	2,104	1,676	1,177
MCE-MIR_4320:rev	1,460	734	783	1,641	5,944	3,169	33	35
MCE-MIR_4740:fwd	1,436	1,132	2,416	4,905	6,939	3,361	4,266	2,259
cand692:a	1,427	767	208	3,378	1,319	290	1,149	1,023
cand73	1,417	904	36	2,137	618	362	58	58
MIR206	1,410	695	2,082	4,617	3,632	614	8,590	4,203
cand135:b	1,395	894	717	2,446	1,027	681	1,205	833
MCE-MIR_1857:fwd	1,393	667	740	2,599	3,355	1,051	38	51
mmu-mir-23a	1,371	1,803	2,575	6,712	3,056	2,461	809	793
cand445	1,302	680	402	1,144	1,486	875	298	129

GCNF-/- Time:	Day 0		Day 1		Day 3		Day 6	
**Array Chip ID:**	**204**	**210**	**124**	**217**	**356**	**357**	**245**	**246**
mmu-mir-26a	1,273	3,298	5,041	9,231	7,266	5,200	2,818	1,853
MCE-MIR_5914:rev	1,263	578	190	3,396	2,102	722	354	131
mmu-mir-191	1,252	1,293	1,426	2,976	2,007	1,192	945	851
mmu-mir-103	1,225	1,763	1,002	3,851	1,679	946	802	1,065
mmu-mir-16	1,224	2,703	742	6,337	1,734	1,038	2,533	1,523
cand563	1,208	688	286	818	3,281	2,545	46	58
mmu-mir-24	1,197	1,855	1,372	5,887	1,874	1,155	1,641	1,279
MCE-MIR_5366:rev	1,181	474	97	1,678	1,151	529	1,089	1,464
MCE-MIR_1544:rev	1,164	268	488	2,581	1,016	501	732	849
MCE-MIR_3958:fwd	1,144	491	163	2,052	868	363	5,160	6,782
MIR166	1,132	400	541	1,443	1,192	494	1,515	1,130
MCE-MIR_2680:fwd	1,122	557	40	1,451	1,505	1,058	42	
MCE-MIR_3572:rev	1,102	438	146	1,774	780	426	1,608	2,547
mmu-mir-134	1,100	702	421	2,294	1,007	373	687	774
cand350	1,097	598	1,540	1,772	706	647	103	160
MCE-MIR_2679:fwd	1,097	437	41	3,910	679	422	3,369	4,772
MCE-MIR_3477:rev	1,083	194	962	3,406	968	211	1,072	720
MCE-MIR_5363:rev	1,051	640	152	2,373	447	304	1,403	1,219
MCE-MIR_5088:rev	1,048	1,080	46	1,718	1,258	449	79	119
MCE-MIR_5641:rev	1,033	1,083	513	2,469	7,519	2,180	437	555
MCE-MIR_1408:fwd	1,018	779	83	516	3,125	2,595		
MCE-MIR_670:fwd	1,016	581	170	1,035	3,615	1,245	41	37
cand618	1,005	456	264	3,426	1,716	563	1,933	1,919
cand24	988	732	169	1,754	1,118	216	352	525
MCE-MIR_2866:rev	987	427	18	1,595	878	697	87	105
MCE-MIR_5014:fwd	983	302		851	1,150	1,022	3,675	597
MCE-MIR_5643:fwd	970	894	108	822	4,642	1,090	41	37
MCE-MIR_4027:rev	970	683	586	2,291	1,853	643	2,196	2,572
cand557	970	640	716	2,071	1,945	612	6,150	5,403
mmu-mir-361	936	659	1,092	2,350	1,617	1,074	458	364
MCE-MIR_5216:rev	917	378	54	858	1,993	1,093	95	74
cand572:a	917	556	194	1,270	575	269	363	355
cand532:b	916	451	73	1,422	1,582	1,248	97	71
MCE-MIR_4674:fwd	915	599	370	2,606	6,114	2,345	1,671	3,048
MCE-MIR_5363:fwd	903	501	399	920	632	900	39	
MCE-MIR_4610:fwd	902	773	205	1,811	3,763	563	40	51
mmu-mir-27b	899	892	456	2,228	724	417	330	492
mmu-mir-494	876	621	336	4,518	2,453	1,240	65	51

GCNF-/- Time:	Day 0		Day 1		Day 3		Day 6	
Array Chip ID:	204	210	124	217	356	357	245	246
cand457:a	875	557	306	645	1,750	873	118	88
MCE-MIR_1364:fwd	871	290	443	650	1,433	839		
mmu-mir-107	858	1,233	665	2,958	1,413	776	566	676
MCE-MIR_5055:rev	853	376	331	2,738	1,050	315	637	615
MCE-MIR_3478:rev	841	199	29	514	314	209	443	336
mmu-mir-30c	833	1,605	1,692	4,343	1,910	1,337	1,353	882
MCE-MIR_5276:fwd	822	521	373	1,664	1,878	1,005	141	105
MCE-MIR_5083:rev	822	416		1,727	2,089	747	187	239
MCE-MIR_5056:rev	815	343	133	1,874	887	294	675	621
mmu-mir-99b	815	643	724	1,868	1,747	1,079	1,974	1,518
MCE-MIR_3541:rev	800	634	130	2,079	1,245	374	206	217
mmu-mir-210	789	612	388	1,420	392	242	574	293
mmu-mir-18	787	703	147	2,361	263	165	89	55
MCE-MIR_2986:fwd	780	351	128	1,502	4,428	2,341	33	
MCE-MIR_1074:rev	779	500	163	1,142	5,403	2,457	58	34
mmu-mir-541	777	633	376	1,300	520	292	279	113
mmu-mir-298	771	544	366	1,340	1,605	1,003	129	76
mmu-mir-127	763	747	290	1,332	490	295	325	261
mmu-mir-429	757	522	190	2,188	506	345	271	361
MIR74	754	420	130	2,992	652	381	250	482
MCE-MIR_6107:fwd	746	479	313	1,052	1,353	571	83	53
cand690	734	533	3,359	932	16,801	7,054	401	445
cand149:b	734	295	156	882	1,875	1,441	41	
cand144:a	732	617	297	1,447	629	444	183	130
cand103:b	712	902	1,210	2,310	1,370	765	295	429
MCE-MIR_6026:rev	712	418	62	929	2,058	343		
MCE-MIR_6050:rev	702	339		1,276	963	545	111	144
cand412	695	396	252	449	3,833	2,738	239	166
MCE-MIR_5411:rev	691	371		214	7,010	4,003		
MCE-MIR_5369:rev	685	386	72	975	543	293	128	95
cand342:b	673	275	532	1,080	1,881	1,464	111	92
MCE-MIR_2902:rev	670	390	214	1,193	5,165	1,380	126	100
cand462	668	292	248	667	5,319	4,579	116	90
mmu-mir-26b	667	595	559	2,328	1,348	922	263	677
mmu-mir-379	663	408	219	1,013	245	189	128	114
MCE-MIR_4755:fwd	659	396	137	812	1,386	656	54	39
cand371:b	638	257	207	1,323	984	308	307	332
cand315:b	618	492	682	1,352	755	423	395	302

GCNF-/- Time:	Day 0		Day 1		Day 3		Day 6	
**Array Chip ID:**	**204**	**210**	**124**	**217**	**356**	**357**	**245**	**246**
mmu-mir-125a	616	770	846	2,255	2,186	1,475	1,926	1,462
MCE-MIR_2968:fwd	614	201	135	429	488	191	68	35
mmu-mir-34a	606	512	360	1,779	725	527	505	247
S-mmu-mir-140	602	535	493	1,076	397	322	894	580
mmu-mir-205	591	312	225	985	342	214	372	165
MCE-MIR_638:fwd	586	294	57	725	238	190	37	56
cand459	568	257	48	1,631	1,447	141	754	758
MCE-MIR_543:fwd	564	271	98	628	1,695	684	244	312
MCE-MIR_406:rev	561	319		320	1,118	568		
cand104:a	548	557	502	1,301	478	356	374	439
MCE-MIR_3557:rev	538	440	218	2,262	1,946	333	1,261	1,020
MCE-MIR_2345:fwd	538	181		776	4,998	3,004		
mmu-mir-363	537	297	243	1,637	476	298		
cand595:a	535	199	87	1,156	1,014	558	57	30
cand564:b	531	438	198	788	337	192	195	157
MCE-MIR_5511:rev	515	413	61	214	2,153	1,321		
MCE-MIR_4198:fwd	513	206	43	753	1,024	436	51	
MCE-MIR_3624:rev	497	257	191	1,008	511	188	676	744
MCE-MIR_5055:fwd	496	254	22	629	432	239	52	42
MIR253	488	321	40	1,172	160	101	40	72
MCE-MIR_2470:fwd	486	286	189	753	792	215	136	129
MCE-MIR_3518:rev	482	246	22	977	965	178	436	391
MCE-MIR_3628:rev	477	237	940	1,122	4,712	904	2,480	1,352
MCE-MIR_3653:rev	466	254		518	1,816	902	99	
cand284:a	459	845	615	2,617	653	429	494	679
MCE-MIR_4010:rev	455	344	73	641	1,198	292	64	91
MCE-MIR_1611:fwd	453	200	184	695	1,774	372	145	86
cand152:b	447	305	568	812	1,172	786	345	149
MCE-MIR_5745:fwd	443	219	599	326	1,080	1,177	44	37
MIR257	438	166	379	857	845	575	27	83
cand317	426	284	280	663	492	356	363	294
MCE-MIR_4030:rev	414	145	67	592	2,598	1,495	21	42
MCE-MIR_2361:fwd	392	182		200	2,707	1,963		
MCE-MIR_5503:rev	390	231	15	492	3,851	2,143		36
cand314	380	557	500	1,650	777	472	476	581
mmu-mir-185	379	323	267	875	686	391	90	164
MCE-MIR_2815:fwd	374	170	46	574	669	309	45	45
cand276:a	370	320	264	936	397	284	248	154

GCNF-/- Time:	Day 0		Day 1		Day 3		Day 6	
**Array Chip ID:**	**204**	**210**	**124**	**217**	**356**	**357**	**245**	**246**
mmu-mir-155	365	100	693	1,204	426	186	233	431
mmu-mir-27a	364	432	115	1,638	311	192	89	280
MCE-MIR_2889:fwd	363	217	211	425	581	245	82	77
MCE-MIR_1066:rev	348	230	93	418	1,272	327	42	46
mmu-mir-342	348	242	289	630	1,251	529	236	243
cand137:a	346	524	244	1,820	247	175	252	510
mmu-mir-302c	341	113	24	240	113	52		
mmu-mir-148a	328	289	116	1,631	204	162	99	283
mmu-mir-291b-3p	327	119		1,550	162	351		
MCE-MIR_1974:fwd	323	111	33	53	2,445	1,915		
cand42:b	323	268	106	657	403	230	589	392
cand570	320	97		252	1,214	1,213		
mmu-mir-30b	319	439	590	2,179	890	650	469	664
MCE-MIR_936:rev	311	129	49	316	324	155	84	90
mmu-mir-376b	310	191	47	564	97	53	56	104
cand65	310	116	28	950	356	74	475	525
MCE-MIR_2983:rev	304	192	131	320	554	327	94	141
MCE-MIR_3439:rev	303	142		494	649	305		35
cand532:a	303	270	35	2,404	473	97	548	430
mmu-mir-382	300	184	105	557	274	191	119	80
cand347	300	113	41	735	408	122	288	206
mmu-mir-181b	299	221	70	1,958	795	508	382	846
MCE-MIR_2078:fwd	291	194	387	751	487	233	848	391
MCE-MIR_3474:rev	288	56	63	694	295	194		
MCE-MIR_1569:rev	286	114	93	403	1,142	269	100	86
cand315:a	279	186	180	468	382	231	105	98
MCE-MIR_4345:rev	278	83		135	618	294		
MCE-MIR_3613:rev	272	91	26	366	238	90		94
mmu-mir-409	272	232	134	508	773	337	72	49
MCE-MIR_3492:fwd	266	157	48	806	2,838	719	79	71
mmu-mir-151	264	189	186	588	346	216	114	41
MCE-MIR_3886:fwd	263	167	89	425	152	79	81	97
mmu-mir-346	262	159	159	747	558	323	358	440
mmu-mir-324-5p	257	193	165	702	353	222	282	176
cand109:b	252	136	88	401	407	198	135	165
cand624	250	116		344	72	91	99	180
MCE-MIR_4922:rev	247	168	30	390	1,030	580		
MCE-MIR_329:fwd	247	84	124	397	343	233	35	

GCNF-/- Time:	Day 0		Day 1		Day 3		Day 6	
Array Chip ID:	204	210	124	217	356	357	245	246
mmu-mir-297	244	83	15	625	236	126		
cand244	244	135	52	145	471	305	33	
MCE-MIR_5473:fwd	239	168	71	272	2,424	693	43	
MCE-MIR_2285:fwd	235	93	36	270	1,230	760	26	72
MCE-MIR_5872:fwd	235	123	60	355	420	279	76	
MCE-MIR_3847:rev	230	148	76	412	161	101	95	83
mmu-mir-195	227	292	73	1,494	264	137	90	353
mmu-mir-324-3p	227	169	127	527	305	180	151	118
MCE-MIR_4726:fwd	224	299	106	695	16,257	5,809	93	152
MCE-MIR_5596:rev	218	124	68	201	456	190	38	
MCE-MIR_1793:fwd	217	126	23	192	405	340	30	32
cand497:a	208	214	103	676	438	150	279	869
mmu-mir-422b	205	264	64	409	139	81	68	182
MCE-MIR_2196:fwd	205	65	17	155	404	185	66	103
MCE-MIR_5790:fwd	204	93		512	707	336	78	
MCE-MIR_1046:rev	204	99	43	276	529	261	51	51
mmu-mir-17-3p	203	146	97	453	137	110	47	
MCE-MIR_3791:rev	198	170		741	834	260	63	53
MCE-MIR_4153:fwd	196	77	33	430	218	143	63	32
MCE-MIR_469:fwd	193	112	73	224	535	282	63	57
MCE-MIR_5004:rev	192	134	29	778	331	114	188	177
mmu-mir-152	191	152	77	822	128	79	163	200
MCE-MIR_18:fwd	188	69	21	128	272	118		
mmu-mir-101b	185	205	16	545	89	53	23	129
MCE-MIR_1756:rev	181	107	58	178	354	273	37	66
MCE-MIR_4031:fwd	181	105	69	196	481	236	44	96
MCE-MIR_1508:fwd	181	103		758	225	105	67	54
MCE-MIR_2563:fwd	179	247	400	1,431	5,729	1,740	1,116	1,010
cand5	177	79	25	927	146	50	474	564
mmu-mir-335	176	120		1,090	395	370	89	115
MCE-MIR_1283:fwd	175	98	26	402	235	164	109	194
MCE-MIR_2092:fwd	175	88	25	231	536	118		
cand334:b	173	138	27	200	145	69	74	92
MCE-MIR_5197:rev	171	67	54	338	124	88	77	
MCE-MIR_1788:rev	171	65	42	146	1,537	2,050		
cand340	170	70	19	197	225	98	106	86
MCE-MIR_1458:rev	168	47	33	453	122	136	69	73
MCE-MIR_3663:fwd	168	97	76	314	417	217	167	214

GCNF-/- Time:	Day 0		Day 1		Day 3		Day 6	
**Array Chip ID:**	**204**	**210**	**124**	**217**	**356**	**357**	**245**	**246**
mmu-mir-28	167	114	67	365	321	189	60	56
cand192	163	69	13	467	642	123	106	77
mmu-mir-31	163	140	137	458	171	121	245	184
cand541	161	118	58	737	1,213	146	309	229
mmu-mir-30d	158	249	151	977	513	295	104	311
MCE-MIR_2680:rev	155	117		890	657	418	58	54
cand501	153	100	24	324	176	71	238	296
cand57	151	116		210	993	256		
MCE-MIR_5339:rev	150	71	36	298	93	70	48	58
MCE-MIR_4716:fwd	149	58	41	310	346	198	36	
MCE-MIR_4791:rev	148	95	96	454	291	222	33	
cand26	143	69	28	220	228	113	114	263
cand386:a	143	95		141	364	265	88	139
MCE-MIR_5473:rev	139	115	28	324	385	193	96	183
MCE-MIR_1342:fwd	139	199	25	335	444	50		60
cand200	139	38		431	9,470	5,914	16	
mmu-mir-433-3p	138	104	116	259	233	128	87	40
cand304	138	117	30	300	113	88	62	465
mmu-mir-484	136	109	76	422	506	285	23	63
mmu-mir-214	135	66	66	371	959	540	1,208	927
MCE-MIR_3471:rev	132	30		335	169	72	248	160
MCE-MIR_1015:rev	131	64	66	265	483	122	166	
MCE-MIR_5418:fwd	131	73	105	211	4,545	3,251	35	42
MCE-MIR_3101:rev	131	55	18	136	467	232		32
MCE-MIR_725:fwd	130	98		389	149	109	196	313
MIR85	129	335	92	913	144	103	42	394
mmu-mir-370	128	58	37	206	183	102	68	
MCE-MIR_4513:fwd	125	67	26	429	757	215	107	103
cand297:b	125	155	41	572	104	53	68	545
MCE-MIR_6084:rev	124	87		187	44	42	29	
MCE-MIR_3441:fwd	124	68	20	236	781	309		
MCE-MIR_3407:rev	122	31	44	125	288	69		
mmu-mir-434-3p	122	98	34	244	100	62	96	139
cand334:a	119	107		500	529	83	63	55
MCE-MIR_2349:rev	119	86	43	207	1,048	283		66
S-mmu-let-7d	119	103	55	220	204	139	31	47
cand275	119	74		223	565	182	32	45
MCE-MIR_6055:rev	119	68	23	200	650	193	40	

GCNF-/- Time:	Day 0		Day 1		Day 3		Day 6	
**Array Chip ID:**	**204**	**210**	**124**	**217**	**356**	**357**	**245**	**246**
MCE-MIR_993:rev	118	20		53	43	30	202	81
mmu-mir-129-5p	115	77	30	169	353	210	32	54
mmu-mir-15a	115	93		327	79	48		259
mmu-mir-19a	113	280		433	34	15		53
cand97	113	88		163	51	35		59
mmu-mir-296	111	106		222	325	231		50
MCE-MIR_2524:fwd	110	69	105	389	521	125	420	563
MCE-MIR_5389:rev	109	66	20	162	141	80	75	73
MCE-MIR_2566:fwd	109	42		214	155	127		
mmu-mir-30a-5p	108	161	45	857	159	97	35	378
S-mmu-mir-302c	105	66	43	137	40	34		
MCE-MIR_1746:rev	102	54		526	977	499		
cand386:b	102	62	37		197	182		51
MCE-MIR_988:fwd	101	84		230	92	86	115	191
cand616:b	100	43	27	155	245	74	40	51
cand374:a	99	166	138	309	771	677	1,365	1,009
cand678	99	83	24	493	138	24	54	35
MCE-MIR_3444:rev	98	74	23	120	92	46		
MCE-MIR_2661:rev	98	31		94	150	86	40	
MCE-MIR_5193:rev	97	36	16	180	110	72	43	
MCE-MIR_5454:rev	96	71		239	198	135	82	147
mmu-mir-546	94	43		153	329	137		
MCE-MIR_4714:fwd	92	54		148	125	77	54	63
MCE-MIR_557:fwd	91	69		241	309	120	84	137
cand78:a	91	38		135				
MCE-MIR_5328:rev	91	59	18	341	4,430	2,582		
MCE-MIR_5210:fwd	89	38		91	83	59	50	38
mmu-mir-188	89	39	19	219	117	81	80	113
MCE-MIR_1773:rev	89	70	23	128	266	93	42	85
mmu-mir-29a	89	129	31	447	36			470
MIR124	89	84	30	394	107	66	68	73
MCE-MIR_3261:fwd	88	42		161	114	68	70	74
cand115:b	88	64		183	3,429	1,570	20	
MCE-MIR_4748:rev	88	66	28	112	140	70	62	127
MCE-MIR_1478:fwd	88	30	55	175	125	64	126	85
mmu-mir-145	86	170	89	328	229	115	91	313
mmu-mir-124a	86	208	37	497	104	83	27	273
MCE-MIR_5295:rev	85	80		241	196	120	83	118

GCNF-/- Time:	Day 0		Day 1		Day 3		Day 6	
**Array Chip ID:**	**204**	**210**	**124**	**217**	**356**	**357**	**245**	**246**
MCE-MIR_1283:rev	84	50		61	821	437		
cand118	84	94	385	317	980	927	845	1,032
cand457:b	83	58	88	101	1,324	632	59	87
mmu-mir-149	82	84	55	305	124	99		72
MCE-MIR_4763:fwd	81	66		151	144	135	54	127
MCE-MIR_5100:rev	81	39	13	187	411	155	89	89
mmu-let-7c	80	99	481	409	880	983	845	1,132
MCE-MIR_777:rev	80	68		58	2,736	500		
MCE-MIR_5488:rev	80	83	16	147	217	121	58	66
MCE-MIR_5322:fwd	79	71		232	164	116	66	135
MCE-MIR_4752:rev	79	62		217	92	99	80	163
MIR202	79	67	31	216	107	57		55
MCE-MIR_734:fwd	78	57	26	122	130	85	42	45
mmu-mir-186	78	36		156	29	22		
MCE-MIR_942:rev	77	56	17	143	813	154	44	76
MCE-MIR_293:fwd	77	31		133	216	111	87	69
cand1:b	77	87	30	247	151	89	65	250
MCE-MIR_5406:rev	76	48	22	82	48	31	32	78
mmu-mir-351	73	42	30	177	197	147	348	181
MCE-MIR_4712:fwd	73	56		171	96	97	64	103
cand286	73	56		531	205	38	144	155
MCE-MIR_3226:fwd	73	30		89	202	196	34	
mmu-mir-425	73	70		187	104	45	37	58
cand278:a	73	38	41	223	110	85	101	161
MCE-MIR_5736:rev	73	55	43	69	349	205	57	70
MCE-MIR_5152:fwd	72	50	18	70	334	169	33	65
MCE-MIR_1931:fwd	70	28		99	326	188	109	108
cand284:b	70	31	49	256	183	140	97	97
MCE-MIR_3820:fwd	70	48		203	116	47	51	156
cand153:a	69	58		188	177	96	33	74
mmu-mir-329	69	70	30	265	180	113	52	50
MIR75	69	36	28	268	450	310	729	608
mmu-mir-200a	68	53	18	436	60	41		183
cand309	68	26		140	83	24	442	260
cand161	67	82	331	236	724	648	732	902
mmu-mir-150	67	75	40	139		35		65
MCE-MIR_399:fwd	67	29	15	97	94	47		
mmu-mir-223	67	53		250	137	122	58	128

GCNF-/- Time:	Day 0		Day 1		Day 3		Day 6	
Array Chip ID:	204	210	124	217	356	357	245	246
MCE-MIR_4202:rev	66	34		98	222	157	26	
cand346	65	37		108	2,007	230	32	44
MCE-MIR_1576:rev	65	35	17	115	164	115	28	36
mmu-mir-126-3p	65	214	17	601	79	48	31	506
mmu-mir-328	64	34	25	136	413	179	43	41
MCE-MIR_4756:rev	64	45		77	73	88	47	105
MCE-MIR_3667:fwd	64	28	20	56	159	125		
mmu-let-7a	64	65	305	251	649	782	403	811
MCE-MIR_5135:fwd	64	47	32	69	259	127	53	
mmu-let-7e	63	59	212	170	464	475	309	571
MCE-MIR_2111:fwd	63	46	24	99	569	287		
cand465:b	62	33		52				
MCE-MIR_959:rev	62	60		79	53	49		
cand337	62	54	54	199	59	34	45	137
MCE-MIR_5597:fwd	60	49		79	80	70	27	65
MCE-MIR_3624:fwd	60	49	21	58	53	42		109
MCE-MIR_3797:fwd	59	26	18	113	413	261	108	67
cand718:a	59	29		64	57	52		
MCE-MIR_2304:rev	59	40		56		27		62
mmu-mir-431	59	47	24	196	108	62	69	
cand490	59	67	215	157	469	405	261	629
MCE-MIR_2134:fwd	58	36		118	308	102		
cand302:b	58	35		299	86	30	74	95
MCE-MIR_1742:rev	57	25		122	956	56		
cand90:a	57	23	72	245	143	76	146	394
MCE-MIR_3684:rev	57	26		102	173	84	87	
MCE-MIR_1495:fwd	56	36		71	108	64		42
cand336	55	43	22	184	66	40	58	147
MCE-MIR_2464:fwd	55	30		89	107	56		
cand709	54	51	195	103	433	381	240	701
mmu-mir-345	54	50	14	141	111	83		37
MCE-MIR_2617:fwd	54	33		97	138	81	19	
mmu-mir-543	54	66	23	208	95	54		
MCE-MIR_364:fwd	53	21		81	111	79	70	85
mmu-mir-207	53	36	11	142	585	250	21	60
mmu-mir-224	52	20	36	68	60	71	24	
cand224	52	15		63	60		20	
MCE-MIR_780:rev	51	37		64	497	228		

GCNF-/- Time:	Day 0		Day 1		Day 3		Day 6	
Array Chip ID:	204	210	124	217	356	357	245	246
mmu-mir-540	51	41	16	74	51	25		
MCE-MIR_5300:fwd	51	52		118	104	66	52	48
MCE-MIR_162:fwd	50	19		83	77	46		
cand104:b	50	32	25	60	26	28		
MCE-MIR_4087:rev	50	29		63	151	118	28	
MIR102	50	36		95	219	132	45	59
MCE-MIR_5143:rev	49	34		57		26		
mmu-mir-187	48	30	17	110	90	56		47
mmu-mir-330	48	34	40	92	88	56		
cand106:a	48	40	94	60	398	357	216	617
cand667:b	48	29	16	178	179	62	45	
cand119	48	39	148	71	358	340	255	635
mmu-let-7b	48	35	187	100	333	378	239	670
MCE-MIR_3646:rev	47	18	31	114	80	43	40	
mmu-mir-181a	47	83	43	239	261	148	511	745
mmu-let-7d	47	40	114	79	322	365	170	582
cand13	47	48		94	136	54		
MCE-MIR_4675:fwd	46	28		81	143	87		
cand172:a	46	32		51		33		
MCE-MIR_1670:fwd	45	16		64	298	113	23	
MCE-MIR_4493:fwd	45	23	14	43	59	38		
mmu-mir-412	45	40	16	69	59	37	20	40
cand146	45	32	29	227	147	47	561	494
cand103:a	44	27	39	54		41		
MCE-MIR_2192:rev	44	18			94	44		
MCE-MIR_3793:rev	44	16		86	59	57	64	33
MCE-MIR_5340:fwd	43	35		85	1,672	363		
MCE-MIR_4179:rev	43	14	29	104	91	92	439	222
mmu-mir-211	43	33		65	29	26		
mmu-mir-101a	42	39		107	25			95
mmu-mir-143	42	128	18	351	43	26	29	313
MCE-MIR_3059:rev	42	21		52				44
MCE-MIR_4239:rev	41	22		69	167	104		
mmu-mir-96	41	27		108				
MCE-MIR_4497:rev	41	25		51	715	315		
mmu-mir-222	41	29	24	143	146	70	58	148
MCE-MIR_4726:rev	41	35		93	152	91	43	79
MCE-MIR_463:fwd	41	37		116	104	63		50

GCNF-/- Time:	Day 0		Day 1		Day 3		Day 6	
**Array Chip ID:**	**204**	**210**	**124**	**217**	**356**	**357**	**245**	**246**
cand705	41	47		84	503	135		58
cand149:a	40	25		65	429	246	44	90
mmu-mir-128a	40	42	24	108	153	114		228
MCE-MIR_4383:rev	40	16		52	92	72	44	
mmu-mir-125b	40	80	19	219	763	454	589	672
mmu-let-7f	39	16	73	37	225	231	92	564
cand415	39	18			42	57	126	94
mmu-mir-22	39	81		209	141	83	91	361
MCE-MIR_4034:rev	38	15		52	115		29	47
cand529	38	41		171	84	39	84	53
cand619	37	29	27	128	123	84	33	40
cand210:a	37	36	23	87	27	36		
MCE-MIR_3888:rev	35	20		44	70	65	24	43
MCE-MIR_1356:fwd	35	23		85	108	77		
MCE-MIR_4745:fwd	35	38		39	53	36		51
MCE-MIR_3113:fwd	35	24		40				
cand203	35	41		90	68	31	29	42
cand500:a	35	31		103	75	51	42	96
MIR4	35	20	22	35	33	30		94
mmu-mir-154	34	32		80	38	19		
MCE-MIR_2501:fwd	34			54	68	36		
cand100:b	34			25	34	38		
MCE-MIR_4711:fwd	34	26	16	29	44	35		
mmu-mir-301	34	28		300	38	26	55	68
MIR121	34	34	25	96	81	46		163
cand106:b	33	36		77	47	43	29	40
MCE-MIR_1059:fwd	33	20		108	76	52		35
cand157	33	42	89	44	74	67	91	332
MCE-MIR_2894:fwd	33	13		50	101	49		35
mmu-mir-487b	32	29	22	101	80	53		44
MCE-MIR_2173:rev	32			25	149	62		
MIR103	32	26		103	48	28	51	120
mmu-mir-350	31	21		152	42	36		
cand64	31	22		144	76	19	253	215
mmu-mir-381	30	22	9	55	47	36		
MCE-MIR_2711:rev	30	17		41	109	64		35
cand664	30	19		28	77	31		
cand91:b	30	21		42	123	44		

GCNF-/- Time:	Day 0		Day 1		Day 3		Day 6	
**Array Chip ID:**	**204**	**210**	**124**	**217**	**356**	**357**	**245**	**246**
MCE-MIR_2166:rev	30	17		49	94	56		
MCE-MIR_4022:fwd	29	18		71	87	53		50
MCE-MIR_3886:rev	29	14			45	36		
cand35	29	20		20	77	83		
MCE-MIR_3333:rev	29			31	100	44		
mmu-mir-128b	29	25	15	95	134	96		165
cand348	29	19	54		69	87	30	362
cand152:a	29	23		85	442	111	95	77
MCE-MIR_4738:fwd	29	15		52	70	54	25	
mmu-let-7g	29	24	57	36	61	42	57	484
cand6	28	27		80	83	24	38	79
mmu-mir-485-3p	28	23	10	61	87	25		
mmu-mir-221	28	22		154	80	46	61	255
mmu-mir-203	28	21	21	78	60	38	30	133
mmu-mir-300	28	20		62	39	30		
mmu-mir-362	28	20		85	58	52	40	
MCE-MIR_4932:rev	27	25		34		24		
MCE-MIR_3695:fwd	27	17	16	39	36	27	17	
MCE-MIR_2722:rev	26	15	13	31				38
cand276:b	26	21		51		20		
mmu-let-7i	26	20	27	27	44	29	109	528
mmu-mir-451	25	261		600				580
MCE-MIR_4069:rev	25	14		44	138	108	56	36
MCE-MIR_89:fwd	25	20		28	63	40	82	108
mmu-mir-485-5p	25	21		33		19		
mmu-mir-323	25	18		55	36	19		
MCE-MIR_4236:rev	25			38	74	34		
MCE-MIR_5167:rev	24			50	49	46		
mmu-mir-486	24	19		51	92	49		70
MCE-MIR_2364:rev	24	25		54	94	60		
MCE-MIR_4015:fwd	24	20		22	32		35	63
MCE-MIR_1226:fwd	23			25	161	67		
cand351	23	18	18	41	122	85	19	
mmu-mir-133b	23	28		61	102	55		120
MCE-MIR_1697:fwd	23			54	53	35		
cand302:a	23	22		25	69	42		
cand120	23	18		51	199	114		49
cand572:b	22	19	50	39	85	64	74	103

GCNF-/- Time:	Day 0		Day 1		Day 3		Day 6	
**Array Chip ID:**	**204**	**210**	**124**	**217**	**356**	**357**	**245**	**246**
MIR43	22	22		62				
mmu-mir-344	22	20		80	30	27	24	43
mmu-mir-122a	22	84			20	17		544
cand650	22	19		39	71	45		
mmu-mir-337	21	16		82				34
MCE-MIR_1482:rev	21	19			29			
MCE-MIR_2474:rev	21	20		71	85	54		
MCE-MIR_6001:rev	21				64	37		
MCE-MIR_1689:rev	21	14		39	41	26		
mmu-mir-377	21		88			251	107	
MCE-MIR_4521:rev	21				25	24		
mmu-mir-503	20	17	12	74	127	99	57	50
MCE-MIR_5180:rev	20			32	30			
MCE-MIR_5008:rev	20	18		43		32		
MCE-MIR_1829:rev	20			122	207	85		
mmu-mir-470	20	14		30	89	61		36
MCE-MIR_4063:rev	20			34	40		60	47
cand184	20	17		25		31	17	
MCE-MIR_5033:rev	19	19		29	30	25		
S-mmu-mir-199a	19	15		100	51	24	106	469
cand213	19	14		34				
mmu-mir-30e	19	15		69				206
MCE-MIR_6026:fwd	19	18	123	44	520	237	209	45
mmu-mir-299	19	18		61	35	26		
cand226	19			34	181	121		110
MCE-MIR_3859:rev	19	15		48	79	26	23	31
mmu-mir-133a	18	31		45	95	60		128
MCE-MIR_1546:rev	18			31	32	31		
cand669	18	15		36	79	59	30	
mmu-mir-132	18	12		20	74	28		60
MCE-MIR_4893:fwd	18	19		36	38			
MCE-MIR_1788:fwd	18	17		26	148	77		28
mmu-mir-206	18				26	22		208
mmu-mir-146	18	15	22	28	42	25		265
cand585	18			31	32			
MCE-MIR_5970:rev	18	16	43	29	58	48	34	
mmu-mir-148b	18	21		53	52	25		
cand210:b	18	19						

GCNF-/- Time:	Day 0		Day 1		Day 3		Day 6	
**Array Chip ID:**	**204**	**210**	**124**	**217**	**356**	**357**	**245**	**246**
MCE-MIR_334:rev	17			50	291	158		
MCE-MIR_4061:rev	17			25	21		36	
mmu-mir-181c	17	20		76	52	33		
MCE-MIR_871:fwd	17	20		21	26			64
mmu-mir-452	17	16		30	39	33		
MCE-MIR_4491:rev	17	15		119	397	424		46
MCE-MIR_4554:rev	17			51	92	56		34
MCE-MIR_1811:rev	16			37	48	28		33
MCE-MIR_5396:rev	16	13	23	25	33		66	45
MCE-MIR_4809:fwd	16							
MCE-MIR_1645:rev	16				24	18		
MCE-MIR_3057:fwd	16					38		
mmu-mir-129-3p	16	20		52	65	35		42
mmu-mir-204	16			29				45
MCE-MIR_1433:fwd	16			33			29	32
mmu-mir-331	15	16		70	76	44		
mmu-mir-410	15			48	37			
cand1:a	15		13	37		40		
MCE-MIR_4762:fwd	15	16			46	25		51
cand70	15			43	132	30		
MCE-MIR_4353:rev	15			27		18	21	
MCE-MIR_4280:fwd	15				32	24		
MCE-MIR_407:fwd	15			22				
cand137:b	15	21		20		35		
cand361	15				39	65	45	58
MIR141	14			48	93	70		
mmu-mir-194	14	15		43				162
cand349:a	14	17		30	156	118		52
MCE-MIR_5236:rev	14			26				
mmu-mir-218	14			52	36	21	33	119
MCE-MIR_3841:fwd	14					34	20	
mmu-mir-339	14	18		32				
mmu-mir-34c	14	13		38		28		95
cand212	14	13		45		21		
mmu-mir-184	14			24	57	34		
mmu-mir-193	14			25	38	23		
mmu-mir-424	13			80	51	33	83	264
MCE-MIR_3754:rev	13			28	47	33		

GCNF-/- Time:	Day 0		Day 1		Day 3		Day 6	
**Array Chip ID:**	**204**	**210**	**124**	**217**	**356**	**357**	**245**	**246**
MCE-MIR_5322:rev	13				72	76		
MCE-MIR_293:rev	13			23		24		36
mmu-mir-483	13	15		40	60	31		33
cand135:a	13	16		34				39
mmu-mir-1	13							436
mmu-mir-30a-3p	13			50	41	34		36
cand707	13					19		
MCE-MIR_1365:rev	13	12		36	65	43		
mmu-mir-468	13		18	60	165	78		
mmu-mir-378	13			20	24	16		
MIR79	12		20	35	134	97	153	
MCE-MIR_3522:rev	12							
mmu-mir-142-5p	12	16		21		16		
MCE-MIR_1192:rev	12	14			68	51		
mmu-mir-212	12	19		35	95	49		33
mmu-mir-29b	12							228
MCE-MIR_5620:fwd	11			28	109	88	18	
MCE-MIR_1642:rev	11				51	54	101	
cand375	11	13		124	209	53	171	279
MCE-MIR_1052:rev	11			27	44	35	21	
MCE-MIR_3084:fwd	11		26	28	197	158		
mmu-mir-376a	11			33				
cand262	11				30		15	90
cand40	11							43
MCE-MIR_3007:rev	10							
mmu-mir-199a	10	13		50				134
MCE-MIR_3429:fwd	10			26	385	211		
mmu-mir-383	10			24	68	38		
mmu-mir-411	10			23	102	44		
MCE-MIR_2339:fwd	10					29		57
mmu-mir-434-5p	10	18		27				
cand564:a	10			27				
MCE-MIR_3667:rev	10			18	69	43	16	
mmu-mir-380-3p	10			26				
mmu-mir-100	10			75	39	26	28	119
MCE-MIR_4280:rev	9				32	23		
S-mmu-mir-9	9			39			37	150
mmu-mir-99a	9			26	34		89	267

GCNF-/- Time:	Day 0		Day 1		Day 3		Day 6	
Array Chip ID:	204	210	124	217	356	357	245	246
cand614	9							300
MCE-MIR_4060:rev	9							
MCE-MIR_3550:fwd	9				37	39	19	
cand178:b	9			18	83	23		
MIR35	9						93	292
cand90:b	9			48	27	27		59
MCE-MIR_4712:rev	8							
MCE-MIR_4472:fwd	8							
mmu-mir-29c	8			21				112
MCE-MIR_3651:fwd	8			19				
MCE-MIR_782:rev	8				31	30		
MCE-MIR_5712:rev	8			61	93	38	90	116
cand129	7				20			
MCE-MIR_4182:rev	7							
mmu-mir-326	7							
mmu-mir-98	7				29	37		102
MCE-MIR_4503:rev	7							
MIR12	7							89
mmu-mir-9	7			33	53	31	22	125
mmu-mir-322	7			22		22	25	45
mmu-mir-199b	6							55
mmu-mir-375	6			29	26	19	19	
mmu-mir-450	5			30				
cand11:a								
cand130								
cand139								
cand144:b								
cand153:b				17				
cand179:b						26		
cand202								
cand22								
cand234								
cand25							33	
cand27					26			
cand278:b					66	26		
cand297:a								
cand324:b								
cand349:b								

GCNF-/- Time:	Day 0		Day 1		Day 3		Day 6	
Array Chip ID:	204	210	124	217	356	357	245	246
cand352:a								
cand352:b								
cand427					123	55		
cand492					1,772	745		
cand497:b								59
cand50								
cand500:b		14		22	42	27		
cand524						16		
cand549								
cand590								
cand7				49	540	441		
cand708:a						19		
cand82								
MCE-MIR_1259:rev								
MCE-MIR_1264:rev						34		
MCE-MIR_1269:rev					22	15		
MCE-MIR_1311:rev					44	33		
MCE-MIR_1356:rev						33		
MCE-MIR_1401:fwd								
MCE-MIR_1412:fwd		12		25	22			
MCE-MIR_1433:rev				21				
MCE-MIR_1442:fwd								
MCE-MIR_151:fwd				28	44	24		41
MCE-MIR_1679:fwd				20				
MCE-MIR_1710:rev								
MCE-MIR_1786:fwd								
MCE-MIR_188:fwd								
MCE-MIR_1905:rev					41	32		
MCE-MIR_1929:fwd								
MCE-MIR_1998:fwd				15				
MCE-MIR_2087:rev								
MCE-MIR_2166:fwd								42
MCE-MIR_2197:fwd								
MCE-MIR_2198:fwd								
MCE-MIR_2205:fwd								
MCE-MIR_2288:fwd					32			46
MCE-MIR_2339:rev								
MCE-MIR_2371:fwd					44			

GCNF-/- Time:	Day 0		Day 1		Day 3		Day 6	
**Array Chip ID:**	**204**	**210**	**124**	**217**	**356**	**357**	**245**	**246**
MCE-MIR_2371:rev								
MCE-MIR_2417:fwd								
MCE-MIR_2419:fwd								
MCE-MIR_2522:rev								
MCE-MIR_2566:rev				31	94	29		
MCE-MIR_2624:rev				28		25		
MCE-MIR_2691:rev								
MCE-MIR_2798:fwd								
MCE-MIR_281:rev								
MCE-MIR_2902:fwd					226	158		
MCE-MIR_291:rev								
MCE-MIR_3032:fwd								
MCE-MIR_3048:rev						49		
MCE-MIR_3057:rev								
MCE-MIR_3330:fwd								
MCE-MIR_3379:fwd					50	58		
MCE-MIR_3513:fwd					66	36		
MCE-MIR_3619:rev								
MCE-MIR_3637:rev								
MCE-MIR_3715:rev								
MCE-MIR_3832:rev								
MCE-MIR_3867:rev								
MCE-MIR_405:rev					35			
MCE-MIR_4063:fwd								
MCE-MIR_4124:fwd								
MCE-MIR_4179:fwd								
MCE-MIR_4207:fwd								
MCE-MIR_4209:fwd								
MCE-MIR_4232:fwd								
MCE-MIR_4303:fwd								
MCE-MIR_4342:fwd								
MCE-MIR_4413:fwd								
MCE-MIR_4442:fwd				29		32		
MCE-MIR_4449:rev								
MCE-MIR_4474:fwd								
MCE-MIR_4491:fwd								
MCE-MIR_4521:fwd								
MCE-MIR_4592:fwd								

GCNF-/- Time:	Day 0		Day 1		Day 3		Day 6	
**Array Chip ID:**	**204**	**210**	**124**	**217**	**356**	**357**	**245**	**246**
MCE-MIR_4607:fwd								
MCE-MIR_4614:rev					39	23		46
MCE-MIR_466:fwd								
MCE-MIR_4667:fwd					28			
MCE-MIR_4789:rev								
MCE-MIR_4799:rev						27		
MCE-MIR_482:fwd								
MCE-MIR_482:rev								
MCE-MIR_4830:fwd							19	
MCE-MIR_4832:rev								
MCE-MIR_4853:fwd						21		
MCE-MIR_4893:rev								
MCE-MIR_4972:fwd								
MCE-MIR_4978:rev								
MCE-MIR_504:fwd								
MCE-MIR_5046:rev								
MCE-MIR_5105:rev				41	96	60		
MCE-MIR_5122:rev				32	55	40		
MCE-MIR_5167:fwd								
MCE-MIR_5180:fwd								
MCE-MIR_5260:fwd								
MCE-MIR_5279:fwd								
MCE-MIR_5287:rev							18	
MCE-MIR_5300:rev								
MCE-MIR_5303:fwd				27	269	139	259	380
MCE-MIR_5374:rev								
MCE-MIR_5504:rev								
MCE-MIR_5581:fwd							22	
MCE-MIR_5581:rev							17	38
MCE-MIR_5598:fwd								
MCE-MIR_5623:rev								
MCE-MIR_5704:rev								58
MCE-MIR_5864:fwd								
MCE-MIR_6033:fwd				23	52	21		
MCE-MIR_6034:rev				42	243	98		
MCE-MIR_6120:fwd							13	
MCE-MIR_689:rev								
MCE-MIR_774:rev					30	22		

GCNF-/- Time:	Day 0		Day 1		Day 3		Day 6	
**Array Chip ID:**	**204**	**210**	**124**	**217**	**356**	**357**	**245**	**246**
MCE-MIR_809:fwd		17			60	36		44
MCE-MIR_81:fwd					18			33
MCE-MIR_855:fwd					66	43		29
MCE-MIR_855:rev					23			
MCE-MIR_871:rev								
MCE-MIR_946:rev					27	34		
MIR100								
MIR122								
MIR140								
MIR161								
MIR167						27		43
MIR169								
MIR177								
MIR180								
MIR184					29		14	30
MIR188								
MIR201				17	27			34
MIR220								36
MIR237								
MIR255								35
MIR41				24				42
MIR47								
MIR52								
MIR71								
MIR77					58	39	45	78
mmu-mir-10a			48	364	752	766	408	269
mmu-mir-10b				32	299	179	132	122
mmu-mir-126-5p								
mmu-mir-135a								
mmu-mir-135b								
mmu-mir-136								
mmu-mir-137								46
mmu-mir-138								
mmu-mir-139								
mmu-mir-140								
mmu-mir-141								
mmu-mir-142-3p								
mmu-mir-144								

GCNF-/- Time:	Day 0		Day 1		Day 3		Day 6	
**Array Chip ID:**	**204**	**210**	**124**	**217**	**356**	**357**	**245**	**246**
mmu-mir-153								
mmu-mir-189						14		
mmu-mir-190								
mmu-mir-192								64
mmu-mir-196a					37	31		
mmu-mir-196b								
mmu-mir-201								
mmu-mir-202						25		
mmu-mir-208								
mmu-mir-213								
mmu-mir-215								
mmu-mir-216								
mmu-mir-217								
mmu-mir-219								
S-mmu-mir-302b								
S-mmu-mir-30e								
mmu-mir-32								
mmu-mir-325								
mmu-mir-33								
mmu-mir-338								
mmu-mir-340								
mmu-mir-34b								
mmu-mir-365				25	57	29		
mmu-mir-367								
mmu-mir-369-3p								
mmu-mir-369-5p								
S-mmu-mir-376a								
S-mmu-mir-376b								
mmu-mir-376c								
mmu-mir-380-5p								
mmu-mir-384								
mmu-mir-433-5p					29			
mmu-mir-448								
mmu-mir-449								
mmu-mir-463								
mmu-mir-464								
mmu-mir-465								
mmu-mir-469								

GCNF-/- Time:	Day 0		Day 1		Day 3		Day 6	
**Array Chip ID:**	**204**	**210**	**124**	**217**	**356**	**357**	**245**	**246**
mmu-mir-471								
mmu-mir-489				23	24			
mmu-mir-539				29	26			
mmu-mir-542-3p								
mmu-mir-542-5p					51	32		
mmu-mir-547								
mmu-mir-7b								
S-mmu-let-7a-1		13		26	19			37
S-mmu-let-7a-2		13		32	303	30		36
S-mmu-let-7b		21			130	22		62
S-mmu-let-7c-1								
S-mmu-let-7c-2								
S-mmu-let-7e					113	60		37
S-mmu-let-7f-1		18						
S-mmu-let-7f-2								
S-mmu-let-7g		23		46				46
S-mmu-let-7i		20		27				65
S-mmu-mir-100								
S-mmu-mir-101a								
S-mmu-mir-101b								
S-mmu-mir-103-1		30		57		34		71
S-mmu-mir-103-2				17		18		39
S-mmu-mir-106a		21		35	22	26		
S-mmu-mir-106b		412		1,122	481	351	252	189
S-mmu-mir-107		23		24				48
S-mmu-mir-10a		16		58	58	32	19	53
S-mmu-mir-10b		26		139	52		57	64
S-mmu-mir-1-1		94		121	37		33	81
S-mmu-mir-1-2		14						52
S-mmu-mir-122a								57
S-mmu-mir-124a-1		31		36	18	45	15	
S-mmu-mir-124a-2		32		32		37		56
S-mmu-mir-124a-3		32		40	17	40		55
S-mmu-mir-125a		39		129	158	119	55	77
S-mmu-mir-125b-1		30		63	231	141		57
S-mmu-mir-125b-2		13						60
S-mmu-mir-127		21		36	43	35		70
S-mmu-mir-128a					94	69		38

GCNF-/- Time:	Day 0		Day 1		Day 3		Day 6	
**Array Chip ID:**	**204**	**210**	**124**	**217**	**356**	**357**	**245**	**246**
S-mmu-mir-128b		987		659	11,124	6,169	22	54
S-mmu-mir-129-1		21		33	61	42	11	80
S-mmu-mir-130a								
S-mmu-mir-132								46
S-mmu-mir-133a-1								58
S-mmu-mir-133a-2								64
S-mmu-mir-133b		466		732	1,398			1,208
S-mmu-mir-134		22		19		20		50
S-mmu-mir-135a-1		24				34		40
S-mmu-mir-135a-2		29			58	59		50
S-mmu-mir-137		13						31
S-mmu-mir-138-1						28		44
S-mmu-mir-138-2		20			34	50		47
S-mmu-mir-139		19			42	34		34
S-mmu-mir-141		12						54
S-mmu-mir-143		16						53
S-mmu-mir-144								51
S-mmu-mir-145		15						71
S-mmu-mir-146								42
S-mmu-mir-148a		14						49
S-mmu-mir-148b								52
S-mmu-mir-149		32,484		31,725	48,968	27,731	26,802	82,672
S-mmu-mir-150		6,648		8,323	9,595	3,195	4,733	5,961
S-mmu-mir-151		466		1,045	677	468	265	283
S-mmu-mir-154								60
S-mmu-mir-15a		30		51	35	35	26	111
S-mmu-mir-15b								65
S-mmu-mir-16-1		34		27	55	49		76
S-mmu-mir-16-2								62
S-mmu-mir-18		49		43	40	48		78
S-mmu-mir-181a				19	53	35	19	43
S-mmu-mir-181c		19		38	18	17		
S-mmu-mir-182								32
S-mmu-mir-183		172		432	313	176	37	38
S-mmu-mir-184		54		80	55	46	30	73
S-mmu-mir-185				19	45	42		
S-mmu-mir-186								
S-mmu-mir-187		44		142	95	78		

GCNF-/- Time:	Day 0		Day 1		Day 3		Day 6	
**Array Chip ID:**	**204**	**210**	**124**	**217**	**356**	**357**	**245**	**246**
S-mmu-mir-188		17		36	77	45		47
S-mmu-mir-190								30
S-mmu-mir-191								
S-mmu-mir-192								
S-mmu-mir-193		32		67	147	110		36
S-mmu-mir-194-1					29	27		30
S-mmu-mir-194-2		60		144	502	400		31
S-mmu-mir-195				18				34
S-mmu-mir-196a-1		12						35
S-mmu-mir-196a-2				31				47
S-mmu-mir-196b		46		88	89	80		82
S-mmu-mir-199b		32		146		37	214	663
S-mmu-mir-20					15			41
S-mmu-mir-200a		33		59	75	49		42
S-mmu-mir-200b		51		137	133	76		38
S-mmu-mir-204		953		1,968	8,145	2,750	161	212
S-mmu-mir-207		21,778		38,057	41,075	23,346	18,995	38,435
S-mmu-mir-208		104		242	408	128		48
S-mmu-mir-21		3,162		9,940	10,083	3,016	3,553	4,791
S-mmu-mir-210		119		299	750	353	90	174
S-mmu-mir-211		5,319		12,359	17,017	10,173	11,819	16,519
S-mmu-mir-212					390			48
S-mmu-mir-214								46
S-mmu-mir-218-1		32		28	81	69	17	61
S-mmu-mir-218-2		41		52	93	82		64
S-mmu-mir-219-2		21		180	93	53		53
S-mmu-mir-22		14		38	47	40		82
S-mmu-mir-221								35
S-mmu-mir-223								
S-mmu-mir-23a		251		631	1,218	687	64	76
S-mmu-mir-23b		51		59	42	43		
S-mmu-mir-24-2		88		315	63	53	27	73
S-mmu-mir-25		290		340	510	336		31
S-mmu-mir-26a-1								28
S-mmu-mir-27a		36		120	46	39		
S-mmu-mir-27b		26		33		30		29
S-mmu-mir-28		69		200	210	143	62	110
S-mmu-mir-290		108		178	411	174		

GCNF-/- Time:	Day 0		Day 1		Day 3		Day 6	
Array Chip ID:	204	210	124	217	356	357	245	246
S-mmu-mir-293		756		3,050	65	73	23	59
S-mmu-mir-294		517		2,171	1,220	574	63	53
S-mmu-mir-295		1,334		3,592	1,286	731	443	197
S-mmu-mir-296		1,858		3,154	5,223	2,523	649	428
S-mmu-mir-298		13			23	21		49
S-mmu-mir-29a								40
S-mmu-mir-29b-1								42
S-mmu-mir-29b-2					17	23		45
S-mmu-mir-300						21		39
S-mmu-mir-30b		22		21	67	74		48
S-mmu-mir-30c-1		1,845		618	7,681	4,423		51
S-mmu-mir-30c-2		20		25	145	149		62
S-mmu-mir-31								70
S-mmu-mir-32					31	52	7	
S-mmu-mir-323		22			316	154		39
S-mmu-mir-326		4,316		5,683	16,328	9,108	1,036	576
S-mmu-mir-328		14,208		20,651	35,079	22,003	13,384	16,728
S-mmu-mir-329		42		55	36	25		35
S-mmu-mir-33								
S-mmu-mir-330		15		31				
S-mmu-mir-331						40		31
S-mmu-mir-335								
S-mmu-mir-337		112		330	71	63	68	56
S-mmu-mir-338								
S-mmu-mir-339				28		17		55
S-mmu-mir-342		53		153	219	116	94	90
S-mmu-mir-345		34		123	92	60		56
S-mmu-mir-346		6,945		11,697	16,518	10,772	3,733	2,870
S-mmu-mir-34a		67		153	129	69		80
S-mmu-mir-34b		31		113	52	49		142
S-mmu-mir-34c		94		188	201	145	57	192
S-mmu-mir-350								30
S-mmu-mir-351		33		70	68	58	30	76
S-mmu-mir-361		27		62	114	69		61
S-mmu-mir-363		185		302	1,086	136		42
S-mmu-mir-365-1		10,026		15,134	21,327	9,434	5,980	8,452
S-mmu-mir-365-2		8,798		14,374	18,879	7,482	2,612	4,453
S-mmu-mir-370								41

GCNF-/- Time:	Day 0		Day 1		Day 3		Day 6	
**Array Chip ID:**	**204**	**210**	**124**	**217**	**356**	**357**	**245**	**246**
S-mmu-mir-377		17			21			28
S-mmu-mir-378		358		404	115	54	102	384
S-mmu-mir-379		17		42	33			47
S-mmu-mir-381		14				17		30
S-mmu-mir-382								52
S-mmu-mir-384							109	43
S-mmu-mir-409						24		
S-mmu-mir-412				32	69	36		46
S-mmu-mir-425		164		413	238	113	39	129
S-mmu-mir-431		67		380	227	101	50	91
S-mmu-mir-448								29
S-mmu-mir-449					37	42		37
S-mmu-mir-451								
S-mmu-mir-465				16	43	26		67
S-mmu-mir-466				15	45	24		
S-mmu-mir-467		968		3,390	3,789	1,989	174	152
S-mmu-mir-468								46
S-mmu-mir-469		30,043		38,060	36,801	19,989	26,652	73,971
S-mmu-mir-471								33
S-mmu-mir-483		582		1,145	2,665	826	1,194	1,045
S-mmu-mir-484		10,130		17,921	15,896	7,407	18,893	31,280
S-mmu-mir-486		46		28	228	144		46
S-mmu-mir-7-1		18		40				92
S-mmu-mir-7-2		65		110	94	65	30	106
S-mmu-mir-7b		99		133	174	81	68	150
S-mmu-mir-92-1				15	35	32		41
S-mmu-mir-92-2		368		576	6,135	3,652	25	60
S-mmu-mir-93		80		117	72	39	35	79
S-mmu-mir-96								41
S-mmu-mir-98								49
S-mmu-mir-99a						13		44
S-mmu-mir-99b		47		172	113	78	60	108
