# Supplementary material for: Novel MicroRNA Candidates and miRNA-mRNA Pairs in Embryonic Stem (ES) Cells
Source: PLoS One. 2008 Jul 2;3(7):e2548. doi: 10.1371/journal.pone.0002548 (PMC2481296; doi:10.1371/journal.pone.0002548)
Supplement: Table S5 — ES-GCNF Time Series. This table shows normalized microarray data for ES (ST5A) and GCNF−/− (ST5B). In each case the values have been normalized and interpolated. The actual experiment was carried out with RNA isolated from the ES cells from Day 0, Day 1, Day 3 and Day 6. Values for other days (Days 2, 4 and 5) have been linearly interpolated from these. (0.39 MB PDF) [file pone.0002548.s012.pdf]

Supplemental Table ST5A: ES-GCNF Time Series ES. This table shows the data for the ES time series. In each case the values have been normalized and interpolated. The actual experiment was carried out with RNA isolated from the ES cells from Day 0, Day 1, Day 3 and Day 6. Values for other days (Days 2, 4 and 5) have been linearly interpolated from these.

Class	ES Time Pattern	ProbeID	ES-Day0	ES-Day1	ES-Day2	ES-Day3	ES-Day4	ES-Day5	ES-Day6
1	Changed	MCE-MIR_810:rev	2.10	0.77	0.04	-0.70	-0.72	-0.74	-0.75
1	Changed	MCE-MIR_5389:fwd	0.56	0.64	0.61	0.58	-0.11	-0.80	-1.49
1	Changed	MCE-MIR_4740:fwd	0.75	1.04	0.68	0.32	-0.30	-0.93	-1.56
1	Changed	MCE-MIR_4673:fwd	0.54	0.89	0.53	0.18	-0.27	-0.71	-1.16
1	Changed	MCE-MIR_3859:fwd	0.79	0.78	0.72	0.66	-0.16	-0.98	-1.80
1	Changed	MCE-MIR_3847:fwd	0.47	0.59	0.62	0.64	-0.07	-0.77	-1.48
1	Changed	MCE-MIR_3513:rev	0.19	0.82	0.69	0.55	-0.10	-0.75	-1.40
1	Changed	MCE-MIR_3111:fwd	0.26	0.62	0.42	0.22	-0.14	-0.51	-0.87
1	Changed	MCE-MIR_2310:rev	0.35	0.32	0.43	0.54	0.00	-0.55	-1.09
1	Changed	MCE-MIR_2111:fwd	0.30	0.51	0.25	-0.01	-0.18	-0.35	-0.52
1	Not Changed	MCE-MIR_6107:fwd	0.28	0.36	0.29	0.23	-0.08	-0.38	-0.69
1	Not Changed	MCE-MIR_6026:rev	0.61	0.63	0.64	0.66	-0.09	-0.85	-1.60
1	Not Changed	MCE-MIR_5470:fwd	1.20	0.75	0.55	0.34	-0.30	-0.95	-1.59
1	Not Changed	MCE-MIR_4932:rev	0.31	0.15	0.03	-0.09	-0.11	-0.14	-0.16
1	Not Changed	MCE-MIR_4922:fwd	0.69	0.69	0.73	0.76	-0.10	-0.96	-1.82
1	Not Changed	MCE-MIR_4791:rev	0.34	0.73	0.32	-0.10	-0.27	-0.43	-0.60
1	Not Changed	MCE-MIR_4493:fwd	0.47	0.37	0.10	-0.17	-0.21	-0.26	-0.31
1	Not Changed	MCE-MIR_2999:fwd	0.49	0.20	0.36	0.52	0.00	-0.52	-1.05
1	Not Changed	MCE-MIR_2337:fwd	0.41	0.50	0.33	0.17	-0.15	-0.47	-0.79
1	Not Changed	MCE-MIR_1784:fwd	0.50	0.64	0.72	0.80	-0.05	-0.89	-1.73
1	Not Changed	MCE-MIR_1514:fwd	0.12	0.33	0.37	0.41	0.00	-0.41	-0.82
2	Changed	MCE-MIR_5216:rev	-1.04	-0.77	0.10	0.98	0.61	0.24	-0.13
2	Changed	MCE-MIR_5192:rev	-0.52	-0.10	0.19	0.48	0.23	-0.01	-0.26
2	Changed	MCE-MIR_4791:fwd	0.04	0.39	0.48	0.56	0.04	-0.49	-1.02
2	Changed	MCE-MIR_4674:fwd	-1.26	-1.14	0.12	1.37	0.84	0.30	-0.23
2	Changed	MCE-MIR_4153:fwd	-1.24	-0.76	0.26	1.27	0.71	0.15	-0.41
2	Changed	MCE-MIR_3820:rev	-0.46	-0.08	0.07	0.22	0.15	0.08	0.01
2	Changed	MCE-MIR_3474:rev	-0.86	-0.37	0.17	0.72	0.42	0.11	-0.19
2	Changed	MCE-MIR_329:fwd	-0.99	-0.03	0.22	0.46	0.29	0.11	-0.06
2	Changed	MCE-MIR_298:rev	-0.88	-0.31	0.37	1.05	0.49	-0.08	-0.64
2	Changed	MCE-MIR_2714:rev	-1.18	-0.02	0.44	0.89	0.43	-0.04	-0.51
2	Changed	MCE-MIR_1539:rev	-0.35	-0.10	0.07	0.24	0.14	0.05	-0.05

Class	ES Time Pattern	ProbeID	ES-Day0	ES-Day1	ES-Day2	ES-Day3	ES-Day4	ES-Day5	ES-Day6
2	Changed	MCE-MIR_1457:rev	-0.30	-0.06	0.09	0.24	0.12	0.01	-0.11
2	Changed	MCE-MIR_1371:fwd	-0.78	-0.24	0.22	0.68	0.36	0.04	-0.28
2	Changed	MCE-MIR_1046:rev	-0.26	-0.25	0.03	0.31	0.18	0.06	-0.07
2	Not Changed	MCE-MIR_783:rev	-0.15	0.22	0.28	0.33	0.05	-0.22	-0.50
2	Not Changed	MCE-MIR_5914:rev	-1.42	0.44	0.70	0.96	0.37	-0.22	-0.81
2	Not Changed	MCE-MIR_5745:fwd	-0.73	-0.13	0.54	1.21	0.46	-0.30	-1.05
2	Not Changed	MCE-MIR_5276:fwd	-0.58	0.21	0.60	0.99	0.29	-0.41	-1.10
2	Not Changed	MCE-MIR_5152:rev	0.13	0.24	0.46	0.68	0.09	-0.50	-1.09
2	Not Changed	MCE-MIR_5005:rev	-0.31	-0.07	0.09	0.25	0.13	0.01	-0.11
2	Not Changed	MCE-MIR_4627:fwd	-0.32	0.07	0.47	0.88	0.26	-0.37	-0.99
2	Not Changed	MCE-MIR_4610:fwd	-1.14	-0.18	0.87	1.93	0.72	-0.50	-1.71
2	Not Changed	MCE-MIR_4198:fwd	-0.03	0.24	0.39	0.54	0.08	-0.38	-0.85
2	Not Changed	MCE-MIR_3685:fwd	-0.37	0.09	0.55	1.02	0.30	-0.43	-1.16
2	Not Changed	MCE-MIR_3492:fwd	-0.33	-0.70	0.21	1.13	0.51	-0.10	-0.72
2	Not Changed	MCE-MIR_3143:fwd	-0.28	0.03	0.19	0.35	0.13	-0.09	-0.31
2	Not Changed	MCE-MIR_2986:fwd	-0.04	-0.20	0.56	1.33	0.39	-0.55	-1.49
2	Not Changed	MCE-MIR_2815:fwd	-0.13	-0.06	0.19	0.43	0.14	-0.14	-0.43
2	Not Changed	MCE-MIR_2285:fwd	-0.15	-0.11	0.38	0.86	0.27	-0.33	-0.93
2	Not Changed	MCE-MIR_1986:fwd	-0.73	0.07	0.41	0.76	0.29	-0.17	-0.64
2	Not Changed	MCE-MIR_1857:fwd	-0.55	0.30	0.78	1.27	0.33	-0.60	-1.53
2	Not Changed	MCE-MIR_1734:fwd	-0.22	-0.07	0.36	0.79	0.25	-0.29	-0.82
2	Not Changed	MCE-MIR_1506:rev	-0.59	-0.11	0.13	0.36	0.22	0.07	-0.08
2	Not Changed	MCE-MIR_136:fwd	-0.10	0.06	0.16	0.25	0.06	-0.12	-0.31
2	Not Changed	MCE-MIR_135:fwd	-0.31	0.15	0.28	0.40	0.11	-0.17	-0.46
2	Not Changed	MCE-MIR_1074:rev	0.13	-0.27	0.57	1.40	0.40	-0.61	-1.62
3	Changed	MCE-MIR_3477:rev	-2.17	-0.84	0.01	0.86	0.78	0.71	0.64
3	Changed	MCE-MIR_3191:rev	-0.30	-0.25	-0.11	0.03	0.12	0.21	0.30
3	Changed	MCE-MIR_3190:rev	-0.24	-0.23	-0.08	0.06	0.11	0.16	0.21
3	Not Changed	MCE-MIR_4297:fwd	-0.50	-0.42	-0.12	0.18	0.23	0.29	0.34
3	Not Changed	MCE-MIR_3478:rev	-0.74	-0.74	-0.32	0.09	0.33	0.57	0.81
3	Not Changed	MCE-MIR_3470:rev	-0.56	-0.45	-0.14	0.17	0.25	0.33	0.41
3	Not Changed	MCE-MIR_3468:rev	-0.33	-0.26	-0.10	0.06	0.13	0.21	0.28
4	Changed	MCE-MIR_968:fwd	-0.05	0.01	0.03	0.04	0.02	-0.01	-0.03
4	Changed	MCE-MIR_89:fwd	-0.01	-0.08	-0.02	0.04	0.03	0.02	0.01
4	Changed	MCE-MIR_782:rev	0.06	0.01	0.00	-0.02	-0.02	-0.02	-0.01
4	Changed	MCE-MIR_774:rev	0.08	-0.02	-0.01	-0.01	-0.01	-0.01	-0.02
4	Changed	MCE-MIR_645:fwd	-0.26	0.07	0.14	0.22	0.08	-0.06	-0.19
4	Changed	MCE-MIR_638:fwd	0.01	0.17	0.25	0.34	0.04	-0.26	-0.55

Class	ES Time Pattern	ProbeID	ES-Day0	ES-Day1	ES-Day2	ES-Day3	ES-Day4	ES-Day5	ES-Day6
4	Changed	MCE-MIR_6054:rev	0.71	-0.23	-0.20	-0.17	-0.10	-0.03	0.03
4	Changed	MCE-MIR_6034:rev	0.21	-0.02	-0.02	-0.03	-0.04	-0.05	-0.05
4	Changed	MCE-MIR_5712:rev	0.17	-0.11	-0.09	-0.06	-0.01	0.03	0.07
4	Changed	MCE-MIR_5620:fwd	0.22	-0.07	-0.09	-0.10	-0.04	0.02	0.08
4	Changed	MCE-MIR_5374:rev	0.04	0.04	-0.01	-0.05	-0.03	-0.01	0.02
4	Changed	MCE-MIR_5322:rev	0.04	0.00	0.02	0.03	0.00	-0.03	-0.06
4	Changed	MCE-MIR_5303:fwd	-0.01	-0.07	-0.03	0.01	0.02	0.03	0.04
4	Changed	MCE-MIR_5300:rev	-0.01	0.18	0.05	-0.09	-0.06	-0.04	-0.02
4	Changed	MCE-MIR_5062:rev	-0.33	-0.06	0.08	0.23	0.13	0.03	-0.07
4	Changed	MCE-MIR_5060:rev	-0.37	-0.11	0.06	0.23	0.15	0.06	-0.02
4	Changed	MCE-MIR_4861:fwd	-0.15	0.08	0.15	0.21	0.06	-0.10	-0.25
4	Changed	MCE-MIR_4716:fwd	-0.28	-0.30	0.06	0.42	0.23	0.04	-0.16
4	Changed	MCE-MIR_4497:rev	0.27	-0.07	0.04	0.15	0.01	-0.13	-0.27
4	Changed	MCE-MIR_4491:rev	0.07	0.07	0.01	-0.06	-0.04	-0.03	-0.02
4	Changed	MCE-MIR_4179:rev	-0.32	-0.26	-0.02	0.22	0.17	0.13	0.08
4	Changed	MCE-MIR_3503:rev	-0.48	-0.08	0.04	0.16	0.14	0.12	0.10
4	Changed	MCE-MIR_3429:fwd	0.18	-0.14	-0.02	0.10	0.03	-0.04	-0.11
4	Changed	MCE-MIR_3416:rev	-0.80	-0.29	-0.05	0.19	0.25	0.32	0.38
4	Changed	MCE-MIR_3379:fwd	0.04	-0.03	-0.02	-0.01	0.00	0.00	0.01
4	Changed	MCE-MIR_334:rev	0.06	0.13	0.05	-0.03	-0.05	-0.07	-0.09
4	Changed	MCE-MIR_3226:fwd	-0.07	-0.06	-0.01	0.05	0.04	0.03	0.02
4	Changed	MCE-MIR_3048:rev	-0.04	-0.05	-0.01	0.03	0.03	0.02	0.02
4	Changed	MCE-MIR_293:fwd	-0.12	-0.20	0.03	0.26	0.13	0.01	-0.12
4	Changed	MCE-MIR_2902:rev	-0.40	-0.11	0.03	0.16	0.13	0.11	0.08
4	Changed	MCE-MIR_2902:fwd	0.16	-0.14	-0.06	0.03	0.02	0.00	-0.01
4	Changed	MCE-MIR_2563:fwd	0.25	-1.19	-0.71	-0.23	0.20	0.62	1.05
4	Changed	MCE-MIR_2524:fwd	-0.41	-0.16	-0.12	-0.09	0.09	0.26	0.44
4	Changed	MCE-MIR_2349:rev	0.49	0.06	0.02	-0.03	-0.10	-0.18	-0.25
4	Changed	MCE-MIR_2169:rev	-0.11	-0.09	-0.04	0.02	0.05	0.08	0.10
4	Changed	MCE-MIR_1931:fwd	0.05	-0.11	-0.01	0.10	0.05	-0.01	-0.07
4	Changed	MCE-MIR_18:fwd	-0.12	0.07	0.10	0.13	0.04	-0.06	-0.16
4	Changed	MCE-MIR_1788:rev	0.56	-0.20	-0.11	-0.02	-0.05	-0.08	-0.11
4	Changed	MCE-MIR_1788:fwd	0.27	-0.05	-0.02	0.00	-0.03	-0.07	-0.10
4	Changed	MCE-MIR_1773:fwd	-0.39	-0.19	0.03	0.25	0.17	0.10	0.03
4	Changed	MCE-MIR_1746:rev	0.67	-0.51	-0.37	-0.22	-0.04	0.14	0.33
4	Changed	MCE-MIR_1504:rev	-0.41	-0.06	0.07	0.20	0.13	0.06	-0.01
4	Changed	MCE-MIR_1394:rev	-0.22	0.14	0.18	0.22	0.06	-0.11	-0.28
4	Changed	MCE-MIR_1226:fwd	0.07	0.17	0.08	-0.02	-0.06	-0.10	-0.14

Class	ES Time Pattern	ProbeID	ES-Day0	ES-Day1	ES-Day2	ES-Day3	ES-Day4	ES-Day5	ES-Day6
4	Not Changed	MCE-MIR_995:rev	-0.23	-0.02	0.06	0.14	0.08	0.02	-0.05
4	Not Changed	MCE-MIR_993:rev	-0.17	-0.28	-0.19	-0.10	0.07	0.25	0.43
4	Not Changed	MCE-MIR_988:fwd	0.28	-0.21	-0.25	-0.29	-0.07	0.16	0.38
4	Not Changed	MCE-MIR_984:rev	-0.22	-0.14	-0.02	0.09	0.10	0.10	0.10
4	Not Changed	MCE-MIR_959:rev	0.12	0.05	0.03	0.01	-0.03	-0.07	-0.11
4	Not Changed	MCE-MIR_946:rev	0.11	0.01	-0.01	-0.02	-0.03	-0.03	-0.04
4	Not Changed	MCE-MIR_942:rev	-0.20	-0.11	0.05	0.22	0.12	0.01	-0.09
4	Not Changed	MCE-MIR_936:rev	-0.23	-0.07	0.01	0.10	0.08	0.07	0.05
4	Not Changed	MCE-MIR_871:rev	0.05	0.02	0.01	-0.01	-0.02	-0.02	-0.03
4	Not Changed	MCE-MIR_871:fwd	0.09	0.09	-0.02	-0.13	-0.07	-0.01	0.05
4	Not Changed	MCE-MIR_855:rev	-0.01	0.05	0.00	-0.04	-0.02	0.00	0.02
4	Not Changed	MCE-MIR_855:fwd	0.00	0.01	0.02	0.02	0.00	-0.01	-0.03
4	Not Changed	MCE-MIR_822:fwd	-0.03	0.39	0.39	0.38	0.00	-0.38	-0.76
4	Not Changed	MCE-MIR_81:fwd	-0.02	0.01	-0.01	-0.02	-0.01	0.01	0.03
4	Not Changed	MCE-MIR_809:fwd	0.05	0.06	0.03	0.00	-0.02	-0.05	-0.07
4	Not Changed	MCE-MIR_780:rev	0.31	-0.17	0.14	0.45	0.10	-0.24	-0.59
4	Not Changed	MCE-MIR_777:rev	-0.07	-0.30	0.20	0.69	0.26	-0.18	-0.61
4	Not Changed	MCE-MIR_755:fwd	-1.40	-0.65	-0.19	0.28	0.47	0.65	0.84
4	Not Changed	MCE-MIR_734:fwd	0.41	-0.01	-0.10	-0.20	-0.12	-0.03	0.05
4	Not Changed	MCE-MIR_725:fwd	0.08	-0.26	-0.23	-0.20	0.00	0.20	0.41
4	Not Changed	MCE-MIR_689:rev	0.01	-0.02	-0.01	-0.01	0.00	0.01	0.02
4	Not Changed	MCE-MIR_670:fwd	0.35	0.12	0.27	0.42	0.02	-0.39	-0.79
4	Not Changed	MCE-MIR_6120:fwd	0.00	0.02	-0.01	-0.05	-0.02	0.02	0.05
4	Not Changed	MCE-MIR_6084:rev	-0.01	0.14	0.06	-0.02	-0.04	-0.05	-0.07
4	Not Changed	MCE-MIR_6055:rev	0.15	-0.01	0.03	0.07	0.00	-0.07	-0.14
4	Not Changed	MCE-MIR_6050:rev	-0.48	-0.71	-0.03	0.65	0.42	0.19	-0.04
4	Not Changed	MCE-MIR_6033:fwd	0.06	-0.04	-0.01	0.02	0.01	-0.01	-0.03
4	Not Changed	MCE-MIR_6026:fwd	0.08	-0.07	-0.03	0.01	0.00	0.00	0.00
4	Not Changed	MCE-MIR_6001:rev	-0.09	-0.05	0.03	0.12	0.06	0.00	-0.07
4	Not Changed	MCE-MIR_5970:rev	-0.03	0.02	0.01	0.00	0.00	0.00	0.00
4	Not Changed	MCE-MIR_5872:fwd	-0.63	0.20	0.27	0.33	0.14	-0.06	-0.25
4	Not Changed	MCE-MIR_5864:fwd	-0.08	0.02	0.01	0.00	0.01	0.02	0.03
4	Not Changed	MCE-MIR_5790:fwd	-0.81	-0.02	0.48	0.97	0.38	-0.20	-0.79
4	Not Changed	MCE-MIR_5736:rev	0.23	-0.07	-0.04	-0.01	-0.02	-0.04	-0.05
4	Not Changed	MCE-MIR_5704:rev	0.00	0.00	-0.05	-0.10	-0.03	0.05	0.13
4	Not Changed	MCE-MIR_5699:fwd	0.32	0.52	0.33	0.14	-0.15	-0.43	-0.72
4	Not Changed	MCE-MIR_5643:fwd	0.57	-0.16	0.12	0.41	0.05	-0.32	-0.68
4	Not Changed	MCE-MIR_5641:rev	0.37	-0.16	-0.02	0.11	0.01	-0.10	-0.20

Class	ES Time Pattern	ProbeID	ES-Day0	ES-Day1	ES-Day2	ES-Day3	ES-Day4	ES-Day5	ES-Day6
4	Not Changed	MCE-MIR_5623:rev	0.09	-0.03	-0.05	-0.07	-0.03	0.01	0.05
4	Not Changed	MCE-MIR_5607:rev	-0.16	0.91	0.95	0.99	0.05	-0.90	-1.84
4	Not Changed	MCE-MIR_5606:fwd	-0.19	0.07	0.13	0.19	0.06	-0.07	-0.20
4	Not Changed	MCE-MIR_5598:fwd	0.07	0.04	0.01	-0.01	-0.02	-0.04	-0.05
4	Not Changed	MCE-MIR_5597:fwd	0.29	0.02	-0.05	-0.12	-0.08	-0.05	-0.01
4	Not Changed	MCE-MIR_5596:rev	0.20	0.36	0.25	0.15	-0.08	-0.32	-0.56
4	Not Changed	MCE-MIR_5581:rev	0.02	-0.07	-0.05	-0.04	0.00	0.05	0.09
4	Not Changed	MCE-MIR_5581:fwd	0.05	0.02	-0.02	-0.07	-0.03	0.01	0.05
4	Not Changed	MCE-MIR_557:fwd	0.38	-0.30	-0.23	-0.16	-0.03	0.10	0.24
4	Not Changed	MCE-MIR_5544:rev	0.56	0.28	0.28	0.27	-0.09	-0.46	-0.83
4	Not Changed	MCE-MIR_5511:rev	0.41	0.48	0.39	0.29	-0.12	-0.53	-0.94
4	Not Changed	MCE-MIR_5504:rev	0.13	0.02	-0.02	-0.06	-0.04	-0.02	-0.01
4	Not Changed	MCE-MIR_5503:rev	0.75	0.18	0.53	0.88	0.05	-0.78	-1.61
4	Not Changed	MCE-MIR_5488:rev	0.37	-0.14	-0.20	-0.26	-0.09	0.08	0.24
4	Not Changed	MCE-MIR_5473:rev	0.36	-0.13	-0.17	-0.22	-0.08	0.05	0.19
4	Not Changed	MCE-MIR_5473:fwd	0.50	-0.03	0.09	0.21	-0.02	-0.26	-0.49
4	Not Changed	MCE-MIR_5454:rev	0.29	-0.41	-0.28	-0.15	0.02	0.19	0.36
4	Not Changed	MCE-MIR_5443:fwd	-0.76	-1.25	-0.44	0.38	0.53	0.69	0.84
4	Not Changed	MCE-MIR_5440:fwd	0.19	0.27	0.41	0.56	0.04	-0.48	-1.00
4	Not Changed	MCE-MIR_543:fwd	-0.15	-0.41	-0.07	0.28	0.20	0.12	0.04
4	Not Changed	MCE-MIR_5418:fwd	0.44	-0.03	0.34	0.71	0.11	-0.49	-1.08
4	Not Changed	MCE-MIR_5411:rev	0.44	-0.15	0.29	0.72	0.14	-0.44	-1.02
4	Not Changed	MCE-MIR_5406:rev	-0.07	0.11	0.01	-0.08	-0.03	0.01	0.06
4	Not Changed	MCE-MIR_5403:fwd	0.25	0.36	0.45	0.54	0.00	-0.54	-1.08
4	Not Changed	MCE-MIR_5399:fwd	-0.48	-0.45	-0.21	0.03	0.20	0.36	0.53
4	Not Changed	MCE-MIR_5396:rev	-0.06	0.08	0.02	-0.03	-0.02	-0.01	0.01
4	Not Changed	MCE-MIR_5389:rev	-0.14	0.07	0.02	-0.03	0.00	0.03	0.05
4	Not Changed	MCE-MIR_5384:rev	-0.05	0.34	0.54	0.74	0.11	-0.53	-1.16
4	Not Changed	MCE-MIR_5374:fwd	-0.83	-0.70	-0.21	0.28	0.38	0.48	0.59
4	Not Changed	MCE-MIR_5369:rev	-0.50	0.13	0.28	0.43	0.16	-0.11	-0.39
4	Not Changed	MCE-MIR_5367:rev	-0.35	-0.12	-0.03	0.06	0.10	0.15	0.19
4	Not Changed	MCE-MIR_5366:rev	-0.69	-0.68	-0.25	0.18	0.33	0.48	0.63
4	Not Changed	MCE-MIR_5363:rev	-1.19	-0.86	-0.38	0.09	0.44	0.78	1.13
4	Not Changed	MCE-MIR_5363:fwd	-0.72	0.50	0.51	0.53	0.13	-0.27	-0.68
4	Not Changed	MCE-MIR_5354:rev	0.49	-0.26	-0.01	0.23	0.04	-0.15	-0.34
4	Not Changed	MCE-MIR_5340:fwd	-0.12	-0.40	0.05	0.51	0.25	-0.01	-0.27
4	Not Changed	MCE-MIR_534:fwd	0.51	0.17	0.49	0.80	0.07	-0.66	-1.39
4	Not Changed	MCE-MIR_5339:rev	-0.08	0.30	0.19	0.08	-0.04	-0.17	-0.29

Class	ES Time Pattern	ProbeID	ES-Day0	ES-Day1	ES-Day2	ES-Day3	ES-Day4	ES-Day5	ES-Day6
4	Not Changed	MCE-MIR_5328:rev	0.22	-0.32	0.35	1.03	0.30	-0.43	-1.16
4	Not Changed	MCE-MIR_5322:fwd	0.17	-0.20	-0.20	-0.19	-0.03	0.14	0.31
4	Not Changed	MCE-MIR_5300:fwd	0.15	-0.24	-0.18	-0.12	0.00	0.13	0.26
4	Not Changed	MCE-MIR_530:fwd	-0.14	0.50	0.58	0.67	0.07	-0.54	-1.14
4	Not Changed	MCE-MIR_5295:rev	0.26	-0.29	-0.20	-0.11	0.00	0.11	0.23
4	Not Changed	MCE-MIR_5291:fwd	0.54	0.68	0.57	0.45	-0.15	-0.75	-1.35
4	Not Changed	MCE-MIR_5287:rev	-0.12	-0.04	-0.02	-0.01	0.03	0.06	0.09
4	Not Changed	MCE-MIR_5279:fwd	-0.07	0.07	0.03	0.00	-0.01	-0.01	-0.01
4	Not Changed	MCE-MIR_5260:fwd	-0.05	0.10	0.02	-0.05	-0.03	0.00	0.02
4	Not Changed	MCE-MIR_5236:rev	-0.04	0.18	0.06	-0.05	-0.05	-0.05	-0.05
4	Not Changed	MCE-MIR_5210:fwd	-0.09	-0.14	-0.13	-0.12	0.02	0.16	0.30
4	Not Changed	MCE-MIR_5197:rev	-0.34	-0.04	0.04	0.12	0.10	0.07	0.05
4	Not Changed	MCE-MIR_5195:rev	-0.51	-0.18	0.05	0.28	0.20	0.12	0.04
4	Not Changed	MCE-MIR_5193:rev	-0.14	-0.08	0.01	0.11	0.07	0.03	-0.01
4	Not Changed	MCE-MIR_5180:rev	0.03	0.08	0.03	-0.01	-0.03	-0.04	-0.06
4	Not Changed	MCE-MIR_5180:fwd	-0.03	0.06	0.03	0.00	-0.01	-0.02	-0.03
4	Not Changed	MCE-MIR_5172:fwd	-1.73	-1.13	-0.40	0.33	0.65	0.98	1.30
4	Not Changed	MCE-MIR_5167:rev	-0.05	-0.04	0.02	0.08	0.04	0.00	-0.05
4	Not Changed	MCE-MIR_5167:fwd	-0.03	0.09	0.04	-0.01	-0.02	-0.03	-0.04
4	Not Changed	MCE-MIR_5152:fwd	0.18	-0.01	-0.02	-0.04	-0.04	-0.04	-0.04
4	Not Changed	MCE-MIR_5143:rev	0.13	-0.03	-0.03	-0.04	-0.03	-0.01	0.01
4	Not Changed	MCE-MIR_5141:rev	-0.14	0.24	0.40	0.55	0.10	-0.35	-0.80
4	Not Changed	MCE-MIR_5135:fwd	0.21	0.01	0.06	0.11	-0.01	-0.13	-0.25
4	Not Changed	MCE-MIR_5122:rev	0.08	-0.02	-0.01	0.00	-0.01	-0.02	-0.03
4	Not Changed	MCE-MIR_5109:rev	-0.49	-0.17	-0.06	0.05	0.14	0.23	0.32
4	Not Changed	MCE-MIR_5105:rev	0.08	-0.18	-0.06	0.07	0.05	0.03	0.01
4	Not Changed	MCE-MIR_5100:rev	-0.17	-0.46	-0.08	0.31	0.22	0.13	0.05
4	Not Changed	MCE-MIR_5089:fwd	0.00	0.05	0.24	0.42	0.09	-0.24	-0.56
4	Not Changed	MCE-MIR_5088:rev	0.23	-0.56	-0.09	0.38	0.20	0.01	-0.17
4	Not Changed	MCE-MIR_5083:rev	0.02	-0.23	0.13	0.48	0.18	-0.13	-0.44
4	Not Changed	MCE-MIR_5079:rev	-0.81	-0.44	-0.14	0.16	0.29	0.41	0.53
4	Not Changed	MCE-MIR_5068:rev	-1.36	-1.52	-0.59	0.34	0.69	1.04	1.40
4	Not Changed	MCE-MIR_5061:rev	-0.17	-0.08	0.07	0.22	0.10	-0.01	-0.13
4	Not Changed	MCE-MIR_5057:fwd	0.07	0.35	0.48	0.61	0.06	-0.50	-1.06
4	Not Changed	MCE-MIR_5056:rev	0.06	-0.29	0.06	0.42	0.17	-0.08	-0.34
4	Not Changed	MCE-MIR_5055:rev	-0.82	-0.28	0.22	0.71	0.38	0.06	-0.27
4	Not Changed	MCE-MIR_5055:fwd	-0.46	-0.59	0.02	0.64	0.38	0.13	-0.12
4	Not Changed	MCE-MIR_5046:rev	0.08	0.02	-0.02	-0.07	-0.04	0.00	0.03

Class	ES Time Pattern	ProbeID	ES-Day0	ES-Day1	ES-Day2	ES-Day3	ES-Day4	ES-Day5	ES-Day6
4	Not Changed	MCE-MIR_504:fwd	0.04	0.01	0.00	-0.02	-0.01	-0.01	0.00
4	Not Changed	MCE-MIR_5033:rev	0.27	0.18	0.04	-0.10	-0.12	-0.13	-0.15
4	Not Changed	MCE-MIR_5030:rev	0.18	-0.02	0.35	0.72	0.16	-0.41	-0.98
4	Not Changed	MCE-MIR_5014:fwd	1.12	-0.21	-0.22	-0.22	-0.19	-0.16	-0.12
4	Not Changed	MCE-MIR_5008:rev	0.27	-0.05	-0.05	-0.04	-0.04	-0.04	-0.05
4	Not Changed	MCE-MIR_5004:rev	-0.53	-0.31	0.03	0.37	0.26	0.15	0.04
4	Not Changed	MCE-MIR_4999:rev	-0.28	-0.60	-0.23	0.14	0.23	0.32	0.41
4	Not Changed	MCE-MIR_4978:rev	0.06	0.06	0.01	-0.03	-0.03	-0.03	-0.03
4	Not Changed	MCE-MIR_4972:fwd	-0.03	0.03	0.01	0.00	0.00	-0.01	-0.01
4	Not Changed	MCE-MIR_4945:fwd	-1.24	-0.27	0.01	0.30	0.35	0.40	0.45
4	Not Changed	MCE-MIR_4922:rev	0.67	0.65	0.39	0.14	-0.24	-0.61	-0.99
4	Not Changed	MCE-MIR_4913:rev	0.64	0.40	0.48	0.56	-0.07	-0.69	-1.32
4	Not Changed	MCE-MIR_4893:rev	0.04	0.00	0.00	0.01	0.00	-0.01	-0.03
4	Not Changed	MCE-MIR_4893:fwd	0.01	-0.06	-0.04	-0.02	0.01	0.04	0.06
4	Not Changed	MCE-MIR_4854:rev	0.52	0.54	0.49	0.44	-0.11	-0.66	-1.21
4	Not Changed	MCE-MIR_4853:fwd	-0.09	-0.07	0.04	0.15	0.07	-0.01	-0.09
4	Not Changed	MCE-MIR_4832:rev	-0.09	-0.04	0.05	0.13	0.06	-0.02	-0.09
4	Not Changed	MCE-MIR_4830:fwd	0.00	0.01	0.05	0.08	0.02	-0.04	-0.11
4	Not Changed	MCE-MIR_4822:rev	0.06	-0.70	0.03	0.76	0.35	-0.05	-0.45
4	Not Changed	MCE-MIR_4821:rev	-0.48	0.23	0.22	0.21	0.07	-0.06	-0.20
4	Not Changed	MCE-MIR_4820:rev	-0.51	0.17	0.21	0.26	0.11	-0.04	-0.19
4	Not Changed	MCE-MIR_482:rev	0.04	0.03	0.00	-0.04	-0.02	-0.01	0.00
4	Not Changed	MCE-MIR_482:fwd	0.11	0.02	-0.01	-0.05	-0.04	-0.03	-0.01
4	Not Changed	MCE-MIR_4809:fwd	0.05	0.09	0.04	-0.02	-0.04	-0.05	-0.07
4	Not Changed	MCE-MIR_4799:rev	-0.51	-0.57	-0.45	-0.34	0.14	0.62	1.11
4	Not Changed	MCE-MIR_4789:rev	0.01	0.03	0.00	-0.04	-0.02	0.00	0.02
4	Not Changed	MCE-MIR_4763:fwd	0.50	-0.16	-0.19	-0.23	-0.10	0.02	0.15
4	Not Changed	MCE-MIR_4762:fwd	0.28	0.01	-0.04	-0.10	-0.07	-0.05	-0.03
4	Not Changed	MCE-MIR_4756:rev	0.25	-0.22	-0.21	-0.19	-0.04	0.12	0.28
4	Not Changed	MCE-MIR_4755:fwd	0.11	0.49	0.48	0.47	-0.02	-0.52	-1.01
4	Not Changed	MCE-MIR_4752:rev	0.25	-0.23	-0.20	-0.17	-0.03	0.12	0.26
4	Not Changed	MCE-MIR_4748:rev	0.15	-0.10	-0.13	-0.16	-0.04	0.08	0.20
4	Not Changed	MCE-MIR_4745:fwd	0.14	0.06	0.01	-0.04	-0.05	-0.06	-0.06
4	Not Changed	MCE-MIR_4743:fwd	0.28	0.31	0.26	0.20	-0.08	-0.35	-0.62
4	Not Changed	MCE-MIR_4738:fwd	0.15	-0.19	-0.07	0.05	0.04	0.02	0.01
4	Not Changed	MCE-MIR_4726:rev	0.24	-0.18	-0.11	-0.04	-0.01	0.03	0.07
4	Not Changed	MCE-MIR_4726:fwd	0.85	-0.63	0.05	0.74	0.20	-0.34	-0.88
4	Not Changed	MCE-MIR_4714:rev	-0.38	0.18	0.77	1.36	0.36	-0.65	-1.65

Class	ES Time Pattern	ProbeID	ES-Day0	ES-Day1	ES-Day2	ES-Day3	ES-Day4	ES-Day5	ES-Day6
4	Not Changed	MCE-MIR_4714:fwd	0.13	-0.13	-0.16	-0.18	-0.03	0.11	0.26
4	Not Changed	MCE-MIR_4712:rev	0.17	-0.03	-0.02	-0.02	-0.03	-0.03	-0.04
4	Not Changed	MCE-MIR_4712:fwd	0.30	-0.29	-0.24	-0.20	-0.03	0.14	0.32
4	Not Changed	MCE-MIR_4711:fwd	0.00	0.00	-0.04	-0.07	-0.02	0.04	0.09
4	Not Changed	MCE-MIR_469:fwd	0.09	0.09	0.05	0.02	-0.03	-0.08	-0.13
4	Not Changed	MCE-MIR_4684:fwd	-0.69	-0.15	0.15	0.46	0.27	0.08	-0.11
4	Not Changed	MCE-MIR_4675:fwd	-0.01	0.11	0.12	0.12	0.00	-0.12	-0.24
4	Not Changed	MCE-MIR_4667:fwd	0.24	0.02	-0.03	-0.08	-0.07	-0.06	-0.04
4	Not Changed	MCE-MIR_4661:rev	0.32	0.96	0.58	0.20	-0.24	-0.69	-1.13
4	Not Changed	MCE-MIR_4661:fwd	0.24	0.57	0.35	0.13	-0.15	-0.43	-0.72
4	Not Changed	MCE-MIR_466:fwd	0.08	0.14	0.04	-0.05	-0.06	-0.07	-0.08
4	Not Changed	MCE-MIR_463:fwd	0.21	-0.20	-0.14	-0.09	-0.01	0.08	0.16
4	Not Changed	MCE-MIR_4625:fwd	-0.26	0.09	0.12	0.16	0.06	-0.04	-0.13
4	Not Changed	MCE-MIR_4614:rev	0.07	0.07	0.01	-0.05	-0.04	-0.03	-0.02
4	Not Changed	MCE-MIR_4607:fwd	-0.04	0.02	0.00	-0.01	0.00	0.01	0.03
4	Not Changed	MCE-MIR_4592:fwd	-0.01	-0.03	0.00	0.04	0.02	0.00	-0.02
4	Not Changed	MCE-MIR_4554:rev	0.34	-0.13	-0.07	-0.01	-0.03	-0.04	-0.05
4	Not Changed	MCE-MIR_4521:rev	-0.05	0.07	0.00	-0.06	-0.03	0.01	0.05
4	Not Changed	MCE-MIR_4521:fwd	0.03	0.01	-0.01	-0.02	-0.01	0.00	0.01
4	Not Changed	MCE-MIR_4513:fwd	-0.29	-0.15	-0.05	0.04	0.10	0.15	0.21
4	Not Changed	MCE-MIR_451:rev	0.68	0.61	0.36	0.10	-0.24	-0.59	-0.93
4	Not Changed	MCE-MIR_4503:rev	0.00	0.01	-0.01	-0.03	-0.01	0.01	0.03
4	Not Changed	MCE-MIR_4491:fwd	0.06	0.03	-0.01	-0.05	-0.03	-0.01	0.01
4	Not Changed	MCE-MIR_4474:fwd	-0.01	0.11	0.02	-0.07	-0.04	-0.02	0.01
4	Not Changed	MCE-MIR_4472:fwd	-0.12	0.22	0.10	-0.03	-0.04	-0.06	-0.07
4	Not Changed	MCE-MIR_4462:fwd	-0.87	-0.21	-0.01	0.18	0.24	0.30	0.36
4	Not Changed	MCE-MIR_4453:fwd	-0.31	0.07	0.13	0.19	0.08	-0.03	-0.14
4	Not Changed	MCE-MIR_4449:rev	-0.04	0.10	0.03	-0.05	-0.03	-0.01	0.01
4	Not Changed	MCE-MIR_4442:fwd	0.03	0.00	0.02	0.05	0.01	-0.03	-0.07
4	Not Changed	MCE-MIR_4413:fwd	-0.04	0.01	0.00	-0.01	0.00	0.02	0.03
4	Not Changed	MCE-MIR_4383:rev	0.02	0.03	0.06	0.10	0.01	-0.07	-0.15
4	Not Changed	MCE-MIR_4353:rev	-0.06	0.02	0.04	0.05	0.02	-0.02	-0.05
4	Not Changed	MCE-MIR_4345:rev	0.20	0.18	0.20	0.22	-0.02	-0.26	-0.51
4	Not Changed	MCE-MIR_4342:fwd	0.00	0.03	0.00	-0.02	-0.01	-0.01	0.00
4	Not Changed	MCE-MIR_4320:rev	-0.39	0.01	0.49	0.98	0.31	-0.36	-1.03
4	Not Changed	MCE-MIR_4303:fwd	-0.04	0.08	0.02	-0.04	-0.02	-0.01	0.01
4	Not Changed	MCE-MIR_4280:rev	0.09	0.01	-0.01	-0.02	-0.02	-0.03	-0.03
4	Not Changed	MCE-MIR_4280:fwd	-0.06	-0.01	0.02	0.05	0.02	0.00	-0.02

Class	ES Time Pattern	ProbeID	ES-Day0	ES-Day1	ES-Day2	ES-Day3	ES-Day4	ES-Day5	ES-Day6
4	Not Changed	MCE-MIR_4274:fwd	-0.22	-0.14	-0.02	0.09	0.09	0.10	0.10
4	Not Changed	MCE-MIR_4273:fwd	-1.06	-0.82	0.07	0.97	0.62	0.28	-0.06
4	Not Changed	MCE-MIR_4239:rev	-0.10	-0.05	0.07	0.18	0.07	-0.04	-0.15
4	Not Changed	MCE-MIR_4236:rev	-0.08	-0.05	0.06	0.17	0.07	-0.03	-0.13
4	Not Changed	MCE-MIR_4232:fwd	-0.05	0.04	0.03	0.02	0.00	-0.01	-0.02
4	Not Changed	MCE-MIR_4226:fwd	-0.08	-0.23	0.09	0.41	0.17	-0.07	-0.30
4	Not Changed	MCE-MIR_4209:fwd	-0.03	0.03	0.02	0.00	0.00	-0.01	-0.01
4	Not Changed	MCE-MIR_4207:fwd	-0.01	0.08	0.02	-0.04	-0.03	-0.01	0.00
4	Not Changed	MCE-MIR_4202:rev	0.03	-0.05	0.04	0.14	0.04	-0.05	-0.15
4	Not Changed	MCE-MIR_4182:rev	-0.05	0.05	0.04	0.03	0.00	-0.02	-0.04
4	Not Changed	MCE-MIR_4179:fwd	-0.02	0.00	0.00	0.00	0.00	0.00	0.00
4	Not Changed	MCE-MIR_4124:fwd	0.02	0.07	0.01	-0.05	-0.03	-0.01	0.01
4	Not Changed	MCE-MIR_4087:rev	-0.08	-0.05	0.12	0.29	0.10	-0.09	-0.29
4	Not Changed	MCE-MIR_4087:fwd	0.52	0.13	0.06	-0.01	-0.12	-0.23	-0.34
4	Not Changed	MCE-MIR_407:fwd	0.04	-0.03	-0.04	-0.05	-0.01	0.02	0.06
4	Not Changed	MCE-MIR_4069:rev	-0.03	-0.01	0.02	0.05	0.02	-0.01	-0.05
4	Not Changed	MCE-MIR_4063:rev	-0.03	0.03	0.01	-0.01	-0.01	0.00	0.00
4	Not Changed	MCE-MIR_4063:fwd	-0.02	0.00	-0.02	-0.04	-0.01	0.02	0.05
4	Not Changed	MCE-MIR_4061:rev	-0.02	-0.02	0.00	0.02	0.01	0.01	0.01
4	Not Changed	MCE-MIR_4060:rev	-0.07	0.02	0.02	0.02	0.01	0.00	-0.01
4	Not Changed	MCE-MIR_406:rev	0.29	0.53	0.39	0.26	-0.12	-0.49	-0.87
4	Not Changed	MCE-MIR_405:rev	0.02	0.07	0.02	-0.03	-0.03	-0.03	-0.02
4	Not Changed	MCE-MIR_4034:rev	-0.15	-0.08	-0.01	0.06	0.06	0.06	0.06
4	Not Changed	MCE-MIR_4031:fwd	0.13	0.05	-0.06	-0.17	-0.08	0.02	0.11
4	Not Changed	MCE-MIR_4030:rev	-0.17	0.07	0.49	0.92	0.24	-0.44	-1.12
4	Not Changed	MCE-MIR_4027:rev	-0.50	-0.22	-0.12	-0.02	0.13	0.29	0.44
4	Not Changed	MCE-MIR_4022:fwd	-0.08	-0.10	-0.01	0.08	0.06	0.03	0.01
4	Not Changed	MCE-MIR_4015:fwd	-0.23	0.07	-0.02	-0.12	-0.01	0.10	0.21
4	Not Changed	MCE-MIR_4010:rev	-0.05	-0.25	0.06	0.37	0.16	-0.05	-0.25
4	Not Changed	MCE-MIR_399:fwd	0.07	0.11	0.06	0.01	-0.04	-0.08	-0.12
4	Not Changed	MCE-MIR_3958:fwd	-1.43	-1.09	-0.51	0.06	0.53	0.99	1.45
4	Not Changed	MCE-MIR_3888:rev	-0.07	0.02	0.03	0.03	0.01	0.00	-0.02
4	Not Changed	MCE-MIR_3886:rev	0.09	0.19	0.08	-0.03	-0.07	-0.11	-0.15
4	Not Changed	MCE-MIR_3886:fwd	-0.09	0.06	0.09	0.12	0.03	-0.06	-0.14
4	Not Changed	MCE-MIR_3867:rev	-0.03	-0.01	-0.04	-0.07	-0.01	0.05	0.10
4	Not Changed	MCE-MIR_3859:rev	-0.13	-0.08	-0.01	0.06	0.06	0.05	0.05
4	Not Changed	MCE-MIR_3847:rev	-0.14	0.12	0.08	0.05	0.01	-0.04	-0.08
4	Not Changed	MCE-MIR_3841:fwd	0.02	-0.07	0.01	0.09	0.04	-0.02	-0.07

Class	ES Time Pattern	ProbeID	ES-Day0	ES-Day1	ES-Day2	ES-Day3	ES-Day4	ES-Day5	ES-Day6
4	Not Changed	MCE-MIR_3837:rev	-0.06	0.15	0.37	0.59	0.12	-0.35	-0.82
4	Not Changed	MCE-MIR_3832:rev	0.03	0.01	0.00	-0.01	-0.01	-0.01	0.00
4	Not Changed	MCE-MIR_3820:fwd	-0.09	0.00	-0.05	-0.10	-0.01	0.08	0.17
4	Not Changed	MCE-MIR_3797:fwd	0.15	-0.23	-0.02	0.19	0.08	-0.03	-0.14
4	Not Changed	MCE-MIR_3793:rev	-0.33	-0.08	0.07	0.22	0.13	0.04	-0.05
4	Not Changed	MCE-MIR_3791:rev	-0.36	-0.26	0.35	0.96	0.37	-0.23	-0.82
4	Not Changed	MCE-MIR_3782:rev	-0.52	-0.15	-0.02	0.11	0.15	0.19	0.24
4	Not Changed	MCE-MIR_3780:rev	-0.25	-0.40	-0.15	0.11	0.17	0.23	0.29
4	Not Changed	MCE-MIR_3762:rev	-1.17	0.17	0.24	0.32	0.23	0.15	0.06
4	Not Changed	MCE-MIR_3754:rev	0.01	-0.01	0.02	0.05	0.01	-0.02	-0.06
4	Not Changed	MCE-MIR_3751:fwd	-0.60	-0.06	-0.13	-0.21	0.06	0.33	0.60
4	Not Changed	MCE-MIR_3715:rev	-0.02	-0.02	0.00	0.03	0.02	0.00	-0.01
4	Not Changed	MCE-MIR_3695:fwd	0.16	0.22	0.07	-0.08	-0.10	-0.13	-0.15
4	Not Changed	MCE-MIR_3686:fwd	-0.62	0.11	0.05	-0.02	0.07	0.16	0.25
4	Not Changed	MCE-MIR_3685:rev	0.07	-0.32	0.07	0.46	0.18	-0.09	-0.37
4	Not Changed	MCE-MIR_3684:rev	0.09	-0.04	-0.01	0.02	0.00	-0.02	-0.04
4	Not Changed	MCE-MIR_3667:rev	0.04	-0.02	-0.01	0.00	0.00	0.00	-0.01
4	Not Changed	MCE-MIR_3667:fwd	-0.01	0.02	0.03	0.03	0.00	-0.02	-0.05
4	Not Changed	MCE-MIR_3663:fwd	-0.04	-0.11	-0.05	0.01	0.04	0.07	0.10
4	Not Changed	MCE-MIR_3653:rev	0.09	0.33	0.30	0.26	-0.03	-0.32	-0.62
4	Not Changed	MCE-MIR_3651:fwd	0.06	0.00	0.00	0.00	-0.01	-0.02	-0.03
4	Not Changed	MCE-MIR_3646:rev	-0.13	0.12	0.10	0.08	0.01	-0.06	-0.13
4	Not Changed	MCE-MIR_3642:rev	0.17	0.02	0.11	0.20	0.01	-0.17	-0.35
4	Not Changed	MCE-MIR_364:fwd	-0.25	-0.32	0.01	0.34	0.20	0.07	-0.06
4	Not Changed	MCE-MIR_3637:rev	0.04	0.06	0.00	-0.06	-0.04	-0.01	0.01
4	Not Changed	MCE-MIR_3628:rev	-0.96	-0.86	-0.02	0.83	0.58	0.34	0.09
4	Not Changed	MCE-MIR_3626:rev	-0.17	-0.42	-0.25	-0.07	0.12	0.30	0.49
4	Not Changed	MCE-MIR_3624:rev	-0.63	-0.43	-0.19	0.05	0.23	0.40	0.58
4	Not Changed	MCE-MIR_3624:fwd	-0.01	0.13	-0.02	-0.17	-0.07	0.03	0.12
4	Not Changed	MCE-MIR_3619:rev	0.06	-0.12	-0.04	0.03	0.03	0.03	0.02
4	Not Changed	MCE-MIR_3613:rev	-0.29	-0.29	-0.07	0.16	0.16	0.17	0.17
4	Not Changed	MCE-MIR_3609:rev	-0.28	0.09	0.22	0.35	0.11	-0.13	-0.37
4	Not Changed	MCE-MIR_3595:fwd	-0.27	-0.19	-0.15	-0.11	0.07	0.24	0.42
4	Not Changed	MCE-MIR_3573:rev	-1.19	-0.64	-0.25	0.15	0.39	0.64	0.89
4	Not Changed	MCE-MIR_3572:rev	-0.73	-0.67	-0.31	0.05	0.30	0.55	0.80
4	Not Changed	MCE-MIR_3571:rev	-0.27	-0.08	0.00	0.08	0.09	0.09	0.09
4	Not Changed	MCE-MIR_3557:rev	-0.56	-0.41	0.07	0.55	0.33	0.11	-0.10
4	Not Changed	MCE-MIR_3550:fwd	0.07	-0.10	-0.05	0.00	0.01	0.03	0.04

Class	ES Time Pattern	ProbeID	ES-Day0	ES-Day1	ES-Day2	ES-Day3	ES-Day4	ES-Day5	ES-Day6
4	Not Changed	MCE-MIR_3543:rev	0.15	-0.02	0.15	0.33	0.06	-0.21	-0.47
4	Not Changed	MCE-MIR_3541:rev	-0.75	-0.24	0.09	0.42	0.29	0.16	0.02
4	Not Changed	MCE-MIR_3531:rev	-1.30	-0.20	0.12	0.44	0.38	0.31	0.24
4	Not Changed	MCE-MIR_3529:rev	0.29	-0.94	-0.07	0.79	0.39	-0.02	-0.43
4	Not Changed	MCE-MIR_3523:rev	-0.81	-0.46	-0.12	0.21	0.30	0.39	0.49
4	Not Changed	MCE-MIR_3522:rev	-0.04	-0.15	-0.08	-0.01	0.04	0.09	0.15
4	Not Changed	MCE-MIR_3518:rev	-1.12	-0.85	-0.27	0.32	0.48	0.64	0.80
4	Not Changed	MCE-MIR_3513:fwd	-0.07	-0.05	0.02	0.09	0.05	0.01	-0.03
4	Not Changed	MCE-MIR_3502:rev	-0.22	-0.25	-0.09	0.07	0.12	0.17	0.22
4	Not Changed	MCE-MIR_3495:rev	-0.25	-0.48	-0.29	-0.11	0.13	0.38	0.62
4	Not Changed	MCE-MIR_3491:rev	-0.60	-0.02	0.09	0.20	0.15	0.11	0.07
4	Not Changed	MCE-MIR_3490:rev	-1.05	-0.08	0.14	0.37	0.29	0.21	0.13
4	Not Changed	MCE-MIR_3488:rev	-0.73	-0.29	0.10	0.48	0.31	0.15	-0.02
4	Not Changed	MCE-MIR_3485:rev	-0.31	-0.07	0.08	0.24	0.13	0.02	-0.09
4	Not Changed	MCE-MIR_3484:rev	-0.92	-0.13	0.05	0.24	0.25	0.25	0.26
4	Not Changed	MCE-MIR_3471:rev	-0.33	-0.37	-0.21	-0.06	0.13	0.32	0.51
4	Not Changed	MCE-MIR_3444:rev	0.04	0.01	0.01	0.02	0.00	-0.02	-0.05
4	Not Changed	MCE-MIR_3441:fwd	-0.05	-0.08	0.17	0.43	0.14	-0.16	-0.46
4	Not Changed	MCE-MIR_3439:rev	-0.01	0.19	0.28	0.37	0.05	-0.28	-0.61
4	Not Changed	MCE-MIR_3429:rev	-1.22	-0.61	0.03	0.66	0.52	0.38	0.24
4	Not Changed	MCE-MIR_3408:fwd	-0.49	-0.22	-0.02	0.19	0.18	0.18	0.17
4	Not Changed	MCE-MIR_3407:rev	-0.13	-0.10	0.05	0.20	0.10	0.00	-0.11
4	Not Changed	MCE-MIR_335:fwd	-0.65	-0.13	0.05	0.22	0.19	0.17	0.14
4	Not Changed	MCE-MIR_3334:fwd	-0.33	0.12	0.28	0.43	0.13	-0.16	-0.46
4	Not Changed	MCE-MIR_3333:rev	-0.01	0.08	0.02	-0.03	-0.03	-0.02	-0.02
4	Not Changed	MCE-MIR_3330:fwd	0.03	-0.02	-0.02	-0.02	0.00	0.01	0.03
4	Not Changed	MCE-MIR_3295:rev	-0.55	-0.10	0.10	0.30	0.19	0.08	-0.02
4	Not Changed	MCE-MIR_3261:fwd	-0.17	-0.22	-0.14	-0.06	0.07	0.20	0.32
4	Not Changed	MCE-MIR_3260:rev	-0.81	-0.43	-0.07	0.29	0.31	0.34	0.37
4	Not Changed	MCE-MIR_321:rev	-0.15	-0.32	0.18	0.68	0.28	-0.13	-0.54
4	Not Changed	MCE-MIR_3155:fwd	-0.14	0.21	0.24	0.28	0.04	-0.20	-0.44
4	Not Changed	MCE-MIR_3147:fwd	-0.56	-0.25	-0.07	0.12	0.19	0.25	0.32
4	Not Changed	MCE-MIR_3134:rev	0.63	-0.30	0.35	0.99	0.22	-0.56	-1.33
4	Not Changed	MCE-MIR_3113:fwd	-0.01	0.18	0.07	-0.04	-0.05	-0.06	-0.07
4	Not Changed	MCE-MIR_3101:rev	0.17	-0.13	-0.01	0.10	0.03	-0.04	-0.12
4	Not Changed	MCE-MIR_3084:fwd	-0.04	0.07	0.05	0.04	0.00	-0.04	-0.08
4	Not Changed	MCE-MIR_3059:rev	-0.05	-0.01	0.01	0.04	0.02	0.00	-0.01
4	Not Changed	MCE-MIR_3057:rev	-0.06	0.04	0.01	-0.01	0.00	0.01	0.02

Class	ES Time Pattern	ProbeID	ES-Day0	ES-Day1	ES-Day2	ES-Day3	ES-Day4	ES-Day5	ES-Day6
4	Not Changed	MCE-MIR_3057:fwd	0.08	-0.01	0.01	0.03	0.00	-0.03	-0.06
4	Not Changed	MCE-MIR_3032:fwd	-0.13	-0.01	0.00	0.01	0.03	0.04	0.06
4	Not Changed	MCE-MIR_3007:rev	-0.06	-0.03	-0.01	0.02	0.02	0.03	0.03
4	Not Changed	MCE-MIR_2983:rev	0.16	0.08	-0.02	-0.12	-0.08	-0.03	0.01
4	Not Changed	MCE-MIR_2977:rev	-0.31	-0.62	0.11	0.84	0.42	-0.01	-0.43
4	Not Changed	MCE-MIR_2968:fwd	-0.04	0.06	0.06	0.07	0.01	-0.05	-0.11
4	Not Changed	MCE-MIR_2953:fwd	0.01	-0.46	-0.15	0.16	0.15	0.15	0.14
4	Not Changed	MCE-MIR_293:rev	-0.12	-0.06	0.00	0.05	0.05	0.04	0.04
4	Not Changed	MCE-MIR_291:rev	0.06	-0.07	-0.01	0.04	0.02	-0.01	-0.03
4	Not Changed	MCE-MIR_2894:fwd	-0.08	-0.03	-0.01	0.00	0.02	0.04	0.06
4	Not Changed	MCE-MIR_2889:fwd	0.01	0.07	0.06	0.06	-0.01	-0.07	-0.13
4	Not Changed	MCE-MIR_2866:rev	-0.48	-0.88	-0.18	0.52	0.43	0.34	0.25
4	Not Changed	MCE-MIR_2817:fwd	-1.22	-0.70	-0.12	0.46	0.49	0.53	0.56
4	Not Changed	MCE-MIR_281:rev	-0.03	0.02	0.01	0.01	0.00	0.00	-0.01
4	Not Changed	MCE-MIR_2798:fwd	-0.08	0.04	0.02	-0.01	0.00	0.01	0.02
4	Not Changed	MCE-MIR_2745:fwd	-0.43	0.20	0.30	0.39	0.12	-0.16	-0.43
4	Not Changed	MCE-MIR_273:fwd	-1.35	-0.07	0.20	0.47	0.36	0.26	0.15
4	Not Changed	MCE-MIR_2722:rev	-0.16	0.09	0.03	-0.04	0.00	0.03	0.06
4	Not Changed	MCE-MIR_2711:rev	-0.11	0.04	0.02	0.01	0.01	0.01	0.01
4	Not Changed	MCE-MIR_2698:rev	-1.27	-0.63	-0.24	0.15	0.41	0.66	0.91
4	Not Changed	MCE-MIR_2691:rev	-0.03	-0.02	-0.01	-0.01	0.01	0.02	0.04
4	Not Changed	MCE-MIR_2680:rev	1.82	-0.27	-0.18	-0.09	-0.26	-0.43	-0.60
4	Not Changed	MCE-MIR_2680:fwd	-0.45	-0.28	0.32	0.91	0.37	-0.17	-0.71
4	Not Changed	MCE-MIR_2679:fwd	-1.42	-1.53	-0.60	0.34	0.70	1.07	1.44
4	Not Changed	MCE-MIR_2661:rev	-0.06	-0.07	-0.04	-0.01	0.02	0.06	0.10
4	Not Changed	MCE-MIR_2624:rev	0.01	-0.01	0.00	0.00	0.00	0.00	0.00
4	Not Changed	MCE-MIR_2617:fwd	0.39	-0.04	-0.06	-0.09	-0.08	-0.07	-0.06
4	Not Changed	MCE-MIR_2566:rev	-0.02	0.00	0.00	0.01	0.01	0.00	0.00
4	Not Changed	MCE-MIR_2566:fwd	-0.15	0.19	0.11	0.04	-0.01	-0.06	-0.11
4	Not Changed	MCE-MIR_2563:rev	0.17	0.10	-0.08	-0.26	-0.12	0.02	0.17
4	Not Changed	MCE-MIR_254:fwd	-0.06	-0.01	0.06	0.14	0.05	-0.04	-0.14
4	Not Changed	MCE-MIR_2522:rev	-0.08	0.01	0.00	0.00	0.01	0.02	0.04
4	Not Changed	MCE-MIR_2501:fwd	-0.15	-0.15	-0.07	0.01	0.06	0.12	0.18
4	Not Changed	MCE-MIR_2474:rev	0.18	0.03	0.01	-0.01	-0.04	-0.07	-0.10
4	Not Changed	MCE-MIR_2470:fwd	-0.36	-0.01	0.08	0.17	0.11	0.04	-0.03
4	Not Changed	MCE-MIR_2464:fwd	-0.02	-0.07	0.03	0.13	0.05	-0.02	-0.10
4	Not Changed	MCE-MIR_2419:fwd	-0.05	0.02	0.01	0.00	0.00	0.01	0.01
4	Not Changed	MCE-MIR_2417:fwd	-0.02	0.00	0.00	0.00	0.00	0.01	0.01

Class	ES Time Pattern	ProbeID	ES-Day0	ES-Day1	ES-Day2	ES-Day3	ES-Day4	ES-Day5	ES-Day6
4	Not Changed	MCE-MIR_2388:rev	-0.65	-0.19	0.03	0.26	0.22	0.19	0.15
4	Not Changed	MCE-MIR_2371:rev	0.03	0.03	0.00	-0.03	-0.02	-0.01	0.01
4	Not Changed	MCE-MIR_2371:fwd	0.02	-0.01	-0.01	-0.01	0.00	0.01	0.02
4	Not Changed	MCE-MIR_2364:rev	0.34	0.10	0.01	-0.08	-0.10	-0.12	-0.14
4	Not Changed	MCE-MIR_2361:fwd	0.79	0.37	0.32	0.28	-0.16	-0.59	-1.02
4	Not Changed	MCE-MIR_2345:fwd	0.18	0.21	0.52	0.84	0.13	-0.58	-1.29
4	Not Changed	MCE-MIR_2339:rev	-0.07	0.13	0.07	0.01	-0.02	-0.05	-0.07
4	Not Changed	MCE-MIR_2339:fwd	0.17	-0.06	-0.02	0.02	-0.01	-0.03	-0.06
4	Not Changed	MCE-MIR_2327:fwd	-1.25	-0.09	0.13	0.35	0.32	0.29	0.26
4	Not Changed	MCE-MIR_2304:rev	0.11	-0.07	-0.03	0.00	0.00	0.00	0.00
4	Not Changed	MCE-MIR_2288:fwd	0.05	-0.06	-0.03	-0.01	0.01	0.02	0.03
4	Not Changed	MCE-MIR_2243:fwd	-0.57	-0.28	-0.06	0.15	0.20	0.26	0.31
4	Not Changed	MCE-MIR_2222:fwd	-0.14	0.01	0.10	0.18	0.07	-0.05	-0.16
4	Not Changed	MCE-MIR_2205:fwd	0.02	0.00	0.01	0.01	0.00	-0.01	-0.02
4	Not Changed	MCE-MIR_2198:fwd	0.05	-0.02	-0.02	-0.01	0.00	0.00	0.00
4	Not Changed	MCE-MIR_2197:fwd	0.04	-0.01	-0.01	0.00	0.00	-0.01	-0.01
4	Not Changed	MCE-MIR_2196:fwd	-0.05	0.09	0.16	0.23	0.04	-0.15	-0.34
4	Not Changed	MCE-MIR_2192:rev	0.31	0.00	0.02	0.04	-0.04	-0.12	-0.20
4	Not Changed	MCE-MIR_2173:rev	0.20	-0.08	0.00	0.09	0.01	-0.07	-0.15
4	Not Changed	MCE-MIR_2171:rev	-0.59	-0.65	-0.30	0.06	0.28	0.49	0.71
4	Not Changed	MCE-MIR_2166:rev	0.16	-0.01	0.01	0.02	-0.02	-0.06	-0.10
4	Not Changed	MCE-MIR_2166:fwd	0.10	0.03	-0.01	-0.05	-0.04	-0.02	-0.01
4	Not Changed	MCE-MIR_2164:rev	-0.27	-0.13	-0.02	0.10	0.10	0.11	0.11
4	Not Changed	MCE-MIR_2139:fwd	-0.97	-0.44	-0.22	0.01	0.27	0.54	0.81
4	Not Changed	MCE-MIR_2134:fwd	-0.03	-0.24	-0.03	0.18	0.11	0.04	-0.03
4	Not Changed	MCE-MIR_2099:fwd	-0.43	0.50	0.38	0.26	0.01	-0.24	-0.49
4	Not Changed	MCE-MIR_2092:fwd	-0.23	0.10	0.16	0.21	0.06	-0.08	-0.22
4	Not Changed	MCE-MIR_2087:rev	-0.01	0.06	0.02	-0.02	-0.02	-0.02	-0.02
4	Not Changed	MCE-MIR_2078:fwd	-0.10	0.47	0.13	-0.20	-0.15	-0.10	-0.05
4	Not Changed	MCE-MIR_1998:fwd	0.05	0.02	0.00	-0.02	-0.02	-0.02	-0.01
4	Not Changed	MCE-MIR_1974:fwd	0.36	-0.05	0.32	0.69	0.12	-0.44	-1.00
4	Not Changed	MCE-MIR_1973:fwd	0.21	0.21	0.48	0.75	0.10	-0.55	-1.20
4	Not Changed	MCE-MIR_1929:fwd	-0.03	0.03	0.00	-0.04	-0.01	0.02	0.04
4	Not Changed	MCE-MIR_1905:rev	-0.02	0.09	0.03	-0.04	-0.03	-0.02	-0.01
4	Not Changed	MCE-MIR_188:fwd	-0.06	0.07	0.02	-0.04	-0.01	0.01	0.03
4	Not Changed	MCE-MIR_1829:rev	0.35	-0.03	-0.02	0.00	-0.05	-0.10	-0.15
4	Not Changed	MCE-MIR_1811:rev	-0.01	-0.07	-0.05	-0.02	0.01	0.05	0.08
4	Not Changed	MCE-MIR_1793:fwd	-0.14	0.29	0.28	0.27	0.02	-0.23	-0.48

Class	ES Time Pattern	ProbeID	ES-Day0	ES-Day1	ES-Day2	ES-Day3	ES-Day4	ES-Day5	ES-Day6
4	Not Changed	MCE-MIR_1792:rev	0.11	-0.01	0.31	0.62	0.14	-0.34	-0.83
4	Not Changed	MCE-MIR_1786:fwd	0.05	-0.03	0.00	0.03	0.01	-0.01	-0.04
4	Not Changed	MCE-MIR_1778:fwd	-0.85	-0.40	0.04	0.48	0.36	0.24	0.12
4	Not Changed	MCE-MIR_1773:rev	0.23	-0.06	-0.05	-0.04	-0.03	-0.03	-0.02
4	Not Changed	MCE-MIR_1756:rev	0.28	-0.01	-0.02	-0.02	-0.05	-0.08	-0.11
4	Not Changed	MCE-MIR_1742:rev	-0.44	-0.45	0.28	1.00	0.44	-0.13	-0.69
4	Not Changed	MCE-MIR_1710:rev	0.05	0.01	0.00	-0.01	-0.01	-0.02	-0.02
4	Not Changed	MCE-MIR_1697:fwd	0.00	0.13	0.07	0.01	-0.03	-0.07	-0.11
4	Not Changed	MCE-MIR_1689:rev	0.02	0.16	0.07	-0.01	-0.05	-0.08	-0.11
4	Not Changed	MCE-MIR_1679:fwd	0.00	0.04	0.02	-0.01	-0.01	-0.02	-0.02
4	Not Changed	MCE-MIR_1670:fwd	-0.28	-0.23	0.07	0.38	0.20	0.02	-0.16
4	Not Changed	MCE-MIR_1645:rev	-0.01	0.07	0.03	-0.01	-0.02	-0.03	-0.03
4	Not Changed	MCE-MIR_1642:rev	-0.08	-0.02	0.00	0.02	0.02	0.02	0.03
4	Not Changed	MCE-MIR_162:fwd	-0.10	-0.18	-0.03	0.12	0.09	0.06	0.03
4	Not Changed	MCE-MIR_1611:fwd	-0.51	-0.11	0.26	0.63	0.27	-0.09	-0.44
4	Not Changed	MCE-MIR_1597:fwd	-0.44	-0.15	0.04	0.22	0.17	0.11	0.06
4	Not Changed	MCE-MIR_1576:rev	0.01	-0.06	-0.01	0.05	0.02	0.00	-0.02
4	Not Changed	MCE-MIR_1569:rev	-0.38	0.08	0.34	0.60	0.19	-0.21	-0.62
4	Not Changed	MCE-MIR_1546:rev	-0.07	-0.02	0.02	0.05	0.03	0.01	-0.02
4	Not Changed	MCE-MIR_1544:rev	-1.22	-0.19	0.28	0.75	0.44	0.13	-0.18
4	Not Changed	MCE-MIR_1538:fwd	-0.41	0.22	0.15	0.09	0.04	-0.02	-0.07
4	Not Changed	MCE-MIR_1536:rev	-1.07	0.07	0.29	0.50	0.28	0.07	-0.14
4	Not Changed	MCE-MIR_1535:rev	-0.68	-0.29	-0.06	0.18	0.23	0.29	0.34
4	Not Changed	MCE-MIR_151:fwd	0.05	-0.02	-0.03	-0.03	-0.01	0.01	0.03
4	Not Changed	MCE-MIR_1508:fwd	-0.72	-0.25	0.09	0.44	0.29	0.14	0.00
4	Not Changed	MCE-MIR_1495:fwd	-0.12	0.07	0.02	-0.02	0.00	0.02	0.04
4	Not Changed	MCE-MIR_1482:rev	-0.04	0.09	0.05	0.01	-0.01	-0.04	-0.06
4	Not Changed	MCE-MIR_1478:fwd	-0.33	-0.15	0.00	0.14	0.13	0.11	0.10
4	Not Changed	MCE-MIR_1458:rev	-0.48	-0.40	0.03	0.47	0.30	0.13	-0.05
4	Not Changed	MCE-MIR_1442:fwd	-0.06	0.11	0.04	-0.03	-0.02	-0.01	-0.01
4	Not Changed	MCE-MIR_1433:rev	0.02	-0.01	0.00	0.01	0.00	-0.01	-0.01
4	Not Changed	MCE-MIR_1433:fwd	-0.06	-0.09	-0.04	0.00	0.03	0.06	0.09
4	Not Changed	MCE-MIR_1412:fwd	0.09	-0.01	0.00	0.00	-0.01	-0.03	-0.04
4	Not Changed	MCE-MIR_1409:fwd	-0.65	-0.18	0.07	0.32	0.24	0.15	0.06
4	Not Changed	MCE-MIR_1408:fwd	0.64	0.27	0.48	0.69	0.00	-0.69	-1.39
4	Not Changed	MCE-MIR_1401:fwd	0.02	0.00	0.00	0.01	0.00	-0.01	-0.02
4	Not Changed	MCE-MIR_1365:rev	0.03	-0.04	0.01	0.05	0.02	-0.02	-0.05
4	Not Changed	MCE-MIR_1365:fwd	-1.39	-0.56	-0.11	0.34	0.46	0.57	0.69

Class	ES Time Pattern	ProbeID	ES-Day0	ES-Day1	ES-Day2	ES-Day3	ES-Day4	ES-Day5	ES-Day6
4	Not Changed	MCE-MIR_1364:fwd	0.07	0.24	0.32	0.39	0.03	-0.34	-0.71
4	Not Changed	MCE-MIR_1356:rev	-0.04	-0.02	0.02	0.06	0.03	-0.01	-0.04
4	Not Changed	MCE-MIR_1356:fwd	-0.07	-0.14	-0.01	0.12	0.08	0.03	-0.01
4	Not Changed	MCE-MIR_1352:fwd	-0.98	-0.15	0.15	0.46	0.32	0.17	0.03
4	Not Changed	MCE-MIR_1342:fwd	0.19	0.69	0.25	-0.19	-0.25	-0.31	-0.38
4	Not Changed	MCE-MIR_1325:rev	-0.73	-0.01	0.20	0.40	0.22	0.04	-0.13
4	Not Changed	MCE-MIR_1311:rev	-0.07	-0.07	0.03	0.14	0.06	-0.01	-0.08
4	Not Changed	MCE-MIR_1283:rev	0.15	0.20	0.23	0.27	-0.01	-0.29	-0.56
4	Not Changed	MCE-MIR_1283:fwd	0.19	-0.21	-0.15	-0.09	0.00	0.09	0.18
4	Not Changed	MCE-MIR_1269:rev	0.05	0.09	0.02	-0.05	-0.04	-0.04	-0.04
4	Not Changed	MCE-MIR_1264:rev	-0.06	-0.04	0.05	0.15	0.06	-0.04	-0.13
4	Not Changed	MCE-MIR_1259:rev	-0.01	0.07	0.03	-0.02	-0.02	-0.02	-0.02
4	Not Changed	MCE-MIR_1192:rev	0.04	0.00	-0.01	-0.01	-0.01	-0.01	0.00
4	Not Changed	MCE-MIR_1190:fwd	-0.28	0.11	0.12	0.13	0.05	-0.03	-0.10
4	Not Changed	MCE-MIR_1066:rev	0.17	-0.02	0.25	0.52	0.11	-0.31	-0.72
4	Not Changed	MCE-MIR_1059:fwd	-0.08	-0.07	0.02	0.12	0.06	0.00	-0.05
4	Not Changed	MCE-MIR_1052:rev	-0.09	-0.07	-0.02	0.02	0.04	0.05	0.07
4	Not Changed	MCE-MIR_1038:fwd	-0.76	-0.33	-0.15	0.04	0.22	0.40	0.58
4	Not Changed	MCE-MIR_1015:rev	-0.32	0.00	-0.02	-0.05	0.04	0.13	0.22
1	Changed	mmu-mir-485-3p	0.81	0.39	0.15	-0.09	-0.26	-0.42	-0.58
1	Changed	mmu-mir-484	0.97	0.28	0.01	-0.26	-0.30	-0.33	-0.37
1	Changed	mmu-mir-467	1.17	1.40	0.41	-0.59	-0.69	-0.79	-0.90
1	Changed	mmu-mir-466	1.26	1.54	0.57	-0.41	-0.70	-0.99	-1.27
1	Changed	mmu-mir-302d	3.41	2.89	0.27	-2.36	-1.88	-1.41	-0.93
1	Changed	mmu-mir-302b	3.91	3.12	0.48	-2.17	-1.97	-1.78	-1.59
1	Changed	mmu-mir-302	3.45	2.79	0.54	-1.71	-1.70	-1.69	-1.68
1	Changed	mmu-mir-295	2.95	2.73	0.99	-0.75	-1.36	-1.98	-2.59
1	Changed	mmu-mir-293	1.87	1.54	0.50	-0.54	-0.83	-1.12	-1.41
1	Changed	mmu-mir-292-5p	1.92	1.68	0.48	-0.73	-0.92	-1.11	-1.31
1	Changed	mmu-mir-292-3p	1.77	1.46	0.44	-0.58	-0.81	-1.03	-1.25
1	Changed	mmu-mir-291b-5p	2.58	2.22	0.68	-0.85	-1.20	-1.54	-1.89
1	Changed	mmu-mir-291a-5p	2.92	2.49	0.81	-0.86	-1.32	-1.79	-2.25
1	Changed	mmu-mir-291a-3p	2.09	2.50	0.93	-0.65	-1.14	-1.62	-2.11
1	Changed	mmu-mir-290	1.63	1.44	0.39	-0.66	-0.80	-0.94	-1.08
1	Changed	mmu-mir-22	1.08	0.32	0.04	-0.25	-0.32	-0.40	-0.47
1	Changed	mmu-mir-195	1.74	1.10	0.38	-0.33	-0.65	-0.96	-1.28
1	Changed	mmu-mir-182	1.73	1.33	0.36	-0.61	-0.77	-0.94	-1.10
1	Changed	mmu-mir-150	1.83	1.38	0.32	-0.75	-0.84	-0.93	-1.01

Class	ES Time Pattern	ProbeID	ES-Day0	ES-Day1	ES-Day2	ES-Day3	ES-Day4	ES-Day5	ES-Day6
1	Not Changed	mmu-mir-96	0.86	0.97	0.28	-0.42	-0.49	-0.56	-0.63
1	Not Changed	mmu-mir-92	1.17	0.82	0.21	-0.40	-0.50	-0.60	-0.70
1	Not Changed	mmu-mir-7	1.98	1.47	0.51	-0.46	-0.81	-1.17	-1.52
1	Not Changed	mmu-mir-363	1.73	1.63	0.64	-0.36	-0.79	-1.21	-1.64
1	Not Changed	mmu-mir-34a	0.81	0.54	0.06	-0.42	-0.38	-0.33	-0.28
1	Not Changed	mmu-mir-31	0.77	0.71	0.13	-0.44	-0.42	-0.39	-0.36
1	Not Changed	S-mmu-mir-302c	0.92	0.62	0.09	-0.45	-0.42	-0.39	-0.37
1	Not Changed	mmu-mir-302c	2.05	1.32	0.21	-0.90	-0.90	-0.89	-0.89
1	Not Changed	mmu-mir-300	0.13	0.50	0.16	-0.19	-0.19	-0.20	-0.21
1	Not Changed	mmu-mir-299	0.69	0.42	0.10	-0.21	-0.27	-0.33	-0.39
1	Not Changed	mmu-mir-297	1.01	1.70	0.56	-0.57	-0.74	-0.90	-1.06
1	Not Changed	mmu-mir-294	1.82	1.74	0.78	-0.18	-0.78	-1.39	-1.99
1	Not Changed	mmu-mir-20b	0.81	0.67	0.10	-0.47	-0.42	-0.37	-0.32
1	Not Changed	mmu-mir-205	1.63	0.71	0.05	-0.61	-0.60	-0.59	-0.58
1	Not Changed	mmu-mir-200b	0.93	0.50	0.04	-0.43	-0.39	-0.35	-0.31
1	Not Changed	mmu-mir-200a	0.55	1.14	0.37	-0.39	-0.47	-0.56	-0.64
1	Not Changed	mmu-mir-183	1.46	0.98	0.21	-0.56	-0.63	-0.70	-0.76
1	Not Changed	mmu-mir-18	1.14	0.59	0.04	-0.51	-0.46	-0.42	-0.38
1	Not Changed	mmu-mir-148b	0.30	0.68	0.17	-0.34	-0.31	-0.27	-0.23
1	Not Changed	mmu-mir-148a	0.96	0.89	0.14	-0.60	-0.53	-0.47	-0.40
1	Not Changed	mmu-mir-101b	1.25	0.69	0.05	-0.59	-0.53	-0.47	-0.41
1	Not Changed	mmu-mir-101a	0.95	0.71	0.16	-0.39	-0.43	-0.48	-0.52
3	Changed	mmu-mir-99a	-2.75	-2.60	-1.09	0.42	1.21	2.00	2.79
3	Changed	S-mmu-mir-9	-1.99	-1.70	-1.15	-0.61	0.61	1.82	3.03
3	Changed	mmu-mir-9	-2.50	-2.27	-1.18	-0.10	0.96	2.02	3.08
3	Changed	mmu-mir-542-3p	-0.20	-0.26	-0.19	-0.12	0.07	0.26	0.45
3	Changed	mmu-mir-503	-0.80	-0.90	-0.53	-0.15	0.32	0.79	1.26
3	Changed	mmu-mir-450	-0.31	-0.14	-0.30	-0.47	-0.03	0.41	0.84
3	Changed	mmu-mir-424	-1.73	-1.63	-0.80	0.02	0.70	1.38	2.06
3	Changed	mmu-mir-383	-0.02	-0.17	-0.13	-0.10	0.02	0.14	0.26
3	Changed	mmu-mir-376a	0.19	-0.61	-0.55	-0.49	0.00	0.49	0.98
3	Changed	mmu-mir-351	-1.61	-1.46	-0.77	-0.09	0.61	1.31	2.01
3	Changed	mmu-mir-344	-0.35	-0.13	-0.34	-0.55	-0.05	0.45	0.95
3	Changed	mmu-mir-335	-1.82	-1.88	-0.55	0.79	0.97	1.15	1.34
3	Changed	mmu-mir-322	-0.87	-0.86	-0.52	-0.17	0.32	0.81	1.29
3	Changed	mmu-mir-218	-1.20	-1.06	-0.83	-0.61	0.31	1.23	2.15
3	Changed	mmu-mir-214	-2.21	-2.15	-1.02	0.12	0.94	1.76	2.58
3	Changed	S-mmu-mir-199a	-2.22	-2.05	-1.28	-0.51	0.76	2.02	3.28

Class	ES Time Pattern	ProbeID	ES-Day0	ES-Day1	ES-Day2	ES-Day3	ES-Day4	ES-Day5	ES-Day6
3	Changed	mmu-mir-181a	-1.68	-1.81	-1.01	-0.20	0.68	1.57	2.46
3	Changed	mmu-mir-134	-0.12	-0.79	-0.41	-0.04	0.21	0.46	0.70
3	Changed	mmu-mir-132	-0.32	-0.10	-0.20	-0.30	0.00	0.31	0.61
3	Changed	mmu-mir-125b	-2.03	-2.55	-1.27	0.01	0.98	1.95	2.92
3	Changed	mmu-mir-10b	-3.50	-3.38	-1.37	0.63	1.59	2.54	3.49
3	Changed	mmu-mir-10a	-5.07	-2.88	-0.74	1.41	1.92	2.43	2.94
3	Changed	mmu-mir-100	-2.48	-2.27	-1.00	0.26	1.05	1.83	2.61
3	Not Changed	mmu-mir-99b	-0.57	-0.68	-0.41	-0.13	0.23	0.60	0.96
3	Not Changed	mmu-mir-216	-0.19	-0.18	-0.04	0.10	0.10	0.10	0.10
3	Not Changed	mmu-mir-199b	-0.21	-0.25	-0.24	-0.23	0.04	0.31	0.58
3	Not Changed	mmu-mir-199a	-0.63	-0.68	-0.53	-0.37	0.18	0.74	1.29
3	Not Changed	mmu-mir-188	-0.46	-0.46	-0.26	-0.06	0.18	0.41	0.65
3	Not Changed	mmu-mir-181b	-0.95	-1.19	-0.72	-0.25	0.39	1.04	1.68
3	Not Changed	mmu-mir-125a	-0.50	-0.68	-0.59	-0.49	0.13	0.76	1.38
3	Not Changed	mmu-let-7i	-0.66	-0.79	-0.84	-0.88	0.09	1.06	2.02
3	Not Changed	mmu-let-7f	-1.19	-0.85	-0.97	-1.10	0.14	1.37	2.61
3	Not Changed	mmu-let-7e	-1.17	-1.04	-0.88	-0.72	0.27	1.27	2.26
3	Not Changed	mmu-let-7d	-0.97	-0.99	-1.10	-1.22	0.10	1.42	2.74
3	Not Changed	mmu-let-7c	-0.79	-1.16	-1.26	-1.37	0.08	1.53	2.97
3	Not Changed	mmu-let-7b	-1.27	-0.93	-1.17	-1.40	0.09	1.59	3.09
3	Not Changed	mmu-let-7a	-1.16	-1.24	-1.25	-1.26	0.19	1.64	3.09
4	Changed	mmu-mir-489	-0.03	-0.02	-0.03	-0.04	0.00	0.04	0.08
4	Changed	mmu-mir-468	0.02	-0.05	-0.02	0.01	0.01	0.02	0.02
4	Changed	mmu-mir-339	-0.05	0.24	0.09	-0.06	-0.07	-0.07	-0.08
4	Changed	mmu-mir-329	0.37	-0.32	-0.44	-0.56	-0.12	0.32	0.76
4	Changed	mmu-mir-20a	0.54	0.26	-0.18	-0.62	-0.31	0.00	0.30
4	Changed	mmu-mir-207	0.19	-0.24	-0.08	0.08	0.05	0.01	-0.02
4	Changed	mmu-mir-17-5p	0.45	0.18	-0.16	-0.49	-0.24	0.01	0.26
4	Changed	mmu-mir-138	-0.08	-0.16	-0.07	0.03	0.06	0.09	0.13
4	Changed	mmu-mir-129-5p	0.30	0.02	0.00	-0.01	-0.06	-0.10	-0.15
4	Not Changed	mmu-mir-98	-0.27	-0.24	-0.24	-0.23	0.05	0.33	0.60
4	Not Changed	mmu-mir-93	0.45	0.05	-0.22	-0.48	-0.21	0.07	0.34
4	Not Changed	mmu-mir-7b	0.08	0.00	0.01	0.01	-0.01	-0.03	-0.05
4	Not Changed	mmu-mir-547	-0.02	0.04	0.01	-0.02	-0.01	0.00	0.00
4	Not Changed	mmu-mir-546	0.13	0.08	0.20	0.33	0.04	-0.25	-0.53
4	Not Changed	mmu-mir-543	0.80	0.04	-0.09	-0.22	-0.20	-0.18	-0.16
4	Not Changed	mmu-mir-542-5p	-0.10	-0.05	-0.07	-0.08	0.01	0.10	0.19
4	Not Changed	mmu-mir-541	0.67	-0.22	-0.37	-0.51	-0.18	0.15	0.48

Class	ES Time Pattern	ProbeID	ES-Day0	ES-Day1	ES-Day2	ES-Day3	ES-Day4	ES-Day5	ES-Day6
4	Not Changed	mmu-mir-540	0.76	0.09	-0.12	-0.32	-0.23	-0.14	-0.04
4	Not Changed	mmu-mir-539	-0.03	-0.32	-0.25	-0.18	0.04	0.26	0.48
4	Not Changed	mmu-mir-494	0.61	0.45	0.36	0.28	-0.14	-0.57	-0.99
4	Not Changed	mmu-mir-487b	-0.09	-0.14	-0.18	-0.22	0.00	0.21	0.42
4	Not Changed	mmu-mir-486	-0.05	-0.03	-0.05	-0.08	-0.01	0.07	0.15
4	Not Changed	mmu-mir-485-5p	0.35	0.08	-0.07	-0.23	-0.14	-0.04	0.05
4	Not Changed	mmu-mir-483	0.01	-0.04	-0.01	0.02	0.01	0.01	0.01
4	Not Changed	mmu-mir-471	0.02	0.00	-0.01	-0.01	0.00	0.00	0.01
4	Not Changed	mmu-mir-470	-0.23	0.00	-0.14	-0.29	-0.03	0.22	0.48
4	Not Changed	mmu-mir-469	-0.02	0.00	-0.01	-0.02	0.00	0.02	0.03
4	Not Changed	mmu-mir-465	0.04	-0.06	-0.02	0.01	0.01	0.01	0.01
4	Not Changed	mmu-mir-464	0.05	-0.03	-0.01	0.01	0.00	-0.01	-0.02
4	Not Changed	mmu-mir-463	-0.01	0.02	0.00	-0.01	-0.01	0.00	0.01
4	Not Changed	mmu-mir-452	0.01	-0.05	-0.01	0.03	0.02	0.01	0.00
4	Not Changed	mmu-mir-451	-0.01	0.20	0.07	-0.06	-0.06	-0.07	-0.07
4	Not Changed	mmu-mir-449	0.11	0.02	0.01	-0.01	-0.03	-0.04	-0.06
4	Not Changed	mmu-mir-448	0.08	0.00	-0.01	-0.02	-0.02	-0.01	-0.01
4	Not Changed	mmu-mir-434-5p	0.30	-0.14	-0.09	-0.04	-0.03	-0.01	0.01
4	Not Changed	mmu-mir-434-3p	0.21	-0.23	-0.27	-0.31	-0.06	0.20	0.46
4	Not Changed	mmu-mir-433-5p	0.07	-0.07	-0.01	0.04	0.02	-0.01	-0.03
4	Not Changed	mmu-mir-433-3p	0.19	-0.46	-0.37	-0.28	0.01	0.30	0.60
4	Not Changed	mmu-mir-431	0.03	-0.39	-0.29	-0.19	0.04	0.28	0.52
4	Not Changed	mmu-mir-429	0.41	0.44	0.12	-0.20	-0.23	-0.26	-0.29
4	Not Changed	mmu-mir-425	0.14	-0.07	-0.13	-0.18	-0.05	0.08	0.21
4	Not Changed	mmu-mir-422b	0.70	0.07	-0.43	-0.94	-0.37	0.20	0.77
4	Not Changed	mmu-mir-412	-0.18	-0.11	0.01	0.14	0.09	0.04	0.00
4	Not Changed	mmu-mir-411	0.16	-0.11	0.01	0.12	0.03	-0.06	-0.15
4	Not Changed	mmu-mir-410	-0.34	-0.27	-0.12	0.03	0.13	0.24	0.34
4	Not Changed	mmu-mir-409	0.42	-0.33	-0.12	0.09	0.04	-0.02	-0.08
4	Not Changed	mmu-mir-384	0.01	0.00	0.01	0.02	0.00	-0.01	-0.03
4	Not Changed	mmu-mir-382	-0.04	-0.72	-0.55	-0.38	0.09	0.56	1.03
4	Not Changed	mmu-mir-381	0.20	0.46	0.13	-0.19	-0.19	-0.20	-0.20
4	Not Changed	mmu-mir-380-5p	-0.03	-0.15	-0.06	0.03	0.05	0.07	0.09
4	Not Changed	mmu-mir-380-3p	-0.56	-0.25	-0.06	0.13	0.19	0.25	0.31
4	Not Changed	mmu-mir-379	0.11	-0.31	-0.43	-0.54	-0.08	0.39	0.86
4	Not Changed	mmu-mir-378	0.19	0.02	-0.02	-0.07	-0.05	-0.04	-0.02
4	Not Changed	mmu-mir-377	0.23	-0.04	0.14	0.32	0.05	-0.22	-0.48
4	Not Changed	mmu-mir-376c	0.13	0.07	0.02	-0.03	-0.05	-0.07	-0.09

Class	ES Time Pattern	ProbeID	ES-Day0	ES-Day1	ES-Day2	ES-Day3	ES-Day4	ES-Day5	ES-Day6
4	Not Changed	S-mmu-mir-376b	-0.10	0.00	0.01	0.02	0.02	0.02	0.02
4	Not Changed	mmu-mir-376b	0.58	0.45	0.13	-0.19	-0.26	-0.33	-0.40
4	Not Changed	S-mmu-mir-376a	-0.07	0.08	0.03	-0.02	-0.01	-0.01	0.00
4	Not Changed	mmu-mir-375	0.00	0.03	0.05	0.07	0.01	-0.05	-0.10
4	Not Changed	mmu-mir-370	-0.18	-0.64	-0.28	0.09	0.21	0.34	0.46
4	Not Changed	mmu-mir-369-5p	-0.20	-0.05	0.02	0.09	0.07	0.05	0.03
4	Not Changed	mmu-mir-369-3p	-0.05	0.01	0.01	0.00	0.01	0.01	0.01
4	Not Changed	mmu-mir-367	0.01	0.08	0.03	-0.02	-0.02	-0.03	-0.04
4	Not Changed	mmu-mir-365	0.02	-0.03	0.01	0.05	0.02	-0.01	-0.04
4	Not Changed	mmu-mir-362	-0.33	0.16	0.02	-0.12	-0.01	0.09	0.20
4	Not Changed	mmu-mir-361	0.48	0.08	-0.25	-0.57	-0.24	0.09	0.41
4	Not Changed	mmu-mir-350	-0.04	0.70	0.27	-0.15	-0.20	-0.26	-0.31
4	Not Changed	mmu-mir-34c	-0.06	0.08	0.01	-0.07	-0.03	0.01	0.06
4	Not Changed	mmu-mir-34b	-0.04	0.05	0.02	0.00	0.00	-0.01	-0.02
4	Not Changed	mmu-mir-346	-0.07	0.03	0.05	0.07	0.02	-0.03	-0.08
4	Not Changed	mmu-mir-345	0.18	0.04	-0.09	-0.23	-0.10	0.03	0.16
4	Not Changed	mmu-mir-342	0.09	-0.26	-0.28	-0.29	-0.02	0.25	0.52
4	Not Changed	mmu-mir-341	1.03	-0.03	-0.19	-0.35	-0.25	-0.15	-0.05
4	Not Changed	mmu-mir-340	-0.08	0.02	0.01	-0.01	0.00	0.02	0.03
4	Not Changed	mmu-mir-338	-0.01	0.10	0.03	-0.04	-0.03	-0.03	-0.03
4	Not Changed	mmu-mir-337	0.07	0.29	0.05	-0.19	-0.13	-0.07	-0.02
4	Not Changed	mmu-mir-331	-0.23	0.08	0.08	0.08	0.04	0.00	-0.04
4	Not Changed	mmu-mir-330	0.07	0.15	-0.06	-0.27	-0.12	0.04	0.20
4	Not Changed	mmu-mir-33	0.01	-0.04	-0.01	0.02	0.01	0.01	0.00
4	Not Changed	mmu-mir-328	-0.05	-0.11	-0.03	0.05	0.05	0.05	0.05
4	Not Changed	mmu-mir-326	-0.15	-0.03	0.02	0.07	0.05	0.02	0.00
4	Not Changed	mmu-mir-325	-0.14	0.02	0.02	0.03	0.03	0.02	0.02
4	Not Changed	mmu-mir-324-5p	-0.21	-0.17	-0.34	-0.51	-0.05	0.41	0.87
4	Not Changed	mmu-mir-324-3p	0.35	0.21	-0.15	-0.50	-0.24	0.03	0.30
4	Not Changed	mmu-mir-323	0.31	0.26	0.13	0.01	-0.12	-0.24	-0.36
4	Not Changed	mmu-mir-320	-0.10	-0.05	-0.08	-0.11	0.00	0.12	0.23
4	Not Changed	mmu-mir-32	0.04	0.01	0.00	-0.02	-0.01	-0.01	-0.01
4	Not Changed	S-mmu-mir-30e	0.02	-0.01	0.00	0.02	0.00	-0.01	-0.02
4	Not Changed	mmu-mir-30e	0.08	0.53	0.19	-0.15	-0.18	-0.22	-0.25
4	Not Changed	mmu-mir-30d	0.73	-0.06	-0.20	-0.34	-0.19	-0.05	0.10
4	Not Changed	mmu-mir-30c	0.42	-0.36	-0.38	-0.41	-0.08	0.25	0.57
4	Not Changed	mmu-mir-30b	0.21	-0.18	-0.21	-0.24	-0.05	0.14	0.33
4	Not Changed	mmu-mir-30a-5p	0.59	0.30	-0.04	-0.38	-0.27	-0.15	-0.04

Class	ES Time Pattern	ProbeID	ES-Day0	ES-Day1	ES-Day2	ES-Day3	ES-Day4	ES-Day5	ES-Day6
4	Not Changed	mmu-mir-30a-3p	0.05	0.09	-0.04	-0.18	-0.08	0.03	0.13
4	Not Changed	S-mmu-mir-302b	-0.01	0.06	0.02	-0.03	-0.02	-0.01	0.00
4	Not Changed	mmu-mir-301	0.24	0.72	0.07	-0.58	-0.37	-0.15	0.07
4	Not Changed	mmu-mir-29c	0.13	0.10	0.05	0.00	-0.05	-0.09	-0.14
4	Not Changed	mmu-mir-29b	0.01	0.19	0.08	-0.03	-0.06	-0.08	-0.10
4	Not Changed	mmu-mir-29a	0.80	0.91	0.29	-0.33	-0.44	-0.56	-0.67
4	Not Changed	mmu-mir-298	0.48	0.20	-0.02	-0.25	-0.19	-0.14	-0.08
4	Not Changed	mmu-mir-296	1.01	-0.10	-0.11	-0.12	-0.17	-0.23	-0.28
4	Not Changed	mmu-mir-291b-3p	-0.01	0.02	0.01	0.00	0.00	-0.01	-0.02
4	Not Changed	mmu-mir-28	0.41	0.00	-0.10	-0.20	-0.12	-0.03	0.05
4	Not Changed	mmu-mir-27b	0.82	0.20	-0.37	-0.93	-0.42	0.09	0.60
4	Not Changed	mmu-mir-27a	0.89	0.72	0.05	-0.62	-0.48	-0.34	-0.21
4	Not Changed	mmu-mir-26b	-0.33	-0.74	-0.58	-0.42	0.14	0.69	1.24
4	Not Changed	mmu-mir-26a	-0.15	-1.02	-0.66	-0.30	0.21	0.71	1.21
4	Not Changed	mmu-mir-25	0.74	0.34	-0.15	-0.64	-0.37	-0.09	0.18
4	Not Changed	mmu-mir-24	0.70	0.00	-0.36	-0.71	-0.29	0.12	0.54
4	Not Changed	mmu-mir-23b	0.59	-0.10	-0.41	-0.72	-0.25	0.21	0.67
4	Not Changed	mmu-mir-23a	1.02	0.39	-0.04	-0.47	-0.39	-0.30	-0.22
4	Not Changed	mmu-mir-224	-0.26	-0.04	-0.18	-0.33	-0.03	0.27	0.57
4	Not Changed	mmu-mir-223	0.44	-0.39	-0.25	-0.11	0.00	0.10	0.21
4	Not Changed	mmu-mir-222	0.29	0.14	-0.17	-0.48	-0.20	0.08	0.35
4	Not Changed	mmu-mir-221	0.30	0.08	-0.23	-0.53	-0.20	0.13	0.45
4	Not Changed	mmu-mir-219	-0.13	-0.16	-0.05	0.05	0.07	0.09	0.11
4	Not Changed	mmu-mir-217	-0.19	-0.18	-0.02	0.13	0.11	0.09	0.07
4	Not Changed	mmu-mir-215	0.07	-0.04	-0.03	-0.01	0.00	0.00	0.01
4	Not Changed	mmu-mir-213	0.03	-0.05	-0.04	-0.04	0.00	0.04	0.07
4	Not Changed	mmu-mir-212	-0.01	-0.10	-0.15	-0.20	-0.02	0.15	0.33
4	Not Changed	mmu-mir-211	0.01	-0.06	-0.01	0.04	0.02	0.00	-0.02
4	Not Changed	mmu-mir-210	1.31	1.13	-0.27	-1.67	-0.92	-0.16	0.59
4	Not Changed	mmu-mir-21	0.32	-0.38	-0.19	0.00	0.04	0.08	0.13
4	Not Changed	mmu-mir-208	0.02	-0.01	0.01	0.02	0.00	-0.02	-0.03
4	Not Changed	mmu-mir-206	-0.13	-0.18	-0.13	-0.09	0.04	0.17	0.30
4	Not Changed	mmu-mir-204	-0.07	-0.07	-0.03	0.02	0.03	0.05	0.07
4	Not Changed	mmu-mir-203	-0.25	-0.13	-0.13	-0.14	0.04	0.21	0.39
4	Not Changed	mmu-mir-202	0.03	-0.05	0.00	0.04	0.02	-0.01	-0.03
4	Not Changed	mmu-mir-201	0.07	0.01	0.00	-0.01	-0.02	-0.02	-0.02
4	Not Changed	mmu-mir-200c	0.98	0.28	-0.20	-0.69	-0.41	-0.12	0.16
4	Not Changed	mmu-mir-19b	1.09	0.68	-0.17	-1.02	-0.61	-0.20	0.21

Class	ES Time Pattern	ProbeID	ES-Day0	ES-Day1	ES-Day2	ES-Day3	ES-Day4	ES-Day5	ES-Day6
4	Not Changed	mmu-mir-19a	1.79	0.98	0.18	-0.62	-0.70	-0.78	-0.86
4	Not Changed	mmu-mir-196b	0.06	-0.05	-0.02	0.01	0.00	0.00	-0.01
4	Not Changed	mmu-mir-196a	0.00	-0.01	0.02	0.06	0.02	-0.02	-0.07
4	Not Changed	mmu-mir-194	0.11	0.38	0.13	-0.12	-0.14	-0.17	-0.19
4	Not Changed	mmu-mir-193	0.06	0.11	0.03	-0.06	-0.05	-0.05	-0.04
4	Not Changed	mmu-mir-192	-0.03	0.08	0.04	0.00	-0.02	-0.03	-0.05
4	Not Changed	mmu-mir-191	1.09	0.26	-0.27	-0.80	-0.45	-0.10	0.25
4	Not Changed	mmu-mir-190	0.04	-0.01	0.01	0.02	0.00	-0.02	-0.04
4	Not Changed	mmu-mir-189	0.10	0.01	0.00	-0.01	-0.02	-0.03	-0.05
4	Not Changed	mmu-mir-187	-0.04	0.20	0.07	-0.07	-0.06	-0.05	-0.05
4	Not Changed	mmu-mir-186	-0.01	0.38	0.11	-0.16	-0.14	-0.11	-0.08
4	Not Changed	mmu-mir-185	0.85	0.29	-0.08	-0.44	-0.32	-0.21	-0.09
4	Not Changed	mmu-mir-184	0.03	0.32	0.04	-0.24	-0.14	-0.05	0.04
4	Not Changed	mmu-mir-181c	-0.19	-0.31	-0.23	-0.16	0.07	0.30	0.52
4	Not Changed	mmu-mir-17-3p	0.72	0.39	-0.10	-0.59	-0.36	-0.14	0.08
4	Not Changed	mmu-mir-16	0.66	0.07	-0.11	-0.29	-0.20	-0.11	-0.03
4	Not Changed	mmu-mir-15b	0.64	0.23	-0.05	-0.32	-0.24	-0.16	-0.08
4	Not Changed	mmu-mir-15a	0.32	0.39	0.19	0.00	-0.15	-0.30	-0.45
4	Not Changed	mmu-mir-155	-0.43	0.09	-0.13	-0.35	-0.04	0.28	0.59
4	Not Changed	mmu-mir-154	0.34	-0.24	-0.26	-0.27	-0.06	0.14	0.35
4	Not Changed	mmu-mir-153	0.00	-0.01	-0.01	0.00	0.00	0.01	0.02
4	Not Changed	mmu-mir-152	-0.34	-0.06	-0.40	-0.74	-0.11	0.52	1.14
4	Not Changed	mmu-mir-151	0.67	0.33	-0.11	-0.55	-0.33	-0.11	0.11
4	Not Changed	mmu-mir-149	0.19	0.42	0.11	-0.19	-0.19	-0.18	-0.17
4	Not Changed	mmu-mir-146	-0.09	-0.22	0.01	0.24	0.13	0.01	-0.10
4	Not Changed	mmu-mir-145	0.45	0.16	-0.21	-0.59	-0.26	0.06	0.39
4	Not Changed	mmu-mir-144	0.00	0.00	-0.01	-0.02	0.00	0.01	0.03
4	Not Changed	mmu-mir-143	0.66	0.01	-0.33	-0.66	-0.28	0.10	0.49
4	Not Changed	mmu-mir-142-5p	0.08	0.05	-0.03	-0.11	-0.05	0.00	0.05
4	Not Changed	mmu-mir-142-3p	0.09	0.07	0.02	-0.04	-0.04	-0.05	-0.05
4	Not Changed	mmu-mir-141	0.07	0.05	0.02	-0.02	-0.03	-0.04	-0.05
4	Not Changed	S-mmu-mir-140	0.23	0.39	-0.13	-0.66	-0.30	0.06	0.42
4	Not Changed	mmu-mir-140	0.05	0.08	0.03	-0.03	-0.04	-0.04	-0.05
4	Not Changed	mmu-mir-139	0.07	0.05	0.00	-0.05	-0.04	-0.02	-0.01
4	Not Changed	mmu-mir-137	0.04	-0.02	-0.01	0.00	0.00	0.00	0.00
4	Not Changed	mmu-mir-136	0.05	-0.04	-0.02	0.01	0.01	0.00	-0.01
4	Not Changed	mmu-mir-135b	0.23	0.07	0.00	-0.06	-0.07	-0.08	-0.08
4	Not Changed	mmu-mir-135a	0.01	-0.07	-0.03	0.02	0.02	0.02	0.03

Class	ES Time Pattern	ProbeID	ES-Day0	ES-Day1	ES-Day2	ES-Day3	ES-Day4	ES-Day5	ES-Day6
4	Not Changed	mmu-mir-133b	0.21	-0.17	-0.05	0.08	0.03	-0.02	-0.07
4	Not Changed	mmu-mir-133a	0.35	-0.15	-0.05	0.06	0.00	-0.07	-0.13
4	Not Changed	mmu-mir-130b	0.75	0.19	-0.48	-1.16	-0.46	0.23	0.93
4	Not Changed	mmu-mir-130a	0.80	0.36	-0.26	-0.88	-0.44	-0.01	0.43
4	Not Changed	mmu-mir-129-3p	0.31	0.11	0.03	-0.06	-0.09	-0.13	-0.17
4	Not Changed	mmu-mir-128b	0.25	0.14	0.01	-0.11	-0.11	-0.10	-0.09
4	Not Changed	mmu-mir-128a	0.26	0.18	0.02	-0.15	-0.13	-0.10	-0.08
4	Not Changed	mmu-mir-127	0.29	-0.59	-0.48	-0.36	0.01	0.38	0.75
4	Not Changed	mmu-mir-126-5p	0.04	0.02	0.01	0.00	-0.01	-0.02	-0.04
4	Not Changed	mmu-mir-126-3p	0.25	-0.15	0.18	0.52	0.12	-0.27	-0.66
4	Not Changed	mmu-mir-124a	1.08	0.02	-0.59	-1.20	-0.49	0.23	0.95
4	Not Changed	mmu-mir-122a	-0.76	-0.81	-0.77	-0.73	0.15	1.02	1.90
4	Not Changed	mmu-mir-107	0.37	-0.07	-0.33	-0.59	-0.19	0.21	0.60
4	Not Changed	mmu-mir-106b	0.55	0.27	-0.17	-0.61	-0.31	-0.01	0.29
4	Not Changed	mmu-mir-106a	0.74	0.50	0.02	-0.46	-0.36	-0.27	-0.17
4	Not Changed	mmu-mir-103	0.26	-0.09	-0.35	-0.61	-0.17	0.27	0.70
4	Not Changed	mmu-mir-1	0.03	0.04	0.02	0.01	-0.01	-0.03	-0.05
4	Not Changed	mmu-let-7g	-0.54	-0.27	-0.61	-0.95	-0.08	0.79	1.66
4	Not Changed	S-mmu-let-7d	0.88	-0.06	-0.09	-0.11	-0.16	-0.21	-0.26
4	Not Changed	kshv-miR-K12-10a	0.70	-0.29	-0.17	-0.06	-0.06	-0.06	-0.06
4	Not Changed	hcmv-miR-UL22A-1	-0.03	0.04	0.02	0.01	0.00	-0.01	-0.02
1	Changed	MIR70	3.54	3.05	0.34	-2.37	-1.94	-1.52	-1.09
1	Changed	MIR43	0.63	0.74	0.21	-0.33	-0.37	-0.42	-0.46
1	Not Changed	MIR4	0.67	0.97	0.23	-0.52	-0.48	-0.45	-0.41
1	Not Changed	MIR30	1.43	1.12	0.34	-0.44	-0.63	-0.82	-1.01
1	Not Changed	MIR253	1.29	0.57	0.03	-0.52	-0.49	-0.46	-0.43
1	Not Changed	MIR216	0.89	0.64	0.08	-0.48	-0.43	-0.37	-0.32
1	Not Changed	MIR136	1.05	0.91	0.24	-0.44	-0.51	-0.59	-0.66
3	Changed	MIR77	-2.27	-2.07	-1.49	-0.91	0.67	2.24	3.82
3	Changed	MIR75	-2.53	-2.17	-0.99	0.19	1.01	1.83	2.65
3	Changed	MIR35	-2.70	-2.61	-1.12	0.36	1.19	2.02	2.85
3	Changed	MIR141	-0.50	-0.55	-0.32	-0.09	0.20	0.49	0.78
4	Changed	MIR79	-0.02	-0.04	-0.04	-0.03	0.01	0.04	0.08
4	Not Changed	MIR88	-0.74	-0.10	-0.03	0.05	0.16	0.27	0.38
4	Not Changed	MIR85	1.08	0.10	-0.62	-1.35	-0.54	0.26	1.07
4	Not Changed	MIR74	0.15	-0.05	-0.39	-0.74	-0.20	0.34	0.88
4	Not Changed	MIR71	0.01	0.09	0.02	-0.04	-0.03	-0.03	-0.02
4	Not Changed	MIR61	-0.07	0.48	0.69	0.90	0.11	-0.67	-1.45

Class	ES Time Pattern	ProbeID	ES-Day0	ES-Day1	ES-Day2	ES-Day3	ES-Day4	ES-Day5	ES-Day6
4	Not Changed	MIR52	-0.02	-0.02	-0.01	0.01	0.01	0.01	0.01
4	Not Changed	MIR47	0.02	0.03	0.01	-0.01	-0.01	-0.02	-0.02
4	Not Changed	MIR41	-0.04	0.26	0.13	0.00	-0.06	-0.12	-0.18
4	Not Changed	MIR257	0.51	0.30	0.01	-0.29	-0.23	-0.18	-0.12
4	Not Changed	MIR255	0.06	0.05	0.00	-0.05	-0.03	-0.02	-0.01
4	Not Changed	MIR237	0.12	-0.08	-0.04	-0.01	0.00	0.01	0.01
4	Not Changed	MIR220	0.08	-0.03	-0.04	-0.05	-0.02	0.01	0.05
4	Not Changed	MIR207	-0.26	-0.38	-0.09	0.21	0.19	0.17	0.15
4	Not Changed	MIR206	-1.60	-0.89	-0.01	0.87	0.71	0.55	0.39
4	Not Changed	MIR202	0.74	0.11	-0.06	-0.23	-0.21	-0.19	-0.16
4	Not Changed	MIR201	0.03	0.02	0.02	0.03	0.00	-0.04	-0.07
4	Not Changed	MIR188	0.00	0.01	-0.01	-0.03	-0.01	0.01	0.04
4	Not Changed	MIR184	0.08	-0.06	-0.05	-0.04	-0.01	0.02	0.05
4	Not Changed	MIR180	0.06	-0.02	-0.01	-0.01	-0.01	-0.01	0.00
4	Not Changed	MIR177	0.02	-0.09	-0.02	0.04	0.03	0.02	0.01
4	Not Changed	MIR169	0.04	-0.01	-0.03	-0.05	-0.02	0.02	0.05
4	Not Changed	MIR167	0.19	-0.06	-0.05	-0.03	-0.02	-0.02	-0.01
4	Not Changed	MIR166	-0.63	-0.55	-0.11	0.34	0.33	0.32	0.31
4	Not Changed	MIR165	-0.66	-0.13	0.00	0.12	0.17	0.22	0.27
4	Not Changed	MIR161	0.03	0.09	0.01	-0.06	-0.04	-0.02	0.00
4	Not Changed	MIR140	0.05	0.03	0.00	-0.03	-0.02	-0.01	0.00
4	Not Changed	MIR124	0.36	0.16	-0.11	-0.37	-0.19	-0.01	0.17
4	Not Changed	MIR122	0.01	-0.06	-0.01	0.04	0.02	0.01	-0.01
4	Not Changed	MIR121	0.65	0.49	0.09	-0.31	-0.31	-0.31	-0.31
4	Not Changed	MIR12	0.01	0.20	0.01	-0.18	-0.09	-0.01	0.07
4	Not Changed	MIR112	-0.46	-0.08	0.05	0.18	0.14	0.10	0.07
4	Not Changed	MIR103	-0.12	-0.15	-0.12	-0.10	0.03	0.16	0.29
4	Not Changed	MIR102	-0.38	0.17	0.11	0.05	0.03	0.02	0.01
4	Not Changed	MIR100	0.04	-0.03	-0.01	0.01	0.00	0.00	-0.01
1	Changed	cand97	1.06	0.81	0.11	-0.59	-0.53	-0.46	-0.40
1	Changed	cand78:a	1.53	1.75	0.44	-0.87	-0.91	-0.95	-0.99
1	Changed	cand647	1.41	1.08	0.24	-0.60	-0.66	-0.71	-0.77
1	Changed	cand465:a	3.30	2.86	0.53	-1.81	-1.72	-1.63	-1.53
1	Changed	cand324:b	-0.09	0.34	0.13	-0.07	-0.09	-0.10	-0.12
1	Changed	cand317	0.62	0.91	0.24	-0.43	-0.44	-0.45	-0.45
1	Changed	cand268:b	1.53	0.92	-0.01	-0.93	-0.72	-0.50	-0.29
1	Changed	cand135:a	-0.11	0.64	0.21	-0.22	-0.20	-0.17	-0.15
1	Changed	cand1:b	0.51	0.63	0.19	-0.26	-0.31	-0.35	-0.40

Class	ES Time Pattern	ProbeID	ES-Day0	ES-Day1	ES-Day2	ES-Day3	ES-Day4	ES-Day5	ES-Day6
1	Not Changed	cand563	0.96	1.19	0.60	0.01	-0.46	-0.92	-1.38
1	Not Changed	cand467	0.55	0.78	0.52	0.26	-0.22	-0.70	-1.19
1	Not Changed	cand465:b	0.50	0.58	0.13	-0.31	-0.31	-0.30	-0.30
1	Not Changed	cand342:a	1.20	1.04	0.26	-0.51	-0.59	-0.66	-0.74
1	Not Changed	cand324:a	1.68	1.55	0.46	-0.64	-0.83	-1.02	-1.21
1	Not Changed	cand276:b	0.06	0.49	0.17	-0.16	-0.17	-0.19	-0.20
1	Not Changed	cand172:b	1.27	0.88	0.15	-0.57	-0.58	-0.58	-0.58
1	Not Changed	cand156	1.09	1.80	0.89	-0.01	-0.63	-1.25	-1.87
1	Not Changed	cand144:a	1.12	0.91	-0.01	-0.94	-0.65	-0.36	-0.07
1	Not Changed	cand1:a	0.02	0.58	0.23	-0.11	-0.17	-0.24	-0.31
2	Changed	cand79	-0.50	-0.29	0.14	0.58	0.30	0.02	-0.25
2	Changed	cand284:b	-0.70	0.20	0.35	0.50	0.19	-0.12	-0.42
2	Changed	cand255	-0.51	0.02	0.27	0.52	0.21	-0.10	-0.41
2	Not Changed	cand595:a	-1.00	0.42	0.72	1.02	0.32	-0.39	-1.09
2	Not Changed	cand342:b	-0.96	0.31	0.53	0.75	0.27	-0.21	-0.69
2	Not Changed	cand149:b	-0.41	0.21	0.61	1.01	0.27	-0.47	-1.22
3	Changed	cand90:b	-1.60	-0.92	-0.60	-0.28	0.42	1.13	1.84
3	Changed	cand90:a	-3.52	-2.15	-1.11	-0.07	1.11	2.28	3.46
3	Changed	cand516	-0.57	-0.11	0.07	0.25	0.19	0.12	0.05
3	Changed	cand375	-4.08	-3.32	-1.06	1.19	1.81	2.43	3.04
3	Changed	cand306	-0.49	-0.34	-0.08	0.17	0.21	0.25	0.28
3	Changed	cand278:a	-0.99	-0.60	-0.66	-0.72	0.14	0.99	1.85
3	Changed	cand27	-0.84	-0.53	-0.23	0.06	0.29	0.51	0.73
3	Not Changed	cand709	-1.09	-0.78	-0.97	-1.15	0.09	1.33	2.57
3	Not Changed	cand618	-1.64	-1.00	-0.13	0.74	0.71	0.68	0.65
3	Not Changed	cand564:a	-0.36	-0.23	-0.22	-0.21	0.06	0.34	0.61
3	Not Changed	cand490	-1.07	-0.78	-0.95	-1.13	0.09	1.31	2.53
3	Not Changed	cand374:a	-1.78	-2.03	-1.11	-0.19	0.76	1.71	2.66
3	Not Changed	cand371:a	-0.97	-0.64	-0.15	0.33	0.40	0.48	0.55
3	Not Changed	cand268:a	-2.40	-1.44	-0.30	0.84	0.97	1.10	1.22
3	Not Changed	cand231	-0.91	-0.97	-0.29	0.40	0.49	0.59	0.69
3	Not Changed	cand161	-0.83	-0.85	-1.02	-1.20	0.05	1.30	2.55
3	Not Changed	cand119	-1.25	-0.79	-0.98	-1.17	0.11	1.39	2.67
3	Not Changed	cand118	-0.84	-1.00	-1.09	-1.18	0.09	1.37	2.64
3	Not Changed	cand106:a	-1.05	-0.65	-0.79	-0.94	0.10	1.15	2.19
4	Changed	cand64	-0.36	-0.07	0.01	0.08	0.10	0.11	0.13
4	Changed	cand594	-0.03	0.10	0.05	0.00	-0.02	-0.04	-0.05
4	Changed	cand501	0.09	-0.05	-0.15	-0.25	-0.07	0.12	0.31

Class	ES Time Pattern	ProbeID	ES-Day0	ES-Day1	ES-Day2	ES-Day3	ES-Day4	ES-Day5	ES-Day6
4	Changed	cand500:a	-0.43	-0.03	0.08	0.19	0.12	0.06	0.00
4	Changed	cand497:a	0.10	-0.02	0.11	0.25	0.05	-0.15	-0.34
4	Changed	cand457:b	0.06	0.01	0.04	0.07	0.01	-0.06	-0.12
4	Changed	cand427	0.06	0.01	0.02	0.04	0.00	-0.05	-0.09
4	Changed	cand361	0.05	-0.10	-0.05	0.00	0.02	0.03	0.04
4	Changed	cand349:a	-0.04	-0.23	-0.13	-0.02	0.06	0.14	0.22
4	Changed	cand346	0.07	-0.09	-0.17	-0.25	-0.05	0.14	0.34
4	Changed	cand309	-0.22	0.10	0.02	-0.06	0.00	0.06	0.11
4	Changed	cand279:b	-0.42	-0.21	0.07	0.34	0.21	0.07	-0.06
4	Changed	cand262	-0.05	0.44	0.09	-0.26	-0.17	-0.08	0.02
4	Changed	cand25	-0.01	0.05	0.02	0.00	-0.01	-0.02	-0.02
4	Changed	cand226	-0.10	-0.09	-0.04	0.00	0.04	0.07	0.11
4	Changed	cand203	0.06	0.28	0.09	-0.10	-0.10	-0.11	-0.11
4	Changed	cand153:b	-0.04	0.13	0.04	-0.04	-0.03	-0.03	-0.03
4	Changed	cand149:a	0.06	-0.14	-0.08	-0.02	0.02	0.06	0.09
4	Changed	cand146	-0.44	-0.13	-0.09	-0.04	0.10	0.23	0.37
4	Changed	cand120	0.00	0.02	-0.04	-0.10	-0.03	0.04	0.11
4	Changed	cand11:b	-0.03	0.07	0.04	0.01	-0.01	-0.03	-0.05
4	Not Changed	cand91:b	-0.13	0.03	0.06	0.10	0.04	-0.02	-0.08
4	Not Changed	cand91:a	-1.45	-1.02	-0.02	0.98	0.74	0.50	0.27
4	Not Changed	cand82	0.01	0.11	0.05	-0.02	-0.03	-0.05	-0.07
4	Not Changed	cand78:b	-0.30	-0.28	-0.07	0.13	0.15	0.17	0.19
4	Not Changed	cand73	0.37	-0.10	0.07	0.24	0.02	-0.19	-0.40
4	Not Changed	cand718:b	-1.02	-0.56	-0.11	0.35	0.40	0.45	0.49
4	Not Changed	cand718:a	0.03	-0.03	-0.01	0.01	0.00	0.00	0.00
4	Not Changed	cand708:b	-0.30	0.45	0.36	0.26	0.00	-0.26	-0.51
4	Not Changed	cand708:a	-0.04	0.04	0.05	0.05	0.01	-0.03	-0.08
4	Not Changed	cand707	0.00	0.37	0.10	-0.17	-0.13	-0.10	-0.07
4	Not Changed	cand706	-0.42	-0.22	0.04	0.31	0.20	0.10	-0.01
4	Not Changed	cand705	0.06	-0.06	0.01	0.07	0.02	-0.03	-0.08
4	Not Changed	cand70	-0.30	-0.10	0.11	0.32	0.16	-0.01	-0.17
4	Not Changed	cand7	0.82	-0.11	0.12	0.35	-0.02	-0.40	-0.77
4	Not Changed	cand699	-1.16	-0.42	-0.03	0.35	0.39	0.42	0.46
4	Not Changed	cand692:b	-0.37	-0.23	-0.04	0.16	0.16	0.16	0.16
4	Not Changed	cand692:a	-1.12	-0.27	0.05	0.37	0.35	0.32	0.29
4	Not Changed	cand690	-0.41	0.35	0.77	1.19	0.28	-0.63	-1.54
4	Not Changed	cand686	-0.04	-0.61	-0.40	-0.19	0.11	0.42	0.72
4	Not Changed	cand68	-1.11	-0.69	-0.24	0.22	0.41	0.61	0.80

Class	ES Time Pattern	ProbeID	ES-Day0	ES-Day1	ES-Day2	ES-Day3	ES-Day4	ES-Day5	ES-Day6
4	Not Changed	cand678	-0.48	0.23	0.21	0.19	0.07	-0.05	-0.17
4	Not Changed	cand669	-0.19	0.06	0.07	0.08	0.04	0.00	-0.04
4	Not Changed	cand667:b	-0.49	-0.04	0.17	0.37	0.18	0.00	-0.19
4	Not Changed	cand667:a	-0.41	0.11	0.14	0.17	0.08	0.00	-0.09
4	Not Changed	cand664	-0.16	0.04	0.10	0.16	0.06	-0.05	-0.15
4	Not Changed	cand650	-0.10	0.10	0.03	-0.04	-0.02	0.01	0.03
4	Not Changed	cand65	-0.71	-0.49	-0.14	0.21	0.29	0.38	0.47
4	Not Changed	cand648	0.28	0.60	0.59	0.58	-0.05	-0.68	-1.31
4	Not Changed	cand624	-0.15	0.08	0.05	0.02	0.01	0.00	-0.01
4	Not Changed	cand619	-0.30	0.00	0.09	0.17	0.09	0.01	-0.06
4	Not Changed	cand617	0.27	0.42	0.35	0.27	-0.08	-0.44	-0.79
4	Not Changed	cand616:b	-0.36	0.16	0.15	0.14	0.06	-0.03	-0.12
4	Not Changed	cand616:a	-0.79	-0.59	-0.08	0.42	0.38	0.34	0.30
4	Not Changed	cand614	-0.05	0.17	0.06	-0.06	-0.05	-0.04	-0.03
4	Not Changed	cand613	-0.43	0.05	0.07	0.10	0.08	0.07	0.05
4	Not Changed	cand6	-0.07	-0.08	-0.05	-0.01	0.03	0.07	0.11
4	Not Changed	cand595:b	-0.25	0.08	0.07	0.06	0.04	0.01	-0.01
4	Not Changed	cand590	-0.03	0.05	0.01	-0.03	-0.01	0.00	0.01
4	Not Changed	cand588	-1.18	-0.67	-0.07	0.54	0.50	0.46	0.42
4	Not Changed	cand585	-0.09	0.09	0.05	0.01	0.00	-0.02	-0.03
4	Not Changed	cand572:b	-0.04	0.31	0.12	-0.07	-0.09	-0.11	-0.12
4	Not Changed	cand572:a	-0.62	-0.15	0.10	0.34	0.23	0.11	-0.01
4	Not Changed	cand570	-0.51	0.39	0.40	0.41	0.09	-0.23	-0.55
4	Not Changed	cand57	-1.02	-0.32	0.14	0.60	0.40	0.20	0.00
4	Not Changed	cand564:b	0.34	-0.42	-0.44	-0.45	-0.07	0.32	0.71
4	Not Changed	cand557	-0.31	-0.33	-0.17	-0.02	0.13	0.28	0.42
4	Not Changed	cand549	-0.07	0.07	0.03	-0.01	-0.01	-0.01	-0.01
4	Not Changed	cand545	-0.63	-0.26	0.20	0.66	0.33	0.01	-0.31
4	Not Changed	cand541	-1.02	-0.43	0.13	0.70	0.45	0.21	-0.04
4	Not Changed	cand532:b	-0.23	0.04	0.49	0.94	0.26	-0.41	-1.09
4	Not Changed	cand532:a	-0.99	-1.29	-0.45	0.39	0.59	0.78	0.97
4	Not Changed	cand529	-0.43	-0.24	0.00	0.25	0.20	0.14	0.09
4	Not Changed	cand524	-0.01	0.04	0.02	0.00	-0.01	-0.02	-0.02
4	Not Changed	cand523	-0.28	0.39	0.27	0.16	-0.01	-0.18	-0.35
4	Not Changed	cand515	-0.75	-0.61	-0.13	0.34	0.36	0.38	0.40
4	Not Changed	cand500:b	-0.17	0.17	0.07	-0.02	-0.02	-0.02	-0.02
4	Not Changed	cand50	0.01	0.12	0.04	-0.05	-0.04	-0.04	-0.04
4	Not Changed	cand5	-0.71	-0.62	-0.26	0.09	0.30	0.50	0.70

Class	ES Time Pattern	ProbeID	ES-Day0	ES-Day1	ES-Day2	ES-Day3	ES-Day4	ES-Day5	ES-Day6
4	Not Changed	cand497:b	-0.05	0.10	0.05	0.00	-0.02	-0.03	-0.05
4	Not Changed	cand492	0.82	-0.26	0.03	0.32	0.01	-0.30	-0.62
4	Not Changed	cand489	-0.70	-0.16	0.01	0.18	0.20	0.23	0.25
4	Not Changed	cand462	0.97	0.18	0.35	0.53	-0.07	-0.68	-1.28
4	Not Changed	cand459	-1.23	-0.41	0.14	0.69	0.48	0.27	0.07
4	Not Changed	cand457:a	0.30	0.83	0.36	-0.11	-0.29	-0.46	-0.64
4	Not Changed	cand445	-0.66	0.62	0.52	0.42	0.06	-0.30	-0.66
4	Not Changed	cand425	-0.13	0.17	0.09	0.01	-0.02	-0.05	-0.08
4	Not Changed	cand42:b	0.01	-0.39	-0.21	-0.03	0.09	0.20	0.32
4	Not Changed	cand42:a	-1.28	-0.02	0.26	0.53	0.35	0.17	-0.01
4	Not Changed	cand418	0.00	0.48	0.30	0.12	-0.09	-0.30	-0.51
4	Not Changed	cand415	-0.17	-0.14	-0.10	-0.07	0.04	0.16	0.28
4	Not Changed	cand412	0.55	0.17	0.29	0.40	-0.03	-0.47	-0.91
4	Not Changed	cand40	0.05	-0.02	-0.02	-0.03	-0.01	0.01	0.03
4	Not Changed	cand386:b	0.14	0.44	0.18	-0.08	-0.15	-0.23	-0.30
4	Not Changed	cand386:a	0.14	-0.01	-0.01	-0.02	-0.03	-0.03	-0.04
4	Not Changed	cand385	-0.40	-0.24	-0.03	0.18	0.17	0.16	0.16
4	Not Changed	cand374:b	-1.17	-0.34	0.08	0.51	0.41	0.31	0.20
4	Not Changed	cand371:b	0.25	0.10	-0.10	-0.31	-0.14	0.02	0.18
4	Not Changed	cand362	-0.22	-0.11	0.23	0.57	0.21	-0.16	-0.52
4	Not Changed	cand352:b	-0.06	-0.03	-0.03	-0.03	0.01	0.05	0.09
4	Not Changed	cand352:a	-0.01	-0.01	-0.02	-0.04	-0.01	0.03	0.06
4	Not Changed	cand351	0.18	0.08	0.01	-0.06	-0.07	-0.07	-0.07
4	Not Changed	cand350	0.47	0.55	-0.07	-0.70	-0.39	-0.08	0.23
4	Not Changed	cand35	0.01	0.11	0.08	0.05	-0.02	-0.08	-0.15
4	Not Changed	cand349:b	0.10	0.02	-0.02	-0.05	-0.03	-0.01	0.01
4	Not Changed	cand348	-0.79	-0.31	-0.61	-0.90	-0.02	0.87	1.76
4	Not Changed	cand347	-0.86	-0.64	-0.16	0.32	0.39	0.45	0.51
4	Not Changed	cand345	0.12	0.70	0.56	0.41	-0.09	-0.59	-1.10
4	Not Changed	cand341	1.33	1.11	0.04	-1.03	-0.76	-0.48	-0.21
4	Not Changed	cand340	-0.24	-0.26	-0.11	0.03	0.11	0.19	0.27
4	Not Changed	cand337	0.28	0.42	-0.11	-0.65	-0.31	0.02	0.36
4	Not Changed	cand336	0.15	0.26	-0.09	-0.45	-0.20	0.04	0.29
4	Not Changed	cand334:b	-0.11	0.06	0.03	0.00	0.00	0.00	0.00
4	Not Changed	cand334:a	-0.58	-0.05	0.30	0.64	0.27	-0.10	-0.47
4	Not Changed	cand315:b	0.49	0.16	-0.19	-0.55	-0.26	0.03	0.32
4	Not Changed	cand315:a	0.41	0.33	-0.06	-0.46	-0.26	-0.07	0.12
4	Not Changed	cand314	0.47	-0.07	-0.28	-0.49	-0.18	0.12	0.43

Class	ES Time Pattern	ProbeID	ES-Day0	ES-Day1	ES-Day2	ES-Day3	ES-Day4	ES-Day5	ES-Day6
4	Not Changed	cand304	0.60	0.22	-0.32	-0.87	-0.37	0.12	0.62
4	Not Changed	cand302:b	-0.25	-0.07	0.01	0.08	0.08	0.08	0.07
4	Not Changed	cand302:a	-0.05	0.07	0.05	0.03	0.00	-0.04	-0.07
4	Not Changed	cand297:b	0.65	0.40	0.15	-0.11	-0.24	-0.36	-0.49
4	Not Changed	cand297:a	-0.04	0.11	0.04	-0.03	-0.03	-0.03	-0.02
4	Not Changed	cand294	-0.61	-0.54	-0.41	-0.29	0.16	0.62	1.07
4	Not Changed	cand286	-0.97	-0.67	-0.11	0.46	0.44	0.43	0.42
4	Not Changed	cand284:a	0.51	-0.09	-0.29	-0.48	-0.18	0.12	0.41
4	Not Changed	cand279:a	-0.59	-0.26	0.14	0.54	0.30	0.05	-0.19
4	Not Changed	cand278:b	0.01	0.07	0.04	0.00	-0.02	-0.04	-0.06
4	Not Changed	cand276:a	0.73	0.73	0.12	-0.48	-0.42	-0.37	-0.31
4	Not Changed	cand275	-0.34	0.23	0.24	0.25	0.06	-0.13	-0.32
4	Not Changed	cand271	-0.07	0.08	0.05	0.02	0.00	-0.03	-0.05
4	Not Changed	cand26	-0.31	0.00	-0.05	-0.11	0.03	0.16	0.29
4	Not Changed	cand252	0.49	0.32	-0.07	-0.46	-0.28	-0.09	0.09
4	Not Changed	cand244	-0.69	0.38	0.35	0.33	0.10	-0.12	-0.35
4	Not Changed	cand24	-0.31	-0.13	-0.02	0.09	0.11	0.12	0.14
4	Not Changed	cand234	0.07	0.11	0.03	-0.05	-0.05	-0.06	-0.06
4	Not Changed	cand224	-0.05	-0.04	0.03	0.09	0.04	-0.01	-0.06
4	Not Changed	cand22	0.02	0.06	0.00	-0.06	-0.03	-0.01	0.01
4	Not Changed	cand213	-0.02	0.22	-0.02	-0.26	-0.12	0.03	0.18
4	Not Changed	cand212	-0.35	0.15	0.00	-0.16	-0.02	0.12	0.25
4	Not Changed	cand210:b	-0.30	0.03	0.05	0.07	0.06	0.05	0.04
4	Not Changed	cand210:a	0.77	0.37	0.06	-0.25	-0.28	-0.32	-0.35
4	Not Changed	cand202	-0.05	-0.03	-0.01	0.01	0.02	0.03	0.04
4	Not Changed	cand200	0.24	-0.34	0.43	1.19	0.34	-0.50	-1.35
4	Not Changed	cand192	-0.74	-0.39	0.13	0.66	0.38	0.11	-0.16
4	Not Changed	cand186	-0.31	-0.15	-0.02	0.12	0.12	0.12	0.12
4	Not Changed	cand184	-0.04	0.03	-0.05	-0.12	-0.03	0.06	0.15
4	Not Changed	cand179:b	0.00	-0.01	0.01	0.03	0.01	-0.01	-0.02
4	Not Changed	cand179:a	-0.26	0.88	0.61	0.34	-0.09	-0.52	-0.95
4	Not Changed	cand178:b	-0.12	0.01	0.00	-0.01	0.01	0.04	0.06
4	Not Changed	cand178:a	0.07	0.52	0.60	0.68	0.03	-0.62	-1.27
4	Not Changed	cand172:a	0.03	0.08	0.02	-0.04	-0.04	-0.03	-0.03
4	Not Changed	cand157	-0.48	-0.30	-0.54	-0.78	-0.04	0.70	1.44
4	Not Changed	cand153:a	0.16	0.15	0.01	-0.13	-0.10	-0.06	-0.03
4	Not Changed	cand152:b	1.00	0.52	0.00	-0.51	-0.42	-0.34	-0.25
4	Not Changed	cand152:a	-0.15	-0.14	-0.10	-0.05	0.05	0.14	0.24

Class	ES Time Pattern	ProbeID	ES-Day0	ES-Day1	ES-Day2	ES-Day3	ES-Day4	ES-Day5	ES-Day6
4	Not Changed	cand151	-0.53	-0.20	0.05	0.30	0.21	0.13	0.05
4	Not Changed	cand144:b	-0.05	0.10	0.06	0.03	-0.01	-0.04	-0.08
4	Not Changed	cand139	-0.04	0.04	0.01	-0.02	-0.01	0.00	0.01
4	Not Changed	cand137:b	0.06	0.23	0.04	-0.15	-0.11	-0.06	-0.01
4	Not Changed	cand137:a	0.41	0.22	-0.17	-0.56	-0.26	0.03	0.33
4	Not Changed	cand135:b	-0.29	0.08	-0.05	-0.18	-0.02	0.14	0.31
4	Not Changed	cand130	-0.01	0.09	0.04	0.00	-0.02	-0.04	-0.06
4	Not Changed	cand13	-0.13	0.19	0.13	0.06	-0.01	-0.08	-0.15
4	Not Changed	cand129	0.06	0.08	0.01	-0.05	-0.04	-0.03	-0.02
4	Not Changed	cand126	0.14	0.63	0.40	0.17	-0.14	-0.45	-0.76
4	Not Changed	cand116	-0.70	0.16	0.24	0.33	0.16	-0.01	-0.17
4	Not Changed	cand115	0.60	-0.09	0.26	0.62	0.08	-0.46	-1.01
4	Not Changed	cand11:a	0.02	0.02	0.00	-0.02	-0.01	0.00	0.01
4	Not Changed	cand109	-0.31	0.24	0.16	0.08	0.01	-0.06	-0.13
4	Not Changed	cand106:b	0.28	0.08	-0.05	-0.18	-0.11	-0.04	0.02
4	Not Changed	cand104:b	-0.07	0.21	0.02	-0.17	-0.08	0.00	0.09
4	Not Changed	cand104:a	0.93	0.55	-0.19	-0.93	-0.52	-0.12	0.28
4	Not Changed	cand103:b	0.69	0.34	-0.23	-0.81	-0.40	0.00	0.41
4	Not Changed	cand103:a	0.16	0.36	0.08	-0.19	-0.16	-0.14	-0.11
4	Not Changed	cand100	0.07	0.04	0.01	-0.03	-0.03	-0.03	-0.03
1	Changed	S-mmu-mir-467	1.77	1.50	0.50	-0.51	-0.80	-1.09	-1.37
1	Changed	S-mmu-mir-425	1.38	0.84	0.13	-0.57	-0.58	-0.59	-0.61
1	Changed	S-mmu-mir-295	2.14	2.03	0.66	-0.70	-1.04	-1.37	-1.71
1	Changed	S-mmu-mir-294	2.87	2.50	0.70	-1.10	-1.38	-1.66	-1.93
1	Changed	S-mmu-mir-210	0.59	0.53	0.11	-0.31	-0.31	-0.30	-0.30
1	Not Changed	S-mmu-mir-293	2.63	2.99	0.85	-1.30	-1.51	-1.73	-1.94
1	Not Changed	S-mmu-mir-290	1.71	0.37	-0.01	-0.39	-0.47	-0.56	-0.64
1	Not Changed	S-mmu-mir-27a	0.20	0.45	0.16	-0.14	-0.18	-0.22	-0.27
1	Not Changed	S-mmu-mir-25	0.88	0.37	0.10	-0.18	-0.29	-0.39	-0.49
1	Not Changed	S-mmu-mir-24-2	0.59	0.69	0.13	-0.42	-0.38	-0.33	-0.28
1	Not Changed	S-mmu-mir-183	1.10	0.85	0.22	-0.41	-0.50	-0.58	-0.67
1	Not Changed	S-mmu-mir-181c	0.04	0.38	0.09	-0.21	-0.15	-0.10	-0.04
1	Not Changed	S-mmu-mir-18	0.64	0.48	0.09	-0.30	-0.30	-0.30	-0.31
2	Not Changed	S-mmu-mir-21	-1.04	-0.26	0.38	1.03	0.50	-0.03	-0.56
3	Changed	S-mmu-mir-351	-0.86	-0.98	-0.45	0.08	0.41	0.73	1.06
3	Changed	S-mmu-mir-345	-0.38	-0.11	-0.18	-0.24	0.03	0.30	0.58
3	Changed	S-mmu-mir-219-2	-1.73	-1.85	-0.51	0.83	0.96	1.09	1.22
3	Changed	S-mmu-mir-199b	-2.17	-2.02	-1.25	-0.48	0.75	1.98	3.21

Class	ES Time Pattern	ProbeID	ES-Day0	ES-Day1	ES-Day2	ES-Day3	ES-Day4	ES-Day5	ES-Day6
3	Changed	S-mmu-mir-135a-2	-0.63	-0.83	-0.40	0.03	0.32	0.61	0.91
3	Changed	S-mmu-mir-125b-1	-0.49	-0.63	-0.39	-0.14	0.21	0.55	0.89
3	Changed	S-mmu-mir-10b	-1.06	-1.17	-0.76	-0.35	0.38	1.11	1.84
3	Changed	S-mmu-mir-10a	-1.77	-1.67	-0.69	0.30	0.79	1.28	1.77
3	Not Changed	S-mmu-mir-412	-0.63	-0.45	-0.20	0.05	0.23	0.41	0.59
3	Not Changed	S-mmu-mir-181a	-0.19	-0.23	-0.13	-0.02	0.08	0.19	0.30
4	Changed	S-mmu-mir-469	-0.36	-0.11	0.02	0.15	0.13	0.10	0.07
4	Changed	S-mmu-mir-350	-0.05	-0.01	0.00	0.00	0.01	0.02	0.03
4	Changed	S-mmu-mir-296	0.06	-0.09	-0.12	-0.16	-0.03	0.11	0.24
4	Changed	S-mmu-mir-23a	0.23	0.24	0.14	0.04	-0.09	-0.22	-0.35
4	Changed	S-mmu-mir-223	0.11	0.03	0.01	-0.02	-0.03	-0.05	-0.06
4	Changed	S-mmu-mir-193	-0.13	-0.07	-0.08	-0.08	0.02	0.12	0.22
4	Changed	S-mmu-let-7e	0.07	-0.06	-0.02	0.03	0.01	-0.01	-0.02
4	Not Changed	S-mmu-mir-99b	-0.58	0.06	-0.04	-0.14	0.05	0.24	0.43
4	Not Changed	S-mmu-mir-99a	0.17	-0.06	-0.03	0.00	-0.01	-0.03	-0.05
4	Not Changed	S-mmu-mir-98	0.03	0.01	-0.05	-0.11	-0.03	0.04	0.11
4	Not Changed	S-mmu-mir-96	0.13	-0.03	-0.01	0.00	-0.01	-0.03	-0.05
4	Not Changed	S-mmu-mir-93	0.45	0.37	-0.04	-0.46	-0.28	-0.11	0.07
4	Not Changed	S-mmu-mir-92-2	0.17	-0.04	0.45	0.94	0.22	-0.51	-1.23
4	Not Changed	S-mmu-mir-92-1	0.30	-0.14	-0.05	0.04	0.00	-0.05	-0.09
4	Not Changed	S-mmu-mir-7b	0.12	0.09	-0.06	-0.21	-0.09	0.02	0.13
4	Not Changed	S-mmu-mir-7-2	0.12	0.04	-0.01	-0.06	-0.05	-0.03	-0.02
4	Not Changed	S-mmu-mir-7-1	0.03	0.04	0.00	-0.04	-0.02	-0.01	0.00
4	Not Changed	S-mmu-mir-486	0.01	0.22	0.13	0.04	-0.05	-0.13	-0.22
4	Not Changed	S-mmu-mir-484	-0.55	-0.23	-0.10	0.04	0.16	0.28	0.40
4	Not Changed	S-mmu-mir-483	-0.35	-0.25	-0.32	-0.39	0.02	0.44	0.85
4	Not Changed	S-mmu-mir-471	0.13	-0.03	-0.04	-0.06	-0.03	0.00	0.02
4	Not Changed	S-mmu-mir-468	0.04	0.09	0.00	-0.10	-0.05	-0.01	0.03
4	Not Changed	S-mmu-mir-466	0.06	0.00	-0.02	-0.03	-0.02	0.00	0.01
4	Not Changed	S-mmu-mir-465	0.07	0.17	-0.05	-0.26	-0.12	0.02	0.17
4	Not Changed	S-mmu-mir-451	0.14	-0.02	-0.02	-0.02	-0.03	-0.03	-0.03
4	Not Changed	S-mmu-mir-449	0.13	0.00	-0.03	-0.05	-0.04	-0.02	0.00
4	Not Changed	S-mmu-mir-448	0.05	0.02	-0.01	-0.04	-0.02	-0.01	0.01
4	Not Changed	S-mmu-mir-431	-0.62	-0.37	-0.03	0.31	0.27	0.24	0.21
4	Not Changed	S-mmu-mir-409	-0.13	-0.11	-0.04	0.04	0.06	0.08	0.10
4	Not Changed	S-mmu-mir-384	0.20	-0.05	-0.07	-0.09	-0.04	0.00	0.04
4	Not Changed	S-mmu-mir-382	-0.23	-0.08	-0.09	-0.10	0.03	0.17	0.31
4	Not Changed	S-mmu-mir-381	0.04	0.07	0.03	-0.01	-0.03	-0.04	-0.06

Class	ES Time Pattern	ProbeID	ES-Day0	ES-Day1	ES-Day2	ES-Day3	ES-Day4	ES-Day5	ES-Day6
4	Not Changed	S-mmu-mir-379	0.24	0.39	0.26	0.13	-0.10	-0.34	-0.58
4	Not Changed	S-mmu-mir-378	0.78	0.12	-0.44	-1.01	-0.41	0.18	0.78
4	Not Changed	S-mmu-mir-377	0.25	0.07	-0.01	-0.08	-0.08	-0.08	-0.08
4	Not Changed	S-mmu-mir-370	-0.23	-0.07	0.01	0.10	0.08	0.06	0.05
4	Not Changed	S-mmu-mir-365-2	-0.40	0.39	0.39	0.39	0.07	-0.26	-0.58
4	Not Changed	S-mmu-mir-365-1	-0.27	0.25	0.22	0.19	0.03	-0.13	-0.29
4	Not Changed	S-mmu-mir-363	1.58	0.09	0.04	-0.02	-0.29	-0.57	-0.84
4	Not Changed	S-mmu-mir-361	0.45	-0.17	-0.14	-0.11	-0.06	-0.01	0.04
4	Not Changed	S-mmu-mir-34c	0.13	0.18	0.01	-0.17	-0.11	-0.05	0.01
4	Not Changed	S-mmu-mir-34b	-0.18	0.22	0.09	-0.04	-0.03	-0.02	-0.02
4	Not Changed	S-mmu-mir-34a	0.41	-0.19	-0.17	-0.15	-0.06	0.03	0.12
4	Not Changed	S-mmu-mir-346	-0.10	0.09	0.20	0.31	0.07	-0.17	-0.41
4	Not Changed	S-mmu-mir-342	-0.39	-0.06	-0.31	-0.55	-0.06	0.44	0.94
4	Not Changed	S-mmu-mir-339	-0.08	0.12	0.01	-0.10	-0.04	0.02	0.07
4	Not Changed	S-mmu-mir-338	-0.04	0.01	0.01	0.01	0.01	0.00	0.00
4	Not Changed	S-mmu-mir-337	0.47	0.03	-0.24	-0.50	-0.21	0.08	0.37
4	Not Changed	S-mmu-mir-335	-0.01	-0.02	0.01	0.04	0.02	-0.01	-0.03
4	Not Changed	S-mmu-mir-331	-0.07	0.07	0.03	0.00	-0.01	-0.01	-0.01
4	Not Changed	S-mmu-mir-330	0.06	0.19	0.01	-0.17	-0.10	-0.03	0.04
4	Not Changed	S-mmu-mir-33	0.00	0.02	-0.01	-0.03	-0.01	0.01	0.03
4	Not Changed	S-mmu-mir-329	0.29	0.36	0.04	-0.28	-0.21	-0.14	-0.07
4	Not Changed	S-mmu-mir-328	0.02	0.03	0.09	0.16	0.03	-0.10	-0.23
4	Not Changed	S-mmu-mir-326	0.14	0.11	0.23	0.35	0.04	-0.28	-0.59
4	Not Changed	S-mmu-mir-323	0.54	0.01	0.05	0.10	-0.07	-0.23	-0.40
4	Not Changed	S-mmu-mir-32	0.15	0.02	0.01	-0.01	-0.03	-0.06	-0.08
4	Not Changed	S-mmu-mir-31	0.11	0.00	-0.03	-0.05	-0.03	-0.01	0.01
4	Not Changed	S-mmu-mir-30c-2	0.37	0.10	0.03	-0.04	-0.10	-0.15	-0.20
4	Not Changed	S-mmu-mir-30c-1	0.93	0.09	0.38	0.67	-0.01	-0.69	-1.36
4	Not Changed	S-mmu-mir-30b	0.34	-0.08	-0.01	0.06	-0.02	-0.10	-0.18
4	Not Changed	S-mmu-mir-300	0.11	-0.30	-0.13	0.04	0.07	0.09	0.12
4	Not Changed	S-mmu-mir-29b-2	0.09	-0.03	-0.03	-0.03	-0.02	0.00	0.02
4	Not Changed	S-mmu-mir-29b-1	0.15	-0.07	-0.02	0.02	0.00	-0.03	-0.05
4	Not Changed	S-mmu-mir-29a	0.09	-0.04	-0.04	-0.04	-0.01	0.01	0.03
4	Not Changed	S-mmu-mir-298	0.20	-0.01	-0.05	-0.08	-0.05	-0.02	0.02
4	Not Changed	S-mmu-mir-28	-0.16	-0.07	-0.16	-0.24	-0.02	0.21	0.44
4	Not Changed	S-mmu-mir-27b	0.16	0.02	-0.03	-0.08	-0.05	-0.02	0.01
4	Not Changed	S-mmu-mir-26a-1	0.12	0.04	0.01	-0.02	-0.04	-0.05	-0.06
4	Not Changed	S-mmu-mir-23b	0.46	0.16	-0.01	-0.18	-0.16	-0.14	-0.13

Class	ES Time Pattern	ProbeID	ES-Day0	ES-Day1	ES-Day2	ES-Day3	ES-Day4	ES-Day5	ES-Day6
4	Not Changed	S-mmu-mir-221	0.13	0.03	-0.01	-0.05	-0.04	-0.04	-0.03
4	Not Changed	S-mmu-mir-22	0.14	0.19	0.06	-0.06	-0.08	-0.11	-0.14
4	Not Changed	S-mmu-mir-218-2	0.11	0.08	-0.07	-0.21	-0.09	0.03	0.16
4	Not Changed	S-mmu-mir-218-1	-0.07	0.07	-0.05	-0.17	-0.05	0.08	0.20
4	Not Changed	S-mmu-mir-214	0.01	-0.14	-0.09	-0.04	0.02	0.08	0.14
4	Not Changed	S-mmu-mir-212	-0.27	-0.19	-0.01	0.18	0.14	0.10	0.06
4	Not Changed	S-mmu-mir-211	-0.59	-0.12	0.05	0.22	0.18	0.15	0.11
4	Not Changed	S-mmu-mir-208	0.09	0.21	0.20	0.19	-0.02	-0.23	-0.45
4	Not Changed	S-mmu-mir-207	-0.18	0.05	0.13	0.20	0.07	-0.07	-0.20
4	Not Changed	S-mmu-mir-204	0.34	-0.11	0.42	0.94	0.21	-0.53	-1.26
4	Not Changed	S-mmu-mir-200b	-0.07	0.33	0.16	-0.01	-0.07	-0.14	-0.20
4	Not Changed	S-mmu-mir-200a	0.22	0.16	0.02	-0.12	-0.11	-0.10	-0.08
4	Not Changed	S-mmu-mir-20	0.06	0.06	0.01	-0.04	-0.04	-0.03	-0.03
4	Not Changed	S-mmu-mir-196b	0.32	-0.06	-0.11	-0.16	-0.08	0.01	0.09
4	Not Changed	S-mmu-mir-196a-2	0.04	-0.08	-0.04	0.00	0.01	0.03	0.04
4	Not Changed	S-mmu-mir-196a-1	0.05	0.05	0.01	-0.03	-0.03	-0.03	-0.03
4	Not Changed	S-mmu-mir-195	0.09	-0.05	-0.03	-0.01	0.00	0.00	0.01
4	Not Changed	S-mmu-mir-194-2	0.29	0.31	0.27	0.22	-0.07	-0.36	-0.66
4	Not Changed	S-mmu-mir-194-1	0.10	0.05	0.03	0.01	-0.03	-0.06	-0.09
4	Not Changed	S-mmu-mir-192	0.09	0.03	0.00	-0.02	-0.03	-0.03	-0.04
4	Not Changed	S-mmu-mir-191	0.09	0.01	-0.01	-0.02	-0.02	-0.02	-0.03
4	Not Changed	S-mmu-mir-190	-0.02	0.08	0.02	-0.04	-0.03	-0.01	0.01
4	Not Changed	S-mmu-mir-188	0.10	0.01	-0.01	-0.03	-0.03	-0.02	-0.02
4	Not Changed	S-mmu-mir-187	0.11	0.02	0.00	-0.02	-0.03	-0.04	-0.05
4	Not Changed	S-mmu-mir-186	0.02	0.04	0.04	0.03	-0.01	-0.04	-0.08
4	Not Changed	S-mmu-mir-185	0.11	0.09	0.04	-0.02	-0.05	-0.07	-0.10
4	Not Changed	S-mmu-mir-184	-0.13	0.10	0.02	-0.06	-0.02	0.03	0.07
4	Not Changed	S-mmu-mir-182	0.09	0.03	-0.02	-0.07	-0.04	-0.01	0.01
4	Not Changed	S-mmu-mir-16-2	0.18	-0.09	-0.08	-0.06	-0.02	0.02	0.06
4	Not Changed	S-mmu-mir-16-1	0.34	0.13	0.00	-0.13	-0.12	-0.11	-0.10
4	Not Changed	S-mmu-mir-15b	0.21	-0.11	-0.08	-0.04	-0.02	0.01	0.03
4	Not Changed	S-mmu-mir-15a	0.18	-0.05	-0.07	-0.09	-0.04	0.01	0.07
4	Not Changed	S-mmu-mir-154	0.16	0.47	0.23	0.00	-0.14	-0.29	-0.43
4	Not Changed	S-mmu-mir-151	0.80	0.25	-0.17	-0.59	-0.34	-0.10	0.15
4	Not Changed	S-mmu-mir-150	-0.52	-0.16	-0.02	0.12	0.16	0.19	0.23
4	Not Changed	S-mmu-mir-149	-0.30	-0.01	0.15	0.31	0.13	-0.05	-0.23
4	Not Changed	S-mmu-mir-148b	0.12	-0.06	-0.08	-0.10	-0.03	0.04	0.11
4	Not Changed	S-mmu-mir-148a	0.07	0.01	-0.03	-0.07	-0.03	0.01	0.05

Class	ES Time Pattern	ProbeID	ES-Day0	ES-Day1	ES-Day2	ES-Day3	ES-Day4	ES-Day5	ES-Day6
4	Not Changed	S-mmu-mir-146	0.13	-0.05	-0.06	-0.08	-0.03	0.02	0.07
4	Not Changed	S-mmu-mir-145	0.21	-0.15	-0.10	-0.04	-0.01	0.03	0.06
4	Not Changed	S-mmu-mir-144	0.10	0.00	-0.03	-0.06	-0.03	0.00	0.02
4	Not Changed	S-mmu-mir-143	0.18	-0.04	-0.06	-0.08	-0.04	0.00	0.04
4	Not Changed	S-mmu-mir-141	0.05	-0.08	-0.06	-0.05	0.00	0.04	0.09
4	Not Changed	S-mmu-mir-139	0.12	-0.07	-0.03	0.02	0.00	-0.01	-0.03
4	Not Changed	S-mmu-mir-138-2	0.22	-0.14	-0.10	-0.05	-0.01	0.02	0.06
4	Not Changed	S-mmu-mir-138-1	0.07	-0.09	-0.07	-0.06	0.00	0.05	0.11
4	Not Changed	S-mmu-mir-137	0.15	-0.04	-0.05	-0.05	-0.03	0.00	0.03
4	Not Changed	S-mmu-mir-135a-1	0.07	-0.19	-0.13	-0.07	0.02	0.11	0.20
4	Not Changed	S-mmu-mir-134	-0.11	-0.08	-0.03	0.02	0.04	0.07	0.10
4	Not Changed	S-mmu-mir-133b	0.89	0.47	-0.14	-0.74	-0.45	-0.16	0.13
4	Not Changed	S-mmu-mir-133a-2	0.10	-0.03	-0.05	-0.06	-0.02	0.02	0.05
4	Not Changed	S-mmu-mir-133a-1	0.07	-0.04	-0.04	-0.05	-0.01	0.02	0.06
4	Not Changed	S-mmu-mir-132	0.04	-0.02	-0.02	-0.01	-0.01	0.00	0.01
4	Not Changed	S-mmu-mir-130a	0.07	-0.01	-0.03	-0.06	-0.02	0.01	0.04
4	Not Changed	S-mmu-mir-129-1	0.19	0.05	-0.01	-0.07	-0.06	-0.05	-0.04
4	Not Changed	S-mmu-mir-128b	0.58	0.48	0.56	0.65	-0.05	-0.76	-1.46
4	Not Changed	S-mmu-mir-128a	0.08	-0.04	0.00	0.03	0.00	-0.02	-0.05
4	Not Changed	S-mmu-mir-127	-0.18	-0.16	-0.22	-0.28	0.00	0.28	0.55
4	Not Changed	S-mmu-mir-125b-2	-0.13	-0.25	-0.17	-0.08	0.06	0.21	0.36
4	Not Changed	S-mmu-mir-125a	-0.32	-0.14	-0.14	-0.15	0.05	0.25	0.45
4	Not Changed	S-mmu-mir-124a-3	0.09	-0.06	-0.06	-0.06	-0.01	0.03	0.08
4	Not Changed	S-mmu-mir-124a-2	0.02	-0.16	-0.10	-0.05	0.02	0.10	0.17
4	Not Changed	S-mmu-mir-124a-1	0.06	-0.11	-0.10	-0.08	0.00	0.08	0.16
4	Not Changed	S-mmu-mir-122a	0.05	-0.01	-0.06	-0.10	-0.03	0.04	0.11
4	Not Changed	S-mmu-mir-1-2	0.03	-0.04	-0.06	-0.08	-0.02	0.05	0.12
4	Not Changed	S-mmu-mir-1-1	-0.06	0.02	-0.02	-0.06	-0.01	0.04	0.09
4	Not Changed	S-mmu-mir-107	0.02	-0.03	-0.04	-0.05	-0.01	0.03	0.07
4	Not Changed	S-mmu-mir-106b	0.79	0.40	-0.11	-0.63	-0.39	-0.15	0.09
4	Not Changed	S-mmu-mir-106a	0.09	0.15	0.03	-0.08	-0.07	-0.06	-0.05
4	Not Changed	S-mmu-mir-103-2	0.12	0.01	-0.02	-0.05	-0.03	-0.02	0.00
4	Not Changed	S-mmu-mir-103-1	0.04	0.09	0.01	-0.06	-0.05	-0.03	-0.01
4	Not Changed	S-mmu-mir-101b	0.02	0.03	-0.02	-0.06	-0.03	0.01	0.04
4	Not Changed	S-mmu-mir-101a	0.08	0.02	-0.02	-0.06	-0.03	-0.01	0.02
4	Not Changed	S-mmu-mir-100	0.04	0.00	-0.02	-0.03	-0.02	0.00	0.02
4	Not Changed	S-mmu-let-7i	0.06	-0.04	-0.05	-0.05	-0.01	0.03	0.06
4	Not Changed	S-mmu-let-7g	-0.02	-0.13	-0.05	0.02	0.04	0.06	0.08

Class	ES Time Pattern	ProbeID	ES-Day0	ES-Day1	ES-Day2	ES-Day3	ES-Day4	ES-Day5	ES-Day6
4	Not Changed	S-mmu-let-7f-2	0.01	0.03	-0.01	-0.05	-0.02	0.01	0.03
4	Not Changed	S-mmu-let-7f-1	0.12	-0.03	-0.04	-0.05	-0.03	0.00	0.03
4	Not Changed	S-mmu-let-7c-2	0.04	0.02	-0.02	-0.05	-0.02	0.00	0.03
4	Not Changed	S-mmu-let-7c-1	0.03	-0.01	-0.02	-0.04	-0.01	0.02	0.04
4	Not Changed	S-mmu-let-7b	-0.05	-0.04	-0.03	-0.03	0.01	0.05	0.09
4	Not Changed	S-mmu-let-7a-2	-0.05	-0.06	-0.01	0.03	0.03	0.03	0.03
4	Not Changed	S-mmu-let-7a-1	-0.01	0.06	-0.02	-0.10	-0.04	0.02	0.08

Supplemental Table ST5B: ES-GCNF Time Series GCNF -/-. This table shows the data for the GCNF -/- time series. In each case the values have been normalized and interpolated. The actual experiment was carried out with RNA isolated from the GCNF -/- ES mutant from Day 0, Day 1, Day 3 and Day 6. Values for the other days (Days 2, 4 and 5) have been linearly interpolated from these.

Class	GCNF -/- Time Pattern	ProbeID	GCNF-Day0	GCNF-Day1	GCNF-Day2	GCNF-Day3	GCNF-Day4	GCNF-Day5	GCNF-Day6
1	Changed	MCE-MIR_810:rev	0.89	2.17	2.25	2.32	2.06	1.80	1.53
1	Changed	MCE-MIR_5389:fwd	0.13	-0.04	0.62	1.27	1.11	0.95	0.79
1	Changed	MCE-MIR_4740:fwd	0.29	1.22	1.35	1.48	1.62	1.76	1.90
1	Changed	MCE-MIR_4673:fwd	0.14	0.33	0.58	0.83	0.69	0.55	0.42
1	Changed	MCE-MIR_3859:fwd	0.22	0.24	0.95	1.67	1.56	1.46	1.35
1	Changed	MCE-MIR_3847:fwd	-0.04	-0.22	0.42	1.07	0.87	0.66	0.46
1	Changed	MCE-MIR_3513:rev	-0.20	0.45	0.73	1.00	1.00	1.00	1.01
1	Changed	MCE-MIR_3111:fwd	0.12	0.16	0.36	0.56	0.52	0.48	0.45
1	Changed	MCE-MIR_2310:rev	0.65	0.14	0.22	0.30	-0.57	-1.45	-2.33
1	Changed	MCE-MIR_2111:fwd	0.15	0.14	0.83	1.53	0.85	0.18	-0.49
1	Not Changed	MCE-MIR_6107:fwd	0.74	0.70	0.71	0.72	0.19	-0.35	-0.88
1	Not Changed	MCE-MIR_6026:rev	0.48	-0.23	0.18	0.58	-0.45	-1.49	-2.52
1	Not Changed	MCE-MIR_5470:fwd	1.35	0.88	1.20	1.52	0.67	-0.19	-1.04
1	Not Changed	MCE-MIR_4932:rev	0.21	0.13	0.04	-0.05	-0.07	-0.09	-0.10
1	Not Changed	MCE-MIR_4922:fwd	0.90	0.45	1.19	1.93	1.11	0.29	-0.53
1	Not Changed	MCE-MIR_4791:rev	0.81	1.43	1.13	0.83	0.51	0.18	-0.14
1	Not Changed	MCE-MIR_4493:fwd	0.19	0.13	0.05	-0.02	-0.06	-0.10	-0.14
1	Not Changed	MCE-MIR_2999:fwd	-0.05	-0.45	-0.02	0.41	-0.14	-0.70	-1.26
1	Not Changed	MCE-MIR_2337:fwd	0.30	0.11	-0.05	-0.21	-0.44	-0.68	-0.91
1	Not Changed	MCE-MIR_1784:fwd	0.20	-0.19	0.54	1.26	-0.48	-2.22	-3.97
1	Not Changed	MCE-MIR_1514:fwd	1.09	0.88	1.03	1.17	0.80	0.42	0.04

Class	GCNF -/- Time Pattern	ProbeID	GCNF-Day0	GCNF-Day1	GCNF-Day2	GCNF-Day3	GCNF-Day4	GCNF-Day5	GCNF-Day6
2	Changed	MCE-MIR_5216:rev	0.09	-0.90	-0.11	0.68	-0.06	-0.80	-1.54
2	Changed	MCE-MIR_5192:rev	0.18	0.27	0.30	0.33	0.38	0.43	0.48
2	Changed	MCE-MIR_4791:fwd	0.92	1.00	1.38	1.77	1.73	1.68	1.63
2	Changed	MCE-MIR_4674:fwd	-0.41	-0.07	0.51	1.08	1.26	1.43	1.60
2	Changed	MCE-MIR_4153:fwd	0.11	0.14	-0.10	-0.35	-0.40	-0.45	-0.51
2	Changed	MCE-MIR_3820:rev	0.05	-0.04	-0.18	-0.31	-0.33	-0.36	-0.38
2	Changed	MCE-MIR_3474:rev	0.43	1.04	0.66	0.29	-0.05	-0.39	-0.73
2	Changed	MCE-MIR_329:fwd	0.44	0.86	0.59	0.32	0.10	-0.13	-0.36
2	Changed	MCE-MIR_298:rev	0.47	0.58	0.77	0.95	1.08	1.21	1.35
2	Changed	MCE-MIR_2714:rev	0.52	1.08	0.81	0.54	-0.15	-0.83	-1.51
2	Changed	MCE-MIR_1539:rev	0.17	0.05	0.02	-0.02	-0.15	-0.28	-0.41
2	Changed	MCE-MIR_1457:rev	0.21	-0.05	-0.06	-0.07	-0.20	-0.32	-0.45
2	Changed	MCE-MIR_1371:fwd	0.26	0.75	0.49	0.24	0.48	0.73	0.98
2	Changed	MCE-MIR_1046:rev	0.32	0.06	0.35	0.63	0.29	-0.04	-0.38
2	Not Changed	MCE-MIR_783:rev	0.15	0.41	0.39	0.38	0.23	0.09	-0.05
2	Not Changed	MCE-MIR_5914:rev	-0.39	-0.23	-0.29	-0.35	-0.58	-0.82	-1.06
2	Not Changed	MCE-MIR_5745:fwd	0.02	0.32	0.67	1.01	0.13	-0.74	-1.62
2	Not Changed	MCE-MIR_5276:fwd	0.43	0.67	0.75	0.82	0.33	-0.17	-0.66
2	Not Changed	MCE-MIR_5152:rev	0.45	0.50	0.66	0.82	0.01	-0.80	-1.61
2	Not Changed	MCE-MIR_5005:rev	-0.09	0.12	0.12	0.12	-0.07	-0.25	-0.43
2	Not Changed	MCE-MIR_4627:fwd	0.62	0.46	0.91	1.35	0.71	0.07	-0.57
2	Not Changed	MCE-MIR_4610:fwd	0.30	0.01	0.27	0.52	-0.50	-1.53	-2.55
2	Not Changed	MCE-MIR_4198:fwd	0.68	0.17	0.57	0.98	0.37	-0.23	-0.84
2	Not Changed	MCE-MIR_3685:fwd	-0.40	0.30	0.97	1.64	0.99	0.35	-0.30
2	Not Changed	MCE-MIR_3492:fwd	0.11	0.16	1.01	1.85	1.06	0.26	-0.54
2	Not Changed	MCE-MIR_3143:fwd	0.45	0.36	0.48	0.60	0.59	0.58	0.57
2	Not Changed	MCE-MIR_2986:fwd	0.35	0.22	1.15	2.09	0.66	-0.76	-2.18
2	Not Changed	MCE-MIR_2815:fwd	0.38	-0.01	0.19	0.40	-0.12	-0.64	-1.15
2	Not Changed	MCE-MIR_2285:fwd	0.58	0.18	1.15	2.13	1.33	0.54	-0.25
2	Not Changed	MCE-MIR_1986:fwd	0.37	0.61	0.53	0.45	0.51	0.56	0.62
2	Not Changed	MCE-MIR_1857:fwd	0.97	1.30	1.27	1.25	0.16	-0.92	-2.01
2	Not Changed	MCE-MIR_1734:fwd	0.06	0.55	0.99	1.43	1.03	0.62	0.21
2	Not Changed	MCE-MIR_1506:rev	0.07	0.35	0.38	0.41	0.29	0.17	0.04
2	Not Changed	MCE-MIR_136:fwd	0.06	0.25	0.31	0.38	0.31	0.23	0.16
2	Not Changed	MCE-MIR_135:fwd	0.25	0.53	0.42	0.30	0.26	0.22	0.17
2	Not Changed	MCE-MIR_1074:rev	0.45	0.11	1.14	2.17	0.80	-0.57	-1.93
3	Changed	MCE-MIR_3477:rev	0.03	1.47	0.37	-0.73	0.10	0.94	1.77
3	Changed	MCE-MIR_3191:rev	-0.24	-0.40	-0.17	0.06	-0.24	-0.54	-0.85

Class	GCNF -/- Time Pattern	ProbeID	GCNF-Day0	GCNF-Day1	GCNF-Day2	GCNF-Day3	GCNF-Day4	GCNF-Day5	GCNF-Day6
3	Changed	MCE-MIR_3190:rev	-0.22	-0.39	-0.12	0.15	-0.29	-0.73	-1.17
3	Not Changed	MCE-MIR_4297:fwd	0.39	0.05	0.19	0.32	0.35	0.37	0.40
3	Not Changed	MCE-MIR_3478:rev	1.80	0.65	0.56	0.46	1.18	1.90	2.62
3	Not Changed	MCE-MIR_3470:rev	0.28	0.23	0.19	0.15	0.46	0.78	1.10
3	Not Changed	MCE-MIR_3468:rev	0.12	0.00	0.00	0.00	0.07	0.14	0.21
4	Changed	MCE-MIR_968:fwd	0.37	0.19	0.08	-0.02	-0.30	-0.58	-0.85
4	Changed	MCE-MIR_89:fwd	0.14	0.08	0.11	0.14	0.70	1.26	1.82
4	Changed	MCE-MIR_782:rev	0.05	0.06	0.13	0.20	0.21	0.22	0.23
4	Changed	MCE-MIR_774:rev	0.04	0.05	0.10	0.15	0.19	0.22	0.26
4	Changed	MCE-MIR_645:fwd	0.44	0.12	0.13	0.14	-0.17	-0.48	-0.79
4	Changed	MCE-MIR_638:fwd	0.67	0.02	-0.38	-0.79	-1.01	-1.24	-1.47
4	Changed	MCE-MIR_6054:rev	0.30	0.55	0.38	0.20	0.84	1.47	2.10
4	Changed	MCE-MIR_6034:rev	-0.07	0.15	0.66	1.16	0.84	0.52	0.20
4	Changed	MCE-MIR_5712:rev	0.04	0.59	0.56	0.52	1.00	1.48	1.95
4	Changed	MCE-MIR_5620:fwd	0.00	0.32	0.52	0.71	0.57	0.42	0.28
4	Changed	MCE-MIR_5374:rev	-0.03	-0.03	0.00	0.02	0.08	0.14	0.20
4	Changed	MCE-MIR_5322:rev	0.01	-0.10	0.19	0.48	0.30	0.13	-0.04
4	Changed	MCE-MIR_5303:fwd	0.11	0.54	0.92	1.31	2.11	2.90	3.70
4	Changed	MCE-MIR_5300:rev	-0.04	-0.01	-0.02	-0.03	0.04	0.12	0.20
4	Changed	MCE-MIR_5062:rev	0.09	-0.01	-0.24	-0.47	-0.28	-0.09	0.11
4	Changed	MCE-MIR_5060:rev	-0.02	-0.24	-0.30	-0.35	-0.34	-0.33	-0.32
4	Changed	MCE-MIR_4861:fwd	0.08	-0.26	-0.09	0.08	-0.29	-0.66	-1.04
4	Changed	MCE-MIR_4716:fwd	0.31	0.60	0.61	0.63	0.27	-0.10	-0.47
4	Changed	MCE-MIR_4497:rev	0.09	-0.17	0.91	1.99	1.23	0.47	-0.29
4	Changed	MCE-MIR_4491:rev	-0.06	1.28	1.63	1.97	1.37	0.77	0.17
4	Changed	MCE-MIR_4179:rev	-0.04	0.61	0.41	0.20	1.13	2.06	2.99
4	Changed	MCE-MIR_3503:rev	0.28	-0.02	-0.50	-0.97	-0.65	-0.33	-0.02
4	Changed	MCE-MIR_3429:fwd	-0.10	-0.13	0.78	1.69	1.10	0.51	-0.09
4	Changed	MCE-MIR_3416:rev	0.54	0.29	-0.03	-0.35	-0.06	0.24	0.53
4	Changed	MCE-MIR_3379:fwd	-0.06	0.06	0.34	0.62	0.43	0.23	0.03
4	Changed	MCE-MIR_334:rev	-0.05	0.30	0.82	1.34	1.00	0.65	0.30
4	Changed	MCE-MIR_3226:fwd	0.37	0.25	0.73	1.22	0.99	0.76	0.53
4	Changed	MCE-MIR_3048:rev	0.01	0.06	0.31	0.55	0.38	0.22	0.05
4	Changed	MCE-MIR_293:fwd	0.30	0.42	0.53	0.64	0.86	1.08	1.30
4	Changed	MCE-MIR_2902:rev	1.29	1.32	2.06	2.80	1.98	1.15	0.32
4	Changed	MCE-MIR_2902:fwd	-0.14	-0.05	0.59	1.23	0.94	0.65	0.36
4	Changed	MCE-MIR_2563:fwd	-0.46	0.76	1.45	2.14	2.18	2.21	2.24
4	Changed	MCE-MIR_2524:fwd	0.17	1.03	0.79	0.55	1.43	2.31	3.19

Class	GCNF -/- Time Pattern	ProbeID	GCNF-Day0	GCNF-Day1	GCNF-Day2	GCNF-Day3	GCNF-Day4	GCNF-Day5	GCNF-Day6
4	Changed	MCE-MIR_2349:rev	0.46	0.44	1.04	1.64	0.99	0.34	-0.31
4	Changed	MCE-MIR_2169:rev	0.53	-0.26	-0.10	0.05	-0.26	-0.58	-0.89
4	Changed	MCE-MIR_1931:fwd	0.26	0.40	0.74	1.07	1.25	1.42	1.59
4	Changed	MCE-MIR_18:fwd	0.96	0.28	0.45	0.61	0.28	-0.06	-0.39
4	Changed	MCE-MIR_1788:rev	0.61	0.39	1.83	3.26	1.93	0.60	-0.73
4	Changed	MCE-MIR_1788:fwd	0.06	-0.14	0.24	0.61	0.38	0.16	-0.07
4	Changed	MCE-MIR_1773:fwd	-0.01	-0.14	-0.14	-0.13	-0.11	-0.08	-0.06
4	Changed	MCE-MIR_1746:rev	0.15	0.16	1.06	1.97	0.94	-0.09	-1.12
4	Changed	MCE-MIR_1504:rev	0.11	-0.03	-0.17	-0.32	-0.35	-0.38	-0.41
4	Changed	MCE-MIR_1394:rev	-0.13	-0.80	-0.73	-0.66	-0.94	-1.22	-1.50
4	Changed	MCE-MIR_1226:fwd	0.13	0.13	0.45	0.77	0.46	0.16	-0.14
4	Not Changed	MCE-MIR_995:rev	0.11	0.14	0.06	-0.03	0.07	0.17	0.27
4	Not Changed	MCE-MIR_993:rev	0.58	0.02	-0.09	-0.19	0.55	1.30	2.05
4	Not Changed	MCE-MIR_988:fwd	0.37	-0.09	-0.21	-0.34	0.40	1.13	1.87
4	Not Changed	MCE-MIR_984:rev	0.03	0.09	0.02	-0.05	0.06	0.16	0.27
4	Not Changed	MCE-MIR_959:rev	0.52	0.23	0.07	-0.10	-0.12	-0.15	-0.17
4	Not Changed	MCE-MIR_946:rev	0.03	0.06	0.15	0.24	0.21	0.17	0.14
4	Not Changed	MCE-MIR_942:rev	0.12	-0.05	0.54	1.13	0.87	0.60	0.34
4	Not Changed	MCE-MIR_936:rev	0.43	-0.05	-0.17	-0.30	-0.22	-0.14	-0.06
4	Not Changed	MCE-MIR_871:rev	-0.07	-0.06	0.00	0.06	0.11	0.15	0.20
4	Not Changed	MCE-MIR_871:fwd	0.08	-0.04	-0.10	-0.16	-0.04	0.09	0.21
4	Not Changed	MCE-MIR_855:rev	-0.07	-0.01	0.01	0.04	0.12	0.19	0.27
4	Not Changed	MCE-MIR_855:fwd	0.02	-0.04	0.15	0.35	0.25	0.16	0.06
4	Not Changed	MCE-MIR_822:fwd	0.34	0.35	0.40	0.45	0.08	-0.29	-0.66
4	Not Changed	MCE-MIR_81:fwd	0.03	-0.05	-0.03	-0.01	0.06	0.14	0.22
4	Not Changed	MCE-MIR_809:fwd	0.04	0.09	0.12	0.15	0.14	0.14	0.14
4	Not Changed	MCE-MIR_780:rev	-0.06	-0.31	0.47	1.26	0.65	0.05	-0.55
4	Not Changed	MCE-MIR_777:rev	0.37	-0.44	1.23	2.90	1.72	0.54	-0.64
4	Not Changed	MCE-MIR_755:fwd	0.50	0.43	0.11	-0.22	0.52	1.26	1.99
4	Not Changed	MCE-MIR_734:fwd	0.40	0.39	0.34	0.28	0.43	0.58	0.73
4	Not Changed	MCE-MIR_725:fwd	0.37	0.11	-0.07	-0.25	0.45	1.16	1.86
4	Not Changed	MCE-MIR_689:rev	-0.01	0.00	-0.02	-0.04	0.01	0.07	0.12
4	Not Changed	MCE-MIR_670:fwd	0.58	-0.13	0.58	1.30	0.05	-1.19	-2.44
4	Not Changed	MCE-MIR_6120:fwd	-0.06	0.22	0.14	0.06	0.17	0.29	0.40
4	Not Changed	MCE-MIR_6084:rev	0.77	0.61	0.09	-0.44	-0.19	0.06	0.31
4	Not Changed	MCE-MIR_6055:rev	0.53	0.34	0.80	1.27	0.94	0.61	0.29
4	Not Changed	MCE-MIR_6050:rev	0.92	-0.32	0.29	0.90	0.68	0.46	0.24
4	Not Changed	MCE-MIR_6033:fwd	-0.03	-0.05	0.04	0.14	0.11	0.09	0.07

Class	GCNF -/- Time Pattern	ProbeID	GCNF-Day0	GCNF-Day1	GCNF-Day2	GCNF-Day3	GCNF-Day4	GCNF-Day5	GCNF-Day6
4	Not Changed	MCE-MIR_6026:fwd	0.14	1.33	1.61	1.89	1.97	2.05	2.13
4	Not Changed	MCE-MIR_6001:rev	0.04	0.22	0.18	0.14	0.10	0.07	0.04
4	Not Changed	MCE-MIR_5970:rev	0.10	0.93	0.60	0.27	0.42	0.56	0.71
4	Not Changed	MCE-MIR_5872:fwd	0.44	0.26	0.34	0.41	0.37	0.33	0.29
4	Not Changed	MCE-MIR_5864:fwd	-0.05	0.07	0.02	-0.03	0.05	0.13	0.22
4	Not Changed	MCE-MIR_5790:fwd	-0.25	-0.39	0.07	0.53	0.07	-0.40	-0.87
4	Not Changed	MCE-MIR_5736:rev	0.35	0.53	0.84	1.16	1.09	1.02	0.95
4	Not Changed	MCE-MIR_5704:rev	-0.02	-0.11	-0.10	-0.09	0.10	0.29	0.49
4	Not Changed	MCE-MIR_5699:fwd	0.32	-0.24	-0.07	0.10	-0.54	-1.18	-1.82
4	Not Changed	MCE-MIR_5643:fwd	1.19	-0.12	0.80	1.72	0.53	-0.66	-1.85
4	Not Changed	MCE-MIR_5641:rev	1.77	1.70	2.31	2.93	2.56	2.20	1.84
4	Not Changed	MCE-MIR_5623:rev	0.03	-0.06	-0.02	0.02	0.08	0.13	0.19
4	Not Changed	MCE-MIR_5607:rev	1.53	0.63	0.35	0.06	-0.65	-1.35	-2.06
4	Not Changed	MCE-MIR_5606:fwd	0.28	0.30	0.16	0.03	-0.03	-0.09	-0.14
4	Not Changed	MCE-MIR_5598:fwd	0.06	0.00	0.02	0.05	0.08	0.11	0.14
4	Not Changed	MCE-MIR_5597:fwd	0.33	0.14	0.09	0.04	0.10	0.17	0.23
4	Not Changed	MCE-MIR_5596:rev	0.94	0.60	0.68	0.76	0.35	-0.05	-0.46
4	Not Changed	MCE-MIR_5581:rev	0.03	-0.09	-0.08	-0.08	0.04	0.16	0.28
4	Not Changed	MCE-MIR_5581:fwd	0.01	-0.03	-0.01	0.00	0.11	0.22	0.33
4	Not Changed	MCE-MIR_557:fwd	0.31	-0.16	0.07	0.30	0.56	0.82	1.07
4	Not Changed	MCE-MIR_5544:rev	0.41	-0.66	0.23	1.12	0.01	-1.09	-2.20
4	Not Changed	MCE-MIR_5511:rev	1.17	-0.24	1.01	2.26	1.00	-0.26	-1.51
4	Not Changed	MCE-MIR_5504:rev	0.00	-0.02	-0.01	-0.01	0.02	0.04	0.06
4	Not Changed	MCE-MIR_5503:rev	0.57	-0.57	1.12	2.81	1.39	-0.03	-1.45
4	Not Changed	MCE-MIR_5488:rev	0.44	0.01	0.18	0.36	0.37	0.38	0.39
4	Not Changed	MCE-MIR_5473:rev	0.50	0.21	0.38	0.56	0.74	0.91	1.08
4	Not Changed	MCE-MIR_5473:fwd	0.72	0.35	1.33	2.31	1.32	0.34	-0.65
4	Not Changed	MCE-MIR_5454:rev	0.26	-0.32	-0.06	0.20	0.48	0.75	1.02
4	Not Changed	MCE-MIR_5443:fwd	0.60	0.25	0.21	0.16	0.39	0.62	0.85
4	Not Changed	MCE-MIR_5440:fwd	0.45	0.25	0.69	1.13	0.36	-0.40	-1.17
4	Not Changed	MCE-MIR_543:fwd	0.54	0.08	0.64	1.20	1.10	1.00	0.90
4	Not Changed	MCE-MIR_5418:fwd	-0.41	-0.08	1.55	3.18	1.76	0.35	-1.07
4	Not Changed	MCE-MIR_5411:rev	0.45	-1.06	0.86	2.77	1.25	-0.26	-1.78
4	Not Changed	MCE-MIR_5406:rev	0.51	0.34	0.07	-0.19	0.02	0.23	0.45
4	Not Changed	MCE-MIR_5403:fwd	0.53	0.35	0.60	0.84	0.21	-0.42	-1.05
4	Not Changed	MCE-MIR_5399:fwd	0.74	0.44	0.29	0.13	0.56	0.99	1.43
4	Not Changed	MCE-MIR_5396:rev	0.09	0.50	0.26	0.01	0.34	0.66	0.99
4	Not Changed	MCE-MIR_5389:rev	0.54	0.26	0.17	0.09	0.33	0.57	0.82

Class	GCNF -/- Time Pattern	ProbeID	GCNF-Day0	GCNF-Day1	GCNF-Day2	GCNF-Day3	GCNF-Day4	GCNF-Day5	GCNF-Day6
4	Not Changed	MCE-MIR_5384:rev	0.73	0.79	1.06	1.33	0.27	-0.79	-1.86
4	Not Changed	MCE-MIR_5374:fwd	0.82	0.55	0.24	-0.07	-0.26	-0.46	-0.66
4	Not Changed	MCE-MIR_5369:rev	0.55	-0.04	-0.19	-0.35	-0.37	-0.39	-0.42
4	Not Changed	MCE-MIR_5367:rev	0.20	0.24	-0.06	-0.36	-0.12	0.13	0.37
4	Not Changed	MCE-MIR_5366:rev	0.92	0.26	0.34	0.41	1.10	1.78	2.46
4	Not Changed	MCE-MIR_5363:rev	0.59	0.26	-0.41	-1.07	-0.03	1.02	2.07
4	Not Changed	MCE-MIR_5363:fwd	1.20	1.02	0.89	0.75	-0.05	-0.85	-1.65
4	Not Changed	MCE-MIR_5354:rev	0.79	-0.42	-0.38	-0.34	-0.91	-1.49	-2.07
4	Not Changed	MCE-MIR_5340:fwd	-0.07	-0.05	1.13	2.31	1.43	0.56	-0.32
4	Not Changed	MCE-MIR_534:fwd	0.77	-0.02	1.11	2.24	0.65	-0.93	-2.52
4	Not Changed	MCE-MIR_5339:rev	0.45	0.51	0.05	-0.41	-0.23	-0.06	0.11
4	Not Changed	MCE-MIR_5328:rev	-0.45	-0.40	1.44	3.28	1.71	0.14	-1.43
4	Not Changed	MCE-MIR_5322:fwd	0.22	-0.19	-0.04	0.10	0.35	0.59	0.84
4	Not Changed	MCE-MIR_5300:fwd	0.21	-0.24	-0.12	0.01	0.09	0.17	0.25
4	Not Changed	MCE-MIR_530:fwd	0.64	0.25	0.92	1.58	0.82	0.06	-0.71
4	Not Changed	MCE-MIR_5295:rev	0.31	-0.20	-0.02	0.17	0.41	0.66	0.91
4	Not Changed	MCE-MIR_5291:fwd	0.50	-0.02	0.71	1.44	0.18	-1.08	-2.33
4	Not Changed	MCE-MIR_5287:rev	-0.10	-0.05	-0.03	-0.02	0.12	0.25	0.38
4	Not Changed	MCE-MIR_5279:fwd	-0.04	0.05	0.03	0.02	0.04	0.07	0.09
4	Not Changed	MCE-MIR_5260:fwd	-0.04	0.02	-0.03	-0.08	0.08	0.24	0.40
4	Not Changed	MCE-MIR_5236:rev	0.02	0.08	0.00	-0.08	-0.04	0.01	0.05
4	Not Changed	MCE-MIR_5210:fwd	0.36	-0.10	-0.09	-0.07	0.07	0.22	0.37
4	Not Changed	MCE-MIR_5197:rev	0.53	0.76	0.27	-0.22	0.17	0.56	0.95
4	Not Changed	MCE-MIR_5195:rev	0.06	0.02	0.05	0.08	0.13	0.19	0.24
4	Not Changed	MCE-MIR_5193:rev	0.35	0.31	0.18	0.05	0.10	0.14	0.18
4	Not Changed	MCE-MIR_5180:rev	0.05	0.09	0.04	-0.01	0.11	0.23	0.34
4	Not Changed	MCE-MIR_5180:fwd	-0.04	0.04	0.04	0.03	0.02	0.01	-0.01
4	Not Changed	MCE-MIR_5172:fwd	0.20	0.41	-0.46	-1.34	-0.50	0.35	1.19
4	Not Changed	MCE-MIR_5167:rev	0.09	0.06	0.14	0.22	0.12	0.02	-0.08
4	Not Changed	MCE-MIR_5167:fwd	-0.06	0.03	0.02	0.01	0.02	0.03	0.04
4	Not Changed	MCE-MIR_5152:fwd	0.37	0.12	0.54	0.96	0.90	0.85	0.80
4	Not Changed	MCE-MIR_5143:rev	0.36	0.18	-0.04	-0.26	-0.18	-0.10	-0.02
4	Not Changed	MCE-MIR_5141:rev	0.42	-0.27	0.10	0.48	-0.12	-0.72	-1.32
4	Not Changed	MCE-MIR_5135:fwd	0.09	0.28	0.45	0.61	0.44	0.26	0.08
4	Not Changed	MCE-MIR_5122:rev	-0.03	0.02	0.13	0.25	0.21	0.18	0.14
4	Not Changed	MCE-MIR_5109:rev	0.17	0.38	0.16	-0.06	0.28	0.62	0.96
4	Not Changed	MCE-MIR_5105:rev	-0.09	0.01	0.23	0.45	0.38	0.30	0.23
4	Not Changed	MCE-MIR_5100:rev	0.16	0.17	0.54	0.91	0.97	1.03	1.09

Class	GCNF -/- Time Pattern	ProbeID	GCNF-Day0	GCNF-Day1	GCNF-Day2	GCNF-Day3	GCNF-Day4	GCNF-Day5	GCNF-Day6
4	Not Changed	MCE-MIR_5089:fwd	-0.02	-0.16	0.09	0.34	0.01	-0.32	-0.66
4	Not Changed	MCE-MIR_5088:rev	1.28	-0.15	0.09	0.32	-0.08	-0.48	-0.88
4	Not Changed	MCE-MIR_5083:rev	0.84	0.53	0.90	1.26	1.01	0.76	0.51
4	Not Changed	MCE-MIR_5079:rev	0.42	0.44	0.17	-0.11	0.51	1.13	1.75
4	Not Changed	MCE-MIR_5068:rev	0.70	0.51	0.03	-0.45	0.76	1.97	3.17
4	Not Changed	MCE-MIR_5061:rev	-0.07	-0.16	-0.37	-0.58	-0.24	0.11	0.46
4	Not Changed	MCE-MIR_5057:fwd	0.81	0.06	0.49	0.92	0.00	-0.93	-1.86
4	Not Changed	MCE-MIR_5056:rev	0.51	0.50	0.19	-0.12	0.50	1.13	1.75
4	Not Changed	MCE-MIR_5055:rev	0.36	1.01	0.40	-0.21	0.36	0.92	1.48
4	Not Changed	MCE-MIR_5055:fwd	1.03	0.02	0.14	0.26	-0.02	-0.30	-0.58
4	Not Changed	MCE-MIR_5046:rev	-0.03	0.00	-0.02	-0.04	0.02	0.09	0.15
4	Not Changed	MCE-MIR_504:fwd	0.05	0.03	0.02	0.01	0.02	0.03	0.05
4	Not Changed	MCE-MIR_5033:rev	0.08	0.22	0.09	-0.04	-0.02	-0.01	0.00
4	Not Changed	MCE-MIR_5030:rev	0.58	-0.34	0.29	0.92	0.03	-0.87	-1.76
4	Not Changed	MCE-MIR_5014:fwd	0.18	0.54	0.47	0.40	0.91	1.42	1.94
4	Not Changed	MCE-MIR_5008:rev	0.03	-0.02	-0.03	-0.04	-0.04	-0.03	-0.02
4	Not Changed	MCE-MIR_5004:rev	0.28	0.26	-0.06	-0.38	0.12	0.63	1.14
4	Not Changed	MCE-MIR_4999:rev	0.42	-0.11	-0.61	-1.10	-0.20	0.70	1.60
4	Not Changed	MCE-MIR_4978:rev	-0.01	0.06	0.03	0.00	0.01	0.03	0.04
4	Not Changed	MCE-MIR_4972:fwd	0.02	0.05	0.06	0.06	0.06	0.06	0.06
4	Not Changed	MCE-MIR_4945:fwd	0.50	0.37	-0.17	-0.70	-0.07	0.56	1.19
4	Not Changed	MCE-MIR_4922:rev	0.33	-0.30	0.48	1.25	0.39	-0.47	-1.33
4	Not Changed	MCE-MIR_4913:rev	0.89	-0.11	0.84	1.78	0.22	-1.33	-2.89
4	Not Changed	MCE-MIR_4893:rev	-0.08	-0.01	0.04	0.10	0.08	0.06	0.04
4	Not Changed	MCE-MIR_4893:fwd	0.08	0.08	0.02	-0.04	-0.01	0.01	0.04
4	Not Changed	MCE-MIR_4854:rev	0.36	-0.73	-0.40	-0.07	-1.49	-2.90	-4.31
4	Not Changed	MCE-MIR_4853:fwd	-0.07	-0.09	0.21	0.51	0.31	0.11	-0.09
4	Not Changed	MCE-MIR_4832:rev	-0.08	0.02	0.20	0.37	0.25	0.13	0.01
4	Not Changed	MCE-MIR_4830:fwd	-0.01	0.00	0.11	0.22	0.18	0.13	0.08
4	Not Changed	MCE-MIR_4822:rev	0.23	-0.22	0.21	0.64	0.23	-0.18	-0.59
4	Not Changed	MCE-MIR_4821:rev	0.39	0.35	0.08	-0.19	-0.12	-0.05	0.02
4	Not Changed	MCE-MIR_4820:rev	0.34	0.34	0.13	-0.08	-0.01	0.06	0.13
4	Not Changed	MCE-MIR_482:rev	-0.01	0.06	0.04	0.01	0.09	0.17	0.25
4	Not Changed	MCE-MIR_482:fwd	0.00	0.03	0.00	-0.03	0.00	0.03	0.06
4	Not Changed	MCE-MIR_4809:fwd	0.09	0.23	0.12	0.01	0.03	0.05	0.06
4	Not Changed	MCE-MIR_4799:rev	-0.62	-0.38	-0.43	-0.48	-0.44	-0.41	-0.37
4	Not Changed	MCE-MIR_4789:rev	-0.05	0.13	0.04	-0.04	-0.01	0.03	0.06
4	Not Changed	MCE-MIR_4763:fwd	0.18	-0.27	-0.03	0.21	0.35	0.49	0.64

Class	GCNF -/- Time Pattern	ProbeID	GCNF-Day0	GCNF-Day1	GCNF-Day2	GCNF-Day3	GCNF-Day4	GCNF-Day5	GCNF-Day6
4	Not Changed	MCE-MIR_4762:fwd	0.04	-0.15	-0.07	0.00	0.05	0.09	0.14
4	Not Changed	MCE-MIR_4756:rev	0.26	-0.19	-0.07	0.05	0.32	0.60	0.88
4	Not Changed	MCE-MIR_4755:fwd	0.86	0.50	0.84	1.18	0.41	-0.36	-1.13
4	Not Changed	MCE-MIR_4752:rev	0.32	0.04	0.02	0.00	0.43	0.86	1.29
4	Not Changed	MCE-MIR_4748:rev	0.54	0.37	0.29	0.22	0.49	0.76	1.04
4	Not Changed	MCE-MIR_4745:fwd	0.33	0.04	0.05	0.07	0.06	0.06	0.06
4	Not Changed	MCE-MIR_4743:fwd	0.61	-0.40	0.00	0.39	-0.18	-0.76	-1.33
4	Not Changed	MCE-MIR_4738:fwd	0.06	-0.03	0.12	0.28	0.27	0.26	0.25
4	Not Changed	MCE-MIR_4726:rev	0.14	-0.08	0.25	0.58	0.60	0.63	0.65
4	Not Changed	MCE-MIR_4726:fwd	0.31	0.33	2.34	4.35	2.99	1.63	0.27
4	Not Changed	MCE-MIR_4714:rev	0.83	-0.51	0.09	0.69	-0.24	-1.16	-2.09
4	Not Changed	MCE-MIR_4714:fwd	0.37	0.02	0.08	0.14	0.27	0.40	0.52
4	Not Changed	MCE-MIR_4712:rev	0.05	-0.01	0.06	0.14	0.15	0.16	0.17
4	Not Changed	MCE-MIR_4712:fwd	0.26	-0.23	-0.10	0.02	0.28	0.53	0.79
4	Not Changed	MCE-MIR_4711:fwd	0.25	0.25	0.26	0.28	0.21	0.15	0.08
4	Not Changed	MCE-MIR_469:fwd	0.77	0.64	0.96	1.28	0.96	0.65	0.33
4	Not Changed	MCE-MIR_4684:fwd	0.60	0.47	0.31	0.15	0.36	0.58	0.79
4	Not Changed	MCE-MIR_4675:fwd	0.14	0.10	0.26	0.42	0.15	-0.12	-0.39
4	Not Changed	MCE-MIR_4667:fwd	-0.07	0.01	-0.01	-0.03	-0.02	-0.02	-0.01
4	Not Changed	MCE-MIR_4661:rev	1.07	-0.29	0.74	1.76	0.42	-0.92	-2.26
4	Not Changed	MCE-MIR_4661:fwd	0.23	-0.16	0.12	0.41	-0.03	-0.48	-0.92
4	Not Changed	MCE-MIR_466:fwd	0.00	0.27	0.21	0.15	0.14	0.12	0.11
4	Not Changed	MCE-MIR_463:fwd	0.10	-0.17	-0.06	0.05	0.07	0.08	0.10
4	Not Changed	MCE-MIR_4625:fwd	0.22	0.21	0.28	0.35	0.21	0.07	-0.08
4	Not Changed	MCE-MIR_4614:rev	-0.03	-0.03	0.02	0.08	0.14	0.19	0.24
4	Not Changed	MCE-MIR_4607:fwd	-0.01	0.01	0.02	0.04	0.07	0.11	0.15
4	Not Changed	MCE-MIR_4592:fwd	0.06	0.02	0.05	0.07	0.08	0.09	0.10
4	Not Changed	MCE-MIR_4554:rev	0.03	-0.13	0.09	0.31	0.20	0.09	-0.01
4	Not Changed	MCE-MIR_4521:rev	0.13	0.09	0.04	0.00	0.02	0.04	0.06
4	Not Changed	MCE-MIR_4521:fwd	0.02	0.00	0.02	0.04	0.06	0.07	0.08
4	Not Changed	MCE-MIR_4513:fwd	0.52	0.74	1.04	1.35	1.33	1.30	1.28
4	Not Changed	MCE-MIR_451:rev	1.30	0.36	-0.08	-0.53	-0.75	-0.97	-1.19
4	Not Changed	MCE-MIR_4503:rev	0.06	-0.01	-0.01	-0.01	0.05	0.12	0.18
4	Not Changed	MCE-MIR_4491:fwd	0.04	0.04	0.04	0.04	0.04	0.03	0.03
4	Not Changed	MCE-MIR_4474:fwd	-0.02	-0.01	-0.04	-0.06	-0.03	0.01	0.04
4	Not Changed	MCE-MIR_4472:fwd	-0.01	0.11	0.01	-0.10	-0.03	0.04	0.10
4	Not Changed	MCE-MIR_4462:fwd	0.10	0.54	0.31	0.07	0.42	0.76	1.11
4	Not Changed	MCE-MIR_4453:fwd	-0.15	-0.31	-0.14	0.04	-0.13	-0.30	-0.47

Class	GCNF -/- Time Pattern	ProbeID	GCNF-Day0	GCNF-Day1	GCNF-Day2	GCNF-Day3	GCNF-Day4	GCNF-Day5	GCNF-Day6
4	Not Changed	MCE-MIR_4449:rev	0.06	0.06	-0.01	-0.08	-0.02	0.03	0.08
4	Not Changed	MCE-MIR_4442:fwd	0.01	0.09	0.13	0.16	0.09	0.02	-0.06
4	Not Changed	MCE-MIR_4413:fwd	0.00	0.04	-0.03	-0.10	0.06	0.22	0.38
4	Not Changed	MCE-MIR_4383:rev	0.15	0.31	0.35	0.39	0.39	0.38	0.38
4	Not Changed	MCE-MIR_4353:rev	0.04	0.13	0.08	0.03	0.10	0.16	0.23
4	Not Changed	MCE-MIR_4345:rev	0.68	-0.34	0.43	1.19	0.44	-0.31	-1.06
4	Not Changed	MCE-MIR_4342:fwd	0.00	0.02	-0.03	-0.08	-0.01	0.06	0.12
4	Not Changed	MCE-MIR_4320:rev	0.73	0.64	1.31	1.99	0.40	-1.18	-2.77
4	Not Changed	MCE-MIR_4303:fwd	-0.01	0.10	0.06	0.02	0.26	0.50	0.74
4	Not Changed	MCE-MIR_4280:rev	-0.05	-0.02	0.01	0.04	0.05	0.06	0.07
4	Not Changed	MCE-MIR_4280:fwd	-0.01	0.09	0.06	0.04	0.29	0.53	0.78
4	Not Changed	MCE-MIR_4274:fwd	-0.02	0.46	0.50	0.54	0.60	0.67	0.73
4	Not Changed	MCE-MIR_4273:fwd	0.29	-0.28	-0.07	0.14	0.29	0.44	0.60
4	Not Changed	MCE-MIR_4239:rev	0.12	0.09	0.36	0.63	0.32	0.01	-0.29
4	Not Changed	MCE-MIR_4236:rev	0.05	-0.06	0.06	0.17	0.06	-0.06	-0.18
4	Not Changed	MCE-MIR_4232:fwd	0.04	0.06	0.03	0.01	0.01	0.00	0.00
4	Not Changed	MCE-MIR_4226:fwd	-0.31	0.08	0.50	0.92	0.68	0.45	0.22
4	Not Changed	MCE-MIR_4209:fwd	0.01	0.08	0.04	0.00	0.02	0.03	0.05
4	Not Changed	MCE-MIR_4207:fwd	-0.01	0.07	0.02	-0.03	0.02	0.07	0.12
4	Not Changed	MCE-MIR_4202:rev	0.38	0.22	0.56	0.91	0.73	0.55	0.37
4	Not Changed	MCE-MIR_4182:rev	0.02	0.19	0.11	0.04	0.07	0.10	0.14
4	Not Changed	MCE-MIR_4179:fwd	0.02	0.05	0.02	-0.02	-0.01	0.01	0.02
4	Not Changed	MCE-MIR_4124:fwd	0.01	0.05	0.03	0.01	0.08	0.15	0.22
4	Not Changed	MCE-MIR_4087:rev	0.13	0.01	0.26	0.52	0.35	0.18	0.02
4	Not Changed	MCE-MIR_4087:fwd	-0.10	0.11	-0.19	-0.50	-0.31	-0.13	0.06
4	Not Changed	MCE-MIR_407:fwd	0.05	0.00	-0.06	-0.11	-0.06	-0.01	0.04
4	Not Changed	MCE-MIR_4069:rev	0.06	0.38	0.57	0.76	0.86	0.95	1.04
4	Not Changed	MCE-MIR_4063:rev	0.08	0.47	0.26	0.05	0.44	0.83	1.23
4	Not Changed	MCE-MIR_4063:fwd	0.00	0.06	0.02	-0.02	0.06	0.14	0.22
4	Not Changed	MCE-MIR_4061:rev	0.11	0.20	0.11	0.02	0.22	0.41	0.60
4	Not Changed	MCE-MIR_4060:rev	0.02	0.10	0.07	0.05	0.15	0.25	0.35
4	Not Changed	MCE-MIR_406:rev	1.33	0.06	0.81	1.55	0.59	-0.36	-1.32
4	Not Changed	MCE-MIR_405:rev	0.00	-0.05	0.00	0.04	0.05	0.06	0.07
4	Not Changed	MCE-MIR_4034:rev	0.02	0.03	0.12	0.22	0.21	0.21	0.20
4	Not Changed	MCE-MIR_4031:fwd	0.82	0.64	0.81	0.99	0.83	0.68	0.52
4	Not Changed	MCE-MIR_4030:rev	0.31	0.08	1.14	2.21	0.88	-0.45	-1.77
4	Not Changed	MCE-MIR_4027:rev	0.57	0.83	0.59	0.34	1.08	1.81	2.55
4	Not Changed	MCE-MIR_4022:fwd	0.07	0.09	0.13	0.16	0.17	0.18	0.19

Class	GCNF -/- Time Pattern	ProbeID	GCNF-Day0	GCNF-Day1	GCNF-Day2	GCNF-Day3	GCNF-Day4	GCNF-Day5	GCNF-Day6
4	Not Changed	MCE-MIR_4015:fwd	0.03	0.06	-0.08	-0.21	0.08	0.38	0.68
4	Not Changed	MCE-MIR_4010:rev	0.70	0.08	0.33	0.57	0.22	-0.13	-0.48
4	Not Changed	MCE-MIR_399:fwd	0.36	0.30	0.21	0.12	0.04	-0.05	-0.13
4	Not Changed	MCE-MIR_3958:fwd	0.59	0.29	-0.05	-0.38	0.87	2.13	3.39
4	Not Changed	MCE-MIR_3888:rev	0.09	0.05	0.11	0.16	0.17	0.18	0.19
4	Not Changed	MCE-MIR_3886:rev	0.11	0.14	0.10	0.06	0.03	0.01	-0.02
4	Not Changed	MCE-MIR_3886:fwd	0.96	0.87	0.22	-0.43	-0.10	0.22	0.55
4	Not Changed	MCE-MIR_3867:rev	-0.02	0.03	-0.02	-0.07	0.02	0.11	0.21
4	Not Changed	MCE-MIR_3859:rev	0.02	0.14	0.13	0.11	0.13	0.15	0.17
4	Not Changed	MCE-MIR_3847:rev	0.89	0.82	0.29	-0.25	0.02	0.28	0.55
4	Not Changed	MCE-MIR_3841:fwd	0.05	0.20	0.20	0.21	0.21	0.22	0.23
4	Not Changed	MCE-MIR_3837:rev	0.29	-0.11	0.31	0.73	0.00	-0.72	-1.44
4	Not Changed	MCE-MIR_3832:rev	-0.01	0.03	0.02	0.01	0.04	0.07	0.10
4	Not Changed	MCE-MIR_3820:fwd	0.18	0.14	0.00	-0.13	0.27	0.67	1.07
4	Not Changed	MCE-MIR_3797:fwd	0.16	0.29	0.87	1.46	1.36	1.27	1.18
4	Not Changed	MCE-MIR_3793:rev	-0.09	0.04	-0.03	-0.10	0.08	0.26	0.44
4	Not Changed	MCE-MIR_3791:rev	0.29	-0.23	0.23	0.68	0.26	-0.16	-0.58
4	Not Changed	MCE-MIR_3782:rev	-0.02	0.06	-0.10	-0.27	-0.02	0.23	0.48
4	Not Changed	MCE-MIR_3780:rev	-0.11	0.78	1.17	1.57	1.70	1.83	1.96
4	Not Changed	MCE-MIR_3762:rev	0.63	0.39	0.00	-0.38	-0.10	0.19	0.47
4	Not Changed	MCE-MIR_3754:rev	-0.02	0.03	0.07	0.12	0.06	0.01	-0.05
4	Not Changed	MCE-MIR_3751:fwd	0.24	-0.07	-0.08	-0.09	-0.10	-0.11	-0.12
4	Not Changed	MCE-MIR_3715:rev	-0.03	0.01	0.02	0.02	0.00	-0.02	-0.04
4	Not Changed	MCE-MIR_3695:fwd	0.10	0.34	0.15	-0.04	-0.02	0.00	0.02
4	Not Changed	MCE-MIR_3686:fwd	0.24	0.11	-0.30	-0.72	-0.31	0.11	0.52
4	Not Changed	MCE-MIR_3685:rev	0.64	0.61	0.51	0.41	0.74	1.07	1.40
4	Not Changed	MCE-MIR_3684:rev	-0.01	0.22	0.29	0.36	0.33	0.30	0.27
4	Not Changed	MCE-MIR_3667:rev	-0.02	0.11	0.23	0.34	0.29	0.25	0.20
4	Not Changed	MCE-MIR_3667:fwd	0.25	0.24	0.43	0.63	0.44	0.25	0.07
4	Not Changed	MCE-MIR_3663:fwd	0.64	0.83	0.79	0.74	1.06	1.38	1.70
4	Not Changed	MCE-MIR_3653:rev	0.97	0.88	1.44	2.00	1.18	0.36	-0.46
4	Not Changed	MCE-MIR_3651:fwd	-0.03	0.09	0.05	0.01	-0.01	-0.04	-0.07
4	Not Changed	MCE-MIR_3646:rev	0.08	0.68	0.36	0.04	0.11	0.18	0.26
4	Not Changed	MCE-MIR_3642:rev	0.47	0.61	0.82	1.03	0.88	0.73	0.58
4	Not Changed	MCE-MIR_364:fwd	-0.03	-0.04	0.10	0.25	0.50	0.74	0.98
4	Not Changed	MCE-MIR_3637:rev	0.04	-0.06	-0.02	0.02	0.13	0.24	0.36
4	Not Changed	MCE-MIR_3628:rev	-0.17	0.93	1.17	1.42	1.83	2.25	2.67
4	Not Changed	MCE-MIR_3626:rev	-0.41	0.33	0.35	0.37	0.68	0.99	1.30

Class	GCNF -/- Time Pattern	ProbeID	GCNF-Day0	GCNF-Day1	GCNF-Day2	GCNF-Day3	GCNF-Day4	GCNF-Day5	GCNF-Day6
4	Not Changed	MCE-MIR_3624:rev	0.45	0.70	0.14	-0.43	0.46	1.34	2.23
4	Not Changed	MCE-MIR_3624:fwd	0.37	0.28	0.10	-0.08	0.16	0.40	0.64
4	Not Changed	MCE-MIR_3619:rev	-0.01	-0.15	-0.02	0.11	0.14	0.18	0.22
4	Not Changed	MCE-MIR_3613:rev	0.53	0.01	-0.17	-0.36	-0.15	0.05	0.25
4	Not Changed	MCE-MIR_3609:rev	0.47	0.72	0.60	0.49	0.70	0.91	1.12
4	Not Changed	MCE-MIR_3595:fwd	0.29	-0.01	-0.04	-0.08	0.26	0.61	0.95
4	Not Changed	MCE-MIR_3573:rev	0.92	0.71	-0.99	-2.69	-1.20	0.30	1.79
4	Not Changed	MCE-MIR_3572:rev	1.02	0.64	0.42	0.19	1.13	2.08	3.03
4	Not Changed	MCE-MIR_3571:rev	0.25	0.09	-0.09	-0.28	-0.11	0.05	0.21
4	Not Changed	MCE-MIR_3557:rev	0.34	0.82	0.62	0.42	1.06	1.71	2.36
4	Not Changed	MCE-MIR_3550:fwd	-0.01	-0.02	0.10	0.22	0.27	0.31	0.36
4	Not Changed	MCE-MIR_3543:rev	-0.01	0.51	1.00	1.50	1.05	0.60	0.16
4	Not Changed	MCE-MIR_3541:rev	0.58	0.29	0.15	0.00	0.00	0.00	0.01
4	Not Changed	MCE-MIR_3531:rev	0.36	0.90	0.64	0.39	0.67	0.95	1.23
4	Not Changed	MCE-MIR_3529:rev	-0.82	0.04	1.13	2.22	1.69	1.16	0.63
4	Not Changed	MCE-MIR_3523:rev	0.25	0.65	0.32	0.00	0.65	1.31	1.96
4	Not Changed	MCE-MIR_3522:rev	0.01	-0.14	-0.08	-0.03	0.15	0.34	0.52
4	Not Changed	MCE-MIR_3518:rev	0.61	-0.06	0.08	0.22	0.76	1.30	1.84
4	Not Changed	MCE-MIR_3513:fwd	-0.06	-0.01	0.14	0.28	0.25	0.23	0.20
4	Not Changed	MCE-MIR_3502:rev	-0.10	0.14	0.20	0.25	0.34	0.43	0.51
4	Not Changed	MCE-MIR_3495:rev	0.57	-0.12	-0.56	-1.01	-0.51	-0.01	0.48
4	Not Changed	MCE-MIR_3491:rev	0.24	0.38	0.26	0.14	0.18	0.22	0.26
4	Not Changed	MCE-MIR_3490:rev	0.38	0.79	0.50	0.21	0.39	0.57	0.75
4	Not Changed	MCE-MIR_3488:rev	0.22	0.36	0.31	0.26	0.35	0.45	0.54
4	Not Changed	MCE-MIR_3485:rev	0.40	0.32	0.38	0.45	0.46	0.46	0.47
4	Not Changed	MCE-MIR_3484:rev	0.28	0.64	0.42	0.19	0.54	0.89	1.23
4	Not Changed	MCE-MIR_3471:rev	0.38	0.88	0.53	0.18	0.86	1.53	2.21
4	Not Changed	MCE-MIR_3444:rev	0.71	0.36	0.13	-0.11	-0.17	-0.24	-0.30
4	Not Changed	MCE-MIR_3441:fwd	0.27	0.05	0.72	1.39	0.70	0.01	-0.68
4	Not Changed	MCE-MIR_3439:rev	0.75	0.28	0.57	0.86	0.61	0.36	0.11
4	Not Changed	MCE-MIR_3429:rev	0.56	0.93	0.57	0.21	0.72	1.24	1.75
4	Not Changed	MCE-MIR_3408:fwd	0.43	-0.57	-0.81	-1.05	-0.77	-0.49	-0.22
4	Not Changed	MCE-MIR_3407:rev	0.58	0.80	0.69	0.59	0.34	0.09	-0.16
4	Not Changed	MCE-MIR_335:fwd	0.07	0.55	0.58	0.62	0.58	0.55	0.51
4	Not Changed	MCE-MIR_3334:fwd	0.48	0.49	0.57	0.65	0.40	0.16	-0.09
4	Not Changed	MCE-MIR_3333:rev	0.08	0.11	0.19	0.26	0.16	0.06	-0.03
4	Not Changed	MCE-MIR_3330:fwd	-0.08	-0.01	0.04	0.09	0.10	0.11	0.12
4	Not Changed	MCE-MIR_3295:rev	0.15	0.54	0.36	0.17	0.40	0.63	0.86

Class	GCNF -/- Time Pattern	ProbeID	GCNF-Day0	GCNF-Day1	GCNF-Day2	GCNF-Day3	GCNF-Day4	GCNF-Day5	GCNF-Day6
4	Not Changed	MCE-MIR_3261:fwd	0.21	-0.08	-0.09	-0.09	0.15	0.39	0.64
4	Not Changed	MCE-MIR_3260:rev	0.27	-0.26	-0.56	-0.86	0.01	0.88	1.75
4	Not Changed	MCE-MIR_321:rev	0.14	-0.62	-0.07	0.48	-0.12	-0.71	-1.31
4	Not Changed	MCE-MIR_3155:fwd	-0.20	0.23	0.45	0.67	0.57	0.47	0.36
4	Not Changed	MCE-MIR_3147:fwd	0.33	0.26	0.20	0.15	0.34	0.53	0.72
4	Not Changed	MCE-MIR_3134:rev	-0.47	-0.01	1.18	2.37	1.62	0.87	0.12
4	Not Changed	MCE-MIR_3113:fwd	0.26	0.28	0.10	-0.08	-0.09	-0.10	-0.12
4	Not Changed	MCE-MIR_3101:rev	0.40	-0.02	0.52	1.07	0.62	0.18	-0.26
4	Not Changed	MCE-MIR_3084:fwd	0.09	0.63	0.89	1.14	1.00	0.87	0.73
4	Not Changed	MCE-MIR_3059:rev	0.28	0.20	0.04	-0.13	-0.09	-0.05	-0.01
4	Not Changed	MCE-MIR_3057:rev	-0.01	-0.01	0.01	0.03	0.09	0.15	0.21
4	Not Changed	MCE-MIR_3057:fwd	0.04	0.10	0.17	0.24	0.16	0.08	0.01
4	Not Changed	MCE-MIR_3032:fwd	-0.06	-0.02	-0.05	-0.07	-0.01	0.06	0.13
4	Not Changed	MCE-MIR_3007:rev	0.02	0.03	0.01	-0.02	0.00	0.02	0.04
4	Not Changed	MCE-MIR_2983:rev	1.02	0.82	0.89	0.96	0.90	0.85	0.79
4	Not Changed	MCE-MIR_2977:rev	0.78	-0.42	0.26	0.95	0.31	-0.32	-0.95
4	Not Changed	MCE-MIR_2968:fwd	1.43	1.10	0.84	0.59	0.34	0.08	-0.17
4	Not Changed	MCE-MIR_2953:fwd	-0.60	0.33	0.67	1.00	0.94	0.87	0.81
4	Not Changed	MCE-MIR_293:rev	-0.08	-0.02	0.02	0.06	0.12	0.18	0.23
4	Not Changed	MCE-MIR_291:rev	0.05	0.00	0.05	0.10	0.06	0.02	-0.02
4	Not Changed	MCE-MIR_2894:fwd	0.07	0.18	0.23	0.29	0.36	0.43	0.50
4	Not Changed	MCE-MIR_2889:fwd	0.54	0.57	0.39	0.20	0.04	-0.12	-0.28
4	Not Changed	MCE-MIR_2866:rev	0.67	-0.63	-0.14	0.34	-0.09	-0.51	-0.94
4	Not Changed	MCE-MIR_2817:fwd	0.02	1.12	0.80	0.47	1.13	1.79	2.45
4	Not Changed	MCE-MIR_281:rev	-0.01	0.03	0.03	0.04	0.03	0.01	-0.01
4	Not Changed	MCE-MIR_2798:fwd	-0.02	0.02	0.00	-0.01	0.03	0.06	0.10
4	Not Changed	MCE-MIR_2745:fwd	1.15	0.57	0.67	0.78	0.15	-0.48	-1.11
4	Not Changed	MCE-MIR_273:fwd	0.20	0.84	0.54	0.23	0.45	0.66	0.88
4	Not Changed	MCE-MIR_2722:rev	0.07	0.22	0.08	-0.05	0.04	0.14	0.23
4	Not Changed	MCE-MIR_2711:rev	0.04	0.03	0.23	0.44	0.31	0.18	0.05
4	Not Changed	MCE-MIR_2698:rev	0.56	0.43	0.00	-0.44	0.27	0.97	1.68
4	Not Changed	MCE-MIR_2691:rev	-0.01	-0.03	0.00	0.03	0.04	0.05	0.06
4	Not Changed	MCE-MIR_2680:rev	0.31	0.08	0.59	1.10	0.58	0.05	-0.47
4	Not Changed	MCE-MIR_2680:fwd	1.22	-0.02	0.61	1.23	0.16	-0.91	-1.97
4	Not Changed	MCE-MIR_2679:fwd	0.43	0.00	-0.26	-0.52	0.66	1.83	3.01
4	Not Changed	MCE-MIR_2661:rev	0.37	0.17	0.27	0.36	0.27	0.17	0.08
4	Not Changed	MCE-MIR_2624:rev	0.02	0.27	0.20	0.13	0.13	0.13	0.12
4	Not Changed	MCE-MIR_2617:fwd	0.11	0.08	0.17	0.27	0.26	0.26	0.25

Class	GCNF -/- Time Pattern	ProbeID	GCNF-Day0	GCNF-Day1	GCNF-Day2	GCNF-Day3	GCNF-Day4	GCNF-Day5	GCNF-Day6
4	Not Changed	MCE-MIR_2566:rev	-0.08	0.04	0.19	0.34	0.26	0.19	0.11
4	Not Changed	MCE-MIR_2566:fwd	0.54	0.60	0.56	0.53	0.28	0.03	-0.22
4	Not Changed	MCE-MIR_2563:rev	0.52	0.53	0.53	0.53	0.53	0.54	0.54
4	Not Changed	MCE-MIR_254:fwd	0.03	0.11	0.14	0.16	0.15	0.14	0.13
4	Not Changed	MCE-MIR_2522:rev	-0.04	0.04	0.02	0.00	-0.01	-0.01	-0.02
4	Not Changed	MCE-MIR_2501:fwd	-0.13	0.01	-0.02	-0.04	-0.08	-0.11	-0.14
4	Not Changed	MCE-MIR_2474:rev	-0.07	-0.01	0.07	0.14	0.03	-0.08	-0.20
4	Not Changed	MCE-MIR_2470:fwd	0.41	0.41	0.12	-0.16	-0.15	-0.15	-0.14
4	Not Changed	MCE-MIR_2464:fwd	0.14	0.16	0.16	0.15	0.00	-0.16	-0.31
4	Not Changed	MCE-MIR_2419:fwd	-0.05	0.02	0.02	0.02	0.03	0.05	0.06
4	Not Changed	MCE-MIR_2417:fwd	-0.07	0.05	0.03	0.01	0.00	-0.01	-0.02
4	Not Changed	MCE-MIR_2388:rev	-0.02	-0.31	-0.20	-0.10	-0.17	-0.25	-0.32
4	Not Changed	MCE-MIR_2371:rev	-0.05	0.01	0.01	0.01	0.17	0.32	0.48
4	Not Changed	MCE-MIR_2371:fwd	-0.05	-0.01	0.05	0.12	0.05	-0.01	-0.07
4	Not Changed	MCE-MIR_2364:rev	0.00	-0.08	0.06	0.20	0.04	-0.11	-0.26
4	Not Changed	MCE-MIR_2361:fwd	0.59	-0.21	1.24	2.69	1.17	-0.36	-1.89
4	Not Changed	MCE-MIR_2345:fwd	0.17	0.42	1.58	2.74	1.06	-0.61	-2.29
4	Not Changed	MCE-MIR_2339:rev	-0.12	0.13	0.09	0.06	-0.01	-0.08	-0.15
4	Not Changed	MCE-MIR_2339:fwd	0.07	0.07	0.08	0.09	0.18	0.28	0.37
4	Not Changed	MCE-MIR_2327:fwd	0.97	0.48	-0.36	-1.21	-0.77	-0.32	0.12
4	Not Changed	MCE-MIR_2304:rev	0.55	0.27	0.08	-0.11	0.01	0.13	0.25
4	Not Changed	MCE-MIR_2288:fwd	0.04	0.01	0.09	0.16	0.43	0.71	0.98
4	Not Changed	MCE-MIR_2243:fwd	0.18	0.05	-0.09	-0.24	0.06	0.35	0.64
4	Not Changed	MCE-MIR_2222:fwd	-0.72	-0.23	-0.42	-0.61	-0.66	-0.72	-0.77
4	Not Changed	MCE-MIR_2205:fwd	0.09	-0.03	0.02	0.08	0.11	0.15	0.19
4	Not Changed	MCE-MIR_2198:fwd	0.01	-0.08	-0.02	0.04	0.09	0.14	0.20
4	Not Changed	MCE-MIR_2197:fwd	0.06	-0.08	-0.02	0.04	0.11	0.17	0.24
4	Not Changed	MCE-MIR_2196:fwd	0.92	0.17	0.59	1.01	1.00	0.99	0.98
4	Not Changed	MCE-MIR_2192:rev	0.37	0.04	0.24	0.44	0.25	0.07	-0.12
4	Not Changed	MCE-MIR_2173:rev	0.19	0.28	0.48	0.67	0.51	0.36	0.20
4	Not Changed	MCE-MIR_2171:rev	-0.48	0.51	0.61	0.70	1.05	1.40	1.74
4	Not Changed	MCE-MIR_2166:rev	0.19	0.27	0.34	0.40	0.23	0.05	-0.12
4	Not Changed	MCE-MIR_2166:fwd	-0.03	-0.07	-0.04	0.00	0.09	0.19	0.28
4	Not Changed	MCE-MIR_2164:rev	0.08	0.03	0.14	0.24	0.16	0.08	0.00
4	Not Changed	MCE-MIR_2139:fwd	0.23	0.22	-0.40	-1.02	-0.11	0.79	1.69
4	Not Changed	MCE-MIR_2134:fwd	0.20	-0.05	0.32	0.69	0.40	0.12	-0.16
4	Not Changed	MCE-MIR_2099:fwd	0.40	0.52	0.06	-0.41	-0.30	-0.19	-0.07
4	Not Changed	MCE-MIR_2092:fwd	0.65	0.18	0.41	0.63	0.24	-0.14	-0.53

Class	GCNF -/- Time Pattern	ProbeID	GCNF-Day0	GCNF-Day1	GCNF-Day2	GCNF-Day3	GCNF-Day4	GCNF-Day5	GCNF-Day6
4	Not Changed	MCE-MIR_2087:rev	0.06	0.00	0.00	-0.01	0.01	0.03	0.04
4	Not Changed	MCE-MIR_2078:fwd	1.35	2.14	1.56	0.98	1.71	2.45	3.19
4	Not Changed	MCE-MIR_1998:fwd	-0.03	-0.11	-0.07	-0.03	0.04	0.11	0.18
4	Not Changed	MCE-MIR_1974:fwd	0.67	-0.59	1.20	2.99	1.47	-0.04	-1.56
4	Not Changed	MCE-MIR_1973:fwd	0.78	-0.30	0.87	2.03	0.42	-1.20	-2.81
4	Not Changed	MCE-MIR_1929:fwd	-0.01	-0.02	-0.03	-0.04	0.00	0.04	0.08
4	Not Changed	MCE-MIR_1905:rev	0.02	0.05	0.15	0.25	0.21	0.16	0.12
4	Not Changed	MCE-MIR_188:fwd	-0.02	0.01	-0.05	-0.11	-0.02	0.07	0.16
4	Not Changed	MCE-MIR_1829:rev	-0.06	0.18	0.46	0.74	0.44	0.14	-0.16
4	Not Changed	MCE-MIR_1811:rev	0.00	-0.12	-0.05	0.02	0.07	0.12	0.18
4	Not Changed	MCE-MIR_1793:fwd	1.00	0.09	0.59	1.08	0.63	0.17	-0.28
4	Not Changed	MCE-MIR_1792:rev	0.01	-0.54	0.29	1.12	0.02	-1.09	-2.19
4	Not Changed	MCE-MIR_1786:fwd	0.02	-0.07	0.03	0.13	0.06	-0.01	-0.08
4	Not Changed	MCE-MIR_1778:fwd	0.27	0.21	0.26	0.32	0.60	0.88	1.16
4	Not Changed	MCE-MIR_1773:rev	0.45	0.19	0.28	0.37	0.50	0.62	0.75
4	Not Changed	MCE-MIR_1756:rev	0.88	0.57	0.75	0.93	0.63	0.34	0.04
4	Not Changed	MCE-MIR_1742:rev	-0.21	-0.42	0.24	0.91	0.29	-0.33	-0.95
4	Not Changed	MCE-MIR_1710:rev	0.00	-0.02	-0.01	0.00	-0.01	-0.01	-0.02
4	Not Changed	MCE-MIR_1697:fwd	0.09	0.27	0.20	0.13	0.09	0.06	0.02
4	Not Changed	MCE-MIR_1689:rev	0.10	0.13	0.08	0.03	0.17	0.31	0.45
4	Not Changed	MCE-MIR_1679:fwd	0.01	0.04	0.00	-0.04	0.08	0.21	0.34
4	Not Changed	MCE-MIR_1670:fwd	0.03	0.09	0.46	0.83	0.58	0.32	0.07
4	Not Changed	MCE-MIR_1645:rev	0.02	0.04	0.01	-0.01	-0.01	-0.02	-0.02
4	Not Changed	MCE-MIR_1642:rev	-0.03	0.40	0.34	0.27	0.64	1.00	1.36
4	Not Changed	MCE-MIR_162:fwd	0.23	0.26	0.22	0.17	0.14	0.11	0.08
4	Not Changed	MCE-MIR_1611:fwd	0.48	0.66	0.86	1.06	0.76	0.46	0.15
4	Not Changed	MCE-MIR_1597:fwd	0.18	0.06	-0.03	-0.11	-0.09	-0.06	-0.03
4	Not Changed	MCE-MIR_1576:rev	0.37	0.35	0.51	0.66	0.48	0.30	0.12
4	Not Changed	MCE-MIR_1569:rev	0.57	0.61	0.84	1.07	0.84	0.60	0.37
4	Not Changed	MCE-MIR_1546:rev	0.04	0.04	0.05	0.07	0.07	0.08	0.08
4	Not Changed	MCE-MIR_1544:rev	0.49	1.23	0.69	0.15	0.74	1.33	1.91
4	Not Changed	MCE-MIR_1538:fwd	0.50	0.62	0.43	0.23	0.10	-0.03	-0.16
4	Not Changed	MCE-MIR_1536:rev	0.18	0.57	0.23	-0.11	0.40	0.91	1.43
4	Not Changed	MCE-MIR_1535:rev	0.07	0.22	0.00	-0.22	0.10	0.41	0.73
4	Not Changed	MCE-MIR_151:fwd	-0.03	-0.03	0.02	0.07	0.11	0.15	0.19
4	Not Changed	MCE-MIR_1508:fwd	0.59	0.50	0.20	-0.09	-0.09	-0.10	-0.10
4	Not Changed	MCE-MIR_1495:fwd	0.36	0.42	0.41	0.40	0.27	0.13	-0.01
4	Not Changed	MCE-MIR_1482:rev	0.22	0.05	-0.01	-0.06	-0.07	-0.09	-0.11

Class	GCNF -/- Time Pattern	ProbeID	GCNF-Day0	GCNF-Day1	GCNF-Day2	GCNF-Day3	GCNF-Day4	GCNF-Day5	GCNF-Day6
4	Not Changed	MCE-MIR_1478:fwd	0.24	0.84	0.44	0.03	0.50	0.96	1.43
4	Not Changed	MCE-MIR_1458:rev	0.39	0.73	0.36	0.00	0.15	0.31	0.46
4	Not Changed	MCE-MIR_1442:fwd	-0.04	0.01	-0.03	-0.08	-0.02	0.04	0.10
4	Not Changed	MCE-MIR_1433:rev	0.04	0.09	0.03	-0.02	0.01	0.04	0.07
4	Not Changed	MCE-MIR_1433:fwd	0.01	0.06	0.00	-0.07	0.08	0.22	0.37
4	Not Changed	MCE-MIR_1412:fwd	0.08	0.09	0.04	-0.02	0.00	0.02	0.03
4	Not Changed	MCE-MIR_1409:fwd	0.01	0.54	0.42	0.29	0.47	0.65	0.83
4	Not Changed	MCE-MIR_1408:fwd	1.07	-0.54	0.74	2.03	0.55	-0.93	-2.41
4	Not Changed	MCE-MIR_1401:fwd	0.03	0.03	0.01	0.00	0.01	0.03	0.04
4	Not Changed	MCE-MIR_1365:rev	0.05	-0.11	0.04	0.19	0.16	0.13	0.09
4	Not Changed	MCE-MIR_1365:fwd	0.25	0.49	-0.03	-0.54	0.23	0.99	1.76
4	Not Changed	MCE-MIR_1364:fwd	0.73	0.67	0.89	1.11	0.11	-0.89	-1.89
4	Not Changed	MCE-MIR_1356:rev	-0.05	-0.03	0.07	0.17	0.11	0.05	0.00
4	Not Changed	MCE-MIR_1356:fwd	0.14	-0.02	0.18	0.38	0.21	0.05	-0.11
4	Not Changed	MCE-MIR_1352:fwd	0.59	0.50	0.20	-0.10	0.16	0.43	0.69
4	Not Changed	MCE-MIR_1342:fwd	0.89	0.30	0.19	0.08	-0.14	-0.35	-0.57
4	Not Changed	MCE-MIR_1325:rev	0.37	0.76	0.47	0.19	0.29	0.39	0.49
4	Not Changed	MCE-MIR_1311:rev	-0.11	-0.07	0.05	0.17	0.08	-0.01	-0.10
4	Not Changed	MCE-MIR_1283:rev	0.24	-0.53	0.74	2.01	1.14	0.27	-0.60
4	Not Changed	MCE-MIR_1283:fwd	0.50	0.24	0.16	0.07	0.52	0.97	1.42
4	Not Changed	MCE-MIR_1269:rev	0.02	0.06	0.06	0.06	0.05	0.04	0.03
4	Not Changed	MCE-MIR_1264:rev	-0.02	0.00	0.26	0.51	0.34	0.16	-0.02
4	Not Changed	MCE-MIR_1259:rev	0.04	0.06	0.02	-0.01	0.02	0.05	0.08
4	Not Changed	MCE-MIR_1192:rev	0.06	0.06	0.21	0.37	0.46	0.55	0.64
4	Not Changed	MCE-MIR_1190:fwd	0.18	0.17	0.26	0.35	0.18	0.01	-0.16
4	Not Changed	MCE-MIR_1066:rev	0.82	0.45	0.93	1.41	0.65	-0.11	-0.87
4	Not Changed	MCE-MIR_1059:fwd	0.10	0.24	0.23	0.23	0.24	0.25	0.27
4	Not Changed	MCE-MIR_1052:rev	-0.04	0.09	0.09	0.09	0.13	0.16	0.20
4	Not Changed	MCE-MIR_1038:fwd	0.11	0.17	-0.21	-0.59	-0.22	0.14	0.51
4	Not Changed	MCE-MIR_1015:rev	0.33	0.75	0.67	0.60	0.84	1.08	1.33
1	Changed	mmu-mir-485-3p	-1.21	-1.16	-1.19	-1.23	-1.28	-1.34	-1.39
1	Changed	mmu-mir-484	-0.50	-0.15	-0.09	-0.03	-0.52	-1.00	-1.49
1	Changed	mmu-mir-467	0.94	1.15	1.22	1.29	1.29	1.29	1.28
1	Changed	mmu-mir-466	1.08	1.19	1.31	1.43	1.15	0.87	0.59
1	Changed	mmu-mir-302d	2.67	2.79	1.77	0.75	-0.56	-1.86	-3.16
1	Changed	mmu-mir-302b	3.04	3.05	2.22	1.39	0.09	-1.21	-2.51
1	Changed	mmu-mir-302	2.79	2.57	1.30	0.02	-0.62	-1.26	-1.90
1	Changed	mmu-mir-295	2.17	2.02	1.56	1.09	1.11	1.13	1.14

Class	GCNF -/- Time Pattern	ProbeID	GCNF-Day0	GCNF-Day1	GCNF-Day2	GCNF-Day3	GCNF-Day4	GCNF-Day5	GCNF-Day6
1	Changed	mmu-mir-293	1.09	1.07	0.81	0.56	0.38	0.21	0.03
1	Changed	mmu-mir-292-5p	1.05	1.22	0.92	0.62	0.39	0.16	-0.07
1	Changed	mmu-mir-292-3p	1.09	1.12	0.91	0.70	0.66	0.63	0.59
1	Changed	mmu-mir-291b-5p	1.55	1.55	1.29	1.03	0.88	0.73	0.58
1	Changed	mmu-mir-291a-5p	1.83	1.79	1.51	1.23	0.82	0.42	0.01
1	Changed	mmu-mir-291a-3p	2.22	0.75	0.14	-0.47	-0.77	-1.08	-1.38
1	Changed	mmu-mir-290	1.03	0.99	0.68	0.37	0.23	0.09	-0.05
1	Changed	mmu-mir-22	-0.86	-0.99	-0.97	-0.95	-0.35	0.25	0.85
1	Changed	mmu-mir-195	-0.21	0.04	-0.68	-1.40	-1.00	-0.61	-0.21
1	Changed	mmu-mir-182	0.94	0.73	0.07	-0.59	-0.53	-0.47	-0.41
1	Changed	mmu-mir-150	0.16	0.28	-0.05	-0.38	-0.29	-0.20	-0.12
1	Not Changed	mmu-mir-96	-0.03	-0.08	-0.32	-0.57	-0.60	-0.62	-0.65
1	Not Changed	mmu-mir-92	0.34	0.46	0.02	-0.41	-0.58	-0.75	-0.92
1	Not Changed	mmu-mir-7	0.94	0.98	0.58	0.17	-0.47	-1.11	-1.75
1	Not Changed	mmu-mir-363	1.07	1.60	0.99	0.37	-0.19	-0.75	-1.31
1	Not Changed	mmu-mir-34a	0.21	0.59	0.17	-0.25	0.04	0.33	0.61
1	Not Changed	mmu-mir-31	0.37	0.80	0.15	-0.49	0.02	0.53	1.04
1	Not Changed	S-mmu-mir-302c	0.65	0.71	0.19	-0.34	-0.42	-0.51	-0.59
1	Not Changed	mmu-mir-302c	1.23	0.47	0.14	-0.18	-0.48	-0.79	-1.09
1	Not Changed	mmu-mir-300	-0.79	-0.68	-0.84	-1.00	-1.07	-1.14	-1.21
1	Not Changed	mmu-mir-299	-1.05	-1.01	-1.08	-1.16	-1.20	-1.24	-1.28
1	Not Changed	mmu-mir-297	0.71	0.46	0.32	0.19	-0.27	-0.72	-1.17
1	Not Changed	mmu-mir-294	1.28	0.70	0.50	0.29	-0.66	-1.61	-2.56
1	Not Changed	mmu-mir-20b	0.58	0.70	0.02	-0.65	-0.85	-1.05	-1.25
1	Not Changed	mmu-mir-205	1.25	1.33	0.67	0.01	0.38	0.75	1.11
1	Not Changed	mmu-mir-200b	0.55	0.91	0.53	0.15	0.35	0.55	0.75
1	Not Changed	mmu-mir-200a	0.18	0.61	0.04	-0.52	-0.23	0.06	0.35
1	Not Changed	mmu-mir-183	0.75	0.42	0.02	-0.38	-0.33	-0.28	-0.24
1	Not Changed	mmu-mir-18	0.18	-0.01	-1.03	-2.04	-2.04	-2.04	-2.04
1	Not Changed	mmu-mir-148b	-0.85	-0.74	-0.82	-0.89	-0.90	-0.91	-0.92
1	Not Changed	mmu-mir-148a	0.10	0.47	-0.41	-1.29	-0.88	-0.47	-0.06
1	Not Changed	mmu-mir-101b	0.01	-0.71	-1.18	-1.65	-1.50	-1.34	-1.19
1	Not Changed	mmu-mir-101a	-0.08	-0.47	-0.63	-0.79	-0.64	-0.50	-0.35
3	Changed	mmu-mir-99a	-2.64	-2.50	-2.54	-2.57	-1.76	-0.95	-0.15
3	Changed	S-mmu-mir-9	-1.92	-1.77	-1.89	-2.00	-1.43	-0.86	-0.29
3	Changed	mmu-mir-9	-2.41	-2.32	-2.25	-2.18	-1.82	-1.47	-1.11
3	Changed	mmu-mir-542-3p	-0.32	-0.29	-0.32	-0.34	-0.32	-0.30	-0.28
3	Changed	mmu-mir-503	-0.97	-0.75	-0.59	-0.44	-0.17	0.09	0.36

Class	GCNF -/- Time Pattern	ProbeID	GCNF-Day0	GCNF-Day1	GCNF-Day2	GCNF-Day3	GCNF-Day4	GCNF-Day5	GCNF-Day6
3	Changed	mmu-mir-450	-0.91	-0.86	-0.97	-1.08	-1.02	-0.97	-0.91
3	Changed	mmu-mir-424	-1.91	-1.75	-1.75	-1.76	-1.04	-0.33	0.39
3	Changed	mmu-mir-383	-0.07	-0.07	0.06	0.18	0.11	0.04	-0.03
3	Changed	mmu-mir-376a	-1.49	-1.40	-1.48	-1.56	-1.41	-1.26	-1.10
3	Changed	mmu-mir-351	-1.72	-1.47	-1.45	-1.43	-0.83	-0.22	0.38
3	Changed	mmu-mir-344	-1.44	-1.26	-1.42	-1.58	-1.52	-1.46	-1.41
3	Changed	mmu-mir-335	-2.74	-2.92	-2.69	-2.45	-2.61	-2.76	-2.92
3	Changed	mmu-mir-322	-0.92	-0.87	-0.85	-0.83	-0.66	-0.49	-0.32
3	Changed	mmu-mir-218	-2.38	-2.33	-2.36	-2.39	-2.04	-1.69	-1.34
3	Changed	mmu-mir-214	-1.88	-1.42	-0.87	-0.32	0.33	0.99	1.65
3	Changed	S-mmu-mir-199a	-2.26	-2.17	-2.27	-2.36	-1.41	-0.46	0.49
3	Changed	mmu-mir-181a	-2.77	-2.33	-2.42	-2.50	-1.50	-0.51	0.49
3	Changed	mmu-mir-134	-1.45	-1.39	-1.93	-2.47	-1.90	-1.33	-0.75
3	Changed	mmu-mir-132	-0.38	-0.35	-0.27	-0.19	-0.26	-0.32	-0.39
3	Changed	mmu-mir-125b	-2.98	-2.93	-2.09	-1.24	-0.72	-0.21	0.31
3	Changed	mmu-mir-10b	-3.56	-3.44	-2.71	-1.98	-1.77	-1.56	-1.34
3	Changed	mmu-mir-10a	-5.09	-3.18	-2.58	-1.98	-1.84	-1.70	-1.56
3	Changed	mmu-mir-100	-2.52	-2.33	-2.38	-2.43	-2.08	-1.74	-1.39
3	Not Changed	mmu-mir-99b	-1.59	-1.21	-1.32	-1.42	-0.87	-0.32	0.23
3	Not Changed	mmu-mir-216	-0.18	-0.21	-0.20	-0.20	-0.15	-0.10	-0.05
3	Not Changed	mmu-mir-199b	-0.29	-0.31	-0.34	-0.38	-0.30	-0.23	-0.15
3	Not Changed	mmu-mir-199a	-0.68	-0.60	-0.74	-0.87	-0.48	-0.09	0.30
3	Not Changed	mmu-mir-188	-0.03	0.04	-0.11	-0.27	0.07	0.41	0.75
3	Not Changed	mmu-mir-181b	-2.17	-1.71	-1.76	-1.81	-1.33	-0.86	-0.38
3	Not Changed	mmu-mir-125a	-1.70	-1.03	-1.04	-1.06	-0.64	-0.22	0.19
3	Not Changed	mmu-let-7i	-0.61	-0.35	-0.59	-0.83	0.14	1.11	2.08
3	Not Changed	mmu-let-7f	-1.38	-0.69	-0.49	-0.29	0.20	0.69	1.18
3	Not Changed	mmu-let-7e	-1.64	-0.58	-0.40	-0.21	0.22	0.65	1.08
3	Not Changed	mmu-let-7d	-1.40	-0.63	-0.42	-0.20	0.29	0.78	1.27
3	Not Changed	mmu-let-7c	-1.99	-0.54	-0.34	-0.13	0.39	0.91	1.44
3	Not Changed	mmu-let-7b	-1.54	-0.44	-0.32	-0.19	0.36	0.91	1.46
3	Not Changed	mmu-let-7a	-2.03	-0.71	-0.43	-0.15	0.27	0.69	1.11
4	Changed	mmu-mir-489	-0.05	-0.01	-0.04	-0.07	-0.08	-0.09	-0.11
4	Changed	mmu-mir-468	0.01	0.59	0.69	0.78	0.75	0.73	0.70
4	Changed	mmu-mir-339	-0.06	-0.07	-0.12	-0.18	-0.19	-0.21	-0.23
4	Changed	mmu-mir-329	-2.58	-2.31	-2.35	-2.38	-2.52	-2.66	-2.80
4	Changed	mmu-mir-20a	0.31	0.27	-0.41	-1.08	-1.11	-1.14	-1.17
4	Changed	mmu-mir-207	0.11	0.06	0.83	1.60	1.10	0.60	0.10

Class	GCNF -/- Time Pattern	ProbeID	GCNF-Day0	GCNF-Day1	GCNF-Day2	GCNF-Day3	GCNF-Day4	GCNF-Day5	GCNF-Day6
4	Changed	mmu-mir-17-5p	0.20	0.31	-0.34	-0.99	-1.05	-1.12	-1.18
4	Changed	mmu-mir-138	-0.13	-0.16	-0.15	-0.13	0.17	0.47	0.77
4	Changed	mmu-mir-129-5p	0.45	0.21	0.47	0.73	0.36	-0.01	-0.38
4	Not Changed	mmu-mir-98	-0.30	-0.25	-0.15	-0.04	0.08	0.21	0.34
4	Not Changed	mmu-mir-93	-0.03	-0.15	-0.59	-1.03	-0.82	-0.61	-0.40
4	Not Changed	mmu-mir-7b	-0.03	0.11	0.07	0.03	0.04	0.05	0.07
4	Not Changed	mmu-mir-547	-0.02	0.03	0.00	-0.03	-0.01	0.01	0.03
4	Not Changed	mmu-mir-546	0.31	-0.14	0.32	0.77	0.50	0.24	-0.03
4	Not Changed	mmu-mir-543	-2.32	-2.12	-2.44	-2.75	-2.77	-2.78	-2.80
4	Not Changed	mmu-mir-542-5p	-0.12	-0.22	-0.08	0.05	0.09	0.12	0.16
4	Not Changed	mmu-mir-541	-2.07	-2.10	-2.73	-3.37	-3.31	-3.25	-3.19
4	Not Changed	mmu-mir-540	-1.25	-1.36	-1.57	-1.78	-1.79	-1.79	-1.79
4	Not Changed	mmu-mir-539	-1.03	-0.98	-1.01	-1.04	-1.01	-0.99	-0.96
4	Not Changed	mmu-mir-494	-0.09	0.52	0.48	0.43	-0.47	-1.37	-2.28
4	Not Changed	mmu-mir-487b	-1.64	-1.28	-1.48	-1.68	-1.73	-1.77	-1.82
4	Not Changed	mmu-mir-486	-0.11	-0.16	-0.07	0.03	0.01	0.00	-0.02
4	Not Changed	mmu-mir-485-5p	-0.88	-0.96	-1.02	-1.08	-1.14	-1.20	-1.26
4	Not Changed	mmu-mir-483	-0.09	-0.05	0.03	0.11	0.11	0.11	0.11
4	Not Changed	mmu-mir-471	0.01	0.05	0.02	-0.01	0.02	0.06	0.10
4	Not Changed	mmu-mir-470	-0.83	-0.72	-0.61	-0.50	-0.59	-0.68	-0.77
4	Not Changed	mmu-mir-469	-0.02	0.02	0.01	0.01	0.04	0.07	0.11
4	Not Changed	mmu-mir-465	0.00	-0.03	-0.02	0.00	0.01	0.02	0.03
4	Not Changed	mmu-mir-464	0.06	0.04	0.00	-0.04	0.00	0.03	0.07
4	Not Changed	mmu-mir-463	0.01	0.04	-0.02	-0.09	0.02	0.14	0.25
4	Not Changed	mmu-mir-452	0.01	-0.11	-0.06	-0.01	-0.05	-0.09	-0.14
4	Not Changed	mmu-mir-451	1.57	1.19	0.52	-0.14	0.56	1.27	1.97
4	Not Changed	mmu-mir-449	0.08	0.02	0.01	0.00	0.00	0.00	0.01
4	Not Changed	mmu-mir-448	0.04	0.00	-0.02	-0.03	-0.03	-0.03	-0.04
4	Not Changed	mmu-mir-434-5p	-1.12	-1.25	-1.28	-1.31	-1.30	-1.29	-1.28
4	Not Changed	mmu-mir-434-3p	-2.60	-2.79	-3.20	-3.61	-3.14	-2.68	-2.21
4	Not Changed	mmu-mir-433-5p	-0.25	-0.22	-0.17	-0.12	-0.17	-0.22	-0.27
4	Not Changed	mmu-mir-433-3p	-2.48	-2.14	-2.53	-2.92	-2.96	-3.00	-3.04
4	Not Changed	mmu-mir-431	-2.35	-2.09	-2.35	-2.60	-2.52	-2.43	-2.35
4	Not Changed	mmu-mir-429	0.19	0.27	-0.33	-0.93	-0.61	-0.30	0.02
4	Not Changed	mmu-mir-425	0.29	0.01	-0.15	-0.32	-0.21	-0.11	-0.01
4	Not Changed	mmu-mir-422b	0.17	-0.28	-0.85	-1.43	-1.07	-0.72	-0.36
4	Not Changed	mmu-mir-412	-0.02	-0.05	-0.24	-0.43	-0.40	-0.37	-0.33
4	Not Changed	mmu-mir-411	-0.19	-0.13	0.05	0.23	0.19	0.15	0.11

Class	GCNF -/- Time Pattern	ProbeID	GCNF-Day0	GCNF-Day1	GCNF-Day2	GCNF-Day3	GCNF-Day4	GCNF-Day5	GCNF-Day6
4	Not Changed	mmu-mir-410	-2.53	-2.50	-2.55	-2.60	-2.59	-2.58	-2.57
4	Not Changed	mmu-mir-409	-2.10	-2.07	-2.01	-1.95	-2.45	-2.96	-3.46
4	Not Changed	mmu-mir-384	0.01	0.02	0.04	0.07	0.06	0.05	0.04
4	Not Changed	mmu-mir-382	-2.66	-2.61	-3.05	-3.49	-3.44	-3.39	-3.34
4	Not Changed	mmu-mir-381	-0.88	-0.95	-0.99	-1.04	-1.13	-1.23	-1.32
4	Not Changed	mmu-mir-380-5p	-0.36	-0.39	-0.37	-0.35	-0.36	-0.37	-0.39
4	Not Changed	mmu-mir-380-3p	-1.48	-1.51	-1.54	-1.57	-1.59	-1.60	-1.62
4	Not Changed	mmu-mir-379	-2.32	-2.34	-3.16	-3.99	-3.85	-3.72	-3.58
4	Not Changed	mmu-mir-378	0.03	0.09	0.05	0.00	-0.05	-0.11	-0.17
4	Not Changed	mmu-mir-377	-0.57	0.45	0.42	0.38	0.41	0.45	0.48
4	Not Changed	mmu-mir-376c	-0.25	-0.18	-0.23	-0.27	-0.29	-0.31	-0.33
4	Not Changed	S-mmu-mir-376b	-0.31	-0.23	-0.27	-0.31	-0.32	-0.34	-0.35
4	Not Changed	mmu-mir-376b	-2.14	-2.49	-3.25	-4.01	-3.77	-3.53	-3.28
4	Not Changed	S-mmu-mir-376a	-0.10	-0.04	-0.09	-0.14	-0.04	0.07	0.17
4	Not Changed	mmu-mir-375	-0.16	-0.02	-0.04	-0.07	0.03	0.13	0.23
4	Not Changed	mmu-mir-370	-1.84	-1.78	-1.94	-2.09	-2.17	-2.25	-2.33
4	Not Changed	mmu-mir-369-5p	-0.33	-0.22	-0.30	-0.38	-0.37	-0.36	-0.34
4	Not Changed	mmu-mir-369-3p	-0.10	-0.09	-0.12	-0.16	-0.15	-0.15	-0.15
4	Not Changed	mmu-mir-367	0.03	0.04	0.01	-0.02	-0.05	-0.08	-0.11
4	Not Changed	mmu-mir-365	-0.19	-0.19	-0.08	0.03	-0.06	-0.15	-0.24
4	Not Changed	mmu-mir-362	-0.18	0.14	0.00	-0.14	-0.11	-0.07	-0.04
4	Not Changed	mmu-mir-361	-0.50	0.09	-0.20	-0.48	-0.53	-0.58	-0.63
4	Not Changed	mmu-mir-350	-0.52	-0.19	-0.42	-0.66	-0.66	-0.67	-0.68
4	Not Changed	mmu-mir-34c	-0.14	-0.17	-0.23	-0.29	-0.12	0.05	0.23
4	Not Changed	mmu-mir-34b	-0.01	-0.10	-0.06	-0.03	0.02	0.06	0.10
4	Not Changed	mmu-mir-346	0.40	0.90	0.69	0.47	0.94	1.40	1.86
4	Not Changed	mmu-mir-345	-0.46	-0.59	-0.55	-0.52	-0.67	-0.83	-0.99
4	Not Changed	mmu-mir-342	-0.99	-0.65	-0.51	-0.37	-0.43	-0.49	-0.54
4	Not Changed	mmu-mir-341	0.03	0.42	0.30	0.17	0.68	1.19	1.70
4	Not Changed	mmu-mir-340	-0.08	-0.01	-0.05	-0.09	-0.07	-0.04	-0.02
4	Not Changed	mmu-mir-338	-0.01	-0.05	-0.04	-0.03	-0.01	0.02	0.04
4	Not Changed	mmu-mir-337	-2.15	-2.06	-2.25	-2.43	-2.40	-2.36	-2.33
4	Not Changed	mmu-mir-331	-0.57	-0.45	-0.40	-0.36	-0.50	-0.64	-0.78
4	Not Changed	mmu-mir-330	-0.19	0.25	-0.04	-0.33	-0.39	-0.45	-0.52
4	Not Changed	mmu-mir-33	0.01	0.08	0.06	0.05	0.01	-0.03	-0.08
4	Not Changed	mmu-mir-328	-0.60	-0.37	-0.02	0.34	0.04	-0.27	-0.57
4	Not Changed	mmu-mir-326	-0.06	0.03	0.06	0.09	0.04	-0.01	-0.06
4	Not Changed	mmu-mir-325	-0.16	-0.18	-0.12	-0.06	-0.11	-0.15	-0.19

Class	GCNF -/- Time Pattern	ProbeID	GCNF-Day0	GCNF-Day1	GCNF-Day2	GCNF-Day3	GCNF-Day4	GCNF-Day5	GCNF-Day6
4	Not Changed	mmu-mir-324-5p	-0.73	-0.32	-0.79	-1.27	-0.95	-0.62	-0.30
4	Not Changed	mmu-mir-324-3p	-0.35	-0.11	-0.53	-0.94	-0.77	-0.59	-0.42
4	Not Changed	mmu-mir-323	-1.28	-1.23	-1.35	-1.48	-1.50	-1.52	-1.54
4	Not Changed	mmu-mir-320	-0.17	-0.22	-0.29	-0.36	-0.20	-0.04	0.12
4	Not Changed	mmu-mir-32	0.02	0.05	0.02	-0.01	0.00	0.02	0.03
4	Not Changed	S-mmu-mir-30e	-0.03	0.01	0.00	-0.01	-0.05	-0.08	-0.12
4	Not Changed	mmu-mir-30e	-0.21	-0.26	-0.38	-0.49	-0.12	0.25	0.62
4	Not Changed	mmu-mir-30d	-0.70	-0.08	-0.41	-0.75	-0.63	-0.50	-0.38
4	Not Changed	mmu-mir-30c	-1.08	-0.41	-0.85	-1.29	-1.01	-0.73	-0.45
4	Not Changed	mmu-mir-30b	-1.11	-0.01	-0.46	-0.92	-0.58	-0.25	0.09
4	Not Changed	mmu-mir-30a-5p	-0.83	-0.43	-1.06	-1.69	-1.37	-1.05	-0.72
4	Not Changed	mmu-mir-30a-3p	-0.55	-0.45	-0.45	-0.44	-0.52	-0.60	-0.68
4	Not Changed	S-mmu-mir-302b	0.02	0.01	0.04	0.07	0.05	0.02	0.00
4	Not Changed	mmu-mir-301	-1.51	-1.16	-1.52	-1.88	-1.44	-1.00	-0.57
4	Not Changed	mmu-mir-29c	-0.03	-0.05	-0.10	-0.15	0.05	0.24	0.44
4	Not Changed	mmu-mir-29b	0.10	-0.01	-0.06	-0.10	0.33	0.77	1.21
4	Not Changed	mmu-mir-29a	0.37	0.41	-0.40	-1.20	-0.54	0.12	0.78
4	Not Changed	mmu-mir-298	-0.29	-0.22	-0.10	0.03	-0.57	-1.18	-1.78
4	Not Changed	mmu-mir-296	0.38	-0.27	0.14	0.56	0.06	-0.43	-0.92
4	Not Changed	mmu-mir-291b-3p	2.29	1.99	1.85	1.72	1.15	0.59	0.02
4	Not Changed	mmu-mir-28	-0.46	-0.39	-0.56	-0.72	-0.94	-1.16	-1.38
4	Not Changed	mmu-mir-27b	0.02	-0.03	-0.64	-1.24	-0.92	-0.60	-0.28
4	Not Changed	mmu-mir-27a	0.10	0.20	-0.52	-1.24	-0.98	-0.71	-0.45
4	Not Changed	mmu-mir-26b	-1.02	-0.44	-0.68	-0.92	-0.84	-0.75	-0.67
4	Not Changed	mmu-mir-26a	-1.48	-0.29	-0.50	-0.71	-0.72	-0.73	-0.75
4	Not Changed	mmu-mir-25	-0.21	-0.30	-0.72	-1.13	-1.20	-1.27	-1.34
4	Not Changed	mmu-mir-24	-0.31	0.15	-0.40	-0.94	-0.49	-0.03	0.42
4	Not Changed	mmu-mir-23b	-0.53	0.18	-0.24	-0.67	-0.45	-0.23	-0.01
4	Not Changed	mmu-mir-23a	-0.28	0.56	0.18	-0.19	-0.33	-0.48	-0.63
4	Not Changed	mmu-mir-224	-0.93	-0.44	-0.68	-0.92	-0.86	-0.80	-0.74
4	Not Changed	mmu-mir-223	0.19	-0.07	0.03	0.13	0.34	0.54	0.75
4	Not Changed	mmu-mir-222	-1.28	-0.85	-0.97	-1.09	-0.75	-0.40	-0.05
4	Not Changed	mmu-mir-221	-1.38	-1.03	-1.19	-1.35	-0.80	-0.25	0.29
4	Not Changed	mmu-mir-219	-0.18	-0.14	-0.16	-0.19	-0.09	0.01	0.10
4	Not Changed	mmu-mir-217	-0.20	-0.18	-0.17	-0.17	-0.15	-0.13	-0.12
4	Not Changed	mmu-mir-215	0.09	0.01	-0.01	-0.04	0.01	0.06	0.11
4	Not Changed	mmu-mir-213	-0.08	-0.06	-0.06	-0.07	-0.04	-0.01	0.01
4	Not Changed	mmu-mir-212	-0.29	-0.28	-0.08	0.11	0.03	-0.06	-0.15

Class	GCNF -/- Time Pattern	ProbeID	GCNF-Day0	GCNF-Day1	GCNF-Day2	GCNF-Day3	GCNF-Day4	GCNF-Day5	GCNF-Day6
4	Not Changed	mmu-mir-211	0.37	0.28	0.09	-0.10	-0.07	-0.03	0.00
4	Not Changed	mmu-mir-210	0.11	0.12	-0.68	-1.49	-0.92	-0.35	0.22
4	Not Changed	mmu-mir-21	-1.72	-1.55	-1.82	-2.08	-2.08	-2.07	-2.07
4	Not Changed	mmu-mir-208	0.02	0.02	0.03	0.04	0.03	0.02	0.01
4	Not Changed	mmu-mir-206	-0.01	0.01	-0.02	-0.05	0.40	0.85	1.29
4	Not Changed	mmu-mir-204	0.06	0.10	-0.01	-0.11	-0.03	0.05	0.13
4	Not Changed	mmu-mir-203	-0.08	0.23	0.02	-0.19	0.11	0.41	0.71
4	Not Changed	mmu-mir-202	0.06	-0.04	0.04	0.12	0.08	0.04	-0.01
4	Not Changed	mmu-mir-201	0.06	0.01	0.03	0.04	0.17	0.29	0.41
4	Not Changed	mmu-mir-200c	0.75	1.13	0.84	0.55	0.89	1.22	1.56
4	Not Changed	mmu-mir-19b	-0.06	-1.60	-2.78	-3.96	-3.57	-3.19	-2.80
4	Not Changed	mmu-mir-19a	-0.14	-1.21	-1.72	-2.23	-2.19	-2.14	-2.10
4	Not Changed	mmu-mir-196b	-0.02	-0.02	0.01	0.04	-0.01	-0.07	-0.12
4	Not Changed	mmu-mir-196a	-0.06	-0.01	0.11	0.23	0.13	0.03	-0.08
4	Not Changed	mmu-mir-194	-0.04	-0.06	-0.13	-0.21	0.08	0.37	0.65
4	Not Changed	mmu-mir-193	-0.05	-0.04	-0.05	-0.05	-0.13	-0.20	-0.27
4	Not Changed	mmu-mir-192	0.02	0.01	-0.01	-0.02	0.06	0.14	0.22
4	Not Changed	mmu-mir-191	-0.68	-0.39	-0.69	-0.99	-0.78	-0.56	-0.34
4	Not Changed	mmu-mir-190	0.03	0.03	0.00	-0.03	-0.03	-0.02	-0.02
4	Not Changed	mmu-mir-189	0.00	0.05	0.06	0.07	0.03	0.00	-0.04
4	Not Changed	mmu-mir-187	-0.07	0.05	-0.07	-0.20	-0.29	-0.37	-0.46
4	Not Changed	mmu-mir-186	-0.07	-0.50	-0.71	-0.92	-0.94	-0.96	-0.99
4	Not Changed	mmu-mir-185	-0.33	-0.04	-0.29	-0.54	-0.75	-0.96	-1.17
4	Not Changed	mmu-mir-184	-0.77	-0.64	-0.62	-0.59	-0.72	-0.86	-0.99
4	Not Changed	mmu-mir-181c	-0.65	-0.53	-0.61	-0.68	-0.69	-0.70	-0.70
4	Not Changed	mmu-mir-17-3p	0.26	0.40	-0.24	-0.88	-0.91	-0.94	-0.97
4	Not Changed	mmu-mir-16	-1.09	-0.99	-1.55	-2.11	-1.52	-0.94	-0.36
4	Not Changed	mmu-mir-15b	-0.40	-0.19	-0.48	-0.76	-0.57	-0.37	-0.18
4	Not Changed	mmu-mir-15a	-0.87	-1.41	-1.68	-1.95	-1.59	-1.22	-0.86
4	Not Changed	mmu-mir-155	-0.12	1.42	0.45	-0.51	0.06	0.63	1.20
4	Not Changed	mmu-mir-154	-1.62	-1.81	-1.96	-2.11	-2.04	-1.98	-1.91
4	Not Changed	mmu-mir-153	-0.03	0.01	-0.04	-0.10	-0.05	0.00	0.04
4	Not Changed	mmu-mir-152	-1.11	-0.72	-1.57	-2.42	-1.81	-1.21	-0.60
4	Not Changed	mmu-mir-151	-0.07	0.30	-0.18	-0.65	-0.73	-0.82	-0.90
4	Not Changed	mmu-mir-149	-0.11	0.34	-0.12	-0.57	-0.65	-0.74	-0.82
4	Not Changed	mmu-mir-146	-0.46	-0.13	-0.35	-0.57	-0.13	0.30	0.73
4	Not Changed	mmu-mir-145	-0.56	-0.31	-0.68	-1.05	-0.62	-0.20	0.22
4	Not Changed	mmu-mir-144	-0.01	0.01	-0.02	-0.05	0.02	0.08	0.14

Class	GCNF -/- Time Pattern	ProbeID	GCNF-Day0	GCNF-Day1	GCNF-Day2	GCNF-Day3	GCNF-Day4	GCNF-Day5	GCNF-Day6
4	Not Changed	mmu-mir-143	-0.32	-0.38	-0.91	-1.44	-0.77	-0.10	0.57
4	Not Changed	mmu-mir-142-5p	0.08	0.10	0.05	0.00	0.08	0.15	0.23
4	Not Changed	mmu-mir-142-3p	0.03	0.04	0.01	-0.02	0.04	0.09	0.15
4	Not Changed	mmu-mir-141	0.02	0.05	0.01	-0.02	0.05	0.12	0.19
4	Not Changed	S-mmu-mir-140	-0.26	-0.06	-0.76	-1.47	-0.72	0.02	0.77
4	Not Changed	mmu-mir-140	-0.01	-0.02	-0.02	-0.02	0.03	0.09	0.14
4	Not Changed	mmu-mir-139	-0.01	-0.05	-0.03	-0.01	0.02	0.06	0.09
4	Not Changed	mmu-mir-137	0.02	0.01	0.00	-0.02	0.07	0.16	0.25
4	Not Changed	mmu-mir-136	0.00	0.02	0.01	0.01	0.03	0.06	0.08
4	Not Changed	mmu-mir-135b	-0.03	0.03	-0.01	-0.04	-0.01	0.02	0.05
4	Not Changed	mmu-mir-135a	-0.03	0.01	-0.02	-0.04	-0.03	-0.01	0.00
4	Not Changed	mmu-mir-133b	0.19	-0.04	0.12	0.29	0.34	0.38	0.43
4	Not Changed	mmu-mir-133a	0.21	-0.06	0.14	0.35	0.41	0.48	0.54
4	Not Changed	mmu-mir-130b	0.20	-0.16	-0.96	-1.76	-1.41	-1.05	-0.70
4	Not Changed	mmu-mir-130a	0.00	-0.26	-0.64	-1.03	-0.68	-0.33	0.02
4	Not Changed	mmu-mir-129-3p	-0.13	-0.19	-0.15	-0.10	-0.08	-0.06	-0.04
4	Not Changed	mmu-mir-128b	-0.05	0.16	0.29	0.42	0.45	0.48	0.52
4	Not Changed	mmu-mir-128a	0.06	0.25	0.32	0.39	0.49	0.58	0.68
4	Not Changed	mmu-mir-127	-2.32	-2.59	-3.20	-3.81	-3.50	-3.19	-2.88
4	Not Changed	mmu-mir-126-5p	0.00	0.03	-0.01	-0.05	-0.01	0.03	0.07
4	Not Changed	mmu-mir-126-3p	-0.25	-0.43	-0.99	-1.56	-0.99	-0.41	0.16
4	Not Changed	mmu-mir-124a	-0.29	-0.33	-0.87	-1.40	-1.08	-0.77	-0.45
4	Not Changed	mmu-mir-122a	0.11	-0.71	-0.76	-0.80	-0.07	0.67	1.40
4	Not Changed	mmu-mir-107	-0.83	-0.60	-1.00	-1.41	-1.15	-0.90	-0.64
4	Not Changed	mmu-mir-106b	-0.12	-0.42	-0.91	-1.40	-1.23	-1.06	-0.88
4	Not Changed	mmu-mir-106a	0.50	0.63	0.08	-0.48	-0.67	-0.85	-1.04
4	Not Changed	mmu-mir-103	-0.90	-0.79	-1.25	-1.71	-1.34	-0.97	-0.61
4	Not Changed	mmu-mir-1	0.16	0.00	0.00	-0.01	0.59	1.20	1.80
4	Not Changed	mmu-let-7g	-0.63	-0.04	-0.40	-0.75	-0.03	0.70	1.42
4	Not Changed	S-mmu-let-7d	0.55	0.54	0.37	0.19	0.05	-0.08	-0.22
1	Changed	MIR70	2.79	2.86	1.81	0.75	-0.51	-1.77	-3.03
1	Changed	MIR43	-0.15	-0.10	-0.28	-0.47	-0.42	-0.37	-0.32
1	Not Changed	MIR4	-0.18	-0.03	-0.20	-0.37	-0.33	-0.30	-0.27
1	Not Changed	MIR30	0.45	0.37	0.41	0.44	-0.10	-0.65	-1.19
1	Not Changed	MIR253	0.26	-0.31	-0.98	-1.64	-1.73	-1.81	-1.89
1	Not Changed	MIR216	0.72	0.64	-0.01	-0.66	-0.90	-1.15	-1.39
1	Not Changed	MIR136	0.31	0.30	-0.06	-0.43	-0.63	-0.83	-1.03
3	Changed	MIR77	-2.27	-2.21	-2.08	-1.96	-1.69	-1.43	-1.16

Class	GCNF -/- Time Pattern	ProbeID	GCNF-Day0	GCNF-Day1	GCNF-Day2	GCNF-Day3	GCNF-Day4	GCNF-Day5	GCNF-Day6
3	Changed	MIR75	-2.35	-1.77	-1.39	-1.01	-0.26	0.49	1.24
3	Changed	MIR35	-2.57	-2.66	-2.64	-2.61	-1.77	-0.93	-0.08
3	Changed	MIR141	-0.63	-0.62	-0.39	-0.17	-0.21	-0.24	-0.28
4	Changed	MIR79	0.04	0.57	0.70	0.84	1.75	2.67	3.58
4	Not Changed	MIR88	-0.02	-0.17	-0.36	-0.54	-0.22	0.11	0.44
4	Not Changed	MIR85	-0.38	-0.11	-0.94	-1.77	-1.34	-0.92	-0.49
4	Not Changed	MIR74	-0.96	-0.74	-1.25	-1.76	-1.44	-1.13	-0.81
4	Not Changed	MIR71	-0.02	0.01	0.00	-0.01	0.01	0.03	0.05
4	Not Changed	MIR61	0.06	0.75	0.87	1.00	0.26	-0.48	-1.22
4	Not Changed	MIR52	-0.06	0.02	0.05	0.08	0.03	-0.02	-0.06
4	Not Changed	MIR47	-0.13	-0.01	-0.05	-0.08	-0.10	-0.12	-0.14
4	Not Changed	MIR41	-0.34	-0.19	-0.22	-0.25	-0.23	-0.20	-0.17
4	Not Changed	MIR257	0.42	1.14	1.02	0.90	0.19	-0.53	-1.24
4	Not Changed	MIR255	0.01	0.07	0.06	0.05	0.28	0.50	0.73
4	Not Changed	MIR237	0.08	-0.08	0.03	0.13	0.19	0.24	0.30
4	Not Changed	MIR220	0.02	-0.11	-0.07	-0.03	0.06	0.14	0.23
4	Not Changed	MIR207	0.32	-0.47	-0.33	-0.20	-0.28	-0.35	-0.43
4	Not Changed	MIR206	-0.34	0.75	0.16	-0.42	0.42	1.26	2.10
4	Not Changed	MIR202	-2.23	-2.11	-2.45	-2.79	-2.86	-2.93	-3.00
4	Not Changed	MIR201	0.00	-0.14	-0.09	-0.03	-0.02	0.00	0.02
4	Not Changed	MIR188	0.03	-0.07	-0.04	0.00	0.08	0.15	0.23
4	Not Changed	MIR184	-0.03	-0.17	-0.06	0.05	0.15	0.25	0.35
4	Not Changed	MIR180	0.05	-0.04	0.02	0.07	0.13	0.19	0.25
4	Not Changed	MIR177	-0.07	-0.13	-0.13	-0.12	-0.08	-0.03	0.02
4	Not Changed	MIR169	0.10	-0.09	-0.04	0.02	0.08	0.14	0.20
4	Not Changed	MIR167	0.00	-0.15	-0.02	0.10	0.11	0.11	0.12
4	Not Changed	MIR166	0.41	0.54	0.26	-0.02	0.68	1.37	2.07
4	Not Changed	MIR165	0.22	0.51	0.26	0.02	0.28	0.54	0.80
4	Not Changed	MIR161	-0.04	-0.08	-0.05	-0.02	0.05	0.12	0.19
4	Not Changed	MIR140	-0.02	-0.03	0.00	0.03	0.08	0.12	0.16
4	Not Changed	MIR124	-2.67	-2.44	-2.89	-3.35	-3.17	-3.00	-2.82
4	Not Changed	MIR122	-0.09	-0.09	-0.05	-0.01	-0.01	-0.02	-0.02
4	Not Changed	MIR121	-0.60	-0.31	-0.52	-0.74	-0.52	-0.31	-0.10
4	Not Changed	MIR12	-0.11	-0.29	-0.25	-0.22	0.00	0.21	0.43
4	Not Changed	MIR112	0.25	0.09	-0.05	-0.20	-0.04	0.11	0.27
4	Not Changed	MIR103	-0.03	-0.03	-0.15	-0.28	0.16	0.59	1.03
4	Not Changed	MIR102	-0.14	-0.02	0.17	0.35	0.19	0.03	-0.13
4	Not Changed	MIR100	-0.04	-0.04	0.01	0.06	0.07	0.07	0.08

Class	GCNF -/- Time Pattern	ProbeID	GCNF-Day0	GCNF-Day1	GCNF-Day2	GCNF-Day3	GCNF-Day4	GCNF-Day5	GCNF-Day6
1	Changed	cand97	0.09	-0.62	-0.88	-1.14	-1.06	-0.98	-0.90
1	Changed	cand78:a	-0.12	-0.62	-0.88	-1.15	-1.10	-1.06	-1.01
1	Changed	cand647	0.68	0.39	-0.19	-0.78	-0.52	-0.26	0.00
1	Changed	cand465:a	2.29	2.16	1.46	0.76	-0.28	-1.33	-2.38
1	Changed	cand324:b	-0.14	-0.12	-0.17	-0.22	-0.14	-0.06	0.01
1	Changed	cand317	0.52	0.66	0.35	0.04	0.37	0.70	1.04
1	Changed	cand268:b	1.24	1.25	0.45	-0.36	0.40	1.16	1.92
1	Changed	cand135:a	-0.24	-0.25	-0.42	-0.60	-0.45	-0.30	-0.15
1	Changed	cand1:b	-0.39	-0.36	-0.61	-0.85	-0.39	0.07	0.53
1	Not Changed	cand563	0.65	-0.15	0.71	1.56	0.31	-0.94	-2.19
1	Not Changed	cand467	1.05	0.30	0.10	-0.10	-0.91	-1.71	-2.52
1	Not Changed	cand465:b	0.43	0.53	0.14	-0.25	-0.28	-0.32	-0.35
1	Not Changed	cand342:a	0.35	0.27	0.11	-0.05	-0.11	-0.16	-0.21
1	Not Changed	cand324:a	0.93	0.89	0.36	-0.17	-0.46	-0.74	-1.03
1	Not Changed	cand276:b	0.12	0.24	0.02	-0.19	-0.17	-0.15	-0.13
1	Not Changed	cand172:b	0.18	0.13	-0.01	-0.16	-0.37	-0.59	-0.80
1	Not Changed	cand156	0.29	-0.43	0.79	2.01	0.97	-0.06	-1.10
1	Not Changed	cand144:a	0.31	0.20	-0.19	-0.59	-0.69	-0.79	-0.89
1	Not Changed	cand1:a	-0.44	-0.13	-0.26	-0.40	-0.41	-0.43	-0.44
2	Changed	cand79	0.01	-0.14	-0.22	-0.30	-0.11	0.08	0.27
2	Changed	cand284:b	-0.34	0.45	0.22	0.00	0.24	0.47	0.71
2	Changed	cand255	-0.21	-0.72	-0.70	-0.67	-0.58	-0.50	-0.41
2	Not Changed	cand595:a	0.08	0.22	0.38	0.54	-0.11	-0.75	-1.40
2	Not Changed	cand342:b	0.16	0.75	1.04	1.32	0.67	0.01	-0.64
2	Not Changed	cand149:b	0.44	0.29	0.91	1.53	0.34	-0.85	-2.05
3	Changed	cand90:b	-1.64	-1.31	-1.48	-1.66	-1.52	-1.39	-1.26
3	Changed	cand90:a	-3.11	-2.08	-2.57	-3.06	-2.30	-1.54	-0.78
3	Changed	cand516	0.02	-0.11	-0.07	-0.02	0.00	0.02	0.04
3	Changed	cand375	-3.97	-3.66	-3.56	-3.46	-2.72	-1.97	-1.23
3	Changed	cand306	-0.33	-0.60	-0.45	-0.31	-0.35	-0.40	-0.44
3	Changed	cand278:a	-2.40	-1.85	-2.22	-2.58	-2.16	-1.74	-1.32
3	Changed	cand27	-0.86	-0.73	-0.74	-0.75	-0.69	-0.64	-0.58
3	Not Changed	cand709	-1.48	-0.50	-0.32	-0.13	0.39	0.90	1.42
3	Not Changed	cand618	0.15	0.58	0.32	0.05	0.78	1.51	2.24
3	Not Changed	cand564:a	-0.75	-0.76	-0.82	-0.88	-0.82	-0.76	-0.70
3	Not Changed	cand490	-1.48	-0.44	-0.30	-0.17	0.30	0.78	1.25
3	Not Changed	cand374:a	-2.76	-2.38	-1.97	-1.56	-0.86	-0.16	0.53
3	Not Changed	cand371:a	-0.10	0.03	-0.14	-0.32	0.08	0.48	0.88

Class	GCNF -/- Time Pattern	ProbeID	GCNF-Day0	GCNF-Day1	GCNF-Day2	GCNF-Day3	GCNF-Day4	GCNF-Day5	GCNF-Day6
3	Not Changed	cand268:a	-1.04	0.82	0.18	-0.47	0.59	1.66	2.72
3	Not Changed	cand231	-0.16	-0.13	-0.10	-0.08	0.49	1.06	1.63
3	Not Changed	cand161	-1.61	-0.41	-0.18	0.05	0.62	1.19	1.76
3	Not Changed	cand119	-1.42	-0.53	-0.34	-0.14	0.44	1.01	1.59
3	Not Changed	cand118	-1.80	-0.57	-0.23	0.10	0.59	1.08	1.58
3	Not Changed	cand106:a	-1.19	-0.56	-0.20	0.17	0.67	1.17	1.66
4	Changed	cand64	0.00	0.51	0.17	-0.16	0.81	1.78	2.76
4	Changed	cand594	0.01	0.00	-0.20	-0.41	-0.15	0.11	0.37
4	Changed	cand501	0.47	0.17	-0.10	-0.38	0.39	1.17	1.94
4	Changed	cand500:a	-0.30	-0.25	-0.34	-0.43	-0.18	0.07	0.32
4	Changed	cand497:a	0.30	0.45	0.09	-0.27	0.49	1.25	2.01
4	Changed	cand457:b	0.36	0.83	1.74	2.64	2.09	1.53	0.98
4	Changed	cand427	-0.09	-0.03	0.28	0.60	0.40	0.21	0.02
4	Changed	cand361	0.08	0.01	0.17	0.32	0.58	0.83	1.09
4	Changed	cand349:a	-0.20	-0.36	0.10	0.56	0.34	0.12	-0.10
4	Changed	cand346	-0.81	-0.30	0.51	1.32	0.55	-0.22	-0.98
4	Changed	cand309	0.42	0.80	0.38	-0.04	1.09	2.21	3.33
4	Changed	cand279:b	0.14	0.23	-0.01	-0.25	0.06	0.37	0.68
4	Changed	cand262	-0.19	-0.19	-0.18	-0.18	0.02	0.21	0.40
4	Changed	cand25	0.00	0.09	0.05	0.01	0.31	0.60	0.89
4	Changed	cand226	-0.05	-0.03	0.41	0.85	0.77	0.69	0.61
4	Changed	cand203	-0.46	-0.74	-0.76	-0.78	-0.58	-0.38	-0.19
4	Changed	cand153:b	0.00	0.05	0.05	0.05	0.08	0.10	0.12
4	Changed	cand149:a	0.18	0.28	0.95	1.62	1.48	1.34	1.20
4	Changed	cand146	-0.01	0.64	0.30	-0.05	1.10	2.25	3.39
4	Changed	cand120	0.06	0.25	0.56	0.86	0.78	0.70	0.62
4	Changed	cand11:b	-0.19	-0.13	-0.38	-0.63	-0.36	-0.08	0.20
4	Not Changed	cand91:b	0.03	-0.06	0.09	0.24	0.16	0.07	-0.01
4	Not Changed	cand91:a	0.73	0.41	0.09	-0.22	0.51	1.24	1.97
4	Not Changed	cand82	0.02	-0.04	-0.05	-0.06	-0.01	0.04	0.09
4	Not Changed	cand78:b	0.28	0.02	-0.05	-0.12	0.09	0.29	0.50
4	Not Changed	cand73	1.79	0.31	0.22	0.13	-0.29	-0.72	-1.15
4	Not Changed	cand718:b	-0.28	0.69	0.46	0.24	0.57	0.90	1.23
4	Not Changed	cand718:a	0.38	0.17	0.15	0.14	0.09	0.04	0.00
4	Not Changed	cand708:b	0.74	0.57	0.71	0.85	0.59	0.33	0.07
4	Not Changed	cand708:a	-0.07	-0.07	-0.02	0.03	0.00	-0.03	-0.06
4	Not Changed	cand707	-0.46	-0.38	-0.49	-0.60	-0.52	-0.44	-0.36
4	Not Changed	cand706	0.01	-0.15	-0.04	0.07	-0.07	-0.21	-0.35

Class	GCNF -/- Time Pattern	ProbeID	GCNF-Day0	GCNF-Day1	GCNF-Day2	GCNF-Day3	GCNF-Day4	GCNF-Day5	GCNF-Day6
4	Not Changed	cand705	0.21	0.20	0.69	1.17	0.94	0.70	0.47
4	Not Changed	cand70	-0.15	0.03	0.11	0.19	0.06	-0.06	-0.18
4	Not Changed	cand7	-0.73	-0.61	0.58	1.77	0.88	-0.02	-0.91
4	Not Changed	cand699	0.34	0.79	0.41	0.02	0.53	1.04	1.55
4	Not Changed	cand692:b	0.11	-0.12	-0.15	-0.19	0.01	0.20	0.40
4	Not Changed	cand692:a	0.32	0.14	-0.39	-0.92	-0.16	0.60	1.35
4	Not Changed	cand690	-0.41	0.55	1.75	2.95	2.03	1.12	0.21
4	Not Changed	cand686	0.26	1.89	1.81	1.73	2.00	2.26	2.53
4	Not Changed	cand68	-0.49	0.69	0.60	0.51	0.92	1.33	1.75
4	Not Changed	cand678	0.07	0.36	-0.18	-0.72	-0.58	-0.44	-0.30
4	Not Changed	cand669	0.06	0.24	0.23	0.23	0.29	0.36	0.42
4	Not Changed	cand667:b	-0.22	0.09	0.04	-0.02	-0.07	-0.11	-0.15
4	Not Changed	cand667:a	0.27	-0.12	-0.01	0.10	0.04	-0.01	-0.06
4	Not Changed	cand664	0.05	-0.04	0.00	0.05	-0.05	-0.16	-0.26
4	Not Changed	cand650	0.14	0.19	0.22	0.25	0.16	0.07	-0.02
4	Not Changed	cand65	0.54	0.49	0.10	-0.29	0.67	1.63	2.59
4	Not Changed	cand648	0.24	-0.53	0.15	0.83	-0.28	-1.39	-2.50
4	Not Changed	cand624	0.70	-0.03	-0.37	-0.70	-0.14	0.43	0.99
4	Not Changed	cand619	-0.02	0.47	0.45	0.43	0.30	0.18	0.06
4	Not Changed	cand617	0.52	-0.10	0.53	1.17	0.91	0.65	0.40
4	Not Changed	cand616:b	0.39	0.39	0.37	0.34	0.48	0.62	0.75
4	Not Changed	cand616:a	-0.44	-0.59	-0.86	-1.14	-0.51	0.12	0.74
4	Not Changed	cand614	0.10	0.00	-0.06	-0.11	0.44	1.00	1.56
4	Not Changed	cand613	0.00	0.24	0.24	0.24	0.40	0.55	0.71
4	Not Changed	cand6	0.04	-0.04	-0.03	-0.03	0.22	0.47	0.73
4	Not Changed	cand595:b	0.03	-0.30	-0.20	-0.11	-0.29	-0.46	-0.64
4	Not Changed	cand590	-0.01	0.04	-0.04	-0.13	-0.07	-0.01	0.05
4	Not Changed	cand588	0.91	1.24	0.67	0.10	0.73	1.35	1.98
4	Not Changed	cand585	0.12	0.25	0.13	0.01	0.03	0.06	0.08
4	Not Changed	cand572:b	0.11	0.77	0.52	0.27	0.64	1.00	1.37
4	Not Changed	cand572:a	0.51	0.07	-0.40	-0.87	-0.44	0.00	0.43
4	Not Changed	cand570	0.46	0.59	1.34	2.09	1.03	-0.03	-1.09
4	Not Changed	cand57	-0.26	-0.79	-0.14	0.51	-0.17	-0.84	-1.52
4	Not Changed	cand564:b	-2.05	-2.21	-2.85	-3.50	-3.21	-2.93	-2.64
4	Not Changed	cand557	0.52	0.84	0.59	0.33	1.28	2.24	3.19
4	Not Changed	cand549	-0.05	-0.04	-0.05	-0.05	0.00	0.05	0.10
4	Not Changed	cand545	0.59	0.50	0.43	0.37	0.45	0.54	0.62
4	Not Changed	cand541	0.10	0.55	0.57	0.58	0.89	1.20	1.51

Class	GCNF -/- Time Pattern	ProbeID	GCNF-Day0	GCNF-Day1	GCNF-Day2	GCNF-Day3	GCNF-Day4	GCNF-Day5	GCNF-Day6
4	Not Changed	cand532:b	0.84	0.17	0.72	1.28	0.60	-0.07	-0.75
4	Not Changed	cand532:a	0.30	0.51	-0.14	-0.79	0.05	0.90	1.75
4	Not Changed	cand529	0.02	0.12	-0.05	-0.23	0.17	0.58	0.98
4	Not Changed	cand524	0.01	0.05	0.02	-0.01	0.04	0.08	0.13
4	Not Changed	cand523	0.25	0.17	0.58	1.00	0.50	0.01	-0.48
4	Not Changed	cand515	0.96	0.36	-0.11	-0.59	0.25	1.08	1.91
4	Not Changed	cand500:b	-0.04	0.18	0.10	0.02	0.05	0.07	0.10
4	Not Changed	cand50	-0.05	0.14	0.02	-0.10	-0.03	0.05	0.12
4	Not Changed	cand5	0.06	0.47	-0.17	-0.82	0.32	1.46	2.60
4	Not Changed	cand497:b	0.01	0.00	-0.07	-0.14	0.00	0.14	0.29
4	Not Changed	cand492	-0.55	-0.58	1.15	2.88	1.70	0.53	-0.64
4	Not Changed	cand489	-0.22	0.15	-0.15	-0.45	-0.14	0.18	0.49
4	Not Changed	cand462	0.26	0.17	1.48	2.80	1.63	0.47	-0.69
4	Not Changed	cand459	0.20	0.04	-0.09	-0.23	0.52	1.27	2.02
4	Not Changed	cand457:a	0.76	0.21	0.57	0.93	0.37	-0.20	-0.76
4	Not Changed	cand445	0.72	0.29	0.37	0.45	0.20	-0.05	-0.30
4	Not Changed	cand425	0.77	0.25	0.37	0.49	0.41	0.32	0.23
4	Not Changed	cand42:b	0.99	0.86	0.56	0.26	0.93	1.61	2.29
4	Not Changed	cand42:a	0.84	1.27	1.38	1.50	1.38	1.27	1.16
4	Not Changed	cand418	0.91	0.21	0.33	0.46	0.00	-0.46	-0.92
4	Not Changed	cand415	0.09	0.22	0.23	0.24	0.78	1.33	1.87
4	Not Changed	cand412	0.19	-0.26	0.89	2.03	1.34	0.64	-0.05
4	Not Changed	cand40	0.03	0.11	0.04	-0.03	0.08	0.20	0.32
4	Not Changed	cand386:b	0.48	0.14	0.43	0.72	0.45	0.17	-0.10
4	Not Changed	cand386:a	0.71	0.66	0.96	1.27	1.21	1.15	1.08
4	Not Changed	cand385	0.11	0.02	-0.04	-0.09	0.04	0.18	0.32
4	Not Changed	cand374:b	0.29	0.77	0.79	0.81	0.99	1.17	1.35
4	Not Changed	cand371:b	0.17	0.46	0.20	-0.07	0.15	0.37	0.60
4	Not Changed	cand362	0.57	-0.67	-0.22	0.24	-0.70	-1.65	-2.59
4	Not Changed	cand352:b	-0.17	-0.25	-0.22	-0.19	-0.12	-0.06	0.01
4	Not Changed	cand352:a	-0.04	-0.09	-0.11	-0.13	0.00	0.12	0.25
4	Not Changed	cand351	-0.16	0.05	0.17	0.28	0.15	0.01	-0.13
4	Not Changed	cand350	0.64	1.11	0.43	-0.26	-0.50	-0.75	-1.00
4	Not Changed	cand35	0.11	-0.03	0.15	0.34	0.24	0.14	0.04
4	Not Changed	cand349:b	0.04	-0.07	-0.06	-0.06	0.01	0.08	0.14
4	Not Changed	cand348	-0.70	-0.24	-0.38	-0.52	-0.06	0.40	0.86
4	Not Changed	cand347	-0.11	-0.14	-0.44	-0.74	-0.23	0.28	0.78
4	Not Changed	cand345	1.81	0.66	0.74	0.83	-0.21	-1.25	-2.29

Class	GCNF -/- Time Pattern	ProbeID	GCNF-Day0	GCNF-Day1	GCNF-Day2	GCNF-Day3	GCNF-Day4	GCNF-Day5	GCNF-Day6
4	Not Changed	cand341	0.21	-1.04	-2.22	-3.39	-3.23	-3.07	-2.91
4	Not Changed	cand340	0.88	0.38	0.40	0.42	0.71	1.01	1.30
4	Not Changed	cand337	-1.07	-0.51	-1.14	-1.78	-1.38	-0.99	-0.59
4	Not Changed	cand336	-1.08	-0.85	-1.22	-1.59	-1.14	-0.69	-0.24
4	Not Changed	cand334:b	1.05	0.33	0.09	-0.14	0.18	0.50	0.83
4	Not Changed	cand334:a	0.07	-0.05	-0.02	0.01	-0.06	-0.14	-0.22
4	Not Changed	cand315:b	-0.43	0.08	-0.45	-0.97	-0.74	-0.51	-0.28
4	Not Changed	cand315:a	-0.02	0.18	-0.17	-0.53	-0.51	-0.50	-0.48
4	Not Changed	cand314	-0.77	-0.15	-0.62	-1.09	-0.69	-0.29	0.11
4	Not Changed	cand304	0.08	-0.27	-0.62	-0.97	-0.30	0.36	1.02
4	Not Changed	cand302:b	0.38	0.70	0.32	-0.07	0.37	0.81	1.25
4	Not Changed	cand302:a	0.15	-0.11	0.01	0.13	0.07	0.02	-0.03
4	Not Changed	cand297:b	0.38	0.43	-0.28	-1.00	-0.25	0.51	1.26
4	Not Changed	cand297:a	-0.10	-0.07	-0.06	-0.05	0.01	0.07	0.14
4	Not Changed	cand294	0.72	0.57	0.48	0.39	0.31	0.22	0.13
4	Not Changed	cand286	-0.24	0.07	-0.27	-0.61	0.04	0.68	1.32
4	Not Changed	cand284:a	-0.87	-0.15	-0.93	-1.72	-1.21	-0.71	-0.20
4	Not Changed	cand279:a	0.57	0.18	-0.10	-0.39	0.04	0.46	0.88
4	Not Changed	cand278:b	0.02	-0.01	0.09	0.19	0.14	0.08	0.03
4	Not Changed	cand276:a	0.38	0.73	0.16	-0.41	-0.21	-0.01	0.20
4	Not Changed	cand275	0.45	0.34	0.66	0.98	0.62	0.27	-0.08
4	Not Changed	cand271	0.16	-0.06	0.06	0.18	0.14	0.11	0.07
4	Not Changed	cand26	0.40	0.19	0.14	0.10	0.68	1.25	1.83
4	Not Changed	cand252	-0.27	-0.12	-0.67	-1.21	-1.09	-0.96	-0.84
4	Not Changed	cand244	0.58	-0.13	0.23	0.60	0.07	-0.47	-1.00
4	Not Changed	cand24	0.82	0.29	-0.01	-0.32	0.13	0.57	1.02
4	Not Changed	cand234	0.03	0.03	0.00	-0.02	0.07	0.17	0.26
4	Not Changed	cand224	0.19	0.05	0.11	0.17	0.21	0.25	0.29
4	Not Changed	cand22	0.07	0.04	0.02	0.00	0.07	0.13	0.19
4	Not Changed	cand213	-0.89	-0.84	-1.02	-1.19	-1.16	-1.12	-1.09
4	Not Changed	cand212	-1.15	-0.92	-1.13	-1.34	-1.31	-1.27	-1.23
4	Not Changed	cand210:b	-0.69	-0.73	-0.90	-1.07	-1.04	-1.01	-0.98
4	Not Changed	cand210:a	-1.80	-1.60	-1.92	-2.25	-2.28	-2.30	-2.33
4	Not Changed	cand202	-0.07	-0.06	-0.01	0.04	0.05	0.06	0.08
4	Not Changed	cand200	-1.35	-1.65	0.81	3.27	1.33	-0.61	-2.56
4	Not Changed	cand192	0.29	0.15	0.37	0.60	0.64	0.67	0.71
4	Not Changed	cand186	0.18	-0.47	-0.49	-0.51	-0.32	-0.14	0.05
4	Not Changed	cand184	0.00	-0.07	-0.13	-0.19	-0.10	-0.01	0.07

Class	GCNF -/- Time Pattern	ProbeID	GCNF-Day0	GCNF-Day1	GCNF-Day2	GCNF-Day3	GCNF-Day4	GCNF-Day5	GCNF-Day6
4	Not Changed	cand179:b	-0.01	-0.04	0.00	0.04	0.04	0.03	0.03
4	Not Changed	cand179:a	1.14	0.97	1.11	1.25	0.62	0.00	-0.63
4	Not Changed	cand178:b	-0.09	-0.10	0.00	0.09	0.05	0.02	-0.01
4	Not Changed	cand178:a	0.15	0.05	0.60	1.14	0.45	-0.24	-0.94
4	Not Changed	cand172:a	0.25	0.11	-0.08	-0.27	-0.27	-0.28	-0.29
4	Not Changed	cand157	-0.39	0.22	-0.13	-0.48	0.19	0.86	1.53
4	Not Changed	cand153:a	0.19	-0.20	-0.04	0.12	0.15	0.19	0.22
4	Not Changed	cand152:b	0.59	1.12	1.14	1.15	0.96	0.76	0.57
4	Not Changed	cand152:a	-0.01	0.14	0.60	1.05	1.16	1.26	1.37
4	Not Changed	cand151	-0.01	-0.19	-0.08	0.03	0.02	0.01	0.00
4	Not Changed	cand144:b	0.02	0.06	0.01	-0.05	-0.02	0.02	0.05
4	Not Changed	cand139	0.05	-0.01	-0.02	-0.02	0.04	0.11	0.17
4	Not Changed	cand137:b	-0.08	-0.07	-0.09	-0.12	-0.15	-0.18	-0.21
4	Not Changed	cand137:a	-0.70	-0.24	-1.29	-2.34	-1.64	-0.94	-0.24
4	Not Changed	cand135:b	-0.19	-0.15	-0.66	-1.17	-0.62	-0.07	0.48
4	Not Changed	cand130	0.00	-0.01	-0.02	-0.03	-0.01	0.00	0.02
4	Not Changed	cand13	0.33	-0.07	0.04	0.16	-0.06	-0.27	-0.48
4	Not Changed	cand129	0.04	0.00	-0.04	-0.07	-0.04	0.00	0.03
4	Not Changed	cand126	0.86	0.13	0.88	1.62	1.00	0.37	-0.25
4	Not Changed	cand116	0.12	0.47	0.34	0.22	0.21	0.21	0.21
4	Not Changed	cand115	-0.48	-0.84	1.04	2.91	1.53	0.15	-1.23
4	Not Changed	cand11:a	-0.02	0.05	-0.02	-0.09	-0.04	0.01	0.06
4	Not Changed	cand109	0.59	0.61	0.43	0.24	0.53	0.81	1.10
4	Not Changed	cand106:b	0.12	0.13	-0.03	-0.19	-0.09	0.02	0.12
4	Not Changed	cand104:b	0.21	0.33	-0.03	-0.39	-0.36	-0.32	-0.29
4	Not Changed	cand104:a	-0.05	0.26	-0.39	-1.04	-0.60	-0.16	0.28
4	Not Changed	cand103:b	-0.15	0.42	-0.01	-0.44	-0.41	-0.37	-0.34
4	Not Changed	cand103:a	0.11	0.51	0.11	-0.29	-0.30	-0.30	-0.31
4	Not Changed	cand100	0.24	0.35	0.25	0.15	0.11	0.08	0.04
1	Changed	S-mmu-mir-467	0.73	0.95	1.18	1.40	0.65	-0.11	-0.86
1	Changed	S-mmu-mir-425	-0.09	-0.21	-0.54	-0.88	-0.87	-0.86	-0.84
1	Changed	S-mmu-mir-295	1.67	1.55	1.01	0.47	0.45	0.43	0.41
1	Changed	S-mmu-mir-294	1.29	1.45	1.26	1.07	0.45	-0.17	-0.79
1	Changed	S-mmu-mir-210	0.47	0.52	0.84	1.16	1.07	0.98	0.89
1	Not Changed	S-mmu-mir-293	1.37	1.14	-0.13	-1.39	-1.30	-1.22	-1.13
1	Not Changed	S-mmu-mir-290	1.01	-0.01	0.52	1.05	0.59	0.13	-0.33
1	Not Changed	S-mmu-mir-27a	0.25	0.53	0.22	-0.10	-0.12	-0.14	-0.17
1	Not Changed	S-mmu-mir-25	1.14	0.60	0.66	0.72	0.23	-0.27	-0.76

Class	GCNF -/- Time Pattern	ProbeID	GCNF-Day0	GCNF-Day1	GCNF-Day2	GCNF-Day3	GCNF-Day4	GCNF-Day5	GCNF-Day6
1	Not Changed	S-mmu-mir-24-2	0.34	0.62	-0.01	-0.65	-0.52	-0.39	-0.26
1	Not Changed	S-mmu-mir-183	1.04	1.08	0.70	0.33	0.07	-0.18	-0.44
1	Not Changed	S-mmu-mir-181c	-0.04	0.03	-0.12	-0.27	-0.24	-0.21	-0.17
1	Not Changed	S-mmu-mir-18	0.10	-0.21	-0.32	-0.43	-0.26	-0.09	0.08
2	Not Changed	S-mmu-mir-21	0.40	0.73	0.51	0.28	0.45	0.61	0.77
3	Changed	S-mmu-mir-351	-0.46	-0.74	-0.69	-0.64	-0.48	-0.33	-0.17
3	Changed	S-mmu-mir-345	-0.17	-0.13	-0.30	-0.48	-0.51	-0.53	-0.56
3	Changed	S-mmu-mir-219-2	-1.62	-1.37	-1.42	-1.46	-1.45	-1.43	-1.41
3	Changed	S-mmu-mir-199b	-2.22	-2.15	-2.29	-2.42	-1.29	-0.16	0.97
3	Changed	S-mmu-mir-135a-2	-0.40	-0.71	-0.55	-0.39	-0.37	-0.34	-0.32
3	Changed	S-mmu-mir-125b-1	-0.24	-0.66	-0.10	0.47	0.26	0.06	-0.15
3	Changed	S-mmu-mir-10b	-0.51	-0.66	-0.75	-0.83	-0.57	-0.30	-0.03
3	Changed	S-mmu-mir-10a	-1.61	-1.61	-1.56	-1.52	-1.42	-1.31	-1.21
3	Not Changed	S-mmu-mir-412	-1.30	-1.17	-1.17	-1.17	-1.16	-1.15	-1.13
3	Not Changed	S-mmu-mir-181a	-0.27	-0.14	-0.12	-0.10	-0.02	0.07	0.16
4	Changed	S-mmu-mir-469	0.32	0.09	-0.03	-0.14	-0.15	-0.15	-0.16
4	Changed	S-mmu-mir-350	0.04	0.09	0.02	-0.05	0.04	0.14	0.23
4	Changed	S-mmu-mir-296	0.17	-0.34	-0.13	0.09	-0.25	-0.59	-0.92
4	Changed	S-mmu-mir-23a	0.83	0.89	1.16	1.43	0.79	0.15	-0.49
4	Changed	S-mmu-mir-223	0.12	0.00	0.02	0.04	0.12	0.21	0.29
4	Changed	S-mmu-mir-193	0.07	-0.20	0.01	0.23	0.01	-0.21	-0.43
4	Changed	S-mmu-let-7e	-0.09	0.07	0.38	0.69	0.53	0.38	0.23
4	Not Changed	S-mmu-mir-99b	-0.55	-0.08	-0.41	-0.74	-0.47	-0.20	0.07
4	Not Changed	S-mmu-mir-99a	0.04	-0.11	0.00	0.11	0.19	0.27	0.35
4	Not Changed	S-mmu-mir-98	0.03	-0.04	-0.04	-0.04	0.13	0.31	0.48
4	Not Changed	S-mmu-mir-96	0.15	0.03	0.02	0.01	0.07	0.14	0.20
4	Not Changed	S-mmu-mir-93	0.39	0.06	-0.31	-0.68	-0.47	-0.26	-0.06
4	Not Changed	S-mmu-mir-92-2	0.50	0.08	1.35	2.62	1.07	-0.47	-2.02
4	Not Changed	S-mmu-mir-92-1	0.27	-0.07	-0.04	0.00	0.03	0.06	0.08
4	Not Changed	S-mmu-mir-7b	0.80	0.60	0.35	0.10	0.44	0.78	1.12
4	Not Changed	S-mmu-mir-7-2	0.36	0.24	0.08	-0.07	0.12	0.31	0.51
4	Not Changed	S-mmu-mir-7-1	0.10	0.09	-0.09	-0.26	-0.03	0.21	0.44
4	Not Changed	S-mmu-mir-486	0.33	-0.34	0.25	0.85	0.50	0.16	-0.19
4	Not Changed	S-mmu-mir-484	0.11	0.12	-0.09	-0.30	0.02	0.34	0.66
4	Not Changed	S-mmu-mir-483	-0.09	0.20	0.37	0.54	0.93	1.31	1.70
4	Not Changed	S-mmu-mir-471	0.04	-0.06	-0.08	-0.09	0.01	0.11	0.21
4	Not Changed	S-mmu-mir-468	0.05	-0.06	-0.01	0.03	0.10	0.18	0.25
4	Not Changed	S-mmu-mir-466	0.11	0.53	0.39	0.26	0.26	0.27	0.27

Class	GCNF -/- Time Pattern	ProbeID	GCNF-Day0	GCNF-Day1	GCNF-Day2	GCNF-Day3	GCNF-Day4	GCNF-Day5	GCNF-Day6
4	Not Changed	S-mmu-mir-465	-0.77	-0.68	-0.68	-0.67	-0.62	-0.57	-0.52
4	Not Changed	S-mmu-mir-451	-0.01	0.15	0.07	-0.02	0.01	0.03	0.05
4	Not Changed	S-mmu-mir-449	0.09	-0.01	0.11	0.22	0.22	0.23	0.24
4	Not Changed	S-mmu-mir-448	0.05	0.00	0.01	0.03	0.02	0.01	-0.01
4	Not Changed	S-mmu-mir-431	-0.61	-0.24	-0.51	-0.77	-0.72	-0.66	-0.61
4	Not Changed	S-mmu-mir-409	-0.50	-0.53	-0.48	-0.44	-0.30	-0.16	-0.02
4	Not Changed	S-mmu-mir-384	-0.07	0.57	0.24	-0.08	0.40	0.89	1.37
4	Not Changed	S-mmu-mir-382	-0.64	-0.65	-0.68	-0.70	-0.63	-0.57	-0.50
4	Not Changed	S-mmu-mir-381	0.10	-0.01	-0.05	-0.08	-0.07	-0.06	-0.06
4	Not Changed	S-mmu-mir-379	-1.64	-1.64	-1.77	-1.90	-1.84	-1.78	-1.71
4	Not Changed	S-mmu-mir-378	0.38	-0.40	-1.09	-1.77	-1.05	-0.32	0.41
4	Not Changed	S-mmu-mir-377	-0.08	-0.27	-0.28	-0.30	-0.30	-0.30	-0.29
4	Not Changed	S-mmu-mir-370	-0.56	-0.48	-0.57	-0.65	-0.60	-0.55	-0.49
4	Not Changed	S-mmu-mir-365-2	0.65	0.52	0.66	0.80	0.54	0.28	0.02
4	Not Changed	S-mmu-mir-365-1	0.41	0.21	0.37	0.53	0.36	0.19	0.02
4	Not Changed	S-mmu-mir-363	1.09	0.38	0.69	1.00	0.31	-0.38	-1.06
4	Not Changed	S-mmu-mir-361	-0.07	-0.15	0.04	0.24	0.18	0.12	0.06
4	Not Changed	S-mmu-mir-34c	0.36	0.51	0.24	-0.02	0.19	0.40	0.60
4	Not Changed	S-mmu-mir-34b	-0.19	0.11	-0.16	-0.44	-0.20	0.03	0.27
4	Not Changed	S-mmu-mir-34a	0.35	0.04	0.07	0.10	0.14	0.18	0.22
4	Not Changed	S-mmu-mir-346	0.12	0.03	0.28	0.54	0.18	-0.17	-0.52
4	Not Changed	S-mmu-mir-342	-0.63	0.04	-0.26	-0.57	-0.40	-0.23	-0.05
4	Not Changed	S-mmu-mir-339	-0.01	-0.05	-0.11	-0.17	-0.03	0.11	0.24
4	Not Changed	S-mmu-mir-338	0.04	0.00	0.02	0.04	0.69	1.34	1.99
4	Not Changed	S-mmu-mir-337	-1.45	-1.36	-2.09	-2.82	-2.62	-2.43	-2.23
4	Not Changed	S-mmu-mir-335	0.02	0.02	-0.02	-0.07	-0.03	0.00	0.04
4	Not Changed	S-mmu-mir-331	0.04	0.03	0.17	0.30	0.20	0.09	-0.01
4	Not Changed	S-mmu-mir-330	-0.07	-0.14	-0.20	-0.27	-0.03	0.21	0.45
4	Not Changed	S-mmu-mir-33	0.07	-0.02	0.00	0.03	0.31	0.60	0.88
4	Not Changed	S-mmu-mir-329	-0.78	-1.08	-1.24	-1.39	-1.36	-1.34	-1.31
4	Not Changed	S-mmu-mir-328	0.22	0.04	0.37	0.71	0.38	0.04	-0.29
4	Not Changed	S-mmu-mir-326	0.31	-0.43	0.37	1.17	0.41	-0.35	-1.10
4	Not Changed	S-mmu-mir-323	-0.17	-0.38	0.24	0.86	0.52	0.18	-0.16
4	Not Changed	S-mmu-mir-32	0.22	0.38	0.42	0.46	0.46	0.47	0.47
4	Not Changed	S-mmu-mir-31	0.14	-0.02	-0.01	0.00	0.28	0.56	0.84
4	Not Changed	S-mmu-mir-30c-2	-0.01	-0.06	0.31	0.68	0.47	0.27	0.06
4	Not Changed	S-mmu-mir-30c-1	0.81	-1.77	0.00	1.77	0.00	-1.76	-3.52
4	Not Changed	S-mmu-mir-30b	0.10	-0.20	0.06	0.31	0.25	0.19	0.13

Class	GCNF -/- Time Pattern	ProbeID	GCNF-Day0	GCNF-Day1	GCNF-Day2	GCNF-Day3	GCNF-Day4	GCNF-Day5	GCNF-Day6
4	Not Changed	S-mmu-mir-300	-0.35	-0.60	-0.48	-0.36	-0.31	-0.26	-0.21
4	Not Changed	S-mmu-mir-29b-2	0.02	-0.02	0.01	0.03	0.08	0.13	0.19
4	Not Changed	S-mmu-mir-29b-1	0.19	-0.09	-0.01	0.06	0.13	0.21	0.29
4	Not Changed	S-mmu-mir-29a	0.07	-0.09	-0.06	-0.02	0.11	0.25	0.39
4	Not Changed	S-mmu-mir-298	0.04	-0.19	-0.14	-0.08	0.06	0.20	0.34
4	Not Changed	S-mmu-mir-28	-0.27	-0.08	-0.20	-0.32	-0.20	-0.09	0.03
4	Not Changed	S-mmu-mir-27b	0.22	0.07	0.00	-0.08	-0.04	0.00	0.04
4	Not Changed	S-mmu-mir-26a-1	0.16	0.02	0.01	0.01	0.14	0.27	0.39
4	Not Changed	S-mmu-mir-23b	0.40	0.52	0.20	-0.13	-0.17	-0.20	-0.24
4	Not Changed	S-mmu-mir-221	-0.02	-0.03	-0.05	-0.07	0.02	0.11	0.21
4	Not Changed	S-mmu-mir-22	-0.37	-0.46	-0.35	-0.23	-0.11	0.02	0.14
4	Not Changed	S-mmu-mir-218-2	0.39	0.47	0.38	0.30	0.32	0.35	0.38
4	Not Changed	S-mmu-mir-218-1	0.38	0.26	0.26	0.26	0.32	0.37	0.43
4	Not Changed	S-mmu-mir-214	-0.04	-0.13	-0.06	0.00	0.10	0.19	0.28
4	Not Changed	S-mmu-mir-212	-0.24	-0.21	0.21	0.63	0.42	0.21	0.00
4	Not Changed	S-mmu-mir-211	0.22	0.50	0.67	0.83	0.88	0.92	0.97
4	Not Changed	S-mmu-mir-208	0.73	-0.15	0.27	0.69	0.28	-0.14	-0.55
4	Not Changed	S-mmu-mir-207	0.26	0.25	0.34	0.42	0.21	0.00	-0.21
4	Not Changed	S-mmu-mir-204	0.80	0.36	1.33	2.29	1.41	0.53	-0.35
4	Not Changed	S-mmu-mir-200b	0.61	0.59	0.44	0.29	0.15	0.02	-0.12
4	Not Changed	S-mmu-mir-200a	0.25	0.21	0.18	0.15	0.12	0.10	0.08
4	Not Changed	S-mmu-mir-20	0.06	-0.06	-0.08	-0.11	0.03	0.16	0.30
4	Not Changed	S-mmu-mir-196b	0.38	0.23	0.18	0.13	0.27	0.42	0.57
4	Not Changed	S-mmu-mir-196a-2	0.08	0.06	0.00	-0.06	0.11	0.28	0.45
4	Not Changed	S-mmu-mir-196a-1	0.11	0.09	0.02	-0.05	0.06	0.17	0.27
4	Not Changed	S-mmu-mir-195	0.06	0.04	0.02	0.00	0.05	0.09	0.14
4	Not Changed	S-mmu-mir-194-2	0.43	0.01	0.79	1.57	0.81	0.05	-0.71
4	Not Changed	S-mmu-mir-194-1	0.03	0.04	0.09	0.14	0.14	0.15	0.15
4	Not Changed	S-mmu-mir-192	0.07	0.11	0.05	-0.01	0.17	0.36	0.54
4	Not Changed	S-mmu-mir-191	0.07	0.07	0.07	0.07	0.08	0.10	0.11
4	Not Changed	S-mmu-mir-190	0.06	0.08	0.02	-0.04	0.07	0.18	0.29
4	Not Changed	S-mmu-mir-188	0.04	-0.07	0.09	0.25	0.26	0.27	0.29
4	Not Changed	S-mmu-mir-187	0.53	0.38	0.25	0.13	0.56	1.00	1.44
4	Not Changed	S-mmu-mir-186	0.09	0.10	0.07	0.04	0.05	0.07	0.09
4	Not Changed	S-mmu-mir-185	0.03	-0.03	0.11	0.26	0.53	0.80	1.06
4	Not Changed	S-mmu-mir-184	0.59	0.46	0.18	-0.11	0.13	0.37	0.61
4	Not Changed	S-mmu-mir-182	0.11	0.05	0.00	-0.05	0.07	0.18	0.30
4	Not Changed	S-mmu-mir-16-2	0.13	-0.12	-0.09	-0.05	0.14	0.33	0.53

Class	GCNF -/- Time Pattern	ProbeID	GCNF-Day0	GCNF-Day1	GCNF-Day2	GCNF-Day3	GCNF-Day4	GCNF-Day5	GCNF-Day6
4	Not Changed	S-mmu-mir-16-1	0.47	-0.04	-0.03	-0.03	0.07	0.17	0.28
4	Not Changed	S-mmu-mir-15b	0.03	-0.16	-0.09	-0.02	0.15	0.32	0.49
4	Not Changed	S-mmu-mir-15a	0.21	0.21	0.06	-0.09	0.24	0.58	0.92
4	Not Changed	S-mmu-mir-154	-0.70	-0.90	-0.85	-0.80	-0.67	-0.54	-0.41
4	Not Changed	S-mmu-mir-151	-0.28	0.02	-0.39	-0.81	-0.67	-0.54	-0.40
4	Not Changed	S-mmu-mir-150	0.59	0.05	-0.05	-0.15	0.10	0.34	0.59
4	Not Changed	S-mmu-mir-149	0.42	-0.17	0.05	0.27	0.13	0.00	-0.14
4	Not Changed	S-mmu-mir-148b	0.03	-0.09	-0.07	-0.05	0.12	0.29	0.47
4	Not Changed	S-mmu-mir-148a	0.08	-0.15	-0.10	-0.05	0.08	0.21	0.34
4	Not Changed	S-mmu-mir-146	0.05	-0.10	-0.04	0.02	0.15	0.27	0.40
4	Not Changed	S-mmu-mir-145	0.00	-0.24	-0.10	0.04	0.11	0.18	0.25
4	Not Changed	S-mmu-mir-144	0.07	-0.12	-0.07	-0.02	0.12	0.27	0.41
4	Not Changed	S-mmu-mir-143	0.12	-0.10	-0.03	0.03	0.14	0.25	0.36
4	Not Changed	S-mmu-mir-141	0.06	-0.06	-0.03	0.00	0.15	0.30	0.44
4	Not Changed	S-mmu-mir-139	0.13	-0.12	0.01	0.13	0.11	0.10	0.08
4	Not Changed	S-mmu-mir-138-2	0.12	-0.12	0.03	0.19	0.21	0.24	0.26
4	Not Changed	S-mmu-mir-138-1	0.01	-0.15	-0.03	0.10	0.17	0.24	0.30
4	Not Changed	S-mmu-mir-137	0.10	-0.19	-0.05	0.08	0.09	0.11	0.12
4	Not Changed	S-mmu-mir-135a-1	0.16	-0.24	-0.14	-0.04	0.04	0.12	0.20
4	Not Changed	S-mmu-mir-134	-0.40	-0.68	-0.63	-0.58	-0.39	-0.21	-0.03
4	Not Changed	S-mmu-mir-133b	0.00	-0.28	-0.05	0.18	0.54	0.90	1.26
4	Not Changed	S-mmu-mir-133a-2	0.04	-0.13	-0.05	0.04	0.15	0.27	0.39
4	Not Changed	S-mmu-mir-133a-1	0.09	-0.10	-0.03	0.05	0.15	0.26	0.37
4	Not Changed	S-mmu-mir-132	0.04	-0.13	-0.06	0.01	0.11	0.21	0.31
4	Not Changed	S-mmu-mir-130a	0.03	-0.06	-0.03	0.01	0.07	0.14	0.20
4	Not Changed	S-mmu-mir-129-1	0.61	-0.20	-0.05	0.10	0.19	0.28	0.37
4	Not Changed	S-mmu-mir-128b	0.94	-0.59	1.14	2.88	1.03	-0.82	-2.67
4	Not Changed	S-mmu-mir-128a	0.06	-0.13	0.21	0.54	0.41	0.27	0.14
4	Not Changed	S-mmu-mir-127	-0.70	-0.88	-0.83	-0.78	-0.63	-0.49	-0.35
4	Not Changed	S-mmu-mir-125b-2	-0.09	-0.29	-0.19	-0.10	0.02	0.13	0.24
4	Not Changed	S-mmu-mir-125a	-0.36	0.09	0.02	-0.05	0.01	0.07	0.14
4	Not Changed	S-mmu-mir-124a-3	0.23	-0.06	-0.06	-0.06	0.03	0.12	0.22
4	Not Changed	S-mmu-mir-124a-2	0.34	-0.05	-0.04	-0.02	0.07	0.17	0.26
4	Not Changed	S-mmu-mir-124a-1	0.25	-0.10	-0.06	-0.02	0.60	1.21	1.82
4	Not Changed	S-mmu-mir-122a	0.12	0.07	0.00	-0.07	0.14	0.34	0.54
4	Not Changed	S-mmu-mir-1-2	0.11	-0.10	-0.08	-0.07	0.12	0.32	0.51
4	Not Changed	S-mmu-mir-1-1	0.85	0.40	-0.01	-0.42	-0.09	0.24	0.57
4	Not Changed	S-mmu-mir-107	0.25	-0.01	-0.08	-0.15	0.01	0.17	0.33

Class	GCNF -/- Time Pattern	ProbeID	GCNF-Day0	GCNF-Day1	GCNF-Day2	GCNF-Day3	GCNF-Day4	GCNF-Day5	GCNF-Day6
4	Not Changed	S-mmu-mir-106b	0.06	0.32	-0.17	-0.66	-0.49	-0.33	-0.16
4	Not Changed	S-mmu-mir-106a	0.19	0.30	0.17	0.03	0.03	0.04	0.04
4	Not Changed	S-mmu-mir-103-2	-0.01	0.01	0.01	0.01	0.09	0.17	0.25
4	Not Changed	S-mmu-mir-103-1	0.21	0.19	0.00	-0.19	0.01	0.21	0.41
4	Not Changed	S-mmu-mir-101b	0.10	-0.03	0.00	0.03	0.08	0.13	0.18
4	Not Changed	S-mmu-mir-101a	-0.01	-0.03	0.02	0.06	0.09	0.13	0.17
4	Not Changed	S-mmu-mir-100	-0.02	-0.04	-0.01	0.02	0.09	0.16	0.23
4	Not Changed	S-mmu-let-7i	0.11	-0.01	-0.06	-0.11	0.07	0.25	0.43
4	Not Changed	S-mmu-let-7g	0.18	0.18	0.05	-0.08	0.03	0.15	0.26
4	Not Changed	S-mmu-let-7f-2	0.02	0.00	-0.01	-0.02	0.52	1.06	1.60
4	Not Changed	S-mmu-let-7f-1	0.11	0.04	-0.01	-0.07	0.55	1.16	1.77
4	Not Changed	S-mmu-let-7c-2	0.01	0.02	-0.02	-0.05	0.03	0.12	0.20
4	Not Changed	S-mmu-let-7c-1	-0.07	-0.06	-0.04	-0.03	0.05	0.12	0.19
4	Not Changed	S-mmu-let-7b	0.08	-0.03	0.13	0.29	0.31	0.33	0.34
4	Not Changed	S-mmu-let-7a-2	-0.04	0.23	0.46	0.70	0.57	0.44	0.31
4	Not Changed	S-mmu-let-7a-1	0.13	0.20	0.07	-0.05	0.11	0.26	0.42
