# Supplementary material for: Novel MicroRNA Candidates and miRNA-mRNA Pairs in Embryonic Stem (ES) Cells
Source: PLoS One. 2008 Jul 2;3(7):e2548. doi: 10.1371/journal.pone.0002548 (PMC2481296; doi:10.1371/journal.pone.0002548)
Supplement: Supplementary file 7 [file pone.0002548.s007.doc]

Sample: Mouse Assorted Total RNAs vs. GCNF-/- (0 day)

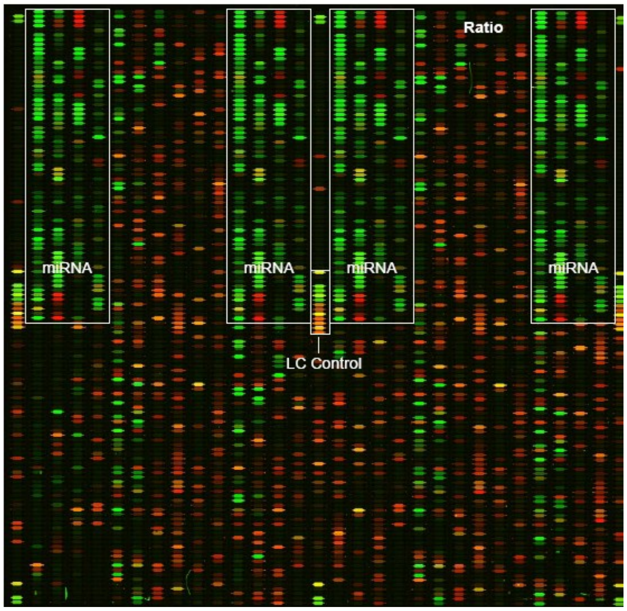

Supplemental Figure S7A: <u>Mouse Array Version 2 image.</u> The two samples used Adult Pool (Cy3) vs. GCNF -/- (Cy5). RNAs expressed at high levels in fully-differentiated adult tissue appear green (Cy3) and RNAs enriched in ES cells in which the orphan nuclear receptor GCNF has been knocked out (GCNF -/-) appear red (Cy5). The majority of miRNAs in miRBase (upper left-hand panel) appear green. The embryonic stem cell cluster (mmu-mir-290-295) appears red. The lower left-hand panel contains the “star (*) sequences” of the miRBase miRNAs (S-mmu-mir) and can be thought of as a sort of negative control, although some S-mmu-mir have been shown to have miRNA activity. The probes on the array are MCE-MIR (545), Cand and MIR occupy the rest of the array. The majority of MCE-MIR yield a strong red color.

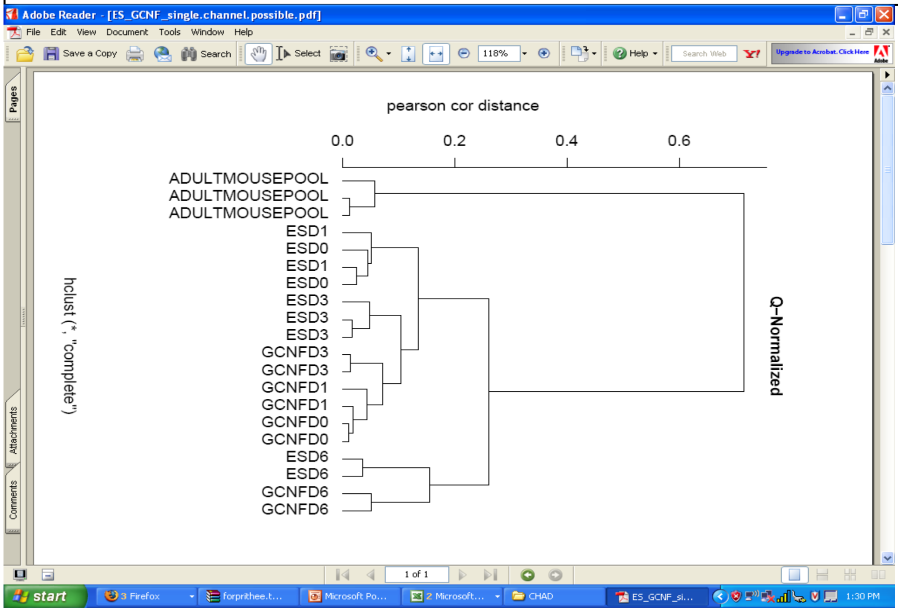

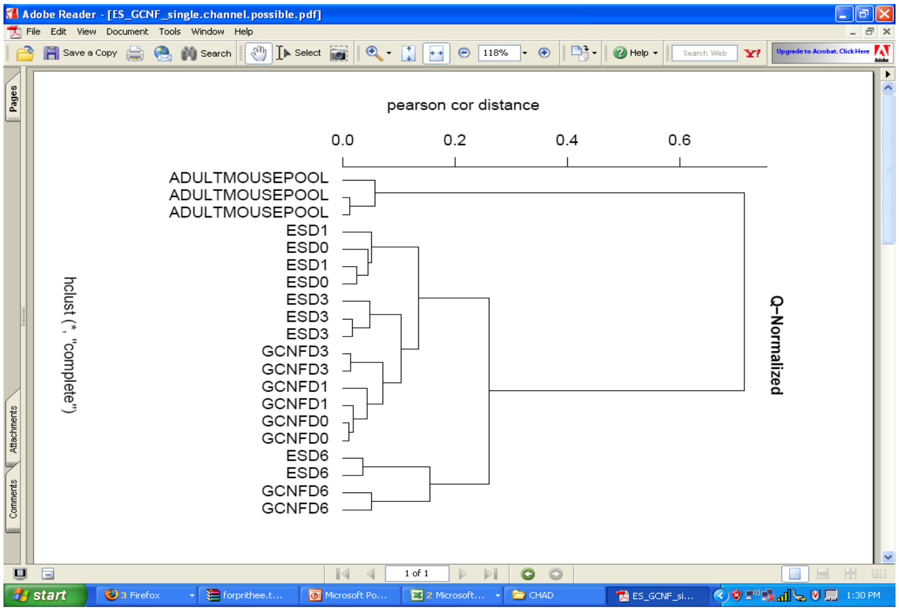

Supplemental Figure S7B: Hierarchical Clustering of 10 arrays. All microarray data are based on six probe replicates for each miRNA prediction (MCE-MIR, Cand and MIR) and eight probe replicates for mmu-mirs. ES and GCNF samples represent RNA isolated from pooled material from two independent 10cm dishes with ~ 75 million cells each from each time point of the ES and GCNF-/- time series experiment. For each time point, two dishes were pooled to harvest 0.5-1.0 mg of RNA for the arrays. Each time point is represented by duplicate arrays with dye swap of the label. The Adult Pool clearly separates as an independent group from ES and GCNF-/- ES Day 6 following retinoic acid treatment (ESD6) and the GCNF-/- Day 6 following retinoic acid treatment (GCNFD6) appear to be more related to each other as a separate group. During Day 0, Day 1 and Day 3 the ES cells appear to be different from GCNF-/- mutant during that period.
